# Brønsted Acid Catalyzed (4 + 2) Cyclocondensation of 3-Substituted Indoles with Donor–Acceptor Cyclopropanes

**DOI:** 10.1021/acs.orglett.1c00470

**Published:** 2021-03-09

**Authors:** Alesandere Ortega, Uxue Uria, Tomás Tejero, Liher Prieto, Efraim Reyes, Pedro Merino, Jose L. Vicario

**Affiliations:** † Department of Organic and Inorganic Chemistry, University of the Basque Country (UPV/EHU). P.O. Box 644, 48080 Bilbao, Spain; ‡ Instituto de Síntesis Química y Catálisis Homogénea (ISQCH), Universidad de Zaragoza, CSIC, 50009 Zaragoza, Spain; ¶ Instituto de Biocomputación y Física de Sistemas Complejos (BIFI), Universidad de Zaragoza, 50009 Zaragoza, Spain

## Abstract

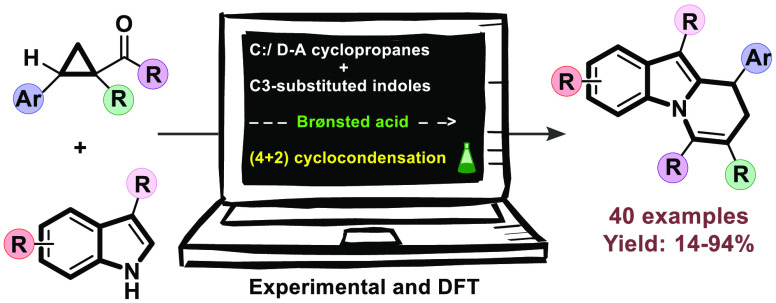

Acylcyclopropanes are employed as useful donor–acceptor cyclopropanes that undergo formal (4 + 2) cyclocondensation with *N*-unprotected 3-substituted indoles in the presence of a Brønsted acid catalyst. The reaction involves the simultaneous alkylation of both the N and C-2 positions of the indole and provides access to the 8,9-dihydropyrido[1,2-*a*]indole scaffold that is the central core of several biologically relevant indole alkaloids in excellent yields and good selectivities.

Donor–acceptor cyclopropanes (DAC) have demonstrated to be very useful functionalized reagents in modern organic synthesis.^1^ These compounds have an enhanced tendency to undergo ring opening in the presence of an external reagent and/or a catalyst to release ring strain, which is also facilitated by the synergistic nature of the electron-withdrawing and electron-donating substituents that contributes to the stabilization of the zwitterionic species formed after the ring-opening event.^2^ Despite this chemistry being well-known for decades, the use of these particular strained reagents as suitable substrates for the construction of carbocyclic and heterocyclic scaffolds through formal cycloaddition chemistry has experienced a renaissance in the past few years.^3^ In particular, the chemical behavior of indoles when reacted with donor–acceptor cyclopropanes has been studied in detail by several research groups, showing that different products can be obtained depending on the reaction conditions or on the substitution pattern of the nucleophilic indole reagent (Scheme 1). In general, donor–acceptor cyclopropanes react with indoles providing the corresponding C2 or C3 alkylation products depending on whether substituents at these positions are already present or not at the starting indole reagent (Scheme 1a),^4^ or alternatively, they undergo dearomative (3 + 2) cycloaddition reaction leading to hexahydrocyclopenta[*b*]indoles (Scheme 1b).^5^ In all cases, the initial ring opening has been reported to be possible through either Lewis acids or strong Brønsted acids as promoters.

**Scheme 1:**
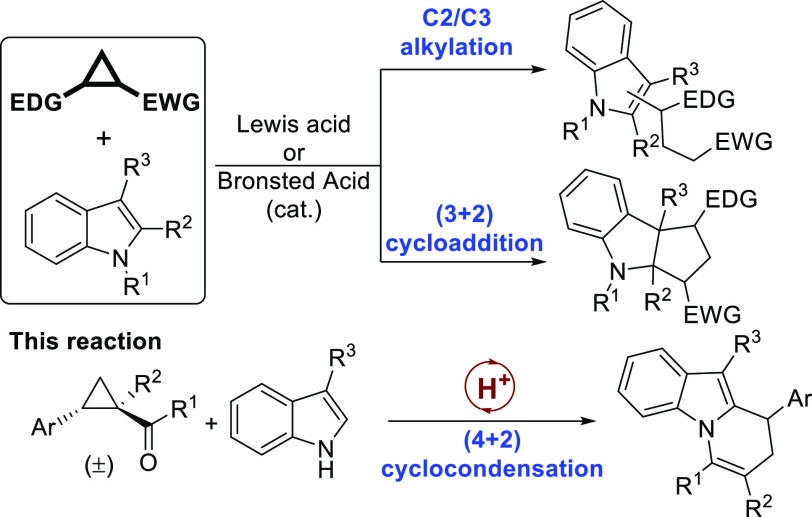
Reactivity of Indoles with DAC and the [4 + 2] Cyclocondensation Reaction Reported Herein

We wish to report herein the interesting alternative behavior observed when a donor–acceptor cyclopropane incorporating an acyl moiety as the electron-withdrawing group reacts with *N*-unprotected C3-substituted indoles under Brønsted acid catalysis (Scheme 1c). In this case, indole acts as a double nucleophile^6^ that, after C-2 alkylation, undergoes intramolecular condensation with the ketone moiety providing a (4 + 2) cyclocondensation product with a general 8,9-dihydropyrido[1,2-*a*]indole architecture present in the core structure of many natural occurring indole alkaloids with relevant biological activity.^7^ While there are many methods to access this scaffold,^8^ the approach shown herein is unconventional and provides multiple possibilities for the introduction of variable substitution patterns. There is only one previous example of a (4 + 2) cyclocondensation between indoles and donor–acceptor cyclopropanes, but in this case the reaction involves the subsequent C2 and C3 alkylation to form a carbazole derivative as the final adduct.^9^

We initially optimized the experimental conditions for the reaction to proceed in the most efficient way, using cyclopropane **1a**(10) and 3-methyl-1*H*-indole (**2a**) as model substrates (Table 1). We first evaluated the performance of diphenylphosphoric acid as catalyst, observing the formation of the expected cyclocondensation adduct **3a**(11) together with minor amounts of regioisomeric product **4a** (entry 1). This compound arises from the competitive participation of the indole as an *N*-nucleophile reacting with the carbocation formed after the acid-catalyzed ring opening. Other acid catalysts were next surveyed, observing that less acidic acetic acid was unable to promote the reaction (entry 2) but more acidic Brønsted acids led to the formation of products **3a** and **4a** in varying ratios with similar levels of chemical efficiency (entries 3–8).

**Table 1:**
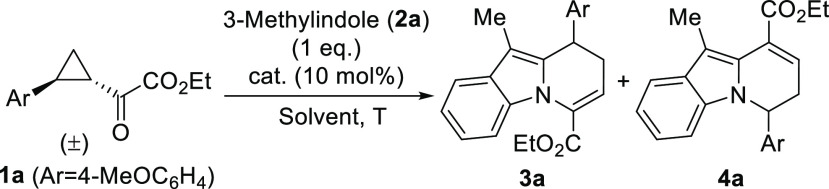
Optimization of the Reaction^a^

entry	catalyst	solvent	*T* (°C)	time (h)	yield (%)^b^	**3a**/**4a** (%)^c^
1	(PhO)_2_P(O)OH	Toluene	50	72	50	1.8/1
2	AcOH	Toluene	50	72	<5	n.d.^d^
3	(+)-CSA	Toluene	50	12	69	1/1
4	CF_3_CO_2_H	Toluene	50	24	39	2.5/1
5	*p*-TsOH	Toluene	50	12	59	1.3/1
6	(PhO)_2_P(O)NHTf	Toluene	50	12	61	2.5/1
7	NHTf_2_	Toluene	50	12	51	3.8/1
8	Concd. HCl (aq.)	Toluene	50	12	66	1.2/1
9	(PhO)_2_P(O)NHTf	THF	50	12	<5	n.d.^d^
10	(PhO)_2_P(O)NHTf	CHCl_3_	50	12	63	2/1
11	(PhO)_2_P(O)NHTf	C_6_H_6_	50	12	61	2.5/1
12	(PhO)_2_P(O)NHTf	*m*-Xylene	50	12	59	2.5/1
13	(PhO)_2_P(O)NHTf	Toluene	r.t.	96	<5	n.d.^d^
14	(PhO)_2_P(O)NHTf	Toluene	100	2	60	5/1

a Reactions carried out with 0.05 mmol of **1a** and **2a**, using 10 mol % of catalyst in 0.25 mL of solvent until consumption of starting material.

b Combined yield of both regioisomers.

c Calculated by NMR analysis of crude reaction mixture.

d n.d. = not determined.

From all acids tested, *N*-trifluoromethanesulfonyl diphenylphosphoramide was found to provide the best results in terms of overall yield and regioselectivity (entry 6). Solvents of varying nature were tested with this catalyst, and it was observed that moving to a more polar solvent like THF suppressed the reaction (entry 9) while changing to chloroform (entry 10) or other arenes (entries 11–12) did not result in any significant improvement in the outcome of the reaction. Finally, the effect of the temperature was also evaluated (entries 13–14). Lower temperatures were observed to suppress the reaction, while at higher temperatures the reaction proceeded with better yield and regioselectivity, obtaining the best results when the reaction was carried out at 100 °C (entry 14).

With an optimized protocol in hand, we next evaluated the applicability of this new transformation and the possibilities offered by the two reaction partners to incorporate structural diversity. We started by surveying the performance of indoles with a variable substitution pattern both at the 3-position or at other positions within the aryl moiety in combination with cyclopropane **1a** (Table 2). As it can be seen in this table, a collection of 3-methylindole reagents with both electron-donating or electron-withdrawing substituents at the 5-, 6-, or 7-position provided the corresponding cyclocondensation products **3a**–**f** in good yields and with high selectivity (entries 1–6), only detecting the competitive formation of regioisomers **4a**–**f** in minor amounts in all cases. In addition, the reaction also demonstrated a wide scope with respect to the substituent placed at the 3-position of the indole reagent (entries 7–9), although the yield was significantly affected by the steric bulk of this substituent. Finally, indoles with functionalized side chains such as benzyl or allyl (entries 10 and 11) and 3-aryl indoles also exhibited high reactivity, providing the desired cyclocondensation products in very high yields and regioselectivities (entries 12–14).

**Table 2:**
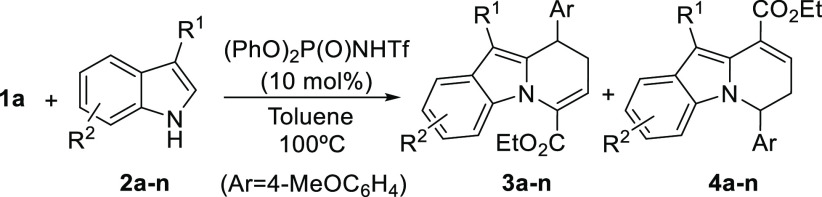
Scope of the Reaction: Indole Reagent^a^

entry	indole (**2**)	R^1^	R^2^	yield (%)^b^	**3**/**4** (%)^c^
1	**2a**	Me	H	59 (50)	5:1
2	**2b**	Me	7-Me	58 (54)	13:1
3	**2c**	Me	6-OMe	61 (61)	>20:1
4	**2d**	Me	6-Me	60 (52)	6.1:1
5	**2e**	Me	6-F	65 (60)	10:1
6	**2f**	Me	5-OMe	50 (42)	5:1
7	**2g**	Et	H	56 (46)	4.3:1
8	**2h**	^i^Pr	H	25 (25)	>20:1
9	**2i**	^t^Bu	H	14 (14)	>20:1
10	**2j**	Bn	H	60 (47)	3.4:1
11	**2k**	CH_2_CH=CH_2_	H	57 (36)	1.5:1
12	**2l**	Ph	H	69 (62)	7.6:1
13	**2m**	4-FC_6_H_4_	H	82 (73)	8:1
14	**2n**	4-MeOC_6_H_4_	H	85 (79)	11:1

a All reactions were carried out at 0.05 mmol scale of **1a** and **2a**–**n**, with 10 mol % of cat. in 0.25 mL of toluene until consumption of starting material.

b Combined yield of both regioisomers. Isolated yield of major adduct **3** is indicted in parentheses.

c Calculated by NMR analysis of crude reaction mixture.

We next evaluated other cyclopropane substrates (Table 3), starting with cyclopropyl ketones **1b**–**d**. These reacted with a variety of 3-substituted indoles. In all cases, the exclusive formation of adducts **5a**–**e** occurred without any *N*-addition byproduct (entries 1–5). We next surveyed cyclopropane **1e** that incorporates two electron-withdrawing substituents as a potentially more reactive substrate. Indeed, the reaction with **2a** led to the exclusive formation of product **5f** in excellent yield (entry 6) and also without the presence of the competitive *N*-addition regioisomer. Other 3-substituted indoles were tested, performing with a similar level of efficiency (entries 7–11). We also evaluated the tolerance of the reaction toward the introduction of substituents at the 5-, 6-, or 7-position of the indole core, and in all cases, the reaction proceeded smoothly (entries 12–16). Changing the R^1^ substituent at the acyl moiety was also found to be possible, as seen with the excellent performance of the reaction that provided adducts **5q**(12) and **5r** (entries 17 and 18). In addition, the alkoxy substituent at the ester moiety of the cyclopropane reagent can also be changed from ethoxy to benzyloxy without any negative effect (entry 18). Remarkably, when cyclopropane **1h** was employed (entry 19**)**, the reaction took place together with spontaneous hydrolysis/decarboxylation, providing adduct **5s** in very high yield.

**Table 3:**
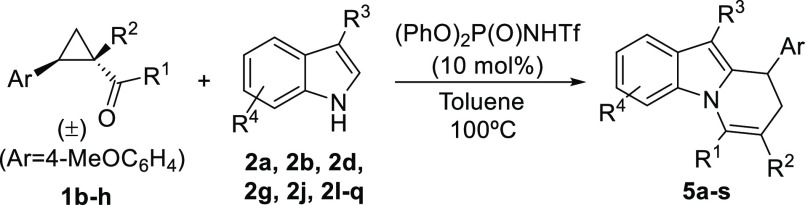
Scope of the Reaction: Cyclopropane Reagent^a^

entry	**1**	**2**	**5**	R^1^	R^2^	R^3^	R^4^	Yield (%)^b^
1	**1b**	**2a**	**5a**	4-NO_2_C_6_H_4_	H	Me	H	80
2	**1b**	**2m**	**5b**	4-NO_2_C_6_H_4_	H	4-FC_6_H_4_	H	80
3	**1b**	**2n**	**5c**	4-NO_2_C_6_H_4_	H	4-MeOC_6_H_4_	H	92
4	**1c**	**2n**	**5d**	4-ClC_6_H_4_	H	4-MeOC_6_H_4_	H	90
5	**1d**	**2n**	**5e**	Ph	H	4-MeOC_6_H_4_	H	85
6	**1e**	**2a**	**5f**	Ph	CO_2_Et	Me	H	94
7	**1e**	**2g**	**5g**	Ph	CO_2_Et	Et	H	92
8	**1e**	**2j**	**5h**	Ph	CO_2_Et	Bn	H	81
9	**1e**	**2l**	**5i**	Ph	CO_2_Et	Ph	H	90
10	**1e**	**2n**	**5j**	Ph	CO_2_Et	4-MeOC_6_H_4_	H	86
11	**1e**	**2m**	**5k**	Ph	CO_2_Et	4-FC_6_H_4_	H	82
12	**1e**	**2b**	**5l**	Ph	CO_2_Et	Me	7-Me	54
13	**1e**	**2d**	**5m**	Ph	CO_2_Et	Me	6-Me	93
14	**1e**	**2o**	**5n**	Ph	CO_2_Et	Ph	6-MeO	79
15	**1e**	**2p**	**5o**	Ph	CO_2_Et	Ph	5-MeO	71
16	**1e**	**2q**	**5p**	Ph	CO_2_Et	Ph	6-F	87
17	**1f**	**2l**	**5q**	4-ClC_6_H_4_	CO_2_Et	Ph	H	90
18	**1g**	**2l**	**5r**	Me	CO_2_Bn	Ph	H	83
19^c^	**1h**	**2l**	**5s**	Ph	H	Ph	H	72

a All reactions were carried out at 0.05 mmol scale of **1** and **2**, using 10 mol % of catalyst in 0.25 mL of toluene until consumption of starting material.

b Isolated yield after purification.

c Starting from cyclopropane **1h** (R^1^ = Ph; R^2^ = CO_2_^t^Bu).

We also examined the scope of the reaction with respect to the possibility of incorporating different aryl substituents at the cyclopropane core different from the *p*-methoxyphenyl group used to date (Scheme 2). Almost all substrates tested cleanly furnished the expected cyclocondensation products in excellent yield regardless of the nature of the substituent (compounds **6a**–**d** and **6g**) and the position in which this was placed within the aryl substituent (compounds **6e**–**f**). Interestingly, both phenyl-substituted cyclopropane and *p*-bromophenyl-substituted substrate performed excellently in the reaction (products **6b**–**c**), showing that there is no full need for a strong electron-donating substituent at the cyclopropane scaffold. This event also opens the way to the use of related cyclopropanes without a clear donor–acceptor substitution pattern.

**Scheme 2:**
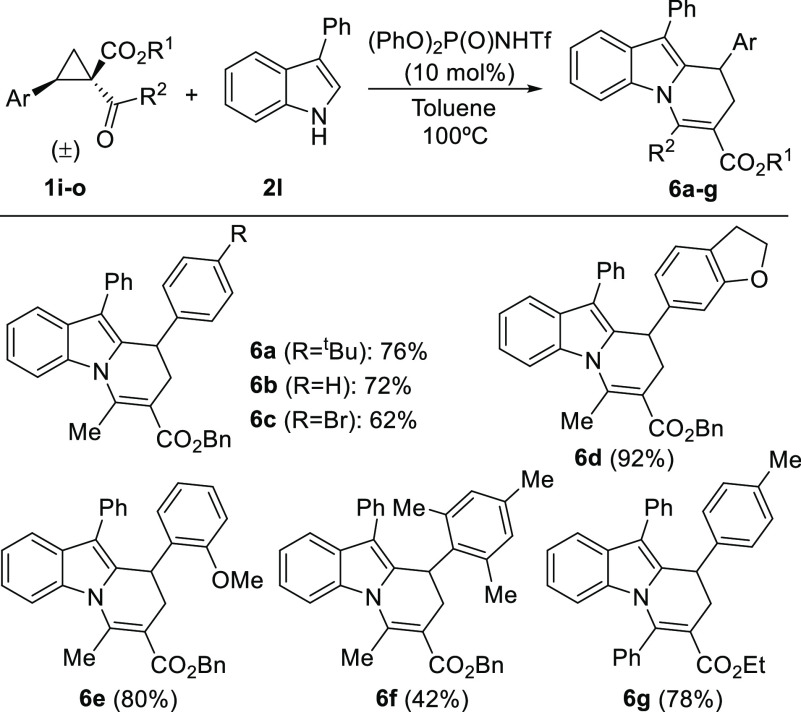
Use of Cyclopropanes with Different EDG

We studied the process in detail by DFT methods to provide a rationale of the observed results. We first studied the reaction of indole **2a** with cyclopropyl derivative **A** as a simplified analogue of substrate **1a**. At the same time, we also evaluated the reaction using model cyclopropane **B** that would lead selectively to the formation of product of general structure **5B**. Both attacks to C-2 and N of the indole moiety were considered^13^ leading to adducts of type **3A** (or **5B**) and **4A** respectively (Scheme 3). The C-attack consists of two steps, i.e., formation of intermediate **IN1A** and then **IN2A** after an H-transfer to recover indole aromaticity. Further cyclization of **IN2A** and dehydration lead to the final product. The alternative pathway leading to adducts of type **4A** involves the N-attack that results in the formation of **IN3A** (actually the direct product is the enol form; see Supporting Information (SI)) which through concomitant C2-attack to the carbonyl moiety and H-transfer yields **IN4A**, which after dehydration provides the final product. The calculated energies for these intermediates and the associated TS are also shown in Scheme 3. For both C-2 and N-attacks, the first step in which the nucleophile-induced cyclopropane ring opening takes place is the rate limiting step. For both cases **A** and **B**, the C-2 attack is preferred over the N-attack, which showed to be higher in energy, and this would explain the more selective formation of adduct **5B** for this particular type of highly activated donor–acceptor cyclopropanes. Several diastereomers can be formed during the process; the lower energy route has been considered in each case (for the complete study, see SI).

**Scheme 3:**
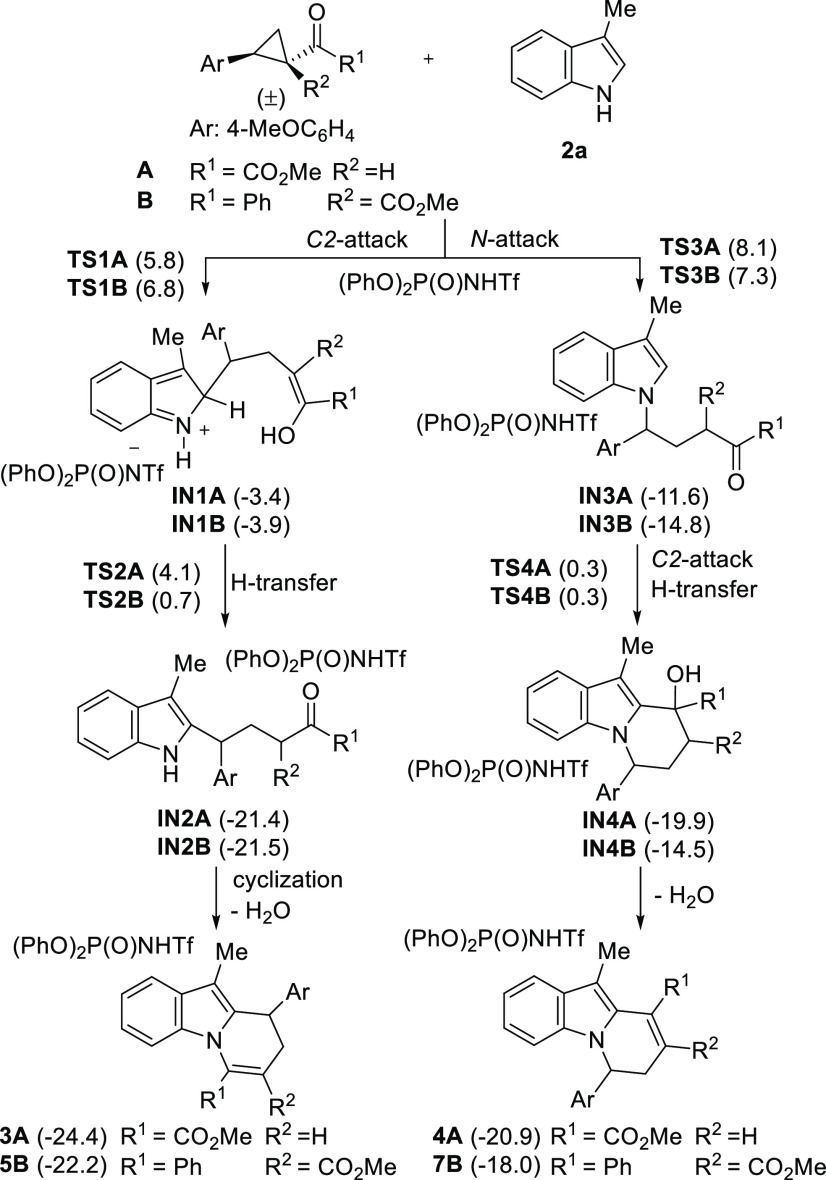
Two Favored Routes for the Reaction between Model Cyclopropanes A and B and 3-Methylindole **2a** and the Calculated Energy Profiles (Relative Free Energies Given in kcal/mol)

In conclusion, we have developed a Brønsted acid catalyzed procedure for performing an unexplored (4 + 2) cyclocondensation between donor–acceptor cyclopropanes and C3-substituted indoles. The methodology described herein presents a broad scope regarding both counterparts of the reaction, providing the corresponding 8,9-dihydropyrido[1,2-*a*]indoles in good yields and with an excellent level of selectivity. This reactivity pattern is particularly attractive, as it shows the alternative behavior of *N*-unprotected C3-substituted indoles, in which N and C-2 positions are simultaneously alkylated due to their double nucleophilic character and also forced by the presence of the C3-substituent of the indole that directs the initial alkylation step to the C2-position. Moreover, mechanistic investigations based on computational studies are in concordance with the observed experimental results.

## Supporting Information Available

Experimental procedures, characterization data of all new compounds and copies of ^1^H and ^13^C NMR spectra; reaction coordinates, computational details and Cartesian coordinates of all stationary points (PDF)

## Accession Codes

CCDC 2060969 contains the supplementary crystallographic data for this paper. These data can be obtained free of charge via www.ccdc.cam.ac.uk/data_request/cif, or by emailing data_request@ccdc.cam.ac.uk, or by contacting The Cambridge Crystallographic Data Centre, 12 Union Road, Cambridge CB2 1EZ, UK; fax: +44 1223 336033.
