# Supplementary material for: Brønsted Acid Catalyzed (4 + 2) Cyclocondensation of 3-Substituted Indoles with Donor–Acceptor Cyclopropanes
Source: Org Lett. 2021 Mar 9;23(6):2326–31. doi: 10.1021/acs.orglett.1c00470 (PMC9490874; doi:10.1021/acs.orglett.1c00470)
Supplement: Supplementary file 1 — ol1c00470_si_001.pdf [file ol1c00470_si_001.pdf]

Supporting information

Brønsted Acid-Catalyzed (4+2) Cyclocondensation of 3-Substituted Indoles with Donor-Acceptor Cyclopropanes

Alesandere Ortega,[†] Uxue Uria,*[,†] Tomás Tejero,[‡] Liher Prieto,[†] Efraim Reyes,[†] Pedro Merino*[,¶] and Jose L. Vicario*[,†]

[†] *Department of Organic and Inorganic Chemistry, University of the Basque Country (UPV/EHU). P.O. Box 644, 48080 Bilbao (Spain)*

[‡] *Instituto de Síntesis Química y Catálisis Homogénea (ISQCH), Universidad de Zaragoza-CSIC, 50009 Zaragoza (Spain)*

[¶] *Instituto de Biocomputación y Fisica de Sistemas Complejos (BIFI), Universidad de Zaragoza, 50009 Zaragoza (Spain)*

Table of contents

1 General methods and materials **S3**

2 Experimental procedures and characterization **S4**

2.1 Synthesis of donor-acceptor cyclopropanes 1a-o S4

2.1.1 Synthesis of acyl-substituted donor-acceptor cyclopropanes (1b-d) S4

2.1.2 Synthesis of cyclopropanes derived from β-ketoesters (1f, 1h-k, 1m-o) S6

2.2 Synthesis of indoles 2a-q S11

2.3 Cyclocondensation of 3-substituted indoles with donor-acceptor cyclopropanes S12

2.3.1 Synthesis of dihydropyridoindoles 3 and 4 S12

2.3.2 Synthesis of dihydropyridoindoles 5 S24

2.3.3 Synthesis of dihydropyridoindoles 6 S34

3 X-Ray Analysis **S39**

3.1 Compound 3a S39

4 NMR spectra **S43**

4.1 Acyl-substituted donor-acceptor cyclopropanes (1a-d) S43

4.2 Cyclopropanes derived from β-ketoesters (1e-o) S47

4.3 Dihydropyridoindoles 3 and 4 S58

4.4 Dihydropyridoindoles 5a-s S82

4.5 Dihydropyridoindoles 6a-g S101

1 General methods and materials [1]

NMR: Monodimensional and/or bidimensional nuclear magnetic resonance proton and carbon spectra (^{1}H NMR and ^{13}C NMR) were acquired at 25 ºC on a Bruker AC-300 spectrometer (300 MHz for ^{1}H and 75.5 MHz for ^{13}C) and a Bruker AC-500 spectrometer (500 MHz for ^{1}H and 125.7 MHz for ^{13}C) at the indicated temperature. Chemical shifts (δ) are reported in ppm relative to residual solvent signals ($CHCl_3$, 7.26 ppm for ^{1}H NMR, $CDCl_3$, 77.16 ppm for ^{13}C NMR) and coupling constants (*J*) in hertz (Hz). The following abbreviations are used to indicate the multiplicity in NMR spectra: s, singlet; d, doublet; t, triplet; q, quartet; app, apparent; m, multiplet; bs, broad signal. ^{13}C NMR spectra were acquired on a broad band decoupled mode using DEPT experiments (Distorsionless Enhancement by Polarization Transfer) for assigning different types of carbon environment. Selective n.O.e., NOESY, COSY, HSQC and HMBC experiments were acquired to confirm precise molecular configuration and to assist in deconvoluting complex multiplet signals.[2]

IR: Infrared spectra (IR) were measured in a Jasco FT/IR 4100 (ATR), in the interval between 4000 and 400 cm^{-1} with a 4 cm^{-1} resolution. Only characteristic bands are given in each case.

MS: Mass spectra (MS) were recorded on an Agilent 7890A gas chromatograph coupled to an Agilent 5975C quadrupole mass spectrometer under electronic impact ionization (EI) at 70 eV. The obtained data is presented in mass units (m/z) and the values found in brackets belong to the relative intensities comparing to the base peak (100%).

HRMS (ESI): High-resolution mass spectra were recorded on an Acquity UPLC coupled to a QTOF mass spectrometer (SYNAPT G2 HDMS) using electrospray ionization (ESI^{+}).

HPLC: The enantiomeric ratio (er) of the products was determined by High Performance Liquid Chromatography on a chiral stationary phase in a Waters 2695 chromatograph coupled to a Waters 2998 photodiode array detector. Daicel *Chiralpak IA, IC, AD-H, AS-H, AZ-3* and *Chiralcel OZ-3* columns (0.46 × 25 cm) were used; specific conditions are indicated for each case.

M.p.: Melting points were measured in a Buchi B-540 apparatus in open capillary tubes and are uncorrected.

Optical rotations $[\alpha]_D^{20}$ were measured at 20 ºC on a Jasco P-2000 polarimeter with a sodium lamp at 589 nm and a path of length of 1 dm. Solvent and concentration are specified in each case.

X-ray: Data collections were performed using an Agilent Supernova diffractometer equipped with an Atlas CCD area detector, and a CuKα micro-focus source with multilayer optics (λ = 1.54184Å, 250µm FWHM beam size). The sample was kept at 150 K with an Oxford Cryosystems Cryostream 700 cooler.

---

[1] SGIker technical support (MEC, GV/EJ and European Social Fund) is gratefully acknowledged (NMR, HRMS and X-ray analysis)

[2] M. Kinss, J. K. M. Sanders, *J. Mag. Res.* **1984**, *56*, 518.

The quality of the crystals was checked under a polarizing microscope, and a suitable crystal or fragment was mounted on a Mitegen Micromount™ using Paratone N inert oil and transferred to the diffractometer.

Miscellaneous: Analytical grade solvents and commercially available reagents were used without further purification. Anhydrous solvents were purified and dried with activated molecular sieves prior to use.[3] For reactions carried out under inert conditions, the argon was previously dried through a column of P_2O_5 and a column of KOH and $CaCl_2$. All the glassware was dried for 12 hours prior to use in an oven at 140 ºC, and allowed to cool under a dehumidified atmosphere.[4] Reactions at reduced temperatures were carried out using a Termo Haake EK90 refrigerator. Reactions were monitored using analytical thin layer chromatography (TLC), in pre-coated silica-backed plates (Merck Kiesegel 60 F254). These were visualized by ultraviolet irradiation, *p*-anisaldehyde, phosphomolybdic acid or potassium permanganate dips.[5] For flash chromatography Silicycle 40-63, 230-400 mesh silica gel was used.[6] For the removal of the solvents under reduced pressure Büchi R-2 series rotatory evaporators were used.

2 Experimental procedures and characterization

2.1 Synthesis of donor-acceptor cyclopropanes 1a-o

Compounds **1a**[7], **1e**[7], **1g**[7] and **1l**[7] were prepared following procedures previously described in the literature.

2.1.1 Synthesis of acyl-substituted donor-acceptor cyclopropanes (1b-d)

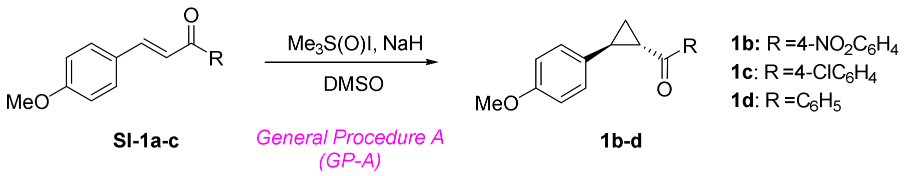

Scheme S1. General Procedure A for the synthesis of ketone substituted cyclopropanes 1b-d

---

[3] (a) W. L. F. Armarego, C. L. L. Chai, *Purification of Laboratory Chemicals*, 7th ed.; Elsevier: Oxford, 2012. (b) D. B. G. Williams, M. Lawton, *J. Org. Chem.* **2010**, *75*, 8351.
[4] G. W. Kramer, A. B. Levy, M. M. Midland, *Organic Synthesis via Boranes*, John Wiley & Sons, New York, 1975.
[5] E. Stahl, *Thin Layer Chromatography*, Springer Verlag: Berlin, 1969.
[6] W. C. Still, H. Kahn, A. J. Mitra, *J. Org. Chem.* **1978**, *43*, 2923.
[7] Ortega, A.; Manzano, R.; Uria, U.; Carrillo, L.; Reyes, E.; Tejero, T.; Merino, P.; Vicario, J. L., *Angew. Chem. Int. Ed.*, **2018**, *57*, 8225.

Alkenes **SI-1a**[8]**, SI-1b**,[9] and **SI-1c**,[10] were reported compounds and were prepared following the procedures described in the literature.

General Procedure A (GP-A) for the synthesis of ketone substituted cyclopropanes 1b-d. DMSO (0.24M) was added to a mixture of trimethylsulfoxonium iodide (1.2 eq.) and sodium hydride (1.2 eq.) and the mixture was stirred at room temperature for 30 minutes. Then, the corresponding alkene (1.0 eq.) in DMSO (0.5M) was added to the solution at 0 ºC and the solution was stirred for 2h at 60 ºC in an oil bath. The reaction was quenched by adding saturated aqueous NH_4Cl solution and was extracted with ethyl acetate (3 x 20 mL). The combined organic phases were washed with saturated aqueous solution of NaCl and dried over Na_2SO_4. Removal of the volatile substances under reduced pressure followed by purification by column chromatography on silica gel gave the corresponding cyclopropanes **1b-d**.

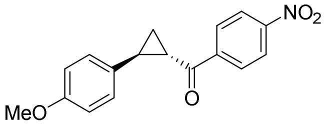

(±)-1b

(±)-*trans*-[2-(4-Methoxyphenyl)cyclopropyl](4-nitrophenyl)methanone (1b). Following *GP-A*, **1b** (416 mg, 1.4 mmol, 40%) was isolated by FC (petroleum ether/EtOAc, 19:1) on silica gel as a yellow solid, starting from **SI-1a** (1.0 g, 3.5 mmol, 1.0 eq.) in DMSO (7.0 mL) and the mixture of trimethylsulfoxonium iodide (0.92 g, 4.2 mmol, 1.2 eq.) and NaH (100 mg, 4.2 mmol, 1.2 eq.) in DMSO (17.5 mL). R_f= 0.50 (petroleum ether/EtOAc, 19:1). **^{1}H NMR** (300 MHz, $CDCl_3$) δ 8.26 (d, *J* = 9.0 Hz, 2H, C_{arom}-H), 8.10 (d, *J* = 9.0 Hz, 2H, C_{arom}-H), 7.11 (d, *J* = 8.7 Hz, 2H, C_{arom}-H), 6.85 (d, *J* = 8.7 Hz, 2H, C_{arom}-H), 3.78 (s, 3H, OCH_3), 2.82 (ddd, *J* = 7.9, 5.2, 3.9 Hz, 1H, CH-PMP), 2.72 (ddd, *J* = 8.8, 6.8, 3.9 Hz, 1H, CHC=O), 1.95 (ddd, *J* = 8.8, 5.2, 4.1 Hz, 1H, C**H**$_a$CH$_b$), 1.67-1.56 (m, 1H, CH$_a$C**H**$_b$). **^{13}C NMR** (75 MHz, $CDCl_3$) δ 197.2 (C=O), 158.7 (**C**$_{arom}$-OCH_3), 150.2 (C_{arom}-NO_2), 142.3 (C_{arom}-C), 131.7 (C_{arom}-C), 129.1 (2×C_{arom}-H), 127.4 (2×C_{arom}-H), 123.8 (2×C_{arom}-H), 114.2 (2×C_{arom}-H), 55.4 (OCH_3), 31.0 (**C**HC=O), 30.1 (C_{arom}**C**HCH_2), 19.8 (CH_2). **IR** (ATR): 2934 (C-H st), 1671 (C=O st), 1515 (NO_2 st as), 1341 (NO_2 st sim) cm^{-1}. **HRMS (ESI) m/z:** $[M+H]^+$ Calcd for $C_{17}H_{16}NO_4$ 298.1079; Found 298.1084. **M.p.**: 61-63 ºC (petroleum ether/EtOAc).

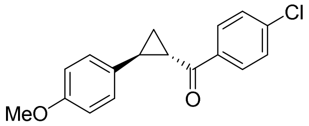

(±)-1c

(±)-*trans*-(4-Chlorophenyl)[2-(4-methoxyphenyl)cyclopropyl]methanone (1c). Following *GP-A*, **1c** (889 mg, 3.1 mmol, 56%) was isolated by FC (petroleum ether/EtOAc, 19:1) on silica gel as a white solid, starting from **SI-1b** (1.5 g, 5.5 mmol, 1.0 eq.) in DMSO (11.0 mL) and the mixture of trimethylsulfoxonium iodide (1.4 g, 6.6 mmol, 1.2 eq.) and NaH (158 mg, 6.6 mmol, 1.2 eq.) in DMSO (27.5 mL). R_f= 0.50 (petroleum ether/EtOAc, 19:1). **^{1}H NMR** (300 MHz, $CDCl_3$) δ 7.93 (d, *J* = 8.6 Hz, 2H, C_{arom}-H), 7.42 (d, *J* = 8.6 Hz, 2H, C_{arom}-H), 7.11 (d, *J* = 8.6 Hz, 2H, C_{arom}-H), 6.86 (d, *J* = 8.6 Hz, 2H, C_{arom}-H), 3.80 (s, 3H, OCH_3), 2.77 (ddd, *J* = 7.9, 5.2, 4.0 Hz, 1H, CH-PMP), 2.67 (ddd, *J* = 9.0, 6.7, 4.0 Hz, 1H, CHC=O), 1.91 (ddd, *J* = 9.0, 5.2, 4.1 Hz, 1H ,C**H**$_a$CH$_b$), 1.54 (ddd, *J* = 7.9, 6.7, 4.1

---

[8] Gaikwad, S.; Goswami, A.; De, S.; Schmittel, M. *Angew. Chem Int. Ed.* **2016**, *55*, 10512.

[9] Kumar, R.; Mohanakrishnan, D.; Sharma, A.; Kaushik, N. K.; Kalia, K.; Sinha, A. K.; Sahal, D. *Eur. J. Med. Chem.* **2010**, *45*, 5292.

[10] Downey, C. W.; Glist, H. M.; Takashima, A.; Bottum, R. S.; Dixon, G. J. *Tetrahedron Lett.* **2018**, *59*, 3080.

Hz, 1H, CH_aCH_b). **^{13}C NMR** (75 MHz, $CDCl_3$) δ 197.5 (C=O), 158.6 (**C**$_{arom}$-OCH_3), 139.4 (C_{arom}-C), 136.2 (C_{arom}-C), 132.3 (C_{arom}-C), 129.6 (2×C_{arom}-H), 129.0 (2×C_{arom}-H), 127.5 (2×C_{arom}-H), 114.2 (2×C_{arom}-H), 55.4 (OCH_3), 30.0 ($C_{arom}CHCH_2$), 29.3 (**C**HC=O), 19. (CH_2). **IR** (ATR): 2937 (C-H st), 1664 (C=O st), 1032 (C-Cl st) cm^{-1}. **MS** (EI) m/z (%): 286.0 (M^+, 17), 147.0 (100), 138.9 (60), 115.0 (30), 113.0 (18), 111.0 (48), 103.0 (22), 91.0 (35), 78.0 (35), 78.0 (20), 77.0 (22), 75.0 (27). **HRMS (ESI) m/z:** $[M+H]^+$ Calcd for $C_{17}H_{16}O_2Cl$ 287.0839; Found 287.0839. **M.p.**: 71-74 ºC (petroleum ether/EtOAc).

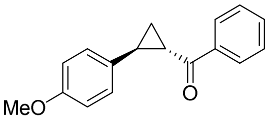

(±)-1d

(±)-*trans*-[2-(4-Methoxyphenyl)cyclopropyl](phenyl)methanone (1d). Following *GP-A*, **1d** (378 mg, 1.5 mmol, 71%) was isolated by FC (petroleum ether/EtOAc, from 19:1 to 9:1) on silica gel as a colorless oil, starting from **SI-1c** (0.50 g, 2.1 mmol, 1.0 eq.) in DMSO (4.2 mL) and the mixture of trimethylsulfoxonium iodide (0.55 g, 2.5 mmol, 1.2 eq.) and NaH (60 mg, 2.5 mmol, 1.2 eq.) in DMSO (10.4 mL). R_f= 0.60 (petroleum ether/EtOAc, 19:1). **^{1}H NMR** (300 MHz, $CDCl_3$) δ 8.01 (dd, *J* = 8.2, 1.4 Hz, 2H, C_{arom}-H), 7.63-7.53 (m, 1H, C_{arom}-H), 7.51-7.40 (m, 2H, C_{arom}-H), 7.13 (d, *J* = 8.7 Hz, 2H, C_{arom}-H), 6.87 (d, *J* = 8.7 Hz, 2H, C_{arom}-H), 3.81 (s, 3H, OCH_3), 2.85 (ddd, *J* = 7.8, 5.2, 4.0 Hz, 1H, CH-PMP), 2.68 (ddd, *J* = 8.9, 6.6, 4.0 Hz, 1H, CHC=O), 1.92 (ddd, *J* = 8.9, 5.2, 4.0 Hz, 1H, **C**H$_a$CH$_b$), 1.53 (ddd, *J* = 7.8, 6.6, 4.0 Hz, 1H, CH$_a$**C**H$_b$). **^{13}C NMR** (75 MHz, $CDCl_3$) δ 198.7 (C=O), 158.6 (**C**$_{arom}$-OCH_3), 137.9 (C_{arom}-C), 132.9 (C_{arom}-H), 132.6 (C_{arom}-C), 128.7 (2×C_{arom}-H), 128.2 (2×C_{arom}-H), 127.5 (2×C_{arom}-H), 114.1 (2×C_{arom}-H), 55.4 (OCH_3), 29.8 (C_{arom}**C**HCH$_2$), 29.3 (**C**HC=O), 19.0 (CH_2). **IR** (ATR): 2934 (C-H st), 1664 (C=O st) cm^{-1}. **MS** (EI) m/z (%): 252.1 (M^+, 30), 250.1 (29), 147.0 (41), 115.0 (24), 105.0 (70), 103.0 (21), 91.0 (24), 78.0 (29), 77.0 (100), 51.0 (31). **HRMS (ESI) m/z:** $[M+H]^+$ Calcd for $C_{17}H_{17}O_2$ 253.1229; Found 253.1228.

2.1.2 Synthesis of cyclopropanes derived from β-ketoesters (1f, 1h-k, 1m-o)

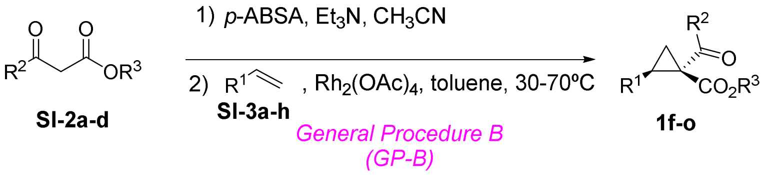

1f: R^1 = 4-OMe-C_6H_4, R^2 = 4-Cl-C_6H_4, R^3 = Et
1h: R^1 = 4-OMe-C_6H_4, R^2 = Ph, R^3 = *t*-Bu
1i: R^1 = 4-*t*-Bu-C_6H_4, R^2 = Me, R^3 = Bn
1j: R^1 = Ph, R^2 = Me, R^3 = Bn
1k: R^1 = 4-Br-C_6H_4, R^2 = Me, R^3 = Bn
1m: R^1 = 2-OMe-C_6H_4, R^2 = Me, R^3 = Bn
1n: R^1 = 2,4,6-Me_3-C_6H_2, R^2 = Me, R^3 = Bn
1o: R^1 = 4-Me-C_6H_4, R^2 = Ph, R^3 = Et

Scheme S2. *General Procedure B* for the synyhesis of cyclopropanes derived from β-ketoesters

Acetoacetates **SI-2b-d** were obtained from commercial sources. Intermediate **SI-2a**[11] was prepared following the procedures described in the literature.

[11] Katritzky, A. R.; Wang, Z.; Wang, M.; Wilkerson, C. R.; Hall, C. D.; Akhmedov, N. G. *J. Org. Chem.* **2004**, *69*, 6617.

Vinylarenes **SI-3a-d** and **SI-3f-g** were obtained from commercial sources. Vinylarene **SI-3e**[12] was a reported compound and it was prepared following the procedure described in the literature.

General Procedure B (GP-B) for the synthesis of donor-acceptor cyclopropanes (**1f, 1h-k, 1m-o*****):*** Triethylamine (1.5 eq.) was added dropwise to a solution of the corresponding β-ketoester **SI-2a-d** (1.0 eq.) and 4-acetamidobenzenesulfonyl azide (*p*-ABSA) (1.2 eq.) in acetonitrile (0.13M), at 0 ºC. The resulting mixture was stirred at room temperature for 8-12 hours, time during which the corresponding sulfonamide precipitated. Volatiles were evaporated, the residue was suspended in CH_2Cl_2, silica gel was added and solvent was evaporated. The resulting solid mixture was loaded onto a pad of silica and eluted with petroleum ether/EtOAc (10:1) to provide the corresponding diazocompound. Benzyl 2-diazo-3-oxobutanoate[13] was synthesized in 10 g-scale[14] and used for the synthesis of cyclopropanes **1i-k** and **1m-n**. The rest of diazocompounds were immediately used in the next step without further purification. The catalyst $Rh_2(OAc)_4$ (0.3-2 mol%) was added to a solution of the corresponding crude diazocompound (1.0 eq.) and vinylarene **SI-3a-o** in dry toluene or dichloromethane (0.15-1 M) and the mixture was stirred between 30-70 ºC in an oil bath. After completion of the reaction, the mixture was quenched with H_2O. The aqueous phase was extracted with EtOAc (2×20 mL) and the combined organic layers were washed with brine (2×20 mL), dried over anhydrous Na_2SO_4, and purified by flash column chromatography to obtain the pure cyclopropane.

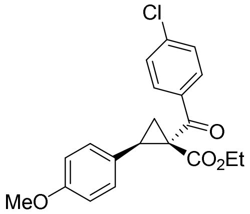

(±)-1f **Ethyl (±)-*cis*-1-(4-chlorobenzoyl)-2-(4-methoxyphenyl)cyclopropane-1-carboxylate (1f).** Following *GP-B*, **1f** (2.08 g, 5.8 mmol, 58%, dr > 20:1) was isolated by FC (petroleum ether/EtOAc, from 19:1 to 9:1) on silica gel as white solid starting from ethyl 4-chlorobenzoylacetate (2.27 g, 10.0 mmol, 1.0 eq.) and *p*-ABSA (2.64 g, 11.0 mmol, 1.1 eq.) in CH_3CN (25 mL) using Et_3N (4.2 mL, 30.0 mmol, 3.0 eq.). After dissolving the crude ethyl 2-diazo-3-oxo-3-(4-chlorophenyl)propanoate (2.09 g, 8.3 mmol, 1.0 eq.) and 4-methoxystyrene (2.8 mL, 20.7 mmol, 2.5 eq.) in toluene (8.3 mL), $Rh_2(OAc)_4$ (11 mg, 0.026 mmol, 2 mol%) was added. R_f= 0.60 (petroleum ether/EtOAc, 9:1). **^{1}H NMR** (300 MHz, $CDCl_3$) δ 7.84 (d, *J* = 8.6 Hz, 2H, C_{arom}-H), 7.42 (d, *J* = 8.6 Hz, 2H, C_{arom}-H), 7.22 (d, *J* = 8.5 Hz, 2H, C_{arom}-H), 6.83 (d, *J* = 8.7 Hz, 2H, C_{arom}-H), 3.78 (s, 3H, OCH_3), 3.77-3.66 (m, 2H, OCH_2), 3.50 (app t, *J* = 8.6 Hz, 1H, C**H**CH_2), 2.39 (dd, *J* = 8.1, 4.8 Hz, 1H, CHC$\mathbf{H_a}H_b$), 1.66 (dd, *J* = 9.2, 4.8 Hz, 1H, CHC$H_a\mathbf{H_b}$), 0.74 (t, *J* = 7.1 Hz, 3H, $CH_2C\mathbf{H_3}$). **^{13}C NMR** (75 MHz, $CDCl_3$) δ 193.9 (C_{arom}**C**=O), 168.4 (COO), 158.9 (**C**$_{arom}$-OCH_3), 139.3 (C_{arom}-Cl), 135.9 (**C**$_{arom}$-CO), 130.3 (2×C_{arom}-H), 129.7 (2×C_{arom}-H), 128.9 (2×C_{arom}-H), 126.6 (C_{arom}-C), 113.6 (2×C_{arom}-H), 61.3 (OCH_2), 55.3 (OCH_3), 42.3 (**C**CH_2), 30.5 (**C**HCH_2), 20.4 (CH**C**H_2), 13.7 (CH_2**C**H_3). **IR** (ATR): 1726 (C=O st), 1673 (C=O st), 1176 (C-O st as), 1086 (C-Cl) cm^{-1}. **MS** (EI) m/z (%): 358.1 (M^+, 15), 356.0 (16), 312.0

[12] Li, R.; Chen, X.; Song, X.-R.; Ding, H.; Wang, P.; Xiao, Q.; Liang, Y.-M. *Adv. Synth. Catal.* **2017**, *359*, 3962.
[13] Meyer, M. E.; Ferreira, E. M.; Stoltz, B. M. *Chem. Commun.* **2006**, 1316.
[14] From benzyl acetoacetate (10.0 g, 52.0 mmol, 1.0 eq.) and *p*-ABSA (15.0 g, 62.4 mmol, 1.2 eq.) in CH_3CN (370 mL), and Et_3N (10.9 mL, 78.0 mmol, 1.5 eq.), to afford bezyl 2-diazo-3-oxobutanoate in >90% yield.

(24), 200.0 (43), 144.9 (21), 141.0 (32), 139.0 (100), 111.0 (37). **HRMS (ESI) m/z:** $[M+Na]^+$ Calcd for $C_{20}H_{19}O_4ClNa$ 381.0870; Found 381.0882. **M.p.**: 89-92 ºC (petroleum ether/EtOAc).

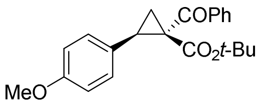

(±)-1h

***tert*-Butyl (±)-*cis*-1-benzoyl-2-(4-methoxyphenyl)cyclopropane-1-carboxylate (1h).** Following *GP-B*, **1h** (938 mg, 2.7 mmol, 72%, dr 2.5:1) was isolated by FC (petroleum ether/EtOAc, from 19:1 to 9:1) on silica gel as yellow oil starting from *tert*-butyl benzoylacetate (815 mg, 3.7 mmol, 1.0 eq.) and *p*-ABSA (1.1 g, 4.4 mmol, 1.2 eq.) in CH_3CN (26 mL) using Et_3N (0.8 mL, 5.5 mmol, 1.5 eq.). After dissolving the crude *tert*-butyl 2-diazo-3-oxo-3-phenylpropanoate (920 mg, 3.7 mmol, 1.0 eq.) and 4-methoxystyrene (2.5 mL, 18.5 mmol, 5.0 eq.) in toluene (3.7 mL), $Rh_2(OAc)_4$ (32 mg, 0.07 mmol, 2 mol%) was added. R_f= 0.50 (petroleum ether/EtOAc, 8:2). **^{1}H NMR** (300 MHz, $CDCl_3$) (* indicates minor diastereoisomer resonances) δ 8.06-7.79 (m, 2H, C_{arom}-H), 7.73-7.62* (m, 2H, C_{arom}-H), 7.59-7.48* (m, 2H, C_{arom}-H), 7.50-7.27 (m, 3H, C_{arom}-H), 7.50-7.27* (m, 1H, C_{arom}-H), 7.34-7.18 (m, 2H, C_{arom}-H), 7.07* (d, J = 8.7 Hz, 2H, C_{arom}-H), 6.83 (d, J = 8.7 Hz, 2H, C_{arom}-H), 6.66* (d, J = 8.7 Hz, 2H, C_{arom}-H), 3.77 (s, 3H, OCH_3), 3.65* (s, 3H, OCH_3), 3.52 (app t, J = 8.6 Hz, 1H, C**H**CH_2), 3.43* (app t, J = 8.6 Hz, 1H, C**H**CH_2), 2.38* (dd, J = 4.9, 3.1 Hz, 1H, C**H**$_a$CH$_b$), 2.33 (dd, J = 8.0, 4.7 Hz, 1H, C**H**$_a$CH$_b$), 1.70* (dd, J = 9.2, 4.9 Hz, 1H, CH$_a$C**H**$_b$), 1.59 (dd, J = 9.1, 4.7 Hz, 1H, CH$_a$C**H**$_b$), 1.17* (s, 9H, $C(CH_3)_3$), 0.90 (s, 9H, $C(CH_3)_3$). **^{13}C NMR** (75 MHz, $CDCl_3$) (* indicates minor diastereoisomer resonances) δ 196.1 (C_{arom}-**C**=O), 193.6* (C_{arom}-**C**=O), 170.2* (COO), 167.3 (COO), 158.8 (**C**$_{arom}$-OCH_3), 158.8* (**C**$_{arom}$-OCH_3), 138.3 (C_{arom}-C), 138.3* (C_{arom}-C), 132.6 (C_{arom}-H), 132.3* (C_{arom}-H), 130.4 (2×C_{arom}-H), 129.2* (2×C_{arom}-H), 128.5 (2×C_{arom}-H), 128.4* (2×C_{arom}-H), 128.1 (2×C_{arom}-H), 128.1* (2×C_{arom}-H), 127.1 (C_{arom}-C), 126.2* (C_{arom}-C), 113.7* (2×C_{arom}-H), 113.5 (2×C_{arom}-H), 82.2* (**C**$(CH_3)_3$), 81.6 (**C**$(CH_3)_3$), 55.4 (OCH_3), 55.2* (OCH_3), 43.4 (**C**CH_2), 43.1* (**C**CH_2), 33.6* (**C**HCH_2), 29.8 (**C**HCH_2), 27.7* (C(**C**$H_3)_3$), 27.4 (C(**C**$H_3)_3$), 20.2 (CH**C**H_2), 18.2* (CH**C**H_2). **IR** (ATR): 1720 (C=O st), 1678 (C=O st), 1166 (C-O st as) cm^{-1}. **MS** (EI) m/z (%): 352.1 (M^+, 1), 278.0 (33), 277.0 (16), 250.1 (26), 235 (21), 200 (21), 147 (31) 105.0 (100), 77.0 (49), 56.1 (20). **HRMS (ESI) m/z:** $[M+Na]^+$ Calcd for $C_{22}H_{24}O_4Na$ 375.1572; Found 375.1569.

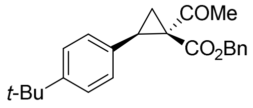

(±)-1i

Benzyl (±)-*cis*-1-acetyl-2-(4-(*tert*-butyl)phenyl)cyclopropane-1-carboxylate (1i). Following *GP-B*, **1i** (771 mg, 2.2 mmol, 42%, dr > 20:1) was isolated by FC (petroleum ether/EtOAc, from 19:1 to 9:1) on silica gel as a white solid starting from benzyl 2-diazo-3-oxobutanoate (1.1 g, 5.2 mmol, 1.0 eq.), 4-*tert*-butylstyrene (4.2 g, 26.0 mmol, 5.0 eq.), toluene (5.2 mL) and $Rh_2(OAc)_4$ (38 mg, 0.08 mmol, 2 mol%). R_f= 0.50 (petroleum ether/EtOAc, 8:2). **^{1}H NMR** (300 MHz, $CDCl_3$) δ 7.40-7.24 (m, 5H, C_{arom}-H), 7.19-7.08 (m, 2H, C_{arom}-H), 7.04-6.91 (m, 2H, C_{arom}-H), 4.86 (d, J = 12.1 Hz, 1H, OC**H$_a$**H$_b$), 4.68 (d, J = 12.1 Hz, 1H, OCH$_a$**H$_b$**), 3.29 (app t, J = 8.7 Hz, 1H, C**H**CH_2), 2.46 (s, 3H, CH_3C=O), 2.25 (dd, J = 8.4, 4.3 Hz, 1H, CHC**H$_a$**H$_b$), 1.78 (dd, J = 8.7, 4.2 Hz, 1H, CHCH$_a$**H$_b$**), 1.33 (s, 9H, $C(CH_3)_3$). **^{13}C NMR** (75 MHz, $CDCl_3$) δ 202.1 (CH_3**C**=O), 168.3 (COO), 150.3 ($C_{arom}C(CH_3)_3$), 135.0 (C_{arom}-C), 131.8 (C_{arom}-C), 128.5 (4×C_{arom}-H), 128.4 (2×C_{arom}-H), 128.3 (C_{arom}-H), 125.1 (2×C_{arom}-H), 67.2 (O**C**H_2), 44.7 (**C**CH_2), 35.6 (**C**H_3C=O), 34.5 (**C**$(CH_3)_3$), 31.4 (C(**C**$H_3)_3$), 29.7

(**C**HCH$_2$), 21.9 (CH**C**H$_2$). **IR** (ATR): 1728 (C=O st), 1691 (C=O st) cm^{-1}. **HRMS (ESI) m/z:** [M+H]$^+$ Calcd for $C_{23}H_{27}O_3$ 351.1960; Found 351.1967. **M.p.**: 48-51 ºC (petroleum ether/EtOAc).

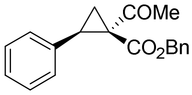

(±)-1j **Benzyl (±)-*cis*-1-acetyl-2-phenylcyclopropane-1-carboxylate (1j).** Following *GP-B*, **1j** (918 mg, 3.1 mmol, 60%, dr > 20:1) was isolated by FC (petroleum ether/EtOAc, from 19:1 to 9:1) on silica gel as yellow oil starting from benzyl 2-diazo-3-oxobutanoate (1.1 g, 5.2 mmol, 1.0 eq.), styrene (1.1 g, 10.4 mmol, 2.0 eq.), toluene (5.2 mL) and $Rh_2(OAc)_4$ (38 mg, 0.08 mmol, 2 mol%). R_f= 0.55 (petroleum ether/EtOAc, 8:2). **^{1}H NMR** (300 MHz, CDCl$_3$) δ 7.37-7.12 (m, 8H, C$_{arom}$-H), 7.09-6.91 (m, 2H, C$_{arom}$-H), 4.85 (d, *J* = 12.1 Hz, 1H, OC**H**$_a$CH$_b$), 4.70 (d, *J* = 12.1 Hz, 1H, OCH$_a$C**H**$_b$), 3.31 (app t, *J* = 8.6 Hz, 1H, C**H**CH$_a$H$_b$), 2.44 (s, 3H, CH$_3$), 2.25 (dd, *J* = 8.2, 4.6 Hz, 1H, CHC**H**$_a$H$_b$), 1.75 (dd, *J* = 9.2, 4.6 Hz, 1H, CHCH$_a$**H**$_b$). **^{13}C NMR** (75 MHz, CDCl$_3$) δ 202.2 (CH$_3$**C**=O), 168.3 (COO), 135.0 (C$_{arom}$-C), 134.9 (C$_{arom}$-C), 128.9 (2×C$_{arom}$-H), 128.6 (4×C$_{arom}$-H), 128.4 (C$_{arom}$-H), 128.3 (2×C$_{arom}$-H), 127.6 (C$_{arom}$-H), 67.3 (OCH$_2$), 44.8 (**C**C=O), 35.7 (**C**HCH$_2$), 29.8 (CH$_3$), 21.8 (CH**C**H$_2$). **IR** (ATR): 1728 (C=O st), 1691 (C=O st), 1166 (C-O st as) cm^{-1}. **MS** (EI) m/z (%): 203.0 (M$^+$-C$_7$H$_7$, 2), 115.0 (17), 91.1 (100), 77.0 (17). **HRMS (ESI) m/z:** [M+Na]$^+$ Calcd for: $C_{19}H_{18}O_3Na$ 317.1154; Found 317.1164.

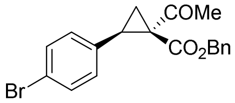

(±)-1k **Benzyl (±)-*cis*-1-acetyl-2-(4-bromophenyl)cyclopropane-1-carboxylate (1k).** Following *GP-B*, **1k** (2.3 g, 6.1 mmol, 59%, dr > 20:1) was isolated by FC (petroleum ether/EtOAc, from 19:1 to 9:1) on silica gel as white solid starting from 2-diazo-3-oxobutanoate (1.1 g, 5.2 mmol, 1.0 eq.), 4-bromostyrene (1.9 g, 10.4 mmol, 2.0 eq.), toluene (5.2 mL) and $Rh_2(OAc)_4$ (38 mg, 0.08 mmol, 2 mol%). R_f= 0.40 (petroleum ether/EtOAc, 8:2). **^{1}H NMR** (300 MHz, CDCl$_3$) δ 7.61-7.15 (m, 5H, C$_{arom}$-H), 7.13-6.76 (m, 4H, C$_{arom}$-H), 4.93 (d, *J* = 11.9 Hz, 1H, OC**H**$_a$CH$_b$), 4.80 (d, *J* = 11.9 Hz, 1H, OCH$_a$C**H**$_b$), 3.24 (app t, *J* = 8.6 Hz, 1H, C**H**CH$_a$H$_b$), 2.44 (s, 3H, CH$_3$), 2.19 (dd, *J* = 8.1, 4.7 Hz, 1H, CHC**H**$_a$H$_b$), 1.73 (dd, *J* = 9.1, 4.7 Hz, 1H, CHCH$_a$**H**$_b$). **^{13}C NMR** (75 MHz, CDCl$_3$) δ 201.9 (CH$_3$**C**=O), 168.0 (COO), 134.9 (C$_{arom}$-C), 133.9 (C$_{arom}$-C), 131.4 (2×C$_{arom}$-H), 130.6 (2×C$_{arom}$-H), 128.8 (2×C$_{arom}$-H), 128.7 (2×C$_{arom}$-H), 128.6 (C$_{arom}$-H), 121.6 (C$_{arom}$-C), 67.5 (OCH$_2$), 44.7 (**C**C=O), 34.6 (**C**HCH$_2$), 29.8 (C**C**H$_3$), 21.8 (CH**C**H$_2$). **IR** (ATR): 1725 (C=O st), 1693 (C=O st), 1166 (C-O st as) cm^{-1}. **MS** (EI) m/z (%): 371.9 (M$^+$, 1), 91.1 (100). **HRMS (ESI) m/z:** [M+Na]$^+$ Calcd for $C_{19}H_{17}BrO_3Na$ 395.0259; Found 395.0265. **M.p.**: 103-106 ºC (petroleum ether/EtOAc).

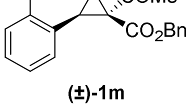

(±)-1m **Benzyl (±)-*cis*-1-acetyl-2-(2-methoxyphenyl)cyclopropane-1-carboxylate (1m).** Following *GP-B*, **1m** (1.3 g, 4.1 mmol, 78%, dr 5:1) was isolated by FC (petroleum ether/EtOAc, from 19:1 to 9:1) on silica gel as a colorless oil starting from benzyl 2-diazo-3-oxobutanoate (1.1 g, 5.2 mmol, 1.0 eq.), 2-methoxystyrene (3.6 mL, 26.0 mmol, 5.0 eq.), toluene (5.2 mL) and $Rh_2(OAc)_4$ (38 mg, 0.08 mmol, 2 mol%). R_f= 0.50 (petroleum ether/EtOAc, 9:1). **^{1}H NMR** (300 MHz, CDCl$_3$) (* indicates minor diastereoisomer resonances) δ 7.61-7.7.47* (m, 1H, C$_{arom}$-H), 7.42-7.36 (m, 1H, C$_{arom}$-H), 7.33-7.20 (m, 3H, C$_{arom}$-H), 7.10-6.98 (m, 3H, C$_{arom}$-H), 6.90-6.82 (m, 1H, C$_{arom}$-H), 6.76 (d, *J* = 8.2 Hz, 1H, C$_{arom}$-H), 5.31* (d, *J* = 12.5 Hz, 1H, OC**H**$_a$H$_b$), 5.25* (d, *J* = 12.4 Hz, 1H, OCH$_a$**H**$_b$), 4.85 (d, *J* = 12.1 Hz, 1H,

OC**H**$_a$H$_b$), 4.65 (d, *J* = 12.1 Hz, 1H, OCH$_a$**H**$_b$), 3.82* (s, 3H, OCH$_3$), 3.69 (s, 3H, OCH$_3$), 3.40* (app t, *J* = 8.7 Hz, 1H, C**H**CH$_2$), 3.13 (app t, *J* = 8.8 Hz, 1H, C**H**CH$_2$), 2.52 (s, 3H, CH$_3$C=O), 2.35* (dd, *J* = 8.6, 4.7 Hz, 1H, CHC**H**$_a$H$_b$), 2.20 (dd, *J* = 8.4, 4.5 Hz, 1H, CHC**H**$_a$H$_b$), 2.02* (s, 3H, CH$_3$C=O), 1.90 (dd, *J* = 9.1, 4.5 Hz, 1H, CHCH$_a$**H**$_b$), 1.72* (dd, *J* = 8.9, 4.7 Hz, 1H, CHCH$_a$**H**$_b$). **^{13}C NMR** (75 MHz, CDCl$_3$) (* indicates minor diastereoisomer resonances) δ 202.7 (CH$_3$**C**=O), 200.1* (CH$_3$**C**=O), 170.1* (COO), 168.5 (COO), 159.0 (**C**$_{arom}$-OCH$_3$), 158.6* (**C**$_{arom}$-OCH$_3$), 135.7* (C$_{arom}$-C), 135.3 (C$_{arom}$-C), 128.90 (C$_{arom}$-H), 128.86* (C$_{arom}$-H), 128.7 (C$_{arom}$-H), 128.6* (C$_{arom}$-H), 128.41 (2×C$_{arom}$-H), 128.39 (2×C$_{arom}$-H), 128.2 (C$_{arom}$-H), 128.0* (C$_{arom}$-H), 123.5 (C$_{arom}$-C), 122.4* (C$_{arom}$-C), 120.3* (C$_{arom}$-H), 120.0 (C$_{arom}$-H), 110.0 (C$_{arom}$-H), 109.7* (C$_{arom}$-H), 66.9* (OCH$_2$), 66.8 (OCH$_2$), 55.3 (OCH$_3$), 55.2* (OCH$_3$), 43.3 (**C**CH$_2$), 43.0* (**C**CH$_2$), 32.8 (**C**H$_3$C=O), 31.5* (**C**H$_3$C=O), 29.8 (**C**HCH$_2$), 29.6* (**C**HCH$_2$), 20.5 (CH**C**H$_2$), 17.5* (CH**C**H$_2$). **IR** (ATR): 1721 (C=O st), 1693 (C=O st), 1170 (C-O st as) cm^{-1}. **MS** (EI) m/z (%): 324.1 (M$^+$, 2), 91.1 (100), 77.0 (11), 65.1 (11). **HRMS (ESI) m/z:** [M+H]$^+$ Calcd for C$_{20}$H$_{21}$O$_4$ 325.1440; Found 325.1449.

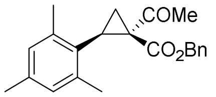

(±)-1n **Benzyl (±)-*cis*-1-acetyl-2-mesitylcyclopropane-1-carboxylate (1n).** Following *GP-B*, **1n** (523 mg, 1.5 mmol, 42%, dr > 20:1) was isolated by FC (petroleum ether/EtOAc, from 19:1 to 9:1) on silica gel as yellow oil starting from benzyl 2-diazo-3-oxobutanoate (0.8 g, 3.7 mmol, 1.0 eq.), 1,3,5-trimethyl-2-vinylbenzene (1.1 g, 7.4 mmol, 2.0 eq.), toluene (5.2 mL) and Rh$_2$(OAc)$_4$ (31 mg, 0.07 mmol, 2 mol%). R$_f$= 0.50 (petroleum ether/EtOAc, 8:2). **^{1}H NMR** (300 MHz, CDCl$_3$) δ 7.37-7.16 (m, 3H, C$_{arom}$-H), 7.09-6.88 (m, 2H, C$_{arom}$-H), 6.75 (s, 2H, C$_{arom}$-H), 4.84 (d, *J* = 12.0 Hz, 1H, OC**H**$_a$CH$_b$), 4.59 (d, *J* = 12.0 Hz, 1H, OCH$_a$C**H**$_b$), 3.16 (app t, *J* = 9.3 Hz, 1H, C**H**CH$_a$H$_b$), 2.59 (s, 3H, COCH$_3$), 2.45 (dd, *J* = 9.1, 4.3 Hz, 1H, CHC**H**$_a$H$_b$), 2.36-2.05 (m, 9H, 3× C$_{arom}$-CH$_3$), 1.99 (dd, *J* = 9.5, 4.3 Hz, 1H, CHCH$_a$**H**$_b$). **^{13}C NMR** (75 MHz, CDCl$_3$) δ 203.4 (CH$_3$**C**=O), 169.2 (COO), 136.5 (C$_{arom}$-C), 135.0 (C$_{arom}$-C), 129.3 (C$_{arom}$-H), 129.0 (C$_{arom}$-C), 128.8 (C$_{arom}$-C), 128.7 (2×C$_{arom}$-H), 128.6 (C$_{arom}$-H), 128.43 (2×C$_{arom}$-H), 128.40 (C$_{arom}$-C), 128.37 (C$_{arom}$-H), 67.2 (OCH$_2$), 42.7 (**C**C=O), 35.7 (**C**HCH$_2$), 30.3 (CO**C**H$_3$), 27.1 (3×C$_{arom}$-**C**H$_3$) 21.0 (CH**C**H$_2$). **IR** (ATR): 1715 (C=O st), 1691 (C=O st), 1176 (C-O st as) cm^{-1}. **MS** (EI) m/z (%): 336.2 (M$^+$, 3), 157.1 (21), 143.1 (15), 142.1 (16), 141.1 (19), 128.1 (20), 115.1 (17), 91.1 (100), 77.0 (17). **HRMS (ESI) m/z: [M+H]$^+$ Calcd for**: C$_{22}$H$_{25}$O$_3$ 336.1754; Found 336.1762.

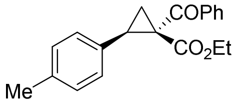

(±)-1o **Ethyl (±)-*cis*-1-benzoyl-2-(*p*-tolyl)cyclopropane-1-carboxylate (1o).** Following *GP-B*, **1o** (1.3 g, 4.2 mmol, 81%, dr > 20:1) was isolated by FC (petroleum ether/EtOAc, 9:1) on silica gel as white solid starting from ethyl benzoylacetate (1.0 g, 5.2 mmol, 1.0 eq.) and *p*-ABSA (1.5 g, 6.2 mmol, 1.2 eq.) in CH$_3$CN (37 mL) using Et$_3$N (1.1 mL, 7.8 mmol, 1.5 eq.). After dissolving the crude ethyl 2-diazo-3-oxo-3-phenylpropanoate (1.1 g, 5.2 mmol, 1.0 eq.) and 4-methylstyrene (3.4 mL, 26.0 mmol, 5.0 eq.) in toluene (5.2 mL), Rh$_2$(OAc)$_4$ (38 mg, 0.08 mmol, 2 mol%) was added. R$_f$= 0.50 (petroleum ether/EtOAc, 8:2). **^{1}H NMR** (300 MHz, CDCl$_3$) δ 7.91 (dd, *J* = 7.2, 1.0 Hz, 2H, C$_{arom}$-H), 7.62-7.51 (m, 1H, C$_{arom}$-H), 7.50-7.40 (m, 2H, C$_{arom}$-H), 7.20 (d, *J* = 7.9 Hz, 2H, C$_{arom}$-H), 7.10 (d, *J* = 7.9 Hz, 2H, C$_{arom}$-H), 3.82-3.63 (m, 2H, OCH$_2$), 3.54 (app t, *J* = 8.6 Hz, 1H, C**H**CH$_2$), 2.42 (dd, *J* = 8.1,

4.7 Hz, 1H, CHC**H$_a$**H$_b$), 2.31 (s, 3H, C$_{arom}$-CH$_3$), 1.67 (dd, *J* = 9.1, 4.7 Hz, 1H, CHCH$_a$**H$_b$**), 0.69 (t, *J* = 7.1 Hz, 3H, CH$_2$C**H$_3$**). **^{13}C NMR** (75 MHz, CDCl$_3$) δ 195.2 (C$_{arom}$**C**=O), 168.6 (COO), 137.6 (C$_{arom}$-C), 136.9 (C$_{arom}$-C), 132.9 (C$_{arom}$-H), 131.8 (C$_{arom}$-C), 129.1 (2×C$_{arom}$-H), 128.9 (2×C$_{arom}$-H), 128.6 (2×C$_{arom}$-H), 128.3 (2×C$_{arom}$-H), 61.2 (OCH$_2$), 42.5 (**C**C=O), 30.6 (**C**HCH$_2$), 21.2 (C$_{arom}$-**C**H$_3$), 20.2 (CH**C**H$_2$), 13.6 (CH$_2$**C**H$_3$). **IR** (ATR): 1730 (C=O st), 1678 (C=O st), 1145 (C-O st as) cm^{-1}. **MS** (EI) m/z (%): 203.11 (M$^+$ - C$_7$H$_5$O, 1), 105.0 (100), 77.0 (50). **HRMS (ESI) m/z:** [M+Na]$^+$ Calcd for C$_{20}$H$_{20}$O$_3$Na 331.1210; Found 331.1322. **M.p.**: 56-59 ºC (petroleum ether/EtOAc).

2.2 Synthesis of indoles 2a-q

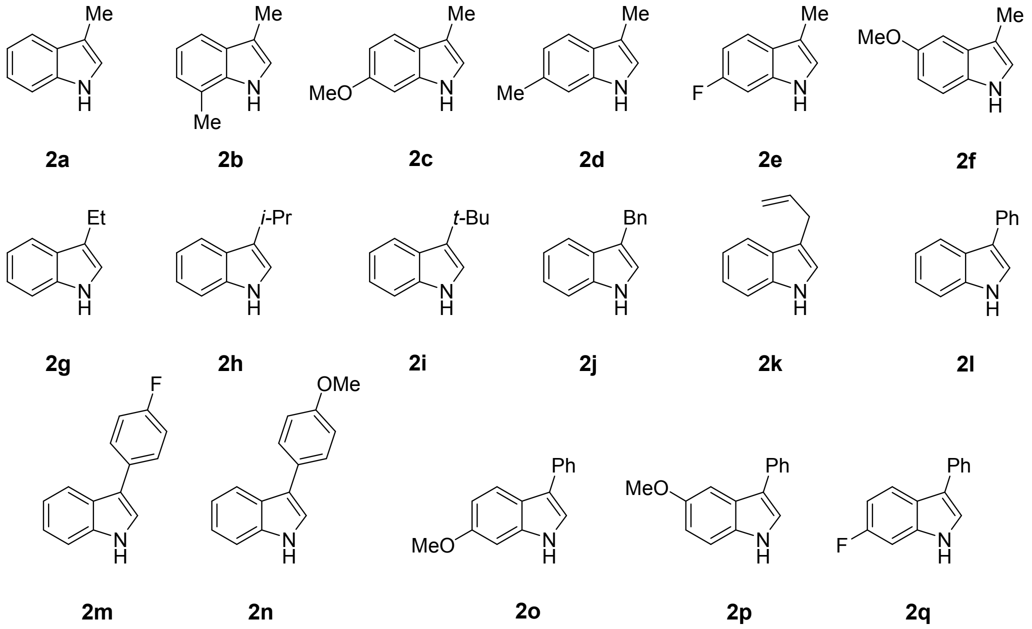

Figure S1. ***Employed indoles***

Indole **2a** was obtained from commercial sources. Compounds **2b-d**[15], **2f-g**[14], **2o**[14], **2e**[16], **2k**[15], **2h**[17], **2i**[18], **2j**[19], **2l-n**[20], **2p**[19], **2o**[21], and **2q**[20] were reported compounds and they were prepared following procedures previously described in the literature.

[15] Zhou, Z.; Li, Y.; Gong, L.; Meggers, E. *Org. Lett.* **2017**, *19*, 222.
[16] Tomakinian, T.; Guillot, R.; Kouklovsky, C.; Vincent, G. *Angew. Chem. Int. Ed.* **2014**, *53*, 11881.
[17] Zhang, Y.; Stephens, D.; Hernandez, G.; Mendoza, R.; Larionov, O. V. *Chem. Eur. J.* **2012**, *18*, 16612.
[18] Zhu, X.; Ganesan, A. *J. Org. Chem.* **2002**, *67*, 2705.
[19] Cano, R.; Yus, M.; Ramón, D. J. *Tetrahedron Lett.* **2013**, *54*, 3394.
[20] O'Brien, C. J.; Droege, D. G.; Jiu, A. Y.; Gandhi, S. S.; Paras, N. A.; Olson, S. H.; Conrad, J. *J. Org. Chem.* **2018**, *83*, 8926.
[21] Gattu, R.; Bhattacharjee, S.; Mahato, K.; Khan, A. T. *Org. Biomol. Chem.* **2018**, *16*, 3760.

2.3 Cyclocondensation of 3-substituted indoles with donor-acceptor cyclopropanes

2.3.1 Synthesis of dihydropyridoindoles 3 and 4

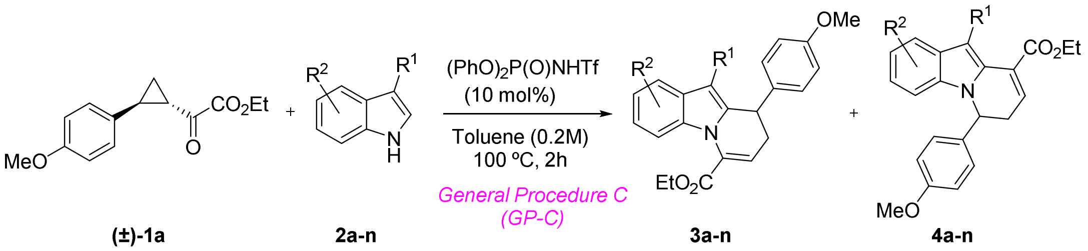

Scheme S3. ***General Procedure C for the synthesis of dihydropiridoindoles (±)-3 and (±)-4***

General Procedure C (GP-C) for the cyclocondensation: An oven-dried 5 mL screw-capped test tube containing a stirring bar was charged with the cyclopropane **1a** (1eq.) and the corresponding indole **2a-n** (1eq.) and dissolved in toluene (0.2 M) under Ar. After adding the diphenyl [(trifluoromethyl)sulfonyl]phosphoramidate catalyst (10 mol%) the mixture was stirred at 100 ºC in an oil bath. When the reaction was judged complete (monitored by TLC), it was warmed to room temperature, quenched by addition of aq. $NaHCO_3$ (sat) (3 mL) and diluted with EtOAc (3 mL). The phases were separated and the aqueous phase was extracted with EtOAc (3×3 mL). The combined organic layers were dried over Na_2SO_4, filtered and evaporated. The residue was purified by column chromatography on silica gel to afford the corresponding pure compounds **3a-n** and **4a-n**.

Ethyl (±)-9-(4-methoxyphenyl)-10-methyl-8,9-dihydropyrido[1,2-a]indole-6-carboxylate (3a) and ethyl (±)-6-(4-methoxyphenyl)-10-methyl-6,7-dihydropyrido[1,2-a]indole-9-carboxylate (4a). Following *GP-C*, **(±)-3a** (9 mg, 0.025 mmol, 50%) and **(±)-4a** (2 mg, 0.004 mmol, 9%) were isolated by FC (petroleum ether/EtOAc, from 25:1 to 19:1) on silica gel as white solids, starting from cyclopropane **1a** (12 mg, 0.05 mmol) and indole **2a** (7 mg, 0.05 mmol) and employing diphenyl [(trifluoromethyl)sulfonyl]phosphoramidate (2 mg, 0.005 mmol) as catalyst in toluene (0.25 mL) at 100 ºC for 2 h in an oil bath.

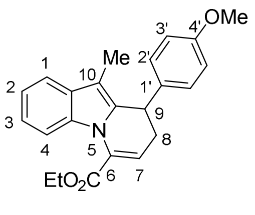

(±)-3a

^{1}H NMR (300 MHz, $CDCl_3$) δ 7.63-7.46 (m, 1H, H-1), 7.26-7.12 (m, 3H, H-2 + H-3 + H-4), 7.05 (d, *J* = 8.7 Hz, 2H, C_{arom}-H), 6.81 (d, *J* = 8.7 Hz, 2H, C_{arom}-H), 6.27 (dd, *J* = 6.6, 3.8 Hz, 1H, H-7), 4.46-4.05 (m, 3H, H-9 + OCH_2), 3.77 (s, 3H, OCH_3), 2.99-2.49 (m, 2H, H-8), 2.15 (s, 3H, C-10-CH_3), 1.37 (t, *J* = 7.1 Hz, 3H, OCH_2**CH_3**). **^{13}C NMR** (75 MHz, $CDCl_3$) δ 163.5 (COO), 158.4 (C_{arom}-O), 134.0 (C-4a + C-9a + C_{arom}-C), 131.3 (C-6), 129.9 (C-10a), 128.6 (2× C_{arom}-H), 122.0 (C-3), 120.3 (C-2), 119.9 (C-7), 118.5 (C-1), 114.0 (2× C_{arom}-H), 112.0 (C-4), 109.4 (C-10), 61.7 (OCH_2), 55.3 (OCH_3), 35.9 (C-9), 30.5 (C-8), 14.3 (OCH_2CH_3), 8.7 (C-10-**CH_3**). **IR** (ATR): 2987 (C-H st), 1727 (C=O st), 1260 (C-O st as) cm^{-1}. **MS** (EI) m/z (%): 362.2 (26), 361.2 (M^+, 100), 289.1 (16), 288.1 (72), 287.1

(23), 286.1 (20), 273.1 (19). **HRMS (ESI) m/z:** $[M+H]^+$ Calcd for $C_{23}H_{24}NO_3$ 362.1756; Found 362.1758. **M.p.**: 126-129 ºC (petroleum ether/EtOAc).

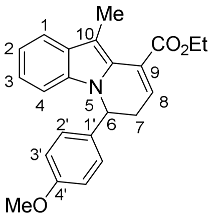

(±)-4a **^{1}H NMR** (300 MHz, $CDCl_3$) δ 7.73-7.52 (m, 1H, H-1), 7.18-6.98 (m, 3H, C_{arom}-H), 6.84 (d, *J* = 8.8 Hz, 2H, C_{arom}-H), 6.76 (d, *J* = 8.8 Hz, 2H, C_{arom}-H), 6.57 (dd, *J* = 7.1, 3.0 Hz, 1H, H-8), 5.63 (app d, *J* = 6.9 Hz, 1H, H-6), 4.45-4.25 (m, 2H, OC**H**$_2$CH$_3$), 3.73 (s, 3H, OCH_3), 3.13 (ddd, *J* = 17.1, 7.2, 3.0 Hz, 1H, H-7a), 2.82 (ddd, *J* = 17.1, 7.1, 1.8 Hz, 1H, H-7b), 2.40 (s, 3H, C-10-CH_3), 1.38 (t, *J* = 7.1 Hz, 3H, OCH_2C**H**$_3$). **^{13}C NMR** (75 MHz, $CDCl_3$) δ 166.6 (COO), 158.9 (C_{arom}-O), 135.6 (C-4a), 133.2 (C_{arom}-C), 129.4 (C-8), 129.3 (C_{arom}-C), 128.6 (C_{arom}-C), 127.4 (C_{arom}-C), 126.9 (2×C_{arom}-H), 123.1 (C_{arom}-H), 119.5 (2×C_{arom}-H), 114.1 (2×C_{arom}-H), 109.9 (C-10), 109.1 (C_{arom}-H), 61.3 (OCH_2), 55.3 (OCH_3), 52.5 (C-6), 33.1 (C-7), 14.4 (OCH_2**C**H_3), 10.5 (C-10-**C**H_3). **IR** (ATR): 2937 (C-H st), 1721 (C=O st), 1250 (C-O st as) cm^{-1}. **MS** (EI) m/z (%): 362.2 (26), 361.2 (M^+, 100), 288.1 (39), 287.1 (15), 286.1 (15), 273.1 (21), 121.0 (15). **HRMS (ESI) m/z:** $[M+H]^+$ Calcd for $C_{23}H_{24}NO_3$ 362.1756; Found 362.1751. **M.p.**: 64-67 ºC (petroleum ether/EtOAc).

Ethyl (±)-9-(4-methoxyphenyl)-4,10-dimethyl-8,9-dihydropyrido[1,2-a]indole-6-carboxylate (3b) and ethyl (±)-6-(4-methoxyphenyl)-4,10-dimethyl-6,7-dihydropyrido[1,2-a]indole-9-carboxylate (4b). Following *GP-C*, **(±)-3b** (10 mg, 0.026 mmol, 54%) and **(±)-4b** (1 mg, 0.002 mmol, 4%) were isolated by FC (petroleum ether/EtOAc, from 93:7 to 95:5) on silica gel as white solids, starting from cyclopropane **1a** (12 mg, 0.05 mmol) and indole **2b** (7 mg, 0.05 mmol) and employing diphenyl [(trifluoromethyl)sulfonyl]phosphoramidate (2 mg, 0.005 mmol) as catalyst in toluene (0.25 mL) at 100 ºC for 2 h.

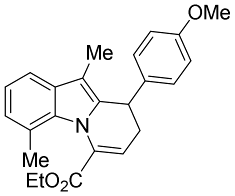

(±)-3b **^{1}H NMR** (300 MHz, $CDCl_3$) δ 7.35 (d, *J* = 7.7 Hz, 1H, H-1), 7.07 (app t, *J* = 7.5 Hz, 1H, H-2), 7.03-6.93 (m, 3H, C_{arom}-H + H-3), 6.79 (d, *J* = 8.7 Hz, 2H, C_{arom}-H), 6.32 (dd, *J* = 6.9, 4.8 Hz, 1H, H-7), 4.46-4.07 (m, 3H, H-9 + OCH_2), 3.77 (s, 3H, OCH_3), 2.84-2.61 (m, 2H, H-8), 2.43 (s, 3H, C-4-**C**H_3), 2.04 (s, 3H, C-10-CH_3), 1.27 (t, *J* = 7.1 Hz, 3H, OCH_2C**H**$_3$). **^{13}C NMR** (75 MHz, $CDCl_3$) δ 164.6 (COO), 158.4 (C_{arom}-O), 135.9 (C-4a), 134.5 (C_{arom}-C), 133.6 (C-9a), 133.0 (C-6), 130.5 (C-10a), 128.8 (2× C_{arom}-H), 125.3 (C-2), 121.9 (C-4), 121.7 (C-3), 120.6 (C-7), 116.1 (C-1), 113.9 (2× C_{arom}-H), 109.2 (C-10), 61.5 (OCH_2), 55.3 (OCH_3), 37.9 (C-9), 31.1 (C-8), 19.4 (C-4-**C**H_3), 14.2 (OCH_2**C**H_3), 9.0 (C-10-**C**H_3). **IR** (ATR): 2958 (C-H st), 1725 (C=O st), 1259 (C-O st as) cm^{-1}. **MS** (EI) m/z (%): 376.2 (27), 375.2 (M^+, 100), 302.1 (38), 287.1 (16). **HRMS (ESI) m/z:** $[M+H]^+$ Calcd for $C_{24}H_{26}NO_3$ 376.1913; Found 376.1905. **M.p.**: 138-141 ºC (petroleum ether/EtOAc).

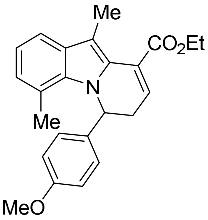

(±)-4b **^{1}H NMR** (300 MHz, $CDCl_3$) δ 7.46 (d, *J* = 7.8 Hz, 1H, H-1), 6.97 (app t, *J* = 7.4 Hz, 1H, H-2), 6.86 (d, *J* = 7.4 Hz, 1H, H-3), 6.82-6.69 (m, 4H, C_{arom}-H), 6.48 (dd, *J* = 7.3, 2.9 Hz, 1H, H-8), 6.16 (d, *J* = 6.8 Hz, 1H, H-6), 4.41-4.23 (m, 2H, OC**H_2**CH_3), 3.73 (s, 3H, OCH_3), 3.14 (ddd, *J* = 16.8, 6.8, 2.9 Hz, 1H, H-7a), 2.81 (dd, *J* = 16.8, 7.3 Hz, 1H, H-7b), 2.49 (s, 3H, C_{arom}-CH_3), 2.34 (s, 3H, C-10-CH_3), 1.36 (t, *J* = 7.1 Hz, 3H, OCH_2C**H_3**). **^{13}C NMR** (75 MHz, $CDCl_3$) δ 166.8 (COO), 158.8 (C_{arom}-O), 134.6 (C_{arom}-C), 134.4 (C_{arom}-C), 129.7 (C_{arom}-C), 129.0 (C_{arom}-C), 128.8 (C-8), 127.9 (C_{arom}-C), 126.7 (2× C_{arom}-H), 126.1 (C_{arom}-H), 120.4 (C_{arom}-C), 119.4 (C_{arom}-H), 117.4 (C_{arom}-H), 114.1 (2× C_{arom}-H), 109.9 (C-10), 61.3 (OCH_2), 55.3 (OCH_3), 54.2 (C-6), 33.4 (C-7), 20.0 (C-4-**C**H_3), 14.4 (OCH_2**C**H_3), 10.5 (C-10-**C**H_3). **IR** (ATR): 2918 (C-H st), 1732 (C=O st), 1248 (C-O st as) cm^{-1}. **MS** (EI) m/z (%): 376.2 (27), 375.2 (M^+, 100), 302.1 (48), 301.1 (17), 300.1 (15), 287.1 (28), 286.1 (15), 121.0 (18). **HRMS (ESI) m/z:** $[M+H]^+$ Calcd for $C_{24}H_{26}NO_3$ 376.1913; Found 376.1909.

Ethyl (±)-3-methoxy-9-(4-methoxyphenyl)-10-methyl-8,9-dihydropyrido[1,2-a]indole-6-carboxylate (3c). Following *GP-C*, **(±)-3c** (12 mg, 0.030 mmol, 61%) was isolated by FC (petroleum ether/EtOAc, 25:1) on silica gel as yellow solid, starting from cyclopropane **1a** (12 mg, 0.05 mmol) and indole **2c** (8 mg, 0.05 mmol) and employing diphenyl [(trifluoromethyl)sulfonyl]phosphoramidate (2 mg, 0.005 mmol) as catalyst in toluene (0.25 mL) at 100 ºC for 2 h.

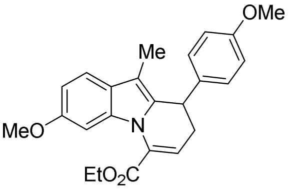

(±)-3c **^{1}H NMR** (300 MHz, $CDCl_3$) δ 7.40 (d, *J* = 8.5 Hz, 1H, H-1), 7.05 (d, *J* = 8.5 Hz, 2H, C_{arom}-H), 6.92-6.76 (m, 3H, C_{arom}-H+ H-2), 6.73 (d, *J* = 2.2 Hz, 1H, H-4), 6.25 (dd, *J* = 6.7, 3.9 Hz, 1H, H-7), 4.63-4.28 (m, 3H, H-9 + OCH_2), 3.86 (s, 3H, OCH_3), 3.77 (s, 3H, OCH_3), 2.87-2.60 (m, 2H, H-8), 2.11 (s, 3H, C-10-CH_3), 1.38 (t, *J* = 7.1 Hz, 3H, OCH_2C**H_3**). **^{13}C NMR** (75 MHz, $CDCl_3$) δ 163.5 (COO), 158.3 (C_{arom}-O), 156.3 (C-O), 134.8 (C-4a), 134.2 (C_{arom}-C), 132.9 (C-9a), 131.3 (C-6), 128.5 (2× C_{arom}-H), 124.3 (C-10a), 120.0 (C-7), 118.8 (C-1), 113.9 (2× C_{arom}-H), 109.2 (C-2 + C-10), 97.0 (C-4), 61.7 (OCH_2), 55.9 (OCH_3), 55.3 (OCH_3), 35.9 (C-9), 30.6 (C-8), 14.4 (OCH_2**C**H_3), 8.7 (C-10-**C**H_3). **IR** (ATR): 2936 (C-H st), 1724 (C=O st), 1247 (C-O st as) cm^{-1}. **MS** (EI) m/z (%): 392.2 (26), 391.2 (M^+, 100), 318.1 (35), 302.1 (13). **HRMS (ESI) m/z:** $[M+H]^+$ Calcd for $C_{24}H_{26}NO_4$ 392.1862; Found 392.1855. **M.p.**: 100-104 ºC (petroleum ether/EtOAc).

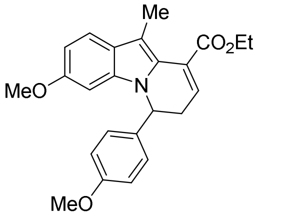

(±)-4c ^{1}**H NMR** (300 MHz, $CDCl_3$) δ 7.47 (d, *J* = 8.6, 1H, H-1), 6.86 (d, *J* = 8.6, 2H, C_{arom}-H), 6.80-6.69 (m, 3H, C_{arom}-H + H-2), 6.52-6.46 (m, 2H, H-4 + H-8), 5.54 (app d, *J* = 6.6 Hz, 1H, H-6), 4.40-4.24 (m, 2H, OC**H**$_2$CH$_3$), 3.76 (s, 3H, OCH_3), 3.73 (s, 3H, OCH_3), 3.11 (ddd, *J* = 17.0, 7.0, 3.1 Hz, 1H, H-7a), 2.80 (ddd, *J* = 17.0, 7.1, 1.8 Hz, 1H, H-7b), 2.36 (s, 3H, C-10-CH_3), 1.37 (t, *J* = 7.1 Hz, 3H, OCH_2C**H**$_3$). ^{13}**C NMR** (75 MHz, $CDCl_3$) δ 166.7 (COO), 158.9 (C-3), 157.4 (C_{arom}-O), 136.5 (C-4a), 133.2 (C_{arom}-C), 128.6 (C_{arom}-C), 128.0 (C-8), 127.0 (2× C_{arom}-H), 126.6 (C_{arom}-C), 123.9 (C_{arom}-C), 120.2 (C_{arom}-H), 114.1 (2× C_{arom}-H), 110.0 (C-10), 109.2 (C_{arom}-H), 92.8 (C_{arom}-H), 61.2 (OCH_2), 55.7 (OCH_3), 55.7 (OCH_3), 52.5 (C-6), 33.1 (C-7), 14.3 (OCH_2**C**H$_3$), 10.5 (C-10-**C**H$_3$). **IR** (ATR): 2934 (C-H st), 1721 (C=O st), 1249 (C-O st as) cm^{-1}. **MS** (EI) m/z (%): 392.2 (27), 391.2 (M^+, 100), 318.1 (23), 303.1 (13). **HRMS (ESI) m/z:** $[M+H]^+$ Calcd for $C_{24}H_{26}NO_4$ 392.1862; Found 392.1866. **M.p.**: 65-68 ºC (petroleum ether/EtOAc).

Ethyl (±)-9-(4-methoxyphenyl)-3,10-dimethyl-8,9-dihydropyrido[1,2-a]indole-6-carboxylate (3d) and ethyl (±)-6-(4-methoxyphenyl)-3,10-dimethyl-6,7-dihydropyrido[1,2-a]indole-9-carboxylate (4d). Following *GP-C*, **(±)-3d** (10 mg, 0.026 mmol, 52%) and **(±)-4d** (2 mg, 0.004 mmol, 8%) were isolated by FC (petroleum ether/EtOAc, from 25:1 to 19:1) on silica gel as yellow solids, starting from cyclopropane **1a** (12 mg, 0.05 mmol) and indole **2d** (7 mg, 0.05 mmol) and employing diphenyl [(trifluoromethyl)sulfonyl]phosphoramidate (2 mg, 0.005 mmol) as catalyst in toluene (0.25 mL) at 100 ºC for 2 h in an oil bath.

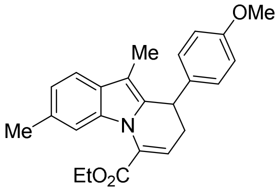

(±)-3d ^{1}**H NMR** (300 MHz, $CDCl_3$) δ 7.40 (d, *J* = 7.9 Hz, 1H, H-1), 7.10-6.89 (m, 4H, C_{arom}-H + H-4 + H-2), 6.79 (d, *J* = 8.7 Hz, 2H, C_{arom}-H), 6.22 (dd, *J* = 6.6, 3.9 Hz, 1H, H-7), 4.49-4.39 (m, 3H, H-9 + OCH_2), 3.76 (s, 3H, OCH_3), 2.85-2.59 (m, 2H, H-8), 2.47 (s, 3H, CH_3), 2.11 (s, 3H, CH_3), 1.37 (t, *J* = 7.1 Hz, 3H, OCH_2C**H**$_3$). ^{13}**C NMR** (75 MHz, $CDCl_3$) δ 163.7 (COO), 158.4 (C_{arom}-O), 134.4 (C-4a), 134.2 (C_{arom}-C), 133.3 (C-9a), 131.7 (C-3), 131.4 (C-6), 128.6 (2× C_{arom}-H), 127.7 (C-10a), 121.9 (C-2), 119.6 (C-9), 118.2 (C-1), 113.9 (2× C_{arom}-H), 112.1 (C-4), 109.2 (C-10), 61.6 (OCH_2), 55.3 (OCH_3), 35.9 (C-9), 30.5 (C-8), 22.1 (C-3-**C**H$_3$), 14.3 (OCH_2**C**H$_3$), 8.7 (C-10-**C**H$_3$). **MS** (EI) m/z (%): 376.2 (26), 375.2 (M^+, 100), 302.2 (56), 301.1 (16), 287.1 (19). **IR** (ATR): 2925 (C-H st), 1725 (C=O st), 1245 (C-O st as) cm^{-1}. **HRMS (ESI) m/z:** $[M+H]^+$ Calcd for $C_{24}H_{26}NO_3$ 376.1913; Found 376.1911. **M.p.**: 76-79 ºC (petroleum ether/EtOAc).

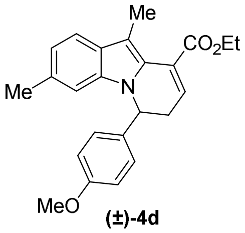

(±)-4d

^{1}H NMR (300 MHz, $CDCl_3$) δ 7.48 (d, *J* = 7.9 Hz, 1H, H-1), 7.02-6.81 (m, 4H, C_{arom}-H + H-4 + H-2), 6.76 (d, *J* = 8.7 Hz, 2H, C_{arom}-H), 6.52 (dd, *J* = 7.2, 3.0 Hz, 1H, H-8), 5.70-5.52 (m, 1H, H-6), 4.50-4.12 (m, 2H, $OC\mathbf{H}_2CH_3$), 3.73 (s, 3H, OCH_3), 3.11 (ddd, *J* = 17.0, 7.2, 3.0 Hz, 1H, H-7a), 2.80 (ddd, *J* = 17.0, 7.2, 1.6 Hz, 1H, H-7b), 2.39 (s, 3H, C_{arom}-CH_3), 2.37 (s, 3H, C_{arom}-CH_3), 1.37 (t, *J* = 7.1 Hz, 3H, $OCH_2C\mathbf{H}_3$). **^{13}C NMR** (75 MHz, $CDCl_3$) δ 166.6 (COO), 158.9 (C_{arom}-O), 136.1 (C-4a), 133.4 (C_{arom}-C), 133.1 (C_{arom}-C), 128.7 (C-8 + C_{arom}-C), 127.5 (C_{arom}-C), 127.3 (C_{arom}-C), 126.9 (2× C_{arom}-H), 121.3 (C_{arom}-H), 119.2 (C_{arom}-H), 114.1 (2x C_{arom}-H), 109.9 (C-10), 108.9 (C_{arom}-H), 61.3 (OCH_2), 55.3 (OCH_3), 52.3 (C-6), 33.1 (C-7), 22.1 (C-3-$\mathbf{C}H_3$), 14.4 ($OCH_2\mathbf{C}H_3$), 10.5 (C-10-$\mathbf{C}H_3$). **IR** (ATR): 2925 (C-H st), 1721 (C=O st), 1250 (C-O st as) cm^{-1}. **MS** (EI) m/z (%): 375.2 (M^+, 36), 281.0 (17), 208.0 (23), 207.0 (100), 96.0 (15). **HRMS (ESI) m/z:** $[M+H]^+$ Calcd for $C_{24}H_{26}NO_3$ 376.1913; Found 376.1908. **M.p.**: 85-88 ºC (petroleum ether/EtOAc).

Ethyl (±)-3-fluoro-9-(4-methoxyphenyl)-10-methyl-8,9-dihydropyrido[1,2-a]indole-6-carboxylate (3e) and ethyl (±)-3-fluoro-6-(4-methoxyphenyl)-10-methyl-6,7-dihydropyrido[1,2-a]indole-9-carboxylate (4e). Following *GP-C*, **(±)-3e** (11 mg, 0.030 mmol, 60%) and **(±)-4e** (1 mg, 0.003 mmol, 5%) were isolated by FC (petroleum ether/EtOAc, from 25:1 to 19:1) on silica gel as yellow solids, starting from cyclopropane **1a** (12 mg, 0.05 mmol), indole **2e** (7 mg, 0.05 mmol) and employing diphenyl [(trifluoromethyl)sulfonyl]phosphoramidate (2 mg, 0.005 mmol) as catalyst in toluene (0.25 mL) at 100 ºC for 2 h.

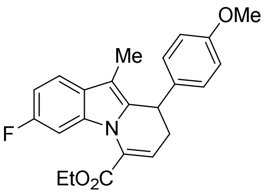

(±)-3e

^{1}H NMR (300 MHz, $CDCl_3$) δ 7.49-7.32 (m, 1H, H-1), 7.03 (d, *J* = 7.9 Hz, 2H, C_{arom}-H), 6.97-6.86 (m, 2H, H-2 + H-4), 6.80 (d, *J* = 8.0 Hz, 2H, C_{arom}-H), 6.41-6.21 (m, 1H, H-7), 4.63-4.07 (m, 3H, H-9 + OCH_2), 3.77 (s, 3H, OCH_3), 2.91-2.54 (m, 2H, H-8), 2.11 (s, 3H, C-10-CH_3), 1.38 (t, *J* = 7.0 Hz, 3H, $OCH_2C\mathbf{H}_3$). **^{13}C NMR** (75 MHz, $CDCl_3$) δ 163.1 (COO), 159.8 (d, $^{1}J_{C\text{-}F}$ = 237.1 Hz, C-3), 158.3 (C_{arom}-O), 134.3 (d, $^{4}J_{C\text{-}F}$ = 3.6 Hz, C-4a), 134.1 (d, $^{3}J_{C\text{-}F}$ = 12.3 Hz, C-4a), 133.8 (C-9a + C_{arom}-C), 131.0 (C-6), 128.5 (2× C_{arom}-H), 126.4 (C-10a), 120.9 (C-7), 118.9 (d, $^{3}J_{C\text{-}F}$ = 10.0 Hz, C-1), 114.0 (2× C_{arom}-H), 109.3 (C-10), 108.6 (d, $^{2}J_{C\text{-}F}$ = 24.3 Hz, C-4), 99.2 (d, $^{2}J_{C\text{-}F}$ = 27.7 Hz, C-2), 61.8 (OCH_2), 55.3 (OCH_3), 35.9 (C-9), 30.5 (C-8), 14.3 ($OCH_2\mathbf{C}H_3$), 8.7 (C-10-$\mathbf{C}H_3$). **IR** (ATR): 2933 (C-H st), 1722 (C=O st), 1247 (C-O st as), 1147 (C-F st) cm^{-1}. **MS** (EI) m/z (%): 380.2 (26), 379.2 (M^+, 100), 307.2 (17), 306.2 (79), 305.2 (29), 304.2 (22), 291.1 (22), 198.1 (22). **HRMS (ESI) m/z:** $[M+H]^+$ Calcd for $C_{23}H_{23}NO_3F$ 380.1662; Found 380.1668. **M.p.**: 123-126 ºC (petroleum ether/EtOAc).

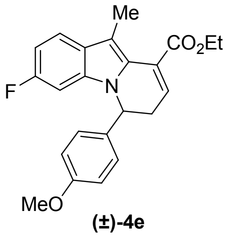

(±)-4e **^{1}H NMR** (300 MHz, $CDCl_3$) δ 7.50 (dd, *J* = 8.6, 5.3 Hz, 1H, H-1), 6.90-6.80 (m, 3H, C_{arom}-H + H-2), 6.80-6.67 (m, 3H, C_{arom}-H + H-4), 6.56 (dd, *J* = 7.1, 3.1 Hz, 1H, H-8), 5.51 (app d, *J* = 7.1 Hz, 1H, H-6), 4.42-4.22 (m, 2H, $OC\mathbf{H}_2CH_3$), 3.74 (s, 3H, OCH_3), 3.13 (ddd, *J* = 17.2, 7.1, 3.1 Hz, 1H, H-7a), 2.81 (ddd, *J* = 17.2, 7.1, 1.9 Hz, 1H, H-7b), 2.36 (s, 3H, C-10-CH_3), 1.37 (t, *J* = 7.1 Hz, 3H, $OCH_2C\mathbf{H}_3$). **^{13}C NMR** (75 MHz, $CDCl_3$) δ 166.4 (COO), 160.7 (d, $^1J_{C-F}$ = 239.2 Hz, C-3), 159.1 (C_{arom}-O), 135.8 (d, $^3J_{C-F}$ = 12.4 Hz, C-4a), 132.8 (C_{arom}-C), 129.2 (C-8), 128.5 (C_{arom}-C), 127.9 (d, $^4J_{C-F}$ = 4.2 Hz, C-10a), 126.9 (2× C_{arom}-H), 126.0 (C_{arom}-C), 120.4 (d, $^3J_{C-F}$ = 10.3 Hz, C-1), 114.2 (2x C_{arom}-H), 110.0 (C-10), 108.2 (d, $^2J_{C-F}$ = 24.7 Hz, C_{arom}-H), 95.6 (d, $^2J_{C-F}$ = 26.5 Hz, C_{arom}-H), 61.4 (OCH_2), 55.4 (OCH_3), 52.8 (C-6), 33.1 (C-7), 14.4 ($OCH_2\mathbf{C}H_3$), 10.5 (C-10-$\mathbf{C}H_3$). **IR** (ATR): 2930 (C-H st), 1728 (C=O st), 1242 (C-O st as), 1179 (C-F st) cm^{-1}. **MS** (EI) m/z (%): 380.2 (26), 379.2 (M^+, 100), 306.2 (29). **HRMS (ESI) m/z:** $[M+H]^+$ Calcd for $C_{23}H_{23}NO_3F$ 380.1662; Found 380.1653.

Ethyl (±)-2-methoxy-9-(4-methoxyphenyl)-10-methyl-8,9-dihydropyrido[1,2-a]indole-6-carboxylate (3f) and ethyl (±)-2-methoxy-6-(4-methoxyphenyl)-10-methyl-6,7-dihydropyrido[1,2-a]indole-9-carboxylate (4f). Following *GP-C*, **(±)-3f** (8 mg, 0.021 mmol, 42%) and **(±)-4f** (2 mg, 0.004 mmol, 8%) were isolated by FC (petroleum ether/EtOAc, from 25:1 to 19:1) on silica gel as yellow solids, starting from cyclopropane **1a** (12 mg, 0.05 mmol), indole **2f** (7 mg, 0.05 mmol) and employing diphenyl [(trifluoromethyl)sulfonyl]phosphoramidate (2 mg, 0.005 mmol) as catalyst in toluene (0.25 mL) at 100 ºC for 2 h.

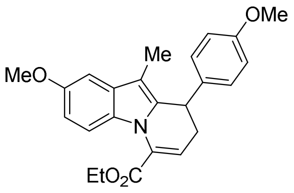

(±)-3f **^{1}H NMR** (300 MHz, $CDCl_3$) δ 7.17-6.92 (m, 4H, C_{arom}-H), 6.91-6.69 (m, 3H, C_{arom}-H), 6.22 (dd, *J* = 6.8, 3.8 Hz, 1H, H-7), 4.55-4.18 (m, 3H, H-9 + OCH_2), 3.88 (s, 3H, OCH_3), 3.76 (s, 3H, OCH_3), 2.84-2.60 (m, 2H, H-8), 2.10 (s, 3H, C-10-CH_3), 1.36 (t, *J* = 7.2 Hz, 3H, $OCH_2C\mathbf{H}_3$). **^{13}C NMR** (75 MHz, $CDCl_3$) δ 163.5 (COO), 158.4 (C_{arom}-O), 154.7 (C-2), 134.7 (C_{arom}-C), 134.0 (C-9a), 131.3 (C-6), 130.5 (C-10a), 129.2 (C-4a), 128.6 (2× C_{arom}-H), 119.3 (C-7), 114.0 (2× C_{arom}-H), 112.7 (C-4), 111.3 (C-3), 109.1 (C-10), 101.0 (C-1), 61.7 (OCH_2), 55.9 (OCH_3), 55.3 (OCH_3), 35.9 (C-9), 30.6 (C-8), 14.4 ($OCH_2\mathbf{C}H_3$), 8.7 (C-10-$\mathbf{C}H_3$). **IR** (ATR): 2926 (C-H st), 1728 (C=O st), 1258 (C-O st as) cm^{-1}. **MS** (EI) m/z (%): 392.2 (27), 391.2 (M^+, 100), 318.1 (22). **HRMS (ESI) m/z:** $[M+H]^+$ Calcd for $C_{24}H_{26}NO_4$ 392.1862; Found 392.1857. **M.p.**: 98-101 ºC (petroleum ether/EtOAc).

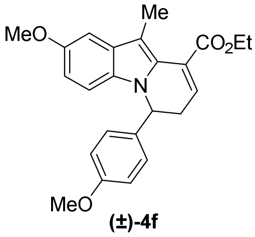

(±)-4f **^{1}H NMR** (300 MHz, $CDCl_3$) δ 7.03 (d, *J* = 2.3 Hz, 1H, H-1), 6.95 (d, *J* = 8.9, 1H, H-3), 6.86-6.70 (m, 5H, C_{arom}-H + H-4), 6.54 (dd, *J* = 7.0, 3.1 Hz, 1H, H-8), 5.56 (d, *J* = 7.1 Hz, 1H, H-6), 4.42-4.18 (m, 2H, OC**H**$_2$CH_3), 3.36 (s, 3H, OCH_3), 3.73 (s, 3H, OCH_3), 3.21-3.01 (m, 1H, H-7a), 2.88-2.71 (m, 1H, H-7b), 2.36 (s, 3H, C-10-CH_3), 1.37 (t, *J* = 7.1 Hz, 3H, OCH_2C**H**$_3$). **^{13}C NMR** (75 MHz, $CDCl_3$) δ 166.6 (COO), 158.9 (C_{arom}-O), 154.1 (**C**-2-OCH_3), 133.3 (C_{arom}-C), 131.0 (C_{arom}-C), 129.5 (C_{arom}-C), 129.0 (C-8), 128.7 (C_{arom}-C), 128.0 (C_{arom}-C), 126.9 (2× C_{arom}-H), 114.0 (2× C_{arom}-H), 113.4 (C_{arom}-H), 109.9 (C_{arom}-H), 109.4 (C-10), 101.1 (C_{arom}-H), 61.3 (OCH_2), 56.1 (OCH_3), 55.3 (OCH_3), 52.7 (C-6), 33.1 (C-7), 14.4 (OCH_2CH_3), 10.6 (C-10-**C**H_3). **IR** (ATR): 2918 (C-H st), 1724 (C=O st), 1249 (C-O st as) cm^{-1}. **HRMS (ESI) m/z:** $[M+H]^+$ Calcd for $C_{24}H_{26}NO_4$ 392.1862; Found 392.1866.

Ethyl (±)-10-ethyl-9-(4-methoxyphenyl)-8,9-dihydropyrido[1,2-a]indole-6-carboxylate (3g) and ethyl (±)-10-ethyl-6-(4-methoxyphenyl)-6,7-dihydropyrido[1,2-a]indole-9-carboxylate (4g). Following *GP-C*, **(±)-3g** (9 mg, 0.023 mmol, 46%) and **(±)-4g** (2 mg, 0.005 mmol, 10%) were isolated by FC (petroleum ether/EtOAc, from 25:1 to 19:1) on silica gel as white solids, starting from cyclopropane **1a** (12 mg, 0.05 mmol), indole **2g** (7 mg, 0.05 mmol) and employing diphenyl [(trifluoromethyl)sulfonyl]phosphoramidate (2 mg, 0.005 mmol) as catalyst in toluene (0.25 mL) at 100 °C for 2 h in an oil bath.

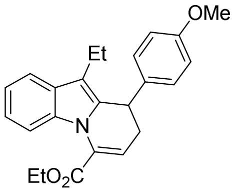

(±)-3g **^{1}H NMR** (300 MHz, $CDCl_3$) δ 7.63-7.53 (m, 1H, H-1), 7.22-7.10 (m, 3H, H-2 + H-3 + H-4), 7.02 (d, *J* = 8.6 Hz, 2H, C_{arom}-H), 6.78 (d, *J* = 8.7 Hz, 2H, C_{arom}-H), 6.23 (dd, *J* = 7.0, 3.6 Hz, 1H, H-7), 4.58-4.28 (m, 3H, H-9 + OCH_2), 3.76 (s, 3H, OCH_3), 2.91-2.50 (m, 4H, C-10-CH_2 + H-8), 1.36 (t, *J* = 7.1 Hz, 3H, OCH_2C**H**$_3$), 1.09 (t, *J* = 7.5 Hz, 3H, C-10-CH_2C**H**$_3$). **^{13}C NMR** (75 MHz, $CDCl_3$) δ 163.5 (COO), 158.3 (C_{arom}-O), 134.3 (C-4a + C_{arom}-C), 133.5 (C-9a), 131.4 (C-6), 128.9 (C-10a), 128.5 (2× C_{arom}-H), 121.9 (C-3), 120.3 (C-2), 119.9 (C-7), 118.7 (C-1), 115.9 (C-10), 113.9 (2× C_{arom}-H), 112.1 (C-4), 61.7 (OCH_2), 55.3 (OCH_3), 35.6 (C-9), 30.6 (C-8), 17.5 (C-10-**C**H_2), 15.3 (C-10-CH_2**C**H_3), 14.4 (OCH_2**C**H_3). **IR** (ATR): 2933 (C-H st), 1724 (C=O st), 1246 (C-O st as) cm^{-1}. **MS** (EI) m/z (%): 376.2 (27), 375.2 (M^+, 100), 360.2 (25), 302.1 (40), 286.1 (26), 273.1 (18), 239.1 (16). **HRMS (ESI) m/z:** $[M+H]^+$ Calcd for $C_{24}H_{26}NO_3$ 376.1913; Found 376.1902. **M.p.**: 50-53 °C (petroleum ether/EtOAc).

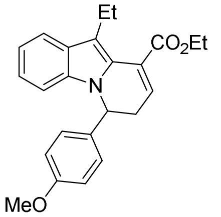

(±)-4g **^{1}H NMR** (300 MHz, $CDCl_3$) δ 7.65 (d, *J* = 7.6 Hz, 1H, H-1), 7.19-7.01 (m, 3H, H-2 + H-3 + H-4), 6.84 (d, *J* = 8.7 Hz, 2H, C_{arom}-H), 6.76 (d, *J* = 8.8 Hz, 2H, C_{arom}-H), 6.53 (dd, *J* = 7.1, 3.0 Hz, 1H, H-8), 5.63 (app d, *J* = 7.0 Hz, 1H, H-6), 4.53-4.19 (m, 2H, OC**H**$_2$CH$_3$), 3.73 (s, 3H, OCH_3), 3.13 (ddd, *J* = 17.1, 7.1, 3.0 Hz, 1H, H-7a), 2.85 (m, 3H, C-10-C**H**$_2$CH$_3$ + H-7b), 1.48-1.17 (m, 6H, C-10-CH$_2$**C**H$_3$ + OCH$_2$C**H**$_3$). **^{13}C NMR** (75 MHz, $CDCl_3$) δ 166.8 (COO), 158.9 (C_{arom}-O), 135.8 (C-4a), 133.3 (C_{arom}-C), 129.3 (C_{arom}-C), 129.2 (C-8), 128.9 (C_{arom}-C), 128.4 (C_{arom}-C), 126.9 (2× C_{arom}-H), 126.6 (C_{arom}-C), 123.0 (C_{arom}-H), 119.8 (C_{arom}-H), 119.4 (C_{arom}-H), 116.7 (C-10), 114.1 (2× C_{arom}-H), 109.2 (C_{arom}-H), 61.3 (OCH_2), 55.3 (OCH_3), 52.4 (C-6), 33.0 (C-7), 18.5 (C-10-**C**H$_2$CH$_3$), 15.0 (CH$_2$**C**H$_3$), 14.3 (CH$_2$**C**H$_3$). **IR** (ATR): 2923 (C-H st), 1722 (C=O st), 1249 (C-O st as) cm^{-1}. **MS** (EI) m/z (%): 376.2 (26), 375.2 (M^+, 100), 360.2 (29), 273.1 (22). **HRMS (ESI) m/z:** $[M+H]^+$ Calcd for $C_{24}H_{26}NO_3$ 376.1913; Found 376.1902. **M.p.**: 48-51 ºC (petroleum ether/EtOAc).

Ethyl (±)-10-isopropyl-9-(4-methoxyphenyl)-8,9-dihydropyrido[1,2-a]indole-6-carboxylate (3h). Following *GP-C*, **(±)-3h** (4.9 mg, 0.012 mmol, 25%) was isolated by FC (petroleum ether/EtOAc 19:1) on silica gel as white solid, starting from cyclopropane **1a** (12 mg, 0.05 mmol), indole 2**h** (8 mg, 0.05 mmol) and employing diphenyl [(trifluoromethyl)sulfonyl]phosphoramidate (2 mg, 0.005 mmol) as catalyst in toluene (0.25 mL) at 100 ºC for 2 h.

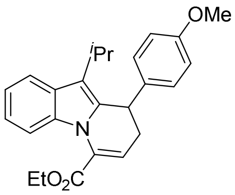

(±)-3h **^{1}H NMR** (300 MHz, $CDCl_3$) δ 7.79-7.60 (m, 1H, H-1), 7.21-7.03 (m, 3H, H-2 + H-3 + H-4), 6.98 (d, *J* = 8.5 Hz, 2H, C_{arom}-H), 6.76 (d, *J* = 8.5 Hz, 2H, C_{arom}-H), 6.20 (ddd, *J* = 7.2, 3.4, 0.8 Hz, 1H, H-7), 4.63-4.54 (m, 1H, H-9), 4.49-4.24 (m, 2H, OCH_2), 3.75 (s, 3H, OCH_3), 3.21-3.04 (m, 1H, C**H**(CH$_3$)$_2$), 2.87-2.57 (m, 2H, H-8), 1.43-1.14 (m, 9H, CH(C**H**$_3$)$_2$ + OCH$_2$C**H**$_3$). **^{13}C NMR** (75 MHz, $CDCl_3$) δ 163.5 (COO), 158.3 (C_{arom}-O), 134.5 (C-4a), 134.2 (C-9-**C**$_{arom}$), 132.6 (C-9a), 131.4 (C-6), 128.4 (2× C_{arom}-H), 127.8 (C-10a), 121.6 (C-3), 120.1 (C-2), 120.0 (C-1), 119.9 (C-7), 119.6 (C-10), 113.8 (2× C_{arom}-H), 112.1 (C-4), 61.7 (OCH_2), 55.3 (OCH_3), 35.8 (C-9), 30.6 (C-8), 26.0 (**C**H(CH$_3$)$_2$), 22.9 (CH(**C**H$_3$)$_2$), 22.7 (CH(**C**H$_3$)$_2$), 14.3 (OCH$_2$**C**H$_3$).**IR** (ATR): 2927 (C-H st), 1725 (C=O st), 1246 (C-O st as) cm^{-1}. **HRMS (ESI) m/z:** $[M+H]^+$ Calcd for $C_{25}H_{28}NO_3$ 390.2069; Found 390.2066. **M.p.**: 59-62 ºC (petroleum ether/EtOAc).

Ethyl (±)-10-(tert-butyl)-9-(4-methoxyphenyl)-8,9-dihydropyrido[1,2-a]indole-6-carboxylate (3i). Following *GP-C*, **(±)-3i** (3 mg, 0.007 mmol, 14%) was isolated by FC (petroleum ether/EtOAc 19:1) on silica gel as white solid, starting from cyclopropane **1a** (12 mg, 0.05 mmol), indole 2**i** (9 mg, 0.05 mmol) and employing diphenyl [(trifluoromethyl)sulfonyl]phosphoramidate (2 mg, 0.005 mmol) as catalyst in toluene (0.25 mL) at 100 ºC for 2 h in an oil bath.

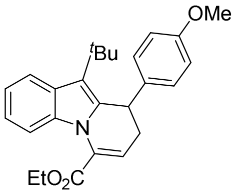

(±)-3i

^{1}H NMR (300 MHz, $CDCl_3$) δ 7.95-7.86 (m, 1H, H-1), 7.23-7.06 (m, 3H, H-2 + H-3 + H-4), 6.95 (d, *J* = 8.7 Hz, 2H, C_{arom}-H), 6.78 (d, *J* = 8.7 Hz, 2H, C_{arom}-H), 6.39-6.07 (m, 1H, H-7), 4.99 (d, *J* = 4.6 Hz, 1H, H-9), 4.56-4.12 (m, 2H, OCH_2), 3.77 (s, 3H, OCH_3), 2.80 (ddd, *J* = 16.1, 5.5, 3.4 Hz, 1H, H-8a), 2.66 (ddd, *J* = 16.1, 7.5, 2.3 Hz, 1H, H-8b), 1.49 (s, 9H, $C(CH_3)_3$), 1.35 (t, *J* = 7.1 Hz, 3H, $OCH_2C\mathbf{H}_3$). **^{13}C NMR** (75 MHz, $CDCl_3$) δ 163.7 (COO), 158.2 (C_{arom}-O), 134.6 (C-4a), 134.5 (C-9-**C**$_{arom}$), 132.4 (C-9a), 131.2 (C-6), 128.6 (2×C_{arom}-H), 128.0 (C-10a), 122.1 (C-3), 121.5 (C-10), 121.4 (C-2), 120.6 (C-7), 119.7 (C-1), 113.8 (2×C_{arom}-H), 111.9 (C-4), 61.6 (OCH_2), 55.3 (OCH_3), 37.4 (C-9), 33.7 ($\mathbf{C}(CH_3)_3$), 31.8 ($C(\mathbf{C}H_3)_3$), 30.9 (C-8), 14.3 ($OCH_2\mathbf{C}H_3$). **IR** (ATR): 2926 (C-H st), 1725 (C=O st), 1246 (C-O st as) cm^{-1}. **HRMS (ESI) m/z:** $[M+H]^+$ Calcd for $C_{26}H_{30}NO_3$ 404.2226; Found 404.2224. **M.p.**: 61-64 ºC (petroleum ether/EtOAc).

Ethyl (±)-10-benzyl-9-(4-methoxyphenyl)-8,9-dihydropyrido[1,2-a]indole-6-carboxylate (3j) and ethyl (±)-10-benzyl-6-(4-methoxyphenyl)-6,7-dihydropyrido[1,2-a]indole-9-carboxylate (4j). Following *GP-C*, **(±)-3j** (10 mg, 0.024 mmol, 47%) and **(±)-4j** (3 mg, 0.006 mmol, 13%) were isolated by FC (petroleum ether/EtOAc, from 25:1 to 19:1) on silica gel as white solids, starting from cyclopropane **1a** (12 mg, 0.05 mmol), indole **2j** (10 mg, 0.05 mmol) and employing diphenyl [(trifluoromethyl)sulfonyl]phosphoramidate (2 mg, 0.005 mmol) as catalyst in toluene (0.25 mL) at 100 ºC for 2 h in an oil bath.

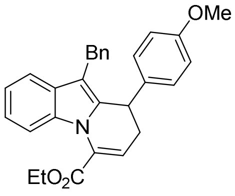

(±)-3j

^{1}H NMR (300 MHz, $CDCl_3$) δ 7.42 (d, *J* = 7.5 Hz, 1H, H-1), 7.22-7.02 (m, 8H, C_{arom}-H+ H-2 + H-3 + H-4), 6.99 (d, *J* = 8.7 Hz, 2H, C_{arom}-H), 6.75 (d, *J* = 8.7 Hz, 2H, C_{arom}-H), 6.27 (dd, *J* = 6.6, 3.8 Hz, 1H, H-7), 4.62-4.26 (m, 3H, H-9 + OCH_2), 4.0 (s, 2H, C-10-CH_2), 3.76 (s, 3H, OCH_3), 2.86-2.58 (m, 2H, H-8), 1.37 (t, *J* = 7.1 Hz, 3H, $OCH_2C\mathbf{H}_3$). **^{13}C NMR** (75 MHz, $CDCl_3$) δ 163.5 (COO), 158.4 (C_{arom}-O), 140.5 (C_{arom}-C), 134.9 (C-4a), 134.4 (C_{arom}-C), 133.8 (C-9a), 131.4 (C-6), 129.2 (C-10a), 128.6 (2× C_{arom}-H), 128.5 (2×C_{arom}-H), 128.3 (2×C_{arom}-H), 125.9 (C_{arom}-H), 122.1 (C-3), 120.5 (C-2), 120.2 (C-7), 119.2 (C-1), 113.9 (2× C_{arom}-H), 112.4 (C-4), 112.0 (C-10), 61.7 (OCH_2), 55.4 (OCH_3), 35.7 (C-9), 30.6 (C-8), 30.3 (C-10-**C**H_2), 14.4 (OCH_2CH_3). **IR** (ATR): 2903 (C-H st), 1724 (C=O st), 1247 (C-O st as) cm^{-1}. **HRMS (ESI) m/z:** $[M+H]^+$ Calcd for $C_{29}H_{28}NO_3$ 438.2069; Found 438.2065. **M.p.**: 54-57 ºC (petroleum ether/EtOAc).

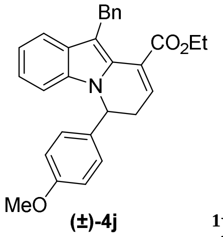

(±)-4j **^{1}H NMR** (300 MHz, $CDCl_3$) δ 7.45 (d, *J* = 7.8, 1H, H-1), 7.25-7.09 (m, 7H, C_{arom}-H + H-2 + H-3), 7.04-6.95 (m, 1H, H-4), 6.88 (d, *J* = 8.8 Hz, 2H, C_{arom}-H), 6.78 (d, *J* = 8.7 Hz, 2H, C_{arom}-H), 6.57 (dd, *J* = 7.2, 3.0 Hz, 1H, H-8), 5.67 (app d, *J* = 7.3 Hz, 1H, H-6), 4.30 (s, 2H, C-10-CH_2), 4.23-4.14 (m, 1H, OC**H_a**CH_3), 4.13-4.03 (m, 1H, OC**H_b**CH_3), 3.74 (s, 3H, OCH_3), 3.16 (ddd, *J* = 17.1, 7.3, 3.0 Hz, 1H, H-7a), 2.84 (ddd, *J* = 17.1, 7.2, 1.7 Hz, 1H, H-7b), 1.16 (t, *J* = 7.1 Hz, 3H, OCH_2C**H_3**). **^{13}C NMR** (75 MHz, $CDCl_3$) δ 166.4 (COO), 159.0 (C_{arom}-O), 141.7 (C_{arom}-C), 135.9 (C-4a), 133.1 (C_{arom}-C), 130.1 (C-8), 129.0 (C_{arom}-C), 128.8 (C_{arom}-C), 128.6 (2×C_{arom}-H), 128.3 (2×C_{arom}-H), 127.9 (C_{arom}-C), 126.9 (2×C_{arom}-H), 125.7 (C_{arom}-H), 123.1 (C_{arom}-H), 120.1 (C_{arom}-H), 119.7 (C_{arom}-H), 114.1 (2x C_{arom}-H), 112.8 (C-10), 109.2 (C_{arom}-H), 61.3 (OCH_2), 55.4 (OCH_3), 52.4 (C-6), 33.0 (C-7), 31.0 (C-10-**C**H_2), 14.1 (OCH_2**C**H_3). **IR** (ATR): 2927 (C-H st), 1720 (C=O st), 1250 (C-O st as) cm^{-1}. **MS** (EI) m/z (%): 438.2 (32), 437.2 (M^+, 100), 364.2 (20), 273.1 (34). **HRMS (ESI) m/z:** $[M+H]^+$ Calcd for $C_{29}H_{28}NO_3$ 438.2069; Found 438.2068. **M.p.**: 47-50 ºC (petroleum ether/EtOAc).

Ethyl (±)-10-allyl-9-(4-methoxyphenyl)-8,9-dihydropyrido[1,2-a]indole-6-carboxylate (3k) and ethyl (±)-10-allyl-6-(4-methoxyphenyl)-6,7-dihydropyrido[1,2-a]indole-9-carboxylate (4k). Following *GP-C*, **(±)-3k** (8 mg, 0.020 mmol, 36%) and **(±)-4k** (2 mg, 0.005 mmol, 22%) were isolated by FC (petroleum ether/EtOAc, from 25:1 to 19:1) on silica gel as orange solids, starting from cyclopropane **1a** (12 mg, 0.05 mmol), indole **2k** (8 mg, 0.05 mmol) and employing diphenyl [(trifluoromethyl)sulfonyl]phosphoramidate (2 mg, 0.005 mmol) as catalyst in toluene (0.25 mL) at 100 ºC for 2 h in an oil bath.

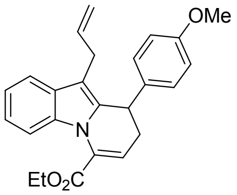

(±)-3k **^{1}H NMR** (300 MHz, $CDCl_3$) δ 7.56 (d, *J* = 6.7 Hz, 1H, H-1), 7.22-7.11 (m, 3H, H-2 + H-3 + H-4), 7.02 (d, *J* = 8.7 Hz, 2H, C_{arom}-H), 6.78 (d, *J* = 8.7 Hz, 2H, C_{arom}-H), 6.26 (dd, *J* = 6.9, 3.7 Hz, 1H, H-7), 5.82 (ddt, *J* = 16.4, 10.0, 6.2 Hz, 1H, C**H**=CH_2), 5.01 (dd, *J* = 16.4, 1.7 Hz, 1H, CH=C**H_a**H_b), 4.93 (dd, *J* = 10.0, 1.7 Hz, 1H, CH=CH_a**H_b**), 4.51 (dd, *J* = 6.0, 2.9 Hz, 1H, H-9), 4.47-4.32 (m, 2H, OCH_2), 3.76 (s, 3H, OCH_3), 3.38 (d, *J* = 6.2 Hz, 1H, C-10-CH_2), 2.84-2.60 (m, 2H, H-8), 1.36 (t, *J* = 7.1 Hz, 3H, OCH_2C**H_3**). **^{13}C NMR** (75 MHz, $CDCl_3$) δ 163.4 (COO), 158.4 (C_{arom}-O), 136.5 (**C**H=CH_2), 134.5 (C-4a), 134.3 (C_{arom}-C), 133.9 (C-9a), 131.4 (C-6), 129.1 (C-10a), 128.5 (2× C_{arom}-H), 122.0 (C-3), 120.4 (C-2), 120.1 (C-7), 118.9 (C1), 115.2 (CH=**C**H_2), 113.9 (2× C_{arom}-H), 112.1 (C-4), 111.3 (C-10), 61.7 (OCH_2), 55.3 (OCH_3), 35.6 (C-9), 30.5 (C-8), 28.7 (C-10-**C**H_2), 14.3 (OCH_2**C**H_3). **IR** (ATR): 2930 (C-H st), 1724 (C=O st), 1246 (C-O st as) cm^{-1}. **MS** (EI) m/z (%): 388.1 (27), 387.2 (M^+, 93), 314.1 (38), 312.1 (22), 298.0 (20), 273.1 (30), 272.1 (21), 254.0 (25), 242.1 (24), 241.0 (26), 228.0 (20), 206.1 (26), 205.1 (20), 204.0 (55), 180.0 (34), 167.1 (21), 127.9 (22), 127.0 (23), 121.0 (41), 115.1 (25), 108.0 (100), 78.1 (38), 77.0 (36), 65.0 (25). **HRMS (ESI) m/z:** $[M+Na]^+$ Calcd for $C_{25}H_{25}NO_3Na$ 410.1732 $[M+Na]^+$; found: 410.1734. **M.p.**: 79-82ºC (petroleum ether/EtOAc).

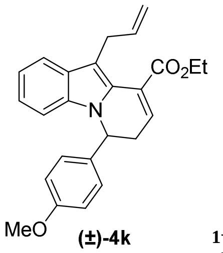

(±)-4k **^{1}H NMR** (300 MHz, $CDCl_3$) δ 7.64 (d, *J* = 7.7 Hz, 1H, H-1), 7.19-6.93 (m, 3H, H-2 + H-3 + H-4), 6.84 (d, *J* = 8.4 Hz, 2H, C_{arom}-H), 6.76 (d, *J* = 8.5 Hz, 2H, C_{arom}-H), 6.59 (d, *J* = 5.9 Hz, 1H, H-8), 6.34-5.76 (m, 1H, C**H**=CH_2), 5.65 (app d, *J* = 7.1 Hz, 1H, H-6), 5.31-4.69 (m, 2H, CH=C**H_2**), 4.49-4.10 (m, 2H, OCH_2), 3.73 (s, 3H, OCH_3), 3.66 (d, *J* = 6.2 Hz, 2H, C-10-CH_2), 3.28-2.97 (m, 1H, H-7a), 2.83 (dd, *J* = 17.2, 7.1 Hz, 1H, H-7b), 1.35 (t, *J* = 7.2 Hz, 3H, OCH_2C**H_3**). **^{13}C NMR** (75 MHz, $CDCl_3$) δ 166.3 (COO), 158.8 (C_{arom}-O), 138.0 (C-8), 135.8 (C-4a), 133.1 (C_{arom}-C), 130.0 (C_{arom}-H), 128.8 (C_{arom}-C), 128.7 (C_{arom}-C), 127.4 (C_{arom}-C), 126.9 (2× C_{arom}-H), 123.1 (C_{arom}-H), 120.0 (C_{arom}-H), 119.6 (C_{arom}-H), 114.8 (CH=**C**H_2), 114.1 (2× C_{arom}-H), 112.0 (C-10), 109.2 (C_{arom}-H), 61.3 (OCH_2), 55.3 (OCH_3), 52.4 (C-6), 33.0 (C-7), 29.6 (C-10-**C**H_2), 14.3 (OCH_2CH_3). **IR** (ATR): 2945 (C-H st), 1729 (C=O st), 1248 (C-O st as) cm^{-1}. **HRMS (ESI) m/z:** $[M+Na]^+$ Calcd for $C_{25}H_{25}NO_3Na$ 410.1732; Found 410.1729. **M.p.**: 85-88 °C (petroleum ether/EtOAc).

Ethyl (±)-9-(4-methoxyphenyl)-10-phenyl-8,9-dihydropyrido[1,2-a]indole-6-carboxylate (3l) and ethyl (±)-6-(4-methoxyphenyl)-10-phenyl-6,7-dihydropyrido[1,2-a]indole-9-carboxylate (4l). Following *GP-C*, **(±)-3l** (13 mg, 0.031 mmol, 62%) and **(±)-4l** (2 mg, 0.004 mmol, 7%) were isolated by FC (petroleum ether/EtOAc, from 25:1 to 19:1) on silica gel as white solids, starting from cyclopropane **1a** (12 mg, 0.05 mmol), indole **2l** (10 mg, 0.05 mmol) and employing diphenyl [(trifluoromethyl)sulfonyl]phosphoramidate (2 mg, 0.005 mmol) as catalyst in toluene (0.25 mL) at 100 °C for 2 h in an oil bath.

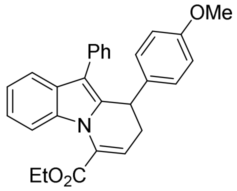

(±)-3l **^{1}H NMR** (300 MHz, $CDCl_3$) δ 7.87 (d, *J* = 7.5 Hz, 1H, H-1), 7.53-7.20 (m, 8H, C_{arom}-H + H-2 + H-3+ H-4), 7.14 (d, *J* = 8.1 Hz, 2H, C_{arom}-H), 6.89 (d, *J* = 8.3 Hz, 2H, C_{arom}-H), 6.41 (dd, *J* = 7.0, 3.6 Hz, 1H, H-7), 4.76-4.29 (m, 3H, H-9 + OCH_2), 3.83 (s, 3H, OCH_3), 3.12-2.57 (m, 2H, H-8), 1.45 (t, *J* = 7.1 Hz, 3H, OCH_2C**H_3**). **^{13}C NMR** (75 MHz, $CDCl_3$) δ 163.2 (COO), 158.3 (C_{arom}-O), 134.5 (C-4a + C_{arom}-C+ C-10-**C**$_{arom}$), 134.1 (C-9a), 131.2 (C-6), 129.4 (2×C_{arom}-H), 128.5 (4×C_{arom}-H), 127.9 (C-10a), 126.5 (C_{arom}-H), 122.4 (C-3), 122.2 (C-2), 121.1 (C-7), 119.5 (C-1), 115.9 (C-10), 114.1 (2× C_{arom}-H), 112.1 (C-4), 61.7 (OCH_2), 55.2 (OCH_3), 35.6 (C-9), 30.9 (C-8), 14.3 (OCH_2**C**H_3). **IR** (ATR): 2981 (C-H st), 1724 (C=O st), 1246 (C-O st as) cm^{-1}. **MS** (EI) m/z (%): 424.2 (30), 423.2 (M^+, 100), 351.2 (17), 350.2 (64), 349.2 (24), 348.2 (22). **HRMS (ESI) m/z:** $[M+H]^+$ Calcd for $C_{28}H_{26}NO_3$ 424.1913; Found 424.1894. **M.p.**: 80-83 °C (petroleum ether/EtOAc).

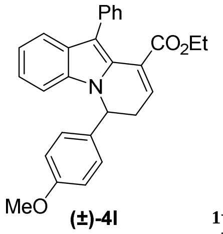

(±)-4l

^{1}H NMR (300 MHz, $CDCl_3$) δ 7.79 (d, *J* = 7.8 Hz, 1H, H-1), 7.63-7.39 (m, 4H, C_{arom}-H), 7.38-7.28 (m, 1H, C_{arom}-H), 7.24-7.07 (m, 3H, C_{arom}-H), 6.93 (d, *J* = 8.3 Hz, 2H, C_{arom}-H), 6.80 (d, *J* = 8.4 Hz, 2H, C_{arom}-H), 6.64 (dd, *J* = 7.2, 2.8 Hz, 1H, H-8), 5.72 (app d, *J* = 7.1 Hz, 1H, H-6), 3.82-3.66 (m, 4H, OCH_3 + OC**H$_a$**H$_b$CH$_3$), 3.35-3.16 (m, 2H, H-7a + OCH$_a$**H$_b$**CH$_3$), 2.90 (dd, *J* = 17.0, 7.1 Hz, 1H, H-7b), 0.85 (t, *J* = 7.1 Hz, 3H, OCH$_2$C**H$_3$**). **^{13}C NMR** (75 MHz, $CDCl_3$) δ 166.8 (COO), 159.1 (C_{arom}-O), 136.0 (C-4a), 135.7 (C_{arom}-C), 132.7 (C_{arom}-C), 130.7 (C-8), 129.6 (2×C_{arom}-H), 129.1 (C_{arom}-C), 128.6 (2×C_{arom}-H), 127.8 (C_{arom}-C), 127.6 (C_{arom}-C), 127.1 (2× C_{arom}-H), 126.4 (C_{arom}-H), 123.4 (C_{arom}-H), 120.5 (C_{arom}-H), 120.2 (C_{arom}-H), 116.1 (C-10), 114.2 (2× C_{arom}-H), 109.5 (C_{arom}-H), 61.1 (OCH_2), 55.4 (OCH_3), 52.7 (C-6), 33.1 (C-7), 13.7 (OCH$_2$**C**H$_3$). **IR** (ATR): 2939 (C-H st), 1726 (C=O st), 1243 (C-O st as) cm^{-1}. **HRMS (ESI) m/z:** $[M+H]^+$ Calcd for $C_{28}H_{26}NO_3$ 424.1913; Found 424.1915. **M.p.**: 56-59 ºC (petroleum ether/EtOAc).

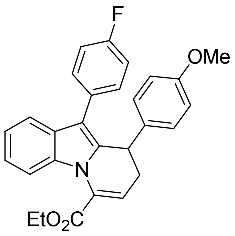

(±)-3m

Ethyl (±)-10-(4-fluorophenyl)-9-(4-methoxyphenyl)-8,9-dihydropyrido[1,2-a]indole-6-carboxylate (3m). Following *GP-C*, **(±)-3m** (15 mg, 0.033 mmol, 73%) was isolated by FC (petroleum ether/EtOAc, 25:1) on silica gel as a white solid, from cyclopropane **1a** (12 mg, 0.05 mmol), indole **2m** (11 mg, 0.05 mmol) and employing diphenyl [(trifluoromethyl)sulfonyl] phosphoramidate (2 mg, 0.005 mmol) as catalyst in toluene (0.25 mL) at 100 ºC for 2 h in an oil bath. R_f= 0.70 (petroleum ether/EtOAc, 9:1).

^{1}H NMR (300 MHz, $CDCl_3$) δ 7.62 (d, *J* = 6.8 Hz, 1H, H-1), 7.38-7.18 (m, 5H, C_{arom}-H), 7.17-6.97 (m, 4H, C_{arom}-H), 6.83 (d, *J* = 8.7 Hz, 2H, C_{arom}-H), 6.35 (dd, *J* = 7.0, 3.4 Hz, 1H, H-7), 4.73-4.23 (m, 3H, H-9 + OCH_2), 3.79 (s, 3H, OCH_3), 2.93-2.56 (m, 2H, H-8), 1.39 (t, *J* = 7.1 Hz, 3H, OCH$_2$C**H$_3$**). **^{13}C NMR** (75 MHz, $CDCl_3$) δ 163.4 (COO), 161.8 (d, $^1J_{C-F}$ = 245.5 Hz, C_{arom}-F), 158.4 (C_{arom}-O), 134.5 (C-4a), 134.4 (C_{arom}-C), 134.3 (C-9a), 131.3 (C-6), 130.9 (d, $^3J_{C-F}$ = 7.9 Hz, 2× C_{arom}-H), 130.1 (d, $^4J_{C-F}$ = 3.3 Hz, C_{arom}-C), 128.6 (2× C_{arom}-H), 127.9 (C-10a), 122.5 (C-3), 122.1 (C-2), 121.3 (C-7), 119.3 (C-1), 115.5 (d, $^2J_{C-F}$ = 21.3 Hz, 2× C_{arom}-H), 115.1 (C-10), 114.2 (2× C_{arom}-H), 112.2 (C-4), 61.8 (OCH_2), 55.3 (OCH_3), 35.7 (C-9), 30.9 (C-8), 14.4 (OCH$_2$**C**H$_3$). **IR** (ATR): 2935 (C-H st), 1724 (C=O st), 1243 (C-O st as), 1225 (C-F st) cm^{-1}. **MS** (EI) m/z (%): 442.2 (30), 441.2 (M^+, 100), 369.1 (18), 368.1 (73), 367.2 (35), 366.1 (33), 324.1 (22), 322.1 (25), 261.0 (34), 260.0 (22), 259.0 (34). **HRMS (ESI) m/z:** $[M+H]^+$ Calcd for $C_{28}H_{25}NO_3F$ 442.1818; Found 442.1814. **M.p.**: 90-93 ºC (petroleum ether/EtOAc).

Ethyl (±)-9,10-bis(4-methoxyphenyl)-8,9-dihydropyrido[1,2-a]indole-6-carboxylate (3n). Following *GP-C*, **(±)-3n** (18 mg, 0.040 mmol, 79%) was isolated by FC (petroleum ether/EtOAc, 25:1) on silica gel as a white solid, from cyclopropane **1a** (12 mg, 0.05 mmol), indole **2n** (11 mg, 0.05 mmol)

and employing diphenyl [(trifluoromethyl)sulfonyl]phosphoramidate (2 mg, 0.005 mmol) as catalyst in toluene (0.25 mL) at 100 ºC for 2 h in an oil bath. R_f= 0.70 (petroleum ether/EtOAc, 9:1).

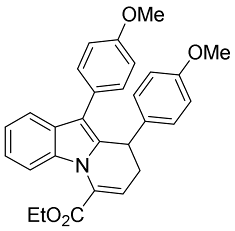

(±)-3n

^{1}H NMR (300 MHz, $CDCl_3$) δ 7.76 (d, *J* = 6.9 Hz, 1H, H-1), 7.39-7.17 (m, 5H, C_{arom}-H), 7.07 (d, *J* = 8.7 Hz, 2H, C_{arom}-H), 6.92 (d, *J* = 8.7 Hz, 2H, C_{arom}-H), 6.83 (d, *J* = 8.7 Hz, 2H, C_{arom}-H), 6.33 (dd, *J* = 7.0, 3.4 Hz, 1H, H-7), 4.86-4.28 (m, 3H, H-9 + OCH_2), 3.83 (s, 3H, OCH_3), 3.80 (s, 3H, OCH_3), 2.89-2.47 (m, 2H, H-8), 1.39 (t, *J* = 7.1 Hz, 3H, OCH_2**CH_3**). **^{13}C NMR** (75 MHz, $CDCl_3$) δ 163.4 (COO), 158.4 (C_{arom}-O), 158.3 (C_{arom}-O), 134.6 (C-4a), 134.5 (C_{arom}-C), 134.0 (C-9a), 131.3 (C-6), 129.5 (2×C_{arom}-H), 128.6 (2× C_{arom}-H), 128.2 (C_{arom}-C), 126.5 (C-10a), 122.4 (C-3), 122.0 (C-2), 121.1 (C-7), 119.5 (C-1), 115.6 (C-10), 114.1 (2× C_{arom}-H), 114.0 (2× C_{arom}-H), 112.1 (C-4), 61.8 (OCH_2), 55.4 (OCH_3), 55.3 (OCH_3), 35.7 (C-9), 31.0 (C-8), 14.4 (OCH_2CH_3). **IR** (ATR): 2959 (C-H st), 1724 (C=O st), 1245 (C-O st as) cm^{-1}. **MS** (EI) m/z (%): 454.2 (27), 453.2 (M^+, 100), 379.2 (17), 380.2 (69). **HRMS (ESI) m/z:** $[M+H]^+$ Calcd for $C_{29}H_{28}NO_4$ 454.2018; Found 454.2016. **M.p.**: 84-87 ºC (petroleum ether/EtOAc).

2.3.2 Synthesis of dihydropyridoindoles 5

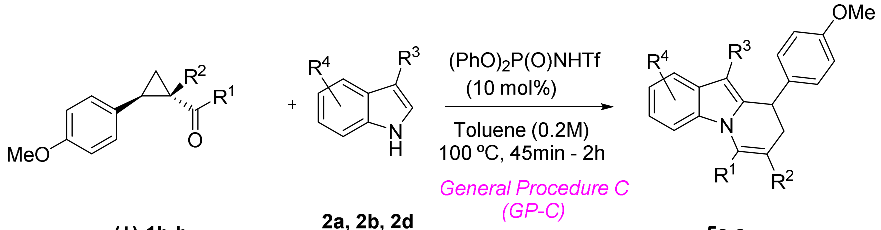

Scheme S4. ***General Procedure C for the synthesis of dihydropiridoindoles (±)-5a-s***

General Procedure C (GP-C) for the cyclocondensation: An oven-dried 5 mL screw-capped test tube containing a stirring bar was charged with the cyclopropane **1b-h** (1eq.) and the corresponding indole **2** (1eq.) and dissolved in toluene (0.2 M) under Ar. After adding the diphenyl [(trifluoromethyl)sulfonyl]phosphoramidate catalyst (10 mol%) the mixture was stirred at 100 ºC in an oil bath. When the reaction was judged complete (monitored by TLC), it was warmed to room temperature, quenched by addition of aq. $NaHCO_3$ (sat) (3 mL) and diluted with EtOAc (3 mL). The phases were separated and the aqueous phase was extracted with EtOAc (3×3 mL). The combined organic layers were dried over Na_2SO_4, filtered and evaporated. The residue was purified by column chromatography on silica gel to afford the corresponding pure compounds **5a-s**.

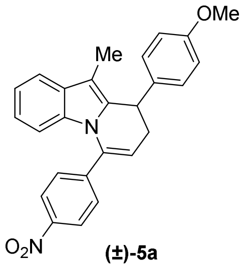

(±)-5a **(±)-9-(4-Methoxyphenyl)-10-methyl-6-(4-nitrophenyl)-8,9-dihydropyrido[1,2-a]indole (5a).** Following *GP-C*, **(±)-5a** (13 mg, 0.031 mmol, 80%) was isolated by FC (petroleum ether/EtOAc, 25:1) on silica gel as a white solid, from cyclopropane **1b** (15 mg, 0.05 mmol) and indole **2a** (7 mg, 0.05 mmol) under diphenyl [(trifluoromethyl)sulfonyl] phosphoramidate (2 mg, 0.005 mmol) in toluene (0.25 mL) at 100 ºC for 2 h in an oil bath. R_f= 0.70 (petroleum ether/EtOAc, 9:1). **^{1}H NMR** (300 MHz, $CDCl_3$) δ 8.26 (d, *J* = 8.7 Hz, 2H, H-3''), 7.64-7.46 (m, 3H, H-2'' + H-1), 7.17-7.03 (m, 3H, H-2' + H-2), 6.92 (app t, *J* = 7.7 Hz, 1H, H-3), 6.81 (d, *J* = 8.7 Hz, 2H, H-3'), 6.25 (d, *J* = 8.3 Hz, 1H, H-4), 5.48 (dd, *J* = 6.5, 3.9 Hz, 1H, H-7), 4.76-4.41 (m, 1H, H-9), 3.76 (s, 3H, OCH_3), 3.04-2.46 (m, 2H, H-8), 2.21 (s, 3H, CCH_3). **^{13}C NMR** (75 MHz, $CDCl_3$) δ 158.4 (C_{arom}-O), 147.8 (C_{arom}-N), 142.9 (C_{arom}-C), 137.4 (C-4a), 135.0 (C_{arom}-C), 134.2 (C-9a), 133.9 (C-6), 130.2 (C-10a), 128.4 (4×C_{arom}-H), 123.9 (2×C_{arom}-H), 121.7 (C-3), 120.2 (C-2), 118.8 (C-1), 114.0 (2× C_{arom}-H), 113.5 (C-7), 112.5 (C-4), 109.6 (C-10), 55.3 (OCH_3), 36.2 (C-9), 30.2 (C-8), 8.7 (C-10-**C**H_3). **IR** (ATR): 2930 (C-H st), 1510 (NO_2 st as), 1344 (NO_2 st sim) cm^{-1}. **MS** (EI) m/z (%): 411.2 (28), 410.2 (M^+, 100), 409.2 (20), 289.1 (14). **HRMS (ESI) m/z:** $[M+H]^+$ Calcd for $C_{26}H_{23}N_2O_3$ 411.1709; Found 411.1704. **M.p.**: 110-113 ºC (petroleum ether/EtOAc).

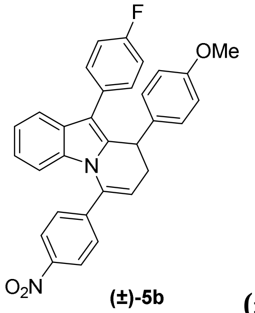

(±)-5b **(±)-10-(4-Fluorophenyl)-9-(4-methoxyphenyl)-6-(4-nitrophenyl)-8,9-dihydropyrido[1,2-a]indole (5b).** Following *GP-C*, **(±)-5b** (18 mg, 0.040 mmol, 80%) was isolated by FC (petroleum ether/EtOAc, 25:1) on silica gel as a white solid, from cyclopropane **1b** (15 mg, 0.05 mmol) and indole **2m** (11 mg, 0.05 mmol) under diphenyl [(trifluoromethyl)sulfonyl]phosphoramidate (2 mg, 0.005 mmol) in toluene (0.25 mL) at 100 ºC for 2 h in an oil bath. R_f= 0.70 (petroleum ether/EtOAc, 9:1). **^{1}H NMR** (300 MHz, $CDCl_3$) δ 8.30 (d, *J* = 8.6 Hz, 2H, C_{arom}-H), 7.74 (d, *J* = 7.9 Hz, 1H, H-1), 7.64 (d, *J* = 7.6 Hz, 2H, C_{arom}-H), 7.33 (dd, *J* = 8.6, 5.5 Hz, 2H, C_{arom}-H), 7.23-6.92 (m, 6H, C_{arom}-H), 6.83 (d, *J* = 8.7 Hz, 2H, C_{arom}-H), 6.38 (d, *J* = 8.4 Hz, 1H, H-4), 5.58 (dd, *J* = 6.8, 3.6 Hz, 1H, H-7), 4.51 (dd, *J* = 5.3, 2.5 Hz, 1H, H-9), 3.78 (s, 3H, OCH_3), 2.96-2.67 (m, 2H, H-8). **^{13}C NMR** (75 MHz, $CDCl_3$) δ 161.9 (d, $^1J_{C\text{-}F}$ = 246.0 Hz, C_{arom}-C), 158.5 (C_{arom}-C), 147.9 (C_{arom}-C), 142.6 (C-6), 137.4 (C_{arom}-C), 135.4 (C_{arom}-C), 134.6 (2×C_{arom}-C), 134.4 (C_{arom}-C), 130.9 (d, $^3J_{C\text{-}F}$ = 7.9 Hz, 2× C_{arom}-H), 130.0 (d, $^4J_{C\text{-}F}$ = 3.2 Hz, C_{arom}-C), 128.3 (3×C_{arom}-H), 128.2 (C_{arom}-H), 124.1 (2×C_{arom}-H), 122.3 (C-3), 121.2 (C-2), 119.6 (C-1), 115.5 (d, $^2J_{C\text{-}F}$ = 21.5 Hz, 2× C_{arom}-H), 115.4 (C-7), 115.3 (C-10), 114.2 (2×

C_{arom}-H), 112.8 (C-4), 55.3 (OCH_3), 36.1 (C-9), 30.8 (C-8). **IR** (ATR): 2951 (C-H st), 1505 (NO_2 st as), 1340 (NO_2 st sim), 1221 (C-F st) cm^{-1}. **HRMS (ESI) m/z:** $[M+H]^+$ Calcd for $C_{31}H_{24}N_2O_3F$ 491.1771; Found 491.1771. **M.p.**: 237-240 ºC (petroleum ether/EtOAc).

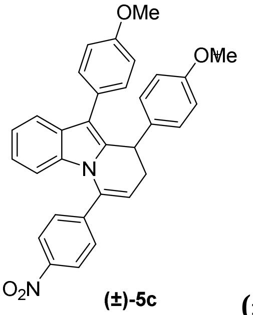

(±)-5c **(±)-9,10-Bis(4-methoxyphenyl)-6-(4-nitrophenyl)-8,9-dihydropyrido[1,2-a]indole (5c).** Following *GP-C*, **(±)-5c** (23 mg, 0.046 mmol, 92%) was isolated by FC (petroleum ether/EtOAc, 25:1) on silica gel as a white solid, from cyclopropane **1b** (15 mg, 0.05 mmol) and indole **2n** (11 mg, 0.05 mmol) under diphenyl [(trifluoromethyl)sulfonyl] phosphoramidate (2 mg, 0.005 mmol) in toluene (0.25 mL) at 100 ºC for 2 h in an oil bath. R_f= 0.70 (petroleum ether/EtOAc, 9:1). **^{1}H NMR** (300 MHz, $CDCl_3$) δ 8.30 (d, J = 8.3 Hz, 2H, C_{arom}-H), 7.79 (d, J = 7.8 Hz, 1H, H-1), 7.64 (d, J = 8.3 Hz, 2H, C_{arom}-H), 7.33 (d, J = 8.7 Hz, 2H, C_{arom}-H), 7.21-7.09 (m, 3H, C_{arom}-H), 7.06-6.92 (m, 3H, C_{arom}-H), 6.85 (d, J = 8.7 Hz, 2H, C_{arom}-H), 6.39 (d, J = 8.3 Hz, 1H, H-4), 5.57 (dd, J = 6.7, 3.4 Hz, 1H, H-7), 4.55 (dd, J = 5.2, 2.4 Hz, 1H, H-9), 3.85 (s, 3H, OCH_3), 3.79 (s, 3H, OCH_3), 2.96-2.66 (m, 2H, H-8). **^{13}C NMR** (75 MHz, $CDCl_3$) δ 158.5 (C_{arom}-C), 158.4 (C_{arom}-C), 147.8 (C_{arom}-C), 142.7 (C-6), 137.4 (C_{arom}-C), 134.93 (C_{arom}-C), 134.85 (C_{arom}-C), 134.4 (C_{arom}-C), 130.5 (2×C_{arom}-H), 128.5 (C_{arom}-C), 128.3 (2×C_{arom}-H), 128.2 (2×C_{arom}-H), 126.4 (C-10a), 124.0 (2×C_{arom}-H), 122.1 (C-3), 120.9 (C-2), 119.8 (C-1), 115.9 (C-10), 115.3 (C-7), 114.1 (4×C_{arom}-H), 112.7 (C-4), 55.4 (OCH_3), 55.3 (OCH_3), 36.1 (C-9), 30.8 (C-8). **IR** (ATR): 2940 (C-H st), 1509 (NO_2 st as), 1343 (NO_2 st sim) cm^{-1}. **HRMS (ESI) m/z:** $[M+H]^+$ Calcd for $C_{32}H_{27}N_2O_4$ 503.1971; Found 503.1962. **M.p.**: 122-125 ºC (petroleum ether/EtOAc).

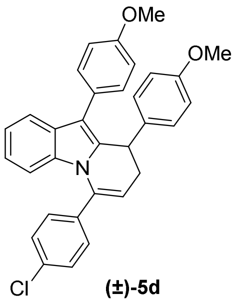

(±)-5d **(±)-6-(4-Chlorophenyl)-9,10-bis(4-methoxyphenyl)-8,9-dihydropyrido[1,2-a]indole (5d).** Following *GP-C*, **(±)-5d** (22 mg, 0.045 mmol, 90%) was isolated by FC (petroleum ether/EtOAc, 25:1) on silica gel as a white solid, from cyclopropane **1c** (14 mg, 0.05 mmol) and indole **2n** (11 mg, 0.05 mmol) under diphenyl [(trifluoromethyl)sulfonyl] phosphoramidate (2 mg, 0.005 mmol) in toluene (0.25 mL) at 100 ºC for 2 h in an oil bath. R_f= 0.70 (petroleum ether/EtOAc, 9:1). **^{1}H NMR** (300 MHz, $CDCl_3$) δ 7.76 (d, J = 7.9 Hz, 1H, H-1), 7.46-7.35 (m, 4H, C_{arom}-H), 7.31 (d, J = 8.7 Hz, 2H, C_{arom}-H), 7.18-7.05 (m, 3H, C_{arom}-H), 7.05-6.88 (m, 3H, C_{arom}-H), 6.83 (d, J = 8.7 Hz, 2H, C_{arom}-H), 6.45 (d, J = 8.3 Hz, 1H, H-4), 5.37 (dd, J = 7.2, 3.1 Hz, 1H, H-7), 4.66-4.40 (m, 1H, H-9), 3.84 (s, 3H, OCH_3), 3.78 (s, 3H, OCH_3), 2.78 (ddd, J = 15.8, 5.5, 3.1 Hz, 1H, H-8a), 2.67 (ddd, J = 15.8, 7.2, 2.2 Hz, 1H, H-8b). **^{13}C NMR** (75 MHz, $CDCl_3$) δ 158.4 (C_{arom}-C), 158.3 (C_{arom}-C), 138.1 (C-6), 135.3 (C_{arom}-Cl), 135.2 (C_{arom}-C), 135.0 (C_{arom}-C), 134.7 (C_{arom}-C), 134.5 (C_{arom}-C), 130.6 (2×C_{arom}-H), 129.0 (4×C_{arom}-H), 128.5

(2×C_{arom}-H), 128.4 (C_{arom}-C), 126.7 (C-10a), 121.8 (C-3), 120.6 (C-2), 119.5 (C-1), 115.5 (C-10), 114.1 (2× C_{arom}-H), 114.0 (2× C_{arom}-H), 113.1 (C-7), 112.0 (C-4), 55.4 (OCH_3), 55.3 (OCH_3), 36.2 (C-9), 30.5 (C-8). **IR** (ATR): 2947 (C-H st), 1645 (arC-C), 1608 (arC-C), 1034 (C-Cl st) cm^{-1}. **HRMS (ESI) m/z:** $[M+H]^+$ Calcd for $C_{32}H_{27}ClNO_2$ 492.1725; Found 492.1729. **M.p.**: 100-103 ºC (petroleum ether/EtOAc).

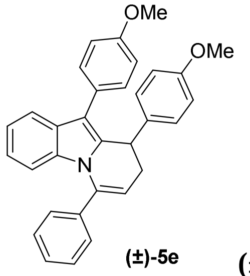

(±)-5e

(±)-9,10-Bis(4-methoxyphenyl)-6-phenyl-8,9-dihydropyrido[1,2-a]indole (5e). Following *GP-C*, **(±)-5e** (19 mg, 0.043 mmol, 85%) was isolated by FC (petroleum ether/EtOAc, 25:1) on silica gel as a white solid, from cyclopropane **1d** (13 mg, 0.05 mmol) and indole **2n** (11 mg, 0.05 mmol) under diphenyl [(trifluoromethyl)sulfonyl]phosphoramidate (2 mg, 0.005 mmol) in toluene (0.25 mL) at 100 ºC for 2 h in an oil bath. R_f= 0.70 (petroleum ether/EtOAc, 9:1). **^{1}H NMR** (300 MHz, $CDCl_3$) δ 7.75 (d, *J* = 7.9 Hz, 1H, H-1), 7.57-7.36 (m, 5H, C_{arom}-H), 7.32 (d, *J* = 8.6 Hz, 2H, C_{arom}-H), 7.21-7.07 (m, 3H, C_{arom}-H), 6.99-6.90 (m, 3H, C_{arom}-H), 6.83 (d, *J* = 8.6 Hz, 2H, C_{arom}-H), 6.41 (d, *J* = 8.4 Hz, 1H, H-4), 5.36 (dd, *J* = 7.3, 3.0 Hz, 1H, H-7), 4.51 (app d, *J* = 5.2 Hz, 1H, H-9), 3.85 (s, 3H, OCH_3), 3.78 (s, 3H, OCH_3), 2.80 (ddd, *J* = 15.8, 5.5, 3.1 Hz, 1H, H-8a), 2.66 (ddd, *J* = 15.8, 7.3, 2.1 Hz, 1H, H-8b). **^{13}C NMR** (75 MHz, $CDCl_3$) δ 158.4 (C_{arom}-O), 158.2 (C_{arom}-O), 139.1 (C-6), 136.7 (C_{arom}-C), 135.5 (C_{arom}-C), 135.3 (C_{arom}-C), 134.8 (C_{arom}-C), 130.6 (2×C_{arom}-H), 128.6 (5×C_{arom}-H), 128.4 (C_{arom}-C), 127.8 (2×C_{arom}-H), 126.9 (C-10a), 121.6 (C-3), 120.5 (C-2), 119.4 (C-1), 115.3 (C-10), 114.1 (2× C_{arom}-H), 114.0 (2× C_{arom}-H), 113.3 (C-7), 111.2 (C-4), 55.4 (OCH_3), 55.3 (OCH_3), 36.2 (C-9), 30.5 (C-8). **IR** (ATR): 2950 (C-H st), 1645 (arC-C), 1608 (arC-C) cm^{-1}. **HRMS (ESI) m/z:** $[M+H]^+$ Calcd for $C_{32}H_{28}NO_2$ 458.2120; Found 458.2100. **M.p.**: 89-92 ºC (petroleum ether/EtOAc).

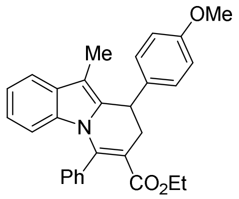

(±)-5f

Ethyl (±)-9-(4-methoxyphenyl)-10-methyl-6-phenyl-8,9-dihydropyrido[1,2-a]indole-7-carboxylate (5f). Following *GP-C*, **(±)-5f** (21 mg, 0.047 mmol, 94%) was isolated by FC (petroleum ether/EtOAc, 25:1) on silica gel as a white solid, from cyclopropane **1e** (16 mg, 0.05 mmol) and indole **2a** (7 mg, 0.05 mmol) under diphenyl [(trifluoromethyl)sulfonyl]phosphoramidate (2 mg, 0.005 mmol) in toluene (0.25 mL) at 100 ºC for 45 min in an oil bath. R_f= 0.70 (petroleum ether/EtOAc, 9:1). **^{1}H NMR** (300 MHz, $CDCl_3$) δ 7.59-7.33 (m, 5H, C_{arom}-H), 7.25-7.02 (m, 4H, C_{arom}-H + H-2), 6.92-6.63 (m, 3H, H-3 + C_{arom}-H), 5.70 (d, *J* = 8.5 Hz, 1H, H-4), 4.53 (app t, *J* = 4.7 Hz, 1H, H-9), 4.02-3.77 (m, 2H, OCH_2), 3.79 (s, 3H, OCH_3), 3.27 (dd, *J* = 15.5, 3.9 Hz, 1H, H-8a), 3.03 (dd, *J* = 15.5, 5.4 Hz, 1H, H-8b), 2.13 (s, 3H, C-10-CH_3), 0.87 (t, *J* = 7.1 Hz, 3H, $OCH_2C\mathbf{H}_3$). **^{13}C NMR** (75 MHz, $CDCl_3$) δ 168.0 (COO), 158.4 (C_{arom}-O), 146.1 (C-6), 136.0 (C_{arom}-C), 135.5 (C_{arom}-C), 135.2 (C_{arom}-C), 133.6 (C_{arom}-C), 131.3 (C-10a), 129.2 (2× C_{arom}-H), 128.5 (5×C_{arom}-H), 122.3 (C-3), 121.1 (C-2), 118.4 (C-1), 114.0 (2× C_{arom}-H), 113.6 (C-4), 111.1 (C-10), 109.1 (C-7), 60.0 (OCH_2), 55.4 (OCH_3), 36.2 (C-9), 31.0 (C-8), 13.8 ($OCH_2\mathbf{C}H_3$), 8.7 (C-10-$\mathbf{C}H_3$). **IR** (ATR): 2980 (C-H st), 1683 (C=O st), 1239 (C-O st as)

cm^{-1}. **MS** (EI) m/z (%): 438.2 (23), 437.2 (M$^+$, 74), 365.2 (23), 364.1 (100), 257.1 (19), 256.1 (50), 254.1 (27), 241.1 (21), 204.0 (24), 135.0 (35) 115.0 (52), 108.0 (38), 78.0 (23), 77.0 (48), 21.0 (65). **HRMS (ESI) m/z:** [M+Na]$^+$ Calcd for $C_{29}H_{27}NO_3Na$ 460.1889; Found 460.1889. **M.p.**: 121-123 ºC (petroleum ether/EtOAc).

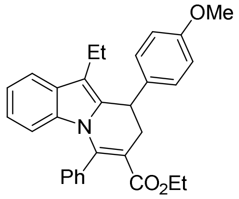

(±)-5g **Ethyl (±)-10-ethyl-9-(4-methoxyphenyl)-6-phenyl-8,9-dihydropyrido[1,2-a]indole-7-carboxylate (5g).** Following *GP-C*, **(±)-5g** (21 mg, 0.046 mmol, 92%) was isolated by FC (petroleum ether/EtOAc, 25:1) on silica gel as a white solid, from cyclopropane **1e** (16 mg, 0.05 mmol) and indole **2g** (7 mg, 0.05 mmol) under diphenyl [(trifluoromethyl)sulfonyl] phosphoramidate (2 mg, 0.005 mmol) in toluene (0.25 mL) at 100 ºC for 45 min in an oil bath. R_f= 0.70 (petroleum ether/EtOAc, 9:1). **^{1}H NMR** (300 MHz, $CDCl_3$) δ 7.59-7.34 (m, 5H, C_{arom}-H), 7.26-7.17 (m, 1H, C_{arom}-H), 7.17-6.99 (m, 3H, H-2 + C_{arom}-H), 6.88-6.69 (m, 3H, H-3 + C_{arom}-H), 5.72 (d, *J* = 8.4 Hz, 1H, H-4), 4.55 (dd, *J* = 5.4, 3.2 Hz, 1H, H-9), 4.02-3.59 (m, 2H, OCH_2), 3.78 (s, 3H, OCH_3),3.27 (dd, *J* = 15.4, 3.2 Hz, 1H, H-8a), 3.02 (dd, *J* = 15.4, 5.4 Hz, 1H, H-8b), 2.65 (m, 2H, C-10-CH_2), 1.12 (t, *J* = 7.5 Hz, 3H, $CH_2C\mathbf{H}_3$), 0.85 (t, *J* = 7.1 Hz, 3H, $CH_2C\mathbf{H}_3$). **^{13}C NMR** (75 MHz, $CDCl_3$) δ 168.0 (COO), 158.3 (C_{arom}-O), 146.1 (C-6), 136.0 (C-4a), 135.8 (C_{arom}-C), 134.6 (C_{arom}-C), 133.9 (C-9a), 130.3 (C-10a), 129.2 (2× C_{arom}-H), 128.4 (5×C_{arom}-H), 122.2 (C-3), 121.1 (C-2), 118.7 (C-1), 117.3 (C-10), 113.9 (2× C_{arom}-H), 113.7 (C-4), 109.1 (C-7), 60.0 (OCH_2), 55.4 (OCH_3), 35.9 (C-9), 31.1 (C-8), 17.3 (C-10-$\mathbf{C}H_2$), 14.8 ($CH_2\mathbf{C}H_3$), 13.8 ($CH_2\mathbf{C}H_3$). **MS** (EI) m/z (%): 452.2 (30), 451.2 (M$^+$, 100), 379.1 (31), 378.2 (86), 362.0 (19), 349.1 (24), 330.1 (20), 300.1 (26), 270.1 (31), 256.1 (34), 255.1 (19), 254.1 (43), 241.0 (33), 217.9 (29), 204.0 (36), 121.0 (50), 115.0 (84), 108.0 (87), 92.0 (21), 89.0 (23), 65.0 (29). **IR** (ATR): 2965 (C-H st), 1683 (C=O st), 1246 (C-O st as) cm^{-1}. **HRMS (ESI) m/z:** [M+Na]$^+$ Calcd for $C_{30}H_{29}NO_3Na$ 474.2045; Found 474.2053. **M.p.**: 123-125 ºC (petroleum ether/EtOAc).

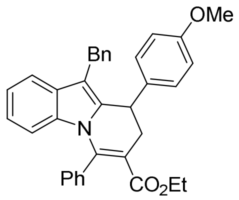

(±)-5h **Ethyl (±)-10-benzyl-9-(4-methoxyphenyl)-6-phenyl-8,9-dihydropyrido[1,2-a]indole-7-carboxylate (5h).** Following *GP-C*, **(±)-5h** (21 mg, 0.040 mmol, 81%) was isolated by FC (petroleum ether/EtOAc, 25:1) on silica gel as a white solid, from cyclopropane **1e** (16 mg, 0.05 mmol) and indole **2j** (10 mg, 0.05 mmol) under diphenyl [(trifluoromethyl)sulfonyl]phosphoramidate (2 mg, 0.005 mmol) in toluene (0.25 mL) at 100 ºC for 45 min in an oil bath. R_f= 0.70 (petroleum ether/EtOAc, 9:1). **^{1}H NMR** (300 MHz, $CDCl_3$) δ 7.57-7.39 (m, 4H, C_{arom}-H), 7.34 (d, *J* = 7.6 Hz, 1H, H-1), 7.23-7.05 (m, 8H, C_{arom}-H), 6.98 (app t, *J* = 7.5 Hz, 1H, H-2), 6.83-6.70 (m, 3H, H-3 + C_{arom}-H), 5.73 (d, *J* = 8.5 Hz, 1H, H-4), 4.57 (dd, *J* = 5.5, 3.3 Hz, 1 H, H-9), 3.99 (s, 1H, C-10-CH_2), 3.93-3.76 (m, 2H, OCH_2), 3.78 (s, 3H, OCH_3), 3.26 (dd, *J* = 15.5, 3.3 Hz, 1H, H-8a), 3.04 (dd, *J* = 15.5, 5.5 Hz, 1H, H-8b), 0.86 (t, *J* = 7.1 Hz, 3H, $OCH_2C\mathbf{H}_3$). **^{13}C NMR** (75 MHz, $CDCl_3$) δ 167.9 (COO), 158.4 (C_{arom}-O), 145.9 (C-6), 140.1 (C_{arom}-C), 136.1 (C_{arom}-C), 135.9 (C_{arom}-C), 135.8 (C-9a), 133.5 (C_{arom}-C), 130.5 (C-10a), 130.2

(C_{arom}-H), 129.3 (C_{arom}-H), 129.0 (C_{arom}-H), 128.5 (5×C_{arom}-H), 128.4 (2×C_{arom}-H), 126.0 (C_{arom}-H), 122.3 (C-3), 121.3 (C-2), 119.2 (C-1), 114.0 (3×C_{arom}-H), 113.7 (C-4), 109.5 (C-7 + C-10), 60.0 (OCH_2), 55.4 (OCH_3), 36.1 (C-9), 31.3 (C-8), 30.1 (C-10-**C**H_2-C_{arom}), 13.7 (OCH_2**C**H_3). **IR** (ATR): 2977 (C-H st), 1683 (C=O st), 1245 (C-O st as) cm^{-1}. **HRMS (ESI) m/z:** $[M+Na]^+$ Calcd for $C_{35}H_{31}NO_3Na$ 536.2202; Found 536.2202. **M.p.**: 135-138 ºC (petroleum ether/EtOAc).

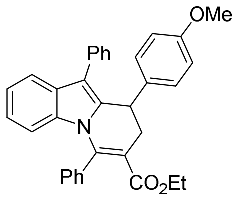

(±)-5i **Ethyl (±)-9-(4-methoxyphenyl)-6,10-diphenyl-8,9-dihydropyrido[1,2-a]indole-7-carboxylate (5i).** Following *GP-C,* **(±)-5i** (22 mg, 0.045 mmol, 90%) was isolated by FC (petroleum ether/EtOAc, 25:1) on silica gel as a white solid, from cyclopropane **1e** (16 mg, 0.05 mmol) and indole **2l** (10 mg, 0.05 mmol) under diphenyl [(trifluoromethyl)sulfonyl] phosphoramidate (2 mg, 0.005 mmol) in toluene (0.25 mL) at 100 ºC for 45 min in an oil bath. R_f= 0.70 (petroleum ether/EtOAc, 9:1). **^{1}H NMR** (300 MHz, $CDCl_3$) δ 7.71 (d, *J* = 7.9 Hz, 1H, H-1), 7.61-7.29 (m, 10H, C_{arom}-H), 7.22-7.04 (m, 3H, H-2 + C_{arom}-H), 6.93-6.80 (m, 3H, H-3 + C_{arom}-H), 5.86 (d, *J* = 8.5 Hz, 1H, H-4), 4.58 (dd, *J* = 5.3, 2.4 Hz, 1H, H-9), 4.06-3.76 (m, 2H, OCH_2), 3.82 (s, 3H, OCH_3), 3.29 (dd, *J* = 15.5, 2.4 Hz, 1H, H-8a), 3.0 (dd, *J* = 15.5, 5,3 Hz, 1H, H-8b), 0.88 (t, *J* = 7.1 Hz, 3H, OCH_2C**H**$_3$). **^{13}C NMR** (75 MHz, $CDCl_3$) δ 167.8 (COO), 158.3 (C_{arom}-O), 145.6 (C-6), 136.0 (C-4a), 135.8 (C_{arom}-C), 135.4 (C_{arom}-C), 134.1 (C-9a), 133.6 (C_{arom}-C), 129.5 (3×C_{arom}-H), 129.4 (C_{arom}-H), 128.6 (7×C_{arom}-H + C-10a), 126.9 (C_{arom}-H), 122.7 (C-3), 121.7 (C-2), 119.6 (C-1), 117.2 (C_{arom}-C), 114.1 (2× C_{arom}-H), 113.7 (C-4), 111.0 (C-7), 60.1 (OCH_2), 55.4 (OCH_3), 35.7 (C-9), 31.5 (C-8), 13.8 (OCH_2**C**H_3). **IR** (ATR): 2926 (C-H st), 1686 (C=O st), 1239 (C-O st as) cm^{-1}. **HRMS (ESI) m/z:** $[M+H]^+$ Calcd for $C_{34}H_{30}NO_3$ 500.2226; Found 500.2226. **M.p.**: 179-182 ºC (petroleum ether/EtOAc).

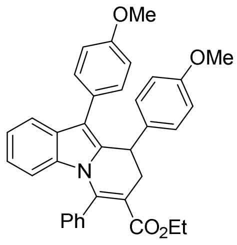

(±)-5j **Ethyl (±)-9,10-bis(4-methoxyphenyl)-6-phenyl-8,9-dihydropyrido[1,2-a]indole-7-carboxylate (5j).** Following *GP-C,* **(±)-5j** (23 mg, 0.043 mmol, 86%) was isolated by FC (petroleum ether/EtOAc, 25:1) on silica gel as a white solid, from cyclopropane **1e** (16 mg, 0.05 mmol) and indole **2n** (11 mg, 0.05 mmol) under diphenyl [(trifluoromethyl)sulfonyl] phosphoramidate (2 mg, 0.005 mmol) in toluene (0.25 mL) at 100 ºC for 45 min in an oil bath. R_f= 0.70 (petroleum ether/EtOAc, 9:1). **^{1}H NMR** (300 MHz, $CDCl_3$) δ 7.64 (d, *J* = 7.8 Hz, 1H, H-1), 7.56-7.23 (m, 7H, C_{arom}-H), 7.19-7.04 (m, 3H, H-2 + C_{arom}-H), 6.93 (d, *J* = 8.7 Hz, 2H, C_{arom}-H), 6.88-6.77 (m, 3H, H-3 + C_{arom}-H), 5.81 (d, *J* = 8.5 Hz, 1H, H-4), 4.52 (dd, *J* = 5.2, 2.5 Hz, 1H, H-9), 4.06-3.64 (m, 8H, OCH_2 + 2×OCH_3), 3.25 (dd, *J* = 15.5, 2.5 Hz, 1H, H-8a), 2.97 (dd, *J* = 15.5, 5.2 Hz, 1H, H-8b), 0.84 (t, *J* = 7.1 Hz, 3H, OCH_2C**H**$_3$). **^{13}C NMR** (75 MHz, $CDCl_3$) δ 167.8 (COO), 158.7 (C_{arom}-C), 158.3 (C_{arom}-O), 145.7 (C-6), 135.9 (C_{arom}-C), 135.8 (C_{arom}-C), 135.0 (C_{arom}-C), 134.2 (C-9a), 130.5 (3×C_{arom}-H), 129.7 (C_{arom}-C), 129.4 (C_{arom}-H), 128.5 (5×C_{arom}-H), 125.9 (C-10a), 122.6 (C-3), 121.6 (C-1), 119.5 (C-2), 116.9 (C-10), 114.1 (2× C_{arom}-H),

114.0 (2× C_{arom}-H), 113.7 (C-4), 110.7 (C-7), 60.1 (OCH_2), 55.4 (OCH_3), 55.3 (OCH_3), 35.7 (C-9), 31.5 (C-8), 13.7 (OCH_2**C**H_3). **IR** (ATR): 2955 (C-H st), 1685 (C=O st), 1263 (C-O st as) cm^{-1}. **HRMS (ESI) m/z:** $[M+H]^+$ Calcd for $C_{35}H_{32}NO_4$ 530.2331; Found 530.2325. **M.p.**: 197-200 ºC (petroleum ether/EtOAc).

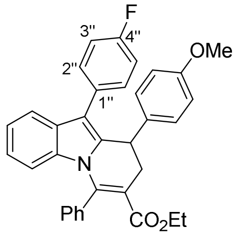

(±)-5k **Ethyl (±)-10-(4-fluorophenyl)-9-(4-methoxyphenyl)-6-phenyl-8,9-dihydropyrido[1,2-a]indole-7-carboxylate (5k).** Following *GP-C*, **(±)-5k** (21 mg, 0.041 mmol, 82%) was isolated by FC (petroleum ether/EtOAc, 25:1) on silica gel as a white solid, from cyclopropane **1e** (16 mg, 0.05 mmol) and indole **2m** (11 mg, 0.05 mmol) under diphenyl [(trifluoromethyl)sulfonyl]phosphoramidate (2 mg, 0.005 mmol) in toluene (0.25 mL) at 100 ºC for 45 min in an oil bath. R_f= 0.70 (petroleum ether/EtOAc, 9:1). **^{1}H NMR** (300 MHz, $CDCl_3$) δ 7.60 (d, *J* = 7.9 Hz, 1H, H-1), 7.57-7.38 (m, 4H, C_{arom}-H), 7.39-7.20 (m, 3H, C_{arom}-H), 7.19-7.00 (m, 5H, C_{arom}-H), 6.92-6.73 (m, 3H, C_{arom}-H), 5.83 (d, *J* = 8.5 Hz, 1H, H-4), 4.49 (dd, *J* = 5.4, 2.6 Hz, 1H, H-9), 3.95-3.69 (m, 2H, OCH_2), 3.80 (s, 3H, OCH_3), 3.24 (dd, *J* = 15.5, 2.6 Hz, 1H, H-8a), 2.98 (dd, *J* = 15.5, 5.4 Hz, 1H, H-8b), 0.85 (t, *J* = 7.1 Hz, 3H, OCH_2C**H_3**). **^{13}C NMR** (75 MHz, $CDCl_3$) δ 167.7 (COO), 162.0 (d, $^1J_{C-F}$ = 246.2 Hz, C_{arom}-F), 158.4 (C_{arom}-O), 145.5 (C-6), 135.9 (C_{arom}-C), 135.7 (C-4a), 135.4 (C_{arom}-C), 133.9 (C_{arom}-C), 131.2 (d, $^3J_{C-F}$ = 8.0 Hz, 2× C_{arom}-H), 129.6 (C-10a + C_{arom}-C), 129.4 (2×C_{arom}-H), 128.5 (5×C_{arom}-H), 122.7 (C-3), 121.8 (C-1), 119.3 (C-2), 116.2 (C-10), 115.6 (d, $^2J_{C-F}$ = 21.4 Hz, 2× C_{arom}-H), 114.1 (2× C_{arom}-H), 113.7 (C-4), 111.0 (C-7), 60.1 (OCH_2), 55.4 (OCH_3), 35.7 (C-9), 31.5 (C-8), 13.7 (OCH_2**C**H_3). **IR** (ATR): 2980 (C-H st), 1685 (C=O st), 1239 (C-O st as), 1221 (C-F st) cm^{-1}. **HRMS (ESI) m/z:** $[M+H]^+$ Calcd for $C_{34}H_{29}NO_3F$ 518.2131; Found 518.2139. **M.p.**: 175-178 ºC (petroleum ether/EtOAc).

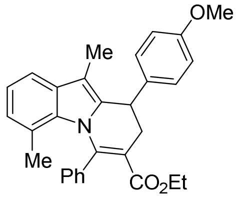

(±)-5l **Ethyl (±)-9-(4-methoxyphenyl)-4,10-dimethyl-6-phenyl-8,9-dihydropyrido[1,2-a]indole-7-carboxylate (5l).** Following *GP-C*, **(±)-5l** (12 mg, 0.027 mmol, 54%) was isolated by FC (petroleum ether/EtOAc, 25:1) on silica gel as a white solid, from cyclopropane **1e** (16 mg, 0.05 mmol) and indole **2b** (7 mg, 0.05 mmol) under diphenyl [(trifluoromethyl)sulfonyl]phosphoramidate (2 mg, 0.005 mmol) in toluene (0.25 mL) at 100 ºC for 45 min in an oil bath. R_f= 0.70 (petroleum ether/EtOAc, 9:1). **^{1}H NMR** (300 MHz, $CDCl_3$) δ 7.38-7.28 (m, 2H, C_{arom}-H), 7.26-7.19 (m, 2H, C_{arom}-H), 7.15-7.00 (m, 5H, C_{arom}-H), 6.87-6.67 (m, 3H, C_{arom}-H), 4.42 (app t, *J* = 5.2 Hz, 1H, H-9), 3.84 (m, 2H, OCH_2), 3.75 (s, 3H, OCH_3), 3.22-2.79 (m, 2H, H-8), 2.00 (s, 3H, C_{arom}-CH_3), 1.43 (s, 3H, C_{arom}-CH_3), 0.86 (t, *J* = 7.1 Hz, 3H, OCH_2C**H_3**). **^{13}C NMR** (75 MHz, $CDCl_3$) δ 169.0 (COO), 158.4 (C_{arom}-O), 144.7 (C-6), 139.1 (C_{arom}-C), 136.4 (C_{arom}-C), 135.6 (C_{arom}-C), 133.3 (C_{arom}-C), 132.6 (C-10a), 129.9 (2×C_{arom}-H), 129.0 (C_{arom}-H), 128.9 (2×C_{arom}-H), 127.6 (2×C_{arom}-H), 125.7 (C-2), 124.5 (C-4), 121.8 (C-3), 115.9 (C-

1), 113.9 (C_{arom}-H), 113.0 (C-10), 110.9 (C-7), 60.2 (OCH_2), 55.3 (OCH_3), 40.5 (C-9), 34.1 (C-8), 20.6 (C_{arom}-CH_3), 13.8 (OCH_2CH_3), 9.2 (C-10-CH_3). **MS** (EI) m/z (%): 452.1 (27), 451.2 (M^+, 74), 379.1 (22), 378.2 (100), 363.2 (21), 270.0 (44), 254.9 (17), 253.9 (25), 248.0 (17), 218.1 (25), 216.9 (18), 204.1 (23), 135.0 (52), 121.0 (31), 115.9 (18), 115.0 (90), 108.0 (50), 78.0 (24), 77.0 (36). **IR** (ATR): 2926 (C-H st), 1692 (C=O st), 1247 (C-O st as) cm^{-1}. **HRMS (ESI) m/z:** $[M+H]^+$ Calcd for $C_{30}H_{29}NO_3Na$ 474.2045; Found 474.2041. **M.p.**: 132-135 ºC (petroleum ether/EtOAc).

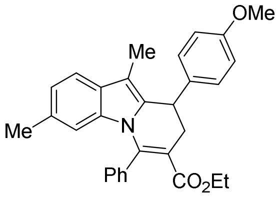

(±)-5m **Ethyl (±)-9-(4-methoxyphenyl)-3,10-dimethyl-6-phenyl-8,9-dihydropyrido[1,2-a]indole-7-carboxylate (5m).** Following *GP-C,* **(±)-5m** (21 mg, 0.047 mmol, 93%) was isolated by FC (petroleum ether/EtOAc, 25:1) on silica gel as a white solid, from cyclopropane **1e** (16 mg, 0.05 mmol) and indole **2d** (7 mg, 0.05 mmol) under diphenyl [(trifluoromethyl)sulfonyl]phosphoramidate (2 mg, 0.005 mmol) in toluene (0.25 mL) at 100 ºC for 45 min in an oil bath. R_f= 0.70 (petroleum ether/EtOAc, 9:1). **^{1}H NMR** (300 MHz, $CDCl_3$) δ 7.56-7.36 (m, 4H, C_{arom}-H), 7.32 (d, J = 7.9 Hz, 1H, H-1), 7.25-7.15 (m, 1H, C_{arom}-H), 7.10 (d, J = 8.3 Hz, 2H, C_{arom}-H), 6.89 (d, 1H, J = 8.0 Hz, H-2), 6.82 (d, J = 8.7 Hz, 2H, C_{arom}-H), 5.42 (s, 1H, H-4), 4.50 (app t, J = 4.7 Hz, 1H, H-9), 4.10-3.64 (m, 2H, OCH_2), 3.78 (s, 3H, OCH_3), 3.25 (dd, J = 15.4, 3.9 Hz, 1H, H-8a), 3.01 (dd, J = 15.4, 5.4 Hz, 1H, H-8b), 2.10 (s, 3H, CH_3), 2.07 (s, 3H, CH_3), 0.86 (t, J = 7.1 Hz, 3H, $OCH_2C\mathbf{H}_3$). **^{13}C NMR** (75 MHz, $CDCl_3$) δ 168.1 (COO), 158.4 (C_{arom}-O), 146.3 (C-6), 136.2 (C_{arom}-C), 135.9 (C_{arom}-C), 134.5 (C_{arom}-C), 133.8 (C_{arom}-C), 131.8 (C-10a), 130.2 (C_{arom}-H), 129.1 (3×C_{arom}-H), 128.5 (3×C_{arom}-H+ C-3), 122.5 (C-2), 118.0 (C-1), 114.1 (C-4), 114.0 (2× C_{arom}-H), 111.0 (C-10), 108.6 (C-7), 60.0 (OCH_2), 55.4 (OCH_3), 36.2 (C-9), 31.0 (C-8), 21.9 (C-3-**C**H_3), 13.8 (OCH_2**C**H_3), 8.7 (C-10-**C**H_3). **IR** (ATR): 2980 (C-H st), 1683 (C=O st), 1244 (C-O st as) cm^{-1}. **HRMS (ESI) m/z:** $[M+Na]^+$ Calcd for $C_{30}H_{29}NO_3Na$ 474.2045; Found 474.2047. **M.p.**: 111-114 ºC (petroleum ether/EtOAc).

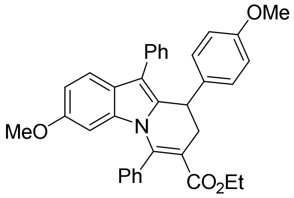

(±)-5n **Ethyl (±)-3-methoxy-9-(4-methoxyphenyl)-6,10-diphenyl-8,9-dihydropyrido[1,2-a]indole-7-carboxylate (5n).** Following *GP-C,* **(±)-5n** (21 mg, 0.040 mmol, 79%) was isolated by FC (petroleum ether/EtOAc, 25:1) on silica gel as a white solid, from cyclopropane **1e** (16 mg, 0.05 mmol) and indole **2o** (11 mg, 0.05 mmol) under diphenyl [(trifluoromethyl)sulfonyl]phosphoramidate (2 mg, 0.005 mmol) in toluene (0.25 mL) at 100 ºC for 45 min in an oil bath. R_f= 0.70 (petroleum ether/EtOAc, 9:1). **^{1}H NMR** (300 MHz, $CDCl_3$) δ 7.61-7.44 (m, 5H, C_{arom}-H), 7.44-7.28 (m, 6H, C_{arom}-H), 7.12 (d, J = 8.6 Hz, 2H, C_{arom}-H), 6.85 (d, J = 8.7 Hz, 2H, C_{arom}-H), 6.73 (dd, J = 8.7, 2.3 Hz, 1H, H-2), 5.40 (d, J = 2.3 Hz, 1H, H-4), 4.52 (dd, J = 5.3, 2.5 Hz, 1H, H-9), 4.13-3.74 (m, 2H, OCH_2), 3.80 (s, 3H, OCH_3), 3.35 (s, 1H, OCH_3), 3.27 (dd, J = 15.5, 2.5 Hz, 1H, H-8a), 2.96 (dd, J = 15.5, 5.3 Hz, 1H, H-8b), 0.86 (t, J = 7.1 Hz, 3H, $OCH_2C\mathbf{H}_3$). **^{13}C NMR** (75 MHz, $CDCl_3$) δ 167.7 (COO), 158.3 (C_{arom}-O), 156.2 (C-3), 145.7 (C-6), 136.7 (C_{arom}-C), 136.0 (C_{arom}-

C), 134.3 (C_{arom}-C), 133.9 (C_{arom}-C), 133.7 (C_{arom}-C), 129.3 (5×C_{arom}-H), 128.6 (6×C_{arom}-H), 126.8 (C_{arom}-H), 123.3 (C-10a), 119.9 (C-1), 117.0 (C-10), 114.0 (2× C_{arom}-H), 111.4 (C-2), 110.8 (C-7), 97.8 (C-4), 60.1 (OCH_2), 55.3 (OCH_3), 55.2 (OCH_3), 35.6 (C-9), 31.5 (C-8), 13.7 (OCH_2**CH_3**). **IR** (ATR): 2984 (C-H st), 1685 (C=O st), 1246 (C-O st as) cm^{-1}. **HRMS (ESI) m/z:** $[M+Na]^+$ Calcd for $C_{35}H_{31}NO_4Na$ 552.2151; Found 552.2148. **M.p.**: 152-155 ºC (petroleum ether/EtOAc).

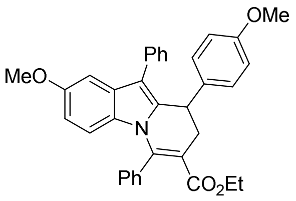

(±)-5o **Ethyl (±)-2-methoxy-9-(4-methoxyphenyl)-6,10-diphenyl-8,9-dihydropyrido[1,2-a]indole-7-carboxylate (5o).** Following *GP-C*, **(±)-5o** (19 mg, 0.035 mmol, 71%) was isolated by FC (petroleum ether/EtOAc, 25:1) on silica gel as a white solid, from cyclopropane **1e** (16 mg, 0.05 mmol) and indole **2p** (11 mg, 0.05 mmol) under diphenyl [(trifluoromethyl)sulfonyl]phosphoramidate (2 mg, 0.005 mmol) in toluene (0.25 mL) at 100 ºC for 45 min in an oil bath. R_f= 0.70 (petroleum ether/EtOAc, 9:1). **^{1}H NMR** (300 MHz, $CDCl_3$) δ 7.61-7.27 (m, 10H, C_{arom}-H), 7.15-7.05 (m, 3H, H-1 + C_{arom}-H), 6.83 (d, *J* = 8.7 Hz, 2H, C_{arom}-H), 6.46 (dd, *J* = 9.2, 2.6 Hz, 1H, H-4), 5.67 (d, *J* = 9.2 Hz, 1H, H-3), 4.51 (dd, *J* = 5.2, 2.5 Hz, 1H, H-9), 3.98-3.67 (m, 2H, OCH_2 3.79 (s, 3H, OCH_3), 3.76 (s, 3H, (OCH_3), 3.24 (dd, *J* = 15.5, 2.5 Hz, 1H, H-8a), 2.96 (dd, *J* = 15.5, 5.2 Hz, 1H, H8-b), 0.84 (t, *J* = 7.1 Hz, 3H, OCH_2C**H_3**). **^{13}C NMR** (75 MHz, $CDCl_3$) δ 167.8 (COO), 158.3 (C_{arom}-O), 155.5 (C-2), 145.7 (C-6), 136.1 (C_{arom}-C), 135.8 (C_{arom}-C), 134.1 (C_{arom}-C), 133.7 (C_{arom}-C), 130.8 (C_{arom}-C), 130.3 (C_{arom}-C), 129.4 (4×C_{arom}-H), 128.6 (7×C_{arom}-H), 126.9 (C_{arom}-H), 117.1 (C-10), 114.4 (C-4), 114.1 (2× C_{arom}-H), 111.6 (C-3), 110.2 (C-7), 102.1 (C-1), 60.1 (OCH_2), 55.9 (OCH_3), 55.3 (OCH_3), 35.7 (C-9), 31.4 (C-8), 13.7 (OCH_2**CH_3**). **IR** (ATR): 2951 (C-H st), 1684 (C=O st), 1248 (C-O st as) cm^{-1}. **HRMS (ESI) m/z:** $[M+Na]^+$ Calcd for $C_{35}H_{31}NO_4Na$ 552.2151; Found 552.2147. **M.p.**: 157-160 ºC (petroleum ether/EtOAc).

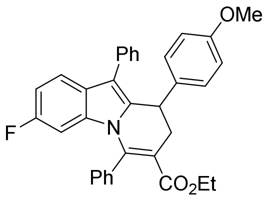

(±)-5p **Ethyl (±)-3-fluoro-9-(4-methoxyphenyl)-6,10-diphenyl-8,9-dihydropyrido[1,2-a]indole-7-carboxylate (5p).** Following *GP-C*, **(±)-5p** (23 mg, 0.044 mmol, 87%) was isolated by FC (petroleum ether/EtOAc, 25:1) on silica gel as a white solid, from cyclopropane **1e** (16 mg, 0.05 mmol) and indole **2q** (11 mg, 0.05 mmol) under diphenyl [(trifluoromethyl)sulfonyl]phosphoramidate (2 mg, 0.005 mmol) in toluene (0.25 mL) at 100 ºC for 45 min in an oil bath. R_f= 0.70 (petroleum ether/EtOAc, 9:1). **^{1}H NMR** (300 MHz, $CDCl_3$) δ 7.67-7.29 (m, 11H, C_{arom}-H), 7.11 (d, *J* = 8.7 Hz, 2H, C_{arom}-H), 6.85 (d, *J* = 8.7 Hz, 3H, H-2 + C_{arom}-H), 5.47 (dd, *J* = 11.4, 2.3 Hz, 1H, H-4), 4.53 (dd, *J* = 5.2, 2.5 Hz, 1H, H-9), 3.94-3.69 (m, 2H, OCH_2), 3.80 (s, 3H, OCH_3), 3.25 (dd, *J* = 15.6, 2.5 Hz, 1H, H-8a), 2.98 (dd, *J* = 15.6, 5.2 Hz, 1H, H-8b), 0.85 (t, *J* = 7.1 Hz, 3H, OCH_2C**H_3**). **^{13}C NMR** (75 MHz, $CDCl_3$) δ 167.6 (COO), 159.5 (d, $^1J_{C-F}$ = 238.2 Hz, C-3), 158.4 (C_{arom}-O), 145.2 (C-6), 135.9 (d, $^3J_{C-F}$ = 12.3 Hz, C-4a), 135.6 (d, $^4J_{C-F}$ = 4.0 Hz, C-10a), 135.2(C_{arom}-C), 134.0 (C_{arom}-C), 133.3 (C_{arom}-C), 129.7 (C_{arom}-H), 129.4 (2×C_{arom}-H), 128.5 (8×C_{arom}-H), 127.1 (C_{arom}-H), 125.8

(C-9a), 120.0 (d, $^3J_{C-F}$ = 9.9 Hz, C-1), 117.0 (C-10), 114.1 (2× C_{arom}-H), 111.5 (C-7), 109.9 (d, $^2J_{C-F}$ = 24.4 Hz, C-4), 100.85 (d, $^2J_{C-F}$ = 29.2 Hz, C-2), 60.2 (OCH_2), 55.3 (OCH_3), 35.7 (C-9), 31.5 (C-8), 13.7 (OCH_2**CH_3**). **IR** (ATR): 2980 (C-H st), 1688 (C=O st), 1244 (C-O st as), 1143 (C-F st) cm^{-1}. **HRMS (ESI) m/z:** $[M+Na]^+$ Calcd for $C_{34}H_{28}NO_3FNa$ 540.1951; Found 540.1955. **M.p.**: 178-181 ºC (petroleum ether/EtOAc).

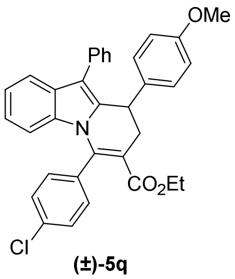

(±)-5q **Ethyl (±)-6-(4-chlorophenyl)-9-(4-methoxyphenyl)-10-phenyl-8,9-dihydropyrido[1,2-a]indole-7-carboxylate (5q).** Following *GP-C*, **(±)-5q** (24 mg, 0.045 mmol, 90%) was isolated by FC (petroleum ether/EtOAc, 25:1) on silica gel as a white solid, from cyclopropane **1f** (18 mg, 0.05 mmol) and indole **2l** (10 mg, 0.05 mmol) under diphenyl [(trifluoromethyl)sulfonyl]phosphoramidate (2 mg, 0.005 mmol) in toluene (0.25 mL) at 100 ºC for 45 min in an oil bath. R_f= 0.70 (petroleum ether/EtOAc, 9:1). **^{1}H NMR** (300 MHz, $CDCl_3$) δ 7.69 (d, *J* = 7.9 Hz, 1H, H-1), 7.54-7.28 (m, 9H, C_{arom}-H), 7.17-7.02 (m, 3H, H-2 + C_{arom}-H), 6.90 (ddd, *J* = 8.5, 7.1, 1.3 Hz, 1H, H-3), 6.84 (d, *J* = 8.7 Hz, 2H, C_{arom}-H), 5.95 (d, *J* = 8.5 Hz, 1H, H-4), 4.55 (dd, *J* = 5.3, 2.5 Hz, 1H, H-9), 4.14-3.66 (m, 2H, OCH_2), 3.80 (s, 3H, OCH_3), 3.28 (dd, *J* = 15.5, 2.5 Hz, 1H, H-8a), 2.95 (dd, *J* = 15.5, 5,3 Hz, 1H, H-8b), 0.93 (t, *J* = 7.1 Hz, 3H, OCH_2C**H_3**). **^{13}C NMR** (75 MHz, $CDCl_3$) δ 167.4 (COO), 158.4 (C_{arom}-O), 144.4 (C-6), 135.6 (C_{arom}-C), 135.5 (C_{arom}-C), 135.3 (C_{arom}-C), 134.1 (C-9a), 133.8 (C_{arom}-C), 133.4 (C-Cl), 129.5 (C_{arom}-C), 129.4 (3×C_{arom}-H), 128.6 (5×C_{arom}-H), 128.4 (2×C_{arom}-H), 127.0 (C_{arom}-H), 122.9 (C-3), 121.9 (C-2), 119.7 (C-1), 117.4 (C-10), 114.1 (2× C_{arom}-H), 113.5 (C-4), 111.6 (C-7), 60.3 (OCH_2), 55.3 (OCH_3), 35.6 (C-9), 31.4 (C-8), 13.8 (OCH_2**CH_3**). **IR** (ATR): 2980 (C-H st), 1686 (C=O st), 1242 (C-O st as), 1088 (C-Cl st) cm^{-1}. **HRMS (ESI) m/z:** $[M+H]^+$ Calcd for $C_{34}H_{29}NO_3Cl$ 534.1836; Found 534.1828. **M.p.**: 175-178 ºC (petroleum ether/EtOAc).

The reaction was carried out in a bigger scale: Following *GP-C*, **(±)-5q** (443 mg, 0.83 mmol, 83%) was isolated by FC (petroleum ether/EtOAc, 25:1) on silica gel as a white solid, from cyclopropane **1f** (360 mg, 1.00 mmol) and indole **2l** (200 mg, 1.00 mmol) under diphenyl [(trifluoromethyl)sulfonyl]phosphoramidate (40 mg, 0.10 mmol) in toluene (0.25 mL) at 100 ºC for 90 min in an oil bath.

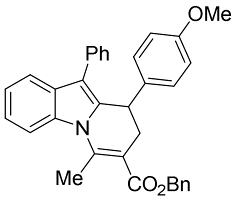

(±)-5r **Benzyl (±)-9-(4-methoxyphenyl)-6-methyl-10-phenyl-8,9-dihydropyrido[1,2-a]indole-7-carboxylate (5r).** Following *GP-C*, **(±)-5r** (21 mg, 0.042 mmol, 83%) was isolated by FC (petroleum ether/EtOAc, 25:1) on silica gel as a white solid, from cyclopropane **1g** (16 mg, 0.05 mmol) and indole **2l** (10 mg, 0.05 mmol) under diphenyl [(trifluoromethyl)sulfonyl]phosphoramidate (2 mg, 0.005 mmol) in toluene (0.25 mL) at 100 ºC for 45 min in an oil bath. R_f= 0.70 (petroleum ether/EtOAc,

9:1). **^{1}H NMR** (300 MHz, $CDCl_3$) δ 7.92-7.63 (m, 2H, H-1 + H-4), 7.48-7.09 (m, 12H, C_{arom}-H), 6.95 (d, *J* = 8.7 Hz, 2H, C_{arom}-H), 6.77 (d, *J* = 8.7 Hz, 2H, C_{arom}-H), 5.22 (d, *J* = 12.8 Hz, 1H, OC**H**$_a$H$_b$), 5.09 (d, *J* = 12.8 Hz, 1H, OCH$_a$**H**$_b$), 4.44 (dd, *J* = 5.1, 2.6 Hz, 1H, H-9), 3.79 (s, 3H, OCH_3), 3.33 (dd, *J* = 15.7, 2.6 Hz, 1H, H-8a), 3.01 (d, *J* = 2.1 Hz, 3H, C-6-CH_3), 2.75-2.63 (m, 1H, H-8b). **^{13}C NMR** (75 MHz, $CDCl_3$) δ 167.5 (COO), 158.3 (C_{arom}-O), 146.4 (C-6), 136.6 (C_{arom}-C), 136.0 (C_{arom}-C), 135.2 (C_{arom}-C), 133.9 (C_{arom}-C), 133.7 (C_{arom}-C), 129.6 (C_{arom}-C), 129.5 (2×C_{arom}-H), 128.6 (4×C_{arom}-H), 128.5 (2×C_{arom}-H), 128.0 (C_{arom}-H), 127.6 (2×C_{arom}-H), 126.8 (C_{arom}-H), 123.0 (C-3), 121.9 (C-2), 120.1 (C-1), 116.3 (C-10), 114.0 (2× C_{arom}-H), 113.7 (C-4), 110.5 (C-7), 66.0 (OCH_2), 55.3 (OCH_3), 35.6 (C-9), 30.5 (C-8), 19.7 (C-6-**C**H_3). **IR** (ATR): 2976 (C-H st), 1698 (C=O st), 1247 (C-O st as) cm^{-1}. **MS** (EI) m/z (%): 438.2 (27), 437.1 (100), 365.1 (27), 364.1 (M^+-$C_8H_7O_2$, 86), 363.1 (20), 349.2 (33), 337.1 (24), 329.1 (18), 301.1 (19). **HRMS (ESI) m/z:** $[M+Na]^+$ Calcd for $C_{34}H_{29}NO_3Na$ 522.2045; Found 522.2047. **M.p.**: 60-63 ºC (petroleum ether/EtOAc).

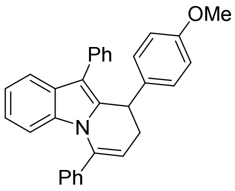

(±)-5s

(±)-9-(4-Methoxyphenyl)-6,10-diphenyl-8,9-dihydropyrido[1,2-a]indole (5s). Following *GP-C*, **(±)-5s** (15 mg, 0.036 mmol, 72%) was isolated by FC (petroleum ether/EtOAc, 99:1) on silica gel as a white solid, from cyclopropane **1h** (18 mg, 0.05 mmol) and indole **2l** (10 mg, 0.05 mmol) under diphenyl [(trifluoromethyl)sulfonyl]phosphoramidate (2 mg, 0.005 mmol) in toluene (0.25 mL) at 100 ºC for 45 min in an oil bath. R_f= 0.60 (petroleum ether/EtOAc, 19:1). **^{1}H NMR** (300 MHz, $CDCl_3$) δ 7.79 (d, *J* = 8.0 Hz, 1H, H-1), 7.57-7.28 (m, 10H, C_{arom}-H), 7.21-7.06 (m, 3H, C_{arom}-H), 6.95 (ddd, *J* = 8.3, 7.1, 1.2 Hz, 1H, H-3), 6.83 (d, *J* = 8.7 Hz, 2H, C_{arom}-H), 6.42 (d, *J* = 8.4 Hz, 1H, H-4), 5.38 (dd, *J* = 7.2, 3.0 Hz, 1H, H-7), 4.53 (app d, *J* = 5.2 Hz, 1H, H-9), 3.78 (s, 3H, OCH_3), 2.80 (ddd, *J* = 15.9, 5.5, 3.1 Hz, 1H, H-8a), 2.673 (ddd, *J* = 15.9, 7.3, 2.1 Hz, 1H, H-8b). **^{13}C NMR** (75 MHz, $CDCl_3$) δ 158.2 (C_{arom}-O), 139.1 (C-6), 136.6 (C_{arom}-C), 135.7 (C_{arom}-C), 135.3 (C_{arom}-C), 134.9 (C_{arom}-C), 129.5 (2×C_{arom}-H), 128.6 (8×C_{arom}-H), 128.2 (C_{arom}-C), 127.8 (2×C_{arom}-H), 126.4 (C_{arom}-C), 121.7 (C-3), 120.6 (C-2), 119.4 (C-1), 115.7 (C-10), 114.0 (2×C_{arom}-H), 113.3 (C-7), 111.5 (C-4), 55.3 (OCH_3), 36.2 (C-9), 30.5 (C-8). **IR** (ATR): 2955 (C-H st), 1650 (arC-C), 1608 (arC-C). cm^{-1}. **IR** (ATR): 2955 (C-H st), 1650 (arC-C), 1608 (arC-C) cm^{-1}. **HRMS (ESI) m/z:** $[M+H]^+$ Calcd for $C_{31}H_{26}NO$ 428.2014; Found 428.2004. **M.p.**: 239-242 ºC (petroleum ether/EtOAc).

2.3.3 Synthesis of dihydropyridoindoles 6

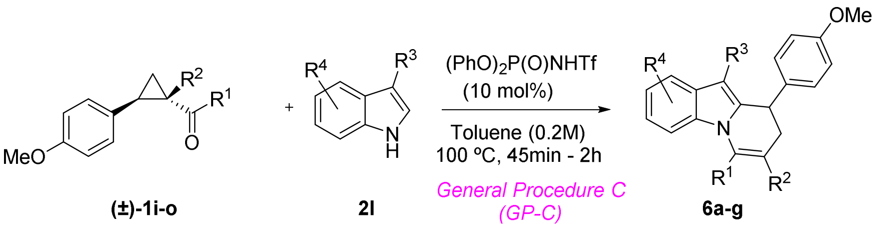

Scheme S5. ***General Procedure C for the synthesis of dihydropiridoindoles (±)-6a-g***

General Procedure C (GP-C) for the cyclocondensation: An oven-dried 5 mL screw-capped test tube containing a stirring bar was charged with cyclopropane **1i-o** (1eq.) and corresponding indole **2l** (1eq.) and dissolved in toluene (0.2 M) under Ar. After adding the diphenyl [(trifluoromethyl)sulfonyl]phosphoramidate catalyst (10 mol%) the mixture was stirred at 100 ºC in an oil bath. When the reaction was judged complete (monitored by TLC), it was warmed to room temperature, quenched by addition of aq. $NaHCO_3$ (sat) (3 mL) and diluted with EtOAc (3 mL). The phases were separated and the aqueous phase was extracted with EtOAc (3×3 mL). The combined organic layers were dried over Na_2SO_4, filtered and evaporated. The residue was purified by column chromatography on silica gel to afford the corresponding pure compounds **6a-g**.

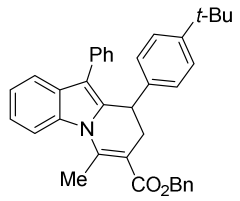

(±)-6a **Benzyl (±)-9-(4-(*tert*-butyl)phenyl)-6-methyl-10-phenyl-8,9-dihydropyrido[1,2-a]indole-7-carboxylate (6a).** Following *GP-C*, **(±)-6a** (20 mg, 0.038 mmol, 76%) was isolated by FC (petroleum ether/EtOAc, 25:1) on silica gel as a white solid, from cyclopropane **1i** (17 mg, 0.05 mmol) and indole **2l** (10 mg, 0.05 mmol) under diphenyl [(trifluoromethyl)sulfonyl]phosphoramidate (2mg, 0.005 mmol) in toluene (0.25 mL) at 100 ºC for 45 min in an oil bath. R_f= 0.70 (petroleum ether/EtOAc, 9:1). **^{1}H NMR** (300 MHz, $CDCl_3$) δ 7.87-7.61 (m, 2H, H-1 + H-4), 7.41-7.13 (m, 14H, C_{arom}-H), 6.96 (d, *J* = 8.3 Hz, 2H, C_{arom}-H), 5.22 (d, *J* = 12.8 Hz, 1H, OC**H**$_a$H$_b$), 5.11 (d, *J* = 12.8 Hz, 1H, OCH$_a$**H**$_b$), 4.47 (dd, *J* = 5.2, 2.9 Hz, 1H, H-9), 3.36 (dd, *J* = 15.8, 2.9 Hz, 1H, H-8a), 3.03 (d, *J* = 2.0 Hz, 3H, C-6-CH_3), 2.74 (dd, *J* = 15.8, 5.2 Hz, 1H, H-8b), 1.30 (s, 9H,C(CH_3)$_3$). **^{13}C NMR** (75 MHz, $CDCl_3$) δ 167.6 (COO), 149.2 (**C**$_{arom}$-O), 146.5 (C-6), 138.4 (C_{arom}-C), 136.6 (C_{arom}-C), 136.0 (C_{arom}-C), 135.2 (C_{arom}-C), 133.7 (C_{arom}-C), 129.7 (C_{arom}-C), 129.5 (2×C_{arom}-H), 128.6 (4×C_{arom}-H), 128.0 (C_{arom}-H), 127.7 (2×C_{arom}-H), 127.1 (2×C_{arom}-H), 126.8 (C_{arom}-H), 125.4 (2×C_{arom}-H), 123.0 (C-3), 121.8 (C-2), 120.1 (C-1), 116.5 (C-10), 113.7 (C-4), 110.5 (C-7), 66.0 (OCH_2), 35.9 (C-9), 34.5 (**C**(CH_3)$_3$), 31.5 (C(**C**H$_3$)$_3$), 30.2 (C-8), 19.7 (C-6-**C**H$_3$). **IR** (ATR): 2961 (C-H st), 1698 (C=O st), 1243 (C-O st as) cm^{-1}. **HRMS (ESI) m/z:** $[M+Na]^+$ Calcd for $C_{37}H_{35}NO_2Na$ 548.2565; Found 548.2569. **M.p.**: 82-85 ºC (petroleum ether/EtOAc).

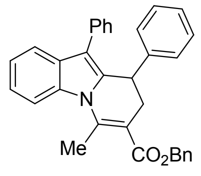

(±)-6b **Benzyl (±)-6-methyl-9,10-diphenyl-8,9-dihydropyrido[1,2-a]indole-7-carboxylate (6b).** Following *GP-C*, **(±)-6b** (17 mg, 0.036 mmol, 72%) was isolated by FC (petroleum ether/EtOAc, 25:1) on silica gel as a white solid, from cyclopropane **1j** (15 mg, 0.05 mmol) and indole **2l** (10 mg, 0.05 mmol) under diphenyl [(trifluoromethyl)sulfonyl]phosphoramidate (2 mg, 0.005 mmol) in toluene (0.25 mL) at 100 ºC for 45 min in an oil bath. R_f= 0.70 (petroleum ether/EtOAc, 9:1). **^{1}H NMR** (300 MHz, $CDCl_3$) δ 8.00-7.57 (m, 2H, H-1 + H-4), 7.46-7.13 (m, 15H, C_{arom}-H), 7.04 (dd, *J* = 7.5, 2.0 Hz, 2H, C_{arom}-H), 5.20 (d, *J* = 12.8 Hz, 1H, OC**H**$_a$H$_b$), 5.08 (d, *J* = 12.8 Hz, 1H, OCH$_a$**H**$_b$), 4.49 (dd, *J* = 5.1, 2.5 Hz, 1H, H-9), 3.36 (dd, *J* = 15.7, 2.6 Hz, 1H, H-8a), 3.01 (d, *J* = 2.2 Hz, 3H, C-6-CH_3), 2.82-2.65 (m, 1H, H-8b). **^{13}C NMR** (75 MHz, $CDCl_3$) δ 167.4 (COO), 146.5 (C-6), 141.8 (C_{arom}-C), 136.6 (C_{arom}-C),

135.6 (C_{arom}-C), 135.3 (C_{arom}-C), 133.6 (C_{arom}-C), 129.6 (C-10a), 129.5 (2×C_{arom}-H), 128.6 (6×C_{arom}-H), 128.0 (C_{arom}-H), 127.6 (2×C_{arom}-H), 127.5 (2×C_{arom}-H), 126.9 (C_{arom}-H), 126.6 (C_{arom}-H), 123.1 (C-3), 121.9 (C-2), 120.1 (C-1), 116.5 (C-10), 113.8 (C-4), 110.4 (C-7), 66.0 (OCH_2), 36.4 (C-9), 30.5 (C-8), 19.7 (C-6-**C**H_3). **IR** (ATR): 2956 (C-H st), 1698 (C=O st), 1248 (C-O st as) cm^{-1}. **HRMS (ESI) m/z:** $[M+Na]^+$ Calcd for $C_{33}H_{27}NO_2Na$ 492.1939; Found 492.1944. **M.p.**: 67-70 ºC (petroleum ether/EtOAc).

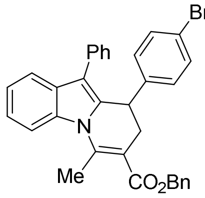

(±)-6c

Benzyl (±)-9-(4-bromophenyl)-6-methyl-10-phenyl-8,9-dihydropyrido[1,2-a]indole-7-carboxylate (6c). Following *GP-C*, **(±)-6c** (15 mg, 0.031 mmol, 62%) was isolated by FC (petroleum ether/EtOAc, 25:1) on silica gel as a white solid, from cyclopropane **1k** (19 mg, 0.05 mmol) and indole **2l** (10 mg, 0.05 mmol) under diphenyl [(trifluoromethyl)sulfonyl]phosphoramidate (2 mg, 0.005 mmol) in toluene (0.25 mL) at 100 ºC for 45 min in an oil bath. R_f= 0.70 (petroleum ether/EtOAc, 9:1). **^{1}H NMR** (300 MHz, $CDCl_3$) δ 7.78-7.53 (m, 2H, H-1 + H-4), 7.40-6.98 (m, 14H, C_{arom}-H), 6.81 (d, *J* = 8.4 Hz, 2H, C_{arom}-H), 5.13 (d, *J* = 12.7 Hz, 1H, OC**H**$_a$CH_b), 4.98 (d, *J* = 12.7 Hz, 1H, OCH_aC**H**$_b$), 4.33 (dd, *J* = 5.2, 2.6 Hz, 1H, H-9), 3.21 (dd, *J* = 15.7, 2.6 Hz, 1H, H-8a), 2.90 (d, *J* = 2.1 Hz, 3H, CH_3), 2.63 (ddd, *J* = 15.7, 5.2, 2.2 Hz, 1H, H-8b). **^{13}C NMR** (75 MHz, $CDCl_3$) δ 167.3 (COO), 146.4 (C-6), 140.9 (C_{arom}-C), 136.5 (C_{arom}-C), 135.2 (C_{arom}-C), 134.8 (C_{arom}-C), 133.4 (C_{arom}-C), 131.7 (2×C_{arom}-H), 129.5 (C_{arom}-C), 129.4 (2×C_{arom}-H), 129.3 (2×C_{arom}-H), 128.7 (4×C_{arom}-H), 128.1 (C_{arom}-H), 127.6 (2×C_{arom}-H), 127.0 (C_{arom}-H), 123.3 (C-3), 122.0 (C-2), 120.6 (C-Br), 120.2 (C-1), 116.8 (C-10), 113.8 (C-4), 110.1 (C-7), 66.1 (OCH_2), 36.0 (C-9), 30.4 (C-8), 19.6 (C-6-**C**H_3). **IR** (ATR): 2942 (C-H st), 1698 (C=O st), 1265 (C-O st as) cm^{-1}. **HRMS (ESI) m/z:** $[M+H]^+$ Calcd for $C_{33}H_{27}BrNO_2$ 548.1225; Found 548.1219. **M.p.**: 152-155 ºC (petroleum ether/EtOAc).

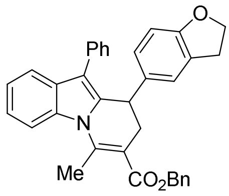

(±)-6d

Benzyl (±)-9-(2,3-dihydrobenzofuran-5-yl)-6-methyl-10-phenyl-8,9-dihydropyrido[1,2-a]indole-7-carboxylate (6d). Following *GP-C*, **(±)-6d** (24 mg, 0.046 mmol, 92%) was isolated by FC (petroleum ether/EtOAc, 25:1) on silica gel as a white solid, from cyclopropane **1l** (17 mg, 0.05 mmol) and indole **2l** (10 mg, 0.05 mmol) under diphenyl [(trifluoromethyl)sulfonyl]phosphoramidate (2 mg, 0.005 mmol) in toluene (0.25 mL) at 100 ºC for 45 min in an oil bath. R_f= 0.70 (petroleum ether/EtOAc, 9:1). **^{1}H NMR** (300 MHz, $CDCl_3$) δ 7.80-7.75 (m, 2H, H-1 + H-4), 7.52-7.17 (m, 12H, C_{arom}-H), 6.96-6.70 (m, 2H, C_{arom}-H), 6.66 (d, *J* = 8.2 Hz, 1H, C_{arom}-H), 5.24 (d, *J* = 12.8 Hz, 1H, OC**H**$_a$$H_b$), 5.10 (d, *J* = 12.8 Hz, 1H, OCH_a**H**$_b$), 4.54 (t, *J* = 8.9 Hz, 2H, OC**H**$_2$CH_2), 4.42 (dd, *J* = 5.1, 2.7 Hz, 1H, H-9), 3.31 (dd, *J* = 15.7, 2.7 Hz, 1H, H-8a), 3.17-2.87 (m, 2H, OCH_2C**H**$_2$), 3.02 (d, *J* = 2.1 Hz, 3H, CH_3), 2.79-2.56 (m, 1H, H-8b). **^{13}C NMR** (75 MHz, $CDCl_3$) δ 167.5 (COO), 158.8 (**C**$_{arom}$-OCH_2), 146.4 (C-6), 136.6 (C_{arom}-C), 136.1 (C_{arom}-C), 135.2 (C_{arom}-C), 133.8 (C_{arom}-C), 133.7 (C_{arom}-C), 129.6 (C_{arom}-C), 129.4 (2×C_{arom}-H), 128.6 (4×C_{arom}-H), 128.0 (C_{arom}-H), 127.6 (2×C_{arom}-H), 127.2 (C_{arom}-C + C_{arom}-H), 126.8 (C_{arom}-H), 123.9 (C_{arom}-H), 123.0 (C-3), 121.9 (C-2), 120.1

(C-1), 116.2 (C-10), 113.7 (C-4), 110.6 (C-7), 109.1 (C_{arom}-H), 71.3 (O**C**H_2CH_2), 66.0 (O**C**H_2C_{arom}), 35.9 (C-9), 30.7 (C-8), 29.9 (OCH_2**C**H_2), 19.7 (C-6-**C**H_3). **IR** (ATR): 2957 (C-H st), 1698 (C=O st), 1242 (C-O st as) cm^{-1}. **HRMS (ESI) m/z:** $[M+Na]^+$ Calcd for $C_{35}H_{29}NO_3Na$ 534.2045; Found 534.2054. **M.p.**: 85-88 ºC (petroleum ether/EtOAc).

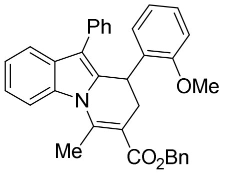

(±)-6e

Benzyl (±)-9-(2-methoxyphenyl)-6-methyl-10-phenyl-8,9-dihydropyrido[1,2-a]indole-7-carboxylate (6e). Following *GP-C*, **(±)-6e** (20 mg, 0.040 mmol, 80%) was isolated by FC (petroleum ether/EtOAc, 25:1) on silica gel as a white solid, from cyclopropane **1m** (16 mg, 0.05 mmol) and indole **2l** (10 mg, 0.05 mmol) under diphenyl [(trifluoromethyl)sulfonyl] phosphoramidate (2 mg, 0.005 mmol) in toluene (0.25 mL) at 100 ºC for 45 min in an oil bath. R_f= 0.70 (petroleum ether/EtOAc, 9:1). **^{1}H NMR** (300 MHz, $CDCl_3$) δ 7.79 (m, 2H, H-1 + H-4), 7.49-7.05 (m, 13H, C_{arom}-H), 6.88 (dd, *J* = 8.2, 1.1 Hz, 1H, C_{arom}-H), 6.75 (app td, *J* = 7.5, 1.1 Hz, 1H, C_{arom}-H), 6.56 (dd, *J* = 7.6, 1.7 Hz, 1H, C_{arom}-H), 5.17 (d, *J* = 12.9 Hz, 1H, OC**H**$_a$H$_b$), 5.03 (d, *J* = 12.9 Hz, 1H, OCH$_a$**H**$_b$), 4.76 (dd, *J* = 5.5, 2.5 Hz, 1H, H-9), 3.76 (s, 3H, OCH_3), 3.41 (dd, *J* = 15.8, 2.5 Hz, 1H, H-8a), 3.03 (d, *J* = 2.1 Hz, 3H, C-6-CH_3), 2.58 (dd, *J* = 15.8, 5.5 Hz, 1H, H-8b). **^{13}C NMR** (75 MHz, $CDCl_3$) δ 167.7 (COO), 156.5 (**C**$_{arom}$-OCH_3), 145.8 (C-6), 136.7 (C_{arom}-C), 136.4 (C_{arom}-C), 135.3 (C_{arom}-C), 133.8 (C_{arom}-C), 130.0 (C_{arom}-C), 129.7 (C-10a), 129.4 (2×C_{arom}-H), 128.8 (C_{arom}-H), 128.5 (2×C_{arom}-H), 128.4 (2×C_{arom}-H), 127.9 (2×C_{arom}-H), 127.5 (2×C_{arom}-H), 126.6 (C_{arom}-H), 122.9 (C-3), 121.8 (C-2), 120.4 (C_{arom}-H), 120.1 (C-1), 115.9 (C-10), 113.7 (C_{arom}-H), 111.3 (C-4), 110.7 (C-7), 65.8 (OCH_2), 55.4 (OCH_3), 31.6 (C-9), 28.3 (C-8), 19.6 (C-6-**C**H_3). **IR** (ATR): 2934 (C-H st), 1698 (C=O st), 1241 (C-O st as) cm^{-1}. **HRMS (ESI) m/z:** $[M+Na]^+$ Calcd for $C_{34}H_{29}NO_3Na$ 522.2045; Found 522.2046. **M.p.**: 74-77 ºC (petroleum ether/EtOAc).

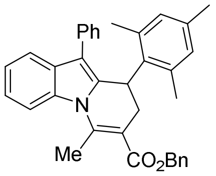

(±)-6f

Benzyl (±)-9-mesityl-6-methyl-10-phenyl-8,9-dihydropyrido[1,2-a]indole-7-carboxylate (6f). Following *GP-C*, **(±)-6f** (16 mg, 0.031 mmol, 42%) was isolated by FC (petroleum ether/EtOAc, 25:1) on silica gel as a white solid, from cyclopropane **1n** (17 mg, 0.05 mmol) and indole **2l** (10 mg, 0.05 mmol) under diphenyl [(trifluoromethyl)sulfonyl]phosphoramidate (2 mg, 0.005 mmol) in toluene (0.25 mL) at 100 ºC for 45 min in an oil bath. R_f= 0.70 (petroleum ether/EtOAc, 9:1). **^{1}H NMR** (300 MHz, $CDCl_3$) δ 7.74 (d, *J* = 8.2 Hz, 1H, H-1), 7.52 (d, *J* = 7.4 Hz, 1H, H-4), 7.41-7.28 (m, 5H, C_{arom}-H), 7.25-7.12 (m, 2H, C_{arom}-H), 7.08-6.98 (m, 5H, C_{arom}-H), 6.65 (s, 1H, C_{arom}-H), 6.33 (s, 1H, C_{arom}-H), 5.33-5.07 (m, 2H, OCH_2), 4.71 (dd, *J* = 11.3, 6.7 Hz, 1H, H-9), 3.06 (s, 3H, C-6-CH_3), 3.00-2.72 (m, 2H, H-8), 2.34 (s, 3H, CH_3), 2.07 (s, 3H, CH_3), 1.82 (s, 3H, CH_3). **^{13}C NMR** (75 MHz, $CDCl_3$) δ 167.7 (COO), 146.6 (C-6), 136.5 (C_{arom}-C), 136.0 (2×C_{arom}-C), 135.8 (C_{arom}-C), 135.0 (C_{arom}-C), 133.8 (C_{arom}-C), 133.4 (C_{arom}-C), 130.6 (2×C_{arom}-C), 129.2 (3×C_{arom}-H), 129.0 (C_{arom}-H), 128.7 (3×C_{arom}-H), 128.2 (C_{arom}-H), 128.1 (2×C_{arom}-H), 127.4 (C_{arom}-H), 126.1 (C_{arom}-H), 122.7 (C-3), 121.7 (C-2), 119.8 (C-1), 117.3 (C-10), 114.3 (C-4), 111.0 (C-7), 66.3 (OCH_2), 35.5 (C-9), 28.1 (C-8), 20.6 (CH_3), 19.8

($3{\times}CH_3$). **IR** (ATR): 2920 (C-H st), 1699 (C=O st), 1243 (C-O st as) cm^{-1}. **HRMS (ESI) m/z:** $[M+Na]^+$ Calcd for $C_{36}H_{33}NO_2Na$ 534.2409; Found 534.2408. **M.p.**: 197-200 ºC (petroleum ether/EtOAc).

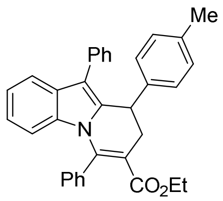

(±)-6g

Ethyl (±)-6,10-diphenyl-9-(*p*-tolyl)-8,9-dihydropyrido[1,2-a]indole-7-carboxylate (6g). Following *GP-C,* **(±)-6g** (19 mg, 0.039 mmol, 78%) was isolated by FC (petroleum ether/EtOAc, 25:1) on silica gel as a white solid, from cyclopropane **1o** (15 mg, 0.05 mmol) and indole **2l** (10 mg, 0.05 mmol) under diphenyl [(trifluoromethyl)sulfonyl]phosphoramidate (2 mg, 0.005 mmol) in toluene (0.25 mL) at 100 ºC for 45 min in an oil bath. R_f= 0.70 (petroleum ether/EtOAc, 9:1). **^{1}H NMR** (300 MHz, $CDCl_3$) δ 7.69 (d, *J* = 7.9 Hz, 1H, H-1), 7.56-7.24 (m, 10H, C_{arom}-H), 7.17-7.01 (m, 5H, C_{arom}-H), 6.84 (ddd, *J* = 8.4, 7.1, 1.3 Hz, 1H, H-3), 5.83 (d, *J* = 8.5 Hz, 1H, H-4), 4.57 (dd, *J* = 5.3, 2.4 Hz, 1H, H-9) 4.07-3.67 (m, 2H, OCH_2), 3.28 (dd, *J* = 15.5, 2.4 Hz, 1H, H-8a), 2.99 (dd, *J* = 15.5, 5,3 Hz, 1H, H-8b), 2.33 (s, 3H, C_{arom}-CH_3), 0.86 (t, *J* = 7.1 Hz, 3H, $OCH_2C\mathbf{H}_3$). **^{13}C NMR** (75 MHz, $CDCl_3$) δ 167.7 (COO), 145.6 (C-6), 138.9 (C_{arom}-C), 136.2 (C_{arom}-C), 136.0 (C_{arom}-C), 135.8 (C_{arom}-C), 135.3 (C_{arom}-C), 133.6 (C_{arom}-C), 129.4 ($6{\times}C_{arom}$-H + C-10a), 128.6 ($5{\times}C_{arom}$-H), 127.3 ($2{\times}C_{arom}$-H), 126.9 (C_{arom}-H), 122.7 (C-3), 121.7 (C-2), 119.6 (C-1), 117.3 (C-10), 113.7 (C-4), 111.0 (C-7), 60.1 (OCH_2), 36.0 (C-9), 31.4 (C-8), 21.2 (C_{arom}-$\mathbf{C}H_3$), 13.7 ($OCH_2\mathbf{C}H_3$). **IR** (ATR): 2984 (C-H st), 1692 (C=O st), 1237 (C-O st as) cm^{-1}. **MS** (EI) m/z (%): 410.2 (M^+-C_3H_5O, 3), 207.0 (21). **HRMS (ESI) m/z:** $[M+H]^+$ Calcd for $C_{34}H_{30}NO_2$ 484.2277; Found 484.2279. **M.p.**: 182-185 ºC (petroleum ether/EtOAc).

3 X-Ray Analysis

3.1 Compound 3a

Suitable crystal for X-ray analysis were obtained by liquid/liguid diffusion at room temperature using CH_2Cl_2 as the first solvent and hexane as the solvent for perform the diffusion (aprox. 7 days). Compound **3a** is completely soluble in the first one but unsoluble in the second one.

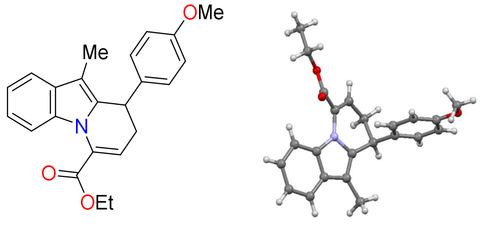

3a

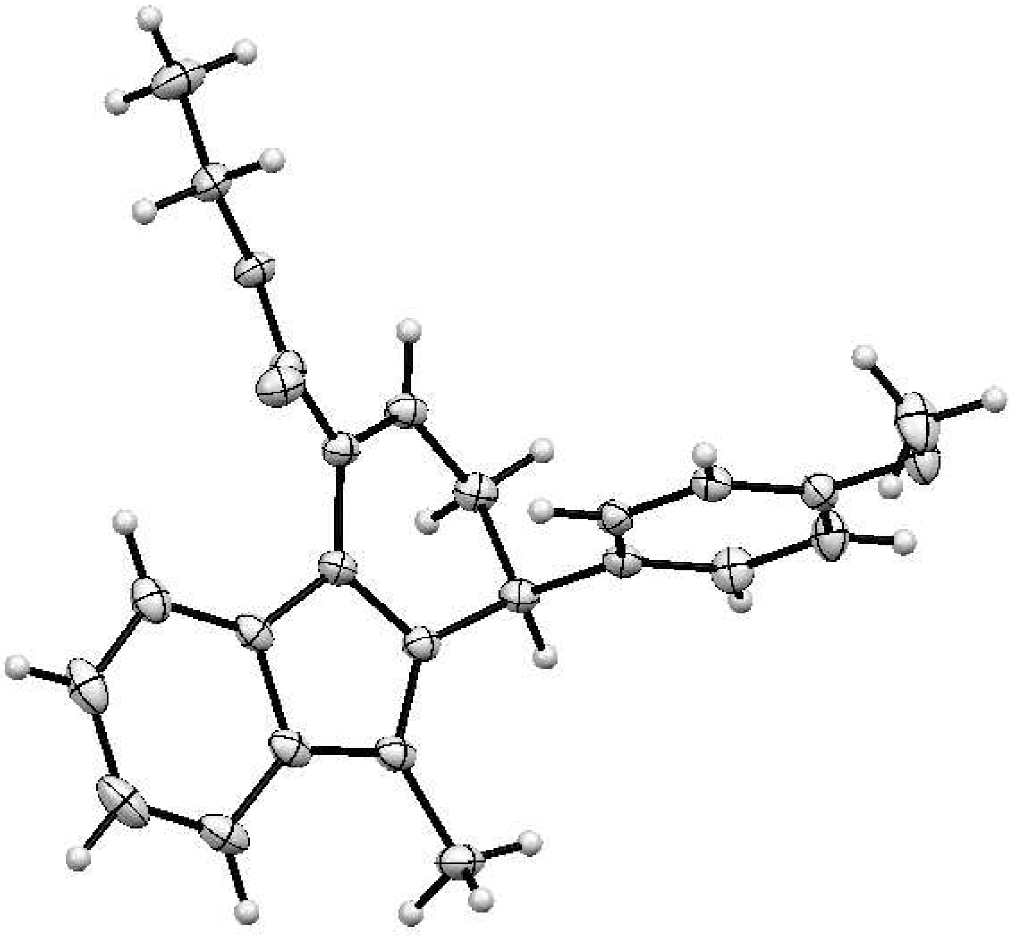

ORTEP for **3a**

Figure S2. Crystal structure determination of 3a and ORTEP plot at 50% probability

Crystal Data for $C_{23}H_{23}NO_3$ (*M* =361.42 g/mol): monoclinic, space group P21/n (no. 14), a = 8.87178(12) Å, b = 11.07423(15) Å, c = 19.5230(3) Å, β = 92.1583(13)°, V = 1916.73(5) Å3, Z = 4, T = 293(2) K, μ(CuKα) = 0.661 mm^{-1}, Dcalc = 1.252 g/cm^3, 23713 reflections measured (9.06° ≤ 2Θ ≤ 152.9°), 3972 unique (Rint = 0.0329, Rsigma = 0.0191) which were used in all calculations. The final R1 was 0.0541 (>2sigma(I)) and wR2 was 0.1614 (all data).

Instrumentation: Intensity data were collected on an Agilent Technologies Super-Nova diffractometer, wich was equipped with monochromated Cu kα radiation (λ= 1.54184 Å) and Atlas CCD detector. Measurement was carried out at 150.01(10) K with the help of an Oxford Cryostream 700 PLUS temperature device.

Table S1. Crystal data and structure refinement for 3a

Identification code	a2015395_AL170
Empirical formula	$C_{23}H_{23}NO_3$
Formula weight	361.42
Temperature/K	293(2)
Crystal system	monoclinic
Space group	$P2_1/n$
a/Å	8.87178(12)
b/Å	11.07423(15)
c/Å	19.5230(3)
α/°	90
β/°	92.1583(13)
γ/°	90
Volume/$Å^3$	1916.73(5)
Z	4
$\rho_{calc}g/cm^3$	1.252
μ/mm^{-1}	0.661
F(000)	768.0
Crystal size/mm^3	0.29 × 0.1786 × 0.1548
Radiation	CuKα (λ = 1.54184)
2Θ range for data collection/°	9.06 to 152.9
Index ranges	-11 ≤ h ≤ 10, -13 ≤ k ≤ 13, -24 ≤ l ≤ 24
Reflections collected	23713
Independent reflections	3972 [R_{int} = 0.0329, R_{sigma} = 0.0451]
Data/restraints/parameters	3972/0/248
Goodness-of-fit on F^2	1.040
Final R indexes [I>=2σ (I)]	R_1 = 0.0330, wR_2 = 0.0817
Final R indexes [all data]	R_1 = 0.0370, wR_2 = 0.0846

Table S2. Fractional Atomic Coordinates (×10^4) and Equivalent Isotropic Displacement Parameters (Å^2×10^3) for 3a. U_{eq} is defined as 1/3 of of the trace of the orthogonalised U_{IJ} tensor.

Atom	*x*	*y*	*z*	U(eq)
O1	1787.3(9)	6795.9(7)	994.2(4)	25.72(18)
O2	350.5(8)	8212.1(7)	462.4(4)	22.58(17)
O3	-1751.1(9)	6778.8(7)	4423.1(4)	27.96(19)
N1	3081.9(9)	8700.8(8)	1837.1(4)	19.97(19)
C1	1694.7(11)	8783.1(9)	1464.9(5)	19.8(2)
C2	653.2(11)	9576.9(9)	1654.5(5)	21.2(2)
C3	937.9(12)	10410.3(9)	2250.5(5)	22.3(2)
C4	1939.1(11)	9789.1(9)	2816.1(5)	19.8(2)
C5	3271.4(11)	9214.7(9)	2494.6(5)	19.5(2)
C6	4730.0(12)	9076.9(9)	2726.2(5)	21.4(2)
C7	5510.2(12)	8465.6(9)	2194.7(6)	22.2(2)
C8	7009.9(12)	8090.2(10)	2147.4(7)	28.2(2)
C9	7438.3(13)	7533.6(10)	1552.8(7)	33.1(3)
C10	6405.6(14)	7369.5(10)	1000.4(7)	32.4(3)
C11	4915.2(13)	7735.6(10)	1028.6(6)	26.0(2)
C12	4472.1(12)	8258.0(9)	1640.5(6)	21.1(2)
C13	1321.1(11)	7818.2(9)	954.6(5)	20.5(2)
C14	-170.4(12)	7287.3(10)	-22.6(5)	24.6(2)
C15	-1228.5(15)	7889.3(12)	-535.9(6)	34.5(3)
C16	1018.7(11)	8915.0(9)	3234.3(5)	19.3(2)
C17	826.7(11)	7701.2(9)	3072.3(5)	19.3(2)
C18	-68.9(11)	6941.9(9)	3460.1(5)	20.2(2)
C19	-805.9(11)	7417.4(9)	4016.3(5)	21.5(2)
C20	-617.0(13)	8629.3(10)	4190.0(6)	26.9(2)
C21	286.7(13)	9363.3(10)	3803.6(6)	25.2(2)
C22	-1869.9(16)	5519.8(11)	4310.6(7)	37.8(3)
C23	5443.9(13)	9476.4(11)	3393.7(6)	28.2(2)

Table S3. Anisotropic Displacement Parameters (Å^2×10^3) for 22a. The Anisotropic displacement factor exponent takes the form: $-2\pi^2[h^2a^{*2}U_{11}+2hka^*b^*U_{12}+\ldots]$.

**Atom**	**U_{11}**	**U_{22}**	**U_{33}**	**U_{23}**	**U_{13}**	**U_{12}**
O1	28.9(4)	20.6(4)	27.2(4)	1.5(3)	-5.1(3)	-0.4(3)
O2	24.5(4)	24.1(4)	18.8(4)	0.7(3)	-4.3(3)	-0.8(3)
O3	30.3(4)	23.2(4)	31.2(4)	-0.8(3)	11.3(3)	-3.1(3)
N1	16.7(4)	21.9(4)	21.2(4)	-0.1(3)	-0.7(3)	-0.2(3)
C1	19.3(5)	20.3(5)	19.6(5)	4.3(4)	-2.2(4)	-2.9(4)
C2	19.4(5)	20.2(5)	23.8(5)	4.5(4)	-3.4(4)	-1.3(4)
C3	21.7(5)	18.4(5)	26.7(5)	1.7(4)	-1.6(4)	0.7(4)
C4	18.7(5)	19.0(5)	21.6(5)	-1.0(4)	-1.4(4)	-1.3(4)
C5	19.5(5)	18.8(5)	20.0(5)	2.4(4)	-0.9(4)	-2.8(4)
C6	18.8(5)	20.4(5)	24.9(5)	5.0(4)	-1.8(4)	-3.5(4)
C7	18.6(5)	18.9(5)	29.2(5)	5.9(4)	0.4(4)	-2.4(4)
C8	18.0(5)	23.3(5)	43.2(7)	7.6(5)	-0.6(4)	-2.0(4)
C9	19.2(5)	23.5(6)	57.1(8)	3.9(5)	10.1(5)	0.9(4)
C10	29.6(6)	22.8(5)	45.9(7)	-3.1(5)	15.0(5)	-3.1(4)
C11	26.2(6)	21.0(5)	31.1(6)	-0.3(4)	6.6(4)	-4.1(4)
C12	18.2(5)	18.2(5)	27.2(5)	3.2(4)	3.0(4)	-1.9(4)
C13	19.0(5)	23.0(5)	19.4(5)	3.8(4)	-0.7(4)	-2.5(4)
C14	24.9(5)	28.2(5)	20.3(5)	-3.2(4)	-2.1(4)	-1.7(4)
C15	40.4(7)	35.2(6)	26.7(6)	2.9(5)	-12.2(5)	-4.1(5)
C16	16.8(5)	19.4(5)	21.4(5)	0.4(4)	-1.8(4)	1.1(4)
C17	16.2(5)	21.4(5)	20.1(5)	-2.3(4)	-0.2(4)	2.7(4)
C18	18.8(5)	17.7(5)	23.8(5)	-2.4(4)	-1.1(4)	0.7(4)
C19	19.1(5)	22.4(5)	23.1(5)	1.8(4)	1.7(4)	-0.3(4)
C20	30.8(6)	23.8(5)	26.8(5)	-3.9(4)	8.5(4)	2.5(4)
C21	29.8(6)	17.9(5)	28.2(6)	-3.1(4)	3.1(4)	0.1(4)
C22	44.8(7)	26.6(6)	43.0(7)	-4.5(5)	16.2(6)	-12.9(5)
C23	23.6(5)	31.7(6)	28.8(6)	2.7(5)	-7.0(4)	-4.6(4)

4 NMR spectra

4.1 Acyl-substituted donor-acceptor cyclopropanes (1a-d)

1a

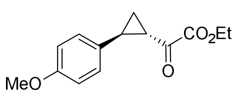

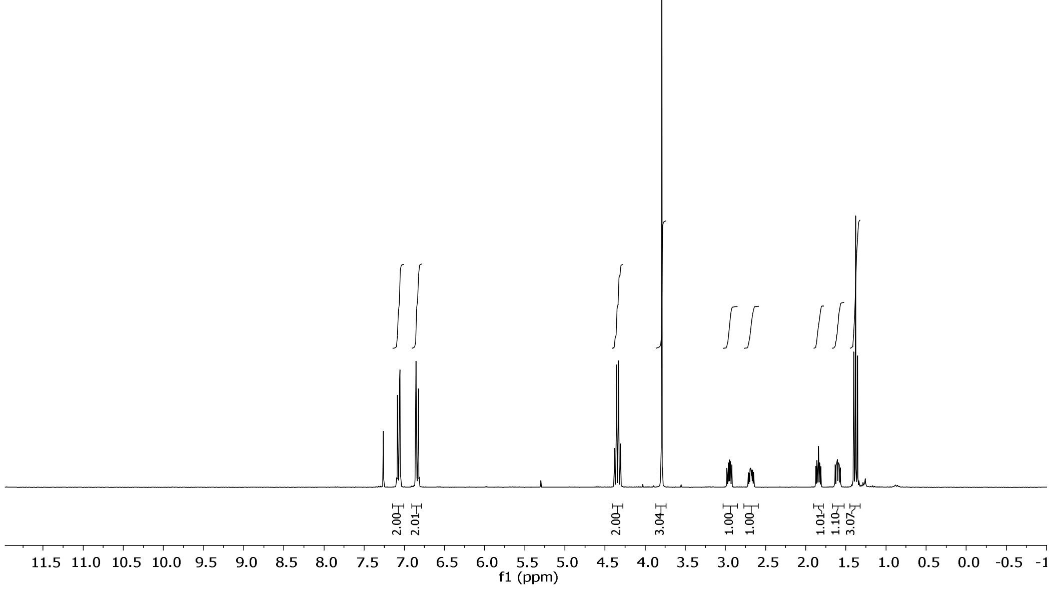

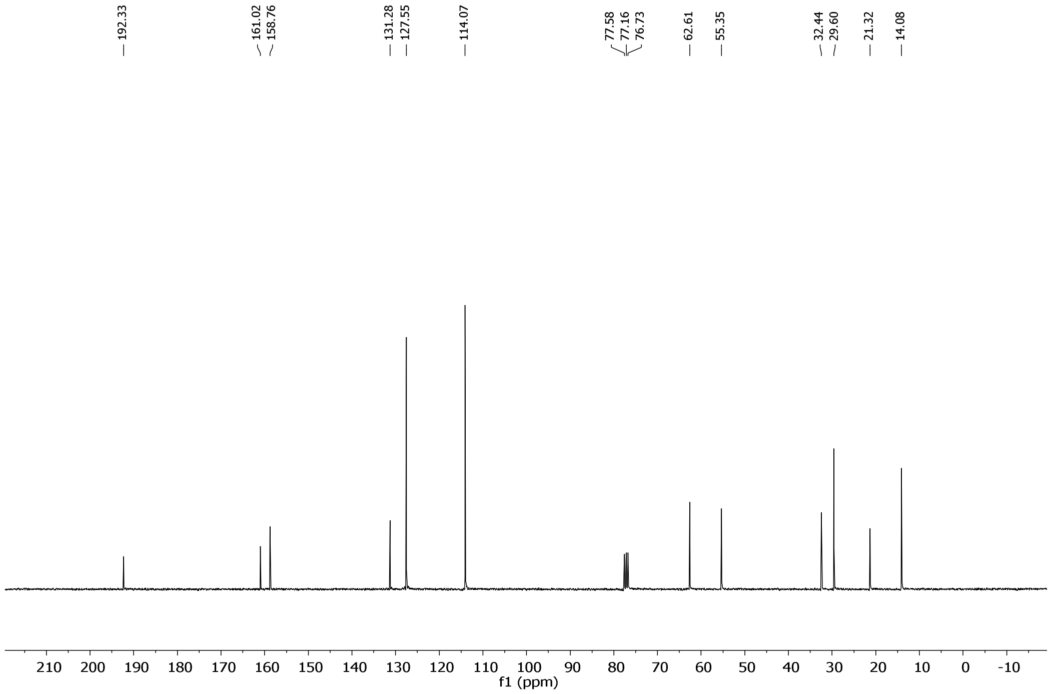

Figure S3. **^{1}H-NMR (300 MHz, $CDCl_3$) and ^{13}C-NMR (75 MHz, $CDCl_3$) spectra of compound 1a**

1b

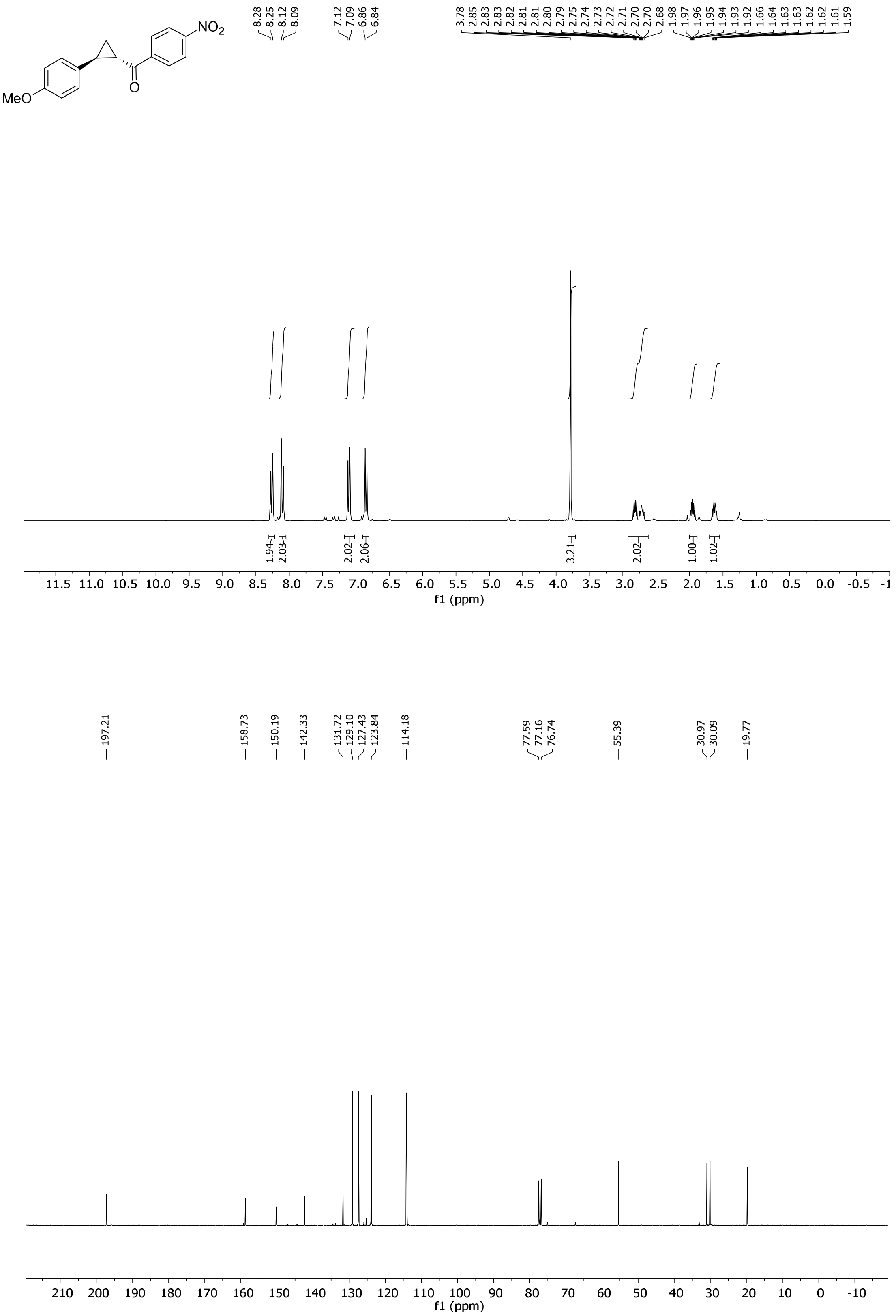

Figure S4. **^{1}H-NMR (300 MHz, $CDCl_3$) and ^{13}C-NMR (75 MHz, $CDCl_3$) spectra of compound 1b**

1c

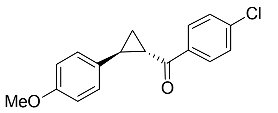

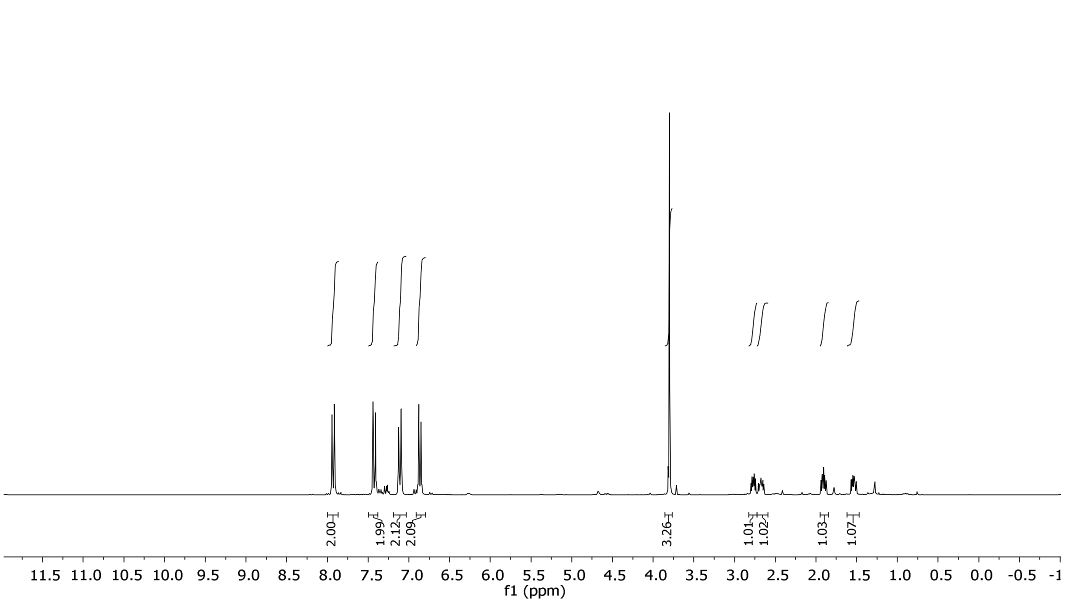

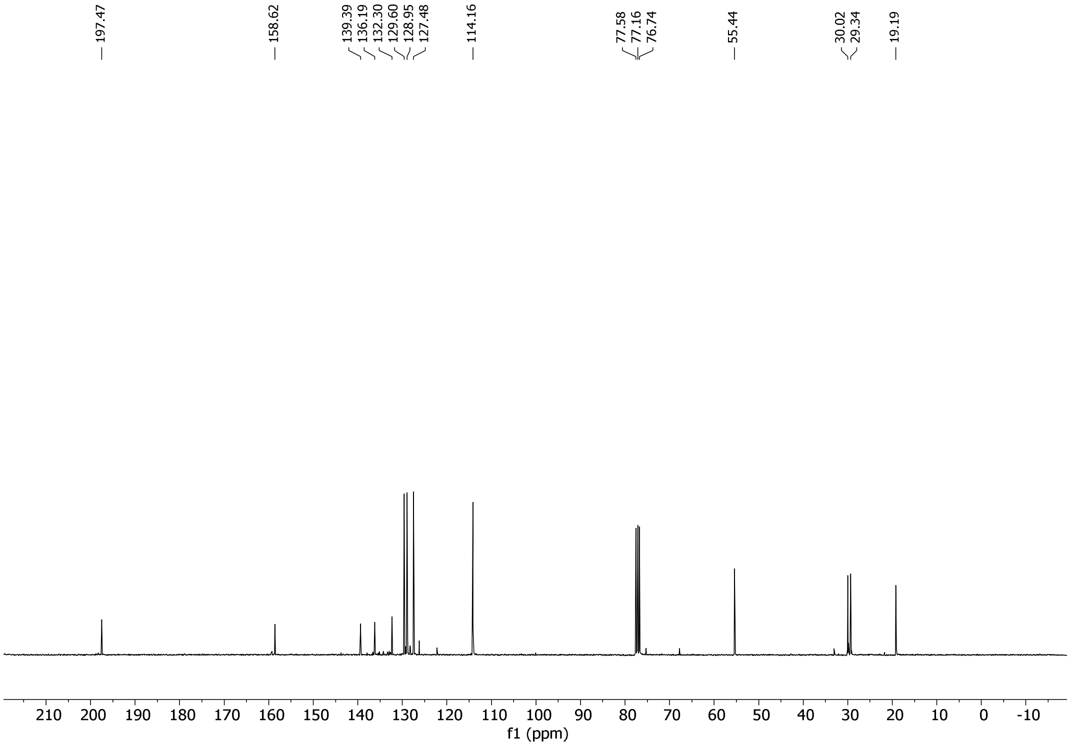

Figure S5. ^{1}H-NMR (300 MHz, $CDCl_3$) and ^{13}C-NMR (75 MHz, $CDCl_3$) spectra of compound 1c

1d

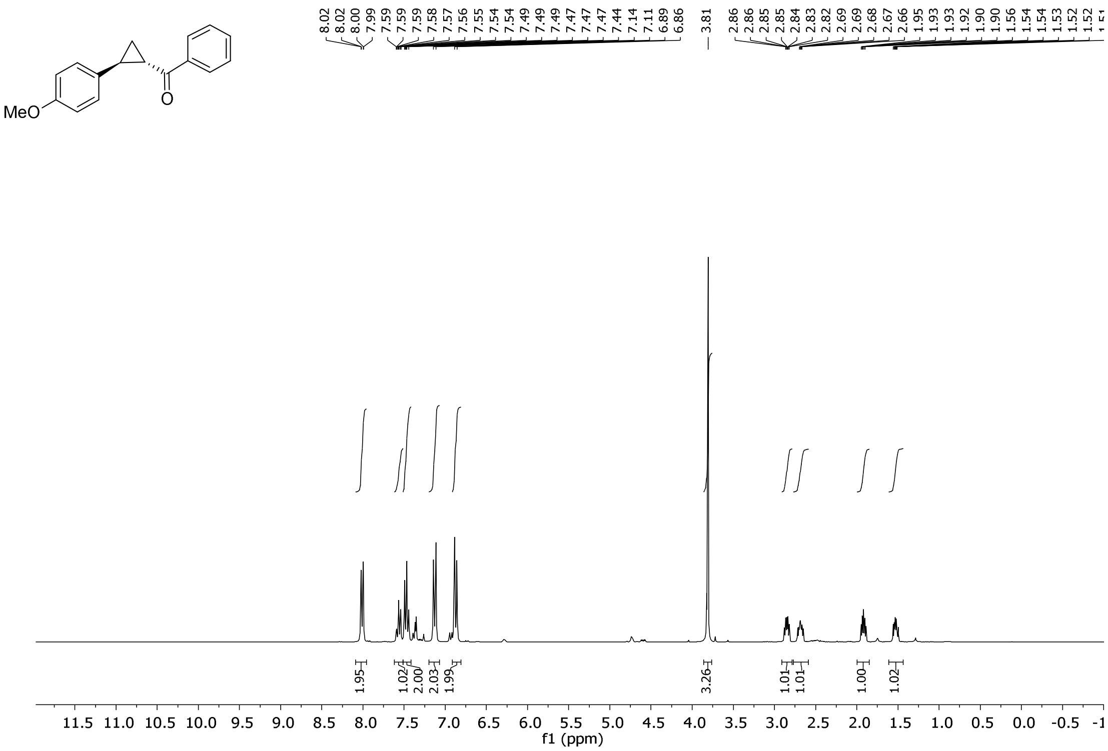

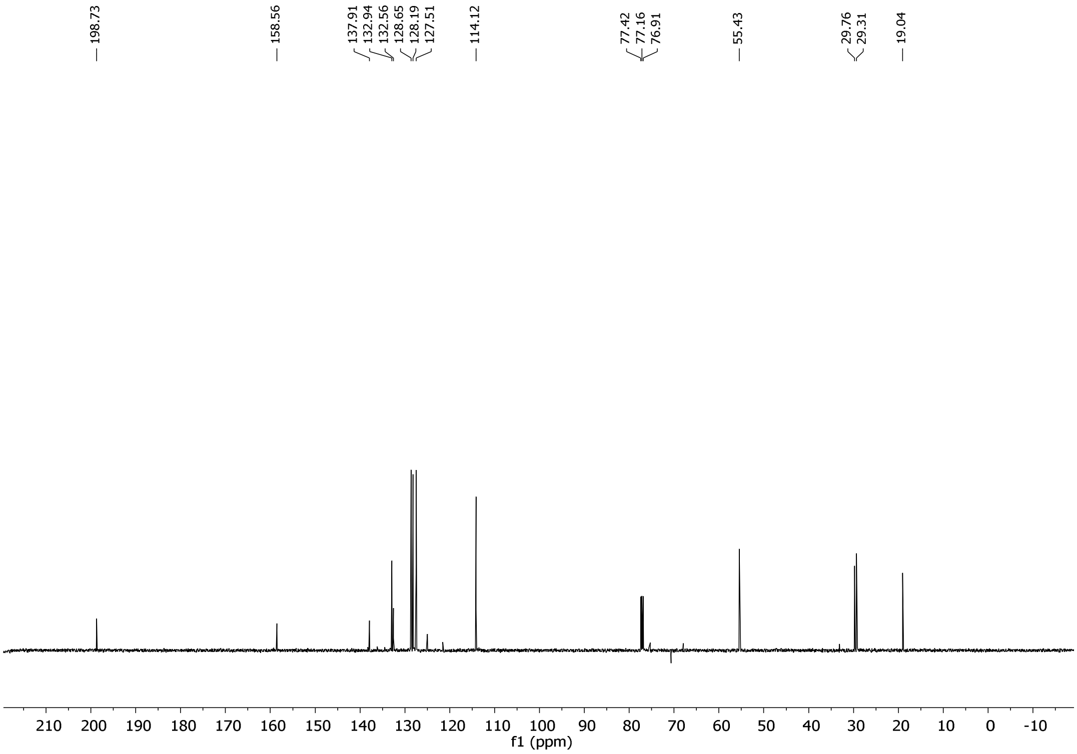

Figure S6. ^{1}H-NMR (300 MHz, $CDCl_3$) and ^{13}C-NMR (75 MHz, $CDCl_3$) spectra of compound 1d

4.2 Cyclopropanes derived from β-ketoesters (1e-o)

1e

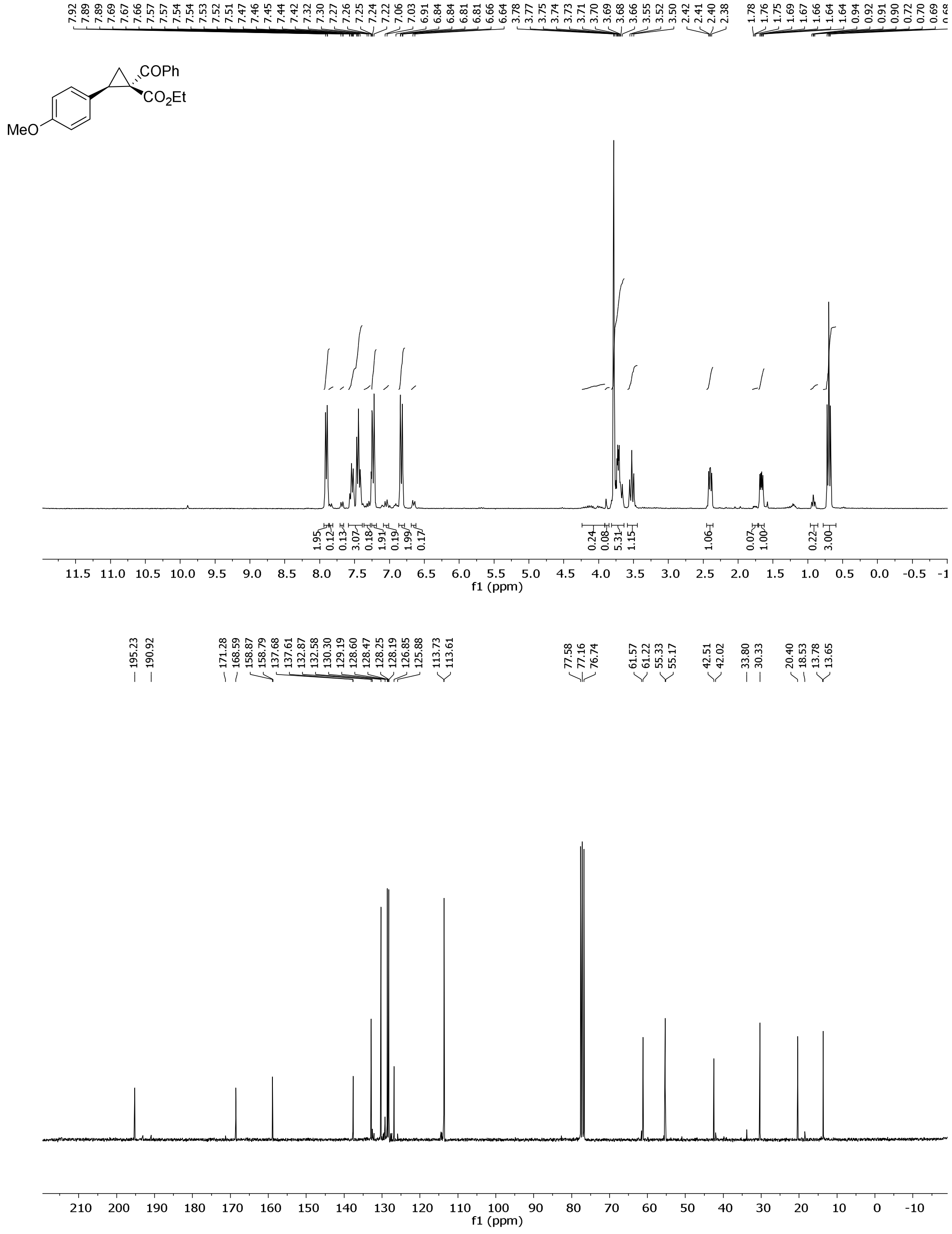

Figure S7. ^{1}H-NMR (300 MHz, $CDCl_3$) and ^{13}C-NMR (75 MHz, $CDCl_3$) spectra of compound 1e

1f

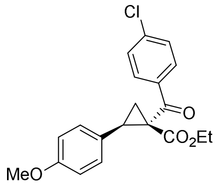

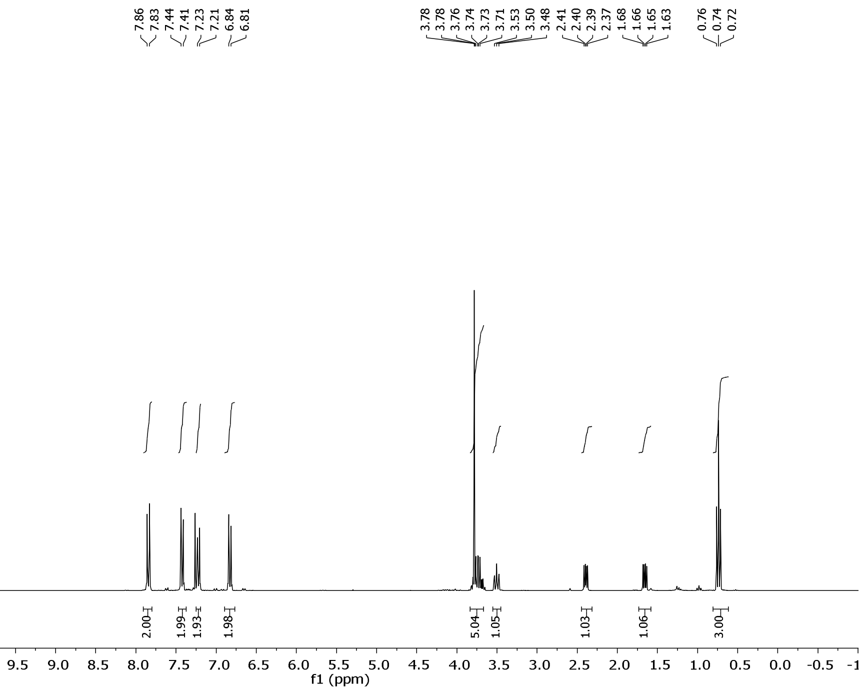

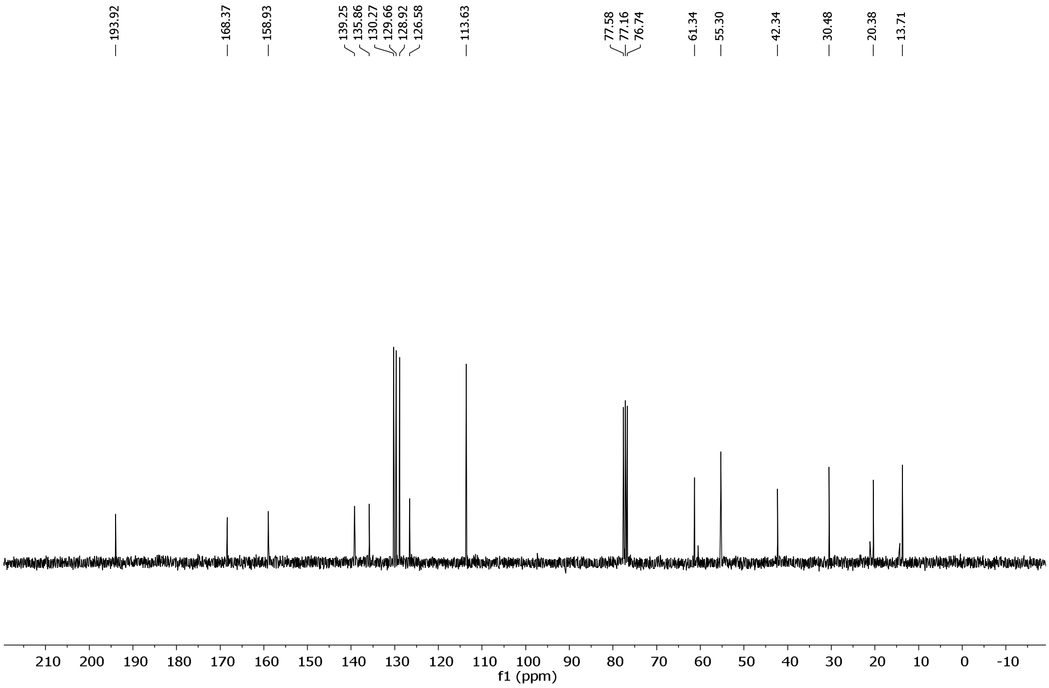

Figure S8. ^{1}H-NMR (300 MHz, $CDCl_3$) and ^{13}C-NMR (75 MHz, $CDCl_3$) spectra of compound 1f

1g

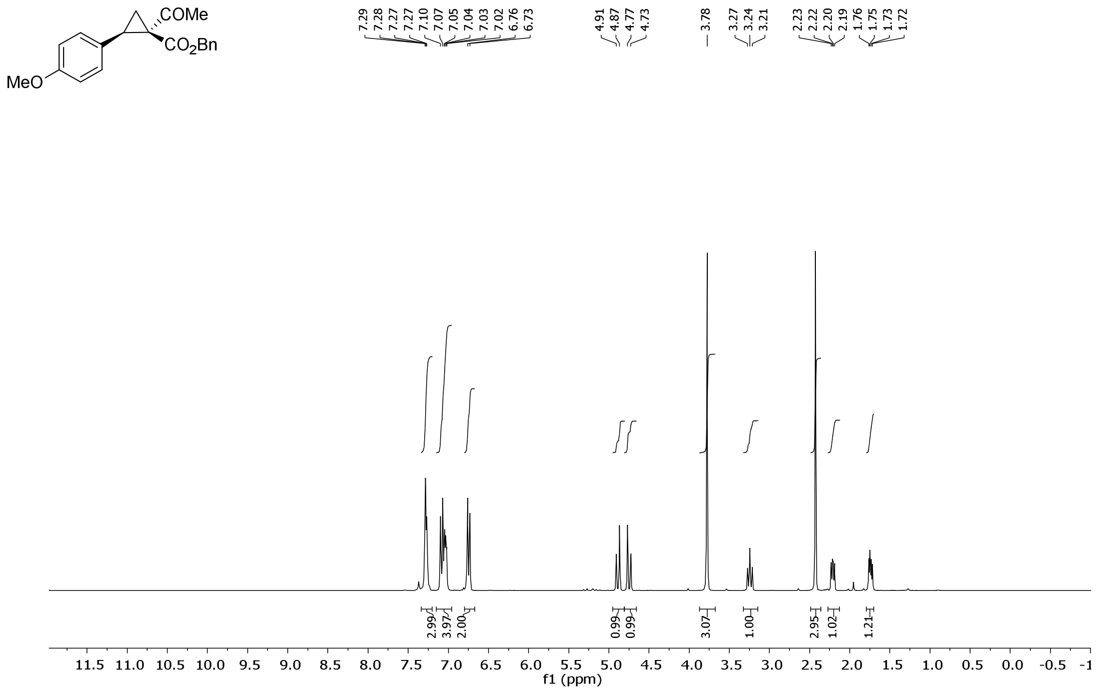

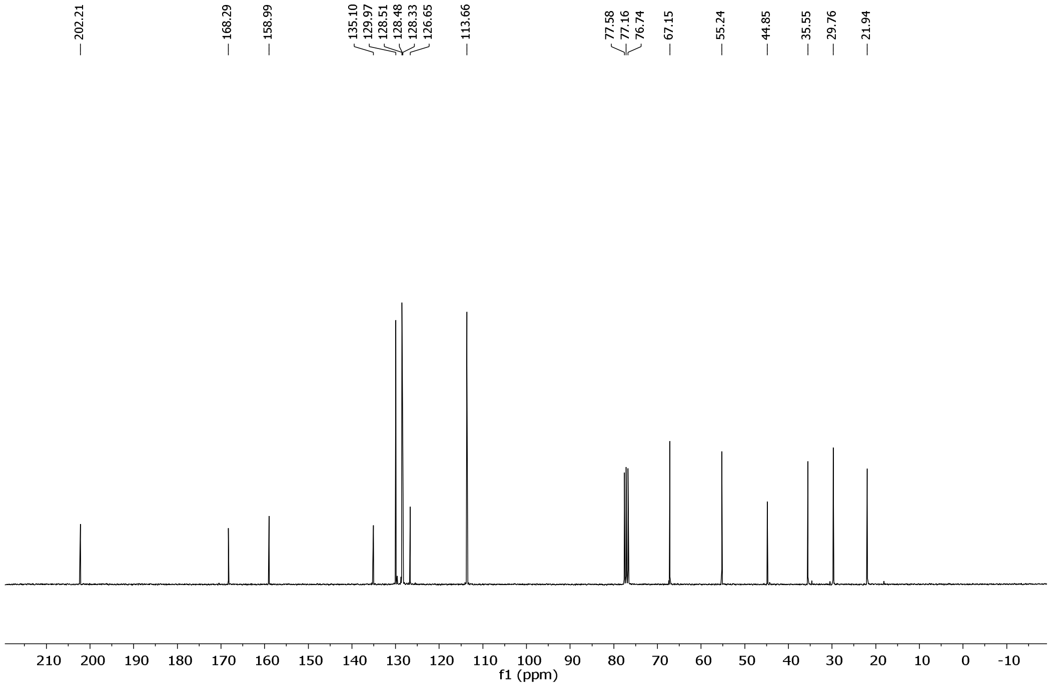

Figure S9. ^{1}H-NMR (300 MHz, $CDCl_3$) and ^{13}C-NMR (75 MHz, $CDCl_3$) spectra of compound 1g

1h

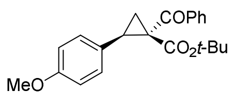

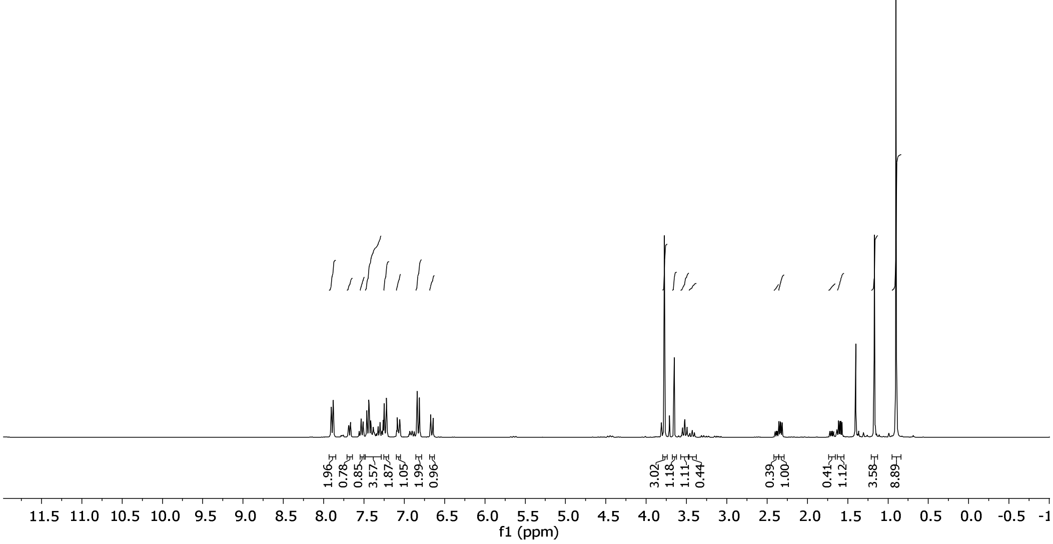

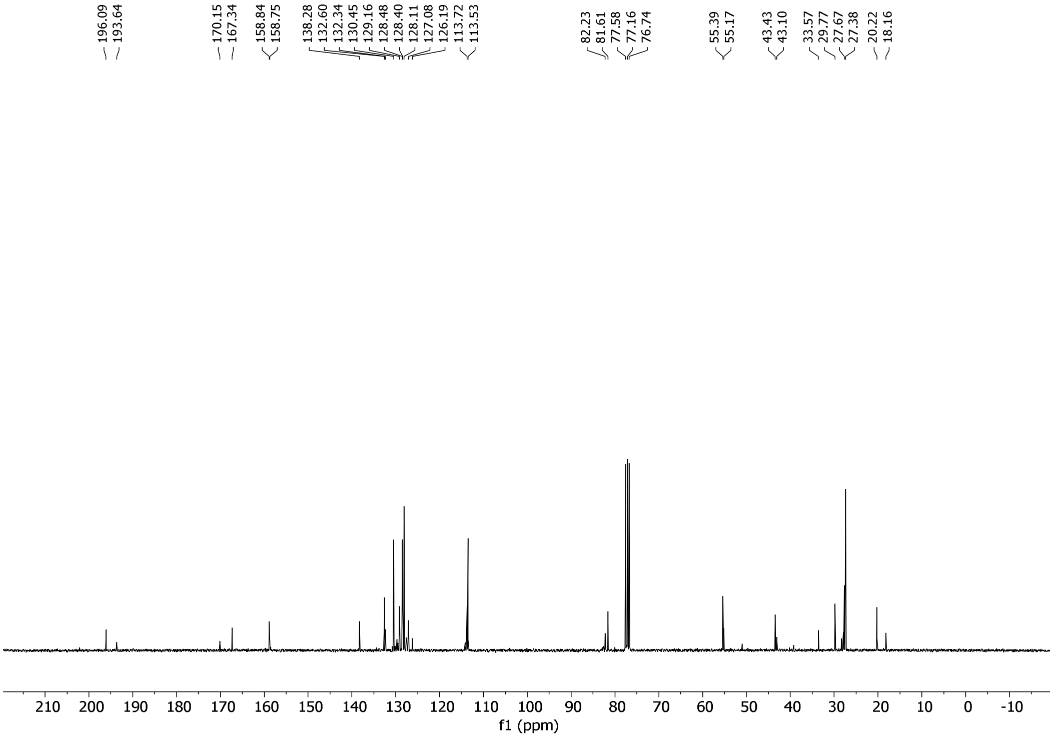

Figure S10. **^{1}H-NMR (300 MHz, $CDCl_3$) and ^{13}C-NMR (75 MHz, $CDCl_3$) spectra of compound 1h**

1i

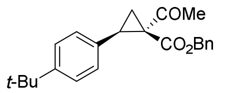

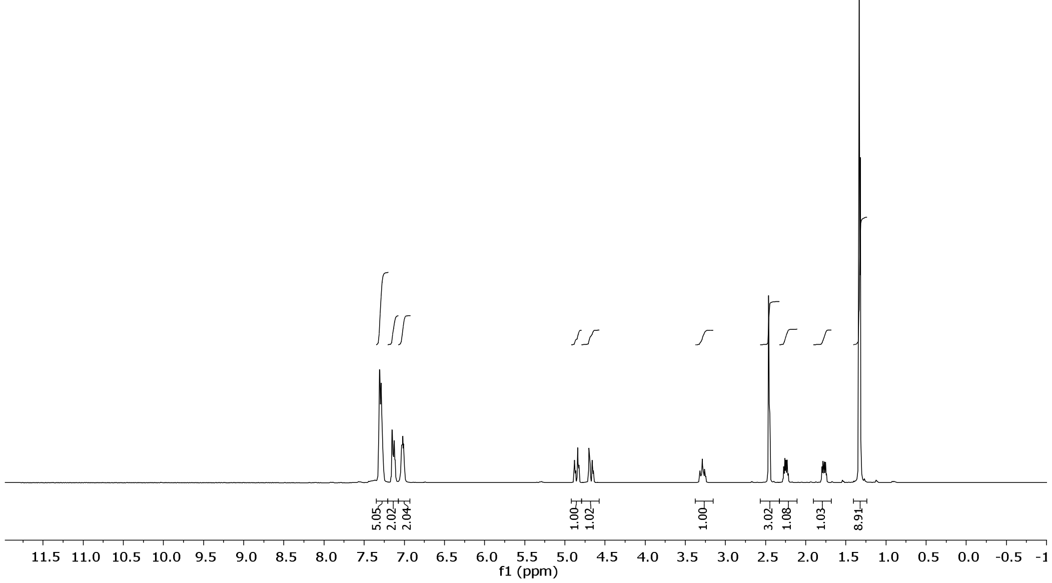

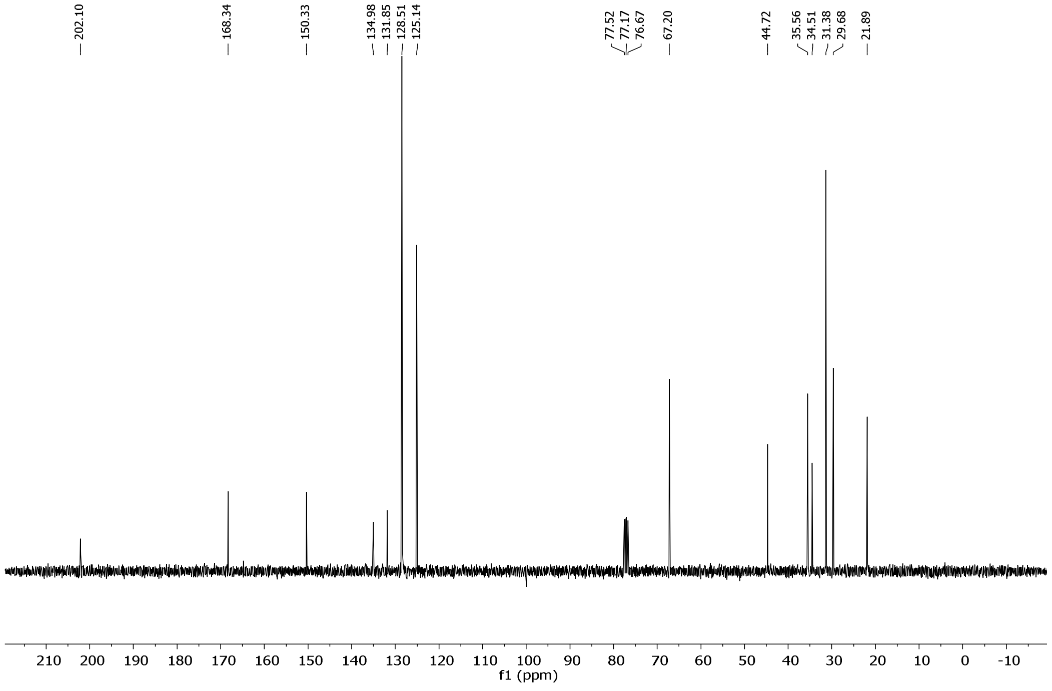

Figure S11. ^{1}H-NMR (300 MHz, $CDCl_3$) and ^{13}C-NMR (75 MHz, $CDCl_3$) spectra of compound 1i

1j

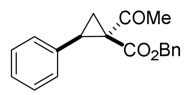

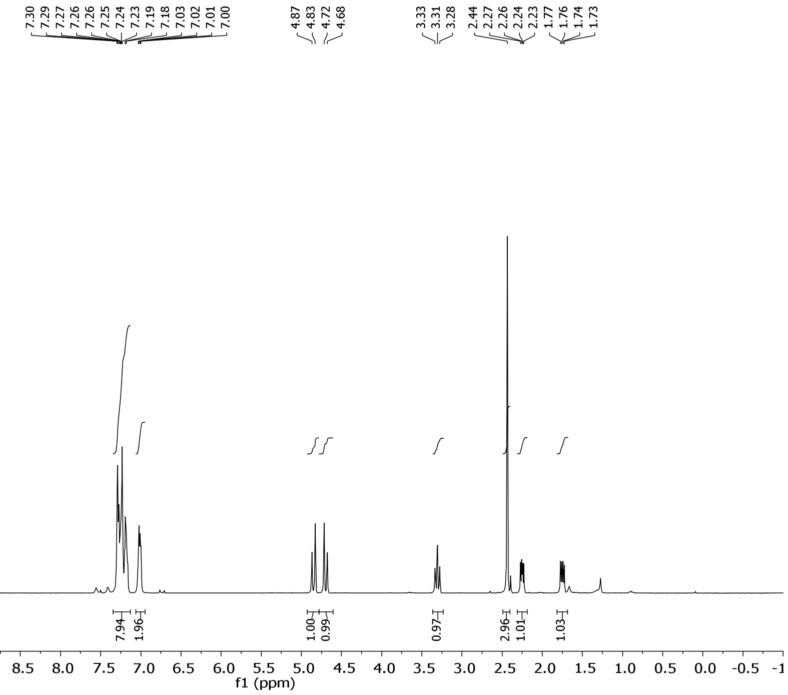

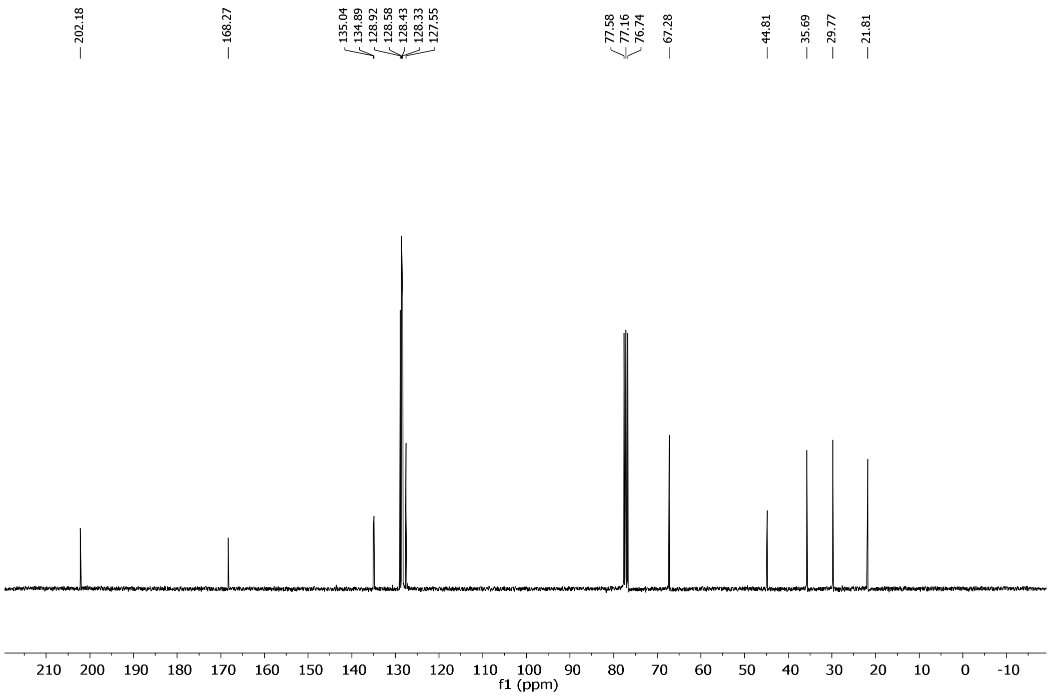

Figure S12. **^{1}H-NMR (300 MHz, $CDCl_3$) and ^{13}C-NMR (75 MHz, $CDCl_3$) spectra of compound 1j**

1k

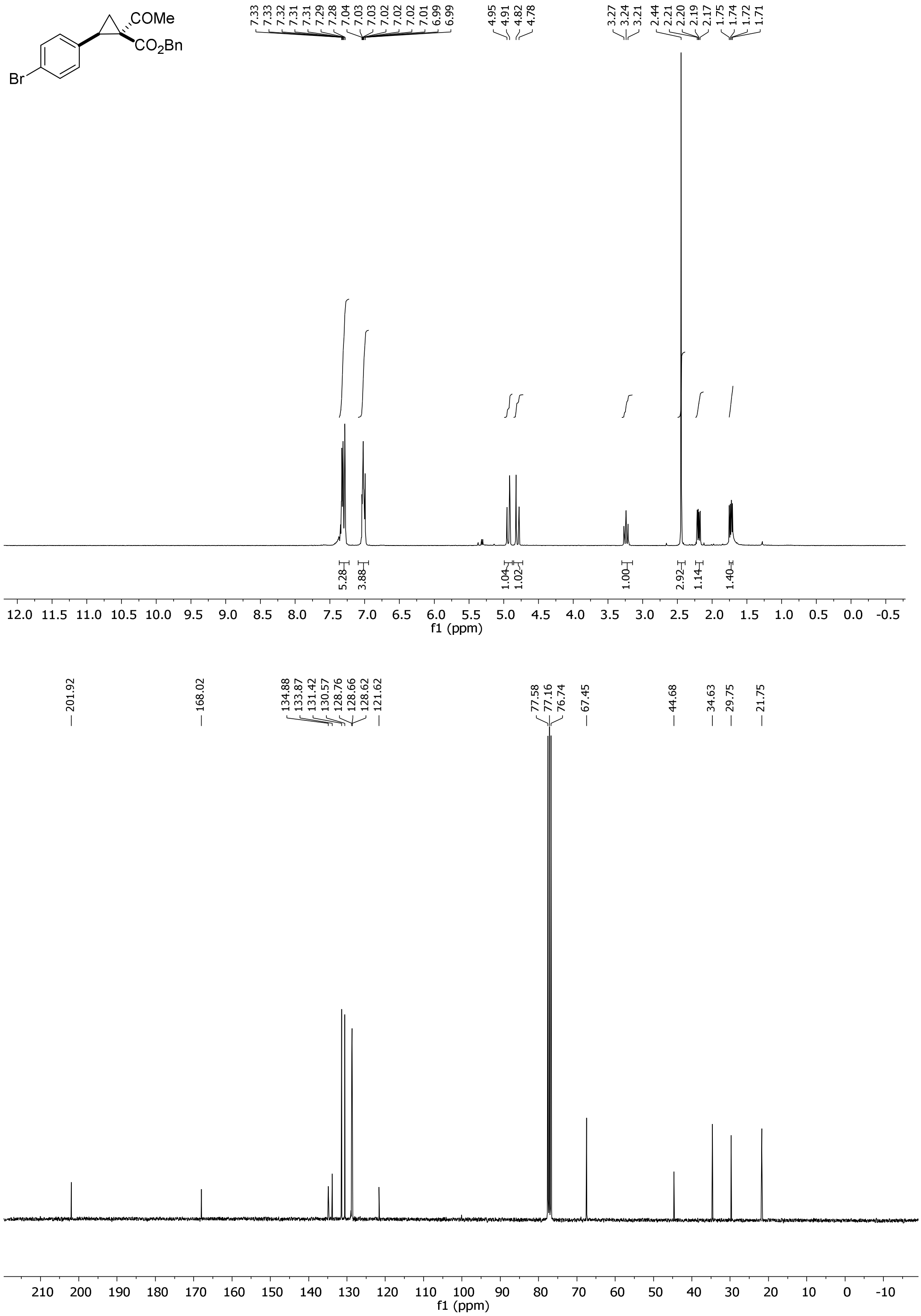

Figure S13. ^{1}H-NMR (300 MHz, $CDCl_3$) and ^{13}C-NMR (75 MHz, $CDCl_3$) spectra of compound 1k

1l

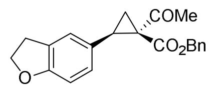

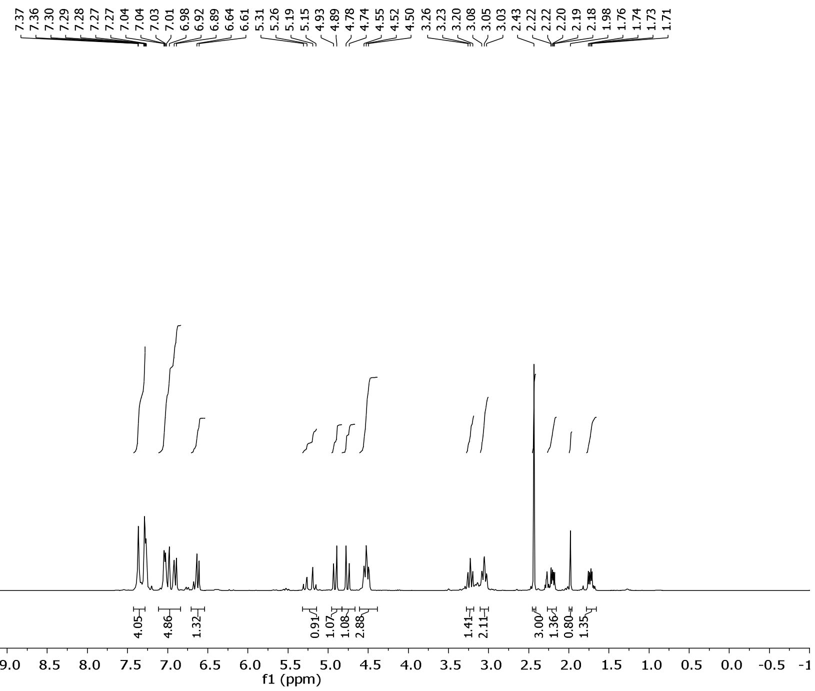

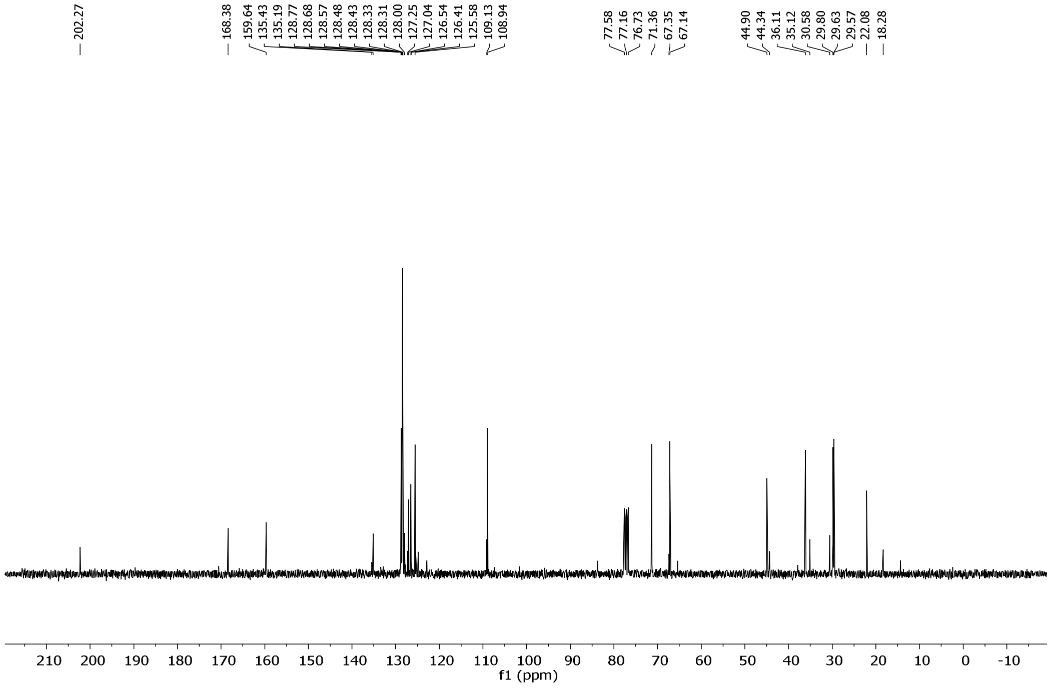

Figure S14. ^{1}H-NMR (300 MHz, $CDCl_3$) and ^{13}C-NMR (75 MHz, $CDCl_3$) spectra of compound 1l

1m

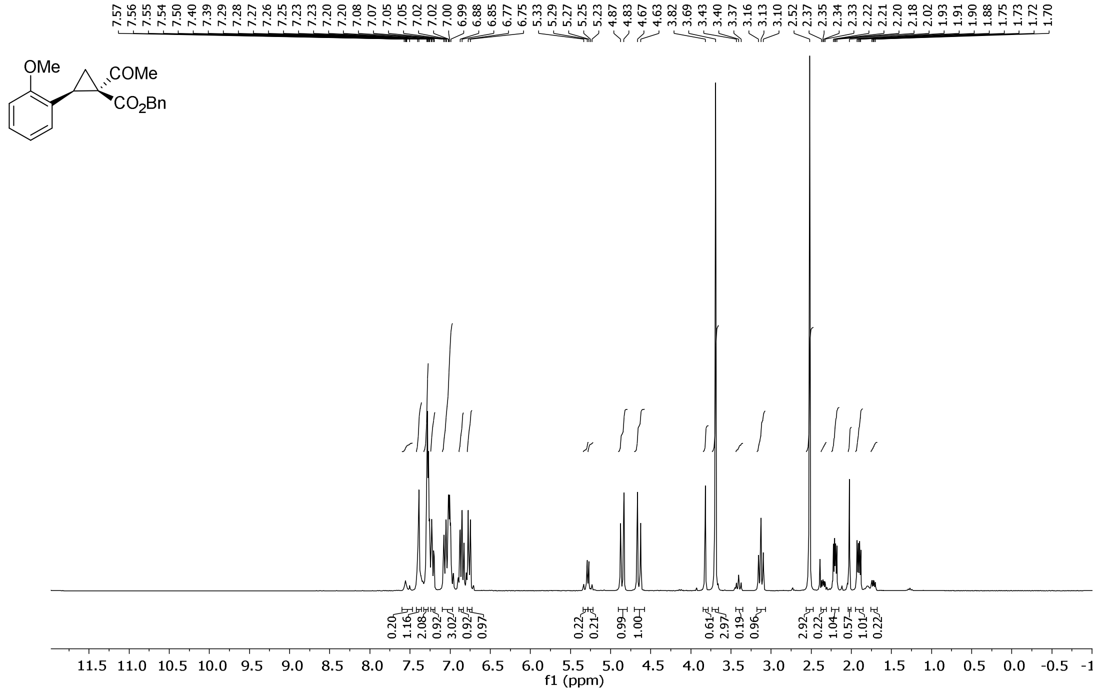

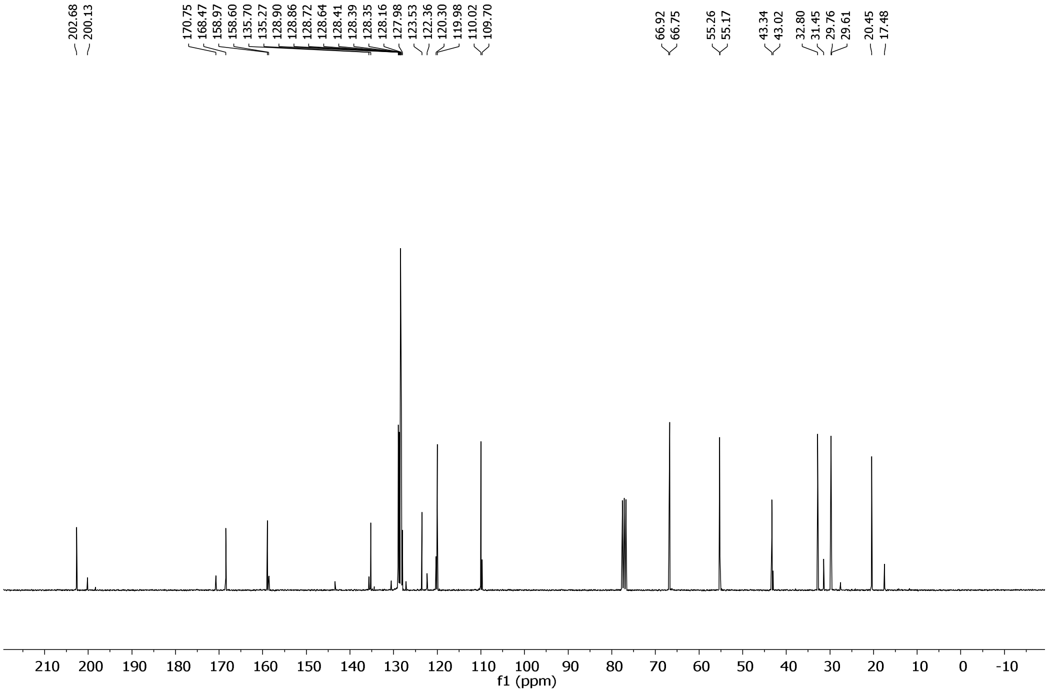

Figure S15. ^{1}H-NMR (300 MHz, $CDCl_3$) and ^{13}C-NMR (75 MHz, $CDCl_3$) spectra of compound 1m

1n

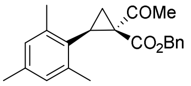

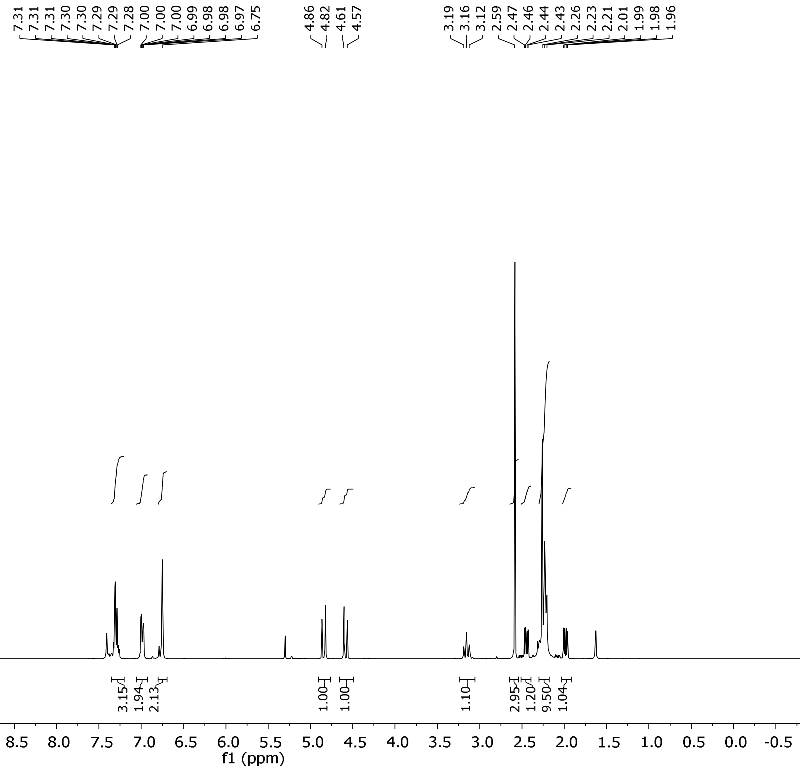

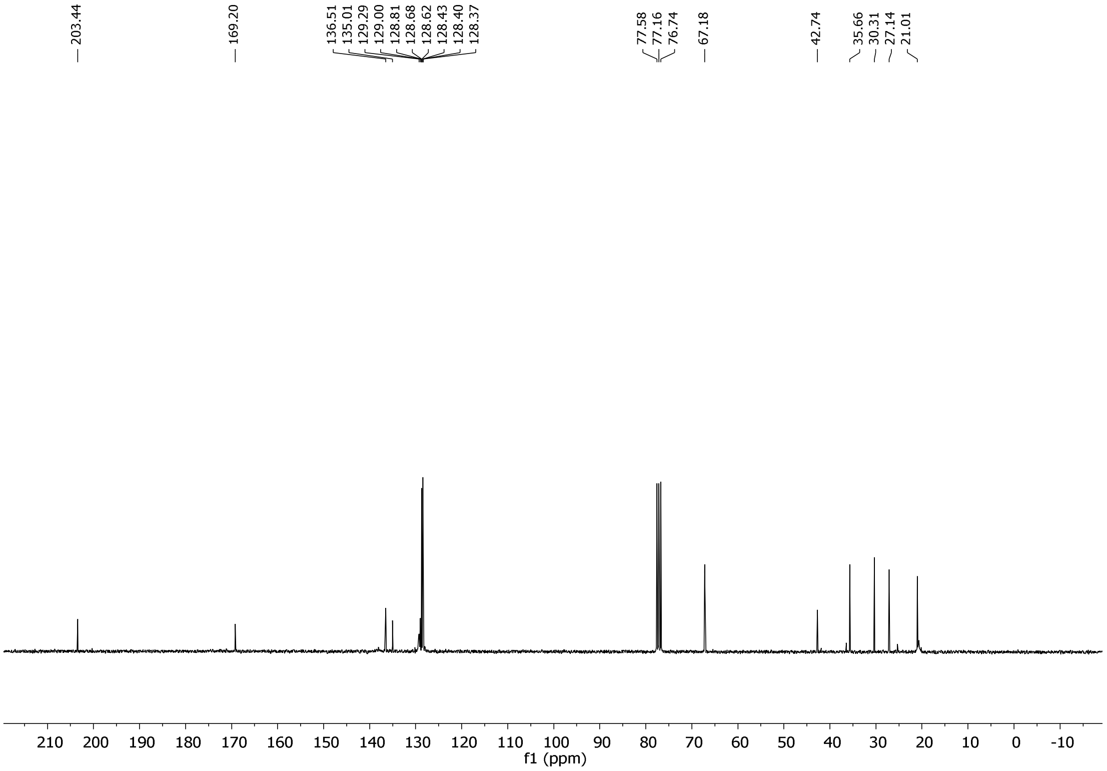

Figure S16. ^{1}H-NMR (300 MHz, $CDCl_3$) and ^{13}C-NMR (75 MHz, $CDCl_3$) spectra of compound 1n

1o

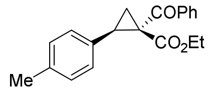

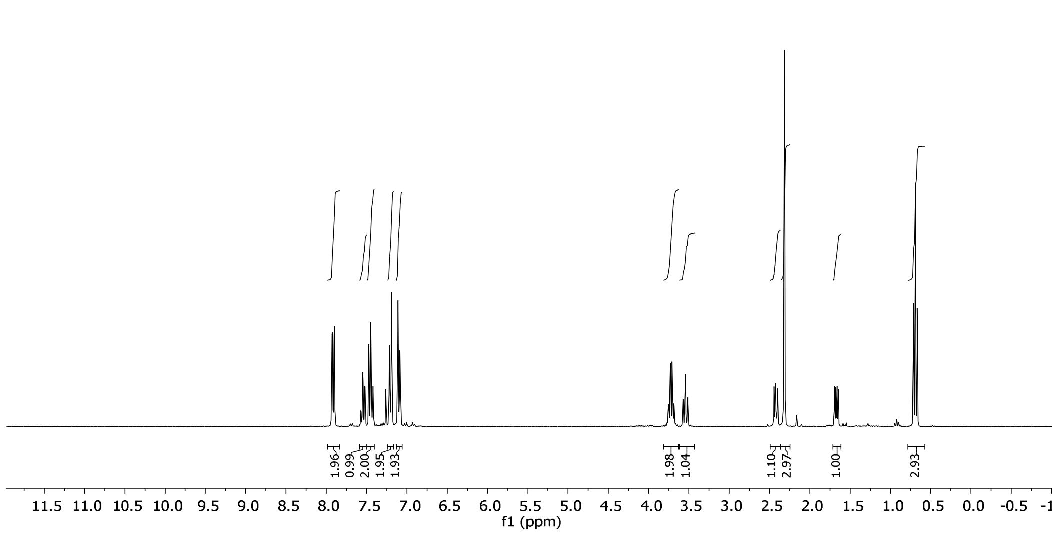

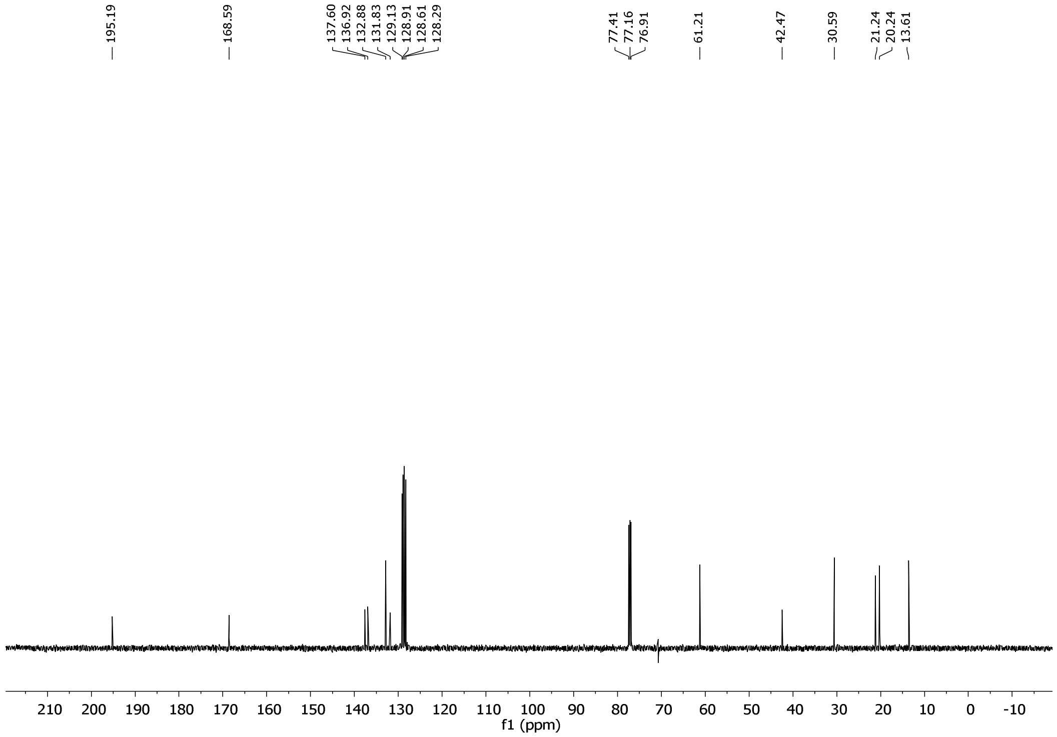

Figure S17. **^{1}H-NMR (300 MHz, $CDCl_3$) and ^{13}C-NMR (75 MHz, $CDCl_3$) spectra of compound 1o**

4.3 Dihydropyridoindoles 3 and 4

3a

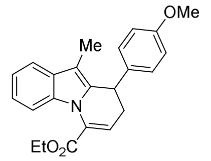

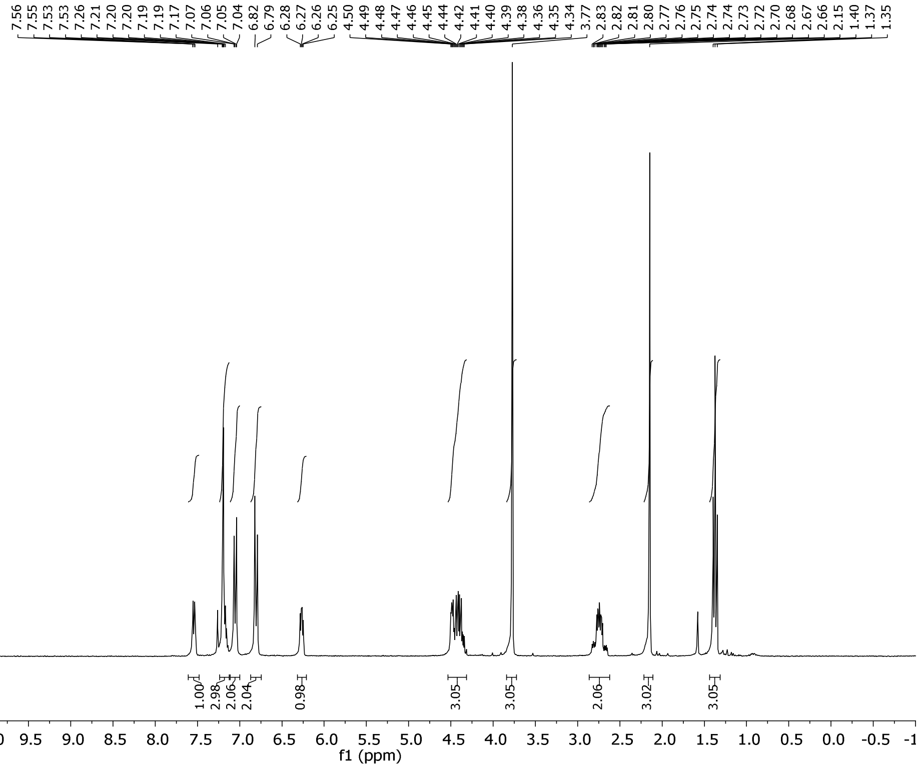

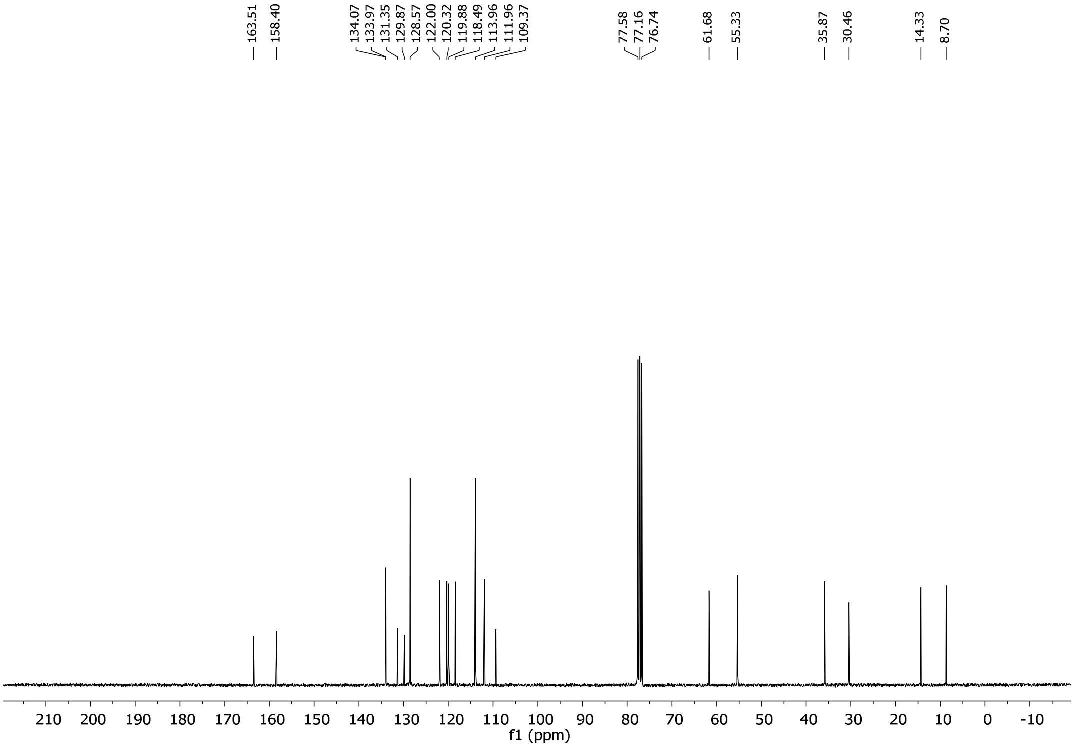

Figure S18. ^{1}**H-NMR (300 MHz, CDCl$_3$) and** ^{13}**C-NMR (75 MHz, CDCl$_3$) spectra of compound 3a**

4a

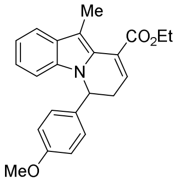

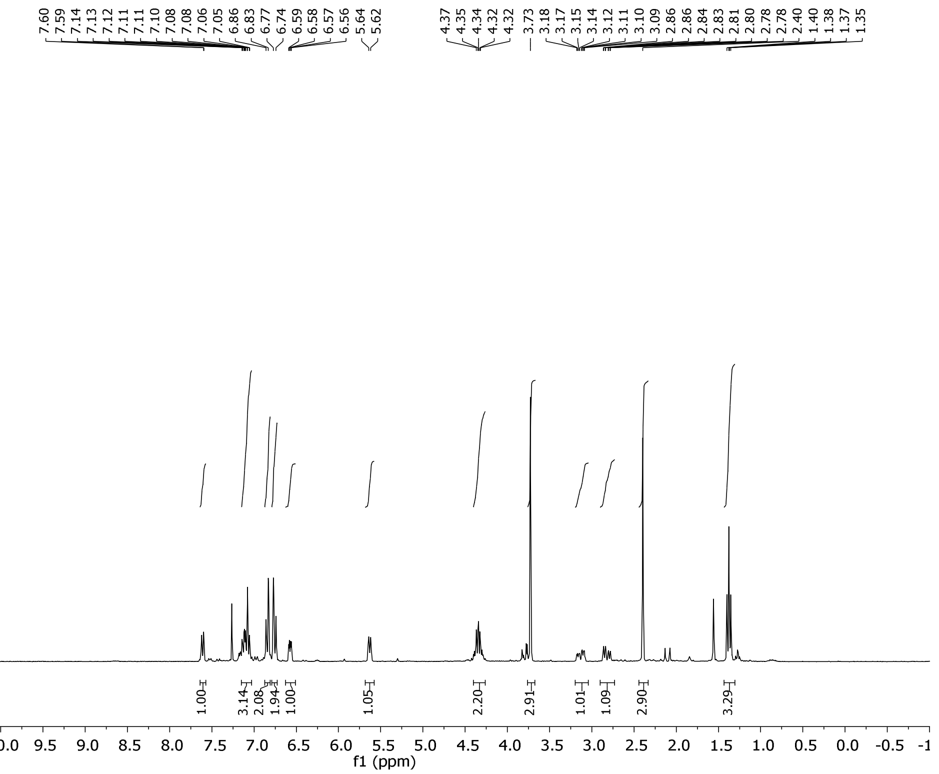

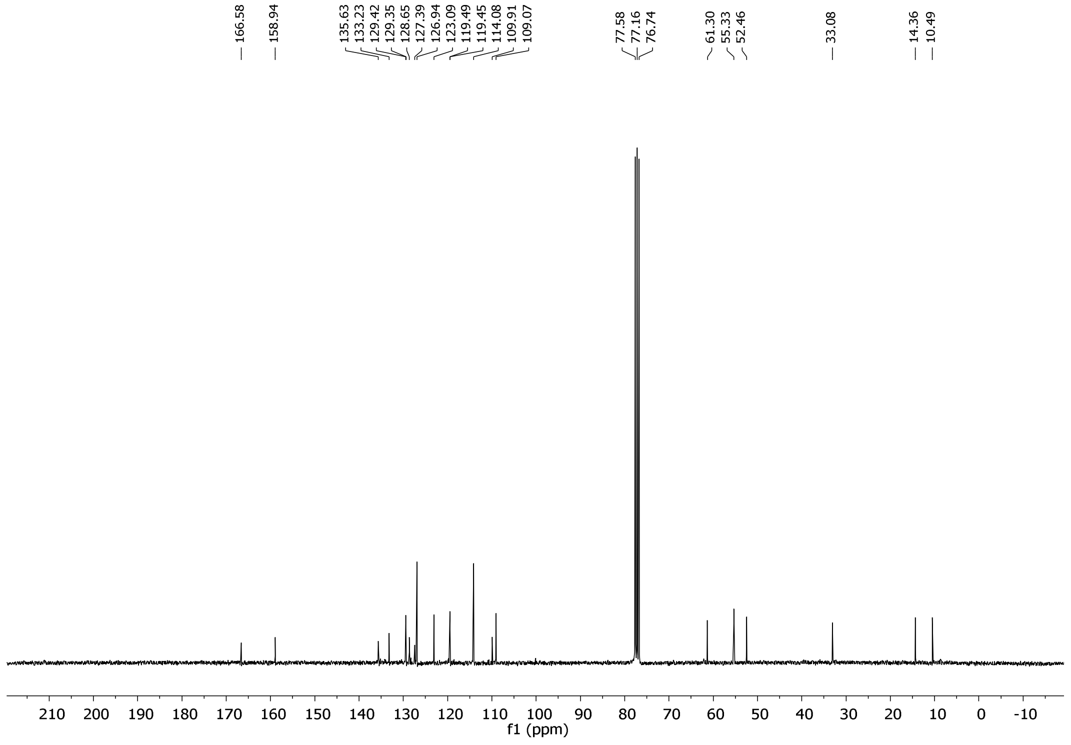

Figure S19. ^{1}H-NMR (300 MHz, $CDCl_3$) and ^{13}C-NMR (75 MHz, $CDCl_3$) spectra of compound 4a

3b

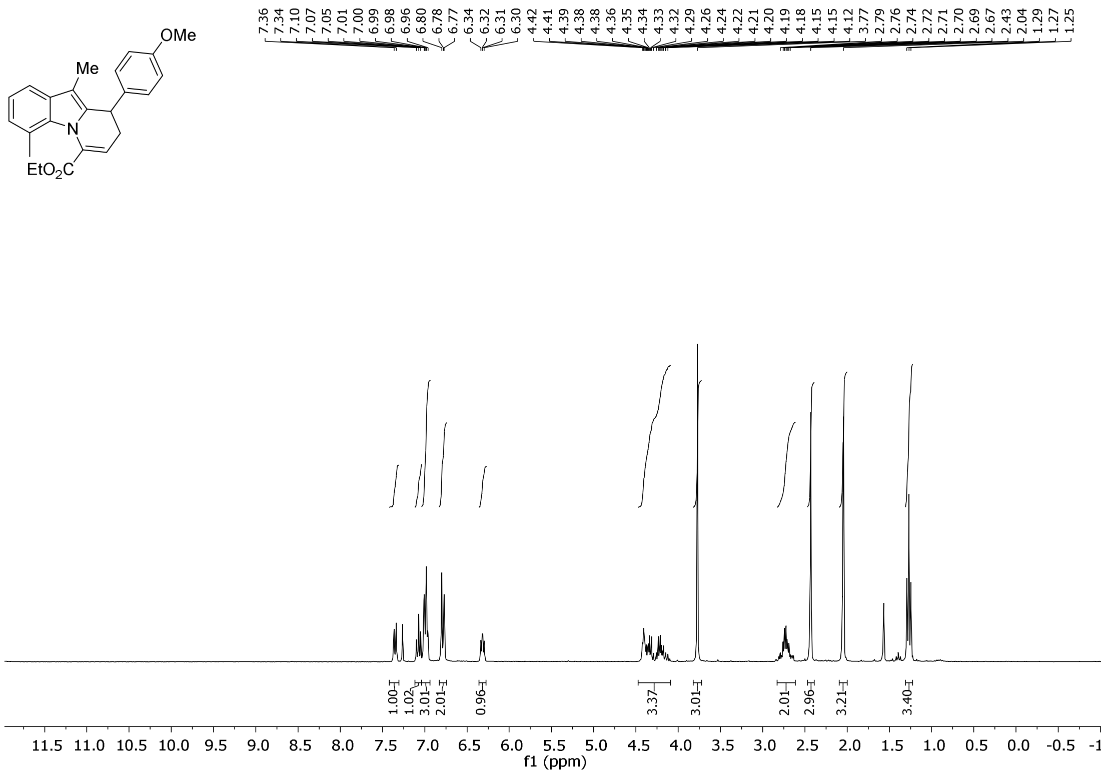

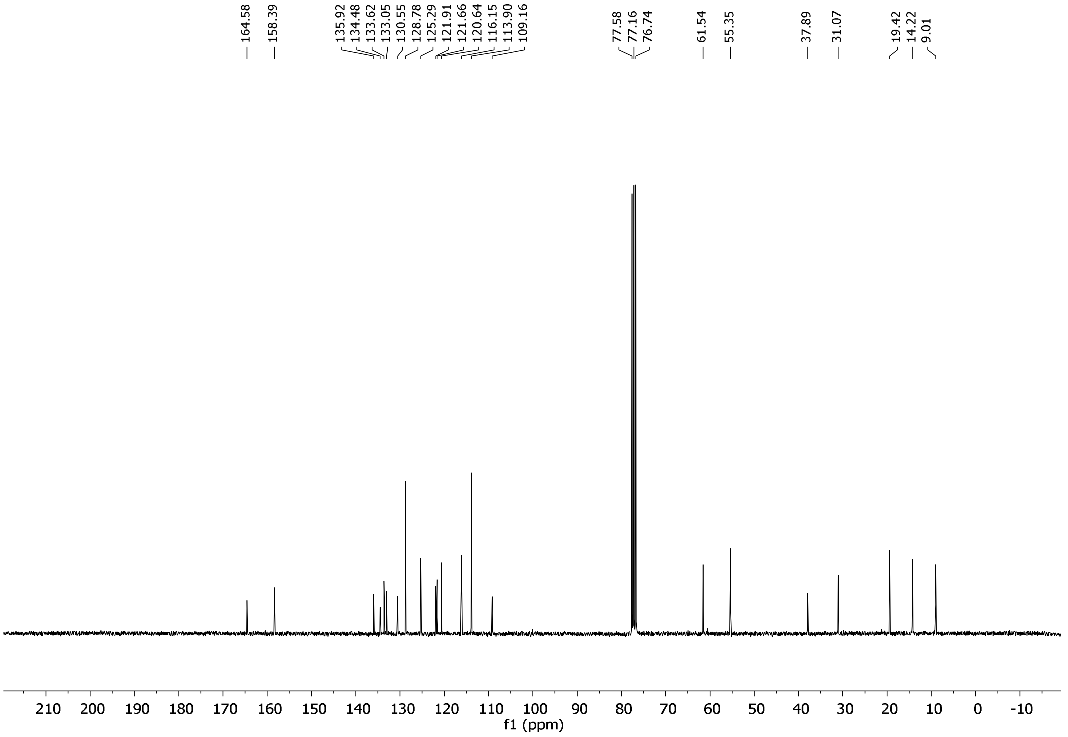

Figure S20. ^{1}H-NMR (300 MHz, $CDCl_3$) and ^{13}C-NMR (75 MHz, $CDCl_3$) spectra of compound 3b

4b

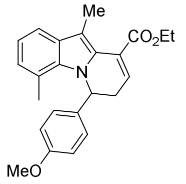

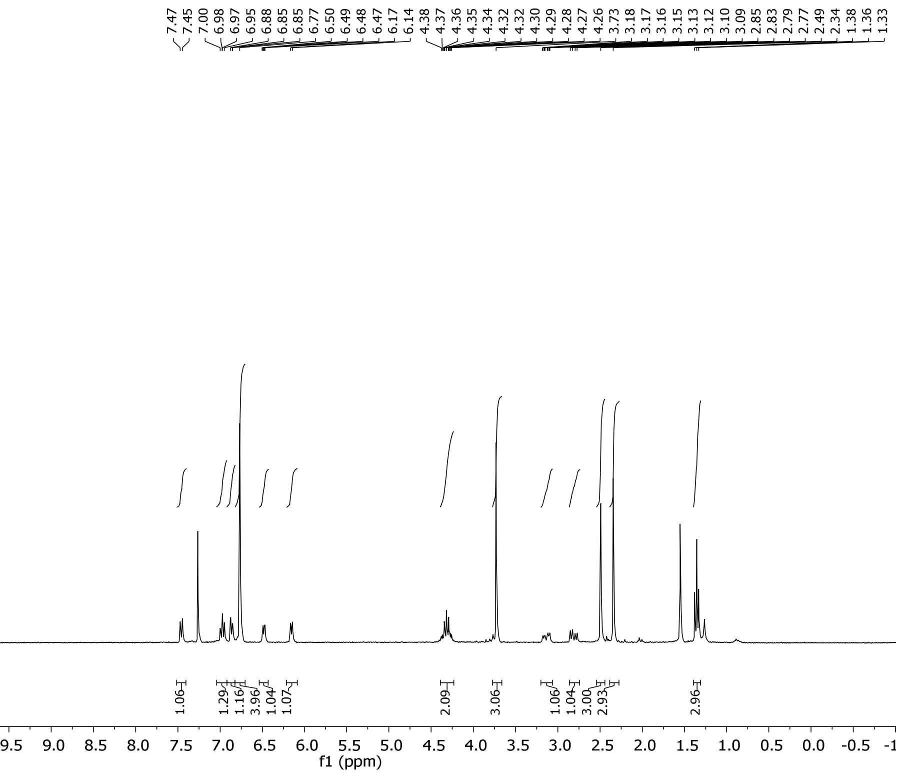

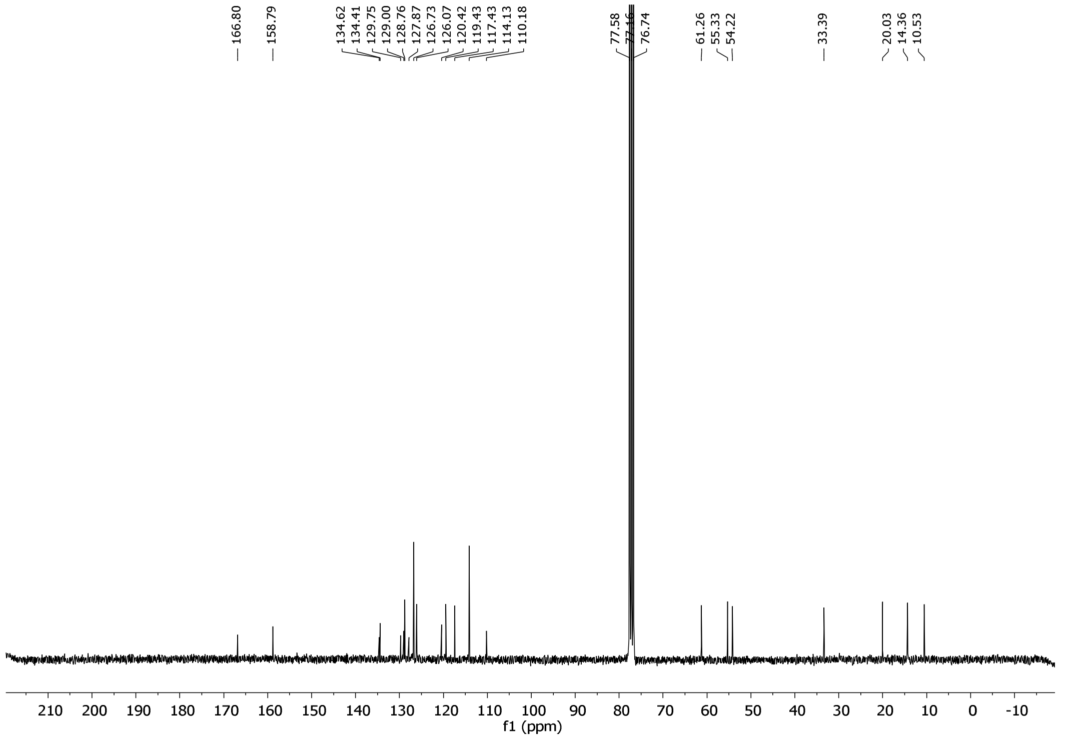

Figure S21. ^{1}**H-NMR (300 MHz, CDCl$_3$) and** ^{13}**C-NMR (75 MHz, CDCl$_3$) spectra of compound 4b**

3c

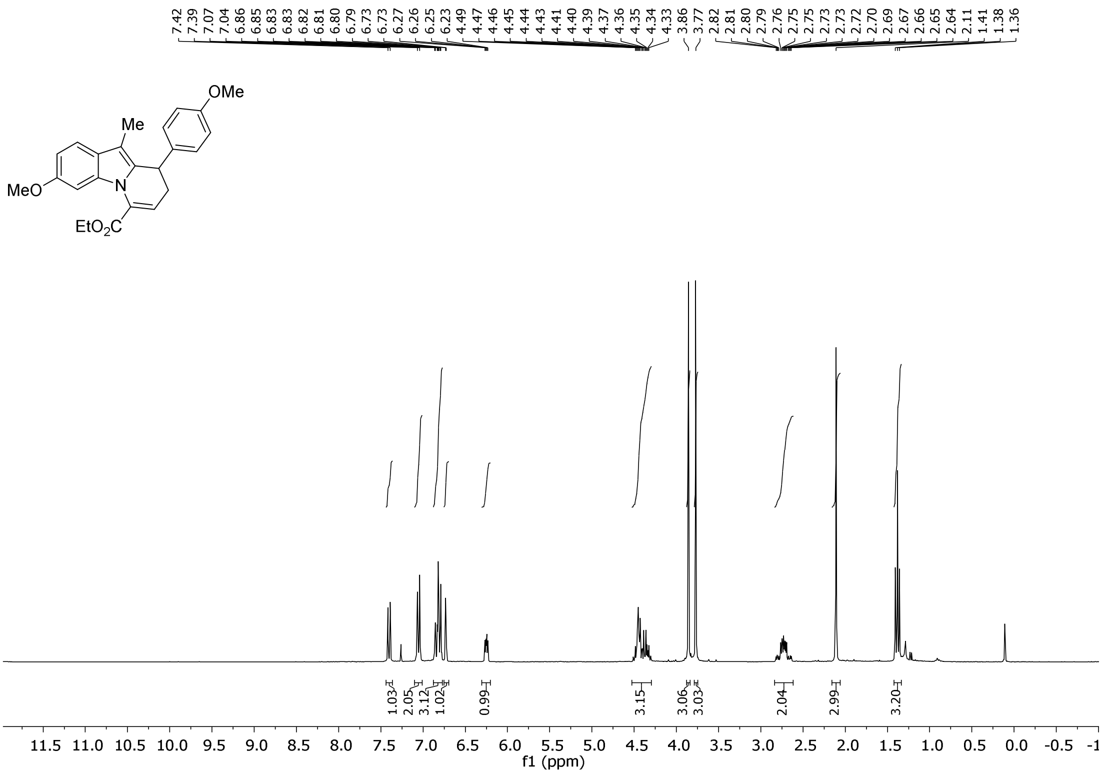

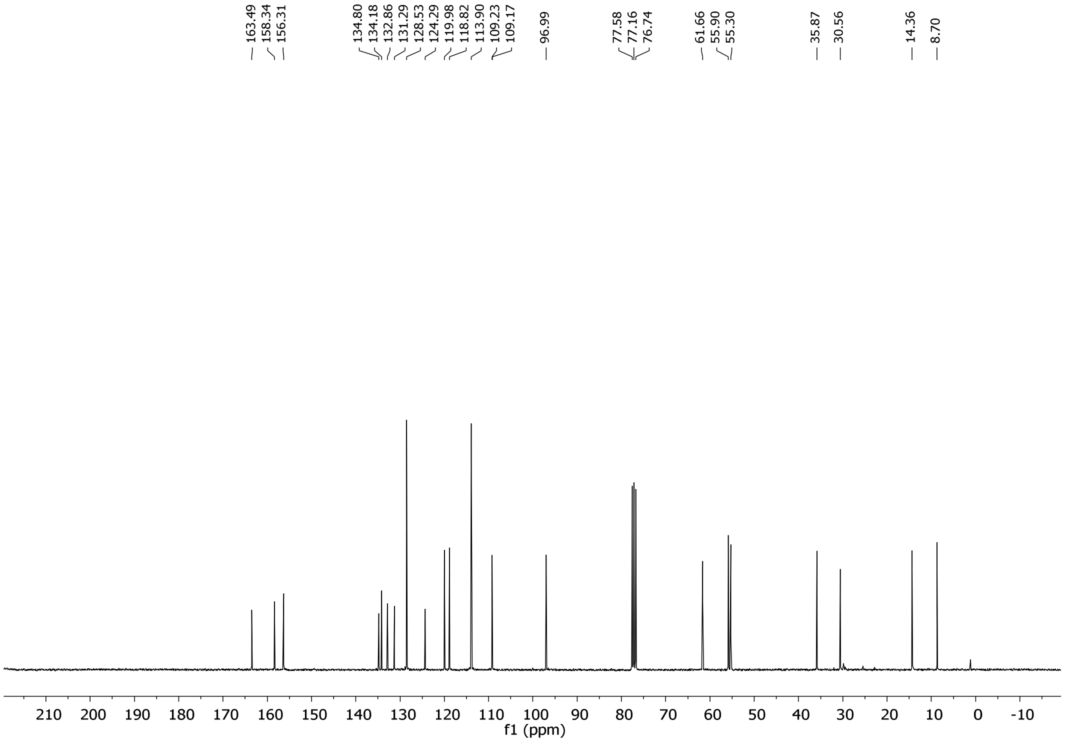

Figure S22. **^{1}H-NMR (300 MHz, $CDCl_3$) and ^{13}C-NMR (75 MHz, $CDCl_3$) spectra of compound 3c**

4c

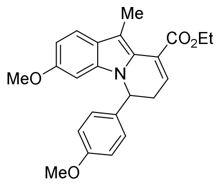

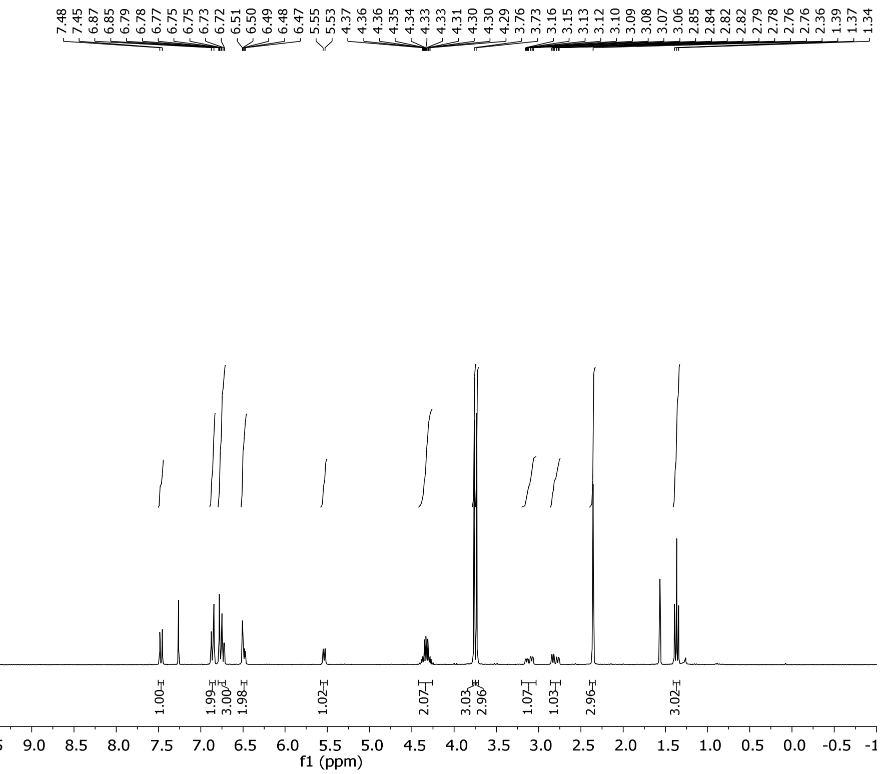

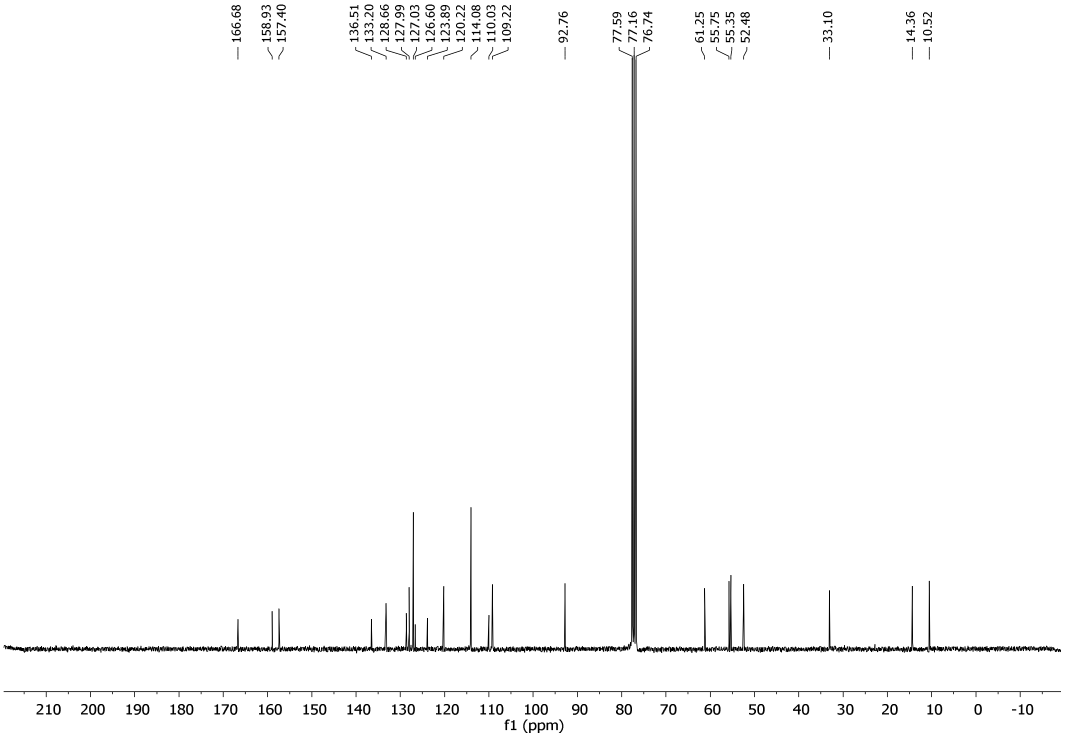

Figure S23. ^{1}H-NMR (300 MHz, $CDCl_3$) and ^{13}C-NMR (75 MHz, $CDCl_3$) spectra of compound 4c

3d

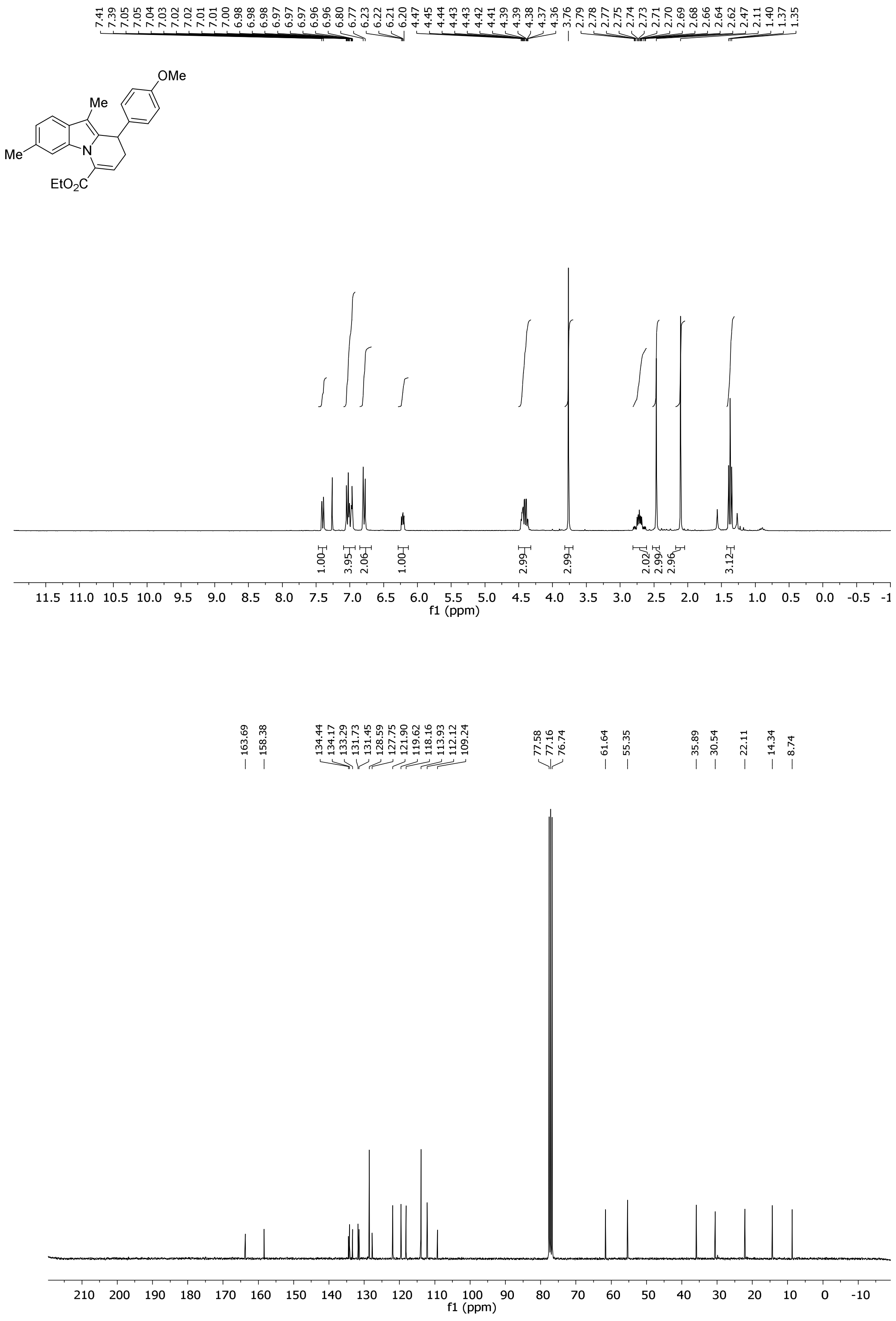

Figure S24. ^{1}H-NMR (300 MHz, $CDCl_3$) and ^{13}C-NMR (75 MHz, $CDCl_3$) spectra of compound 3d

4d

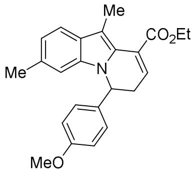

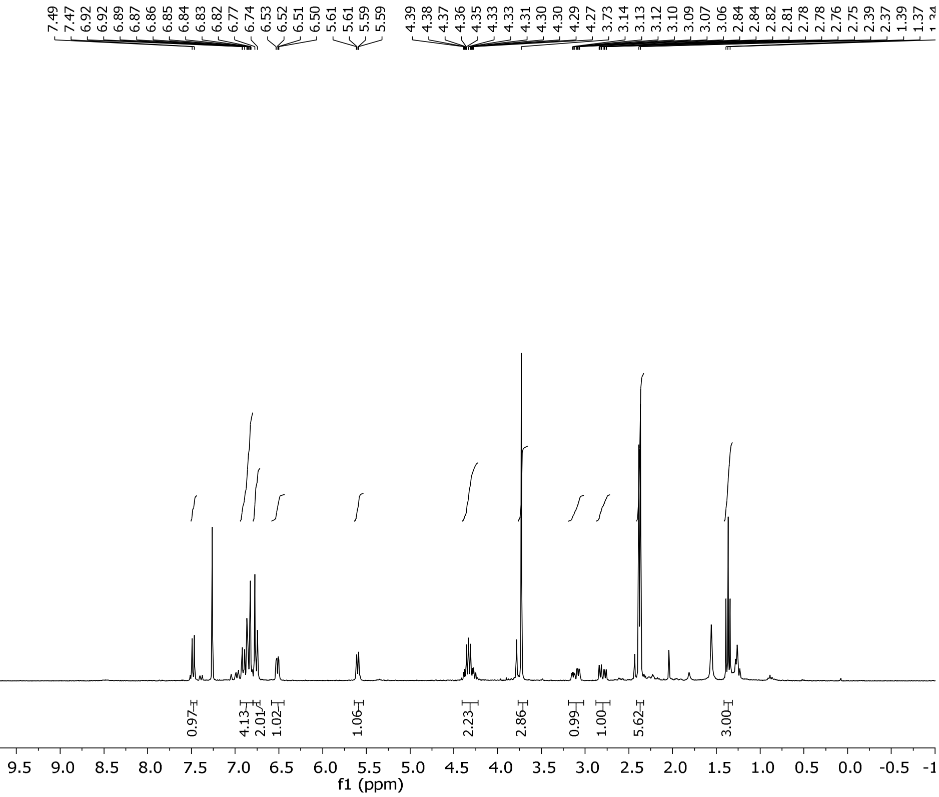

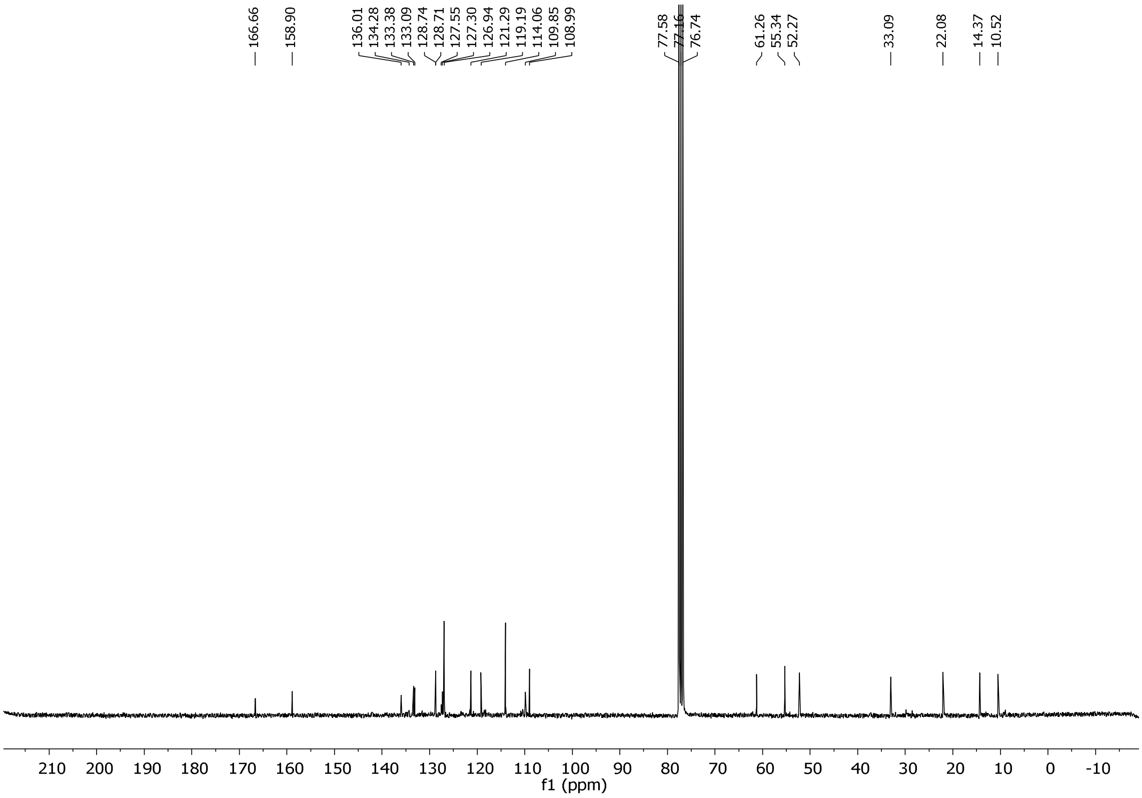

Figure S25. ^{1}H-NMR (300 MHz, $CDCl_3$) and ^{13}C-NMR (75 MHz, $CDCl_3$) spectra of compound 4d

3e

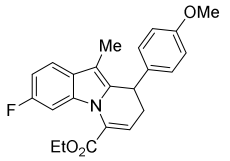

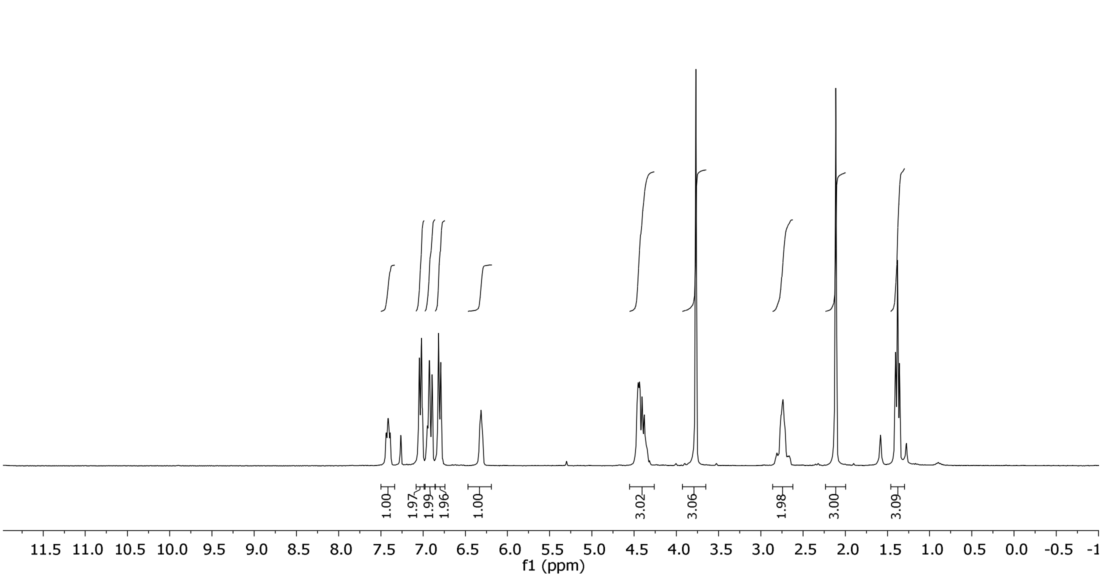

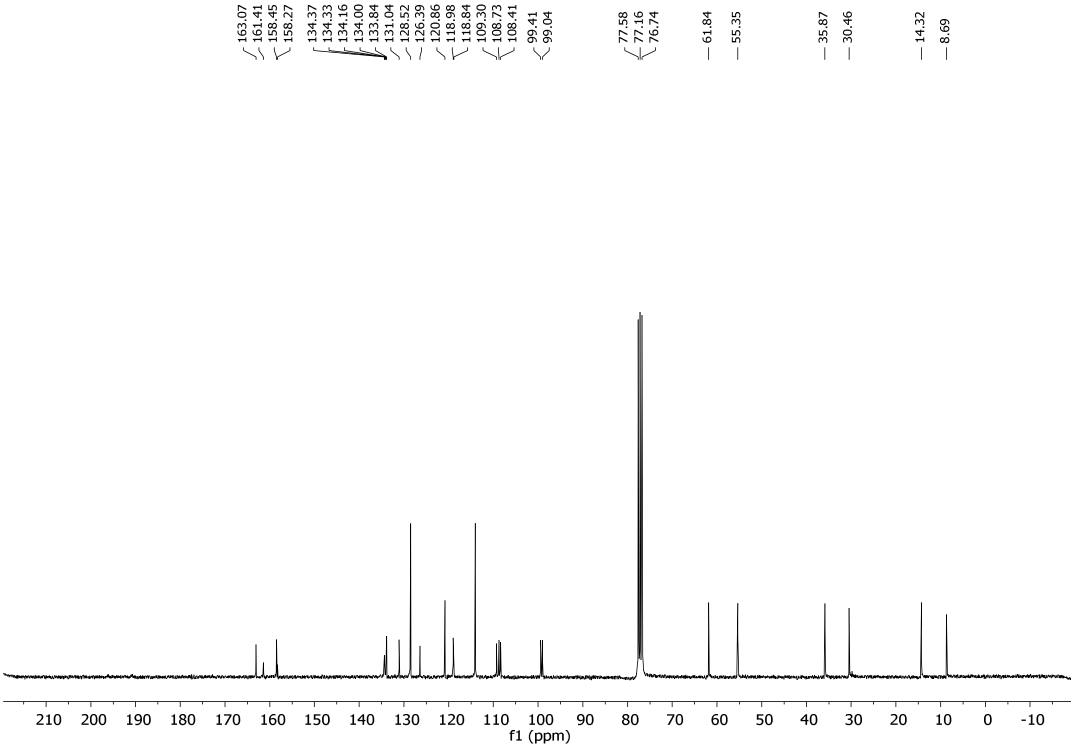

Figure S26. ^{1}H-NMR (300 MHz, $CDCl_3$) and ^{13}C-NMR (75 MHz, $CDCl_3$) spectra of compound 3e

4e

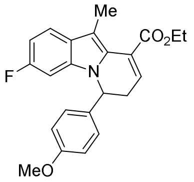

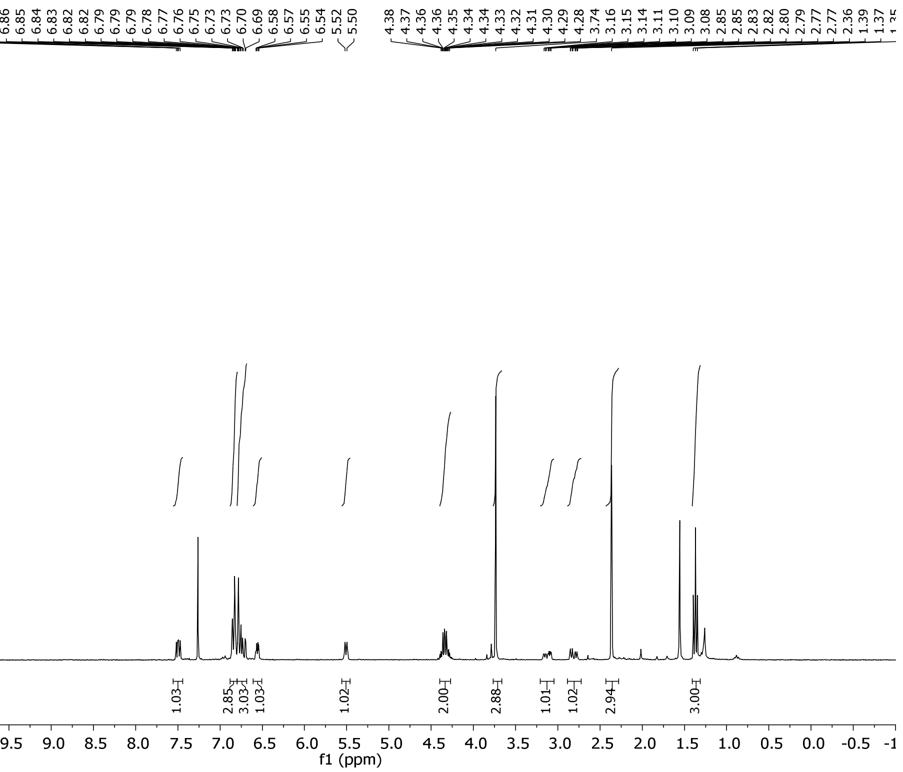

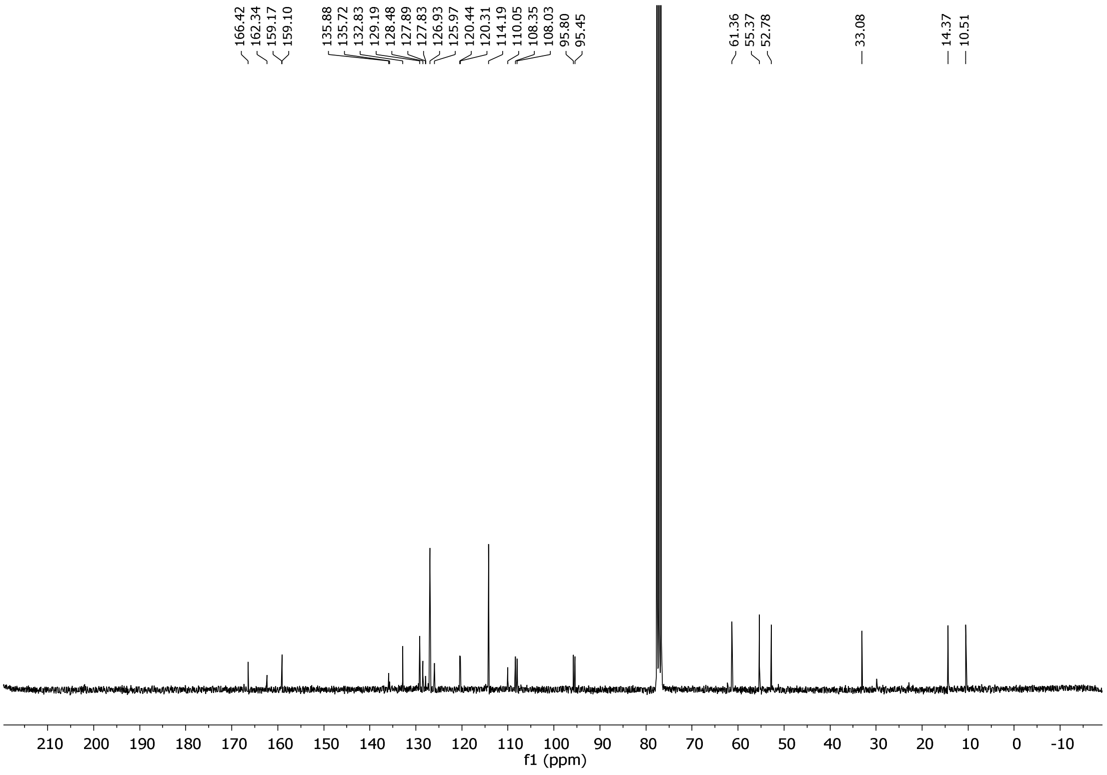

Figure S27. [1]**H-NMR (300 MHz, $CDCl_3$) and** [13]**C-NMR (75 MHz, $CDCl_3$) spectra of compound 4e**

3f

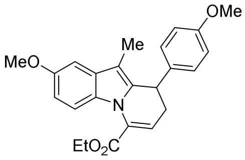

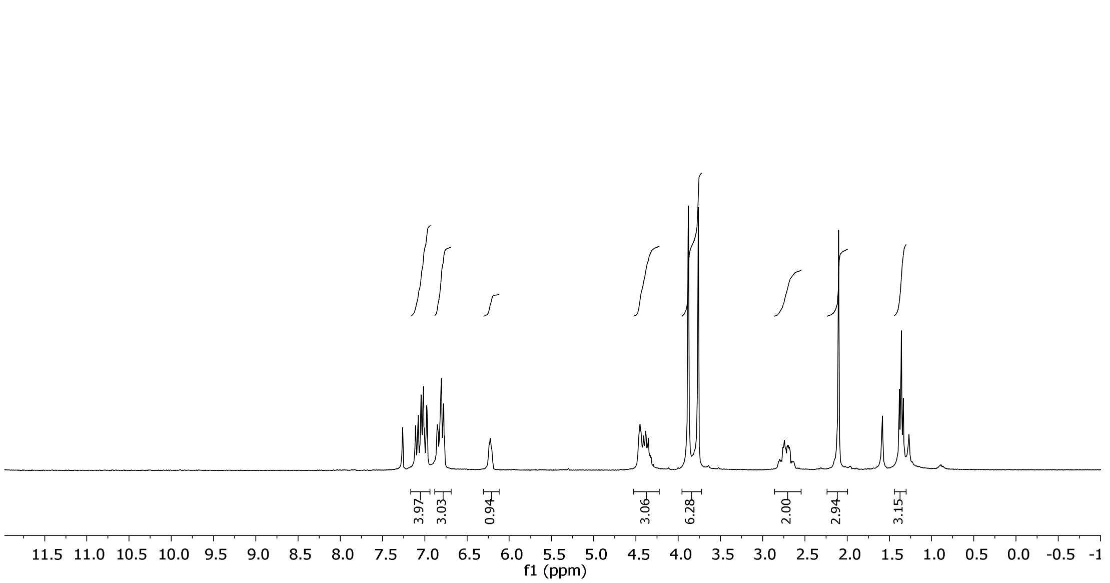

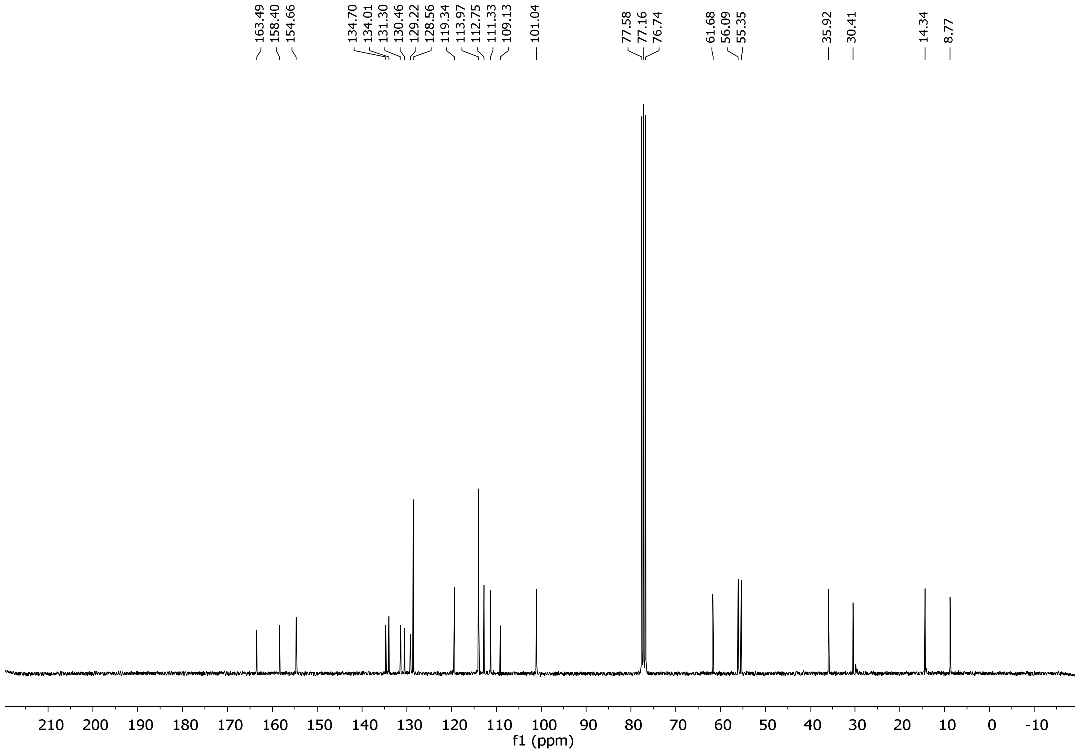

Figure S28. ^{1}H-NMR (300 MHz, $CDCl_3$) and ^{13}C-NMR (75 MHz, $CDCl_3$) spectra of compound 3f

4f

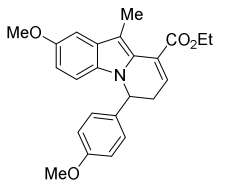

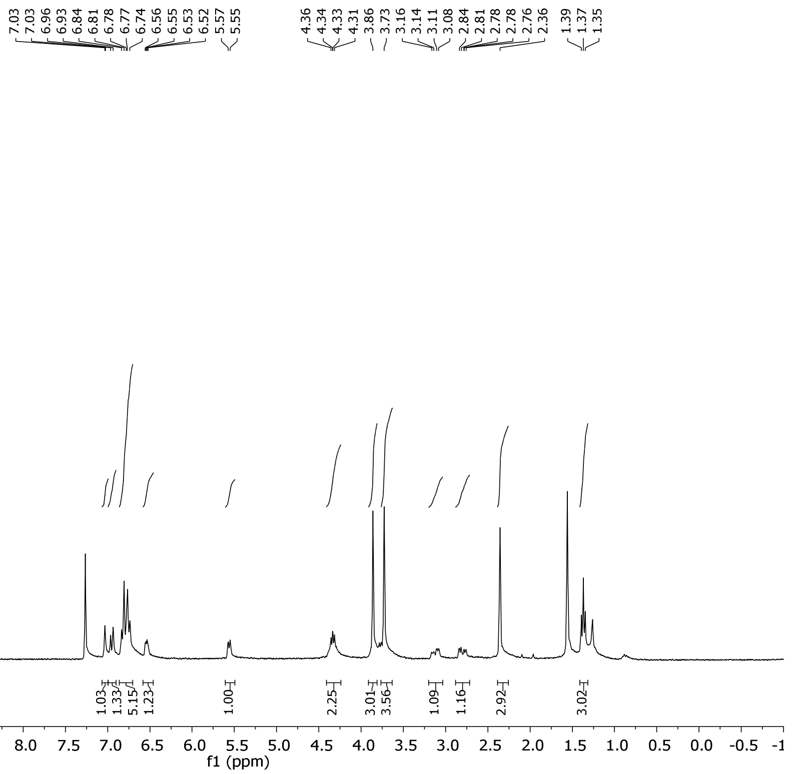

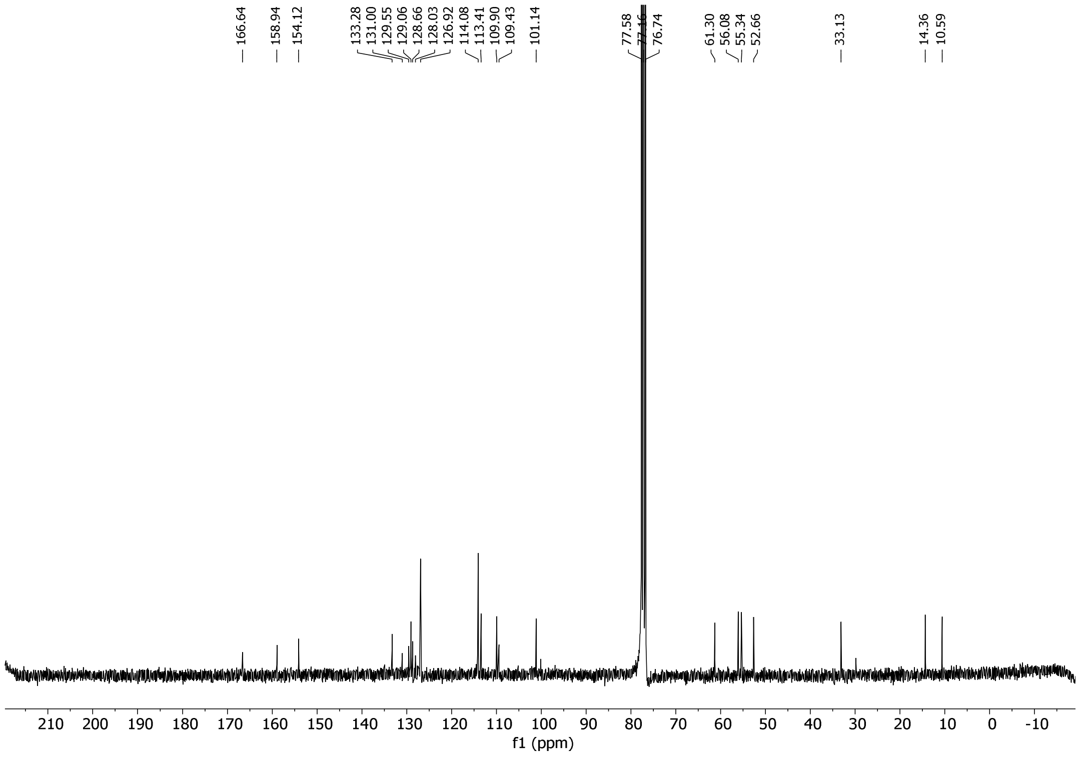

Figure S29. ^{1}H-NMR (300 MHz, $CDCl_3$) and ^{13}C-NMR (75 MHz, $CDCl_3$) spectra of compound 4f

3g

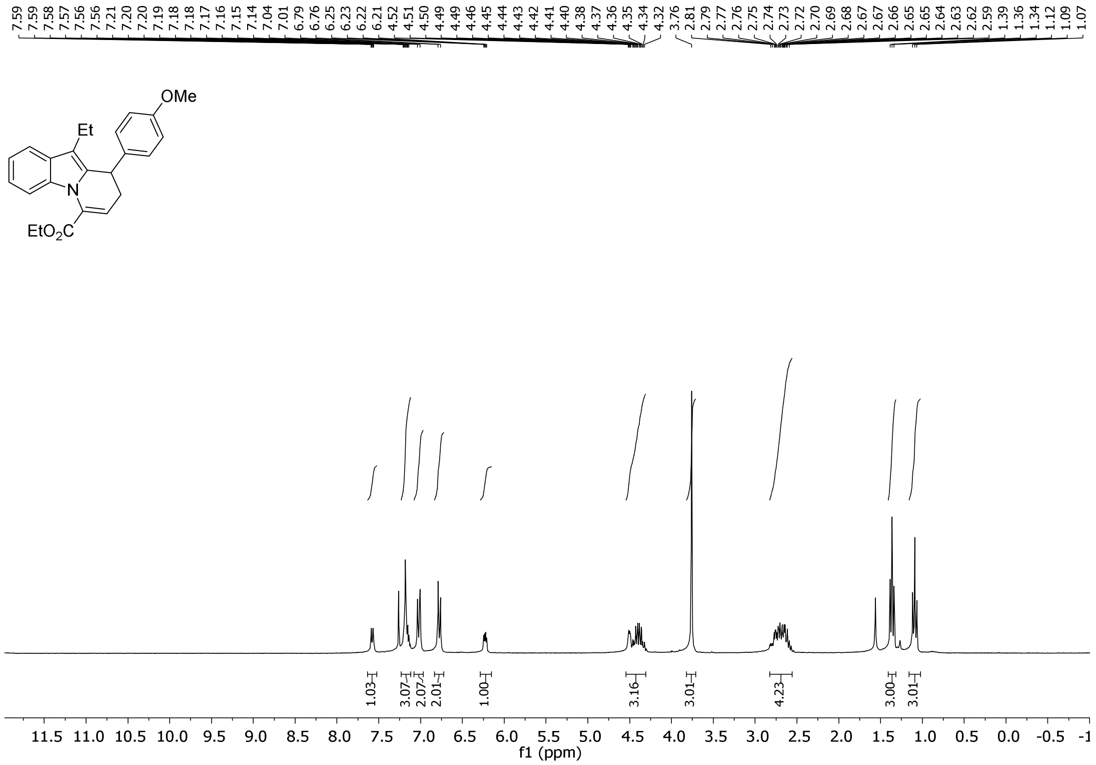

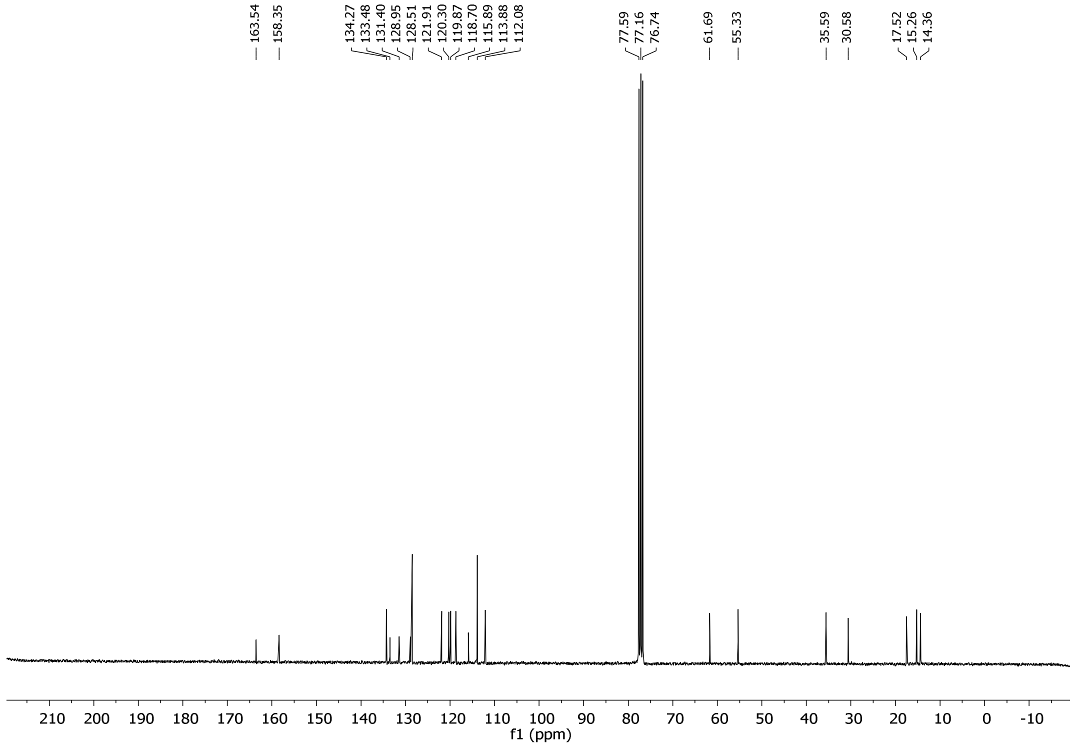

Figure S30. **^{1}H-NMR (300 MHz, $CDCl_3$) and ^{13}C-NMR (75 MHz, $CDCl_3$) spectra of compound 3g**

4g

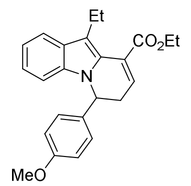

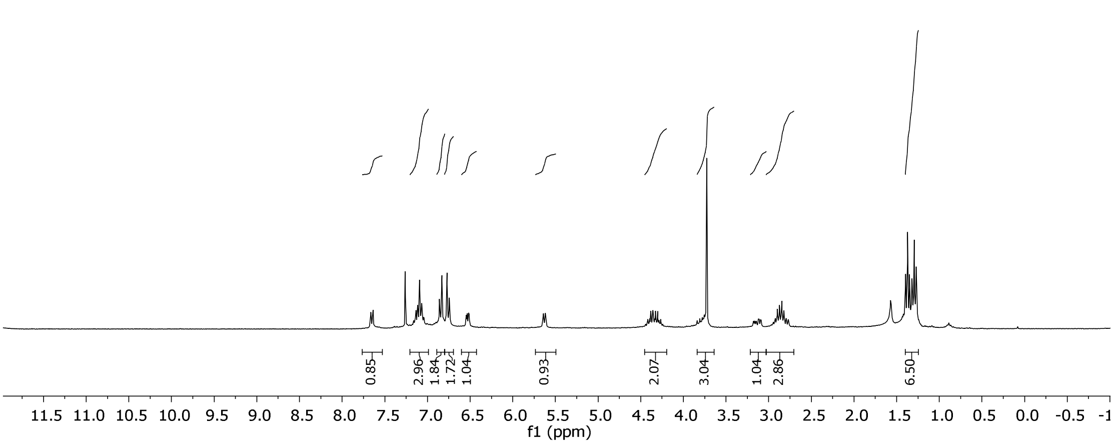

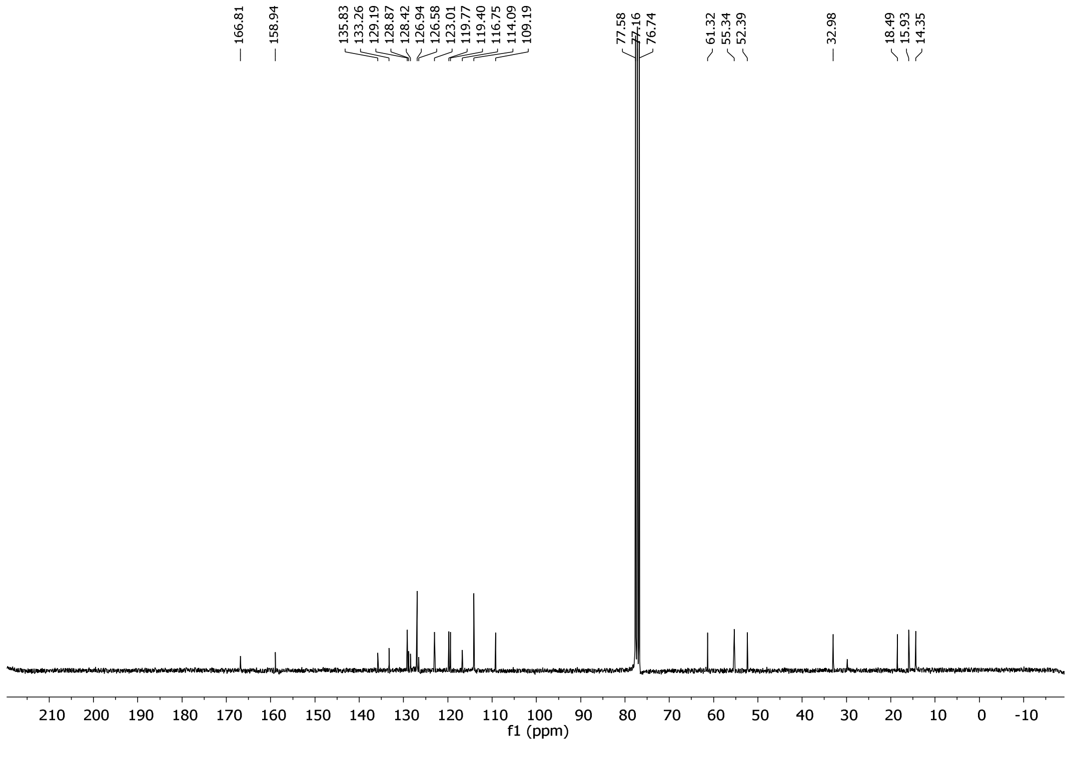

Figure S31. ^{1}H**-NMR (300 MHz,** $CDCl_3$**) and** ^{13}C**-NMR (75 MHz,** $CDCl_3$**) spectra of compound 4g**

3h

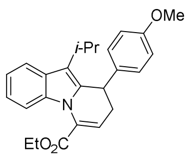

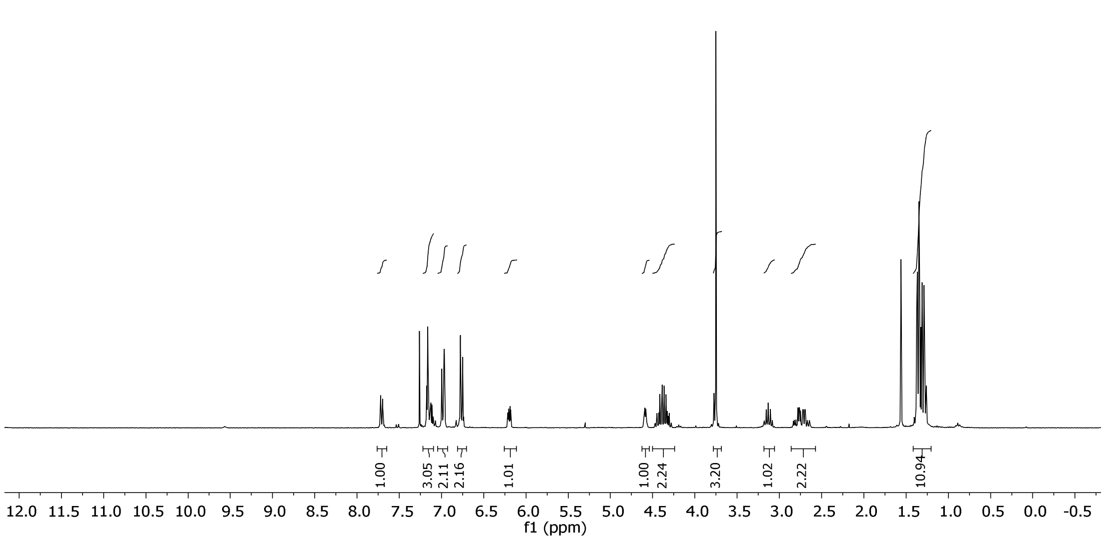

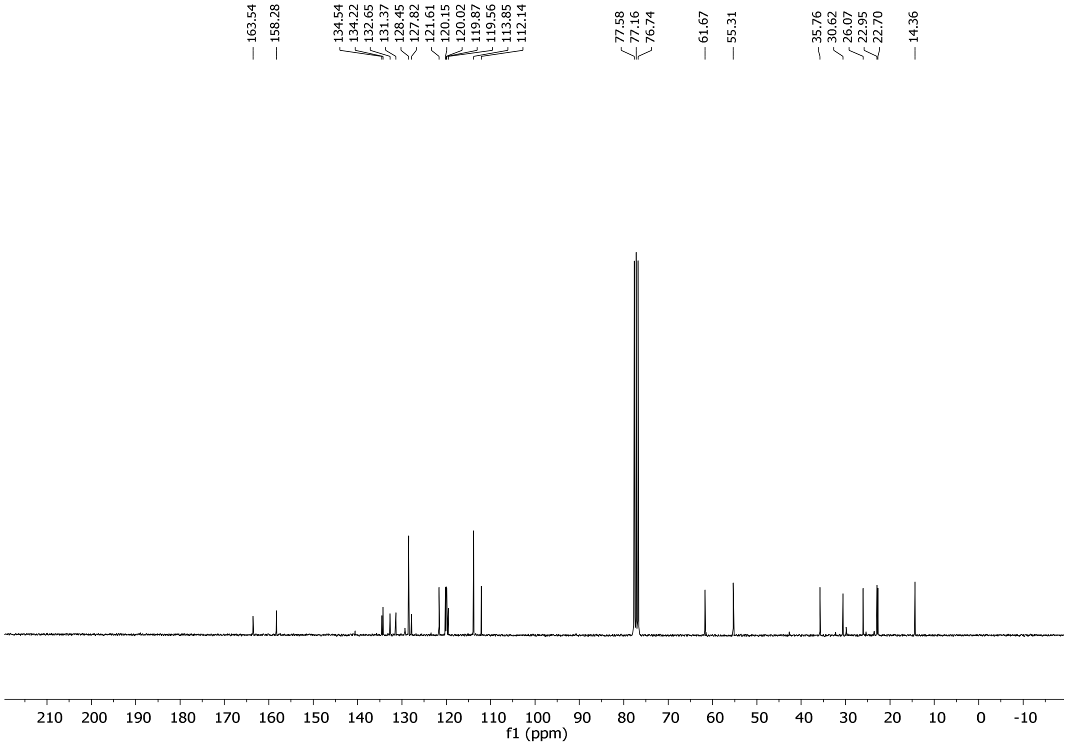

Figure S32. ^{1}H-NMR (300 MHz, $CDCl_3$) and ^{13}C-NMR (75 MHz, $CDCl_3$) spectra of compound 3h

3i

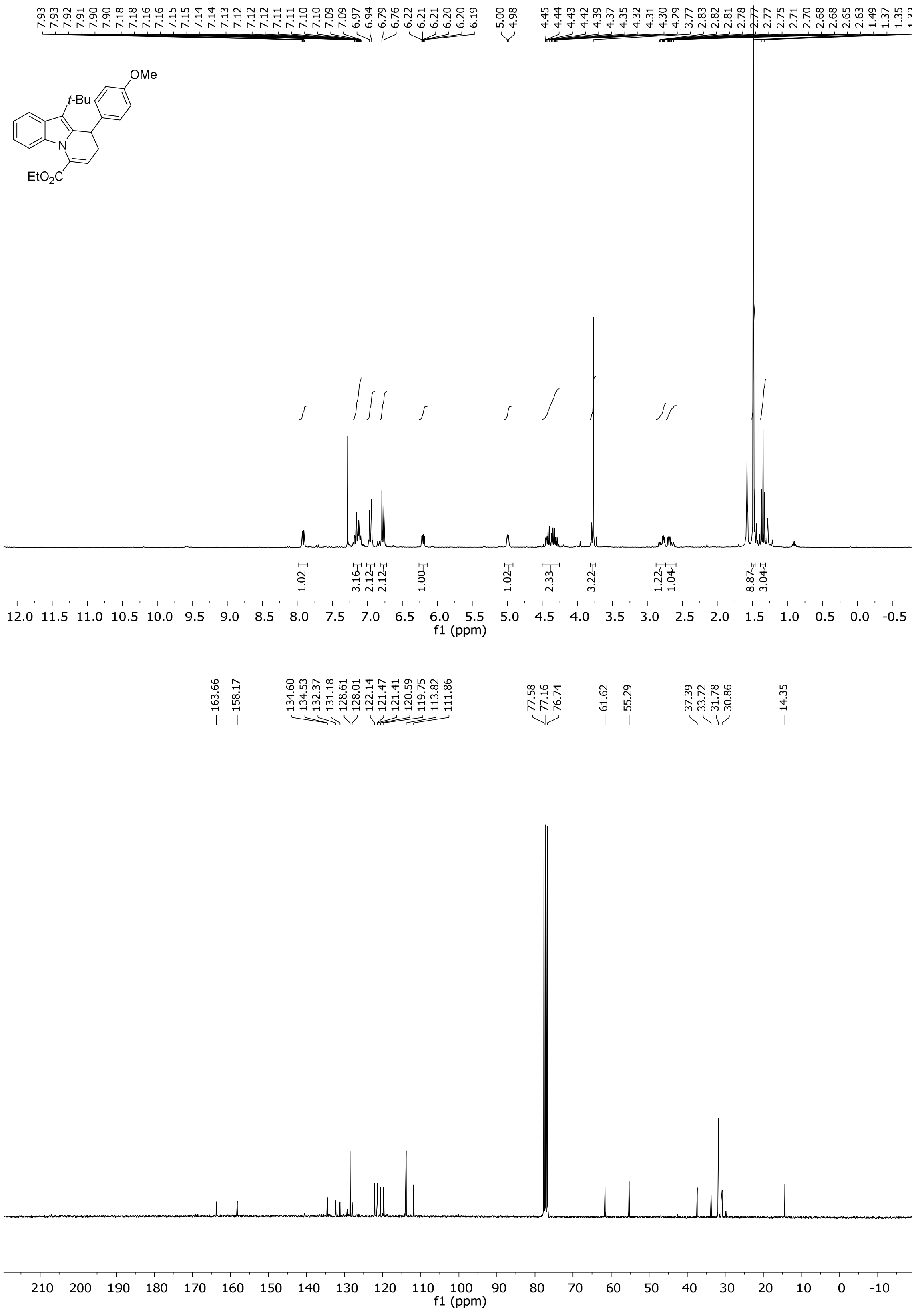

Figure S33. ^{1}H-NMR (300 MHz, $CDCl_3$) and ^{13}C-NMR (75 MHz, $CDCl_3$) spectra of compound 3i

3j

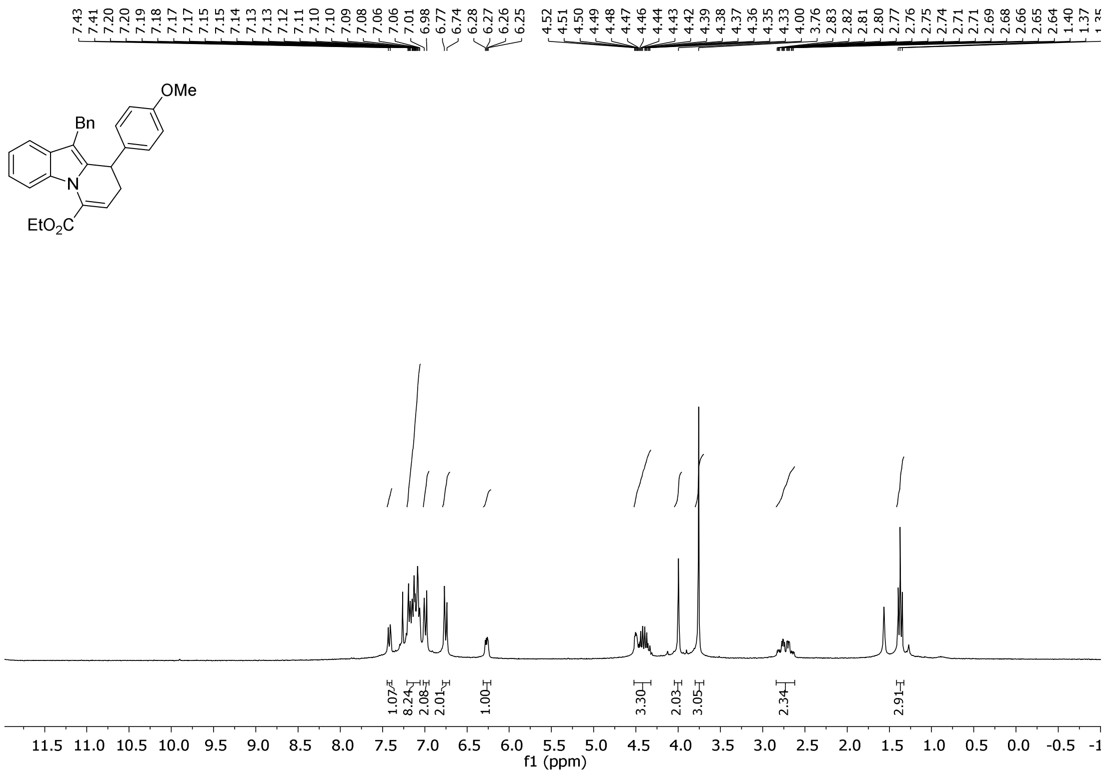

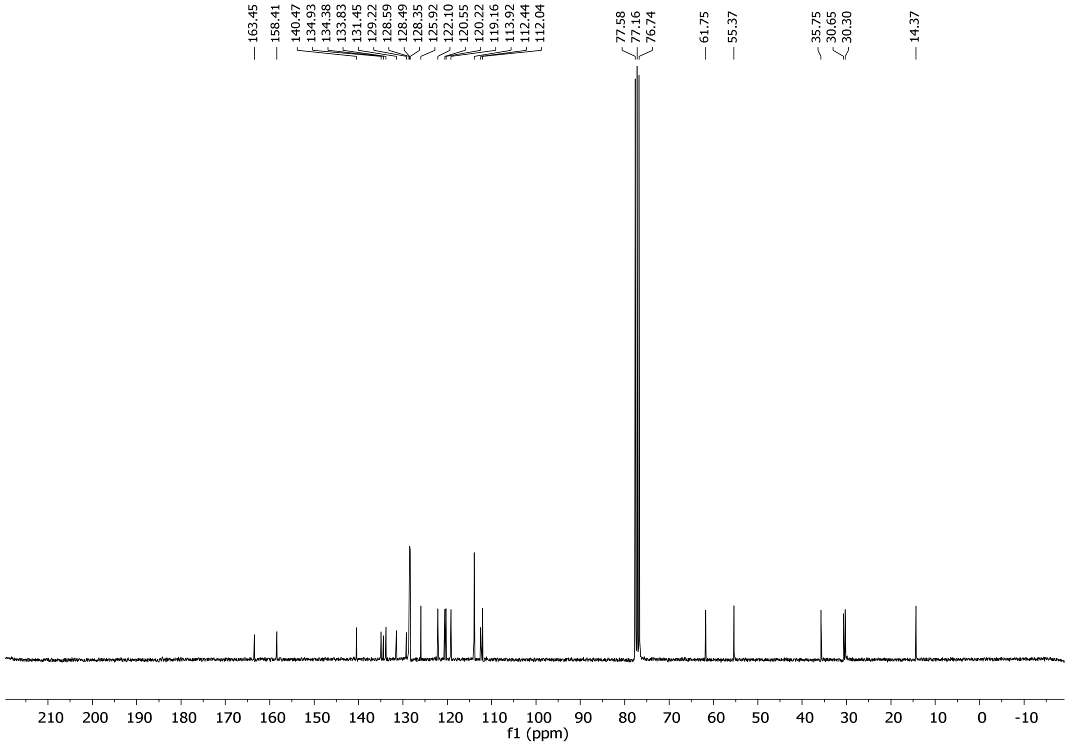

Figure S34. ^{1}H-NMR (300 MHz, $CDCl_3$) and ^{13}C-NMR (75 MHz, $CDCl_3$) spectra of compound 3j

4j

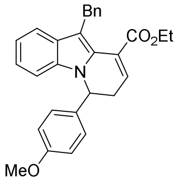

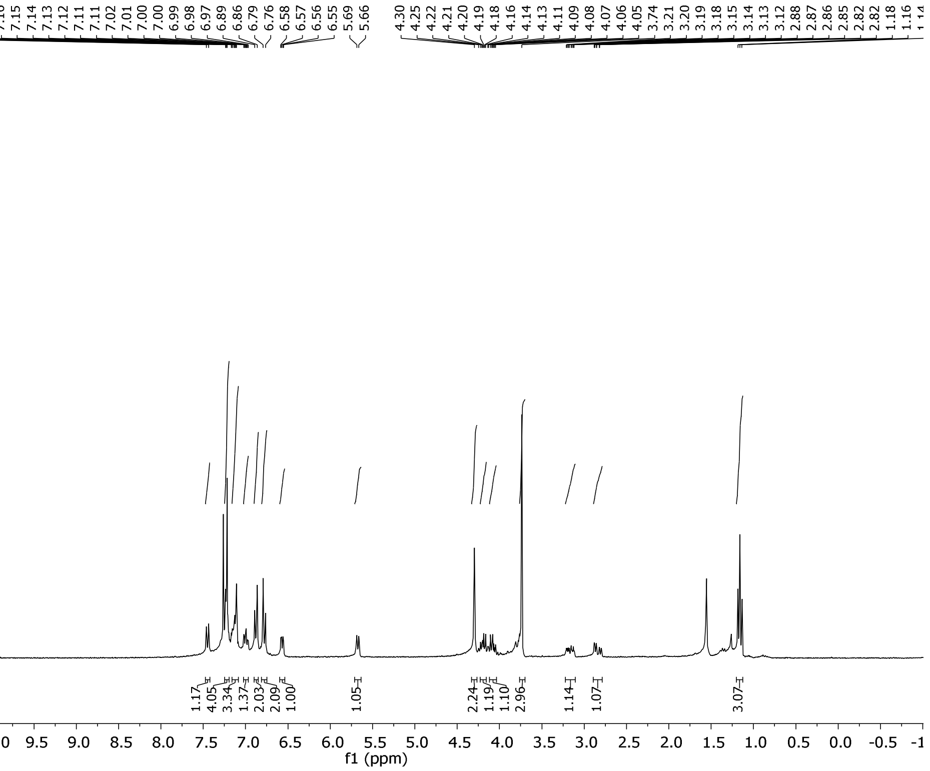

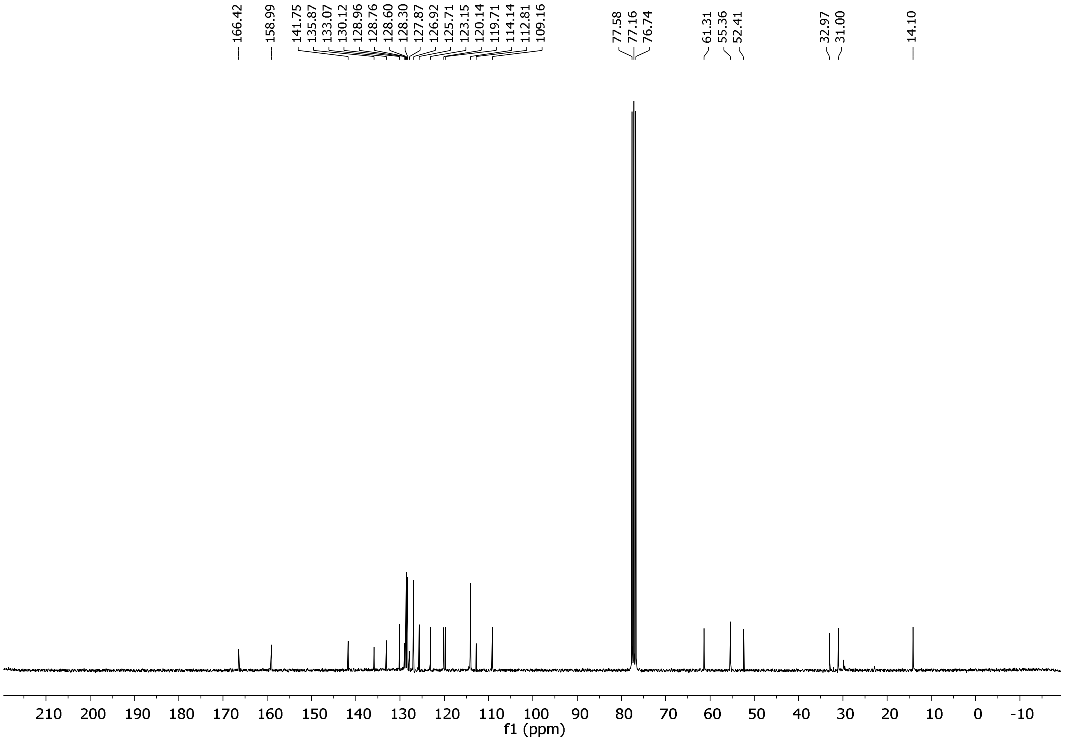

Figure S35. **^{1}H-NMR (300 MHz, $CDCl_3$) and ^{13}C-NMR (75 MHz, $CDCl_3$) spectra of compound 4j**

3k

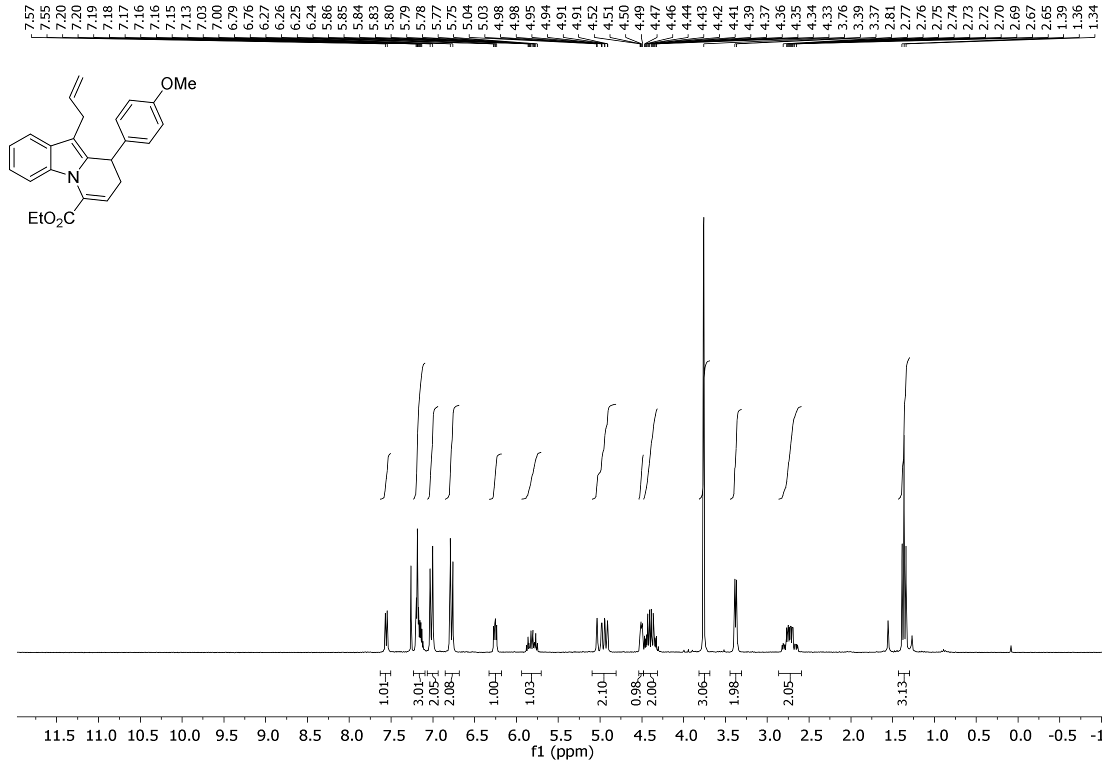

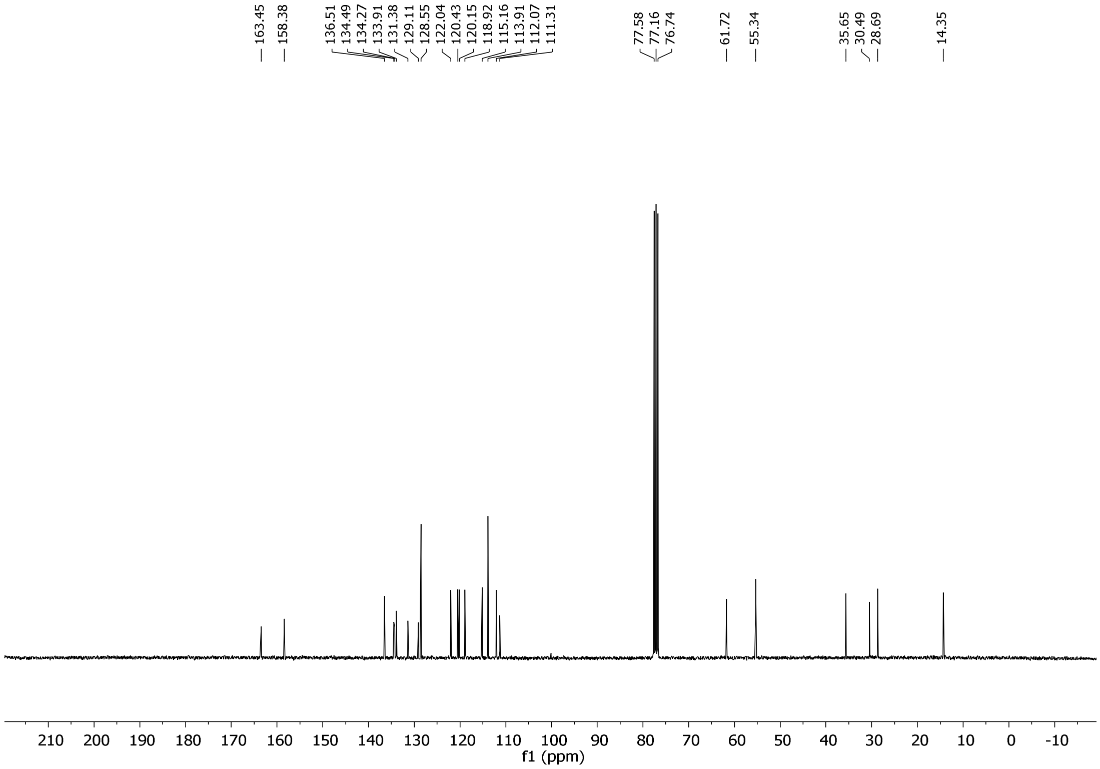

Figure S36. ^{1}H-NMR (300 MHz, $CDCl_3$) and ^{13}C-NMR (75 MHz, $CDCl_3$) spectra of compound 3k

4k

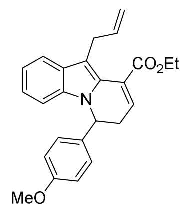

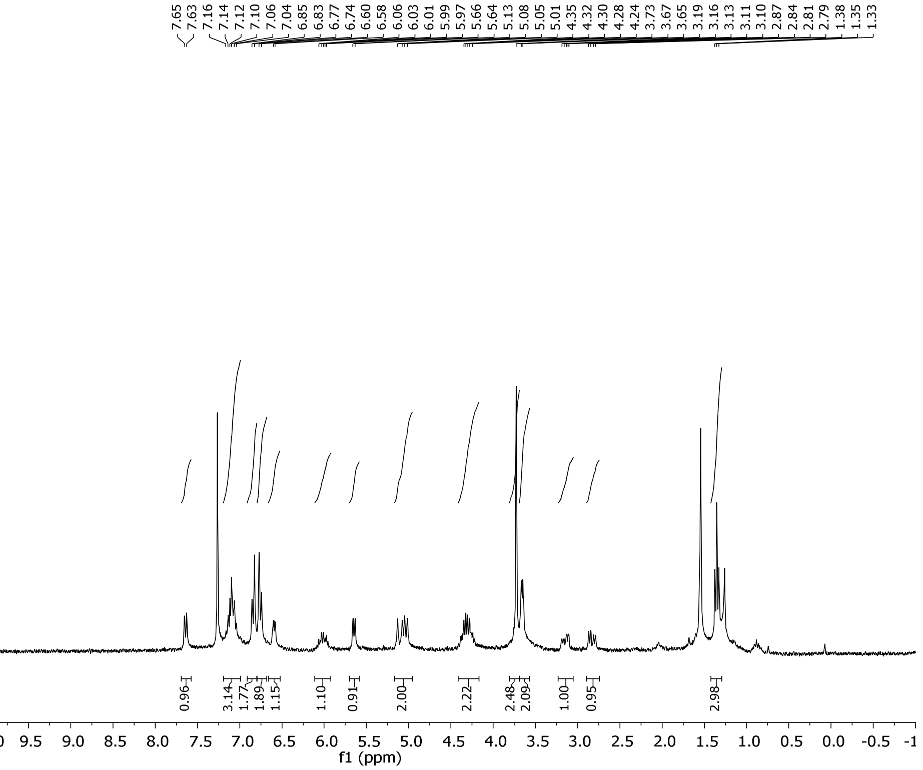

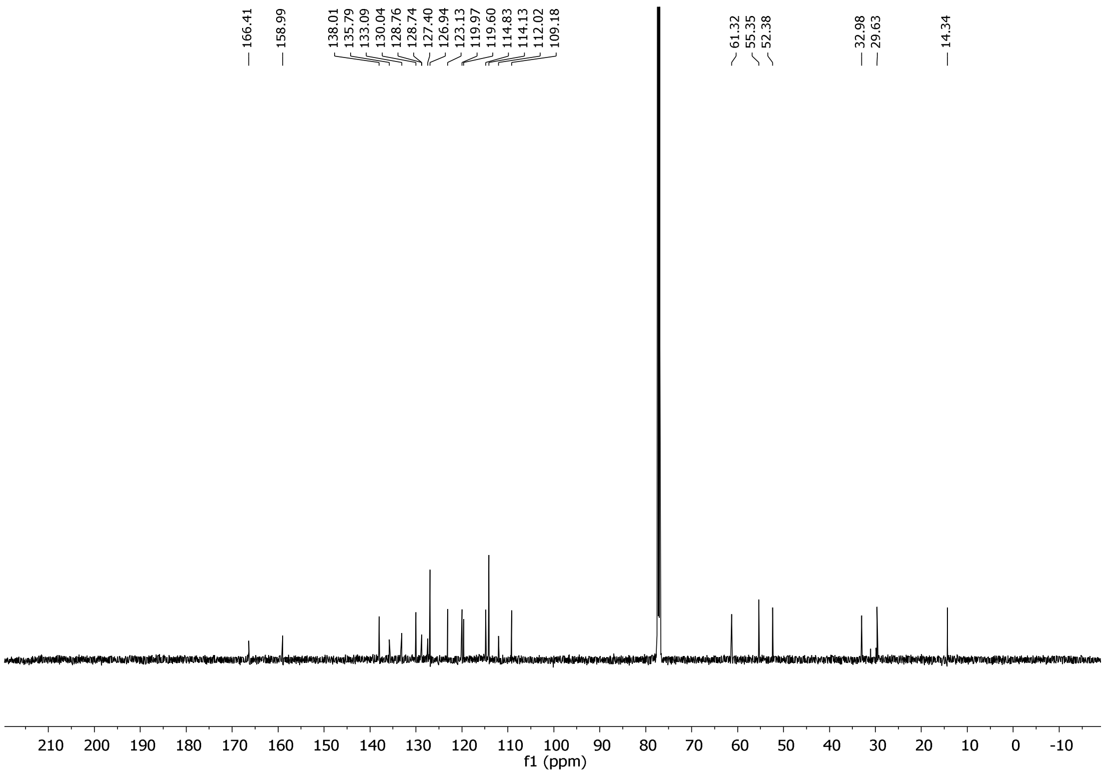

Figure S37. **^{1}H-NMR (300 MHz, $CDCl_3$) and ^{13}C-NMR (75 MHz, $CDCl_3$) spectra of compound 4k**

3l

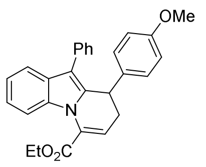

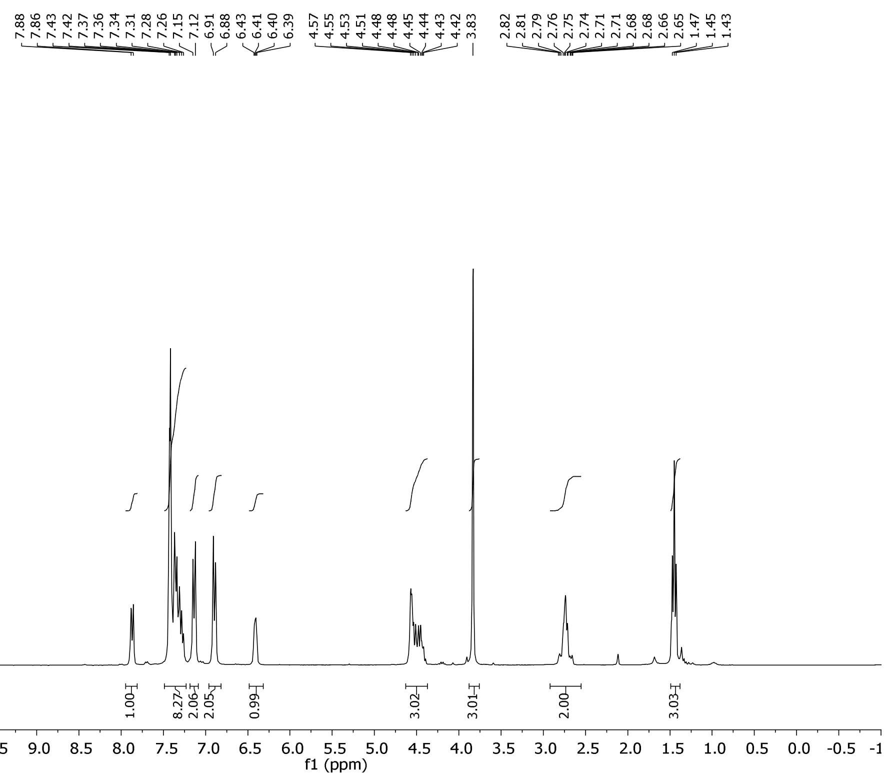

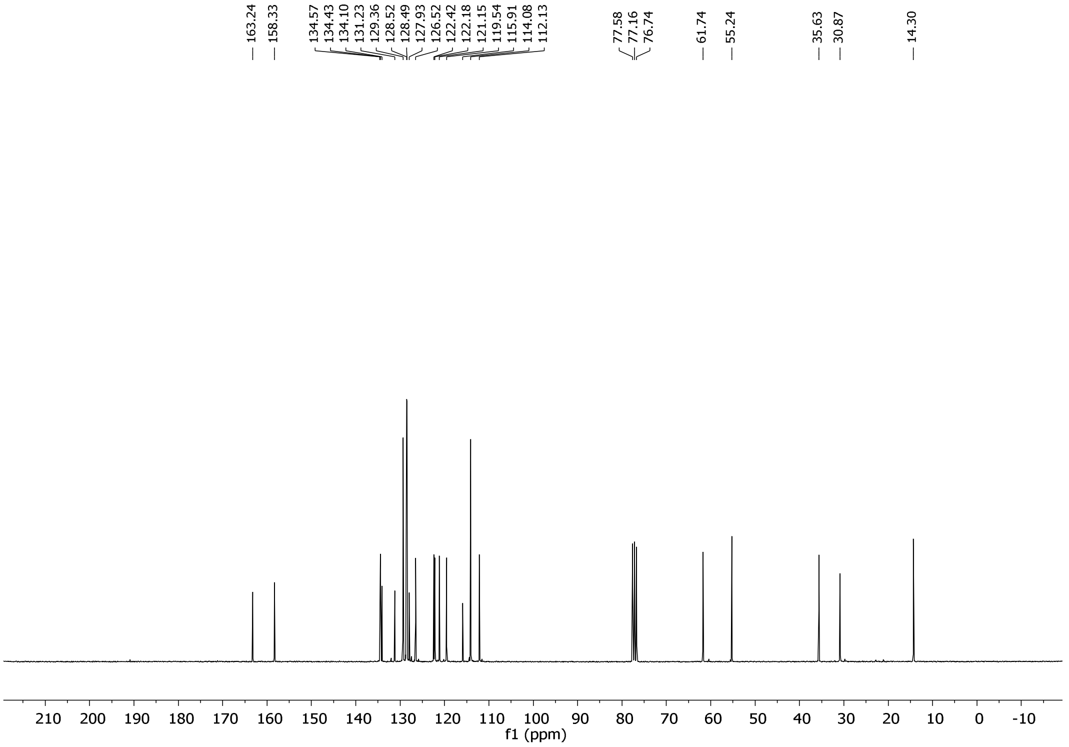

Figure S38. **^{1}H-NMR (300 MHz, $CDCl_3$) and ^{13}C-NMR (75 MHz, $CDCl_3$) spectra of compound 3l**

4l

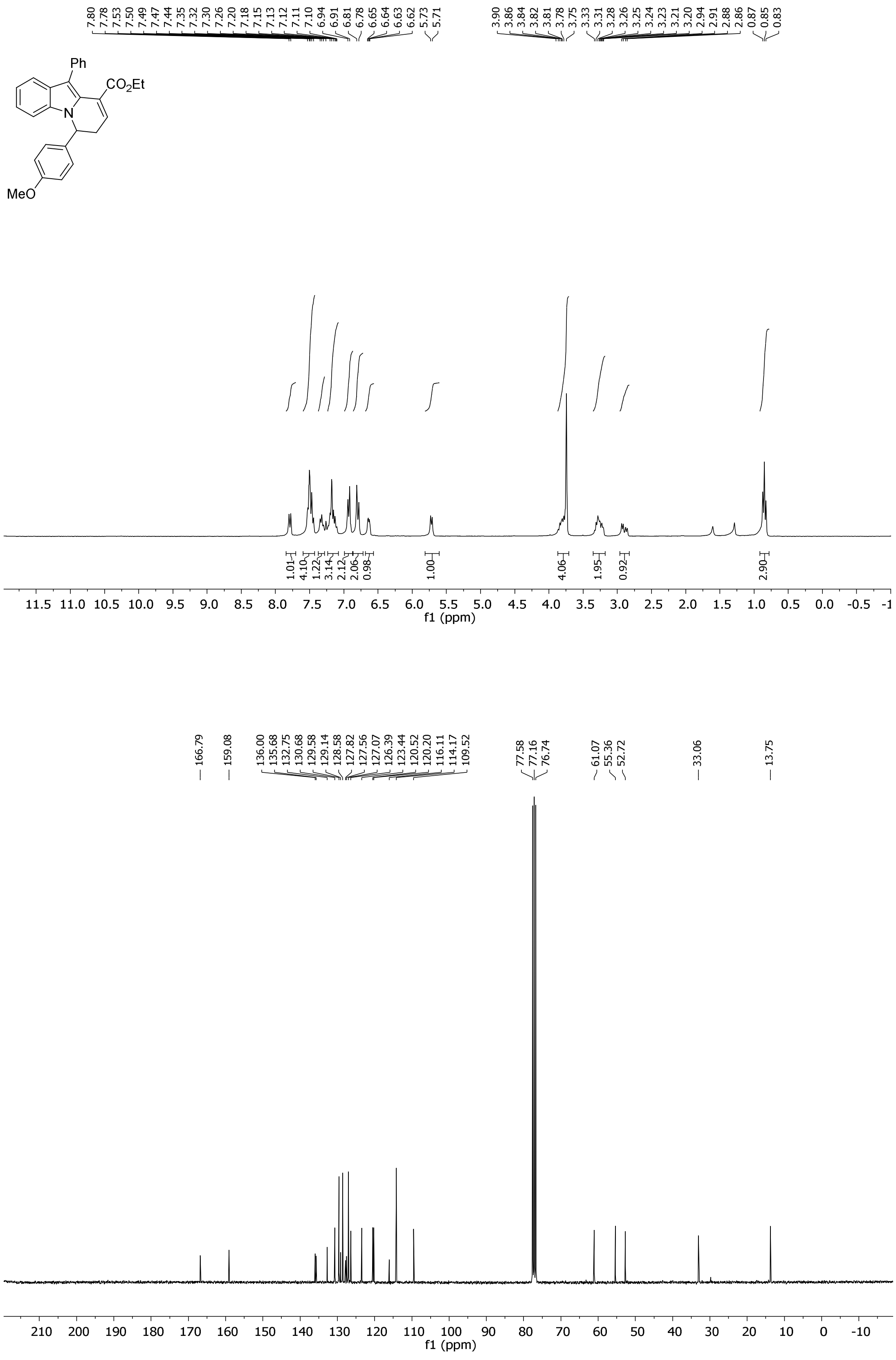

Figure S39. ^{1}H-NMR (300 MHz, $CDCl_3$) and ^{13}C-NMR (75 MHz, $CDCl_3$) **spectra of compound 4l**

3m

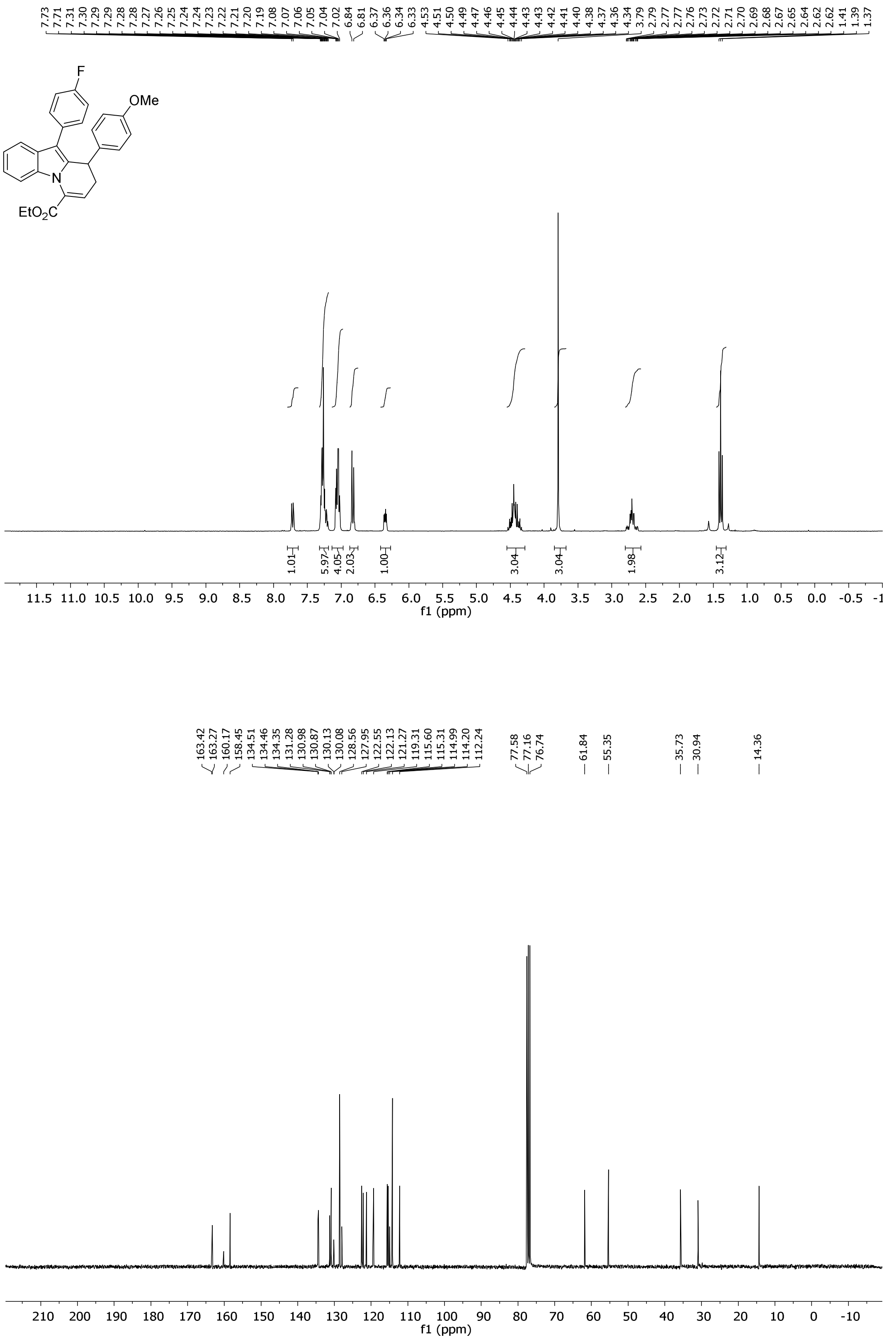

Figure S40. ^{1}H-NMR (300 MHz, $CDCl_3$) and ^{13}C-NMR (75 MHz, $CDCl_3$) spectra of compound 3m

3n

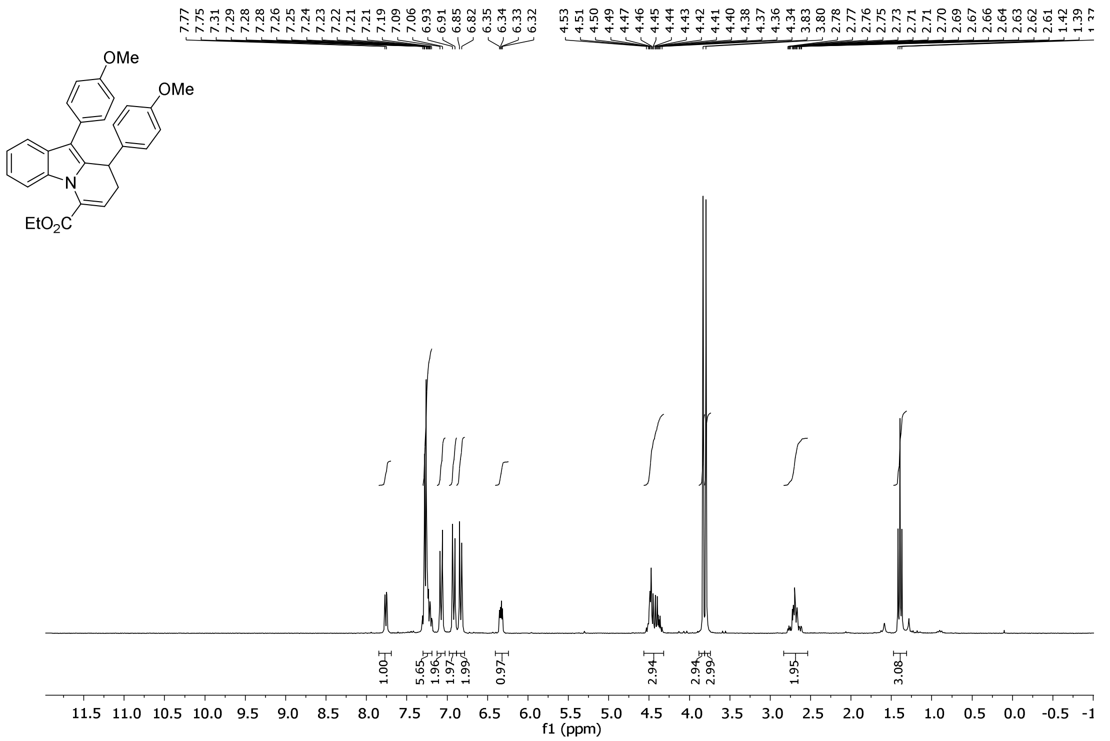

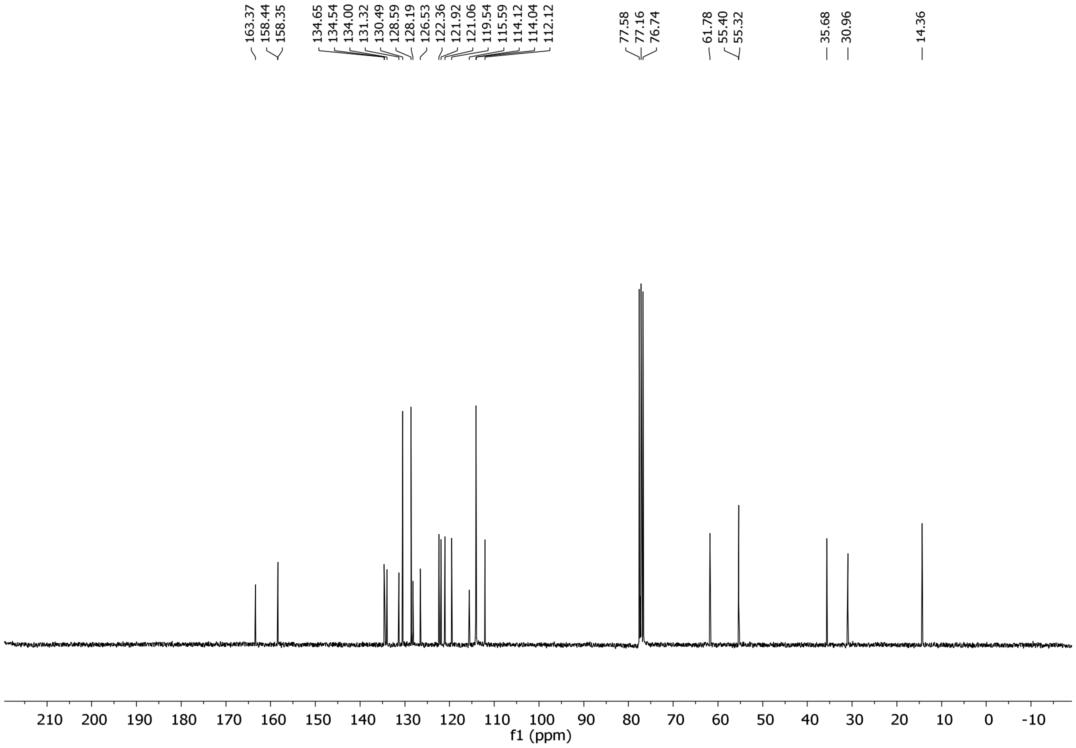

Figure S41. **^{1}H-NMR (300 MHz, $CDCl_3$) and ^{13}C-NMR (75 MHz, $CDCl_3$) spectra of compound 3n**

4.4 Dihydropyridoindoles 5a-s

5a

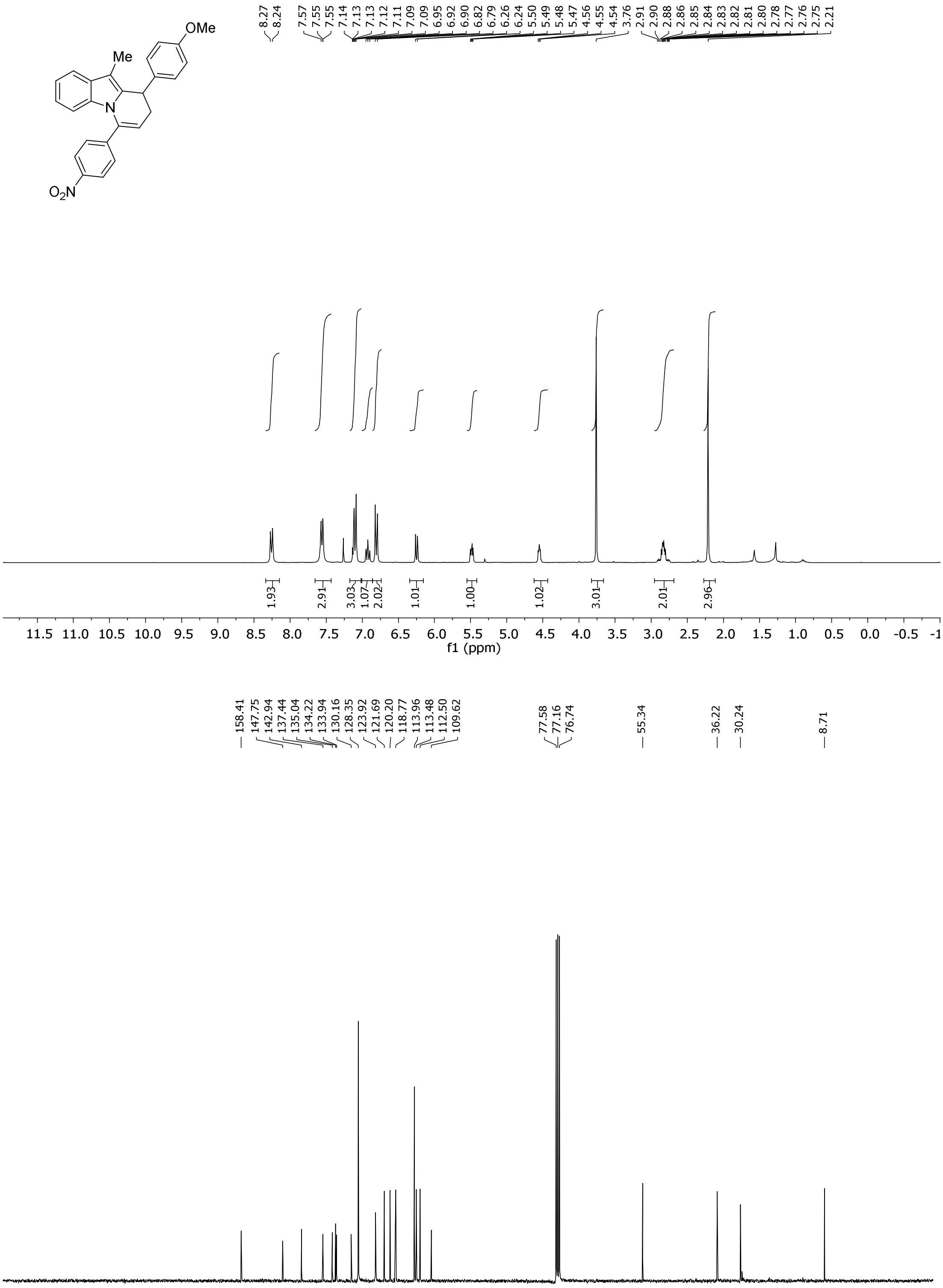

Figure S42. **^{1}H-NMR (300 MHz, $CDCl_3$) and ^{13}C-NMR (75 MHz, $CDCl_3$) spectra of compound 5a**

5b

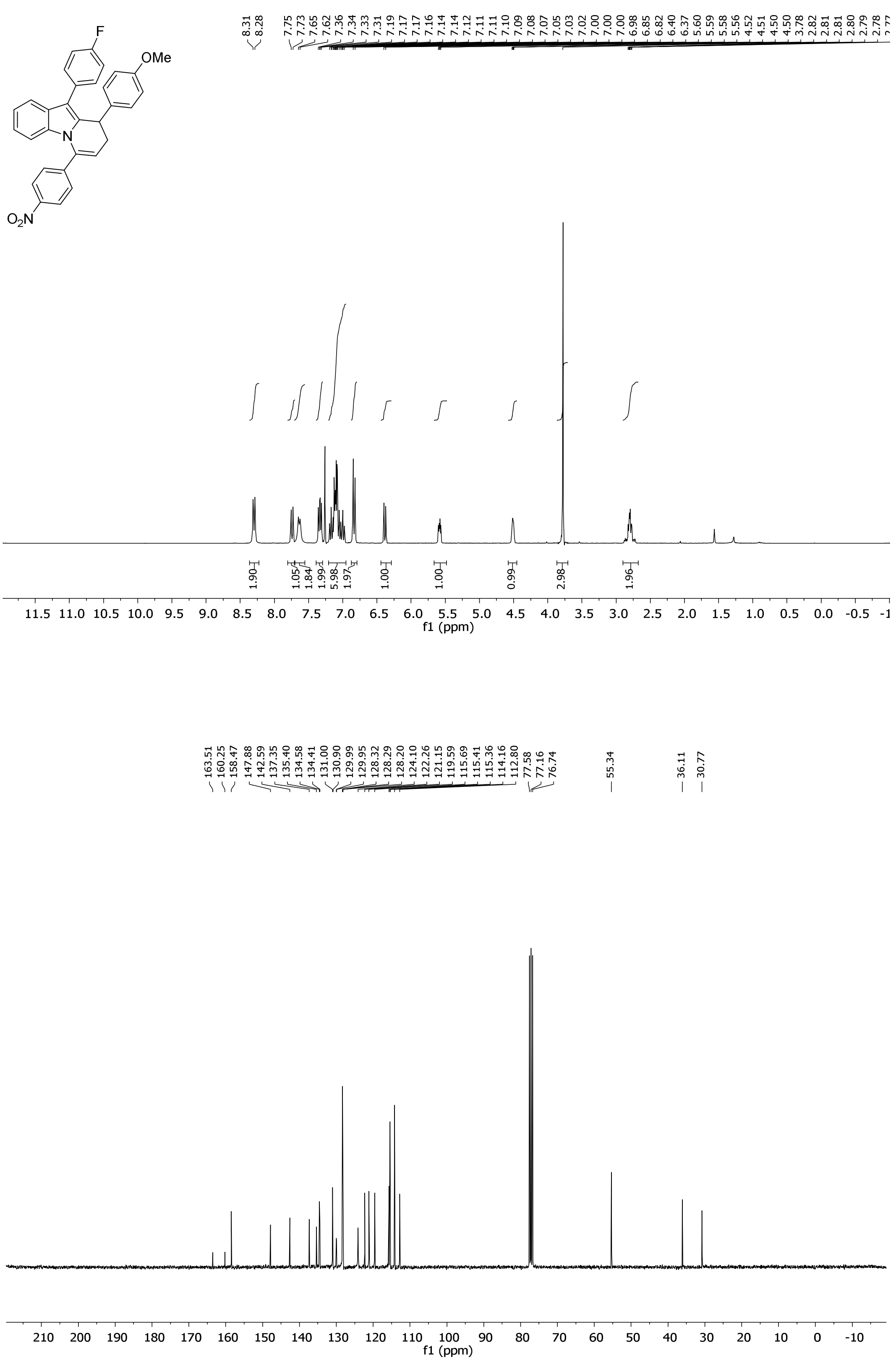

Figure S43. **^{1}H-NMR (300 MHz, $CDCl_3$) and ^{13}C-NMR (75 MHz, $CDCl_3$) spectra of compound 5b**

5c

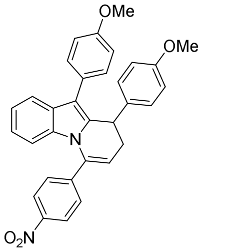

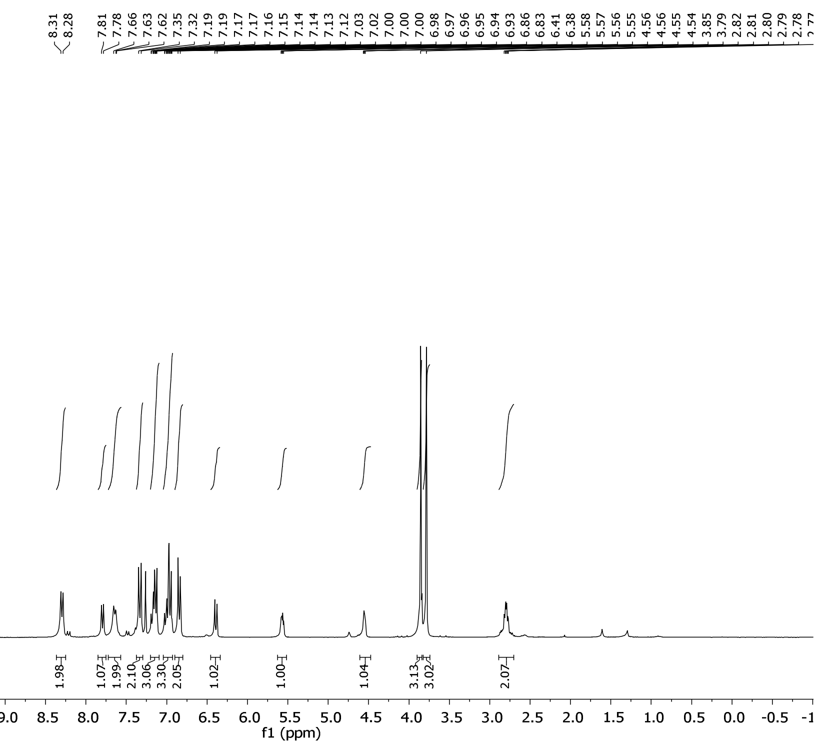

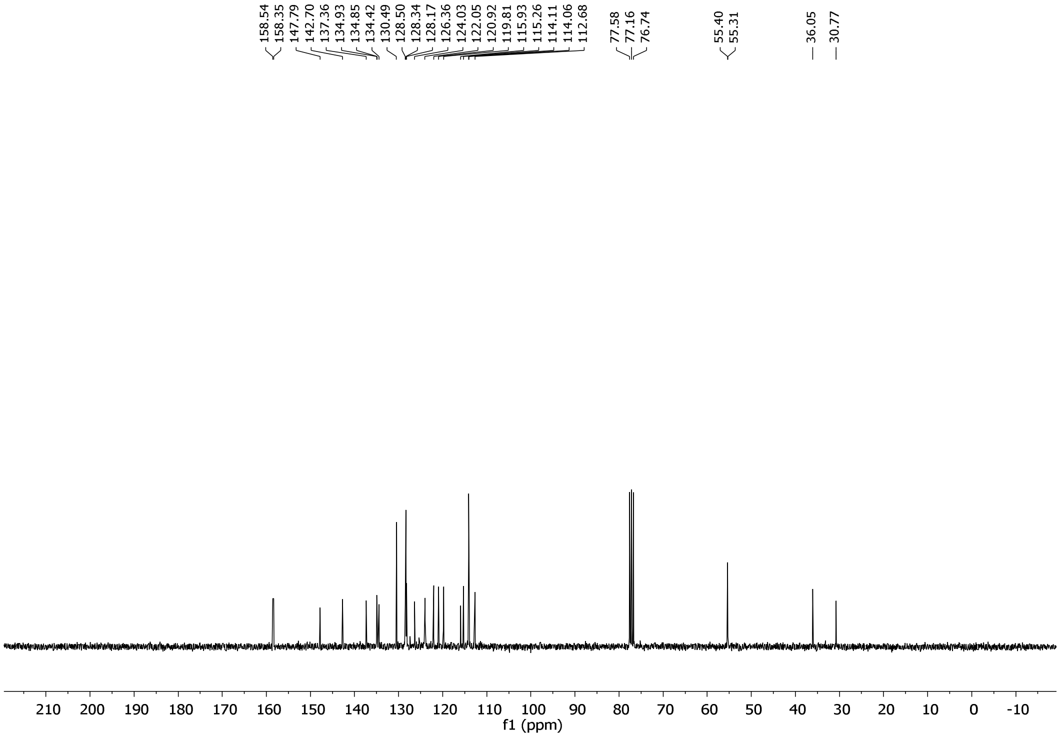

Figure S44. ^{1}H-NMR (300 MHz, $CDCl_3$) and ^{13}C-NMR (75 MHz, $CDCl_3$) spectra of compound 5c

5d

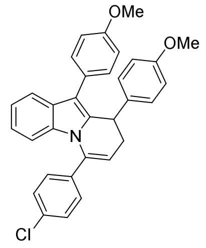

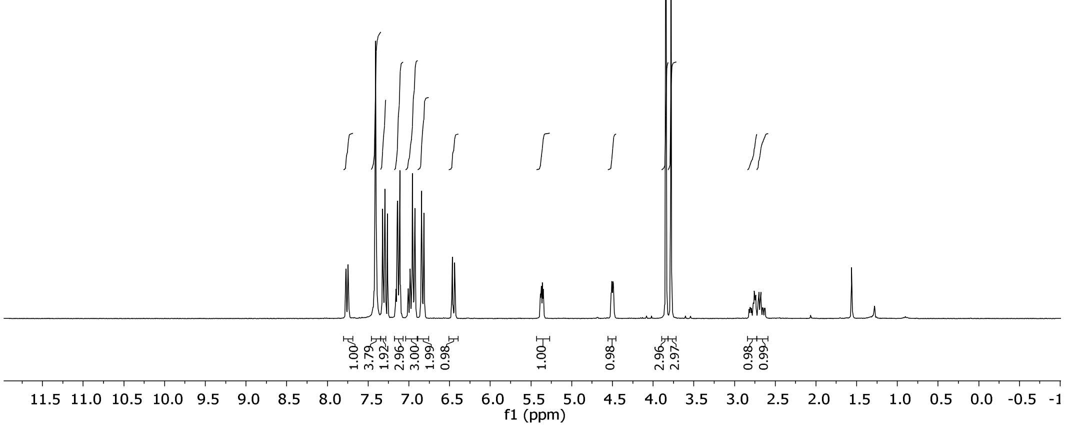

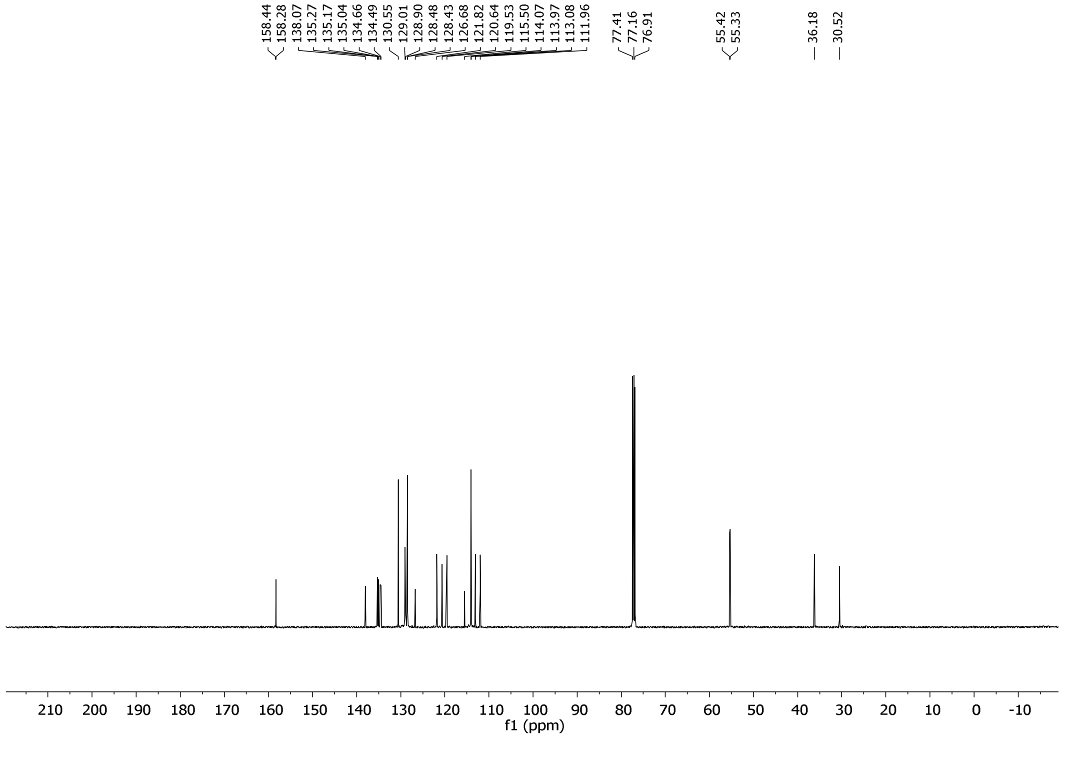

Figure S45. ^{1}**H-NMR (300 MHz, CDCl$_3$) and** ^{13}**C-NMR (75 MHz, CDCl$_3$) spectra of compound 5d**

5e

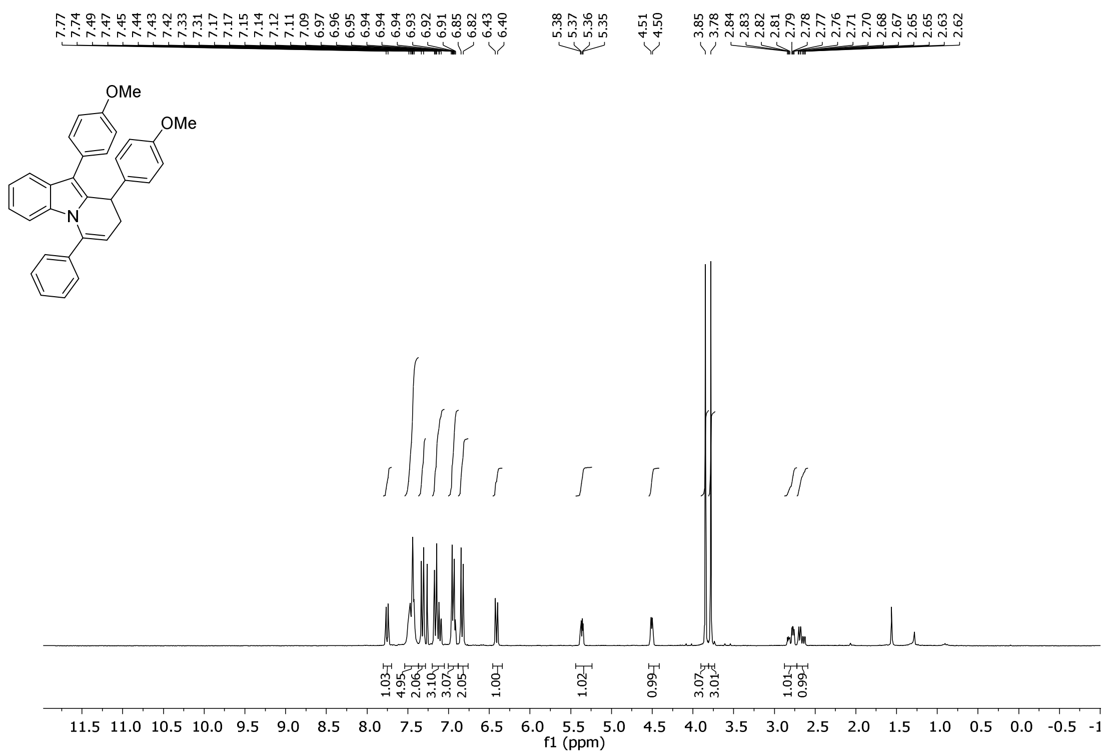

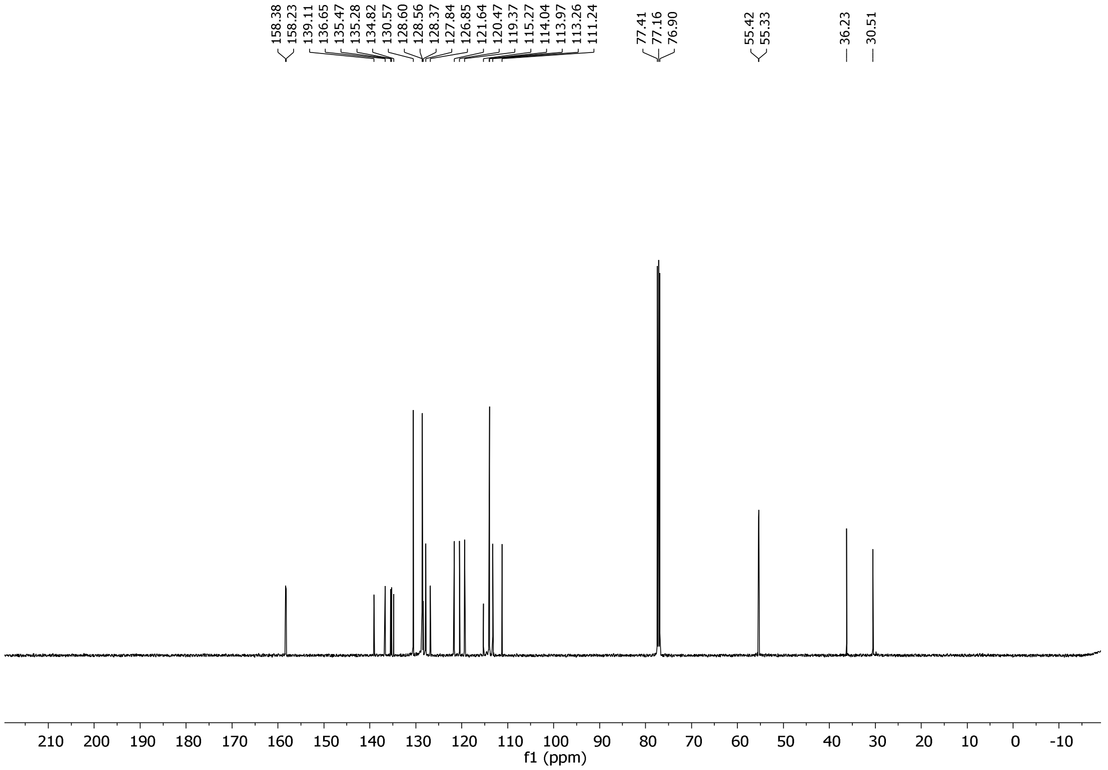

Figure S46. ^{1}**H-NMR (300 MHz,** $CDCl_3$**) and** ^{13}**C-NMR (75 MHz,** $CDCl_3$**) spectra of compound 5e**

5f

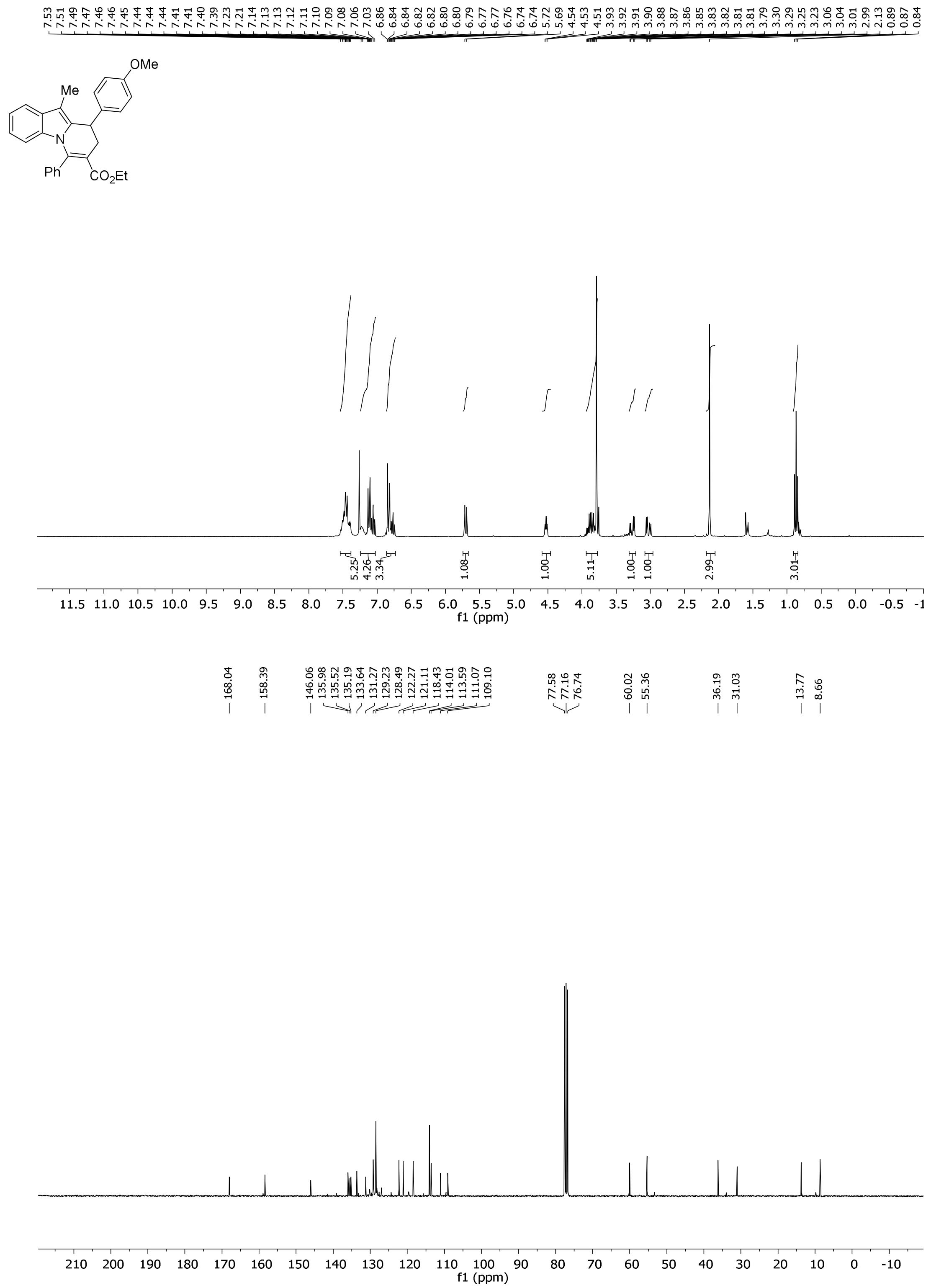

Figure S47. ^{1}H-NMR (300 MHz, $CDCl_3$) and ^{13}C-NMR (75 MHz, $CDCl_3$) spectra of compound 5f

5g

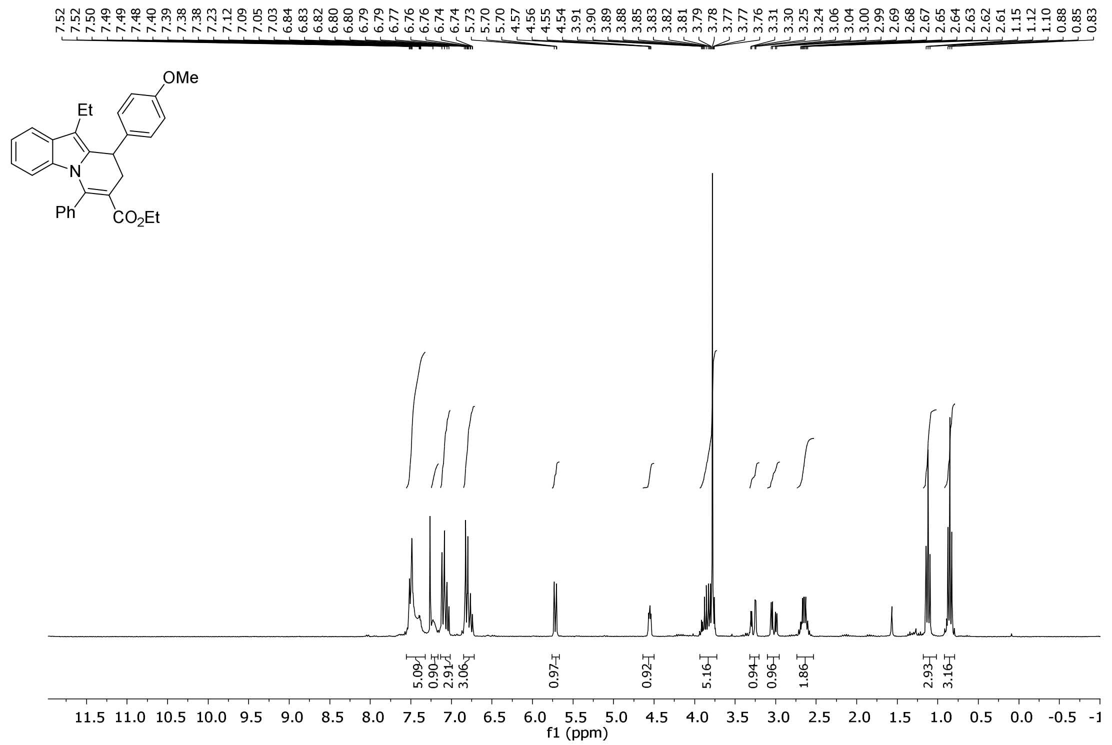

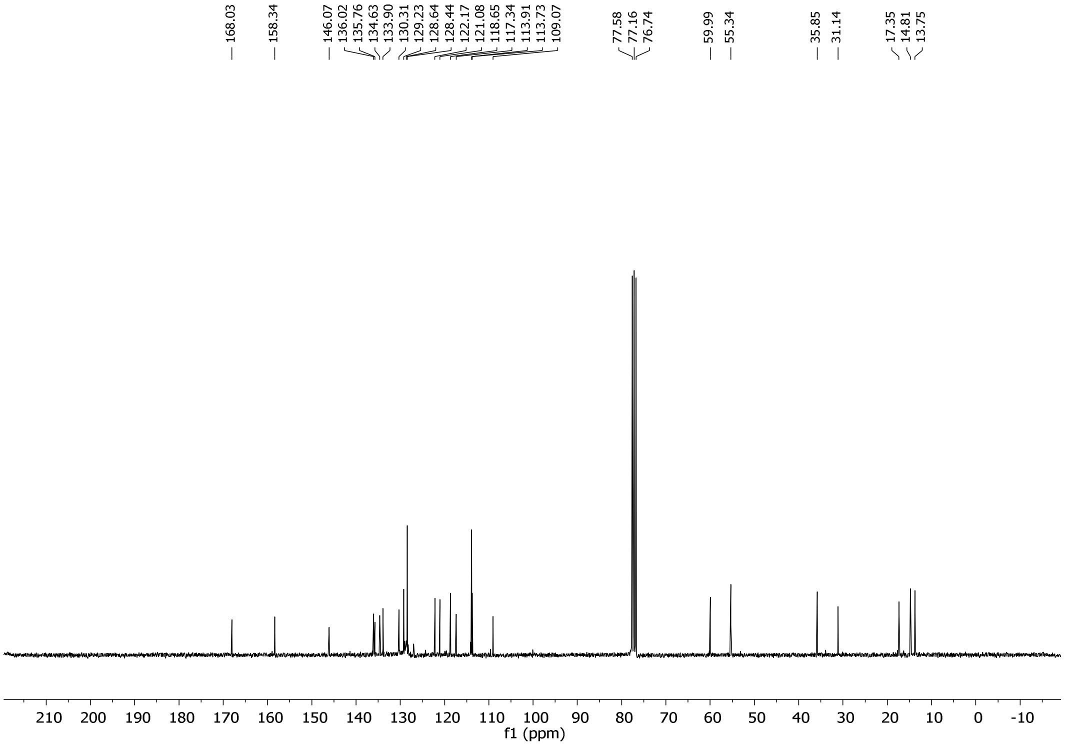

Figure S48. ^{1}**H-NMR (300 MHz,** $CDCl_3$**) and** ^{13}**C-NMR (75 MHz,** $CDCl_3$**) spectra of compound 5g**

5h

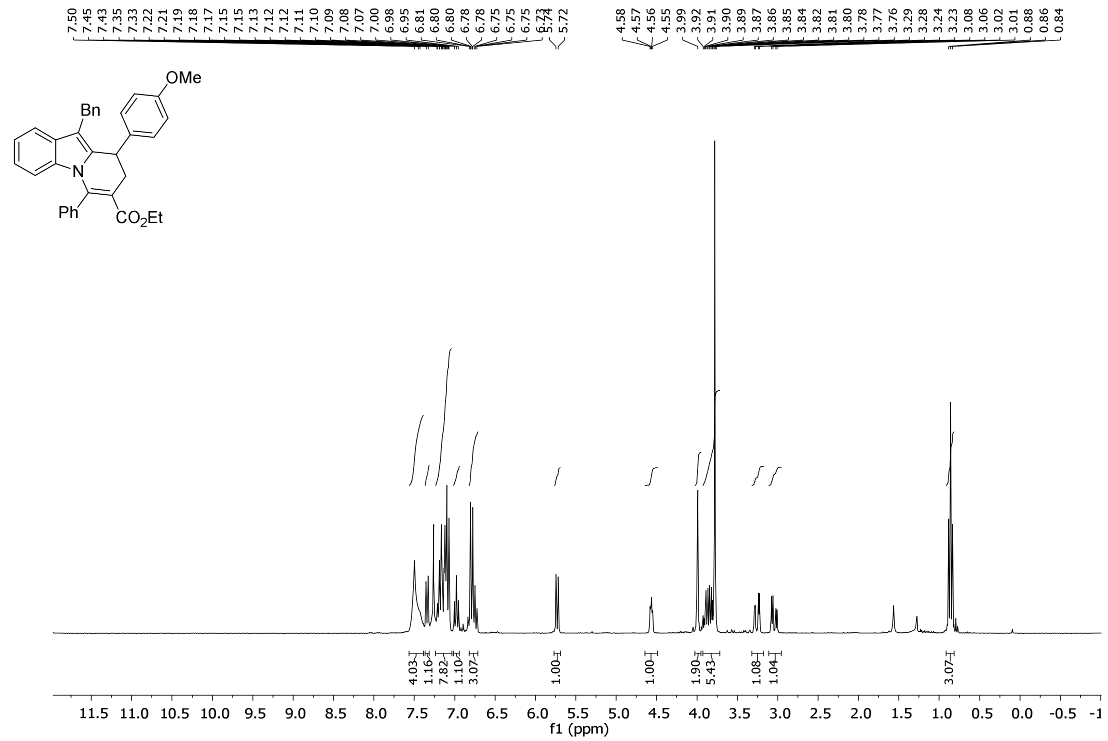

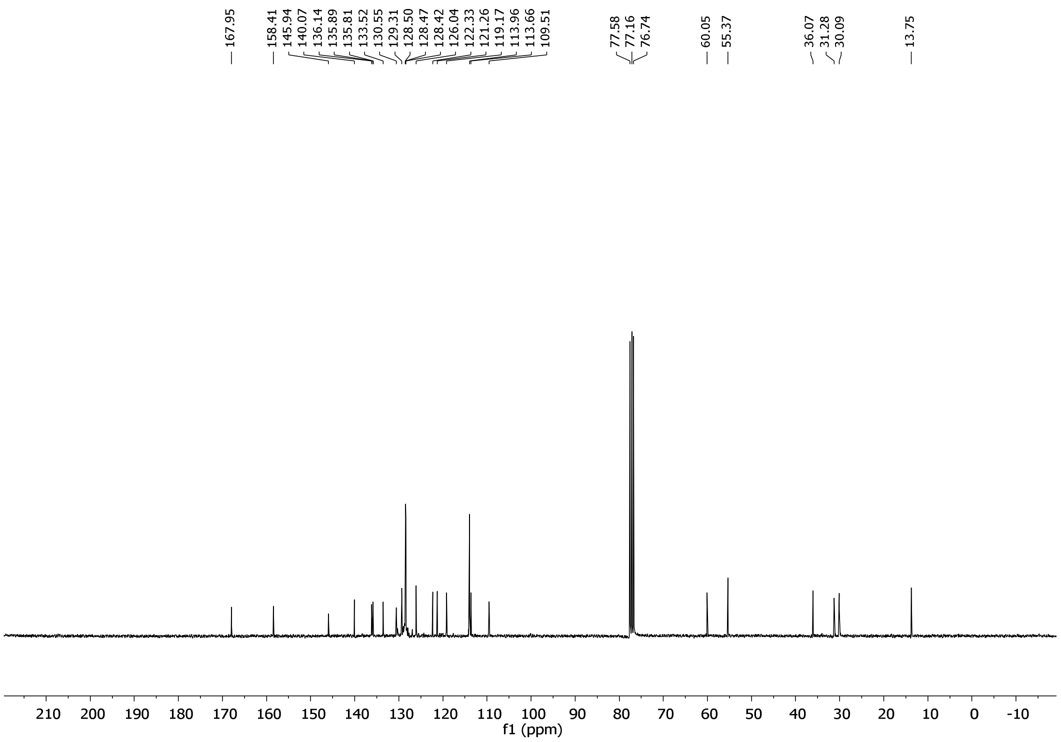

Figure S49. ^{1}H-NMR (300 MHz, $CDCl_3$) and ^{13}C-NMR (75 MHz, $CDCl_3$) spectra of compound 5h

5i

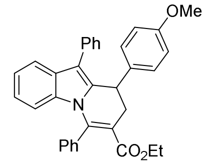

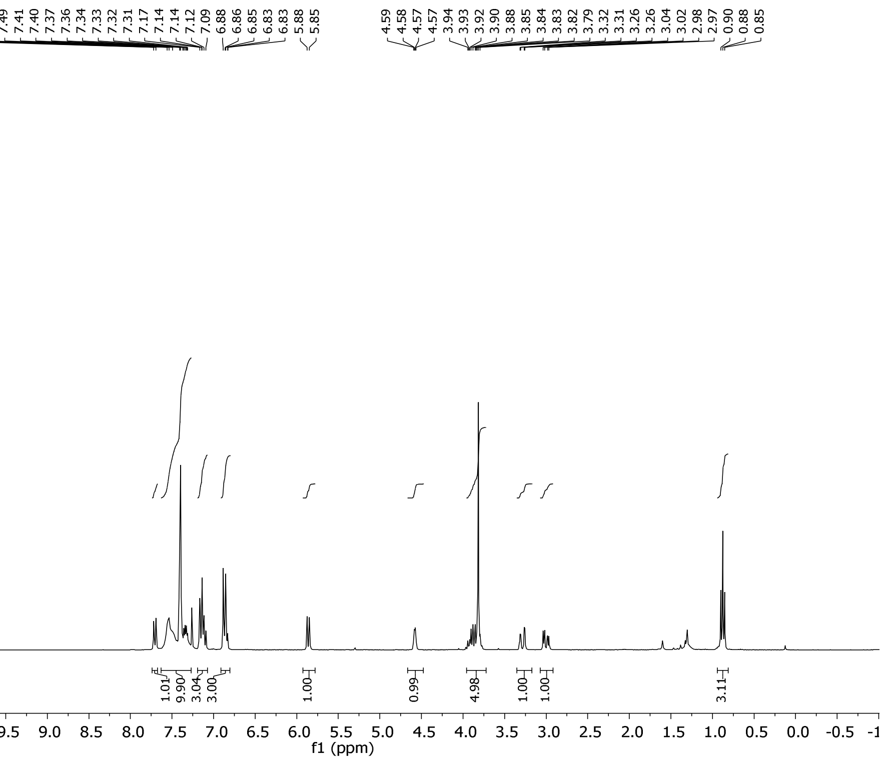

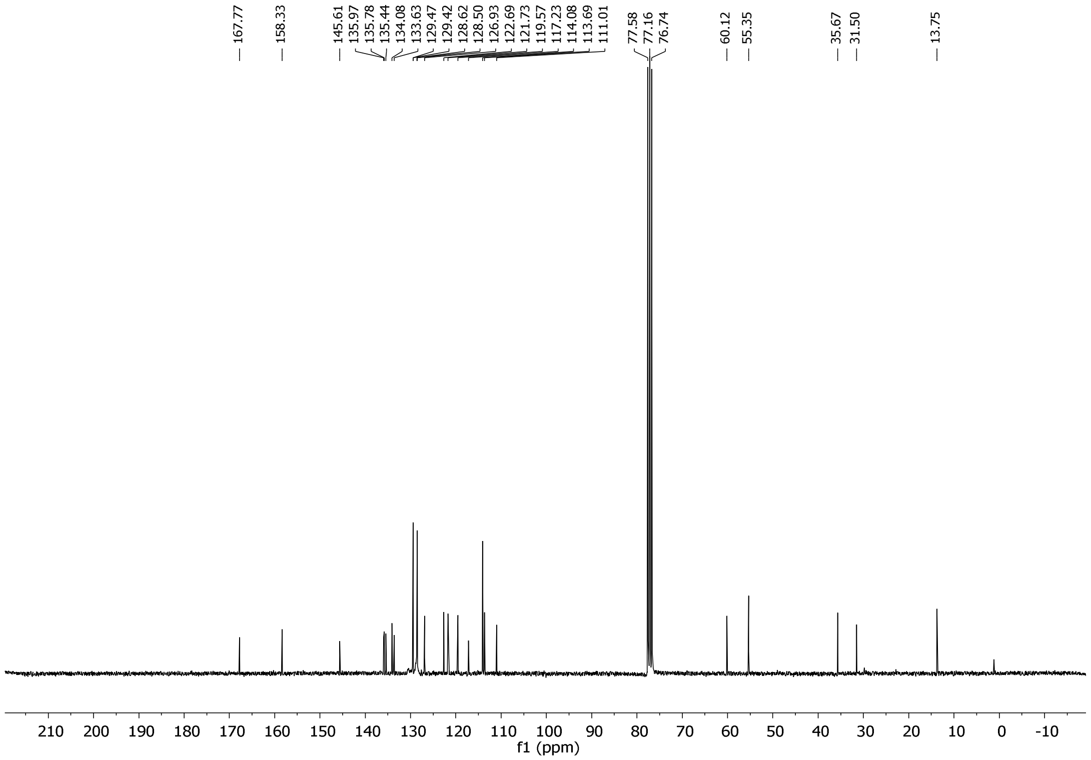

Figure S50. **^{1}H-NMR (300 MHz, $CDCl_3$) and ^{13}C-NMR (75 MHz, $CDCl_3$) spectra of compound 5i**

5j

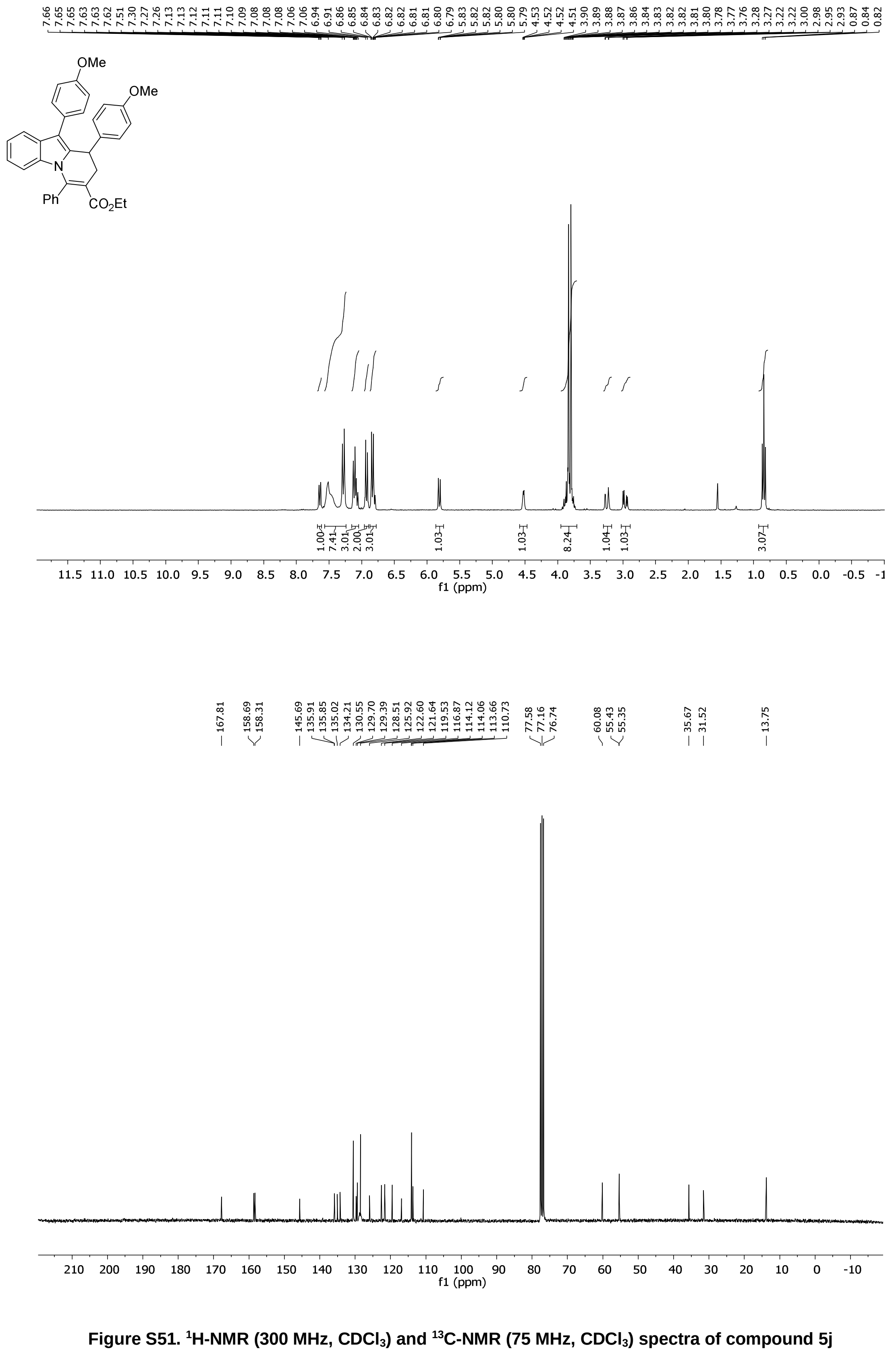

Figure S51. ^{1}H-NMR (300 MHz, $CDCl_3$) and ^{13}C-NMR (75 MHz, $CDCl_3$) spectra of compound 5j

5k

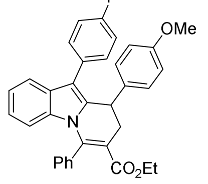

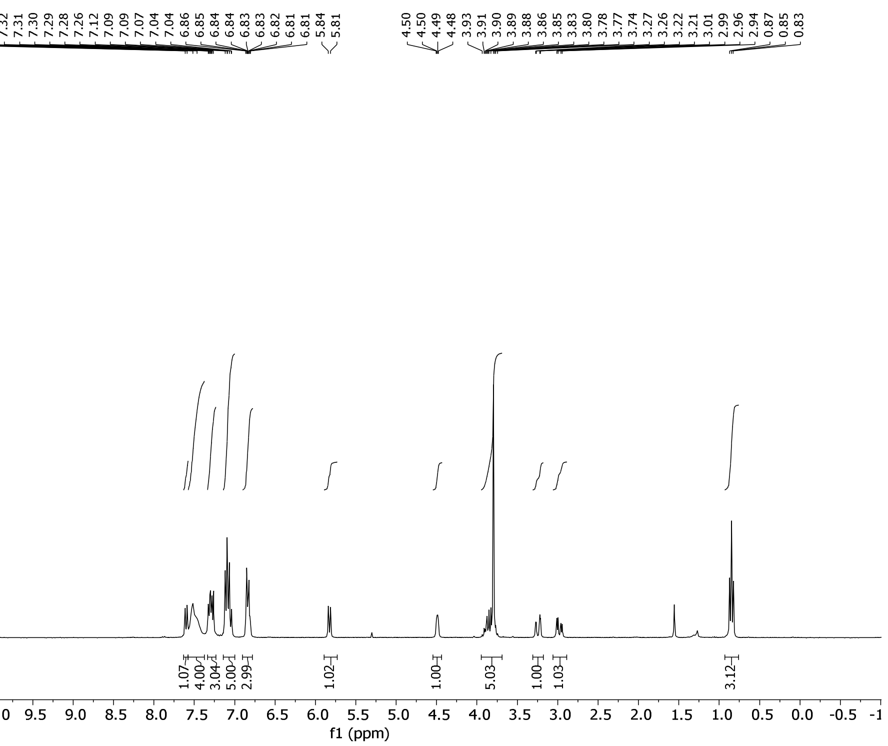

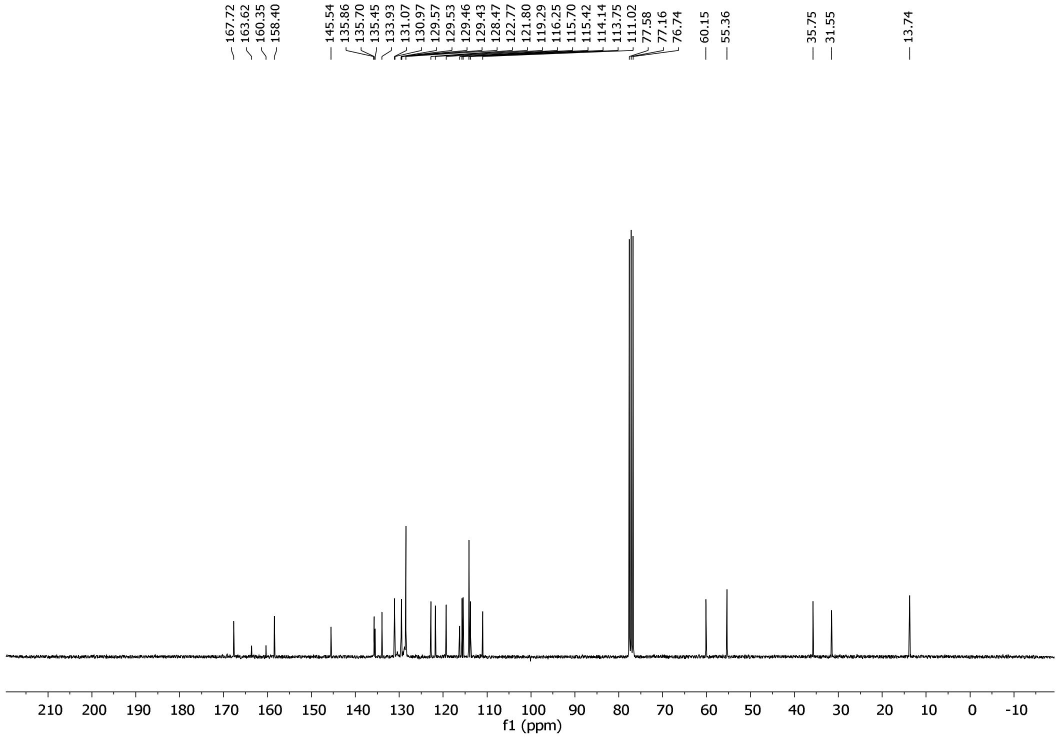

Figure S52. **^{1}H-NMR (300 MHz, $CDCl_3$) and ^{13}C-NMR (75 MHz, $CDCl_3$) spectra of compound 5k**

5l

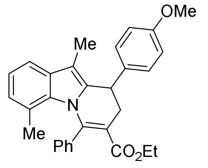

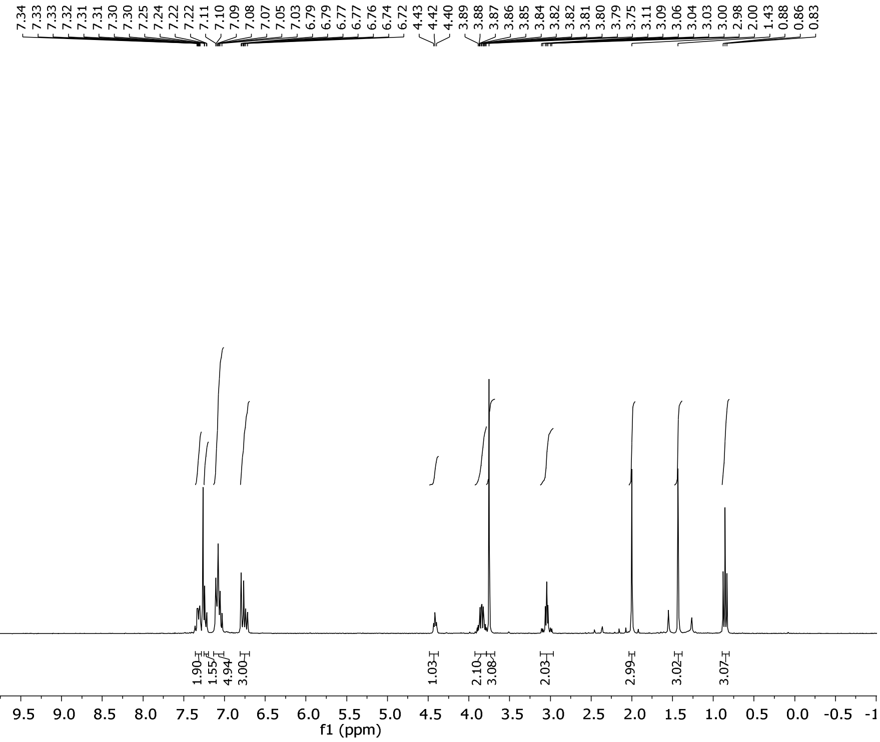

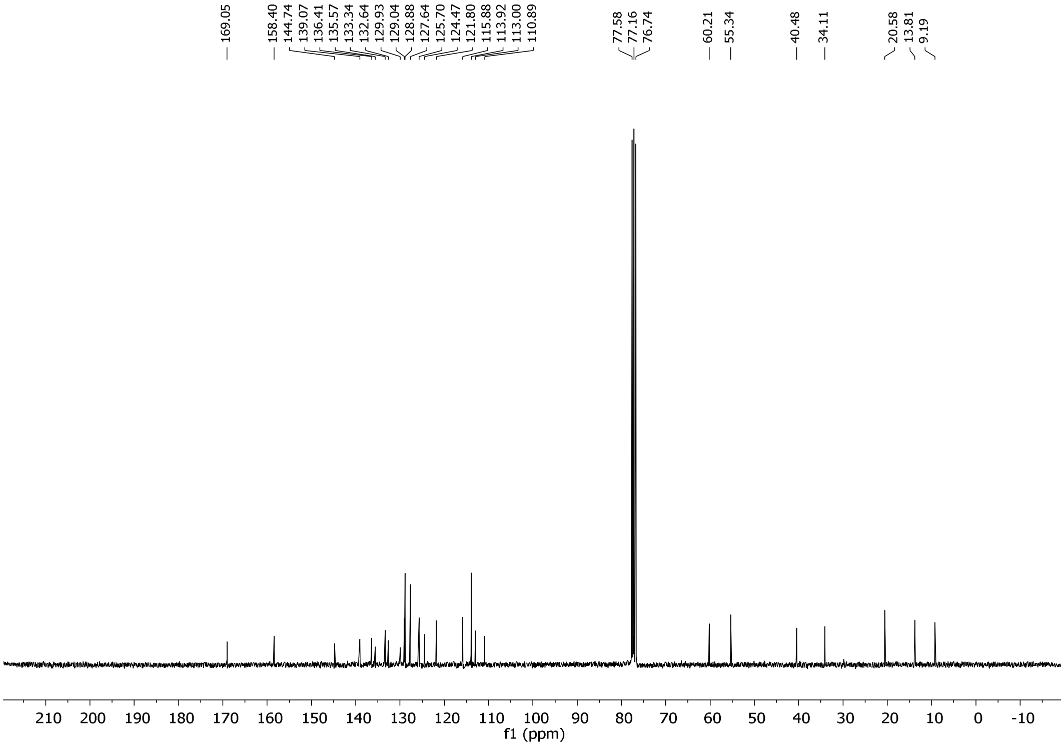

Figure S53. ^{1}H-NMR (300 MHz, $CDCl_3$) and ^{13}C-NMR (75 MHz, $CDCl_3$) spectra of compound **5l**

5m

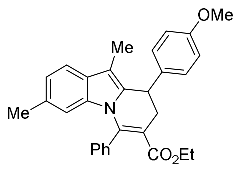

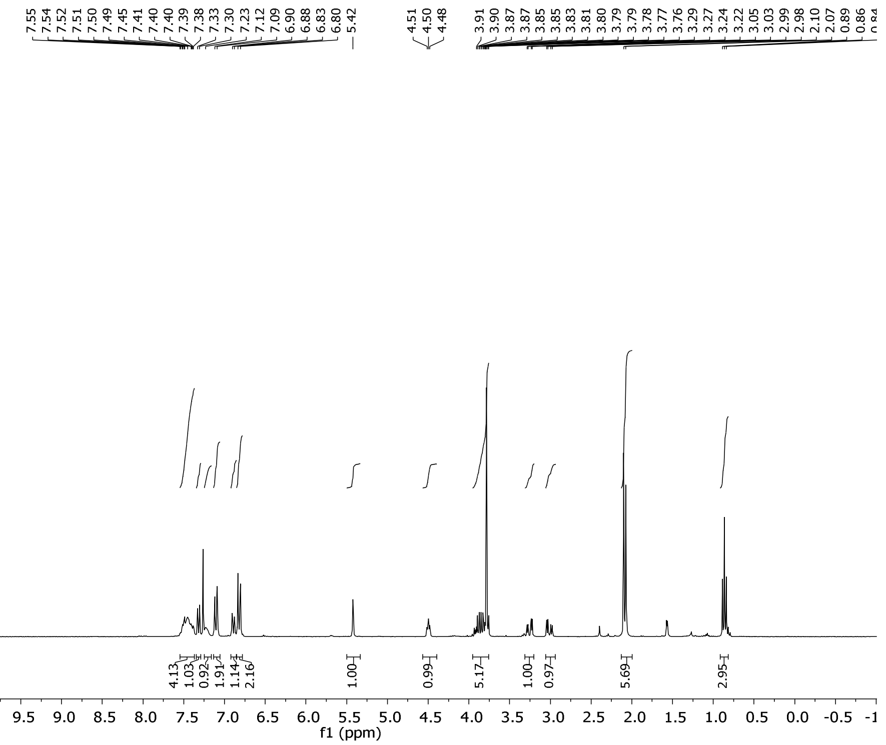

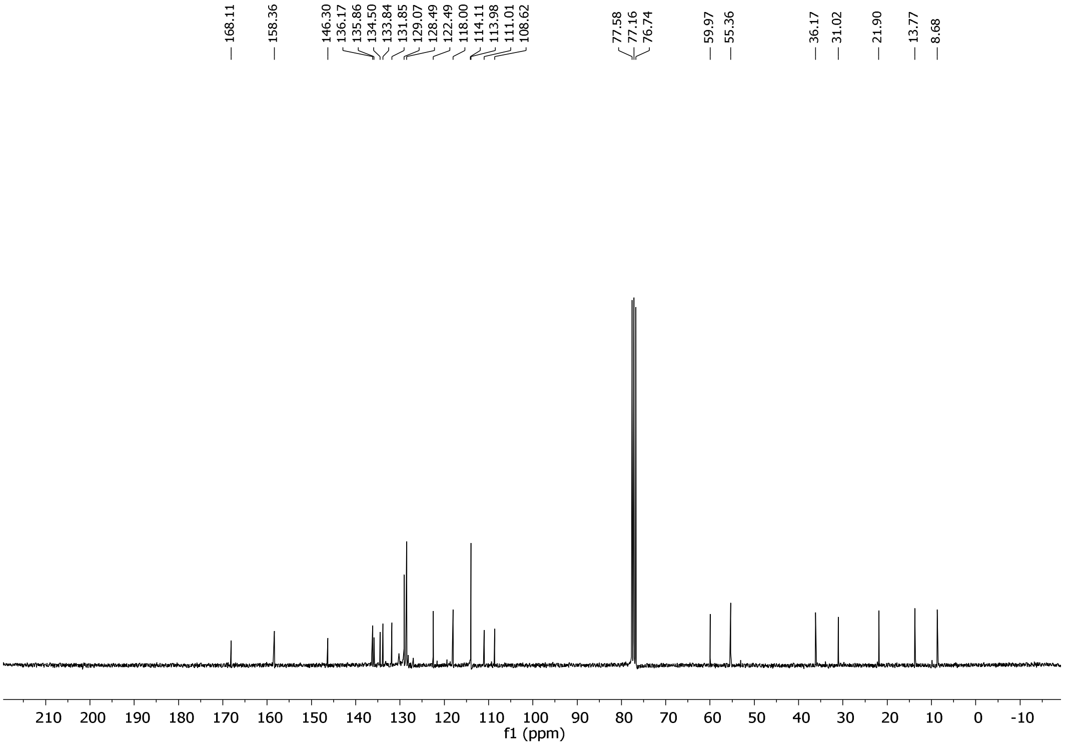

Figure S54. [1]**H-NMR (300 MHz, $CDCl_3$) and** [13]**C-NMR (75 MHz, $CDCl_3$) spectra of compound 5m**

5n

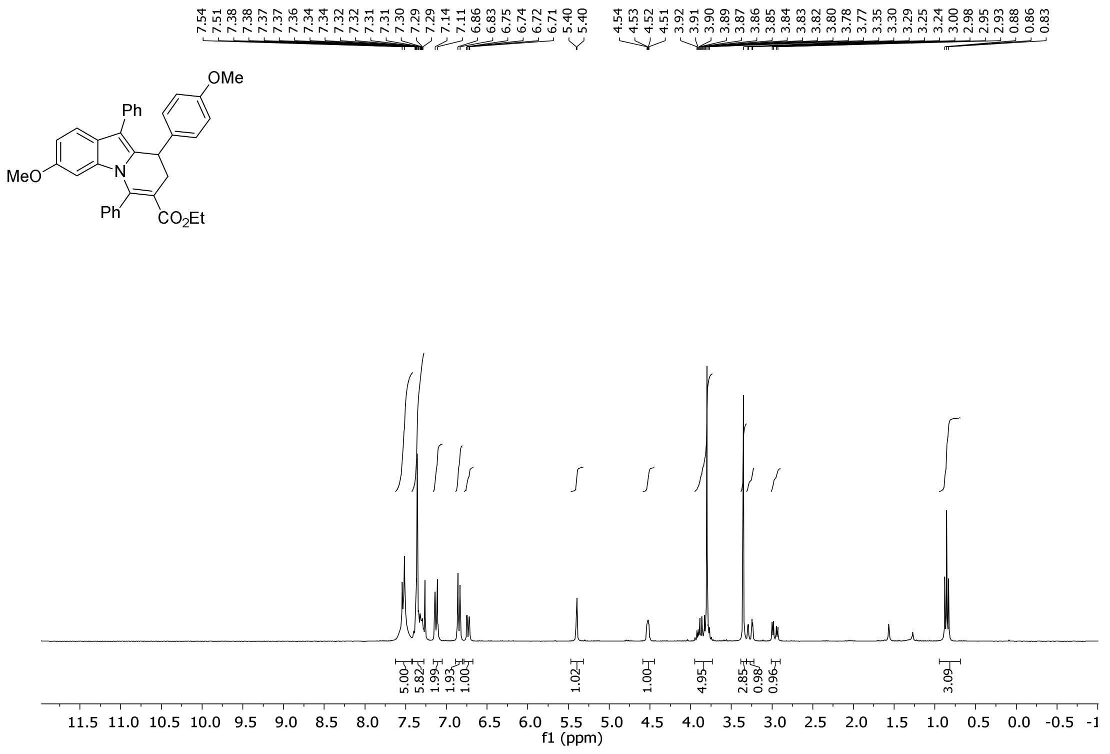

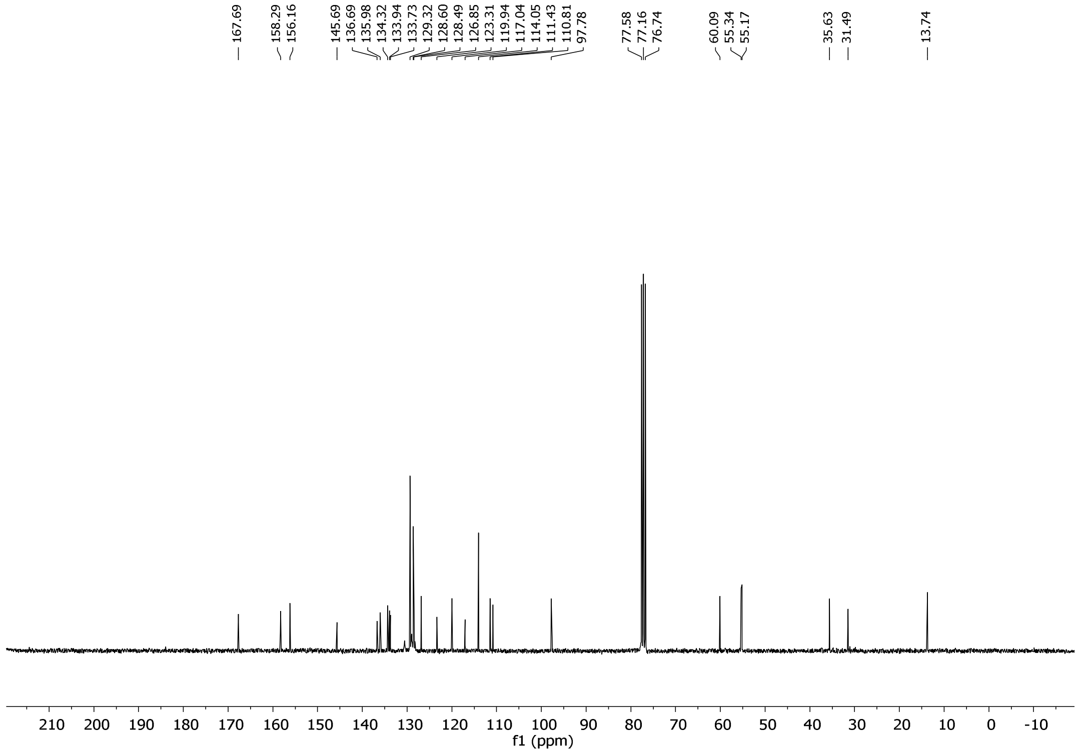

Figure S55. ^{1}H-NMR (300 MHz, $CDCl_3$) and ^{13}C-NMR (75 MHz, $CDCl_3$) spectra of compound 5n

5o

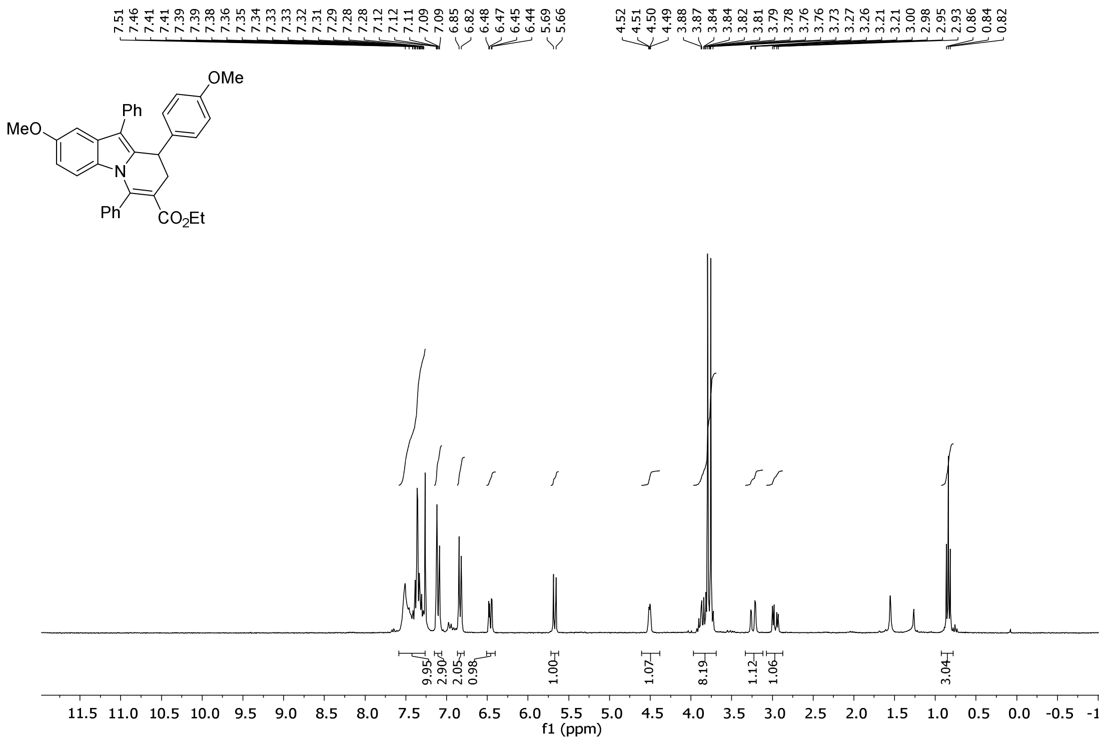

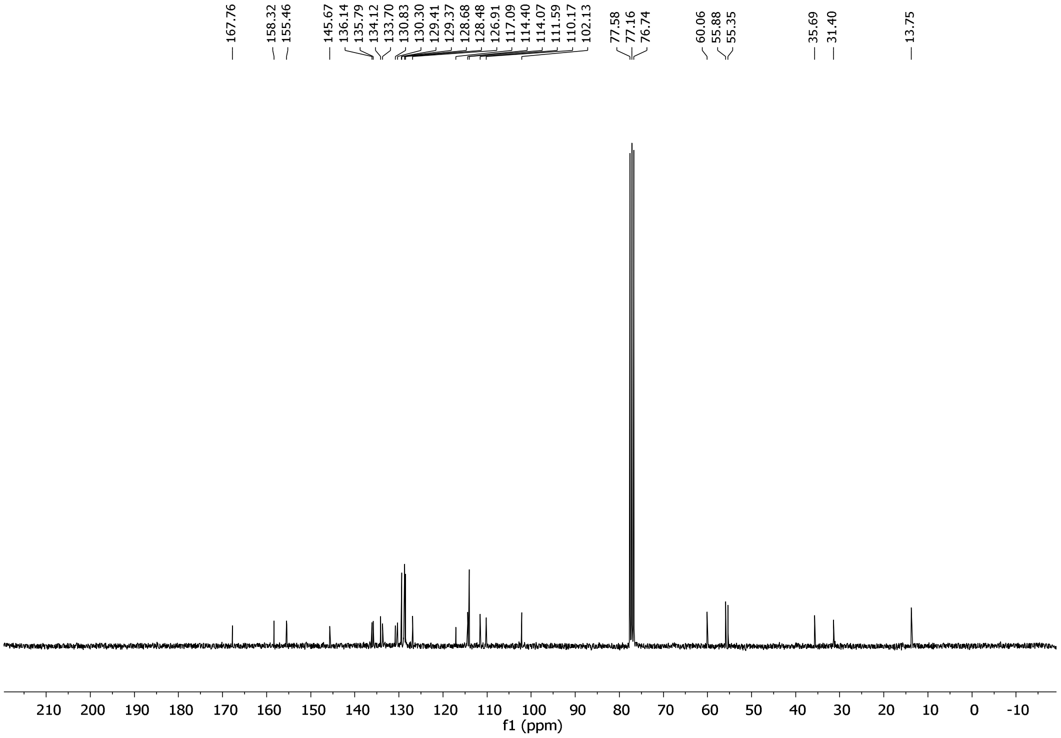

Figure S56. ^1H**-NMR (300 MHz,** $CDCl_3$**) and** ^{13}C**-NMR (75 MHz,** $CDCl_3$**) spectra of compound 5o**

5p

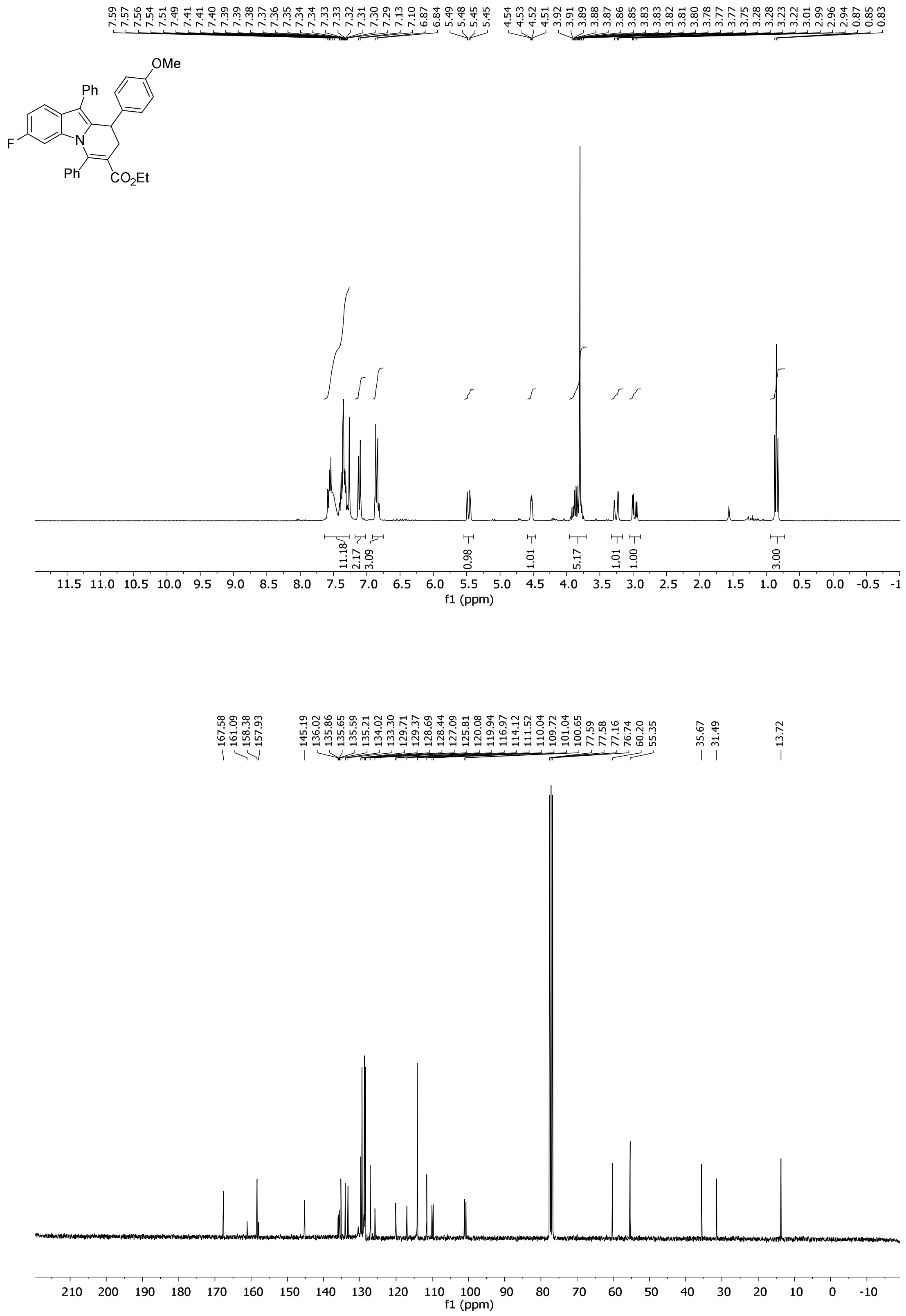

Figure S57. **^{1}H-NMR (300 MHz, $CDCl_3$) and ^{13}C-NMR (75 MHz, $CDCl_3$) spectra of compound 5p**

5q

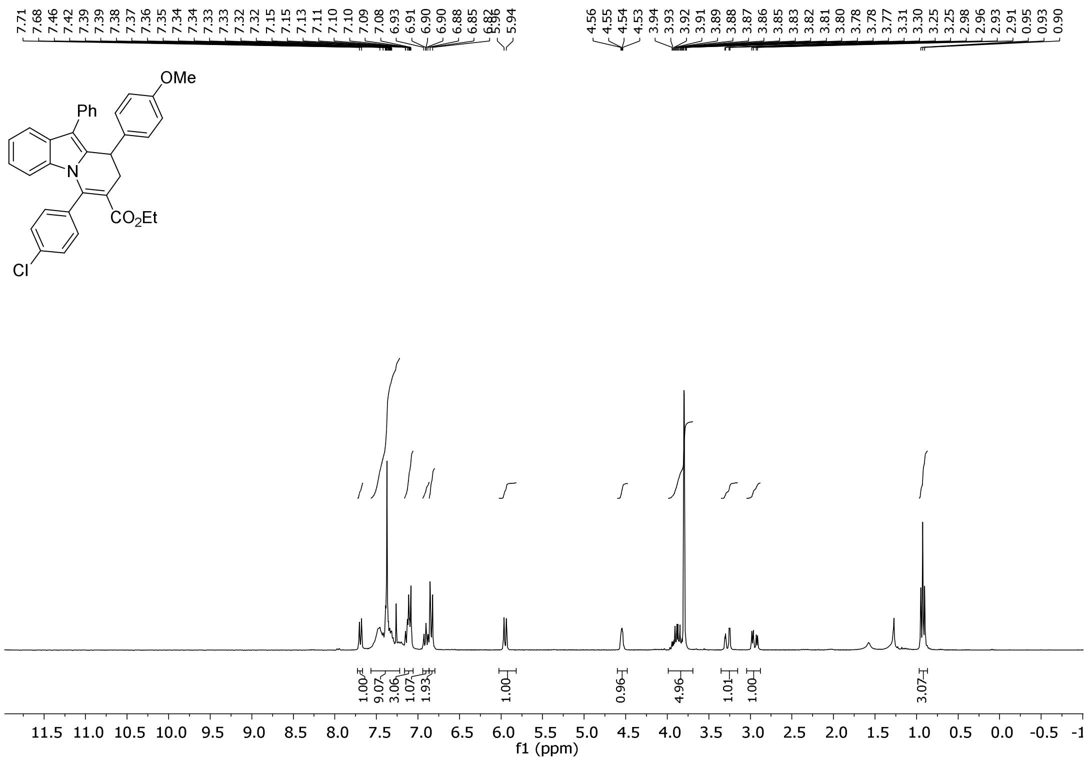

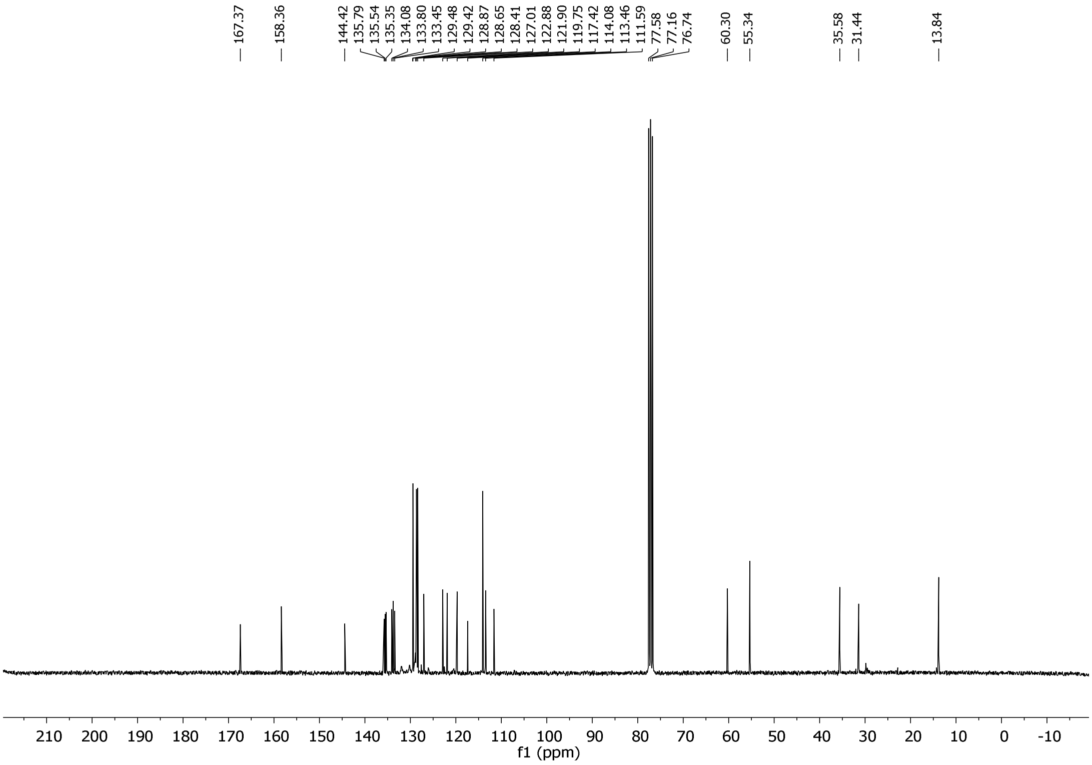

Figure S58. ^{1}H**-NMR (300 MHz,** $CDCl_3$**) and** ^{13}C**-NMR (75 MHz,** $CDCl_3$**) spectra of compound 5q**

5r

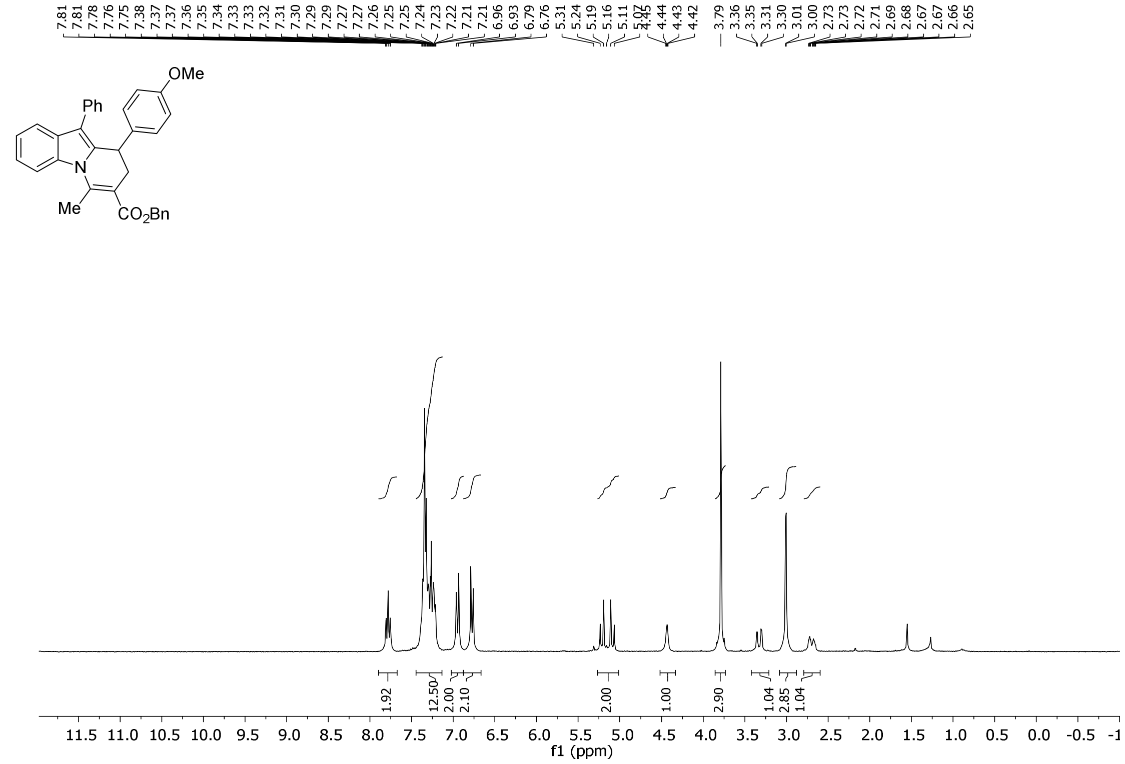

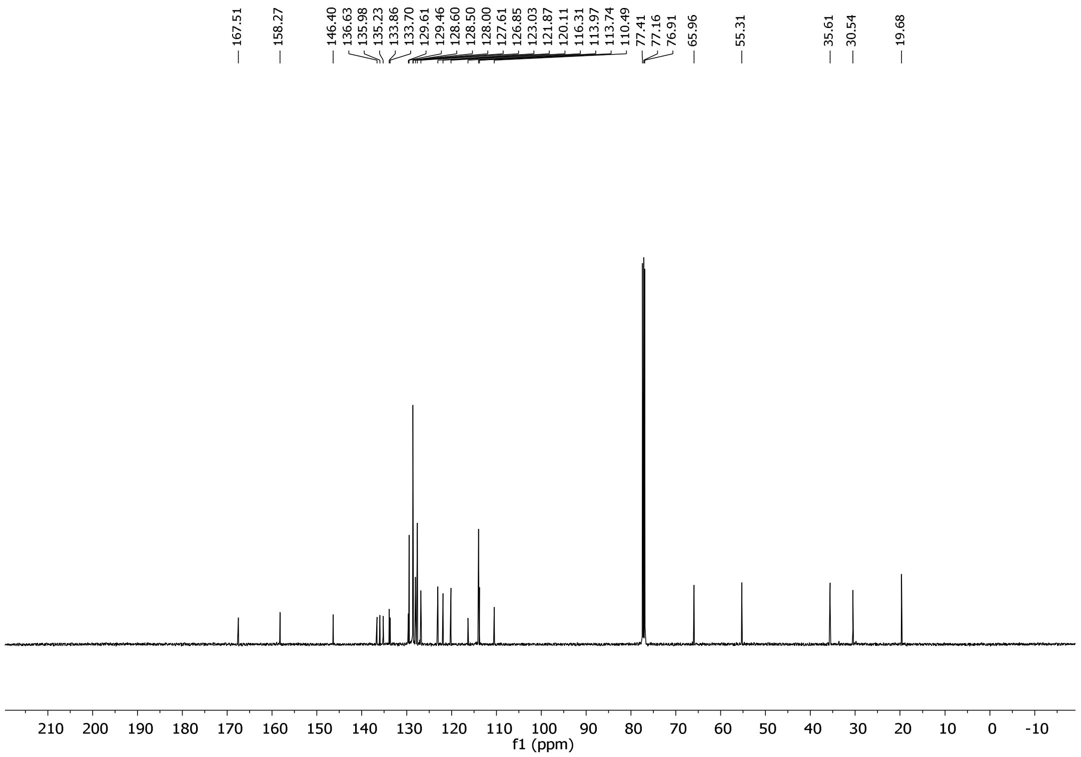

Figure S59. ^{1}H-NMR (300 MHz, $CDCl_3$) and ^{13}C-NMR (75 MHz, $CDCl_3$) spectra of compound 5r

5s

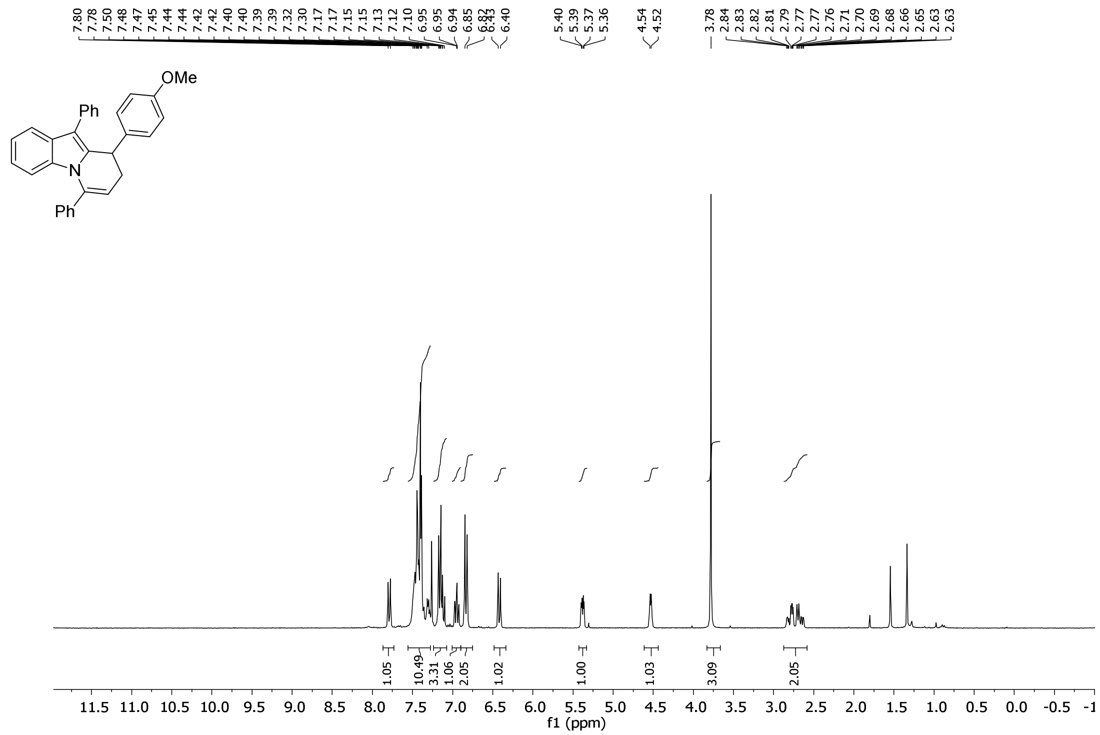

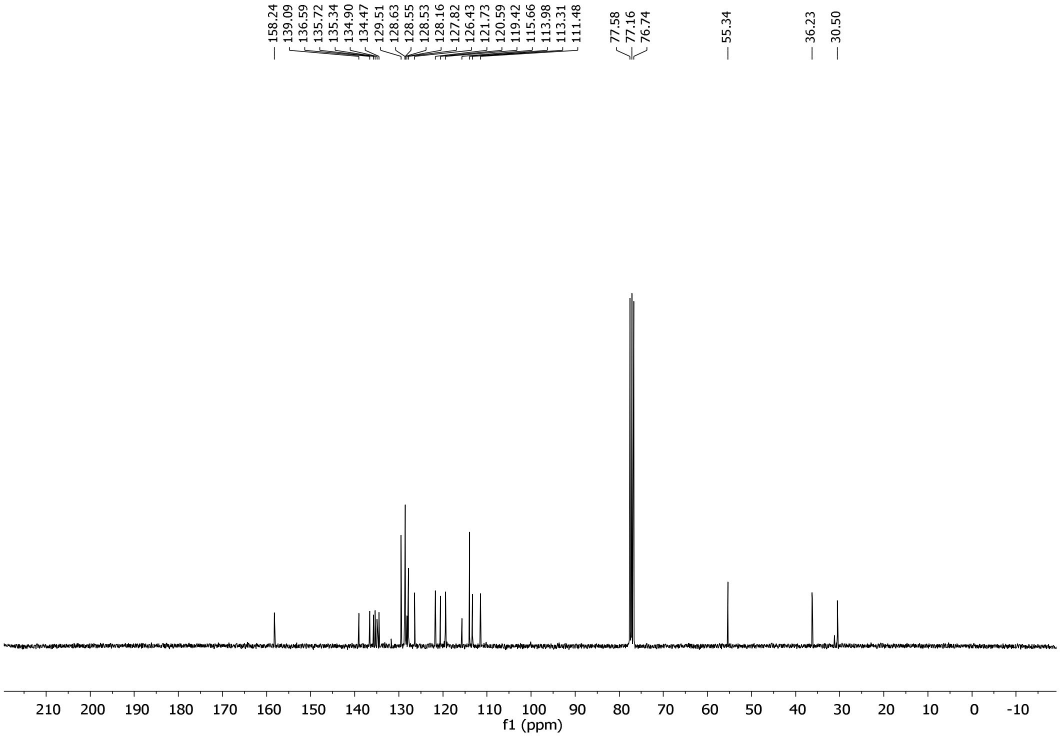

Figure S60. ^{1}**H-NMR (300 MHz, CDCl$_3$) and** ^{13}**C-NMR (75 MHz, CDCl$_3$) spectra of compound 5s**

4.5 Dihydropyridoindoles 6a-g

6a

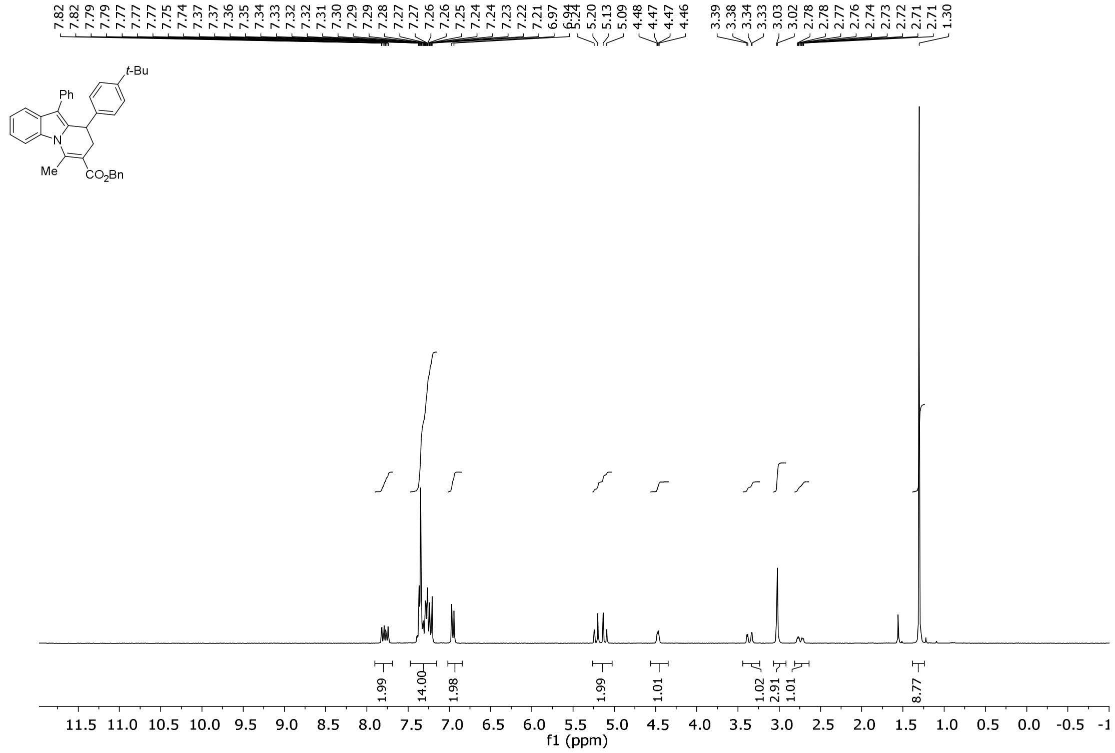

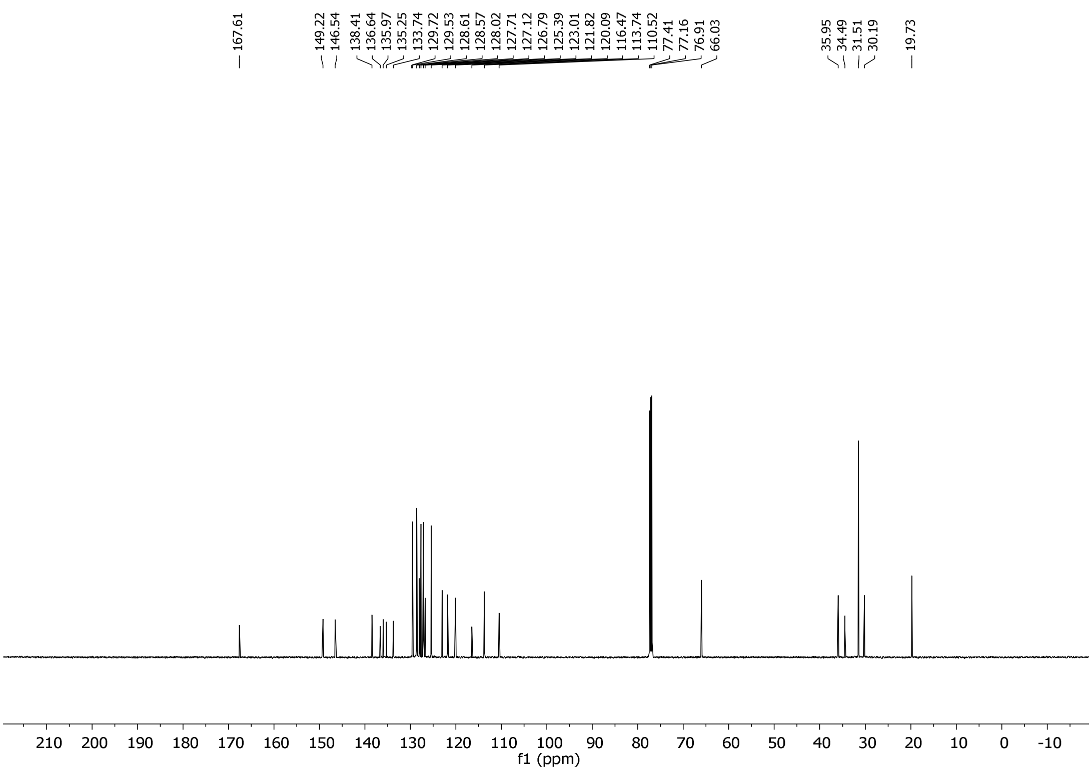

Figure S61. **^{1}H-NMR (300 MHz, $CDCl_3$) and ^{13}C-NMR (75 MHz, $CDCl_3$) spectra of compound 6a**

6b

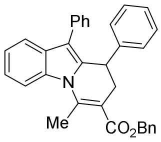

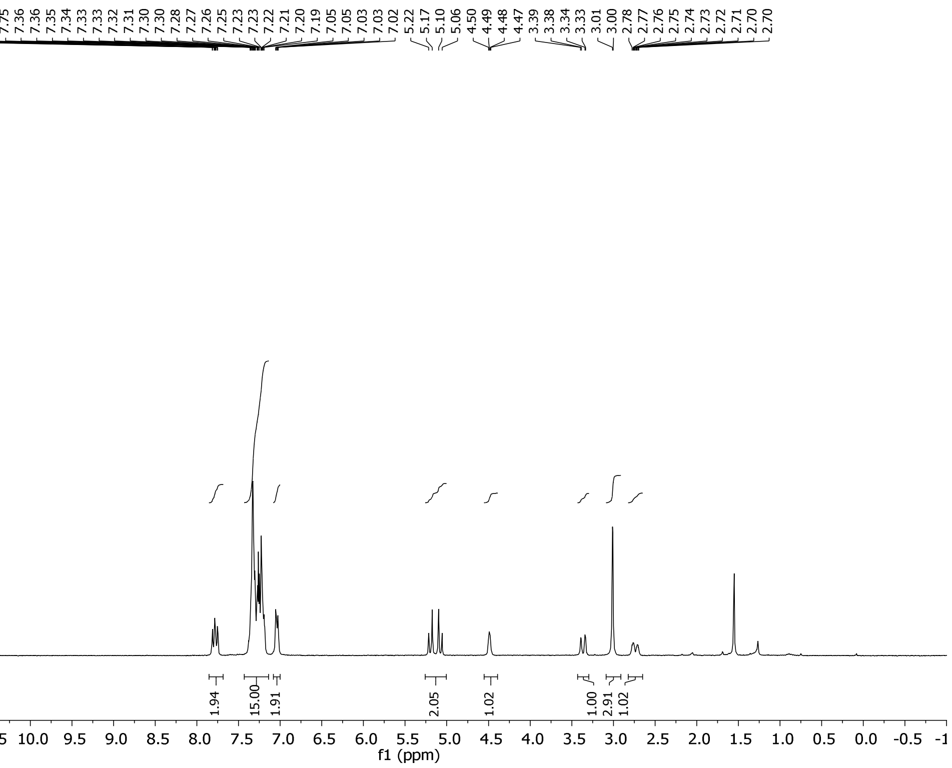

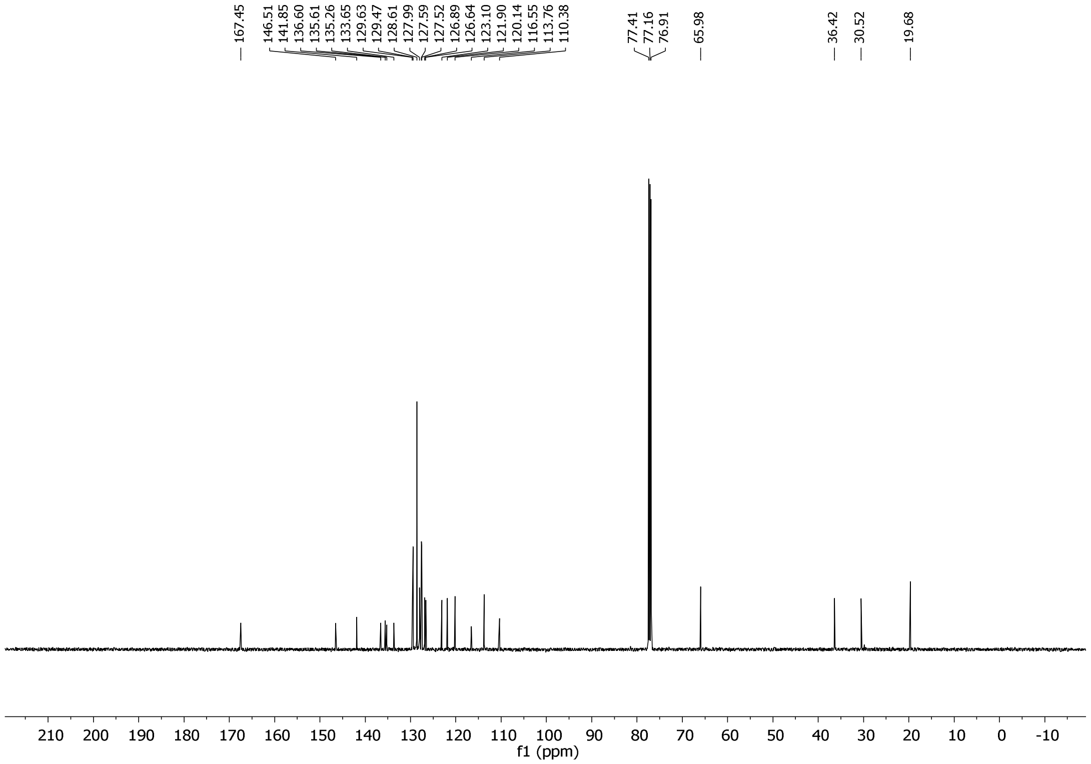

Figure S62. ^{1}**H-NMR (300 MHz,** $CDCl_3$**) and** ^{13}**C-NMR (75 MHz,** $CDCl_3$**) spectra of compound 6b**

6c

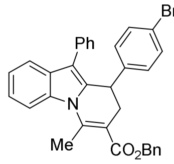

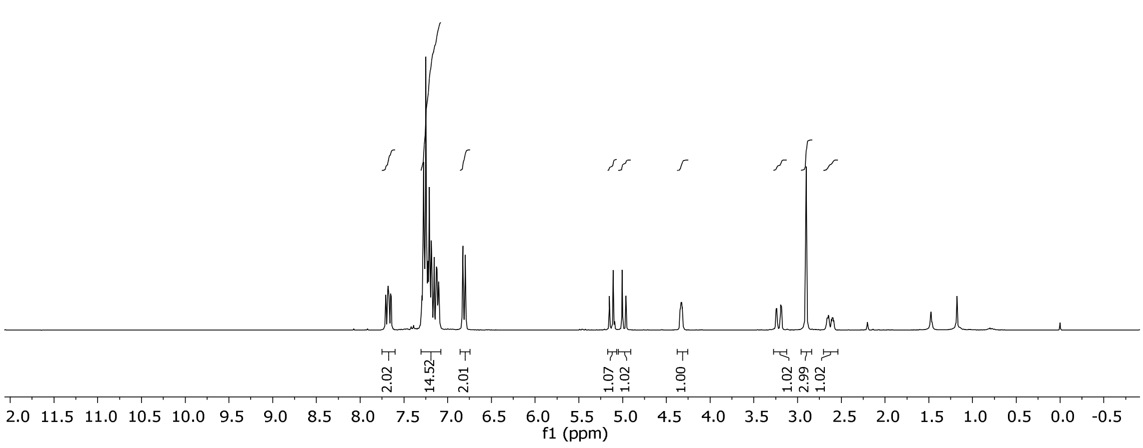

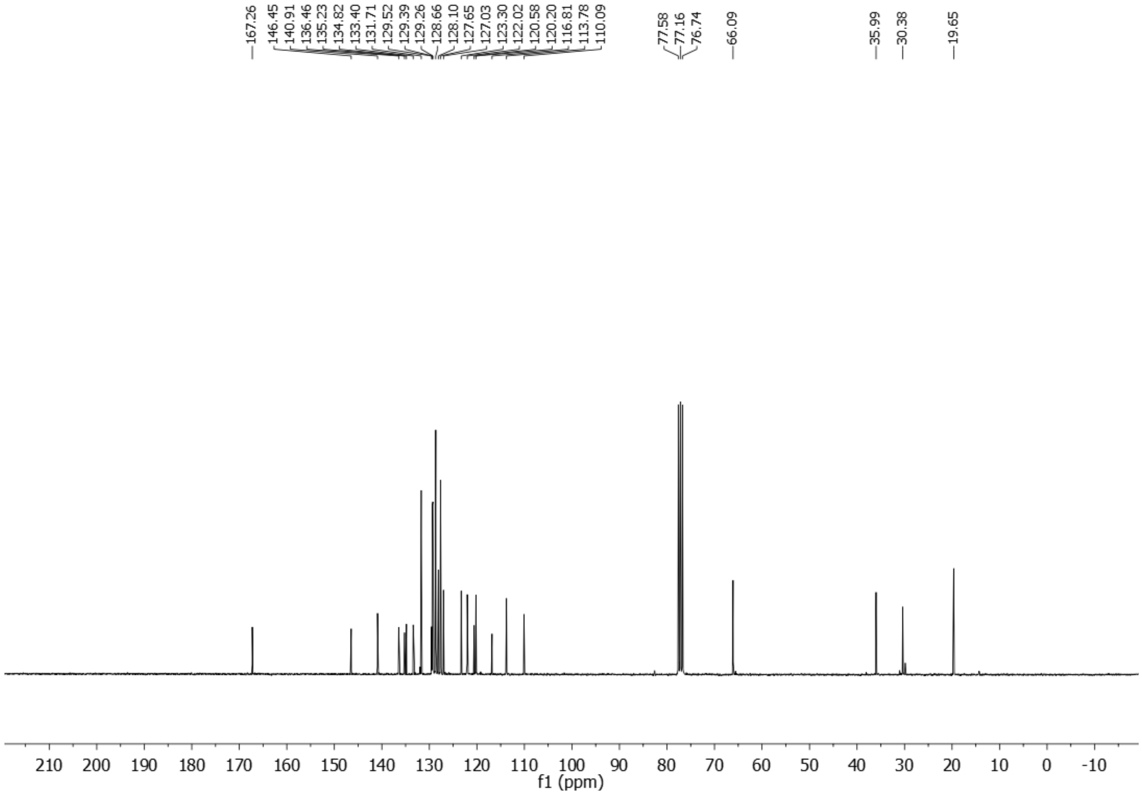

Figure S63. ^{1}**H-NMR (300 MHz, CDCl$_3$) and** ^{13}**C-NMR (75 MHz, CDCl$_3$) spectra of compound 6c**

6d

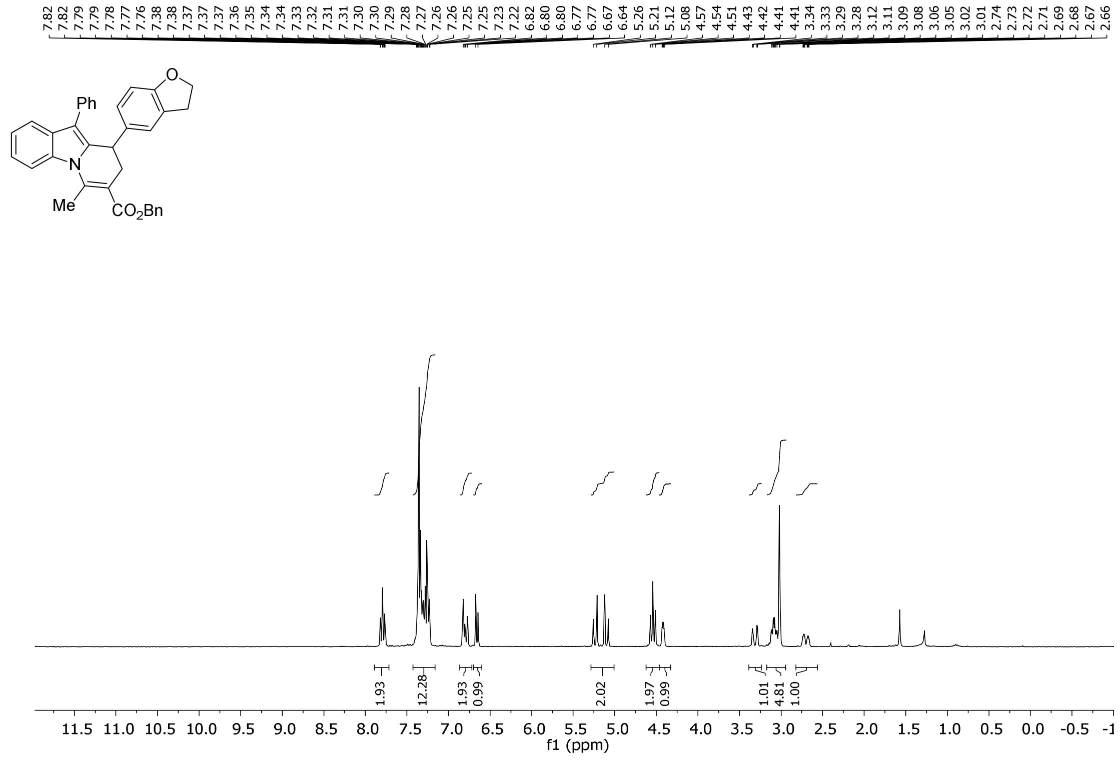

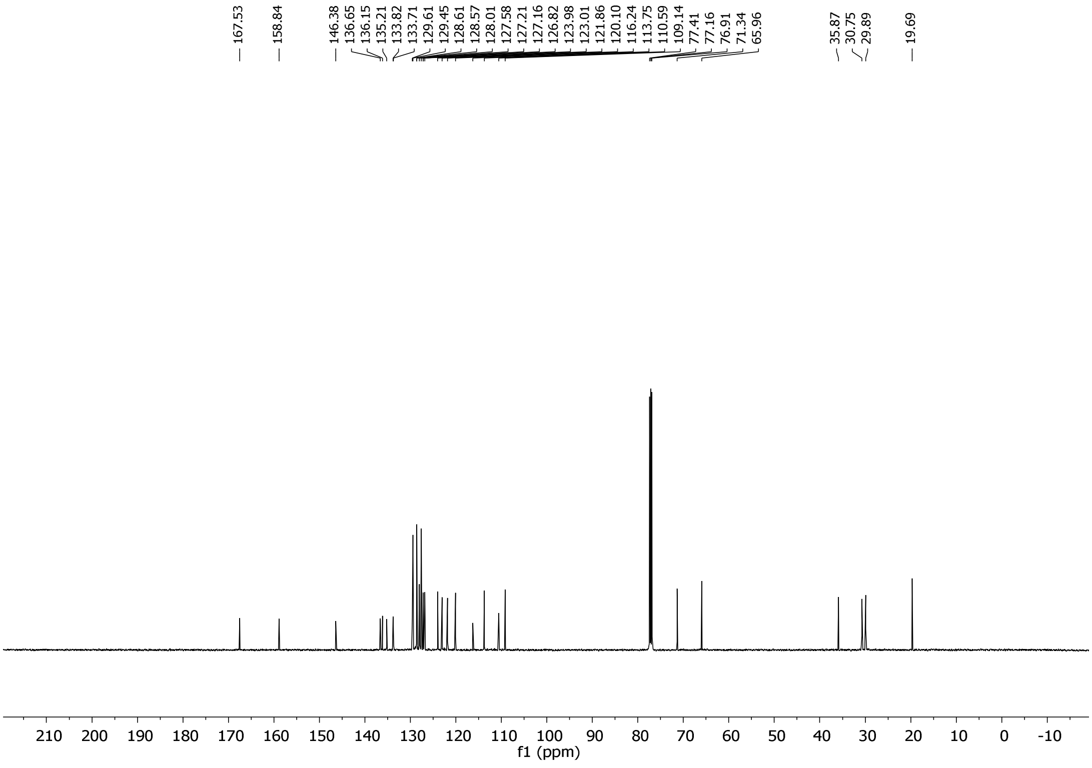

Figure S64. ^{1}H-NMR (300 MHz, $CDCl_3$) and ^{13}C-NMR (75 MHz, $CDCl_3$) spectra of compound 6d

6e

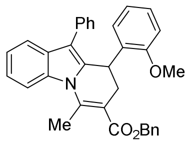

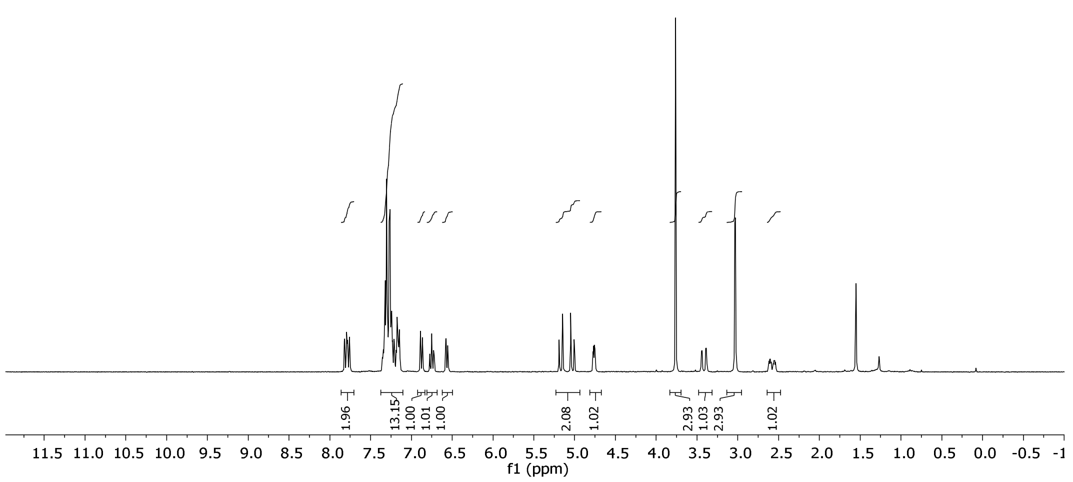

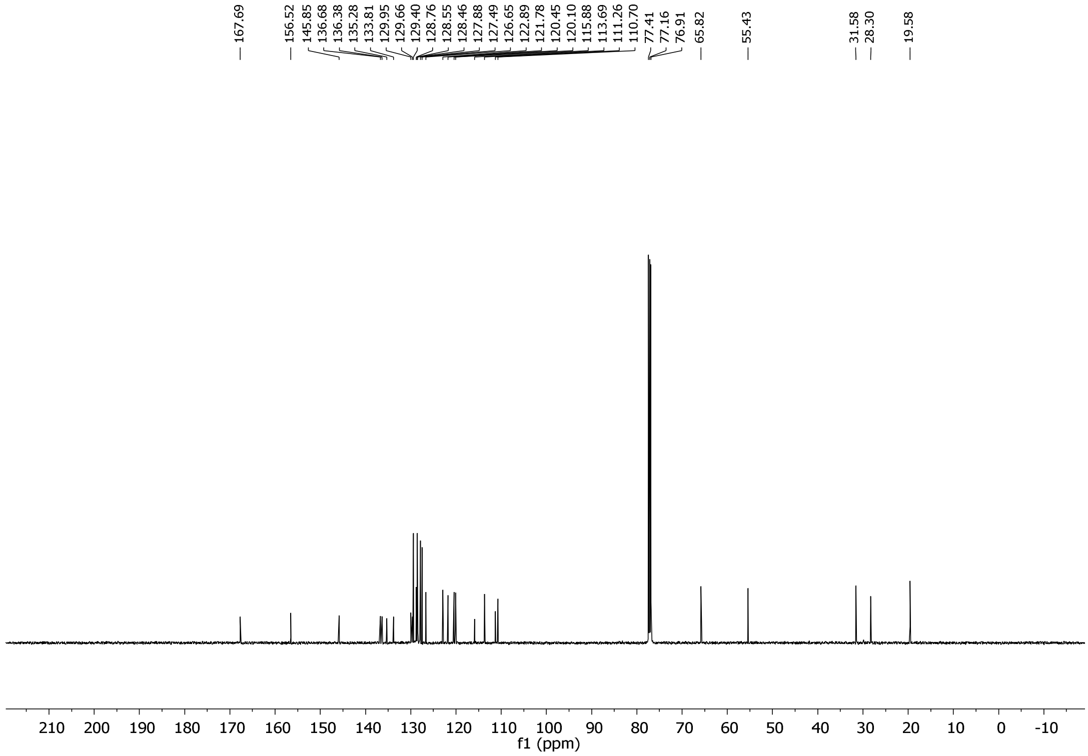

Figure S65. ^{1}H-NMR (300 MHz, $CDCl_3$) and ^{13}C-NMR (75 MHz, $CDCl_3$) spectra of compound 6e

6f

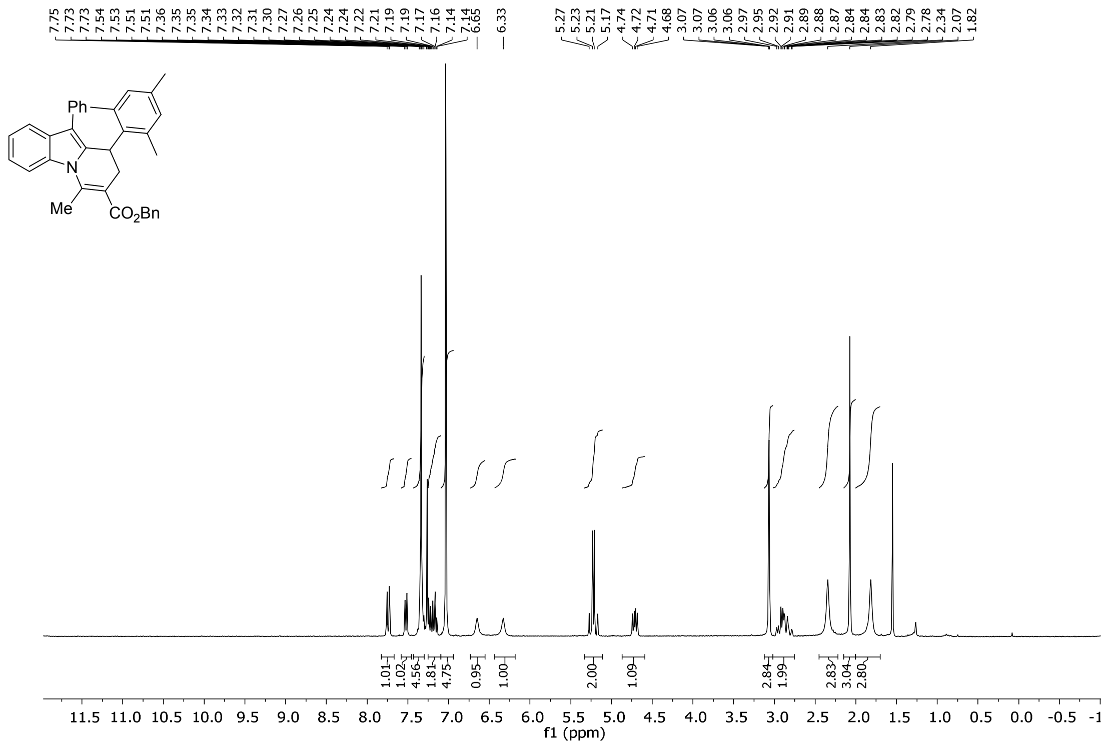

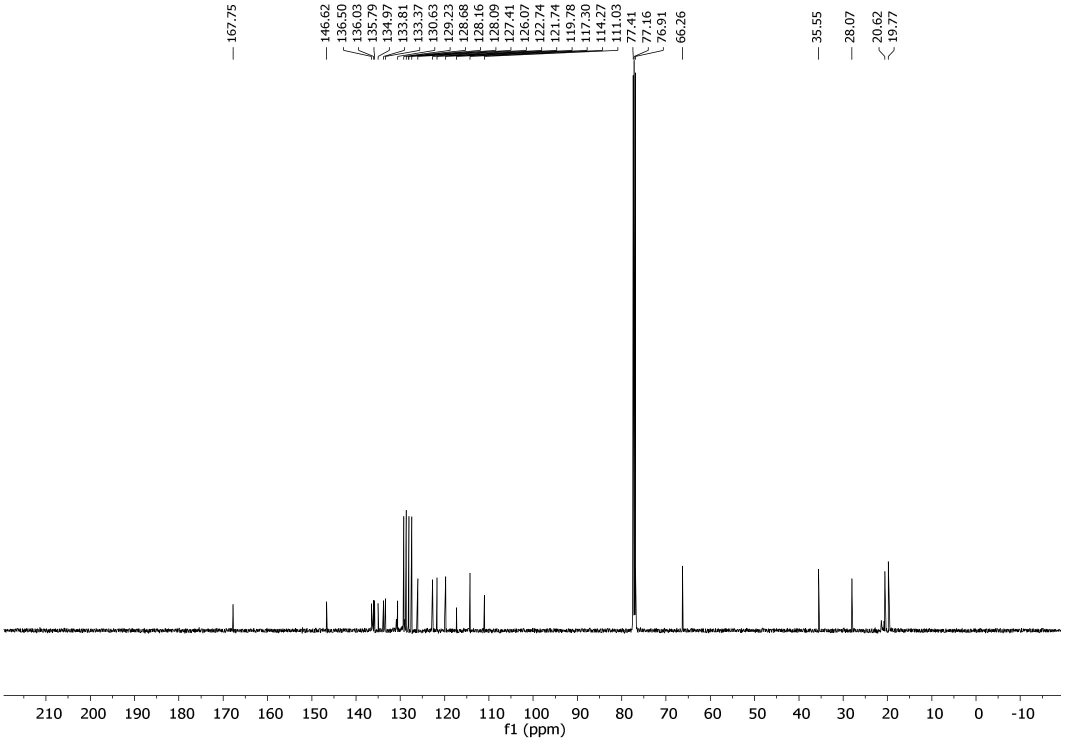

Figure S66. ^{1}H-NMR (300 MHz, $CDCl_3$) and ^{13}C-NMR (75 MHz, $CDCl_3$) spectra of compound 6f

6g

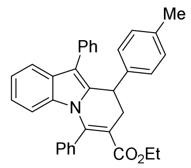

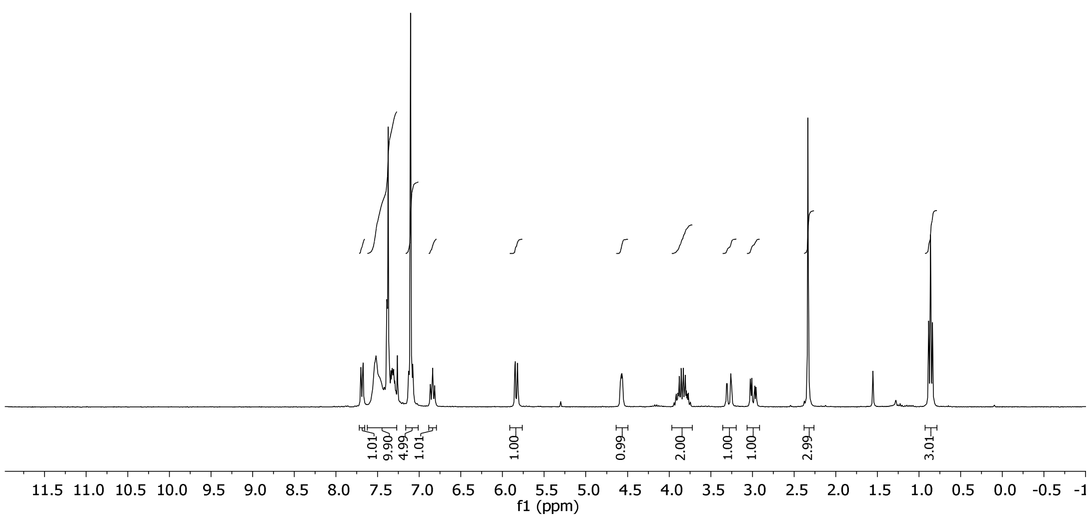

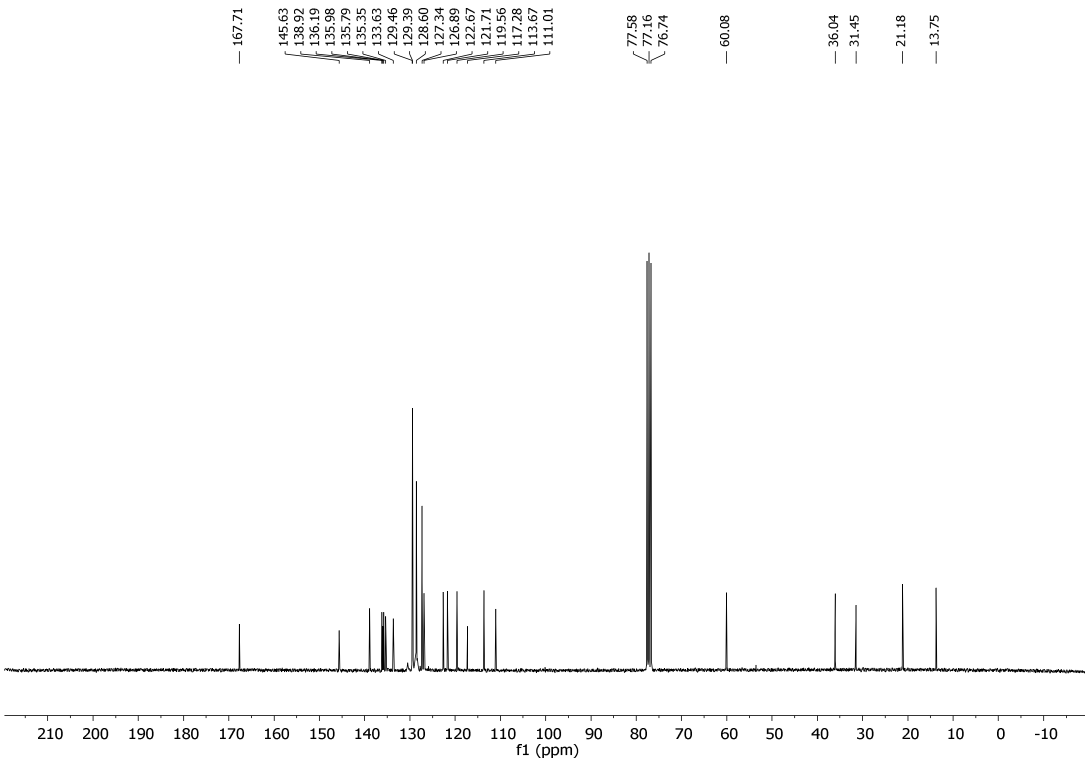

Figure S67. ¹H-NMR (300 MHz, $CDCl_3$) and ¹³C-NMR (75 MHz, $CDCl_3$) spectra of compound 6g

5. Computational Studies

5.1. Computational Methods

All of the calculations were performed using the Gaussian09 program.[22] Computations were done using B3LYP functional[23] in conjunction with Grimme's dispersion correction[24] and standard basis set def2SVP.[25] Geometry full optimizations were made at b3lyp-d3bj/def2SVP level. Solvent effects (toluene) were considered using the PCM model.[26] The nature of stationary points was defined on the basis of calculations of normal vibrational frequencies (force constant Hessian matrix). The optimizations were carried out using the Berny analytical gradient optimization method.[27] Minimum energy pathways for the reactions studied were found by gradient descent of transition states in the forward and backward direction of the transition vector (IRC analysis),[28] using the Hratchian-Schlegel algorithm.[29] Analytical second derivatives of the energy were calculated to classify the nature of every stationary point, to determine the harmonic vibrational frequencies, and to provide zero-point vibrational energy corrections. The thermal and entropic contributions to the free energies were also obtained from the vibrational frequency calculations, using the unscaled frequencies. Structural representations were generated using CYLView.[30]

---

[22] Frisch, M. J.; Trucks, G. W.; Schlegel, H. B.; Scuseria, G. E.; Robb, M. A.; Cheeseman, J. R.; Scalmani, G.; Barone, V.; Mennucci, B.; Petersson, G. A.; Nakatsuji, H.; Caricato, M.; Li, X.; Hratchian, H. P.; Izmaylov, A. F.; Bloino, J.; Zheng, G.; Sonnenberg, J. L.; Hada, M.; Ehara, M.; Toyota, K.; Fukuda, R.; Hasegawa, J.; Ishida, M.; Nakajima, T.; Honda, Y.; Kitao, O.; Nakai, H.; Vreven, T.; Montgomery, J., J. A.; Peralta, J. E.; Ogliaro, F.; Bearpark, M.; Heyd, J. J.; Brothers, E.; Kudin, K. N.; Staroverov, V. N.; Kobayashi, R.; Normand, J.; Raghavachari, K.; Rendell, A.; Burant, J. C.; Iyengar, S. S.; Tomasi, J.; Cossi, M.; Rega, N.; Millam, J. M.; Klene, M.; Knox, J. E.; Cross, J. B.; Bakken, V.; Adamo, C.; Jaramillo, J.; Gomperts, R.; Stratmann, R. E.; Yazyev, O.; Austin, A. J.; Cammi, R.; Pomelli, C.; Ochterski, J. W.; Martin, R. L.; Morokuma, K.; Zakrzewski, V. G.; Voth, G. A.; Salvador, P.; Dannenberg, J. J.; Dapprich, S.; Daniels, A. D.; Farkas, Ö.; Foresman, J. B.; Ortiz, J. V.; Cioslowski, J.; Fox, D. J.; Gaussian, Inc., Wallingford CT,: 2009.
[23] (a) Becke, A. D. *J. Chem. Phys.* **1993**, *98*, 5648-5652. (b) Lee, C.; Yang, W.; Parr, R. G. *Phys. Rev. B* **1988**, *37*, 785-789.
[24] (a) Grimme, S.; Antony, J.; Ehrlich, S.; Krieg, H. *J. Chem. Phys.* **2010**, *132*, 154104-154119. (b) Grimme, S.; Ehrlich, S.; Goerigk, L. *J. Comput. Chem.* **2011**, *32*, 1456-1465.
[25] (a) Weigend, F. *Phys. Chem. Chem. Phys.* **2006**, *8*, 227-236. (b) Weigend, F.; Ahlrichs, R. *Phys. Chem. Chem. Phys.* **2005**, *7*, 3297-3305.
[26] (a) J. Tomasi and M. Persico, *Chem. Rev.*, 1994, **94**, 2027-2094. (b) M. Cossi, G. Scalmani, N. Rega and V. Barone, *J. Chem. Phys.*, 2002, **117**, 43-54.
[27] (a) Schlegel, H. B. *J. Comput. Chem.* **1982**, *3*, 214218. (b) Schlegel, H. B. In *Modern Electronic Structure Theory*; Yarkony, D. R., Ed.; World Scientific Publishing: Singapore, 1994.
[28] (a) Fukui, K. *Acc. Chem. Res.* **1981**, *14*, 363-368. (b) Fukui, K. *J. Phys. Chem.* **1970**, *74*, 4161-4163.
[29] Hratchian, H. P.; Schlegel, H. B. *J. Phys. Chem. A* **2002**, *106*, 165-169.
[30] C. Y. Legault, *Université de Sherbrooke*, 2009, http://www.cylview.org.

5.2. Reaction of cyclopropane A with indole 2a

During the reaction of cyclopropane **A** with indole **2a**, diastereomers can be formed. **a** and **b** series correspond to the more and less favoured pathways, respectively. In the main manuscript only the favoured **a** series is shown.

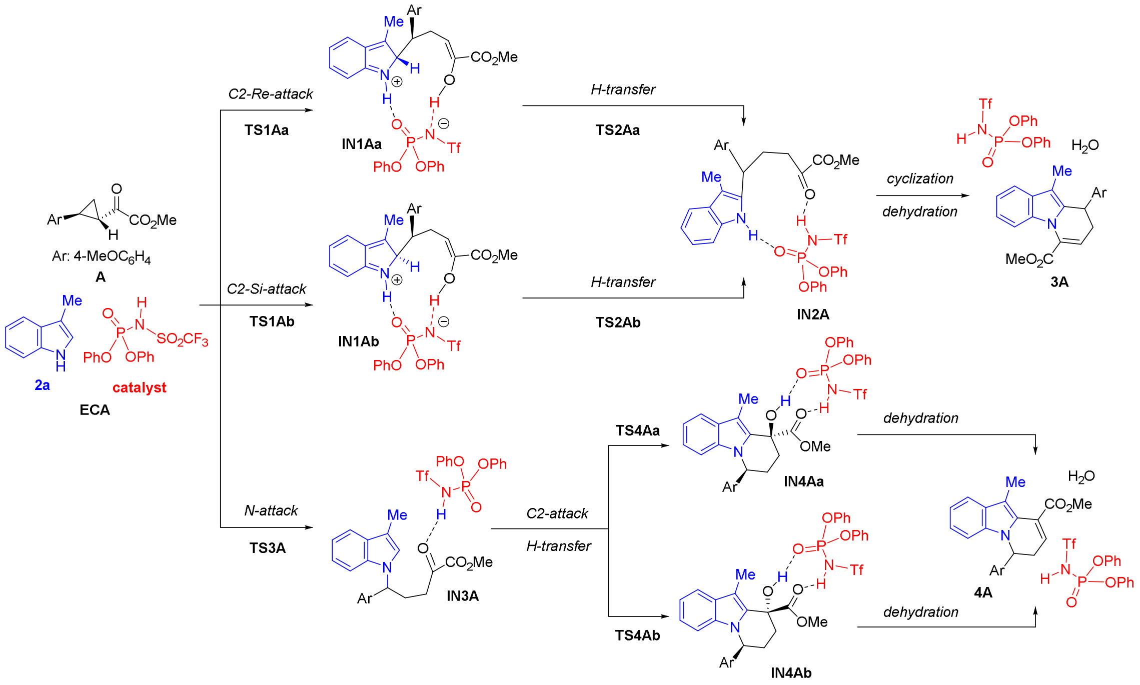

Scheme S6. Reaction of cyclopropane **A** with indole **2a**

5.2.1. Energy Profiles

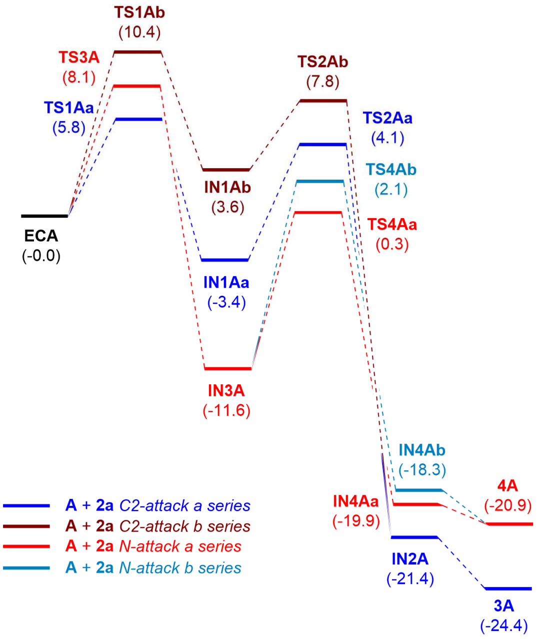

Figure S68. Energy profile (free relative energies given in kcal/mol) for the reaction of cyclopropane **A** with indole **2a**

5.2.2. Energy values

Table S4. Total energy and zero-point vibrational energy (hartrees); absolute (hartrees) and relative (kcal/mol) energies (b3lyp-gd3bj/def2svp/pcm=toluene) for the reaction of cyclopropane **A** with indole **2a**.

	E	ZPVE	E_0	ΔE_0[b]	G	ΔG[b]	im. freq[c]
**A**	-804.136659	0.247877	-803.888782		-803.935601		
**2a**	-402.887911	0.157466	-402.730445		-402.762883		
**$(PhO)_2P(O)NHTf$**	-1970.887857	0.238259	-1970.649598		-1970.705829		
**H_2O**	-76.358890	0.021227	-76.337663		-76.355327		
**ECA**[a]	-3177.992997	0.647542	-3177.345455	0.0	-3177.432973	0.0	
**TS1Aa**	-3177.984896	0.647568	-3177.337328	5.1	-3177.423772	5.8	-249.6
**TS1Ab**	-3177.977210	0.647789	-3177.329421	10.1	-3177.416409	10.4	-215.9
**IN1Aa**	-3177.999129	0.649241	-3177.349888	-2.8	-3177.438386	-3.4	
**IN1Ab**	-3177.987086	0.648915	-3177.338171	4.6	-3177.427253	3.6	
**TS2Aa**	-3177.989128	0.645838	-3177.343290	1.4	-3177.426360	4.1	-788.5
**TS2Ab**	-3177.981755	0.644684	-3177.337071	5.3	-3177.420488	7.8	-1347.9
**IN2A**	-3178.030531	0.650798	-3177.379733	-21.5	-3177.467035	-21.4	
**3A**	-3178.032854	0.648961	-3177.383893	-24.1	-3177.471792	-24.4	
**TS3A**	-3177.981802	0.648155	-3177.333647	7.4	-3177.420067	8.1	-147.5
**IN3a**	-3178.013123	0.649770	-3177.363353	-11.2	-3177.451409	-11.6	
**TS4Aa**	-3178.001903	0.649796	-3177.352107	-4.2	-3177.432540	0.3	-171.7
**TS4Ab**	-3177.995834	0.649555	-3177.346279	-0.5	-3177.429566	2.1	-298.3
**IN4Aa**	-3178.032773	0.652039	-3177.380734	-22.1	-3177.464657	-19.9	
**IN4Ab**	-3178.024747	0.651364	-3177.373383	-17.5	-3177.462083	-18.3	
**4A**	-3178.019465	0.648653	-3177.380812	-22.2	-3177.466317	-20.9	

[a] encounter complex formed by the two reagents and the catalyst. [b] referred to **ECA**. [c]All minima have no imaginary fequencies and transition structures presented one imaginary frequency that is listed in the table.

5.2.3. Optimized geometries

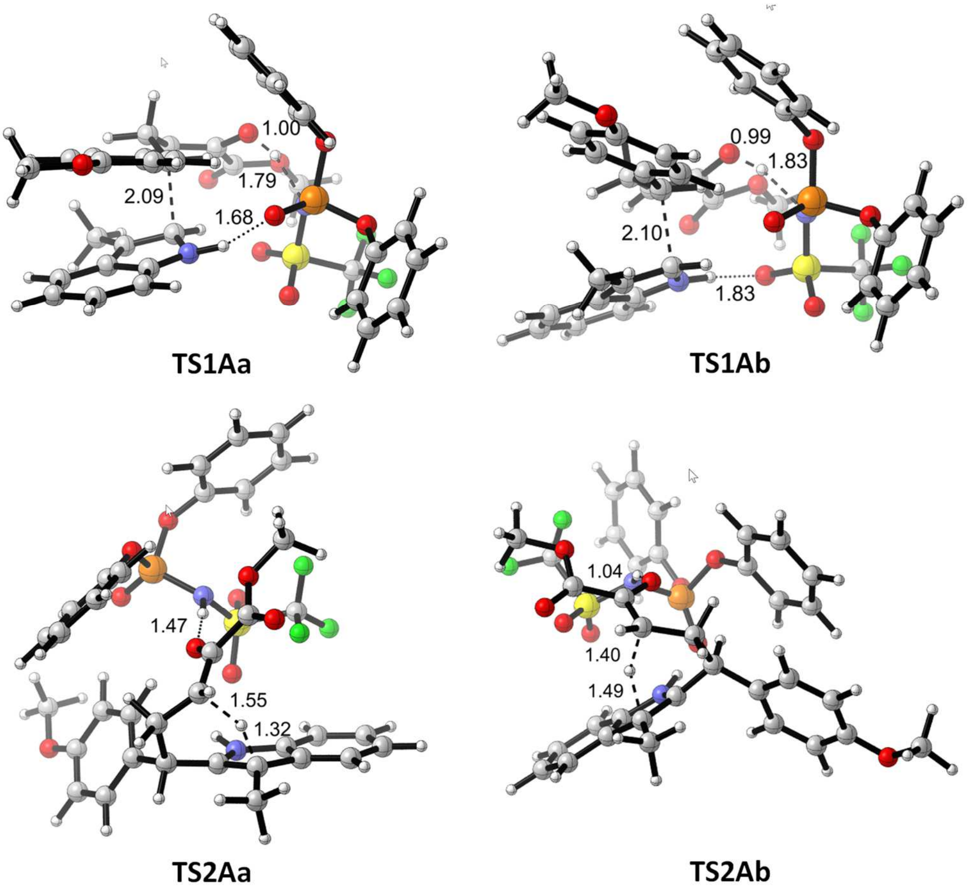

Figure S69. Optimized (b3lyp-gd3bj/def2svp/pcm=toluene) geometries of the located transition structures for the *C2*-attack.

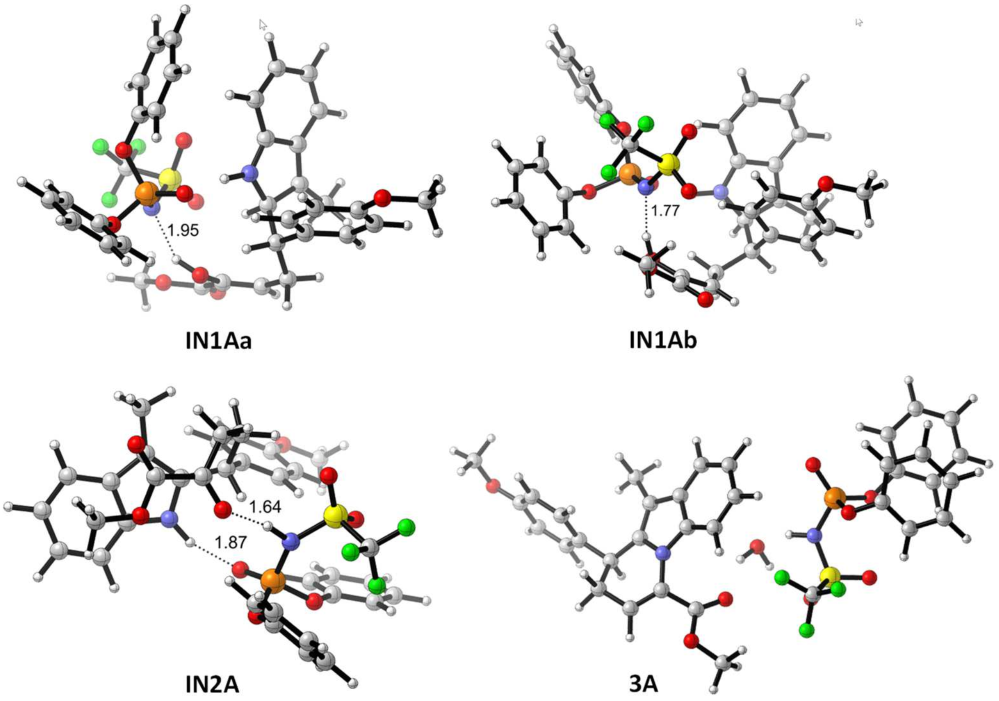

Figure S70. Optimized (b3lyp-gd3bj/def2svp/pcm=toluene) geometries of the located minima for the *C2*-attack.

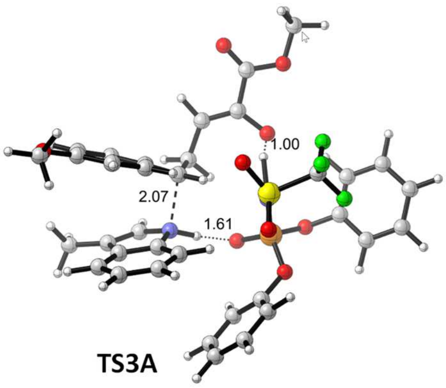

TS3A

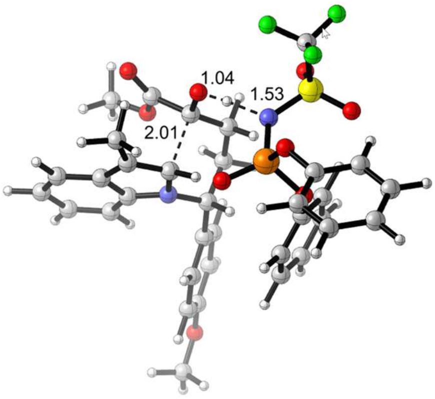

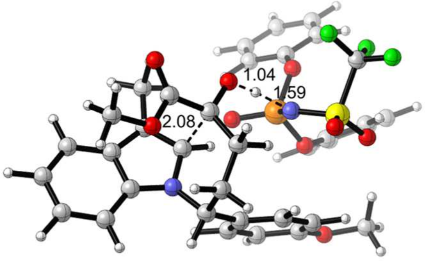

TS4Aa **TS4Ab**

Figure S71. Optimized (b3lyp-gd3bj/def2svp/pcm=toluene) geometries of the located transition structures for the *N*-attack.

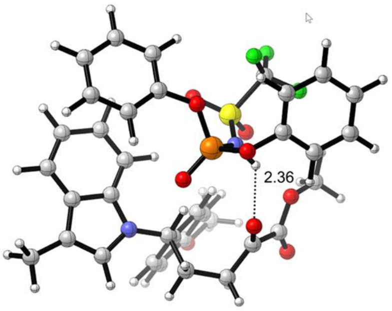

IN3A

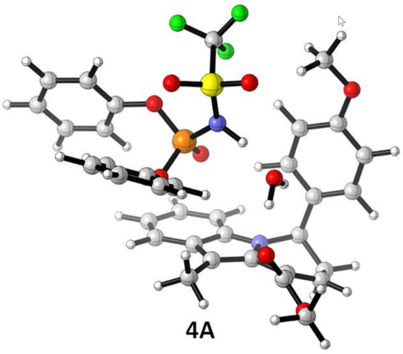

4A

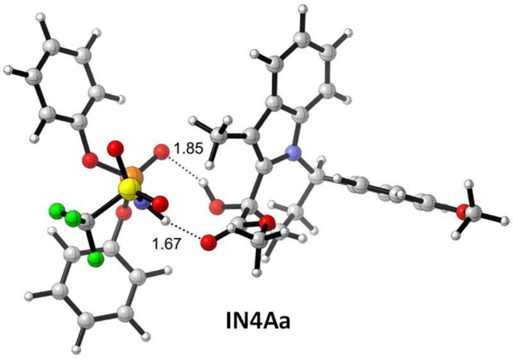

IN4Aa

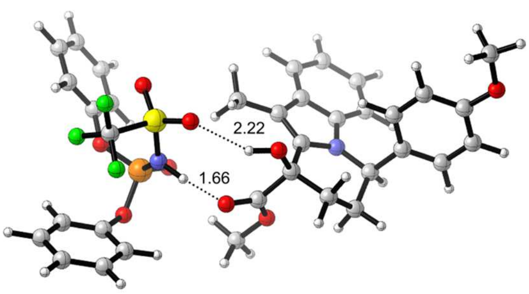

IN4Ab

Figure S72. Optimized (b3lyp-gd3bj/def2svp/pcm=toluene) geometries of the located minima for the *N*-attack.

5.3. Reaction of cyclopropane B with indole 2a

During the reaction of cyclopropane **B** with indole **2a**, diastereomers can be formed. **a** and **b** series correspond to the more and less favoured pathways, respectively. In the main manuscript only the favoured **a** series is shown. Although two intermediates **IN3Ba** and **IN3Bb** can be formed, only one **TS3B** is possible due to the required coordination between the reagent and the catalyst.

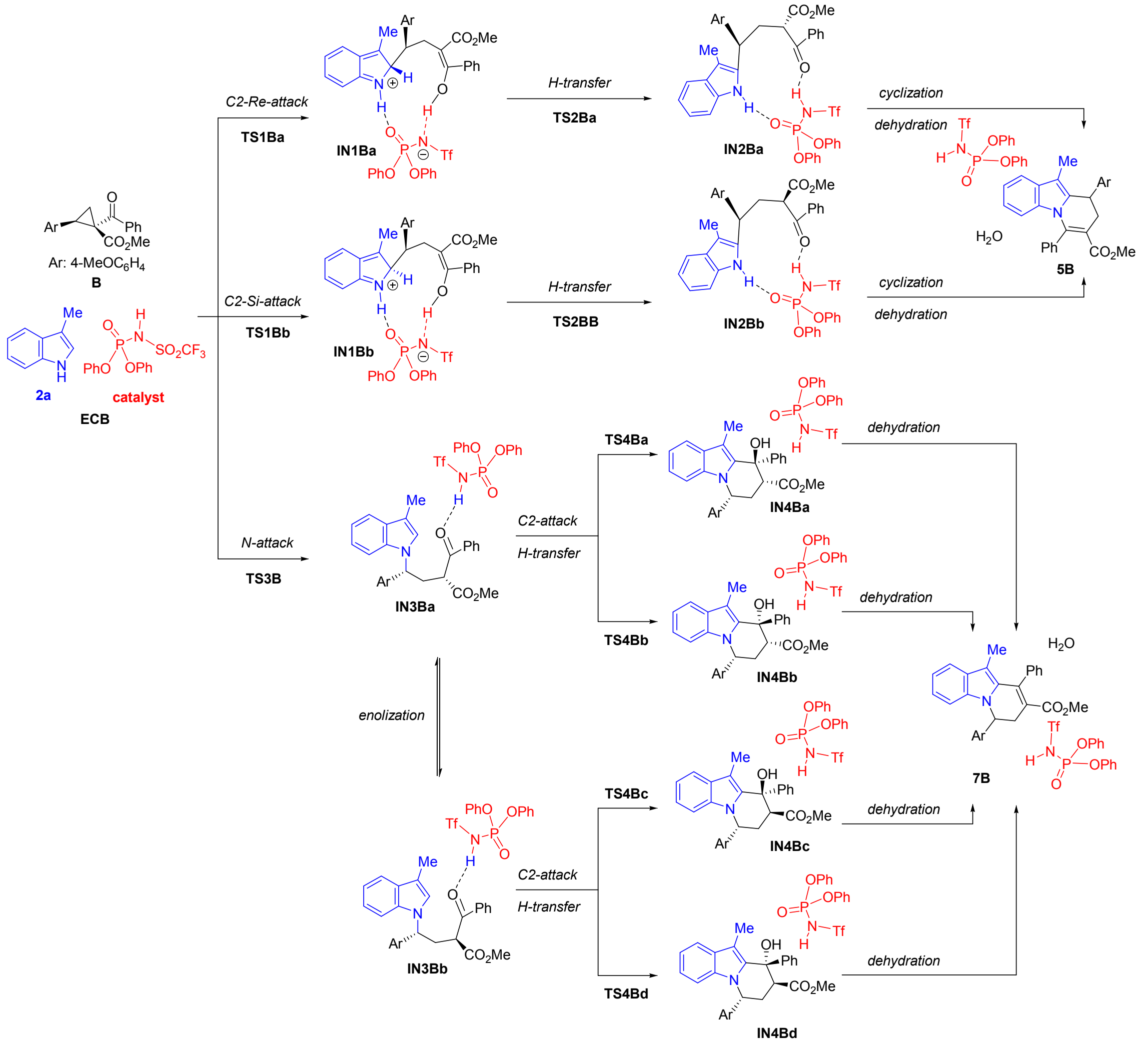

Scheme S7. Reaction of cyclopropane **B** with indole **2a**

5.3.1. Energy Profiles

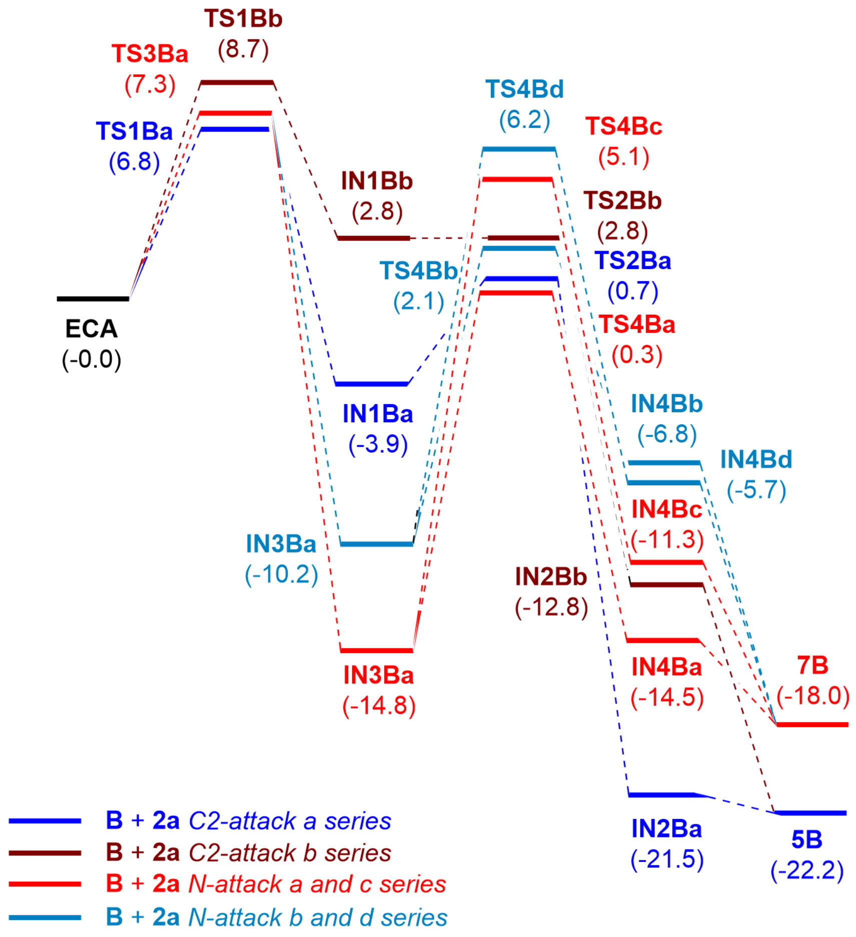

Figure S72. Energy profile (free relative energies given in kcal/mol) for the reaction of cyclopropane **B** with indole **2a**

5.3.2. Energy values

Table S5. Total energy and zero-point vibrational energy (hartrees); absolute (hartrees) and relative (kcal/mol) energies (b3lyp-gd3bj/def2svp/pcm=toluene) for the reaction of cyclopropane **B** with indole **2a**.

	E	ZPVE	E_0	ΔE_0[b]	G	ΔG[b]	im. freq[c]
**B**	-1035.069736	0.329060	-1034.740676		-1034.793930		
**2a**	-402.887911	0.157466	-402.730445		-402.762883		
**(PhO)$_2$P(O)NHTf**	-1970.887857	0.238259	-1970.649598		-1970.705829		
**H2O**	-76.358890	0.021227	-76.337663		-76.355327		
**ECB**[a]	-3408.917001	0.728241	-3408.188760	0.0	-3408.283475	0.0	
**TS1Ba**	-3408.910452	0.729495	-3408.180957	4.9	-3408.272711	6.8	-352.6
**TS1Bb**	-3408.905479	0.728517	-3408.176962	7.4	-3408.269565	8.7	-136.6
**IN1Ba**	-3408.926049	0.730252	-3408.195797	-4.4	-3408.289761	-3.9	
**IN1Bb**	-3408.917315	0.731029	-3408.186286	1.6	-3408.279017	2.8	
**TS2Ba**	-3408.918701	0.726374	-3408.192327	-2.2	-3408.282311	0.7	-800.5
**TS2Bb**	-3408.912052	0.724731	-3408.187321	0.9	-3408.279045	2.8	-1466.3
**IN2Ba**	-3408.955075	0.731390	-3408.223685	-21.9	-3408.317674	-21.5	
**IN2Bb**	-3408.937882	0.730660	-3408.207222	-11.6	-3408.303951	-12.8	
**5B**	-3408.958833	0.730555	-3408.228278	-24.8	-3408.318820	-22.2	
**TS3Ba**	-3408.910994	0.729872	-3408.181122	4.8	-3408.271765	7.3	-188.6
**IN3Ba**	-3408.941242	0.731425	-3408.209817	-13.2	-3408.307074	-14.8	
**IN3Bb**	-3408.937014	0.731003	-3408.206011	-10.8	-3408.299777	-10.2	
**TS4Ba**	-3408.917736	0.731593	-3408.186143	1.6	-3408.279254	2.6	-298.9
**TS4Bb**	-3408.925095	0.731981	-3408.193114	-2.7	-3408.279558	2.5	-336.8
**TS4Bc**	-3408.912783	0.730736	-3408.182047	4.2	-3408.275322	5.1	-319.6
**TS4Bd**	-3408.913640	0.730913	-3408.182727	3.8	-3408.273572	6.2	-348.7
**IN4Ba**	-3408.951888	0.733931	-3408.217957	-18.3	-3408.306630	-14.5	
**IN4Bb**	-3408.936018	0.733543	-3408.202475	-8.6	-3408.294330	-6.8	
**IN4Bc**	-3408.945866	0.732914	-3408.212952	-15.2	-3408.301439	-11.3	
**IN4Bd**	-3408.936720	0.733356	-3408.203364	-9.2	-3408.292500	-5.7	
**7B**	-3408.950035	0.729934	-3408.220101	-19.7	-3408.312212	-18.0	

[a] encounter complex formed by the two reagents and the catalyst. [b] referred to **ECB**. [c]All minima have no imaginary fequencies and transition structures presented one imaginary frequency that is listed in the table.

5.3.3. Optimized geometries

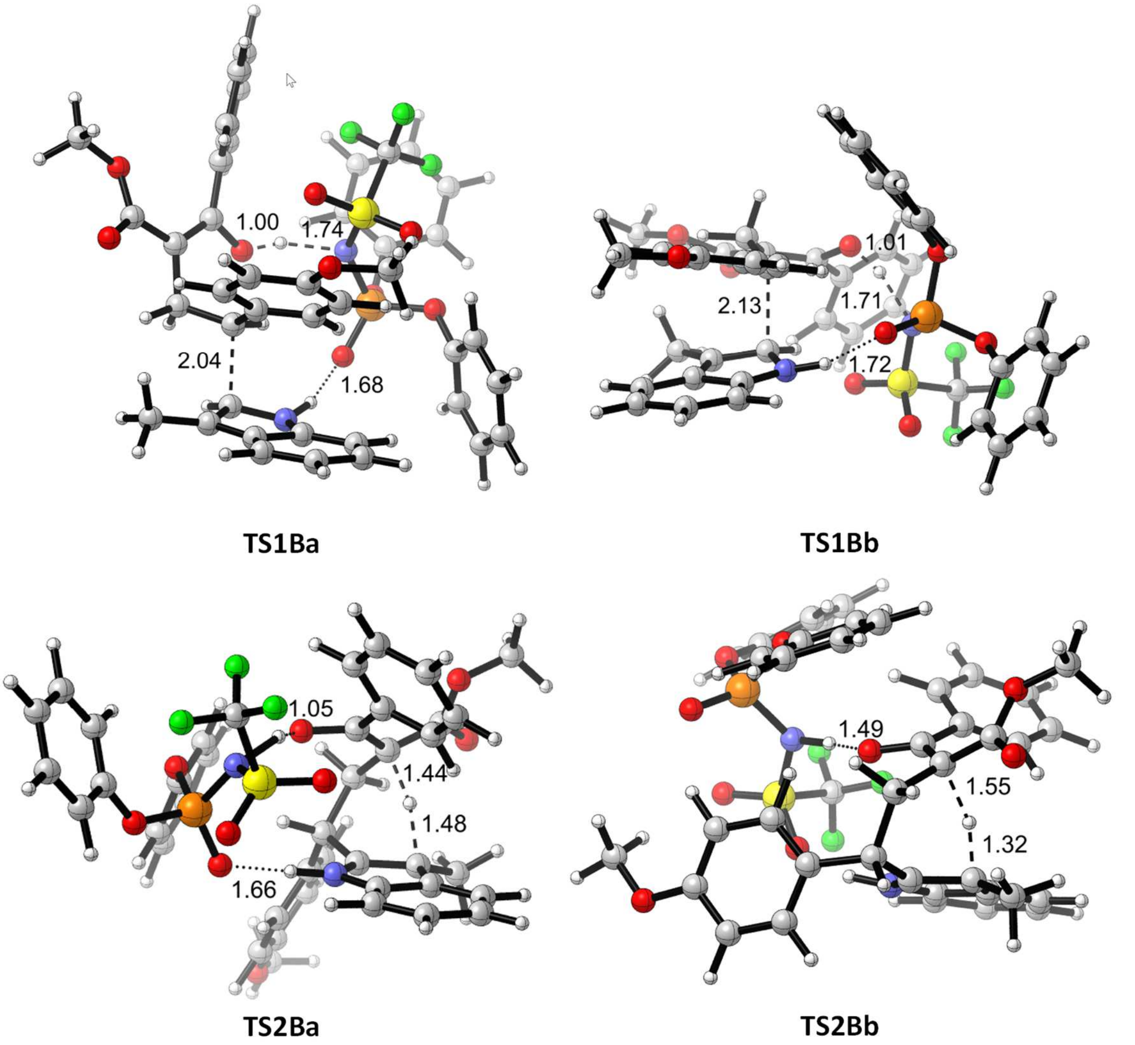

Figure S73. Optimized (b3lyp-gd3bj/def2svp/pcm=toluene) geometries of the located transition structures for the *C2*-attack.

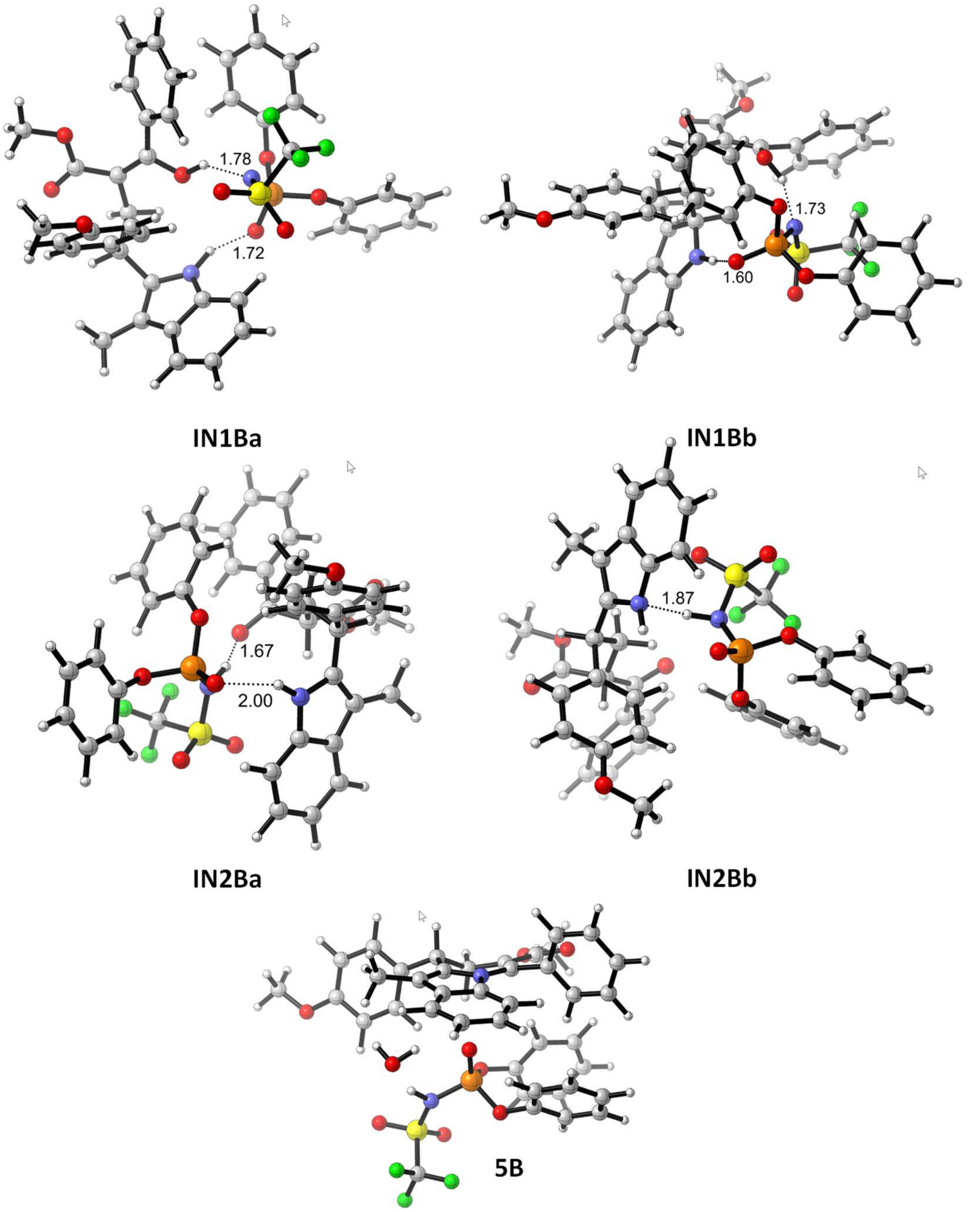

Figure S74. Optimized (b3lyp-gd3bj/def2svp/pcm=toluene) geometries of the located minima for the *C2*-attack.

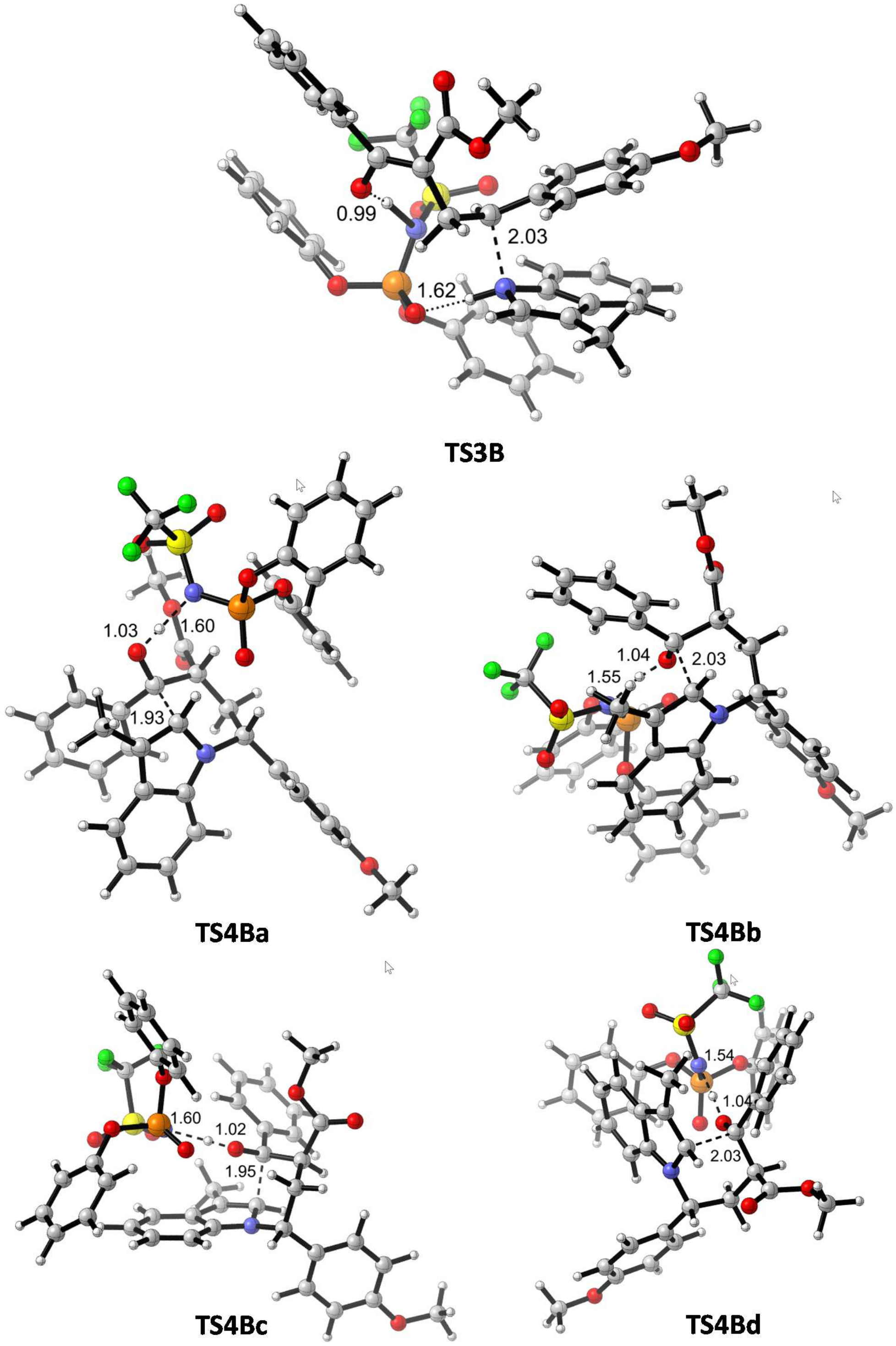

Figure S75. Optimized (b3lyp-gd3bj/def2svp/pcm=toluene) geometries of the located transition structures for the *N*-attack.

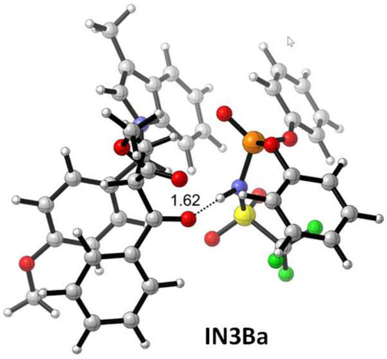

IN3Ba

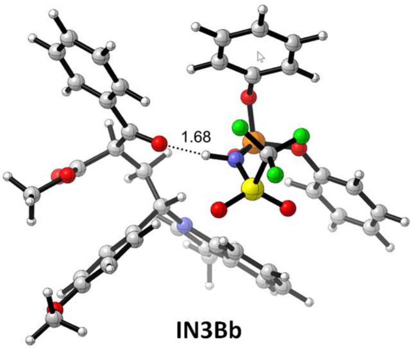

IN3Bb

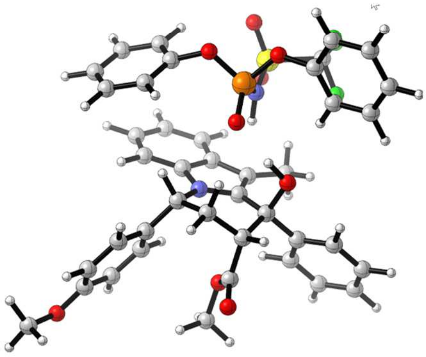

IN4Ba

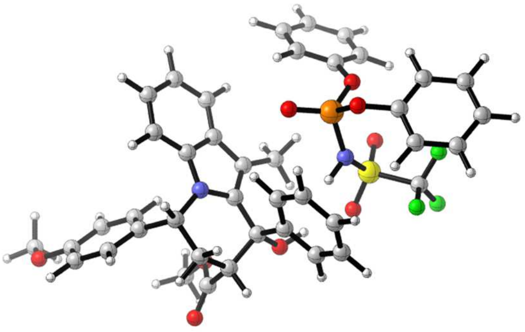

IN4Bb

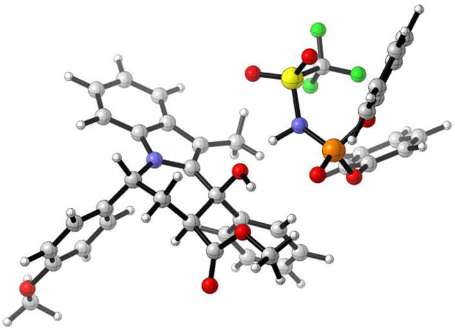

IN4Bc

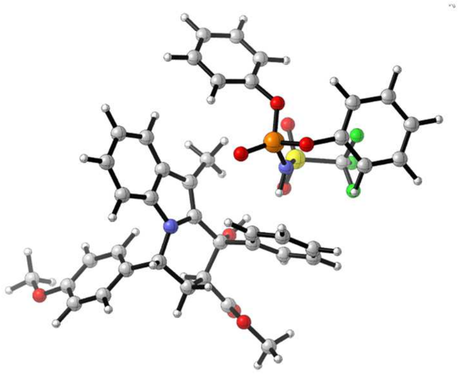

IN4Bd

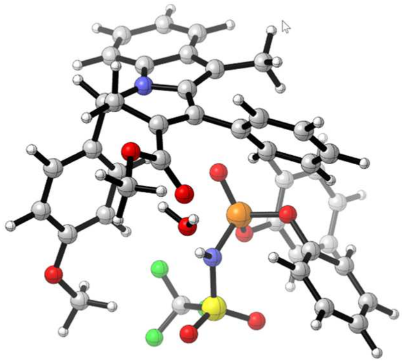

7B

Figure S76. Optimized (b3lyp-gd3bj/def2svp/pcm=toluene) geometries of the located minima for the *N*-attack.

6. Cartesian coordinates

A

C	-1.3222217147	0.4016606166	0.6773899001
O	-1.3321141213	0.4112803075	1.8876542841
C	-1.8313590800	-0.7459788769	-0.1084007771
C	-1.1166241715	-1.2885867522	-1.3660337002
C	-2.4604041293	-0.6512742121	-1.4847145783
H	-0.2882822362	-0.6696656518	-1.7138469595
H	-2.5024366259	0.3314659838	-1.9553104487
H	-3.3275530298	-1.2939387816	-1.6574108528
C	-0.8907783748	-2.7544725753	-1.4983091793
C	0.3529196332	-3.2250905691	-1.9331830450
C	-1.8803261406	-3.7097683919	-1.1984937391
C	0.6190036233	-4.5913588815	-2.0725598475
H	1.1408949742	-2.5065880505	-2.1736832488
C	-1.6333187462	-5.0701272847	-1.3281329272
H	-2.8667828108	-3.3889998206	-0.8543612331
C	-0.3786548208	-5.5267395394	-1.7678659951
H	1.6026520084	-4.9092841752	-2.4169306025
H	-2.4003456325	-5.8108271534	-1.0949935321
O	-0.2303394116	-6.8718682784	-1.8652517354
C	1.0059205226	-7.3906383029	-2.2957527895
H	1.2606675439	-7.0519883487	-3.3164617272
H	0.9046469700	-8.4840223948	-2.2983596925
H	1.8293030113	-7.1085933228	-1.6147007848
H	-2.2270373084	-1.5155831950	0.5594315529
C	-0.7493443943	1.6229773787	-0.0840653992
O	-0.5905315832	1.6741078598	-1.2831130256
O	-0.4477580508	2.6111765420	0.7512866296
C	0.0994323178	3.7908937125	0.1605714763
H	1.0388468853	3.5607913765	-0.3642311283
H	-0.6064947758	4.2248799299	-0.5635122576
H	0.2812431789	4.4885843917	0.9861049033

B

C	0.2984437238	0.5198054288	0.2935278737
O	1.2784994640	0.3475603561	0.9937167094
C	-0.1000786949	-0.5022443556	-0.7468650635
C	0.8134496447	-1.7275822414	-0.8816086430
C	0.8115780058	-0.6861747421	-1.9467445363
H	1.6371855613	-1.6671717953	-0.1632398403
H	1.6732616181	-0.0186704371	-2.0152113025
H	0.2905931438	-0.8918916561	-2.8845836998
C	0.2046802951	-3.0806210464	-1.0491027442
C	0.0585173082	-3.6970998382	-2.2927444521
C	-0.2714265648	-3.7605167980	0.0876903377
C	-0.5387369068	-4.9563214268	-2.4193637489
H	0.4161253457	-3.1903844293	-3.1911920205
C	-0.8694404244	-5.0089145279	-0.0196306501
H	-0.1774126275	-3.2886816635	1.0690834073

C	-1.0094070274	-5.6197888581	-1.2786971989
H	-0.6334675529	-5.4025444141	-3.4087603292
H	-1.2420043684	-5.5406187824	0.8578682263
O	-1.6073508774	-6.8379983591	-1.2893325434
C	-1.7813270620	-7.4999443652	-2.5199315459
H	-2.4141316745	-6.9174814204	-3.2138955568
H	-2.2800348576	-8.4525748856	-2.2970645331
H	-0.8153174776	-7.7084721912	-3.0145651770
C	-0.5057095379	1.7780826745	0.3968035222
C	-1.3712251545	2.2066199316	-0.6224527338
C	-0.3444582924	2.5735528621	1.5426103074
C	-2.0721678374	3.4066519123	-0.4899657220
H	-1.4892028319	1.6166674689	-1.5326627158
C	-1.0541815224	3.7646383801	1.6790956086
H	0.3464251777	2.2318572218	2.3151414021
C	-1.9203032715	4.1831755430	0.6623018557
H	-2.7374577111	3.7376509626	-1.2907149491
H	-0.9321112993	4.3731554525	2.5782761991
H	-2.4747283613	5.1190263307	0.7669948471
C	-1.5538381288	-0.7942479392	-0.9516875992
O	-2.0915700620	-1.0120280568	-2.0107662606
O	-2.1886256712	-0.8430827718	0.2311286024
C	-3.5817634693	-1.1476757843	0.1877477930
H	-3.9307166685	-1.1283089114	1.2269948204
H	-3.7436034115	-2.1416654562	-0.2546880984
H	-4.1230078011	-0.4008543513	-0.4122419281

(PhO)2P(O)NTf

N	1.9568605423	-0.9418486626	-0.6447922630
S	3.2708262230	-0.1034933612	-0.0329990434
O	1.0332847890	-2.5810784853	1.3489179252
O	2.8948350453	0.3988856304	1.2776146632
O	4.4967960002	-0.8299175458	-0.3096527692
C	3.3063606662	1.3464158663	-1.2294367832
P	0.6926571436	-1.5824091064	0.3229698224
O	-0.1750989226	-2.0968975657	-0.9522081857
O	-0.0746284053	-0.3160275439	0.9356573141
C	-1.3837705773	-2.7892041582	-0.8694099541
C	-0.2883573250	0.9331235502	0.3623341814
C	-1.6703145790	-3.6748418739	0.1721900047
C	-2.2925710607	-2.5647751252	-1.9046948738
C	-0.8267120560	1.0715811259	-0.9168384435
C	0.0312583548	2.0420558434	1.1445200818
C	-2.9032167071	-4.3337976545	0.1684909638
C	-3.5162722602	-3.2368998049	-1.8958795362
H	-2.0278849463	-1.8751634552	-2.7080843622
C	-1.0444163446	2.3575929574	-1.4190920947
C	-0.1987791954	3.3194259122	0.6326276098
C	-3.8271544278	-4.1200788551	-0.8577201530
H	-3.1368535990	-5.0259537965	0.9806710286
H	-4.2300280549	-3.0653606083	-2.7048276889
C	-0.7339805160	3.4813551132	-0.6493396182
H	-1.4644508264	2.4774560716	-2.4203986056

H	0.0503489824	4.1940660186	1.2376847382
H	-4.7862122894	-4.6421612015	-0.8504165913
H	-0.9081640835	4.4830526230	-1.0479440824
H	0.4683456832	1.8825529807	2.1303349293
H	2.1724954889	-1.4785304837	-1.4859083977
H	-1.0733164182	0.1886847303	-1.5049675875
H	-0.9379552519	-3.8416941705	0.9622561551
F	3.2825022240	0.8708618483	-2.4740119835
F	4.4318796359	2.0153895776	-1.0315115917
F	2.2721203376	2.1472196993	-1.0438068088

2a

C	-2.2492051222	0.7800598558	-0.0900928372
C	-0.3569168839	1.9873781470	-0.2759095714
C	0.0614640995	0.6896098059	-0.0892515147
C	-1.1379465949	-0.1056043149	0.0328890475
H	0.2368894120	2.8899358705	-0.4104556463
N	-1.7403006693	2.0460002797	-0.2770603561
C	-1.3777335605	-1.4772431990	0.2338222983
H	-0.5424829531	-2.1753971487	0.3309952086
C	-3.5724278460	0.3258349997	-0.0169229411
H	-4.4151865209	1.0148391345	-0.1127138512
C	-2.6912622918	-1.9308117722	0.3068451321
C	-3.7772333962	-1.0371442453	0.1825557323
H	-2.8901115592	-2.9937698145	0.4626277604
H	-4.7980345053	-1.4219632763	0.2440102611
H	-2.2893322707	2.8853195165	-0.3963152763
C	1.4687320741	0.1861773460	-0.0234814422
H	1.6783750603	-0.3051274727	0.9422499224
H	1.6689900238	-0.5604263464	-0.8111908321
H	2.1943524440	1.0042101143	-0.1464668541

ECA

0 1

C	-0.5251464974	1.8434808647	-0.8087485612
C	-1.9120968820	2.1771040909	2.0637446707
C	-0.9286728218	2.3718953528	3.0500753571
C	-0.4779908841	1.2731018975	3.7772313642
C	-1.0008690186	-0.0181813360	3.5475356628
C	-1.9807487325	-0.2413293458	2.5853635843
C	-2.4224404288	0.8638368708	1.8465276440
C	-2.5799698501	3.0494457772	1.1233240632
C	-3.4454474060	2.2429146141	0.4039904447
N	-3.3547281312	0.9437399479	0.8351341501
H	-4.1040494174	2.5109003253	-0.4197239574
H	-0.5207861656	3.3685820942	3.2370923236
H	0.2906281728	1.4084171171	4.5421317966
H	-0.6171847374	-0.8636487509	4.1220886973
H	-2.3838895662	-1.2370941680	2.3979357159
H	-3.7796400712	0.1171464717	0.4132595321
C	-2.4288603746	4.5353927880	1.0313616142
H	-2.9775629988	4.9387687205	0.1663171793
H	-1.3721597590	4.8304575942	0.9283990466

H	-2.8174152071	5.0387890778	1.9338546358
O	-1.1800961918	0.8302687532	-1.0301486400
C	-0.9273182627	3.0651111459	-1.6492801241
O	-1.7541814994	3.0107209663	-2.5219658887
O	-0.2308938736	4.1683917922	-1.3428960172
C	-0.5456529705	5.3421611775	-2.0981580379
H	-0.3794036004	5.1650770211	-3.1705662440
H	0.1198088189	6.1301476462	-1.7274532603
H	-1.5974553990	5.6226745624	-1.9451008447
C	0.6500537225	1.7813836856	0.0612257547
C	1.5720060402	2.8589287962	0.5529042051
C	2.0322507901	2.0558330509	-0.6184086625
H	0.6433722433	0.8710106226	0.6631244309
H	1.3073203571	3.8993122444	0.3727659058
H	1.9551797742	2.5734753362	-1.5786343095
C	3.0308185782	0.9570622622	-0.6040528337
C	3.5616274876	0.4939499765	-1.8217891691
C	3.4047149196	0.2890447953	0.5677308721
C	4.4204880457	-0.5965401644	-1.8681750555
H	3.2782518021	0.9905494210	-2.7536913378
C	4.2594947943	-0.8135478770	0.5379507608
H	2.9966902651	0.5998156901	1.5310354729
C	4.7658817029	-1.2721945245	-0.6866709794
H	4.8227576191	-0.9646008646	-2.8135259247
H	4.4999649074	-1.3200173565	1.4715616984
H	2.0142217285	2.6518758378	1.5296944432
P	-2.4692428222	-2.2529685619	-0.7607962722
O	-3.6423496811	-1.8008469354	0.0260415733
O	-2.5556350195	-1.7571263765	-2.2861742937
O	-2.2380281040	-3.8413230326	-0.8579174203
N	-0.9472690379	-1.6811393593	-0.2900156971
S	0.1275519013	-2.3459987100	0.7620509594
O	0.9109714373	-1.2760002335	1.3629734653
O	-0.5016471133	-3.4014607535	1.5418874283
H	-0.8301904733	-0.6476136601	-0.4802768798
O	5.5756317110	-2.3511200120	-0.8257420848
C	5.8926734300	-3.1148003319	0.3160199781
H	6.4507662408	-2.5237691693	1.0644269388
H	4.9861650853	-3.5275008645	0.7918198924
H	6.5271755680	-3.9427958257	-0.0269266541
C	-1.4915773933	-1.6364682056	-3.1802661151
C	-1.2600741992	-0.3784583727	-3.7367083387
C	-0.6984442803	-2.7379230731	-3.5000381273
C	-0.1971143513	-0.2262530536	-4.6293547570
H	-1.8820884608	0.4652796177	-3.4385401364
C	0.3672564118	-2.5662189042	-4.3875177528
H	-0.9072789567	-3.7066823085	-3.0466105807
C	0.6215549221	-1.3138104316	-4.9524367606
H	-0.0050661121	0.7565131268	-5.0665710422
H	1.0030130068	-3.4198162942	-4.6330903867
H	1.4568500178	-1.1853065796	-5.6443691240
C	-2.8468613606	-4.8524581122	-0.1112798688
C	-2.0296731840	-5.9129170618	0.2765968637
C	-4.2081779965	-4.8261719303	0.1899652069
C	-2.5942115847	-6.9796872782	0.9770565566

H	-0.9678124175	-5.8788912695	0.0367660495
C	-4.7555987181	-5.8992103249	0.8993901139
H	-4.8177515995	-3.9736417138	-0.1068626340
C	-3.9569757378	-6.9765745601	1.2909760407
H	-1.9609271722	-7.8145261944	1.2858164406
H	-5.8199388890	-5.8872136776	1.1452365880
H	-4.3944506539	-7.8110555759	1.8432624467
C	1.3345245938	-3.1913908472	-0.4021218917
F	1.7506437969	-2.3310776106	-1.3180805151
F	2.3722648807	-3.6220710269	0.3110653396
F	0.7606604731	-4.2328402527	-1.0025873474

IN1Aa

0 1

C	1.5161778329	-2.5310737496	-1.4715279280
C	-1.3834597759	-0.4861872050	2.5688362773
C	-1.8261845254	-0.4366567905	3.9307771301
C	-1.9028060430	0.7827152913	4.5458961994
C	-1.5379010381	1.9944628670	3.8539277095
C	-1.1073244143	2.0070671748	2.5534293226
C	-1.0310201488	0.7506802758	1.8882163480
C	-1.1643993491	-1.5241147420	1.6823235889
C	-0.6246807926	-0.9379552941	0.4202090514
N	-0.6373440110	0.4835713680	0.6605583481
H	0.4383069634	-1.2709801116	0.3974273721
H	-2.0929660772	-1.3546226386	4.4576594881
H	-2.2352135283	0.8540810488	5.5833867344
H	-1.5969164980	2.9403159969	4.3980234741
H	-0.8126834812	2.9242055494	2.0458857833
H	-0.2829876223	1.1680936282	-0.0525145003
C	-1.2977834467	-2.9807758765	1.9290441888
H	-0.3944017904	-3.5080492635	1.5829084273
H	-2.1446608497	-3.3956416972	1.3583580387
H	-1.4582640952	-3.2029122985	2.9922732423
O	1.4948656436	-1.3136470479	-2.0450109869
C	0.3914000154	-3.2291734067	-1.2124064410
C	-1.2422590899	-1.2626933773	-0.9712821375
C	-1.0099416328	-2.7228416817	-1.4074124605
H	0.5424233582	-4.2355561968	-0.8191709933
H	-0.6479028496	-0.6353081716	-1.6462714722
H	-1.7035873556	-3.4006505276	-0.8891846417
H	-1.2985017686	-2.7752733918	-2.4734044684
C	-2.6797604241	-0.7901708502	-1.0516551141
C	-2.9439898703	0.5159581200	-1.5058143704
C	-3.7667891459	-1.5687970244	-0.6449213530
C	-4.2385696149	1.0180389554	-1.5492210518
H	-2.1099501411	1.1483886504	-1.8223329219
C	-5.0785534397	-1.0815526890	-0.6846323202
H	-3.6094369942	-2.5903060069	-0.2925447848
C	-5.3213269482	0.2220961729	-1.1383851982
H	-4.4435634105	2.0299759274	-1.9031099514
H	-5.8957111920	-1.7271588608	-0.3638893639
C	2.8448317765	-3.1180114143	-1.0976512555

O	3.0047543009	-4.1816269751	-0.5488436379
O	3.8408989415	-2.2922664074	-1.4495029380
C	5.1452272056	-2.6545587235	-1.0044725230
H	5.4302640361	-3.6405168776	-1.4001759570
H	5.8212539158	-1.8759662943	-1.3752350633
H	5.1711612174	-2.6880150020	0.0940061448
O	-6.5498808468	0.7915664458	-1.2178795100
C	-7.6770532364	0.0381843899	-0.8373825594
H	-7.6316821671	-0.2626028646	0.2248973966
H	-8.5526734689	0.6837690154	-0.9868966382
H	-7.7924855763	-0.8697965417	-1.4564757562
H	2.2485214142	-0.7589327324	-1.7359432634
N	2.7238597610	0.7741699889	-0.6359747653
P	1.9112101088	2.0870813964	-1.1362084417
S	2.7911604521	0.3380734323	0.8908041730
O	0.4080015554	2.0090533111	-1.2330243175
O	2.3687520529	3.3984929005	-0.2721097372
O	2.6147739833	2.3763814512	-2.5638166327
O	2.0238448038	1.1947423270	1.8125096473
O	2.6448140500	-1.1199899016	1.0269203304
C	2.1581848604	3.4334077827	-3.3433966413
C	2.8238132486	4.6579749061	-3.2770499554
C	1.0536073462	3.2501201668	-4.1765105841
C	2.3799882492	5.7157506497	-4.0747228623
H	3.6721878830	4.7637481423	-2.5999300188
C	0.6190526359	4.3153203497	-4.9688629674
H	0.5508636755	2.2825054412	-4.1866076257
C	1.2795778882	5.5473605058	-4.9207772047
H	2.8965835059	6.6774576624	-4.0309158258
H	-0.2430607621	4.1807663600	-5.6263877816
H	0.9353877458	6.3770792644	-5.5423062274
C	1.5452506562	4.1175151845	0.5749501520
C	1.9485148061	4.2594533228	1.9040621360
C	0.3661097276	4.7073743123	0.1107080042
C	1.1614380911	5.0089017363	2.7796737661
H	2.8600369072	3.7600621167	2.2291717001
C	-0.4189261540	5.4459035379	1.0010413100
H	0.0674014238	4.5691625045	-0.9276112706
C	-0.0260519656	5.6009803143	2.3341926080
H	1.4745738918	5.1208439285	3.8204266059
H	-1.3445204499	5.9042787475	0.6446023749
H	-0.6424917731	6.1827270132	3.0234149718
C	4.5719855557	0.6800517076	1.3376048887
F	4.8101215602	1.9891033056	1.2499176831
F	4.8039336139	0.2804119625	2.5847337700
F	5.4025509743	0.0386878443	0.5177175641

IN1Ab

0 1

C	0.9494758354	2.0374792327	-2.2533513450
C	-3.2010803484	-2.2528055359	-0.3222823117
C	-3.9779383236	-2.7028528596	0.7962662487
C	-3.3150780150	-3.1367061285	1.9091508278

C	-1.8723959576	-3.1465934384	1.9659702331
C	-1.0785821938	-2.7471715476	0.9228177644
C	-1.7456875043	-2.2855291905	-0.2476186088
C	-3.5361521364	-1.7199064644	-1.5461115539
C	-2.2675382599	-1.3126257638	-2.2347735676
N	-1.2242571825	-1.8154476358	-1.3679748027
H	-2.1785878816	-1.7486506802	-3.2453320811
H	-5.0682548459	-2.6783993410	0.7560275135
H	-3.8727059270	-3.4718584168	2.7857070126
H	-1.3940922090	-3.4741398901	2.8919470572
H	0.0064044502	-2.7222838906	0.9998564538
H	-0.1815501273	-1.8446687213	-1.5415688416
C	-4.8893596200	-1.4461477247	-2.0875657440
H	-5.1570824788	-0.3930732541	-1.8911430632
H	-5.6520361675	-2.0764171515	-1.6098252068
H	-4.9307198562	-1.5966419664	-3.1769433382
O	1.8028224221	0.9964494282	-2.3604114152
C	-0.3143589802	1.9608330129	-2.7099706843
C	-2.1918389959	0.2648128600	-2.3871089743
C	-0.8917615371	0.6532342353	-3.1580585977
H	-0.9396154639	2.8514853534	-2.6339546103
H	-0.1101455694	-0.1063978336	-3.0167576118
H	-1.1308762821	0.6565644061	-4.2366651875
C	-2.4003327261	0.9377771967	-1.0483228941
C	-3.5862671255	1.6319702133	-0.7900161918
C	-1.4423630688	0.8764408563	-0.0186147706
C	-3.8442403654	2.2187722574	0.4538016257
H	-4.3310759268	1.7414996082	-1.5840532880
C	-1.6806118102	1.4448688236	1.2238449944
H	-0.4833050981	0.3930795374	-0.1909034309
C	-2.8899179521	2.1142602461	1.4753187971
H	-4.7786609606	2.7580564695	0.6054596747
H	-0.9194998854	1.4029218152	2.0012588538
C	1.4329503068	3.2515755470	-1.5199780401
O	0.7979853589	4.2643384996	-1.3457161929
O	2.6646031026	3.0336306999	-1.0290083315
C	3.1502369945	3.9700834492	-0.0719033392
H	2.4937600481	3.9708338102	0.8093725237
H	4.1501738067	3.6197198821	0.2102828194
H	3.1953401777	4.9818448786	-0.5017687482
O	-3.0396736076	2.6351570899	2.7172222955
C	-4.2000071576	3.3741256319	3.0125589631
H	-4.1016121486	3.7124236105	4.0525504175
H	-4.3021132232	4.2575633119	2.3565934652
H	-5.1147141885	2.7595321589	2.9216004054
P	2.4515443813	-1.6956200839	-0.6107226246
O	1.3421369071	-2.2543317128	-1.4559538230
O	3.8078263391	-1.6914249736	-1.4993706410
O	2.7826898672	-2.6577160165	0.6662367841
N	2.3097821696	-0.2047365305	-0.0112337399
S	1.9021866486	0.2876001412	1.4435804752
O	1.4585528307	1.6833447817	1.3884299939
O	1.0962248052	-0.6804342602	2.2009502074
H	2.1773733522	0.8016091744	-1.4635936991
H	-3.0557389672	0.5234549566	-3.0183692287

C	4.9311526825	-0.9713970017	-1.0968183241
C	5.0733060842	0.3533776652	-1.5124889610
C	5.8835105270	-1.5867278139	-0.2851832622
C	6.1978242772	1.0713210856	-1.1020951934
H	4.2986871520	0.8127961420	-2.1265876684
C	7.0043016794	-0.8558856985	0.1184886533
H	5.7362486286	-2.6215113896	0.0274067526
C	7.1628369137	0.4723368121	-0.2858988967
H	6.3113518173	2.1115851730	-1.4160694364
H	7.7534277097	-1.3288318521	0.7578509725
H	8.0368776975	1.0421507084	0.0375820354
C	3.2353198151	-3.9600962501	0.6216683023
C	4.0372145365	-4.3685407158	1.6913172905
C	2.9162673154	-4.8378521189	-0.4187879875
C	4.5247671788	-5.6759215714	1.7206228111
H	4.2630028279	-3.6466524560	2.4775562243
C	3.4189524393	-6.1415321421	-0.3773628540
H	2.2850072809	-4.4960600009	-1.2394244600
C	4.2201458993	-6.5667537338	0.6857934628
H	5.1514975552	-5.9980519594	2.5557133557
H	3.1776531819	-6.8311512532	-1.1899559985
H	4.6073753595	-7.5877154269	0.7078844271
C	3.5454585595	0.3889465802	2.3400821025
F	4.3536311185	1.2406190024	1.7098654819
F	3.3436416241	0.8237792874	3.5819845763
F	4.1423680403	-0.8028524154	2.3949130861

IN2A

0 1

C	2.0868227045	-1.6232462708	-1.5937701515
C	4.7922510695	0.1017764266	0.6759524207
C	6.1333127085	-0.1944051440	0.9833095194
C	6.4359691025	-0.7212635676	2.2355580475
C	5.4190664152	-0.9611019770	3.1875922253
C	4.0844494968	-0.6722031172	2.9130929122
C	3.7811667922	-0.1354780223	1.6544587935
C	4.1376185493	0.6197685864	-0.4986033429
C	2.7778078326	0.6742608506	-0.1966487832
N	2.5840123907	0.2323828896	1.0999886665
H	-0.4236730141	-1.3395455868	-0.8145484039
H	1.6588827271	0.1756984148	1.5305890640
H	6.9216562135	-0.0180414922	0.2475801754
H	7.4721876036	-0.9557933541	2.4902399698
H	5.6868222659	-1.3777206180	4.1617070753
H	3.2998825801	-0.8563039682	3.6500562022
C	4.8566799182	0.9939087929	-1.7555954644
H	5.2455722591	0.0948280667	-2.2649355296
H	4.2193324878	1.5408828361	-2.4615619485
H	5.7232326232	1.6369878696	-1.5295475321
O	1.0834482977	-1.9563636177	-0.9767823348
C	2.0775447896	-0.6894836182	-2.7726487486
C	1.5658522912	1.1247829608	-0.9655271265
C	1.5737892614	0.7469655016	-2.4623176082

H	1.4043802324	-1.1463784103	-3.5175194676
H	0.7364793557	0.5711683585	-0.5158873968
H	2.1920012126	1.4587173789	-3.0269508754
H	0.5477915954	0.8703188839	-2.8326449442
C	1.2144123771	2.5914490790	-0.7610197947
C	2.1907199443	3.5954664220	-0.6645668293
C	-0.1286402816	2.9704206170	-0.6774406210
C	1.8337121096	4.9288804057	-0.4914133368
H	3.2479777043	3.3247120793	-0.7052108980
C	-0.5061313658	4.3047741210	-0.5030356214
H	-0.9159987744	2.2190683745	-0.7460639708
C	0.4800899971	5.2958263150	-0.4074197799
H	2.5889839048	5.7124724740	-0.4068975429
H	-1.5661019222	4.5452962724	-0.4319402355
C	3.4242438297	-2.2776548379	-1.2215774761
O	4.3750582781	-2.3119919041	-1.9674829937
O	3.3641875851	-2.8267659490	-0.0155036406
C	4.5589837316	-3.4545999092	0.4634514427
H	4.8268463678	-4.3036695284	-0.1828145695
H	4.3325254704	-3.7934329413	1.4799228979
H	5.3844791930	-2.7315946639	0.4821441094
H	3.0860070461	-0.6750298174	-3.1990327716
P	-1.3095973289	-0.5877736206	1.2244903536
O	-0.1984619085	0.2537690119	1.7287384062
O	-1.2687265046	-2.0526966196	1.8729938766
O	-2.8023346875	-0.0860913448	1.5182770882
N	-1.3005695654	-0.8873936971	-0.4457882497
S	-2.2751267308	-0.3390401372	-1.6591013389
O	-1.5337145683	-0.5065400992	-2.9020617775
O	-2.9278717430	0.9073875262	-1.2883274240
O	0.2231232194	6.6179259557	-0.2272493036
C	-1.1165126298	7.0378545028	-0.1300904091
H	-1.6335324800	6.5705739746	0.7280801400
H	-1.6880266209	6.8087897382	-1.0478570546
H	-1.0964037168	8.1261927594	0.0156602520
C	-3.6353401113	-1.6292087956	-1.7099474813
F	-4.3514208704	-1.5784874673	-0.5901487966
F	-4.4196161045	-1.3684766696	-2.7461304078
F	-3.1050158720	-2.8359941385	-1.8424269452
C	-1.8592622628	-3.1945338819	1.3296835571
C	-1.0763775857	-4.0336288495	0.5375543456
C	-3.1950221200	-3.4743747156	1.6079109941
C	-1.6588151088	-5.1879677482	0.0097731238
H	-0.0385566779	-3.7637304377	0.3338922850
C	-3.7635140706	-4.6326704438	1.0716018182
H	-3.7697806488	-2.7850062775	2.2275221326
C	-2.9990963377	-5.4884311072	0.2735164773
H	-1.0606346864	-5.8534739564	-0.6166666199
H	-4.8108138453	-4.8640835080	1.2784425503
H	-3.4489709092	-6.3912125742	-0.1452448381
C	-3.2888370922	1.2231931319	1.6285416112
C	-4.6384044360	1.3917165668	1.3248039903
C	-2.4857405296	2.2899858503	2.0275337716
C	-5.2002946195	2.6643450530	1.4250066228
H	-5.2192741164	0.5304372359	0.9944303472

C	-3.0644956674	3.5602598945	2.1155022658
H	-1.4315276288	2.1329524736	2.2496921406
C	-4.4153426221	3.7535876824	1.8186963751
H	-6.2564287456	2.8059373915	1.1847090043
H	-2.4410114617	4.4032664423	2.4216956587
H	-4.8574865614	4.7494912379	1.8926906765

IN3A

0 1

C	-0.3969860711	1.5892439341	-2.6066640160
C	0.2959293556	-4.2108740105	0.2935367527
C	0.8646209309	-4.8162415843	1.4300773356
C	1.2227337511	-4.0180389663	2.5125969525
C	1.0246279864	-2.6211748042	2.4831653470
C	0.4602960536	-1.9974799690	1.3757756864
C	0.1049911810	-2.7993781381	0.2840612439
C	-0.1801656790	-4.7104465144	-0.9734992971
C	-0.6239669090	-3.6122071107	-1.6762865004
N	-0.4523544692	-2.4533574659	-0.9318401201
H	-1.0443593947	-3.5767295236	-2.6777522804
H	1.0215145650	-5.8978061942	1.4596150184
H	1.6740783206	-4.4733551247	3.3970141098
H	1.3326871045	-2.0093993258	3.3330620848
H	0.3128003213	-0.9207298509	1.3706712474
C	-0.1749720078	-6.1365344470	-1.4258104907
H	0.8473709006	-6.5528921288	-1.4466592147
H	-0.5966001103	-6.2394981731	-2.4373378032
H	-0.7657616702	-6.7795399429	-0.7503628112
O	0.8099423874	1.5188658003	-2.6709191351
C	-1.3327956007	0.5488479105	-3.1641281186
C	-0.8266437741	-1.0878647577	-1.2720305042
C	-0.9030690120	-0.8783475538	-2.7933855708
H	0.0875383080	-1.0971081793	-3.2183751655
H	-1.6308436117	-1.5697275861	-3.2443425424
C	-1.0364112001	2.8181246262	-1.9283947239
O	-2.1683045325	3.1814720994	-2.1012418203
O	-0.1395648508	3.4358860369	-1.1439065138
C	-0.5884493820	4.6008034623	-0.4343985145
H	-1.4205984500	4.3357378410	0.2303216151
H	0.2707744931	4.9401810184	0.1517698677
H	-0.9123546572	5.3693855942	-1.1499619174
P	2.8453159375	0.4935074349	-0.4831752667
O	2.2535613859	-0.6855276719	-1.1371979149
O	3.6986061669	1.4073806429	-1.4955494402
O	3.8403598849	0.2581869805	0.7540738419
N	1.7513895137	1.6316092424	0.1971847448
S	1.3826929525	1.9458468256	1.7774308308
O	0.0948683787	2.6324074738	1.7904718916
O	1.6740609906	0.8001672615	2.6197679672
C	-2.0828524803	-0.6378763830	-0.5503011328
C	-3.3000460059	-1.3254480473	-0.6865174463
C	-2.0562680344	0.5020543884	0.2526487548
C	-4.4497473729	-0.8708259017	-0.0539403528

H	-3.3419337977	-2.2377755323	-1.2872056599
C	-3.2025861669	0.9768218378	0.8987162834
H	-1.1208338635	1.0409276593	0.4048049645
C	-4.4127913327	0.2901676718	0.7418504953
H	-5.4002388078	-1.3987399260	-0.1505539021
H	-3.1252934160	1.8651597618	1.5242768691
O	-5.5844733538	0.6569654155	1.3174169977
C	-5.6094950766	1.8072290817	2.1299309246
H	-4.9399583591	1.7070714652	3.0031751876
H	-5.3214657239	2.7130862108	1.5662511387
H	-6.6428958829	1.9200950367	2.4835862463
H	0.0052189049	-0.4669900623	-0.9182127049
H	1.2131719374	2.2236122888	-0.4537004622
H	-2.3586026930	0.7651426291	-2.8408274313
H	-1.3062376180	0.6601359855	-4.2629367786
C	2.6147868712	3.2764671671	2.2622690687
F	2.5571592071	4.2731044131	1.3809101410
F	2.2837956650	3.7309320336	3.4626624035
F	3.8463802705	2.7882501350	2.2973838096
C	4.0819547511	2.7231083345	-1.2588420114
C	3.3158062998	3.7475417974	-1.8167387826
C	5.2185996882	2.9896927428	-0.4972965700
C	3.7028050953	5.0717575291	-1.6009815527
H	2.4309236388	3.4864506391	-2.3977050497
C	5.5925825233	4.3197003692	-0.2881810247
H	5.7856323559	2.1607298490	-0.0725776900
C	4.8379384092	5.3606056558	-0.8365544071
H	3.1118429672	5.8830077616	-2.0327333300
H	6.4796577982	4.5410798109	0.3095855124
H	5.1352614029	6.3980953285	-0.6690941868
C	4.0967861024	-0.9276484984	1.4551777633
C	4.4207867401	-0.7783767604	2.8016787893
C	4.0644605570	-2.1774086888	0.8386715197
C	4.7294638766	-1.9152626509	3.5507505697
H	4.4114698449	0.2178935203	3.2424038368
C	4.3696761502	-3.3039234909	1.6055744728
H	3.7704799299	-2.2698794974	-0.2054828911
C	4.7068141361	-3.1796243742	2.9550398176
H	4.9821301773	-1.8092665551	4.6083175990
H	4.3224596274	-4.2896593428	1.1393488589
H	4.9388235904	-4.0683229095	3.5458173972

IN4Aa

0 1

C	-2.3146709768	2.6758064224	-0.2897636867
C	-3.3498471826	2.3427552584	0.6279383789
C	-4.3714392767	3.2445680699	0.9560840363
C	-4.3257408523	4.5069853029	0.3703519186
C	-3.2950667124	4.8642409691	-0.5268210406
C	-2.2921947490	3.9603560896	-0.8628590344
C	-1.4729035974	1.5142724438	-0.4291360185
C	-2.0124444432	0.5403228670	0.3918910342
H	-5.1795746208	2.9678836141	1.6349469299

H	-5.1058927377	5.2346059018	0.6066882581
H	-3.2917445809	5.8652528415	-0.9646348641
H	-1.4997001520	4.2412724625	-1.5606957904
N	-3.1355162750	1.0486641476	1.0516102145
C	-4.0764599380	0.2826579798	1.8534233689
H	-4.3836234348	0.9206943930	2.6982536876
C	-0.2539804639	1.4525826376	-1.2930892821
H	0.0998476103	0.4292579007	-1.4576371980
H	-0.4556262376	1.8842276136	-2.2865072483
H	0.5821019307	2.0144861866	-0.8484255516
C	-3.3567580784	-0.9484342296	2.4268566228
H	-2.6209946174	-0.6274047733	3.1796727137
H	-4.0966646778	-1.5912568041	2.9251014248
C	-1.5027968082	-0.8383769244	0.7347742552
C	-2.6346212927	-1.7125522889	1.3261699730
H	-2.1664169208	-2.6249600533	1.7234821370
H	-3.3379805307	-2.0033336727	0.5328387715
C	-5.3253709786	-0.0862265413	1.0640174876
C	-5.3098355569	-0.2160271895	-0.3266960714
C	-6.5293834717	-0.3383869476	1.7409036779
C	-6.4561925967	-0.5947041243	-1.0341886444
H	-4.3898301548	-0.0158648259	-0.8793056549
C	-7.6769592933	-0.7161628911	1.0541968838
H	-6.5696566838	-0.2309763044	2.8290817316
C	-7.6498370881	-0.8489290656	-0.3447608796
H	-6.4048069372	-0.6797798880	-2.1191692367
H	-8.6168827009	-0.9075831250	1.5749688832
O	-8.8152535552	-1.2167845703	-0.9341107650
C	-8.8572206641	-1.3498733725	-2.3354683959
H	-8.1597266781	-2.1279105154	-2.6954630718
H	-9.8831096351	-1.6450392744	-2.5927530177
H	-8.6187817867	-0.3991217949	-2.8456200241
O	-0.4793122918	-0.7931977650	1.7063411900
C	-0.9847347243	-1.5795690450	-0.5084491241
O	0.0775113457	-2.1721136070	-0.5549376876
O	-1.8423834100	-1.5727111972	-1.5181915084
C	-1.4146731767	-2.2209340202	-2.7272233386
H	-0.4378802745	-1.8274225091	-3.0409115509
H	-1.3297731242	-3.3055918877	-2.5664905610
H	-2.1883071102	-2.0024592455	-3.4717916438
H	1.5345511188	-1.3889025154	-0.2941025857
N	2.3314494814	-0.7086405912	-0.3038350087
P	2.5698610250	0.2059725233	1.1036629764
S	2.8784369186	-0.3985472681	-1.8316877556
O	1.4820078544	1.1482729001	1.4682394163
O	2.7836618451	-0.9273390771	2.2198374943
O	4.0359775258	0.8181136427	0.9016342200
O	1.9352852739	-0.9791737746	-2.7773596350
O	3.3569310459	0.9705583907	-1.9340681629
H	-0.0083466962	0.0643251645	1.6656092508
C	4.3911974626	-1.5050571342	-1.9044619446
F	4.0478011148	-2.7478598020	-1.5971202287
F	4.8876542605	-1.4682357889	-3.1336499145
F	5.3116709356	-1.0769598638	-1.0439220190
C	4.4269055942	2.1435635122	0.6927731535

C	5.5584600492	2.3274749130	-0.0996611235
C	3.7539653035	3.2167771347	1.2752936929
C	6.0330448805	3.6225249389	-0.3094053279
H	6.0381350263	1.4580071489	-0.5482285204
C	4.2394280919	4.5083867968	1.0477818851
H	2.8629628730	3.0427853340	1.8772447283
C	5.3754278375	4.7166443010	0.2621257399
H	6.9183298197	3.7762059267	-0.9306790947
H	3.7181255413	5.3578832738	1.4949940584
H	5.7468217798	5.7295436487	0.0924112715
C	3.2764954235	-2.2051742595	1.9557162002
C	2.3567610898	-3.2380228724	1.7821229012
C	4.6507024864	-2.4135161901	1.8636010021
C	2.8360193635	-4.5202682841	1.5068007598
H	1.2917450226	-3.0086677999	1.8414609837
C	5.1149543951	-3.7024417196	1.5876218434
H	5.3324929281	-1.5723656090	1.9948037411
C	4.2117628575	-4.7542395102	1.4084115559
H	2.1283079395	-5.3393154365	1.3603620280
H	6.1894825046	-3.8821699177	1.5078347551
H	4.5805651894	-5.7584457851	1.1883619747

IN4Ab

0 1

C	2.4699972599	2.4699289816	-0.7620699433
C	3.6815020671	1.9435149919	-1.2940831463
C	4.6852248321	2.7690756578	-1.8183109298
C	4.4456142485	4.1409256141	-1.8342971290
C	3.2410868309	4.6815190023	-1.3328466660
C	2.2548821667	3.8598826600	-0.7959877233
C	1.6968892986	1.3570148826	-0.2717429425
C	2.4473363876	0.2222201355	-0.5188976678
H	5.6253826935	2.3580641110	-2.1898343405
H	5.2071415129	4.8124081361	-2.2382437068
H	3.0880373630	5.7629553980	-1.3635294286
H	1.3279197051	4.2854575325	-0.4041362209
N	3.6360619802	0.5713453550	-1.1619892862
C	4.7500861239	-0.3183717906	-1.4390896812
H	5.2002660754	0.0103768766	-2.3898300951
C	0.3298660766	1.4438972979	0.3313470277
H	0.1569388047	0.6213454199	1.0335498636
H	0.2041019959	2.3807746492	0.8950145251
H	-0.4568400235	1.4208803253	-0.4407866139
C	4.2074942919	-1.7422127929	-1.6428589997
H	3.6470248705	-1.7811471834	-2.5883950571
H	5.0577743556	-2.4347155371	-1.7284465742
C	2.0965885878	-1.2306176286	-0.2483111835
C	3.3186096155	-2.1549809452	-0.4780320225
H	2.9617856189	-3.1906570055	-0.5849147924
H	3.8854228776	-2.1133341956	0.4638476407
C	5.8236452114	-0.2467019320	-0.3616167388
C	5.5317604319	0.1402033959	0.9483317802
C	7.1455954530	-0.6065973823	-0.6690593427

C	6.5214602419	0.1625146827	1.9370601520
H	4.5143821179	0.4358696759	1.2124390183
C	8.1409346077	-0.5902700954	0.3005922052
H	7.4016179448	-0.9006330547	-1.6915629765
C	7.8355744418	-0.2056541976	1.6176496782
H	6.2531716137	0.4724808280	2.9464064205
H	9.1712706335	-0.8636324607	0.0660084202
O	8.8671184722	-0.2157543953	2.4988353972
C	8.6265082033	0.1695463990	3.8322810525
H	7.8900103081	-0.4911902953	4.3242506882
H	9.5859096169	0.0895139751	4.3604454980
H	8.2656008131	1.2117279471	3.8996873643
O	1.6864171487	-1.4406043796	1.0808075008
C	0.9619733812	-1.6235614706	-1.2068456737
O	-0.0935602324	-2.1078730737	-0.8281008291
O	1.2334564248	-1.3877901659	-2.4747502708
C	0.1737686771	-1.5639449919	-3.4323848125
H	0.6306531987	-1.3852362664	-4.4118872313
H	-0.2303422315	-2.5844851740	-3.3760836681
H	-0.6210435103	-0.8301996848	-3.2364785378
H	-1.5086401565	-1.5204621547	-0.1857878378
N	-2.3285983123	-0.9209582029	0.0372946527
P	-3.0181542651	-0.1657676444	-1.3168646646
S	-2.3751995935	-0.5325612102	1.6385124627
O	-2.1025732197	0.6473230021	-2.1457068120
O	-3.5964222178	-1.4266349702	-2.1382042739
O	-4.3327005232	0.5216840845	-0.7092954428
O	-1.1755952118	-1.1143612278	2.2355308947
O	-2.7815752181	0.8468457409	1.8433343240
H	0.7200962368	-1.4942993781	1.1492811401
C	-3.7960453515	-1.5917766260	2.2522921727
F	-3.5706711071	-2.8623200581	1.9516059378
F	-3.8889113418	-1.4517182456	3.5668651571
F	-4.9320172699	-1.1970945752	1.6835729458
C	-4.5936578314	1.8764987067	-0.4778700835
C	-5.4066141986	2.1627999724	0.6169109149
C	-4.1028941183	2.8781918665	-1.3141248299
C	-5.7432508221	3.4913985828	0.8777989917
H	-5.7481329992	1.3458935709	1.2520892447
C	-4.4445408018	4.2047278585	-1.0327358865
H	-3.4574631098	2.6226869607	-2.1537802799
C	-5.2632664575	4.5158602901	0.0554464013
H	-6.3779477857	3.7263420630	1.7352198869
H	-4.0628460810	4.9997696460	-1.6773764790
H	-5.5247483263	5.5551762182	0.2653693904
C	-4.2229252608	-2.5192404682	-1.5393805699
C	-3.4649021709	-3.6611824135	-1.2818010210
C	-5.5774613279	-2.4515355210	-1.2194575624
C	-4.0855250551	-4.7650732624	-0.6940273761
H	-2.4013191762	-3.6629784527	-1.5263564665
C	-6.1857346543	-3.5622879151	-0.6289119768
H	-6.1316732385	-1.5350692531	-1.4242351003
C	-5.4440688541	-4.7175546410	-0.3660482628
H	-3.5006119265	-5.6629918330	-0.4829369772
H	-7.2462735667	-3.5206793574	-0.3706819672

H	-5.9244662706	-5.5814769843	0.0981903517

3A

0 1

C	-2.7852512013	1.1001155188	0.6427373416
C	-2.3309506805	-0.2424414616	0.5972262272
C	-1.0893343385	-0.6164007997	1.1231361659
C	-0.2710105191	0.3920053378	1.6278212746
C	-0.6839966509	1.7380084828	1.6348560730
C	-1.9431456528	2.0961103903	1.1631132469
C	-4.1307699432	1.1352740803	0.1052655624
C	-4.4558998769	-0.1557061064	-0.2397183475
H	-0.7537679963	-1.6508128022	1.1324065094
H	0.7172081608	0.1363126156	2.0137987761
H	0.0088354007	2.4971890394	1.9973867085
H	-2.2710931203	3.1379086731	1.1886070499
N	-3.3476622857	-0.9978396035	0.0087086426
C	-3.3344233762	-2.3334419925	-0.4037338369
C	-4.5040834917	-2.9880864889	-0.5731257098
C	-5.7009075479	-0.7902734479	-0.8068388817
C	-5.7883327791	-2.2643974527	-0.3182763657
H	1.0449420076	0.4210801805	-1.2698870460
N	2.0785852726	0.2211647067	-1.0560610813
P	3.0016021676	1.2177029978	-0.0747954392
S	2.6373154157	-1.1012476928	-1.8751143386
O	2.2481293410	2.1962709618	0.7362290578
O	3.8343311318	0.0872283561	0.7465085865
O	4.0744225431	1.8392337193	-1.1168155630
O	1.6714712039	-1.3931333415	-2.9299426504
O	4.0674263432	-1.0141058523	-2.1289464235
O	-0.3990721314	0.3529963206	-1.9859678502
H	-0.0763442192	-0.0304907249	-2.8177897468
H	-0.8417110096	-0.4142704638	-1.5691344504
C	2.4443270195	-2.5181944702	-0.6321152708
F	1.4585315467	-2.2732490130	0.2221264587
F	2.1633643244	-3.6254644812	-1.3179351079
F	3.5708963594	-2.7047860087	0.0389652318
C	5.1574820148	0.2626646701	1.1432138237
C	6.1294564962	-0.5467251066	0.5582195767
C	5.4816561007	1.2384837714	2.0856226520
C	7.4640555490	-0.3655912870	0.9283980276
H	5.8265028042	-1.2761820346	-0.1925555637
C	6.8215396518	1.4173638593	2.4354372491
H	4.6900735982	1.8563269231	2.5125390073
C	7.8132472246	0.6177802256	1.8594000537
H	8.2374890136	-0.9894415453	0.4742283325
H	7.0899758681	2.1908200888	3.1581837227
H	8.8601174114	0.7633781743	2.1345864985
C	5.0530424249	2.7742345968	-0.7881597873
C	6.3594901734	2.4870803201	-1.1824170666
C	4.7362156299	3.9511353304	-0.1074651116
C	7.3751788453	3.3917449878	-0.8719533921
H	6.5576455592	1.5508921337	-1.7047484680

C	5.7655724368	4.8460926955	0.1977452666
H	3.7046976263	4.1389292583	0.1903327937
C	7.0832599044	4.5706090723	-0.1777969449
H	8.4027810457	3.1683811998	-1.1676743364
H	5.5301361589	5.7676516913	0.7354748422
H	7.8818897599	5.2740837265	0.0677708598
C	-6.9755501150	-0.0423210694	-0.4744815975
C	-7.3052878640	0.2683151859	0.8489519467
C	-7.8765233320	0.3327310154	-1.4816595122
C	-8.4866446101	0.9405384416	1.1707050975
H	-6.6123050555	0.0042974116	1.6517565823
C	-9.0582792938	1.0040368013	-1.1818303084
H	-7.6418613495	0.1036301190	-2.5248075097
C	-9.3740931831	1.3161463606	0.1498530591
H	-8.7009066177	1.1710754178	2.2138190663
H	-9.7574290801	1.3036230512	-1.9645333293
O	-10.5418420444	1.9755572762	0.3524679449
C	-10.9081468902	2.3271195744	1.6663646544
H	-11.8715820366	2.8489613394	1.5949109072
H	-11.0294257096	1.4379113330	2.3111610129
H	-10.1681918663	3.0029021925	2.1320456106
H	-6.0069910412	-2.2579250558	0.7658724935
H	-5.6064608846	-0.8255131547	-1.9079793282
H	-6.6355274833	-2.7654410952	-0.8073847702
C	-4.9433442998	2.3869564632	-0.0229409831
H	-5.7972588562	2.2665542646	-0.6996174725
H	-5.3405533698	2.7148396084	0.9532376581
H	-4.3149362495	3.2063561838	-0.4078568521
H	-4.4918041708	-4.0047459684	-0.9646071603
C	-2.0523850681	-2.9114532394	-0.8911798766
O	-1.1239029080	-2.2557639817	-1.3228456117
O	-2.0421558862	-4.2447824967	-0.8567267491
C	-0.8852958837	-4.8855012477	-1.4063972997
H	-0.0084946885	-4.6984297438	-0.7721884720
H	-1.1180939026	-5.9562678533	-1.4321162689
H	-0.6701199746	-4.5053530626	-2.4150997703

4A

0 1

C	2.5343811817	0.7285851213	2.2188040089
C	3.2770711036	-0.4134643173	1.7960398508
C	3.3820589317	-1.5629689236	2.5899046495
C	2.7376559039	-1.5540036146	3.8224431252
C	2.0027360442	-0.4301684925	4.2623091042
C	1.8943847634	0.7066144191	3.4708122461
C	2.6061074027	1.7105343864	1.1703418758
C	3.3661944318	1.1392875308	0.1555232834
H	3.9311863498	-2.4433829770	2.2510340147
H	2.7984853203	-2.4374199791	4.4625224507
H	1.5103761866	-0.4619477763	5.2373177123
H	1.3045792939	1.5630274771	3.8039434719
N	3.7845257929	-0.1366156792	0.5471693075
C	4.4919448374	-1.0240873737	-0.3714683895

H	4.9846670449	-1.7928673542	0.2435341893
C	5.5757177444	-0.1729940484	-1.0572931284
H	6.3099076742	0.1291567299	-0.2880016714
H	6.1309987944	-0.7627620775	-1.7986162658
C	3.9043548555	1.6592968256	-1.0923516874
C	4.9704043666	1.0562312836	-1.6752802865
C	3.5175002028	-1.6966877797	-1.3433987212
C	2.2910303336	-2.1740943354	-0.8605343444
C	3.7741953163	-1.8354289391	-2.7187916104
C	1.3290452323	-2.7240933439	-1.7120583444
H	2.0245278212	-2.0648417459	0.1897278788
C	2.8308892122	-2.3925261550	-3.5768399349
H	4.7135525942	-1.4840325009	-3.1473111995
C	1.5863923518	-2.8231348221	-3.0872501038
H	0.3727610247	-3.0267109146	-1.2890081085
H	3.0204497860	-2.4728860580	-4.6485754079
O	0.7082524464	-3.2942125053	-3.9993391376
C	-0.6460223575	-3.4520344288	-3.6199196145
H	-1.0760314973	-2.4977975984	-3.2755629827
H	-1.1842919196	-3.7790323891	-4.5188531543
H	-0.7704576086	-4.2152537072	-2.8328011648
H	-0.4690628980	-0.1919505217	-1.3255340524
N	-1.2545607798	-0.5031837619	-0.6710191547
P	-0.8521681924	-0.5255380521	0.9586682426
S	-2.7597863891	-0.5655460068	-1.3568341976
O	0.3559760323	-1.2907816769	1.3143144639
O	-0.7475143725	1.0133492310	1.4575278906
O	-2.2642812471	-1.0789434260	1.5248740377
O	-3.7511034560	0.2175282243	-0.6357629261
O	-2.5575901862	-0.4256012452	-2.7934089606
O	0.8561401295	0.3732773186	-1.9962894304
H	1.1768917698	1.2958268607	-1.9128767294
H	1.6012274903	-0.1928615144	-1.7384907987
C	-3.2570760038	-2.3878176462	-1.0756076092
F	-4.2266124062	-2.4610743736	-0.1791579527
F	-3.6902734663	-2.8741442036	-2.2340679461
F	-2.2206825570	-3.1178989389	-0.6669411226
C	-1.6301373421	2.0134580992	1.0535499118
C	-1.4234854217	2.6494224267	-0.1707652420
C	-2.6840197308	2.3696487973	1.8922680442
C	-2.3058322927	3.6560753789	-0.5670140691
H	-0.5835415205	2.3532541083	-0.7972758367
C	-3.5584751412	3.3794891395	1.4846421178
H	-2.8148316493	1.8506377872	2.8418552325
C	-3.3754378500	4.0199929223	0.2557318899
H	-2.1546542409	4.1536085221	-1.5274238237
H	-4.3923759741	3.6611743919	2.1315919618
H	-4.0674795454	4.8031912361	-0.0612022121
C	-2.7002127812	-0.9689726455	2.8360361263
C	-4.0681815897	-0.7692541703	3.0210947168
C	-1.8130839293	-1.0462885952	3.9113775515
C	-4.5600474895	-0.6437391368	4.3214865484
H	-4.7098308218	-0.7005040974	2.1418537782
C	-2.3223294097	-0.9068213489	5.2058230330
H	-0.7480547303	-1.2085936985	3.7342619751

C	-3.6899225396	-0.7075040779	5.4159211146
H	-5.6295954680	-0.4866522334	4.4794516202
H	-1.6381830063	-0.9596761148	6.0560300015
H	-4.0784642219	-0.6025559830	6.4311905317
C	2.0295418602	3.0901865482	1.2366219957
H	2.6901131563	3.8366608771	0.7704290596
H	1.0552219584	3.1579038222	0.7311423449
H	1.8786002118	3.3907637860	2.2836933866
C	3.2060631968	2.7706751855	-1.7890663027
O	1.9923891085	2.8841367310	-1.8476502135
O	4.0327692799	3.6197927757	-2.3987782715
H	5.3724478123	1.4528135728	-2.6090549803
C	3.4226024177	4.6502519690	-3.1828357541
H	2.7891158470	5.2896107193	-2.5512357725
H	4.2471945850	5.2304239479	-3.6126885275
H	2.7997608321	4.2140521529	-3.9775606017

TS1Aa

0 1

C	1.1843157072	-3.2074103785	-1.2788945475
C	-2.5565352534	-1.7010139707	2.5086817957
C	-3.7791937329	-1.9730113275	3.1772356912
C	-4.5888648120	-0.9145710030	3.5261831037
C	-4.2043163260	0.4311549925	3.2392166516
C	-3.0082174565	0.7409756830	2.6229055770
C	-2.1715086382	-0.3338764896	2.2535554975
C	-1.5611121078	-2.5200861785	1.9422199574
C	-0.6564168953	-1.6415994991	1.2653247198
N	-0.9849838634	-0.3311548581	1.6125127223
H	0.3745579320	-1.8947673258	1.0180001316
H	-4.0745718520	-3.0028904782	3.3896699755
H	-5.5402268579	-1.0953503861	4.0309297526
H	-4.8804537032	1.2410497762	3.5237761672
H	-2.7221788892	1.7697708095	2.4020499384
H	-0.5212494241	0.4842644639	1.1609519772
C	-1.4713456167	-4.0051356180	1.9949041991
H	-0.4576993213	-4.3546306958	1.7599890378
H	-2.1661012565	-4.4805978575	1.2797686416
H	-1.7486026080	-4.3707302957	2.9956734845
O	1.2559814735	-2.0268939372	-1.9231569436
C	0.0051430451	-3.7844303398	-0.9832303497
C	-1.4447914266	-1.7091068664	-0.6652888775
C	-1.3262019294	-3.1193809180	-1.1926121660
H	0.0441706060	-4.7820230243	-0.5470637314
H	-0.6279910257	-1.0729975114	-1.0067033751
H	-2.1364733294	-3.7591769211	-0.8168362440
H	-1.5230095171	-3.0077493161	-2.2792666841
C	-2.7210734302	-1.0273617918	-0.6003354835
C	-2.7669364797	0.3766923682	-0.7949651985
C	-3.9245925905	-1.6828369884	-0.2673782894
C	-3.9532148838	1.0759768272	-0.6784322002
H	-1.8446171377	0.9129826553	-1.0174207873
C	-5.1200829945	-0.9878252640	-0.1293393624

H	-3.9242752707	-2.7593046971	-0.0903811478
C	-5.1391607800	0.4060806345	-0.3232987928
H	-3.9893306258	2.1551291680	-0.8324490408
H	-6.0228788013	-1.5282200181	0.1501426580
C	2.4565420703	-3.8953706226	-0.8760998530
O	2.5039401360	-4.9361071215	-0.2634837692
O	3.5276972665	-3.1987281459	-1.2714554495
C	4.7839052023	-3.6478597790	-0.7682529850
H	4.9639987547	-4.6955904035	-1.0499331150
H	5.5415376283	-2.9894190042	-1.2068855979
H	4.7941482073	-3.5615169659	0.3279940234
O	-6.2384707524	1.1721217011	-0.1868005483
C	-7.4487864748	0.5789408919	0.2353918868
H	-7.3388094768	0.1014099494	1.2248669495
H	-8.1852946494	1.3895226454	0.3052222373
H	-7.8088288706	-0.1730481079	-0.4881080236
H	1.9293890922	-1.4225151791	-1.5074406508
N	2.3495772898	-0.0772437682	-0.4053997337
P	1.4993414945	1.2867972827	-0.6700389458
S	2.7985326346	-0.4759064122	1.0775447266
O	0.1568594428	1.4754331707	-0.0198223207
O	2.5094950779	2.5246705987	-0.3772531660
O	1.3662290629	1.2961370984	-2.2944751205
O	2.2317779780	0.3839092821	2.1297010609
O	2.7195498468	-1.9335272356	1.2512969785
C	2.0949935941	3.7062366361	0.2190149990
C	1.8630825910	3.7359308636	1.5950179308
C	1.9510508316	4.8463140606	-0.5715498025
C	1.4783657932	4.9415448797	2.1849349928
H	1.9909473328	2.8166214514	2.1691455885
C	1.5664701732	6.0472175002	0.0326696033
H	2.1516637593	4.7795299980	-1.6425365877
C	1.3277996125	6.0966936061	1.4091371394
H	1.2964446371	4.9784380554	3.2617892199
H	1.4553575819	6.9472853761	-0.5768337371
H	1.0282981874	7.0364952099	1.8786426095
C	0.1352246895	1.3829915846	-2.9252943199
C	-0.3505710333	0.2575915983	-3.5935957948
C	-0.5945004572	2.5738999977	-2.8887550248
C	-1.5897792418	0.3315932614	-4.2356741732
H	0.2386898215	-0.6596428361	-3.5742923107
C	-1.8331673995	2.6325424810	-3.5316339931
H	-0.1982099959	3.4315555923	-2.3429645617
C	-2.3348490035	1.5138413529	-4.2046348602
H	-1.9782098810	-0.5468776290	-4.7566159375
H	-2.4112591583	3.5591997169	-3.5014042225
H	-3.3068018718	1.5633684522	-4.7001550761
C	4.6288395760	-0.0920017831	1.0877238222
F	4.8163060343	1.2209979259	1.0159276362
F	5.1669463078	-0.5504548520	2.2156409324
F	5.2365596315	-0.6731117978	0.0536118860

TS1Ab

0 1

C	-1.2725041752	-2.1026417488	1.9561518986
C	0.5235285207	-3.8314767289	-2.0656101213
C	0.8337978031	-5.1659752715	-2.4359303936
C	-0.1337886610	-6.1363834200	-2.2708507031
C	-1.4133304154	-5.8150085986	-1.7297044480
C	-1.7562159036	-4.5269964457	-1.3573382852
C	-0.7803296946	-3.5285865024	-1.5448965242
C	1.2546239677	-2.6211638853	-2.0821038284
C	0.4164890318	-1.6270627624	-1.4771066477
N	-0.8304612547	-2.2059199513	-1.2579515969
H	0.4703430734	-0.5425847190	-1.6072809318
H	1.8175776428	-5.4144222887	-2.8403378305
H	0.0779679199	-7.1712145289	-2.5476923926
H	-2.1425223912	-6.6175815702	-1.5957596003
H	-2.7174700527	-4.2867704759	-0.9010375961
H	-1.6641844452	-1.7300934273	-0.8867204164
C	2.6364916112	-2.4205184623	-2.5876192548
H	2.8814481025	-1.3584015484	-2.7070684070
H	2.7661181991	-2.9276656269	-3.5566705594
H	3.3779512484	-2.8504241846	-1.8910402627
O	-0.8700555258	-0.8654975813	2.3179548797
C	-0.4208131929	-3.0301278832	1.4863506062
C	1.3691197650	-1.5422025700	0.3948827855
C	1.0263562089	-2.7972715234	1.1638474583
H	-0.8398239661	-4.0176553290	1.2988922089
H	0.7162049082	-0.6946728827	0.5951595524
H	1.4356394326	-3.6844593115	0.6564203049
H	1.6033243594	-2.7100057921	2.1064944028
C	2.7651064447	-1.1657426676	0.2201666998
C	3.0909385728	0.1415382102	-0.2201941670
C	3.8262342730	-2.0485095429	0.5062049525
C	4.4075230713	0.5366864047	-0.3557617176
H	2.2807598040	0.8388351673	-0.4426582831
C	5.1570990251	-1.6513337746	0.3928168505
H	3.6180782763	-3.0634263002	0.8478712403
C	5.4571314778	-0.3485682257	-0.0414577144
H	4.6617087666	1.5464748125	-0.6795980613
H	5.9480334066	-2.3564822811	0.6452182164
C	-2.7257719110	-2.4572665080	2.0531465900
O	-3.2027288691	-3.4983158289	1.6573652036
O	-3.4366878693	-1.4723685478	2.6111508074
C	-4.8522944612	-1.6470677783	2.6010577063
H	-5.1332453187	-2.5643258450	3.1390358107
H	-5.2725388909	-0.7627791886	3.0920297803
H	-5.2116097897	-1.7146608740	1.5641894096
O	6.7065171280	0.1376702491	-0.1779142554
C	7.8108246776	-0.6885521159	0.1254945102
H	7.8402540372	-1.5823148430	-0.5221446459
H	8.7099872401	-0.0857147684	-0.0546701720
H	7.7984987373	-1.0117477652	1.1811530739
H	-1.4910794465	-0.1747686141	1.9682598401
N	-1.9682403378	1.0133893281	0.6635230902
P	-0.6800172419	1.9423602901	0.2812013706
S	-2.9549335037	0.5261212043	-0.4816485158

O	0.3529247516	1.4162519554	-0.6656185221
O	-1.2920785085	3.3825535446	-0.1672360001
O	-0.0701278243	2.2387826895	1.7638442905
O	-2.6510523530	1.0163415251	-1.8327855232
O	-3.2184950671	-0.9269233517	-0.3376523687
C	-0.7067448025	4.1831928725	-1.1345481704
C	-0.7536796069	3.8012037109	-2.4767105824
C	-0.1102329746	5.3810535264	-0.7384327477
C	-0.1833273039	4.6416138542	-3.4346461315
H	-1.2407251217	2.8612456351	-2.7391289926
C	0.4545631320	6.2143699363	-1.7087797008
H	-0.1085780175	5.6481865828	0.3200188055
C	0.4225343241	5.8452339634	-3.0565320319
H	-0.2156829429	4.3536528638	-4.4882576822
H	0.9194250933	7.1563188089	-1.4076977969
H	0.8646467762	6.4974240946	-3.8131954691
C	1.2815507886	2.1560176507	2.0519938603
C	1.7243333314	1.1047375218	2.8570971432
C	2.1704182804	3.1190207044	1.5680651401
C	3.0794678863	1.0261739518	3.1881904952
H	0.9990666307	0.3618097979	3.1913451206
C	3.5221893360	3.0284048072	1.9070937435
H	1.8004555305	3.9210599411	0.9271959507
C	3.9806916858	1.9844090680	2.7164571267
H	3.4331990259	0.2024686894	3.8128633159
H	4.2222942263	3.7772611724	1.5291095853
H	5.0406204782	1.9141464112	2.9701918752
C	-4.5691066825	1.3426978207	-0.0194083658
F	-4.4515498298	2.6557971339	-0.1698188121
F	-5.5370274641	0.8914516998	-0.8129812260
F	-4.8899451928	1.0779442415	1.2461777538

TS2Aa

0 1

C	-0.6039931626	-0.9026314863	2.0587724730
C	-2.4834472587	-3.6235588625	-0.1001687516
C	-2.1434301510	-4.9801043053	-0.0764779183
C	-1.4318243709	-5.5072811119	-1.1557837790
C	-1.0639159245	-4.6999215377	-2.2485838872
C	-1.4038405320	-3.3483356875	-2.2973306280
C	-2.1126908302	-2.8345066447	-1.2112710234
C	-3.1717368506	-2.7347755677	0.8516453188
C	-3.2496501169	-1.4624259083	0.1747412867
N	-2.5856584391	-1.5390255909	-0.9874128200
H	-2.2981808144	-2.1914070230	1.6772433623
H	-2.4208163863	-5.6122654091	0.7698529127
H	-1.1523360569	-6.5632174551	-1.1528757259
H	-0.4986616387	-5.1380771851	-3.0740042547
H	-1.1064324254	-2.7083776431	-3.1270854596
H	-2.2599407088	-0.7286258314	-1.5145950728
O	-0.4093448883	-0.1529357618	1.0655863379
C	-1.8742921206	-1.0548110877	2.6343911585
C	-3.6966749288	-0.2152428480	0.8455947795

C	-2.9132232610	-0.0311580735	2.2495624589
H	-4.7477601295	-0.3898433270	1.1247122866
H	-3.6752659394	0.0391595689	3.0371491271
H	-2.4271672839	0.9492999056	2.1745222183
C	-3.6531085306	1.0064304336	-0.0545943817
C	-4.8372135393	1.5931817872	-0.5307335208
C	-2.4358928823	1.5614685485	-0.4712024333
C	-4.8033571069	2.6872654689	-1.3906904798
H	-5.8053498775	1.1865356542	-0.2236300474
C	-2.3801312073	2.6487819663	-1.3445533918
H	-1.5087052454	1.1337360151	-0.1005075523
C	-3.5720821773	3.2216660198	-1.8105545366
H	-5.7218598688	3.1475843032	-1.7594321627
H	-1.3993777322	3.0219291690	-1.6400133444
O	-3.6263734838	4.2788385955	-2.6549793091
C	-2.4108941580	4.8346030154	-3.1135698029
H	-1.7954110716	5.2180395590	-2.2811064302
H	-2.6791610454	5.6663944670	-3.7780290832
H	-1.8110838642	4.0981135413	-3.6759943025
H	0.7476911453	0.1931443547	0.2352205855
N	1.4032500148	0.5625573558	-0.5605710513
S	1.1514931311	-0.2371884715	-1.9696982880
O	0.9562201050	3.1269843896	-1.3137281362
O	2.0498921607	0.2621221627	-3.0025988374
O	-0.2707958484	-0.4331922449	-2.2555851200
P	1.7968478134	2.2132204644	-0.5124564324
O	1.8358505047	2.4536197653	1.0902006203
O	3.3582667473	2.3062354290	-0.8738790713
C	0.7389538848	2.6239198382	1.9288423063
C	4.3222396028	1.3883572376	-0.4655062442
C	0.7689932692	1.9424564278	3.1454831144
C	-0.3246331777	3.4574598007	1.5846144896
C	4.7248749899	1.3333159683	0.8696960466
C	4.8854375379	0.5574647042	-1.4326324144
C	-0.2934949801	2.0957921843	4.0358316939
C	-1.3867493935	3.5941898598	2.4826289596
H	-0.3199749933	3.9707454186	0.6241170028
C	5.7143775884	0.4179674283	1.2384607183
C	5.8742514867	-0.3513044529	-1.0501392311
C	-1.3749004315	2.9210665610	3.7067670510
H	-0.2821318017	1.5579245499	4.9864501486
H	-2.2322471651	4.2305273698	2.2126424857
C	6.2897439493	-0.4259240238	0.2828322717
H	6.0399145219	0.3696104817	2.2804040225
H	6.3171514786	-1.0106051961	-1.8001276448
H	-2.2099445939	3.0324602292	4.4016520765
H	7.0638717486	-1.1383890688	0.5765209401
H	4.5194286331	0.6186328471	-2.4573079587
C	1.7777345515	-1.9411721350	-1.4658554722
F	1.0051157853	-2.4538088022	-0.5123061727
F	3.0254194342	-1.8726604478	-1.0219375176
F	1.7349208672	-2.7255621894	-2.5378167290
H	4.2619294983	1.9993836330	1.5972307586
H	1.6092713878	1.2810573545	3.3583672982
C	-4.2143994601	-3.2385598582	1.8288030103

H	-5.0949053467	-3.6368772708	1.3008434095
H	-3.7971858512	-4.0438482371	2.4517968778
H	-4.5495142850	-2.4409025872	2.5073797467
H	-1.9460199896	-1.5862757408	3.5854357986
C	0.5587779073	-1.7312817480	2.5812057284
O	0.4628609638	-2.6720620197	3.3317124411
O	1.7150147483	-1.2763358569	2.0767970907
C	2.8944419821	-2.0198100108	2.3776381473
H	3.7163474651	-1.4965185895	1.8757608385
H	3.0618197670	-2.0624028628	3.4643231408
H	2.8033756553	-3.0474626721	1.9951238804

TS2Ab

0 1

C	-0.1752145955	1.3974641043	2.1916855559
C	-1.2705469304	3.4696527420	-1.2002806336
C	-0.9413385105	4.7886230308	-1.5388111416
C	0.1100534414	4.9990102706	-2.4305414761
C	0.8310885318	3.9196333532	-2.9791720129
C	0.5138073850	2.5992737894	-2.6661605763
C	-0.5478334872	2.4018536037	-1.7827642259
C	-2.2585234412	2.8746354384	-0.3008658987
C	-2.1106338413	1.4568341343	-0.4663019469
N	-1.0831822563	1.2116674825	-1.2931348322
H	-1.5370968015	2.5404710240	0.9651091850
H	-1.4864836066	5.6315108130	-1.1082674109
H	0.3888972614	6.0195852338	-2.7025636254
H	1.6630447353	4.1207920357	-3.6574869342
H	1.0899797261	1.7604257269	-3.0561525844
H	-0.6627535343	0.2660850551	-1.4582026226
O	0.0843050273	0.1800216947	1.8243986173
C	-1.4898817205	1.8593611192	2.1897047624
C	-2.6857869600	0.3980706690	0.4290349280
C	-2.6226130820	0.8837446290	1.9165665647
H	-2.0144527081	-0.4685261849	0.3442598878
H	-3.5669963413	1.3740974609	2.1849438603
H	-2.5244476790	-0.0083329584	2.5519391001
C	-4.0628837379	-0.0775252802	-0.0161409903
C	-4.3101803677	-0.2846733607	-1.3852865769
C	-5.0733670535	-0.4193115263	0.8846726111
C	-5.5160722927	-0.8079942805	-1.8329682927
H	-3.5340586497	-0.0429938874	-2.1150838498
C	-6.2958104459	-0.9480665662	0.4525696871
H	-4.9182741297	-0.3017780288	1.9573664461
C	-6.5253778392	-1.1469590804	-0.9151005213
H	-5.7057664050	-0.9679621130	-2.8957837294
H	-7.0540517714	-1.2020009696	1.1926936714
O	-7.6691816429	-1.6516051080	-1.4387078395
C	-8.7132771764	-2.0203046358	-0.5679835900
H	-9.0842104330	-1.1587429832	0.0161209956
H	-9.5280283731	-2.4012909666	-1.1976128019
H	-8.4007820550	-2.8148254935	0.1337016012
H	0.9936432815	-0.0135293373	1.3604376047

N	1.9261101575	-0.4819933962	0.1758639181
S	2.7428650991	0.7522990569	-0.4613284385
O	0.0652085679	-1.1753398101	-1.6584755269
O	3.0095934620	0.6022364640	-1.8967066174
O	2.2164776266	2.0222954340	0.0537644952
P	1.1805859236	-1.6119695877	-0.7594873342
O	0.6889158707	-2.7015024861	0.3525861735
O	2.2617910480	-2.4531236627	-1.6041077339
C	-0.6132324512	-2.8558890092	0.8011882191
C	3.5702321321	-2.7254305696	-1.2373620876
C	-0.8291746537	-2.8177182527	2.1795219932
C	-1.6714422591	-3.0694589553	-0.0883214509
C	4.5455403344	-2.5662573397	-2.2214130437
C	3.8979222783	-3.1636853163	0.0470611703
C	-2.1235126548	-2.9933386104	2.6743584589
C	-2.9630328021	-3.2293186808	0.4194091672
H	-1.4803825759	-3.0747432313	-1.1604263685
C	5.8745068411	-2.8588027001	-1.9143311631
C	5.2350053724	-3.4406613420	0.3437394910
C	-3.1938448723	-3.1938686340	1.7972808810
H	-2.2949250486	-2.9645159846	3.7532054379
H	-3.7974901418	-3.3653547442	-0.2714955671
C	6.2241104666	-3.2935507445	-0.6319002598
H	6.6438575729	-2.7331152344	-2.6797005018
H	5.5010273243	-3.7777736256	1.3483557738
H	-4.2079760003	-3.3139690859	2.1831649455
H	7.2669351203	-3.5131637066	-0.3927180586
H	3.1160826633	-3.2833723777	0.7972473035
C	4.4303987119	0.5859555001	0.3702402362
F	5.2343828224	-0.1770665902	-0.3540271548
F	4.3141632876	0.0604137750	1.5899544931
F	4.9709070296	1.8005741583	0.4790862982
H	0.0173016183	-2.6345964679	2.8419661387
H	4.2454948839	-2.1979522225	-3.2028353204
C	-3.4874496674	3.5940657262	0.1918251679
H	-4.0718133269	3.9734189009	-0.6617596464
H	-4.1458603065	2.9345339136	0.7716790759
H	-3.2199380468	4.4579077850	0.8206708818
H	-1.6794823456	2.7389461837	2.8120024705
C	0.9271848969	2.3411408553	2.6023341193
O	0.7654600001	3.5329126162	2.6967943191
O	2.0479850379	1.6832945042	2.8885986268
C	3.1898477726	2.4973232285	3.1773887732
H	3.9917390734	1.8051254723	3.4523274543
H	3.4662486631	3.0645901092	2.2780391111
H	2.9669289727	3.1936900349	3.9980317137

TS3A

0 1

C	1.1812714914	3.2912273040	-1.0762786488
C	-3.9651484815	-1.2277331009	-0.8248508941
C	-4.8880442286	-1.7162154152	0.1044047662
C	-4.4041905820	-2.2307953865	1.3119179377

C	-3.0281715841	-2.2553373938	1.5883522987
C	-2.0929810581	-1.7516584359	0.6771383434
C	-2.5862969200	-1.2473575448	-0.5196042779
C	-4.1018882397	-0.6536466206	-2.1623117189
C	-2.8426971452	-0.3559275388	-2.5896306394
N	-1.8968981107	-0.5980611154	-1.5699188560
H	-2.5141563588	0.0653513519	-3.5365863966
H	-5.9590895608	-1.7022278046	-0.1094327120
H	-5.1064258981	-2.6267975165	2.0493385701
H	-2.6685134513	-2.6714912694	2.5312201281
H	-1.0330571242	-1.7587004454	0.9242175984
H	-0.9202141880	-0.9006686125	-1.8417770113
C	-5.3909660435	-0.4416435780	-2.8797209146
H	-5.9570188258	-1.3833506956	-2.9706072937
H	-5.2339230669	-0.0343314704	-3.8886993234
H	-6.0275183630	0.2643642388	-2.3185527965
O	2.1014176856	2.3228420079	-1.0279122111
C	-0.1049769948	3.1802166635	-1.4766097565
C	-1.3885240282	1.2560309119	-0.7997874188
C	-0.7391833174	1.9106252652	-1.9809069018
H	-0.7181481065	4.0790506311	-1.4049456989
H	0.0209476379	1.2323300939	-2.3955870035
H	-1.4686619274	2.1465013304	-2.7694105734
C	1.6706104378	4.6594454320	-0.6795203062
O	0.9930607104	5.6615392287	-0.7069278248
O	2.9508520195	4.6397530850	-0.2889451031
C	3.4955785186	5.8941813934	0.1090315233
H	2.9387408894	6.3082202218	0.9635003393
H	4.5374990675	5.6996762955	0.3908390961
H	3.4501211038	6.6209758949	-0.7165406188
P	1.6535784573	-1.3100856050	-1.2151344102
O	0.5896248859	-1.2344440093	-2.2743674773
O	3.0925975278	-1.1361592812	-1.9134280776
O	1.7856903028	-2.7858259991	-0.5445184339
N	1.5188181896	-0.1519640111	-0.0767720975
S	1.2832514257	-0.1799455813	1.4972511041
O	0.4575314290	0.9759983668	1.8764456767
O	0.9340357964	-1.4987286316	2.0431128168
H	1.7549208828	1.4407857019	-0.7234194500
C	-2.6239297394	1.6806346243	-0.2012754468
C	-3.6788676651	2.2673768175	-0.9410067904
C	-2.8461181651	1.3762705518	1.1610114656
C	-4.9069457691	2.5082270917	-0.3517953646
H	-3.5299547030	2.5242693956	-1.9901998618
C	-4.0751950740	1.6103325829	1.7599808325
H	-2.0214499188	0.9623653875	1.7434098645
C	-5.1238515735	2.1598270198	0.9980191940
H	-5.7318644916	2.9565384117	-0.9074603281
H	-4.2141943177	1.3579229303	2.8097614901
O	-6.3581053553	2.3977683950	1.4747833865
C	-6.6663220883	2.0511674818	2.8114238557
H	-6.5290384892	0.9705119385	2.9880244168
H	-6.0466936573	2.6152467222	3.5292353630
H	-7.7211048439	2.3123973989	2.9636576781
H	-0.6719597431	0.8405195532	-0.0934850308

C	2.9638649803	0.1953487509	2.2245491065
F	3.4388558719	1.3342882005	1.7335434194
F	2.8470923585	0.3047008244	3.5455285735
F	3.8073034720	-0.7945428471	1.9445355739
C	4.2786541457	-0.9449882200	-1.2075407633
C	4.7430167848	0.3539750850	-1.0054381094
C	4.9816215944	-2.0567188156	-0.7433110142
C	5.9458965865	0.5361019712	-0.3187751713
H	4.1530867502	1.2019207918	-1.3547760676
C	6.1842618308	-1.8585587294	-0.0620002127
H	4.5745029616	-3.0541169846	-0.9131402395
C	6.6680408266	-0.5641190535	0.1516026053
H	6.3118431715	1.5502211299	-0.1433174153
H	6.7426664999	-2.7215743077	0.3081519262
H	7.6065231704	-0.4136184648	0.6900429530
C	0.7319897773	-3.6626870552	-0.3053847433
C	0.6377498405	-4.2225030281	0.9692776783
C	-0.1785633648	-3.9955258146	-1.3121622664
C	-0.3993280702	-5.1151829231	1.2437760200
H	1.3562952164	-3.9201153162	1.7287689004
C	-1.2178781443	-4.8823625357	-1.0177282802
H	-0.0757017461	-3.5483037710	-2.2997185218
C	-1.3354410279	-5.4410993681	0.2567999531
H	-0.4815663671	-5.5496864287	2.2429016682
H	-1.9410428642	-5.1350444029	-1.7966820397
H	-2.1531075153	-6.1295303457	0.4810263903

TS4Aa

0 1

C	2.9110621701	-2.2698471457	2.0560965855
C	3.4150300457	-1.3924145698	1.0262875030
C	4.7858422064	-1.4086462463	0.6811135995
C	5.6135335548	-2.2678158763	1.3826758630
C	5.1338863309	-3.1229170536	2.4148774883
C	3.7965342993	-3.1312231668	2.7519323032
C	1.5123709555	-2.0911695685	2.1254630033
C	1.1773218807	-1.1604826998	1.1001924535
H	5.1790536414	-0.7719540575	-0.1075087853
H	6.6774853692	-2.2910177791	1.1340747015
H	5.8363803588	-3.7778403873	2.9340254720
H	3.4135643283	-3.7920509395	3.5320395101
N	2.3671748137	-0.6778800551	0.5284917250
C	2.2549484514	0.1862887760	-0.6538848710
H	1.4593803531	0.9009451022	-0.3935696163
C	0.5043742690	-2.7566135093	2.9836806564
H	-0.0493064514	-3.4930692768	2.3728063278
H	-0.2348522042	-2.0225812089	3.3384588348
H	0.9622954278	-3.2801514754	3.8338685856
C	1.7452713837	-0.6425055030	-1.8371690586
H	2.5133951365	-1.3572733010	-2.1567192336
H	1.5510550112	0.0460602326	-2.6728065492
C	0.3529088296	-2.3076865822	-0.3243344058
C	0.4301483126	-1.3518461615	-1.5154746186

H	0.0997314987	-1.9513747399	-2.3812408777
H	-0.3632973347	-0.6080829206	-1.3718818102
C	3.5111260152	0.9936485842	-0.8922554371
C	4.2231421325	0.9633661063	-2.0989702922
C	3.9825680569	1.8254311097	0.1298813711
C	5.3708349123	1.7323409539	-2.2744306078
H	3.8872302689	0.3306417587	-2.9209921348
C	5.1326009658	2.5993640368	-0.0253136551
H	3.4414715276	1.8630277253	1.0780061113
C	5.8390739936	2.5568879588	-1.2391877148
H	5.9278788666	1.7131341326	-3.2126938032
H	5.4670935810	3.2294624103	0.7980829605
O	6.9623009437	3.2661212241	-1.4966918644
C	7.4770609979	4.1221226845	-0.5009922420
H	6.7550759252	4.9121105652	-0.2274526398
H	8.3749223147	4.5904882035	-0.9245867233
H	7.7583716086	3.5663479059	0.4113629981
O	-0.8383744547	-2.6214225388	0.1055457083
C	1.2631732520	-3.5345727436	-0.2823332349
O	0.9718986929	-4.5674199628	0.2595560754
O	2.4273180134	-3.3185313742	-0.9126700109
C	3.3805542077	-4.3855041766	-0.8589701829
H	2.9709203519	-5.2892935075	-1.3318523151
H	3.6389677825	-4.6096264365	0.1851113675
H	4.2628473819	-4.0275314617	-1.4010366013
N	-2.4534692285	-0.6251322830	-0.1021793800
P	-2.4111389096	0.4954851643	1.0941187208
S	-3.4073661862	-0.6106272971	-1.4015983888
O	-1.3224665499	0.2065398665	2.0736724516
O	-3.9027409909	0.5876667486	1.6998891004
O	-2.2950391061	1.9916648726	0.4410139092
O	-2.7163874048	-1.2887389014	-2.5003633916
O	-4.0692420872	0.6784931172	-1.6449003563
C	-4.7880654325	-1.7778624545	-0.9153874526
H	0.3173069569	-0.4869987363	1.2007679550
H	-1.5184079158	-1.8360990388	0.0003657026
C	-1.1593294377	2.4111142162	-0.2185453500
C	-1.0698388970	2.2535022229	-1.6048107756
C	-0.1301541319	3.0202430637	0.5067726056
C	0.0682885781	2.7233160881	-2.2688152117
H	-1.8982234274	1.7859996757	-2.1376758331
C	0.9933221730	3.4976845381	-0.1723053596
H	-0.2337533914	3.1117243259	1.5886977211
C	1.0989609140	3.3474369901	-1.5593247213
H	0.1414656119	2.6065977610	-3.3527732617
H	1.7981764962	3.9810087139	0.3851405393
H	1.9880769697	3.7068825680	-2.0808037953
C	-4.5903821199	1.7497304806	2.0226311954
C	-5.3995010937	2.3406137480	1.0522190889
C	-4.4847165359	2.2733493073	3.3100109819
C	-6.1276092633	3.4823413191	1.3920093588
H	-5.4274871801	1.9020328029	0.0537197951
C	-5.2187146070	3.4175669672	3.6357612937
H	-3.8351344839	1.7790131794	4.0345386059
C	-6.0407564051	4.0222070817	2.6802601644

H	-6.7649855528	3.9561014101	0.6415987282
H	-5.1467667414	3.8366883314	4.6423208062
H	-6.6134205549	4.9158644805	2.9387764671
F	-4.2787762270	-2.9339928553	-0.4953311510
F	-5.5226020718	-1.2477409333	0.0576957619
F	-5.5626367424	-2.0017885858	-1.9733564429

TS4Ab

0 1

C	4.5884342808	1.7856425083	0.1088178572
C	4.6778434144	0.7780551918	1.1338120730
C	5.8753794294	0.5702391520	1.8474385783
C	6.9502752209	1.3901912273	1.5455904542
C	6.8738471350	2.4052469150	0.5508865890
C	5.7096682815	2.6073359303	-0.1644725388
C	3.2923574040	1.7018399991	-0.4552053970
C	2.6423697790	0.6164245861	0.1955415332
H	5.9547142039	-0.2035914980	2.6118908372
H	7.8881544473	1.2570009087	2.0902732686
H	7.7512543674	3.0253464995	0.3570618961
H	5.6476753011	3.3797913129	-0.9337833493
N	3.4787817940	0.1414129286	1.2143753275
C	3.1444376958	-1.0985046353	1.9207195573
H	3.8682057555	-1.1925803142	2.7461451056
C	2.6808241425	2.5056541680	-1.5421170816
H	1.6162449664	2.6753827076	-1.3263207952
H	3.2024256024	3.4623341318	-1.6862046813
H	2.7334115072	1.9390780380	-2.4896113752
C	3.3754238360	-2.2571881317	0.9374171997
H	4.4447156011	-2.2913692983	0.6944097727
H	3.1259838696	-3.2093749492	1.4274008647
C	2.6261443002	-0.9101359975	-1.2223388510
C	2.5376236245	-2.1460139662	-0.3380143084
H	2.8095996804	-2.9762066756	-1.0159105822
H	1.4772307601	-2.3017941778	-0.1162653479
C	1.7340883633	-0.9972780047	2.4900014759
C	1.3101949610	0.2055721270	3.0845585130
C	0.8113382285	-2.0436516435	2.4122669941
C	0.0122230520	0.3643196041	3.5468719503
H	2.0035297399	1.0472528031	3.1526768644
C	-0.4994754049	-1.9008649313	2.8722872990
H	1.0777153767	-2.9959707215	1.9553193009
C	-0.9167612997	-0.6844513609	3.4266636388
H	-0.3253868193	1.3083496169	3.9771196512
H	-1.1966674737	-2.7241900274	2.7350404533
O	-2.1707235132	-0.4302974059	3.8506491602
C	-3.1675692959	-1.4145444695	3.6394354620
H	-2.9876585712	-2.3050266631	4.2686446560
H	-4.1211062321	-0.9544769958	3.9278176236
H	-3.2109335884	-1.7186145384	2.5830145181
O	1.5977626863	-0.6432015274	-1.9730602230
C	3.9335322091	-0.5996563486	-1.9357239051
O	4.0092406125	-0.0547137073	-3.0055303351

O	4.9895285433	-1.0183115543	-1.2213733912
C	6.2789719856	-0.7154084327	-1.7631784865
H	6.4292019578	-1.2471427905	-2.7137845787
H	6.3740908893	0.3650872513	-1.9350951951
H	7.0056748341	-1.0469732475	-1.0130905756
N	-0.6511271982	-0.9364251209	-0.6609326876
P	-1.2470798752	0.5458415478	-0.3023429229
S	-1.3928639027	-2.3580391839	-0.5749607764
O	-0.1718706851	1.5618793834	-0.1087707476
O	-2.2686192729	0.8801158584	-1.5318612594
O	-2.2257719491	0.3997170686	0.9837566274
O	-0.3754137876	-3.4140293699	-0.5344598854
O	-2.5285754896	-2.4293244724	0.3519591072
C	-2.1807746474	-2.5421055176	-2.2679431533
H	1.5580059041	0.5841831636	0.3228520431
H	0.7010504299	-0.8526433776	-1.4951369000
C	-2.9693576834	2.0620943244	-1.7161584316
C	-2.4064322076	3.3141548932	-1.4544230097
C	-4.2755065133	1.9438163203	-2.1948598931
C	-3.1816698385	4.4583468801	-1.6629068686
H	-1.3865550568	3.3757470824	-1.0759822548
C	-5.0351919367	3.0952291166	-2.4005024249
H	-4.6762212433	0.9469693737	-2.3798734810
C	-4.4938279914	4.3567199886	-2.1317309354
H	-2.7496956243	5.4402014692	-1.4539795945
H	-6.0608193291	3.0041668424	-2.7657928853
H	-5.0930804000	5.2563293057	-2.2885813452
C	-3.5346809124	0.8477491315	1.0672742559
C	-3.7793257191	2.1228457383	1.5754725529
C	-4.5746137128	0.0148027280	0.6543839282
C	-5.0958719361	2.5798315938	1.6571712552
H	-2.9351650890	2.7410058837	1.8833927318
C	-5.8875270067	0.4825667466	0.7455135758
H	-4.3328046655	-0.9709008917	0.2586876795
C	-6.1518945611	1.7629996981	1.2410084081
H	-5.2959185622	3.5824513737	2.0418200200
H	-6.7096590159	-0.1588291288	0.4187764944
H	-7.1803868755	2.1258566934	1.3015389629
F	-2.5604139186	-3.8048617230	-2.4410318412
F	-1.3104719394	-2.2147283537	-3.2202951614
F	-3.2507884292	-1.7530536592	-2.3666056603

ECB

0 1

C	-2.0258895356	-1.5799525324	-1.4791308888
O	-0.9580557516	-2.1213641463	-1.2213389610
C	-2.0887738302	-0.0822245781	-1.6252291800
C	-2.0717381030	0.6857877857	-0.2551718619
C	-3.2660430102	0.7305694305	-1.1250660745
H	-1.4208089316	1.5597249622	-0.2856131275
H	-4.1544919411	0.1829803879	-0.8128877300
H	-3.4552597178	1.6637983910	-1.6548175271
C	-1.9919018462	-0.1166582209	0.9969272193
C	-0.7574029555	-0.2454556553	1.6441722961

C	-3.0873322929	-0.8165595733	1.5268154856
C	-0.6009714296	-1.0542938436	2.7688878206
H	0.1058879833	0.2862811778	1.2465306230
C	-2.9469779949	-1.6357747949	2.6432517888
H	-4.0706245039	-0.7261708147	1.0632845334
C	-1.6971272874	-1.7740222128	3.2668435355
H	0.3826383165	-1.1373149804	3.2276767157
H	-3.7953791656	-2.1878456635	3.0511112337
O	-1.6404368338	-2.6132350212	4.3309244980
C	-0.3883973255	-2.8620592278	4.9279914741
H	0.3343277209	-3.2706492631	4.1993737842
H	-0.5587967915	-3.6030958851	5.7200781332
H	0.0419103570	-1.9494482015	5.3786703741
C	-3.2548683746	-2.4062131640	-1.5735548095
C	-4.3639090842	-2.0147842246	-2.3407430181
C	-3.2734498553	-3.6398627634	-0.8997078113
C	-5.4791574585	-2.8485790763	-2.4310531669
H	-4.3441623742	-1.0679132960	-2.8821208625
C	-4.3969904679	-4.4587481793	-0.9736211399
H	-2.3958038696	-3.9260049644	-0.3181219089
C	-5.5003814597	-4.0642155640	-1.7402369788
H	-6.3352900528	-2.5492378771	-3.0396677230
H	-4.4156576443	-5.4098267942	-0.4367996391
H	-6.3802658325	-4.7092805635	-1.8013374333
C	-1.2600324969	0.5504852859	-2.6965520468
O	-1.2957345104	1.7337331997	-2.9583708535
O	-0.4853436939	-0.3312130384	-3.3362751720
C	0.3422393657	0.1572058475	-4.4036853055
H	0.6361911337	-0.7293253870	-4.9779533940
H	1.2392826928	0.6388191986	-3.9907357394
H	-0.2200439308	0.8624351954	-5.0306098828
N	1.5792763324	-1.3413775901	-1.0040960211
S	2.3552119961	-2.7948438040	-0.8389166567
O	2.9780435653	0.5010807708	-2.4663744285
O	3.7925843252	-2.5725825894	-0.9215955354
O	1.6608977817	-3.8152653490	-1.5998876319
P	2.3821449576	0.1279798121	-1.1666194257
O	1.1707803599	1.1025863590	-0.6646852611
O	3.3843864476	0.1320040004	0.1013294828
C	1.4129943137	2.4699362727	-0.5291839110
C	4.7485765244	0.4099360455	0.0737125422
C	2.0656802273	2.9319008768	0.6161911618
C	0.9735867985	3.3442245872	-1.5225382866
C	5.2414127696	1.5628918669	-0.5372335316
C	5.5889303435	-0.4871370260	0.7307638980
C	2.2812119659	4.3038936341	0.7685457576
C	1.1913698152	4.7154067409	-1.3549057678
H	0.4438566797	2.9497462621	-2.3890728818
C	6.6139684841	1.8185722525	-0.4787919219
C	6.9568556194	-0.2145100217	0.7868002867
C	1.8444310978	5.1989879084	-0.2147995614
H	2.7915226805	4.6734692566	1.6606307514
H	0.8464846735	5.4094890336	-2.1245886953
C	7.4727453234	0.9370341208	0.1835960752
H	7.0115444530	2.7160293676	-0.9582870807

H	7.6237459525	-0.9124687344	1.2981445503
H	2.0137914338	6.2709746518	-0.0930673202
H	8.5443272177	1.1441289473	0.2251518053
H	5.1583865964	-1.3857815217	1.1708419920
H	0.5421230223	-1.4502723597	-1.1750386968
C	-2.4871737611	3.8655358048	0.7155264901
C	-1.0026587525	3.3653925992	2.3294120327
C	-2.1857743263	2.7967693296	2.7438085776
C	-3.1561056350	3.1028817589	1.7201849800
H	-0.0265425637	3.3560206127	2.8087395317
N	-1.1788882423	4.0068269175	1.1158138707
C	-4.5150413469	2.7859917062	1.5438678076
H	-5.0439671095	2.1935945480	2.2945276956
C	-3.1435497145	4.3126091285	-0.4382349127
H	-2.6133733722	4.8799790221	-1.2059525387
C	-5.1715133720	3.2349370901	0.4014439810
C	-4.4920248050	3.9928015098	-0.5781413588
H	-6.2283193240	2.9987861858	0.2549413544
H	-5.0320595657	4.3278426332	-1.4670192886
H	-0.4529933773	4.4885699756	0.6010880466
C	1.9954672774	-3.1963145646	0.9586824759
F	2.6589579606	-4.2986552228	1.2881786645
F	2.3952795627	-2.1970980049	1.7464912408
F	0.6952214730	-3.3954490565	1.1329264872
H	4.5638117503	2.2370397899	-1.0605961197
H	2.3960091948	2.2169834354	1.3697016095
C	-2.4260121250	1.9957827430	3.9826386465
H	-3.2067081602	2.4516573549	4.6154729870
H	-2.7590738860	0.9748663577	3.7371438943
H	-1.5106517542	1.9100582194	4.5865548774

IN1Ba

0 1

C	-0.5056572759	2.0498293372	-1.1893383723
C	-2.7366954355	-3.2541128884	0.3469047747
C	-3.1316748093	-4.0611411985	1.4647741291
C	-2.1638842746	-4.4788462846	2.3343063051
C	-0.7789078838	-4.1293154370	2.1320795232
C	-0.3424120580	-3.3811651440	1.0691559792
C	-1.3333386741	-2.9127232664	0.1638927574
C	-3.4423716034	-2.6471389507	-0.6666163363
C	-2.4829643640	-1.8353956441	-1.4854267699
N	-1.1896465073	-2.1270590744	-0.8933004666
H	-2.4866912796	-2.1675007419	-2.5404713294
H	-4.1819889610	-4.3161968863	1.6155666656
H	-2.4290958823	-5.0814981487	3.2050167128
H	-0.0428634479	-4.4655466357	2.8660620302
H	0.7031004189	-3.1124612586	0.9454759219
H	-0.2385944781	-1.8491937097	-1.2243388265
O	0.1994193945	0.9259817856	-1.0165308290
C	-1.6599157680	1.8930240265	-1.9136105901
C	-2.8900562528	-0.3192703716	-1.4837462797
C	-1.9631944447	0.5106302629	-2.4353150985

H	-1.0196538215	-0.0194143893	-2.6051439055
H	-2.4833544609	0.5976736793	-3.3994347016
C	-3.0760309217	0.2342503490	-0.0835860942
C	-4.2973289863	0.8346337226	0.2428681997
C	-2.0766551367	0.2151696881	0.9084803249
C	-4.5368075611	1.3905010956	1.5047716412
H	-5.0736148626	0.9120729254	-0.5224075565
C	-2.2964252019	0.7567932222	2.1675827647
H	-1.0902955800	-0.1868770342	0.6971825148
C	-3.5314722251	1.3480107807	2.4804026562
H	-5.5015544454	1.8554085338	1.7059267518
H	-1.4984180153	0.7418210187	2.9102417298
O	-3.6571111238	1.8541307736	3.7327044943
C	-4.8539983353	2.5020306210	4.0881282906
H	-4.7323195188	2.8429074500	5.1249373747
H	-5.0543340830	3.3778773479	3.4442756943
H	-5.7235418224	1.8211958289	4.0354345429
O	1.4713127959	-2.0490483201	-1.2835062556
N	2.0700881657	-0.1132238630	0.4951768154
S	1.9286825936	-0.4312530409	2.0447147059
O	0.8250587886	0.3500517625	2.6197783288
O	2.0525216822	-1.8529158374	2.3949915733
H	0.9526834608	0.9634701431	-0.3749695767
H	-3.8866499637	-0.3098959169	-1.9455966365
P	2.5007987926	-1.1306267446	-0.6917431865
O	3.8086463093	-1.9185152585	-0.1299129173
O	3.0737726764	-0.1594325495	-1.8628645896
C	4.6072441098	-2.8164504931	-0.8083313950
C	3.6369771560	1.0832006454	-1.6027593957
C	4.1835368753	-3.5132352617	-1.9440658205
C	5.8900816455	-3.0029844090	-0.2854105840
C	4.7287129454	1.2193804633	-0.7434148874
C	3.0543907706	2.1949386524	-2.2110852274
C	5.0726280696	-4.3984496853	-2.5607206658
C	6.7626712710	-3.8946184769	-0.9098391498
H	6.1751439993	-2.4460487880	0.6087896356
C	5.2307117757	2.4959363050	-0.4826015960
C	3.5710089081	3.4651583286	-1.9492785003
C	6.3594173320	-4.5936068141	-2.0524481548
H	4.7479177567	-4.9435269825	-3.4504884273
H	7.7654445463	-4.0412628771	-0.5013334081
C	4.6541752893	3.6202991473	-1.0802278135
H	6.0727010329	2.6092943194	0.2041299434
H	3.1052251467	4.3391243578	-2.4072627021
H	7.0449025246	-5.2883936639	-2.5425691263
H	5.0429141581	4.6175387500	-0.8633418159
H	2.1917845976	2.0493356514	-2.8618433960
C	-2.7829765484	2.8271811735	-2.0158528239
O	-3.8471158807	2.5223569275	-2.5299685344
O	-2.5928818569	4.0362203091	-1.4499462944
C	-3.7177664865	4.9028521921	-1.4221776222
H	-4.0995212762	5.0937045539	-2.4366361194
H	-4.5323267136	4.4656211255	-0.8236806093
H	-3.3701707254	5.8363286421	-0.9616485537
C	0.0955873337	3.2613184360	-0.5844948551

C	0.1942075849	4.4682101120	-1.2947937881
C	0.7056266280	3.1583926094	0.6782642117
C	0.8816232600	5.5513400888	-0.7503903629
H	-0.2567586681	4.5446280997	-2.2834370705
C	1.3948380679	4.2445143456	1.2180857792
H	0.6488315822	2.2323245960	1.2531100824
C	1.4853908906	5.4428950477	0.5070738120
H	0.9605318294	6.4822717981	-1.3173986207
H	1.8711353696	4.1406041870	2.1950988503
H	2.0335765102	6.2901453945	0.9264280428
H	5.1586548766	0.3383622585	-0.2680615194
H	3.1717805469	-3.3675519767	-2.3224892585
C	3.4581180500	0.3798470218	2.7543551314
F	3.4696335635	1.6792137534	2.4552984773
F	3.4668667149	0.2413776058	4.0774164307
F	4.5608237452	-0.1828230736	2.2555053259
C	-4.9078251738	-2.6430357469	-0.8899618387
H	-5.3278437418	-1.6987476514	-0.4989156369
H	-5.1570213369	-2.6888379315	-1.9610877144
H	-5.4024616682	-3.4742686566	-0.3696035487

IN1Bb

0 1

C	-0.6364302814	1.7422848146	-1.7523350520
C	2.9542209946	0.5026014289	2.5166470368
C	3.9111630397	0.7259325592	3.5573845854
C	4.0597083492	-0.2309576913	4.5245975222
C	3.2687705512	-1.4351879676	4.5080907975
C	2.3313784942	-1.6954838901	3.5412505777
C	2.1592627928	-0.7130680889	2.5239134369
C	2.5674785483	1.2460767971	1.4148489664
C	1.5214520368	0.4704554765	0.6835368336
N	1.3285289171	-0.7104114426	1.5015467565
H	0.5762573940	1.0527457542	0.6833363387
H	4.5102783117	1.6382230105	3.5674096402
H	4.7856421246	-0.0937490505	5.3282044210
H	3.4275292397	-2.1667349305	5.3044513428
H	1.7305127442	-2.6054008472	3.5333285796
H	0.7024355536	-1.5069675894	1.2184330447
O	-0.8667861431	0.4364157670	-1.9629492513
C	0.6705312402	2.1540801006	-1.7731915748
C	1.8018391869	0.0094604686	-0.7893054415
C	1.7949243469	1.1389144578	-1.8364704971
H	0.9436050561	-0.6330296900	-1.0090283609
H	2.7406030623	1.6920817564	-1.8159393067
H	1.7738502658	0.6224842731	-2.8122570471
C	3.0420605792	-0.8594641425	-0.8525933119
C	2.9133638732	-2.2596672661	-0.8457285817
C	4.3326572823	-0.3211769113	-0.8695746727
C	4.0311263521	-3.0849947098	-0.8648253738
H	1.9190734175	-2.7079881614	-0.8143258658
C	5.4691265260	-1.1369032626	-0.8880261795
H	4.4738137300	0.7618720726	-0.8733696418

C	5.3213185296	-2.5315513614	-0.8874009645
H	3.9284385329	-4.1713012252	-0.8680271419
H	6.4557187311	-0.6748503524	-0.9058050726
O	6.3546016139	-3.4124401788	-0.9076194442
C	7.6713958797	-2.9166267851	-0.9365299490
H	7.9019168743	-2.3074368106	-0.0434018065
H	8.3369090959	-3.7900612926	-0.9523126565
H	7.8616231177	-2.3053457274	-1.8374845316
H	-1.5474675495	0.0675689799	-1.3250361083
N	-2.0779542960	-0.7901019804	0.0816560816
S	-2.2073724608	0.0274307615	1.4564908319
O	-0.2131063631	-2.6809424549	0.6245154320
O	-2.1867731977	-0.8203302433	2.6539494910
O	-1.3556798119	1.2299068540	1.4304786483
P	-1.5569216110	-2.3373461988	0.0506726567
O	-1.6199052852	-2.6553164261	-1.5498597517
O	-2.6666464237	-3.3298101413	0.6865061495
C	-0.5365355952	-3.1797998684	-2.2438655043
C	-4.0283367497	-3.0795044753	0.7380669851
C	-0.0899440598	-4.4762327875	-1.9812383650
C	0.0848766752	-2.3798536459	-3.2036312226
C	-4.6649203736	-3.2735233707	1.9635961728
C	-4.7388926681	-2.6682292927	-0.3916675260
C	1.0048907348	-4.9736694188	-2.6921699338
C	1.1751031748	-2.8921510349	-3.9114615243
H	-0.2792039959	-1.3643488067	-3.3571088456
C	-6.0409839625	-3.0620439190	2.0563666733
C	-6.1138337629	-2.4470917058	-0.2810676570
C	1.6402159332	-4.1851213055	-3.6565293821
H	1.3649591013	-5.9844348694	-2.4862438966
H	1.6731964140	-2.2676391952	-4.6566285450
C	-6.7686866384	-2.6459335124	0.9372424807
H	-6.5447786640	-3.2088224859	3.0146159211
H	-6.6751770194	-2.1175430147	-1.1585318602
H	2.5016754141	-4.5755833494	-4.2022829022
H	-7.8439484855	-2.4711443656	1.0163070453
C	1.0965973109	3.5626411685	-1.8614128884
O	2.2382638522	3.9458877011	-1.6590384958
O	0.1301160902	4.4173824296	-2.2421731567
C	0.4765558289	5.7951008402	-2.2827720005
H	0.8247378262	6.1441499271	-1.2981728343
H	1.2770321327	5.9791744029	-3.0154174427
H	-0.4373514689	6.3283676631	-2.5721225518
C	-1.8524376561	2.5632854631	-1.5426858105
C	-2.9463788458	2.3713962608	-2.4003467523
C	-1.9664578136	3.4730151224	-0.4810020986
C	-4.1202409438	3.1050183898	-2.2241010138
H	-2.8656782834	1.6468875570	-3.2128943768
C	-3.1451172753	4.1901257711	-0.2942798418
H	-1.1376328222	3.5852197826	0.2168275832
C	-4.2223358008	4.0150828596	-1.1701498427
H	-4.9615101852	2.9563064983	-2.9050895495
H	-3.2321735201	4.8784131651	0.5494771771
H	-5.1466956193	4.5781397597	-1.0196898663
H	-4.2171344148	-2.5226542739	-1.3382208968

H	-4.0650406381	-3.5665792018	2.8256777600
H	-0.5908303175	-5.0735101168	-1.2181126987
C	-3.9542701537	0.7241411724	1.3411706122
F	-4.8239177674	-0.1412270996	1.8485619615
F	-4.0094251676	1.8541280503	2.0423378971
F	-4.2825593433	0.9785602972	0.0793129623
C	3.0006591424	2.6190133993	1.0708669340
H	3.8847768489	2.9230721158	1.6471647371
H	3.1840119080	2.7638329108	-0.0026130955
H	2.1818236721	3.3217244461	1.3076041098

IN2Ba

0 1

C	1.4261474123	1.7560195395	2.9902654652
C	1.5165609917	1.5969814809	4.3846938397
C	0.3630066168	1.7286317810	5.1527746657
C	-0.8816527666	2.0227070860	4.5558689249
C	-1.0020237623	2.1933447325	3.1795914689
C	0.1582120046	2.0477027399	2.4106930969
C	2.3753849003	1.6674697033	1.9055214557
C	1.6629342507	1.8921274762	0.7361181958
N	0.3357430282	2.1242170421	1.0493235249
H	2.4746443945	1.3638833697	4.8565487379
H	0.4170004536	1.5961017169	6.2361310993
H	-1.7718140059	2.1060569677	5.1837195437
H	-1.9615876801	2.4024239131	2.7063878137
H	-0.4248548851	2.3110211470	0.4014036909
C	2.1110813756	1.7511148968	-0.6938636778
C	1.7446506895	0.3509795866	-1.2543041831
H	3.2064221524	1.8168240435	-0.6963567030
H	2.1520430443	0.2569442718	-2.2715951310
H	0.6536970142	0.2684032336	-1.3311975162
C	1.5867819017	2.8625700396	-1.5899578478
C	2.3999417629	3.9740604849	-1.8694557567
C	0.2932518909	2.8531626673	-2.1244544978
C	1.9430726882	5.0314591527	-2.6466197242
H	3.4137373007	4.0090000260	-1.4617915501
C	-0.1860110052	3.9119460360	-2.9043507278
H	-0.3851846905	2.0209524658	-1.9340015144
C	0.6397513255	5.0116673488	-3.1707949379
H	2.5762264213	5.8938035104	-2.8633146281
H	-1.2049293543	3.8640439974	-3.2869769890
O	0.2737628893	6.0869104576	-3.9143722375
C	-1.0311355877	6.1352086861	-4.4406921580
H	-1.2241366984	5.3009021487	-5.1396487490
H	-1.1151403389	7.0838584314	-4.9875400069
H	-1.7965935530	6.1109294045	-3.6441175995
N	-1.5273254385	-0.7510238540	0.9020878691
S	-1.9302081437	-1.0665921204	2.4819343078
O	-2.2953088987	1.7177547457	0.0099827366
O	-3.0411196040	-0.2077736403	2.8684919350
O	-0.7162007278	-1.2426488300	3.2537958211
C	-2.6497774557	-2.7924602182	2.3093341577

P	-2.3716984281	0.2505320930	-0.1555679968
O	-3.8546447503	-0.3763582542	-0.1066579512
O	-1.7353627691	-0.1825681672	-1.5784971029
C	-5.0189219461	0.3964537835	-0.1714693957
C	-1.6492446026	-1.4157513683	-2.2123881937
C	-5.6444363317	0.5645837119	-1.4043584167
C	-5.5352513454	0.9400916004	1.0022488710
C	-2.3461309760	-2.5485653032	-1.7894605247
C	-0.7995015876	-1.4578286951	-3.3201203636
C	-6.8316096708	1.2998014291	-1.4605056147
C	-6.7215472168	1.6741807187	0.9301816758
H	-5.0032552782	0.7784061757	1.9402319860
C	-2.1780867116	-3.7404462200	-2.5009348188
C	-0.6442955521	-2.6550709110	-4.0182327070
C	-7.3701635584	1.8549992239	-0.2958531309
H	-7.3352823785	1.4392325497	-2.4197795186
H	-7.1397105808	2.1089435196	1.8408401580
C	-1.3328195327	-3.8007929749	-3.6108827485
H	-2.7182849941	-4.6317087138	-2.1735991893
H	0.0310602037	-2.6945319586	-4.8752566930
H	-8.2971630438	2.4305931720	-0.3438733349
H	-1.2028758321	-4.7395340231	-4.1523305533
C	3.7457903871	-0.9593765399	-0.3594225686
O	4.5302972792	-0.3241478383	-1.0225059207
O	4.1082632725	-1.9155177257	0.5068213186
C	5.5082518560	-2.1804998362	0.6151969918
H	5.9220835441	-2.4719278720	-0.3613812162
H	6.0408575324	-1.2873068397	0.9734220736
H	5.6076318339	-3.0002513368	1.3360084607
H	-0.2710326817	-0.5505588300	-3.6157557052
H	-0.6550761495	-1.2453324895	0.5937416356
C	2.2374025447	-0.7990574482	-0.3638149888
H	1.9599070154	-0.5598076288	0.6756948833
C	1.4905936184	-2.0970596906	-0.6492565854
O	0.4540306188	-2.3253013786	-0.0347413356
C	1.9509393148	-3.0591835912	-1.6810017552
C	2.8737262527	-2.7114121338	-2.6826876986
C	1.3790619743	-4.3431413022	-1.6815267053
C	3.2096983279	-3.6357950518	-3.6725447833
H	3.3225282230	-1.7188758482	-2.7078320785
C	1.7343270420	-5.2696406183	-2.6567877240
H	0.6487467348	-4.5846850850	-0.9087739144
C	2.6457602058	-4.9151491865	-3.6581221015
H	3.9172089326	-3.3564855912	-4.4562233058
H	1.2929528815	-6.2686955467	-2.6470474535
H	2.9182342794	-5.6392278990	-4.4298616700
H	-5.2021971953	0.1201038101	-2.2978340297
H	-3.0101862422	-2.5092044640	-0.9281632102
F	-1.7620005418	-3.5879445017	1.7202313872
F	-3.7585640703	-2.7623411563	1.5743624488
F	-2.9345696200	-3.2600237806	3.5164008121
C	3.8327008439	1.3549083200	2.0530587201
H	3.9975829564	0.3126990782	2.3807754884
H	4.3842649023	1.4954637805	1.1126220049
H	4.3021038572	2.0018929960	2.8129919645

IN2Bb

0 1

C	0.2635926728	-4.1739987056	0.7121398982
C	-0.0281827905	-5.4404109411	0.1805575102
C	-1.3418279409	-5.9024669629	0.2312235415
C	-2.3700563917	-5.1199076568	0.7944800613
C	-2.1095563918	-3.8564714320	1.3250701305
C	-0.7880179132	-3.4073903450	1.2763279840
C	1.4673572261	-3.3705001235	0.7811044937
C	1.1215311747	-2.1755128140	1.3664313947
N	-0.2550172388	-2.1730338390	1.6368351608
H	0.7575879376	-6.0460865526	-0.2771380558
H	-1.5854776691	-6.8830964637	-0.1844651559
H	-3.3925987087	-5.5040286844	0.8044927183
H	-2.8975425786	-3.2247533040	1.7378265351
H	-0.6930071162	-1.5656811164	2.3249617981
C	1.9365805496	-0.9161822848	1.5325404678
C	2.0712995486	-0.2076597319	0.1649721094
H	2.9434922078	-1.2216561573	1.8577336611
H	1.0854672294	0.0672039279	-0.2305498916
H	2.4839661162	-0.9069466201	-0.5742797841
C	1.3616713664	-0.0122711709	2.6103007260
C	1.8738130919	-0.0197723602	3.9176872748
C	0.2574402728	0.8061735426	2.3517670179
C	1.2923363128	0.7451929073	4.9228515185
H	2.7449381366	-0.6379420903	4.1487276327
C	-0.3528348177	1.5705125787	3.3488224278
H	-0.1583067439	0.8596234263	1.3480328558
C	0.1645062968	1.5397975593	4.6509032862
H	1.6887119706	0.7413629393	5.9397993552
H	-1.2302118383	2.1605936661	3.0891084675
O	-0.3483726552	2.2357607412	5.6946367080
C	-1.5015957539	3.0185259679	5.4835995519
H	-1.3229506669	3.8209041058	4.7452175781
H	-1.7536250639	3.4731035576	6.4507749672
H	-2.3538458160	2.4058518198	5.1393020141
N	-1.6144962532	-0.7543605313	-0.4459619047
S	-1.6808877153	-1.7018018233	-1.8379828165
O	-3.0056703079	-0.6298140215	1.8270921301
O	-2.9626583126	-2.3578630375	-2.0144787176
O	-0.4089098658	-2.4066170302	-1.8786375434
C	-1.5814854472	-0.3861406963	-3.1727158311
P	-2.8497180159	-0.0513294187	0.4738553438
O	-4.0532504223	-0.1311839527	-0.5873351099
O	-2.4918549504	1.5261781664	0.5971349986
C	-5.2912077835	0.4858875375	-0.4736003011
C	-2.2574511780	2.3823872934	-0.4808805690
C	-5.8893020055	0.8945711280	-1.6654378662
C	-5.9048166981	0.6869176716	0.7636663052
C	-3.2979540424	3.1904650281	-0.9390942172
C	-0.9869188798	2.4410535824	-1.0489580735
C	-7.1318783861	1.5273041880	-1.6147112377

C	-7.1456113665	1.3311893484	0.7968274849
H	-5.4188833123	0.3389217859	1.6766711627
C	-3.0558231267	4.0620396757	-2.0035417447
C	-0.7569379493	3.3115135570	-2.1161559137
C	-7.7608846756	1.7524469240	-0.3850085186
H	-7.6088925366	1.8508068450	-2.5426646405
H	-7.6349872079	1.4968869085	1.7593001553
C	-1.7896498513	4.1216301728	-2.5954721925
H	-3.8655485561	4.6957298068	-2.3725362960
H	0.2357364597	3.3309176149	-2.5689168383
H	-8.7313915190	2.2517672148	-0.3492924127
H	-1.6096074941	4.8004889451	-3.4320071422
C	4.3868750844	0.6434664612	0.5322562190
O	4.9511656569	0.7949424715	1.5879897631
O	4.9477247932	0.0448573540	-0.5316786037
C	6.2931830150	-0.4077702473	-0.3724044935
H	6.3554461421	-1.1620097440	0.4260466236
H	6.5834959848	-0.8447104814	-1.3349696187
H	6.9546785457	0.4323676024	-0.1144766461
H	-0.1904502313	1.7950769674	-0.6881256137
H	-0.9049871851	-1.1799138631	0.1875485533
C	2.9536846888	1.0455049148	0.2280835850
H	2.6320993838	1.6773723891	1.0701253383
C	2.8459870575	1.8855354195	-1.0505758998
O	2.0661701229	1.5748668415	-1.9307529669
C	3.6879001745	3.1157056105	-1.1694565744
C	3.7596158564	3.7426733203	-2.4258285977
C	4.3791322128	3.6754852396	-0.0810909406
C	4.5131967504	4.9010530603	-2.5950167541
H	3.2160803517	3.2934838948	-3.2586814490
C	5.1277629032	4.8419721965	-0.2522742314
H	4.3394939485	3.2133620413	0.9056133938
C	5.1980125311	5.4541489242	-1.5062120085
H	4.5697608320	5.3777963312	-3.5763698242
H	5.6572748968	5.2741830546	0.5996460857
H	5.7874970758	6.3649180408	-1.6367575636
F	-0.4137863543	0.2306658529	-3.1119024796
F	-1.7047587474	-0.9953727227	-4.3454200818
F	-2.5656203393	0.4993782563	-3.0341193750
H	-4.2785977238	3.1263611866	-0.4672799183
H	-5.3640171934	0.7177524170	-2.6044858633
C	2.7981351242	-3.7497335158	0.2145557158
H	3.5793061319	-3.0201919520	0.4755634288
H	3.1249768087	-4.7367261532	0.5820297499
H	2.7559640261	-3.8119594048	-0.8867455151

IN3Ba

0 1

C	-2.3479131763	-1.2350189229	0.6742387551
C	0.8835378103	4.5313957280	0.4501774469
C	1.9699392229	5.1807930680	-0.1650834005
C	2.6105266521	4.5619608748	-1.2347107375
C	2.1797121327	3.3049872054	-1.7093419505

C	1.1003726987	2.6484434667	-1.1280322026
C	0.4692048089	3.2590245397	-0.0380727567
C	0.0184329131	4.8633563206	1.5561618896
C	-0.8500712288	3.8049230134	1.6933573790
N	-0.5836096317	2.8266066257	0.7448711052
H	-1.6485647958	3.6769334098	2.4201625466
H	2.3052411602	6.1568415061	0.1955999669
H	3.4633452801	5.0484895227	-1.7133123285
H	2.7054188069	2.8250421852	-2.5369386795
H	0.7665741372	1.6916181835	-1.5210449429
C	0.0761903014	6.1056547484	2.3872921810
H	1.0537682704	6.2121164383	2.8889470123
H	-0.7002491987	6.1046319091	3.1674228614
H	-0.0669716639	7.0120571359	1.7737591545
O	-1.2396337405	-1.6711984051	0.3910284808
C	-2.5061402637	-0.3135529376	1.8737446768
C	-1.2794806724	1.5619863566	0.5564183212
C	-1.4734286523	0.8431082927	1.9094539611
H	-0.4876565403	0.4928519462	2.2430944347
H	-1.8284707715	1.5469869738	2.6740051168
P	2.2620693687	-0.3563739035	1.0902137372
O	1.7964705614	0.8874240882	1.7343303320
O	2.2795362721	-1.5872435555	2.1276396968
O	3.7265202966	-0.3882844128	0.4371184220
N	1.2671118370	-0.9567202480	-0.1536792909
S	1.4260791760	-0.9742568993	-1.7903573445
O	0.0879258839	-0.8786158840	-2.3664618772
O	2.5254448742	-0.1286813019	-2.2251211905
C	-2.5563352557	1.6802456136	-0.2569352168
C	-3.7233249845	2.2623334603	0.2668880249
C	-2.6117202904	1.1277026233	-1.5389936657
C	-4.9127140787	2.2499392004	-0.4500627944
H	-3.7071517881	2.7192846581	1.2596207029
C	-3.7978018296	1.1158536955	-2.2790568641
H	-1.7221852437	0.6541136747	-1.9607432771
C	-4.9636486526	1.6553435887	-1.7247527347
H	-5.8287877687	2.6833423673	-0.0445588878
H	-3.8030781688	0.6499937472	-3.2631777914
O	-6.1788246501	1.6386681842	-2.3266893012
C	-6.2846727511	1.0964435466	-3.6242723779
H	-5.6587373609	1.6474607785	-4.3485498948
H	-5.9970595119	0.0309122240	-3.6448840795
H	-7.3384954713	1.1903894384	-3.9181355429
H	-0.5946852417	0.9449237944	-0.0330764935
H	0.3107189065	-1.2554201400	0.1457581469
H	-3.5052271612	0.1287315531	1.8830963728
C	1.9744561978	-2.7357283816	-2.1189214757
F	1.1629727241	-3.5843860394	-1.5005640962
F	1.9335123121	-2.9560570512	-3.4269964765
F	3.2172969259	-2.9051597313	-1.6820446069
C	2.2720368112	-2.9363766345	1.7766818642
C	1.0761761679	-3.6429326353	1.9057651595
C	3.4432855391	-3.5440344675	1.3275090185
C	1.0618096205	-4.9975607827	1.5658820389
H	0.1799628827	-3.1324815383	2.2630889403

C	3.4110121239	-4.8994445932	0.9898201565
H	4.3541094661	-2.9515201790	1.2351714109
C	2.2231558751	-5.6267451248	1.1059632379
H	0.1308254127	-5.5615657165	1.6578977200
H	4.3205919112	-5.3860604544	0.6302543326
H	2.2025267214	-6.6854761601	0.8378699228
C	4.5008042417	0.6858781731	-0.0182504771
C	5.3468176122	0.4111205281	-1.0910357241
C	4.4523163571	1.9435587763	0.5791705196
C	6.1763069186	1.4239245913	-1.5728236710
H	5.3223145290	-0.5809277928	-1.5419317670
C	5.2867806276	2.9474887390	0.0800590215
H	3.7543942702	2.1398993205	1.3917303677
C	6.1500966997	2.6946259227	-0.9877181094
H	6.8402775491	1.2197921986	-2.4160557784
H	5.2422367912	3.9413332464	0.5290739070
H	6.7961672872	3.4876373498	-1.3707824156
C	-3.5200166124	-1.6121842012	-0.1563281669
C	-4.8365922059	-1.2178944866	0.1331541692
C	-3.2649509858	-2.3579011422	-1.3236700261
C	-5.8795028637	-1.5633058046	-0.7271265782
H	-5.0598661056	-0.6272577578	1.0204607918
C	-4.3057733771	-2.7001890712	-2.1794513088
H	-2.2324116703	-2.6293238438	-1.5467683956
C	-5.6163939603	-2.3034445451	-1.8816460290
H	-6.8971519990	-1.2385917310	-0.5022119989
H	-4.0995173074	-3.2719922177	-3.0870162039
H	-6.4341115214	-2.5697928325	-2.5559829104
C	-2.3520385543	-1.1170012261	3.1599138276
O	-1.7125896117	-2.1302215669	3.2956327570
O	-3.0048613764	-0.5106832449	4.1623881086
C	-2.8652570433	-1.1040715940	5.4570946221
H	-1.8071412421	-1.1326250167	5.7560034429
H	-3.4418785971	-0.4735520773	6.1438910376
H	-3.2576753682	-2.1315582804	5.4555887392

IN3Bb

0 1

C	2.3215920779	-1.4518166567	-0.9750195872
C	-1.0694208068	4.3823568723	-0.9392633548
C	-2.2044823225	5.0077617653	-0.3922145688
C	-2.7906055449	4.4676236999	0.7491608636
C	-2.2558838986	3.3168321439	1.3642362277
C	-1.1220531561	2.6890510222	0.8560893564
C	-0.5447167715	3.2191021407	-0.3040299843
C	-0.2356250882	4.6493308518	-2.0854308733
C	0.7242700990	3.6622149461	-2.0984160449
N	0.5455491507	2.7906117228	-1.0335856867
H	1.5282894815	3.5153280777	-2.8145167709
H	-2.6224256780	5.9020701158	-0.8623250158
H	-3.6835390752	4.9322259828	1.1730492890
H	-2.7395267606	2.8978509157	2.2482496203
H	-0.6921785652	1.8272620542	1.3624505735

C	-0.4005424677	5.7689355250	-3.0638143786
H	-1.3832833575	5.7291142883	-3.5655306988
H	0.3733854931	5.7369351053	-3.8457782323
H	-0.3359322004	6.7540663713	-2.5697255535
O	1.3308195553	-1.3654762882	-0.2639546364
C	2.8346071678	-0.2617716371	-1.7716203770
C	1.3457678004	1.6252896522	-0.6685967088
C	1.8728152855	0.9333979134	-1.9391000058
H	0.9924708770	0.6133370137	-2.5143562328
H	2.4279830132	1.6545597933	-2.5553041734
P	-2.0213298425	-0.5282322316	-1.1740716573
O	-1.3932435258	0.5587168240	-1.9487484089
O	-1.9289002291	-1.9271555591	-1.9748264975
O	-3.5718210401	-0.4299285206	-0.7883487771
N	-1.2713024338	-0.8943032899	0.3098385436
S	-1.7226324834	-0.7109173092	1.8867534744
O	-0.5030205629	-0.4762881205	2.6510060301
O	-2.9125366578	0.1108720064	2.0147975092
C	2.3994208173	1.9675391151	0.3698674922
C	3.3742773162	2.9503055800	0.1329602613
C	2.3998469895	1.3170935123	1.6056960927
C	4.3269857185	3.2591075832	1.0956026806
H	3.3888073498	3.4833783330	-0.8198995530
C	3.3529726615	1.6142849271	2.5860204181
H	1.6410862427	0.5618853803	1.8216633512
C	4.3260666312	2.5892663242	2.3319981606
H	5.0882306708	4.0211134877	0.9191491333
H	3.3151415982	1.0874189458	3.5388674634
O	5.3048553670	2.9445717010	3.2044363506
C	5.3328209383	2.3400400884	4.4757346853
H	4.4054149520	2.5347180106	5.0436176209
H	5.4798530721	1.2463220844	4.4087774121
H	6.1816117554	2.7806880567	5.0155043655
H	0.6475410146	0.9304452259	-0.1993357688
H	-0.2561966882	-1.0848938260	0.2222278588
H	3.0108551214	-0.6293109269	-2.7953496546
C	-2.2595644079	-2.4463146680	2.3511522493
F	-1.3552070061	-3.3296203408	1.9405539714
F	-2.3750708008	-2.5142224279	3.6705628235
F	-3.4333589748	-2.7170810415	1.7917563604
C	-2.0601864904	-3.1960921463	-1.4275969068
C	-0.9053705952	-3.9616466488	-1.2644021306
C	-3.3168054494	-3.6927497154	-1.0854082883
C	-1.0118995831	-5.2503997033	-0.7406355713
H	0.0583286016	-3.5365638395	-1.5431553548
C	-3.4103491316	-4.9839163800	-0.5587958158
H	-4.1971095234	-3.0637402916	-1.2211772360
C	-2.2633760354	-5.7632324841	-0.3840219875
H	-0.1094973992	-5.8520485562	-0.6093565062
H	-4.3894922774	-5.3800173946	-0.2803148823
H	-2.3448889137	-6.7708385526	0.0293489969
C	-4.3627890622	0.7190657919	-0.6401206801
C	-5.4033165509	0.6221720413	0.2808652436
C	-4.1437194535	1.8722785828	-1.3903405365
C	-6.2600285685	1.7109153581	0.4457711536

H	-5.5084580581	-0.2914507383	0.8658539596
C	-5.0089523016	2.9541860657	-1.2073405353
H	-3.3005012958	1.9328161057	-2.0769174378
C	-6.0672429358	2.8784996925	-0.2998436673
H	-7.0772238223	1.6474374503	1.1680688024
H	-4.8353402602	3.8693632799	-1.7757557283
H	-6.7353053677	3.7318305409	-0.1641090443
C	3.0159354845	-2.7676985671	-1.0959160836
C	4.0704542759	-3.0009936382	-1.9950244224
C	2.5745224840	-3.8173948345	-0.2698667178
C	4.6634174066	-4.2613803269	-2.0719594723
H	4.4378987501	-2.2055276815	-2.6446209882
C	3.1685223296	-5.0745323335	-0.3471334776
H	1.7611555347	-3.6216549548	0.4299193684
C	4.2132272442	-5.2995243561	-1.2509142527
H	5.4795439995	-4.4345871842	-2.7766362681
H	2.8210953314	-5.8822581679	0.3011605731
H	4.6793857327	-6.2856409773	-1.3137877013
C	4.2253083233	0.1933455255	-1.3147844026
O	4.9159839184	0.9439061917	-1.9547359918
O	4.5812560441	-0.3564613815	-0.1501002862
C	5.8601822858	0.0128545260	0.3743821248
H	5.9462270604	1.1052481167	0.4300976940
H	5.9106280556	-0.4256240934	1.3772326383
H	6.6624593422	-0.3870946375	-0.2638132179

IN4Ba

0 1

C	-0.6503407794	-1.5886076594	1.6316621851
C	-1.0604213406	-1.6934489751	0.2741651344
C	-1.1559916970	-2.9276298715	-0.3821455119
C	-0.8441235956	-4.0678019210	0.3508179494
C	-0.4444605002	-3.9867636824	1.7022044679
C	-0.3422727074	-2.7601635641	2.3481101612
C	-0.6271004992	-0.1808421565	1.9623406232
C	-1.0716805731	0.4970910188	0.8314280572
H	-1.4699902212	-2.9956518016	-1.4228008651
H	-0.9073712167	-5.0447934466	-0.1323528054
H	-0.2074541713	-4.9041255692	2.2459678579
H	-0.0138854931	-2.7029567180	3.3860433882
N	-1.3191759616	-0.4169319768	-0.1899779055
C	-1.6788371660	-0.1260022725	-1.5729760881
H	-0.9430089411	-0.6617359343	-2.1970140983
C	-0.2199446728	0.3876968573	3.2906663589
H	0.0942891251	1.4353292156	3.2040501538
H	-1.0434617880	0.3535461501	4.0233943947
H	0.6153556351	-0.1898922344	3.7136961750
C	-1.4861366929	1.3723220569	-1.8688319748
H	-0.4206458962	1.5538591377	-2.0546802027
H	-2.0260090712	1.6326047682	-2.7889047331
C	-1.1454979418	1.9875275058	0.5782600389
C	-1.9424270140	2.2968449370	-0.7299902625
H	-1.6587498992	3.3218169036	-1.0021761154

C	-3.0566302472	-0.6646947722	-1.9546595724
C	-3.4530403854	-0.6206650768	-3.2945201819
C	-3.9415879499	-1.2352991478	-1.0287014732
C	-4.6963081161	-1.1040898355	-3.7136904272
H	-2.7797659526	-0.1975063023	-4.0455639148
C	-5.1802173999	-1.7252095918	-1.4263280768
H	-3.6594525947	-1.2871371369	0.0217670348
C	-5.5743968817	-1.6602515788	-2.7724748256
H	-4.9643518577	-1.0421989717	-4.7678162602
H	-5.8702561908	-2.1680369481	-0.7058194494
O	-6.8023123390	-2.1567774760	-3.0655828299
C	-7.2546815727	-2.1122208800	-4.3986206703
H	-6.6029194525	-2.6962654483	-5.0734960768
H	-8.2598710260	-2.5540885387	-4.4046348598
H	-7.3162202803	-1.0757487436	-4.7768678576
O	0.1822722901	2.4776656938	0.3813781880
H	1.3302558157	-0.3426752829	1.2596423128
N	2.3284610826	-0.4279859587	0.9841538699
P	2.7380727352	0.0632907959	-0.5845928417
S	3.3072353655	-1.1761348571	2.1067260312
O	1.5709009935	0.7437563968	-1.2069439954
O	4.1066945891	0.8795326887	-0.4608304957
O	3.3298499025	-1.2030577377	-1.3551109294
O	2.4637436278	-1.8597894358	3.0722677611
O	4.4217087732	-1.8127824651	1.4224492821
H	0.6266474696	1.9218610169	-0.2903616377
C	4.0333600619	0.2821730548	3.0504934044
F	3.0724962723	1.1629765487	3.3164138103
F	4.5394799462	-0.1776527976	4.1847169378
F	4.9866519687	0.8655278248	2.3415858228
C	2.5493538258	-2.3088655294	-1.7076489069
C	1.6741530005	-2.2136402884	-2.7894562229
C	2.7199291151	-3.4940444208	-0.9960293677
C	0.9695328964	-3.3536958310	-3.1837656267
H	1.5675815734	-1.2616412281	-3.3108593909
C	2.0069359508	-4.6241456493	-1.4031045202
H	3.4036599731	-3.5103664236	-0.1468404187
C	1.1409213007	-4.5593724152	-2.4980234404
H	0.2845136223	-3.2967115621	-4.0328127660
H	2.1282367068	-5.5601507554	-0.8541506666
H	0.5881173804	-5.4479540855	-2.8103037920
C	4.2395365904	2.2479551653	-0.2166036351
C	3.3022642547	2.9724752859	0.5191315206
C	5.3897179967	2.8474885767	-0.7275176937
C	3.5292526658	4.3347935177	0.7337364405
H	2.4002138838	2.5109291876	0.9190083352
C	5.6059595376	4.2070696645	-0.4958820859
H	6.0967876549	2.2397755671	-1.2939340781
C	4.6757518342	4.9555525014	0.2325600526
H	2.7925831239	4.9070034207	1.3017998768
H	6.5060373456	4.6829938294	-0.8916178237
H	4.8450778984	6.0199847718	0.4081206009
C	-1.7275982011	2.7924350936	1.7415455583
C	-1.3154691440	4.1221264549	1.9051334567
C	-2.6873046730	2.2711593658	2.6184711496

C	-1.8540900680	4.9148610449	2.9216451195
H	-0.5579557702	4.5254956937	1.2332462091
C	-3.2240707513	3.0616332042	3.6360443446
H	-3.0086740606	1.2370391203	2.5055081909
C	-2.8115981219	4.3880246031	3.7912220475
H	-1.5207706738	5.9494443392	3.0349417225
H	-3.9666301882	2.6357542129	4.3154371763
H	-3.2318524560	5.0057318401	4.5886065535
C	-3.4579282398	2.3380377953	-0.6466166016
O	-4.1342640596	3.0917426518	-1.3023634610
O	-3.9766168344	1.4284212051	0.1912365175
C	-5.3984781869	1.4136812475	0.3057773907
H	-5.8604339300	1.2016080595	-0.6682931614
H	-5.6378572417	0.6162240327	1.0188991737
H	-5.7608570983	2.3837679316	0.6760467602

IN4Bb

0 1

C	1.5093883230	2.0856209169	-0.0993728198
C	2.4518203671	1.6063501057	-1.0461146225
C	3.1144849309	2.4625446924	-1.9335353247
C	2.7974267823	3.8188158106	-1.8783705311
C	1.8596107671	4.3137164150	-0.9476853113
C	1.2180155373	3.4590359746	-0.0546470135
C	1.0455114842	0.9528345482	0.6663391320
C	1.7058575466	-0.1544314993	0.1723734274
H	3.8605470107	2.0887367926	-2.6363588641
H	3.2915797236	4.5118521434	-2.5634159587
H	1.6424985517	5.3846808436	-0.9258901737
H	0.5007547043	3.8454212223	0.6711826551
N	2.5693756660	0.2383700624	-0.8653615212
C	3.6774980140	-0.5393488949	-1.4066154278
H	3.6859506855	-0.3717778702	-2.4977406813
C	0.0405490485	1.0728073047	1.7687592809
H	-0.0046296709	0.1789180803	2.3971836274
H	0.3004778139	1.9206806841	2.4235013404
H	-0.9704298748	1.2888149383	1.3891584047
C	3.4328691090	-2.0440011271	-1.1912915359
H	2.7300835594	-2.4090595354	-1.9535873529
H	4.3764918048	-2.5865677952	-1.3340722419
C	1.5191936517	-1.6389075144	0.4291401227
C	2.8585002265	-2.3835269412	0.1897971417
H	2.6369306366	-3.4600932333	0.1985916354
C	5.0359216691	-0.0786188301	-0.8783548588
C	5.1772570597	0.7952008403	0.1996116720
C	6.2029229307	-0.5413037698	-1.5096213169
C	6.4394692921	1.1927256287	0.6546701745
H	4.2922173309	1.1745554525	0.7079169154
C	7.4641289211	-0.1592214132	-1.0711935332
H	6.1240334776	-1.2159166737	-2.3672748514
C	7.5942250105	0.7141018770	0.0220020021
H	6.5035183280	1.8784797996	1.4988574153
H	8.3711647600	-0.5197041206	-1.5593430212

O	8.8611449322	1.0387587575	0.3837651208
C	9.0547039995	1.9087488904	1.4736126306
H	8.6370489340	1.4943016955	2.4092312394
H	10.1397992827	2.0295546894	1.5912384072
H	8.6006703936	2.9001735631	1.2942827806
O	1.1301657579	-1.9377683218	1.7513918431
H	-1.5460405417	-1.2968195971	-0.4124075899
N	-2.3553816250	-0.7203536944	-0.1428765344
P	-2.6443224148	0.5576280736	-1.2535416765
S	-2.7930093376	-1.0096845977	1.4239236984
O	-1.4013205891	1.0991767286	-1.8278879222
O	-3.5987570922	-0.0854943925	-2.3759352704
O	-3.6530649009	1.5217503162	-0.4668521930
O	-1.7008526577	-1.7671683555	2.0349704526
O	-3.3811162110	0.1616846090	2.0451612975
H	0.1731371285	-1.8052388403	1.8532325719
C	-4.1804126960	-2.2630050435	1.2755988140
F	-3.8215697742	-3.2413212196	0.4524212419
F	-4.4128059770	-2.7615167023	2.4809447707
F	-5.2726589964	-1.6674895835	0.8227692287
C	-3.3675473737	2.7085031214	0.2192506727
C	-4.1868022008	2.9984750921	1.3087878163
C	-2.3530220966	3.5720969509	-0.1927334754
C	-3.9884461076	4.1929726212	2.0021530876
H	-4.9486325804	2.2782291854	1.6048316714
C	-2.1693685429	4.7639337950	0.5158173240
H	-1.7133853583	3.3117779547	-1.0356048279
C	-2.9807394097	5.0795516559	1.6079363141
H	-4.6227468112	4.4275402637	2.8599609565
H	-1.3796088865	5.4491255932	0.2002511243
H	-2.8279111611	6.0133917402	2.1531332335
C	-4.8009551093	-0.7526465814	-2.1603901486
C	-4.8221634431	-2.1371777322	-2.3171984864
C	-5.9550506296	-0.0359326066	-1.8424923264
C	-6.0279818084	-2.8208207513	-2.1476723533
H	-3.8949244262	-2.6568011877	-2.5545646799
C	-7.1540743796	-0.7319644980	-1.6744445741
H	-5.9018075415	1.0464953341	-1.7276577128
C	-7.1942165180	-2.1215402683	-1.8254477657
H	-6.0523195466	-3.9067059879	-2.2635595689
H	-8.0637305508	-0.1814663547	-1.4241044649
H	-8.1355481066	-2.6588605746	-1.6917311332
C	0.4946720481	-2.2351792054	-0.5656962509
C	0.1703189245	-1.6165596124	-1.7834992353
C	-0.1061413364	-3.4674227375	-0.2548336507
C	-0.7206007981	-2.2244318162	-2.6758825129
H	0.5789695638	-0.6366187099	-2.0230545089
C	-0.9952546873	-4.0726354024	-1.1445173069
H	0.1229891472	-3.9401837598	0.7009870391
C	-1.2953496577	-3.4587363803	-2.3651352525
H	-0.9763517481	-1.7125698595	-3.6056519938
H	-1.4582252204	-5.0264259588	-0.8815650687
H	-1.9809302553	-3.9389678885	-3.0673064219
C	3.9067486485	-2.2253818165	1.2859852800
O	4.7900817113	-3.0330403422	1.4466482744

O	3.7742575805	-1.1191489302	2.0215569351
C	4.7399593137	-0.9298787110	3.0519954632
H	4.6938250530	-1.7550496830	3.7780241330
H	5.7528028371	-0.8829680612	2.6271104186
H	4.4833266709	0.0201472197	3.5360490805

IN4Bc

0 1

C	2.3507109885	-2.4218370791	-1.6179079481
C	3.1497491039	-2.5881863284	-0.4522761811
C	4.0004259172	-3.6889893125	-0.2831072447
C	4.0208598834	-4.6449324462	-1.2955227317
C	3.2208754723	-4.5085752197	-2.4513609450
C	2.3887940153	-3.4065433784	-2.6212480646
C	1.6304828949	-1.1818508465	-1.4723439591
C	2.0183490106	-0.6435358096	-0.2604220580
H	4.6312637275	-3.7916355966	0.6013078203
H	4.6710030616	-5.5172158725	-1.1941352385
H	3.2612129728	-5.2815655322	-3.2223951172
H	1.7735832028	-3.3053666193	-3.5185222482
N	2.9154831501	-1.5089099120	0.3737967824
C	3.6058762586	-1.2698497341	1.6282947133
H	3.6146662182	-2.2217341464	2.1850454226
C	0.6269218677	-0.6585977854	-2.4507339589
H	0.0132010536	0.1416004175	-2.0221686840
H	1.1080692158	-0.2539544517	-3.3579499566
H	-0.0542294369	-1.4641333240	-2.7607446284
C	2.8033589260	-0.2531413063	2.4565801130
H	1.8649137958	-0.7088855447	2.8041714879
H	3.3949904251	0.0360332456	3.3364381559
C	1.4990404716	0.5808772462	0.4519228115
C	2.4888079349	0.9728537852	1.5939689687
H	3.4184402390	1.3215307858	1.1243746873
C	5.0497547699	-0.8305472725	1.4186453616
C	5.5035439911	-0.3295267566	0.1975329885
C	5.9598726700	-0.8984359455	2.4866119506
C	6.8218390069	0.1110895093	0.0342581261
H	4.8237787531	-0.2926885397	-0.6554787621
C	7.2720428776	-0.4669203595	2.3428358250
H	5.6344526468	-1.3007877121	3.4506314208
C	7.7156358777	0.0475060620	1.1115140967
H	7.1368612095	0.4928793860	-0.9361976585
H	7.9835468431	-0.5194470698	3.1687369767
O	9.0105044811	0.4448486815	1.0610896463
C	9.5157101170	0.9705971720	-0.1444939492
H	8.9728299512	1.8820555504	-0.4533755065
H	10.5669927319	1.2273135883	0.0410412547
H	9.4659742286	0.2334563656	-0.9661403651
O	0.2397713079	0.1844830312	1.0102067307
H	-1.1276860202	-0.2086379778	0.1437334015
N	-2.1601086780	-0.2460724923	-0.0786274659
P	-2.9715567989	1.0282159516	0.6946744496
S	-2.6476189433	-1.8101624913	-0.3697711517

O	-2.3186560976	1.4582243982	1.9545250198
O	-2.9806843953	2.2313690263	-0.3766137802
O	-4.5144158586	0.5966654292	0.7026848685
O	-1.4399738040	-2.5914339299	-0.5809076138
O	-3.7082428795	-2.2242031451	0.5359488214
H	-0.0090739869	0.7969465277	1.7335433056
C	-3.4778530874	-1.6638121564	-2.0452899790
F	-2.6211690424	-1.2215798953	-2.9573949539
F	-3.9076779865	-2.8695782356	-2.3933063875
F	-4.5134792335	-0.8322399785	-1.9709715026
C	-5.2851680470	0.0147579037	1.7104926068
C	-6.3511620858	-0.7738415000	1.2830047729
C	-5.0309102302	0.2403240196	3.0620388715
C	-7.1903101025	-1.3485613448	2.2373176347
H	-6.4919787806	-0.9379808197	0.2146283111
C	-5.8784918464	-0.3494324540	4.0053546791
H	-4.1849742273	0.8579265500	3.3624755271
C	-6.9568514654	-1.1394637388	3.6007157466
H	-8.0264792979	-1.9717744001	1.9122479872
H	-5.6889263129	-0.1833706057	5.0683805000
H	-7.6131829037	-1.5947254992	4.3454063593
C	-3.4142990576	2.1326859291	-1.6973458414
C	-2.4708310776	1.9307980115	-2.7029569601
C	-4.7721324888	2.2701214248	-1.9844109341
C	-2.9037659323	1.8547341849	-4.0287987855
H	-1.4183374020	1.8356712625	-2.4401977876
C	-5.1914634728	2.1928372174	-3.3139167347
H	-5.4789823613	2.4217811910	-1.1683056723
C	-4.2609016431	1.9830522916	-4.3367486866
H	-2.1721152481	1.6895814660	-4.8226671293
H	-6.2530450872	2.2947712822	-3.5504074541
H	-4.5947774465	1.9202170206	-5.3747624858
C	1.3397714938	1.7896534895	-0.4751961194
C	2.3016683151	2.0688895633	-1.4562029113
C	0.2698151743	2.6762098786	-0.3076679560
C	2.1823004741	3.1975876534	-2.2673808277
H	3.1393887197	1.3836630754	-1.5966929110
C	0.1472988230	3.8060207202	-1.1220262032
H	-0.4844688447	2.4929092338	0.4541815692
C	1.0998104787	4.0692248351	-2.1070912091
H	2.9356685138	3.3948241378	-3.0337545815
H	-0.7077668304	4.4716515058	-0.9859590066
H	1.0018268852	4.9485514832	-2.7478765507
C	2.0152433884	2.1578506963	2.4173252349
O	2.6736166157	3.1276748946	2.6806849329
O	0.7414611005	1.9895416341	2.8486690766
C	0.1143142343	3.0758137824	3.5428699297
H	0.3046552440	4.0192284861	3.0129725813
H	-0.9548634108	2.8377452074	3.5447185942
H	0.5133531207	3.1502573747	4.5649326662

IN4Bd

0 1

C	1.6025030148	2.3611317346	-0.3559855735
C	2.5452652600	1.8104388475	-1.2598008311
C	3.2230677384	2.5988203940	-2.1973400140
C	2.9108436660	3.9562246172	-2.2430671220
C	1.9627277928	4.5192897457	-1.3624868486
C	1.3110295374	3.7341380579	-0.4151614476
C	1.1449167427	1.2932384350	0.5017832759
C	1.8044371590	0.1481855659	0.0929598189
H	3.9746437760	2.1736007128	-2.8641888684
H	3.4167036877	4.5974629316	-2.9688185267
H	1.7487034595	5.5895050101	-1.4209982488
H	0.5886108620	4.1744901585	0.2737336670
N	2.6508708648	0.4572347486	-0.9869146829
C	3.8228803889	-0.3204108508	-1.3706891460
H	3.9431952240	-0.2208575870	-2.4617701525
C	0.1486578887	1.5214234686	1.5944746339
H	0.1049738094	0.6997488348	2.3133568614
H	0.4138133188	2.4321419235	2.1556642145
H	-0.8636274720	1.6997266857	1.2003481573
C	3.5840081196	-1.8027267833	-1.0637088685
H	2.8749137515	-2.2308299514	-1.7853701604
H	4.5315770873	-2.3429064063	-1.1936597024
C	1.6634623776	-1.3013558273	0.5393598893
C	3.0556544159	-2.0042389439	0.3549398856
H	3.7162673024	-1.5226419026	1.0901515747
C	5.0932466126	0.1985788634	-0.7094022182
C	5.0718292703	0.8542193916	0.5239292266
C	6.3358522537	-0.0142507261	-1.3264667872
C	6.2527358761	1.2805045079	1.1406332011
H	4.1185723990	1.0504141358	1.0184298016
C	7.5189918749	0.4024756687	-0.7278580722
H	6.3771975598	-0.5137239941	-2.2992293776
C	7.4874020322	1.0537197778	0.5170257210
H	6.1927189136	1.7909699995	2.1011370797
H	8.4874047179	0.2432899183	-1.2054941753
O	8.6871840483	1.4279578439	1.0282170654
C	8.7204292900	2.0880001042	2.2721363069
H	8.3014110618	1.4619560694	3.0805297199
H	9.7768303565	2.2927820644	2.4910615156
H	8.1679127511	3.0445691478	2.2443486172
O	1.3645649708	-1.3898117671	1.9118907177
H	-1.4083521574	-1.1578077761	-0.1922040329
N	-2.2137978501	-0.5484124071	0.0079652508
P	-2.5229986892	0.5656234227	-1.2613598910
S	-2.6107914296	-0.6126758821	1.6114159130
O	-1.2925528863	1.0212177792	-1.9298469918
O	-3.5010191856	-0.2236100901	-2.2644804653
O	-3.5147752843	1.6319935169	-0.5935566594
O	-1.5002358160	-1.2745048306	2.2960818734
O	-3.1890053843	0.6344790858	2.0748626435
H	0.4079633599	-1.2942544588	2.0496233933
C	-3.9998266419	-1.8714805446	1.6769032926
F	-3.6620052325	-2.9595265195	0.9945445637
F	-4.2030308950	-2.1908554753	2.9465379936
F	-5.1026919031	-1.3435647550	1.1691856237

C	-3.2210431242	2.9131872496	-0.1114994842
C	-3.9913729883	3.3562323433	0.9623033880
C	-2.2469559350	3.7169750942	-0.7030796768
C	-3.7848169238	4.6461580936	1.4527039672
H	-4.7216480595	2.6797100728	1.4045784268
C	-2.0546902391	5.0065416338	-0.1970847997
H	-1.6419565440	3.3374210627	-1.5258613780
C	-2.8179135847	5.4755384711	0.8744653952
H	-4.3806214946	5.0014550628	2.2964978276
H	-1.2949469831	5.6441335835	-0.6540425914
H	-2.6588528085	6.4844159946	1.2611732247
C	-4.6976624027	-0.8503486856	-1.9297507726
C	-4.7218770772	-2.2430939556	-1.8889090326
C	-5.8445000038	-0.0928962970	-1.6893969805
C	-5.9230380039	-2.8927516152	-1.5970089670
H	-3.7998473243	-2.7930417052	-2.0716348098
C	-7.0389385676	-0.7550180016	-1.3973015867
H	-5.7893254574	0.9947700737	-1.7292658241
C	-7.0819197329	-2.1518772643	-1.3499673200
H	-5.9494393917	-3.9840665464	-1.5578195426
H	-7.9427777870	-0.1721904233	-1.2060331124
H	-8.0196242278	-2.6623520349	-1.1201057290
C	0.6101517822	-2.0501285794	-0.3000576267
C	0.2879824258	-1.6625116491	-1.6098279882
C	-0.0120304277	-3.1892509352	0.2402018697
C	-0.6184356594	-2.4099621375	-2.3696811566
H	0.7106166354	-0.7488809584	-2.0245151752
C	-0.9155515456	-3.9343326760	-0.5185901683
H	0.2184347790	-3.4831755882	1.2645262685
C	-1.2103006258	-3.5552617270	-1.8322098955
H	-0.8732870742	-2.0770999560	-3.3777322049
H	-1.3945849839	-4.8124053822	-0.0797051725
H	-1.9079802604	-4.1458334362	-2.4307113997
C	2.9945819063	-3.4657996811	0.7620575282
O	3.0917598037	-3.8820416343	1.8875061153
O	2.8083122184	-4.2744111613	-0.3048117752
C	2.6617463632	-5.6618506642	-0.0231774919
H	1.7565583589	-5.8395327826	0.5778290833
H	2.5741346660	-6.1650770807	-0.9938338415
H	3.5294005327	-6.0438188680	0.5347727750

5B

0 1

C	-0.3787853970	-2.5206057982	2.8651967227
C	-1.5309202526	-2.0394654343	2.1905929273
C	-2.5534423080	-1.3962122554	2.9016070427
C	-2.4045809455	-1.2544969707	4.2827061543
C	-1.2713972338	-1.7378359415	4.9596849599
C	-0.2534801487	-2.3744931139	4.2544786542
C	0.5253786450	-3.0708483303	1.8779318601
C	-0.0616730558	-2.8817886364	0.6498288683
H	-3.4377632322	-0.9997852935	2.4163494560
H	-3.1959019091	-0.7538758966	4.8460348148

H	-1.1897103706	-1.6087336314	6.0413029622
H	0.6367123021	-2.7439180958	4.7682745952
N	-1.3402050879	-2.3032001228	0.8208897534
C	-2.1958683840	-2.0107310923	-0.2419592794
C	-1.7157944780	-2.0288982052	-1.5203319275
C	0.4115723430	-3.1917042315	-0.7477629793
C	-0.2448757039	-2.2349712433	-1.7615463535
C	-3.6142972436	-1.7275803145	0.1111531388
C	-4.4385708712	-2.7728314098	0.5433196723
C	-4.1180329666	-0.4211575782	0.0735307259
C	-5.7544095498	-2.5162026514	0.9328275696
H	-4.0411944305	-3.7892902217	0.5818698914
C	-5.4297480576	-0.1644990318	0.4684699457
H	-3.4748430181	0.4015042868	-0.2369508126
C	-6.2519672177	-1.2109387811	0.8986886910
H	-6.3915479499	-3.3377060140	1.2685059992
H	-5.8062486708	0.8600556852	0.4457876759
H	2.6002319755	0.8335681574	1.2220627165
N	2.2644240842	1.5707105068	0.5388034652
P	0.6477414756	1.3980479370	0.1547675457
S	3.4044136447	2.6096848361	-0.0681715904
O	0.1107020552	0.1526618824	0.7591518215
O	-0.0629459877	2.7689752751	0.6223482529
O	0.6580790863	1.4671973999	-1.4549965033
O	4.6767901623	1.9057873646	-0.1051249154
O	2.8598772713	3.3488885288	-1.1958038236
O	2.5625444912	-0.5318259062	2.1843068796
H	3.0259390123	-1.2404851947	1.7115323467
H	1.6183898347	-0.7413450629	2.0438875529
C	3.5320302511	3.8372288889	1.3429103490
F	3.8403033047	3.1930110683	2.4628958536
F	4.4809946058	4.7187749845	1.0594170354
F	2.3723175296	4.4611386829	1.4958369877
C	-1.4244643958	2.8651150084	0.9362085357
C	-1.9774000119	2.0846170473	1.9523524418
C	-2.1906867105	3.7752455399	0.2100098821
C	-3.3355841879	2.2309870447	2.2440770049
H	-1.3651059399	1.3578450894	2.4849339815
C	-3.5459734257	3.9129462746	0.5173117599
H	-1.7235768830	4.3465893399	-0.5927328981
C	-4.1207939380	3.1434758114	1.5340452735
H	-3.7786668858	1.6144848047	3.0284895696
H	-4.1551525222	4.6210593422	-0.0487411786
H	-5.1816819823	3.2520419614	1.7698854910
C	-0.3978662757	2.0064267755	-2.1902732357
C	-0.1784364878	3.1865269158	-2.8968259576
C	-1.6307851748	1.3607850010	-2.1971807924
C	-1.2383398735	3.7263071320	-3.6323024786
H	0.8036130906	3.6584662810	-2.8448880521
C	-2.6833139891	1.9100531324	-2.9314945349
H	-1.7547062330	0.4474300554	-1.6195077426
C	-2.4876012731	3.0941193498	-3.6497890656
H	-1.0862589251	4.6515970926	-4.1929038660
H	-3.6500790756	1.4018918262	-2.9334734381
H	-3.3091841757	3.5266541573	-4.2250830454

C	1.9216183950	-3.1232432839	-0.8893771318
C	2.5882464807	-1.9003701952	-0.6945616740
C	2.6915629326	-4.2341856586	-1.2302163451
C	3.9689179151	-1.7935608240	-0.7988900614
H	2.0036033412	-1.0195900909	-0.4422909684
C	4.0867747265	-4.1527538242	-1.3440152389
H	2.2028495740	-5.1980292726	-1.3972410758
C	4.7355258977	-2.9300860969	-1.1220746096
H	4.4747016507	-0.8356526645	-0.6541790309
H	4.6512342394	-5.0471018093	-1.6059564490
O	6.0747113835	-2.7452249325	-1.2021656179
C	6.8976739309	-3.8395951541	-1.5322625481
H	7.9295116904	-3.4647046152	-1.5415512706
H	6.8176497990	-4.6523843447	-0.7877229515
H	6.6553325289	-4.2486932403	-2.5297260724
H	-7.2801671095	-1.0085783486	1.2081508398
H	0.2601834912	-1.2567414658	-1.7133703016
H	0.0883936979	-4.2191980333	-0.9972301773
H	-0.0699634378	-2.6159418140	-2.7732516019
C	-2.5962910034	-1.7444547754	-2.6737283156
O	-3.7963654380	-1.5661861733	-2.6653813232
O	-1.8735291279	-1.6582206291	-3.8186353736
C	-2.5975066513	-1.3138284310	-4.9929123303
H	-3.4450075142	-1.9972695397	-5.1501247075
H	-2.9862692973	-0.2863221724	-4.9205415652
H	-1.8846261518	-1.3855384661	-5.8237895884
C	1.8098053690	-3.7475177385	2.2481422885
H	2.4227743947	-4.0061379098	1.3785229818
H	2.4082518982	-3.1087439283	2.9185076422
H	1.6056300663	-4.6785615757	2.8046432206

7B

0 1

C	3.5830292079	1.4081272131	2.5578051333
C	4.0443260048	0.0883000854	2.2780945804
C	4.4318096841	-0.7952476561	3.2961349139
C	4.3595464665	-0.3333318258	4.6065324411
C	3.9121521909	0.9744986906	4.9061130456
C	3.5249000483	1.8457428511	3.8956412209
C	3.2535767931	2.0298916162	1.3068995592
C	3.4970614543	1.0750656019	0.3137955761
H	4.7606063610	-1.8115185955	3.0730491651
H	4.6497313346	-0.9981494825	5.4236686627
H	3.8674832130	1.2969402455	5.9488207484
H	3.1723418042	2.8520435432	4.1333205386
N	4.0131762767	-0.0826848489	0.9193713530
C	4.3947337362	-1.2768503442	0.1906758948
H	5.2596250992	-1.7162561146	0.7147704975
C	4.8628324045	-0.8360769050	-1.2070845272
H	5.8094246283	-0.2774058742	-1.0998278858
H	5.0745539839	-1.7141505403	-1.8261957747
C	3.2502598402	1.0503171435	-1.1147529972
C	3.8177913749	0.0424513016	-1.8549829850

C	3.2883423573	-2.3198658432	0.1213872256
C	1.9968382824	-2.0849540565	0.5992401806
C	3.5642102172	-3.5651212434	-0.4708195200
C	0.9888966376	-3.0518052634	0.4602800304
H	1.7429966094	-1.1358399603	1.0765377769
C	2.5780601106	-4.5303771020	-0.6119909398
H	4.5729280973	-3.7821061959	-0.8336743441
C	1.2705437688	-4.2779289488	-0.1578435223
H	-0.0065545617	-2.8263034085	0.8350637555
H	2.7857450611	-5.4945573307	-1.0794281898
O	0.3719525157	-5.2664826518	-0.3617534479
C	-0.9805762889	-5.0406030635	-0.0212806216
H	-1.4015806194	-4.1849439280	-0.5740666141
H	-1.5297282684	-5.9480028052	-0.3038964755
H	-1.1042343647	-4.8703236238	1.0612782969
H	-0.9918661548	-0.9272794670	-0.8933506434
N	-1.6903301351	-0.7662467277	-0.1165052480
P	-1.3096183756	0.5012307827	0.9203121800
S	-3.1705390368	-1.4954456888	-0.3125746721
O	0.0908281213	0.5415931728	1.3917307440
O	-1.7118654566	1.8968278455	0.2056658104
O	-2.4656143429	0.2271511292	2.0177314202
O	-4.2783239191	-0.5556734308	-0.2320502510
O	-3.0177421259	-2.4280298814	-1.4210622488
O	0.3939433944	-0.7453254634	-1.7574827756
H	1.0897604377	-1.1602823348	-1.2237631458
H	0.8654552860	-0.3847520276	-2.5389399393
C	-3.3351548467	-2.5622312924	1.2631337837
F	-4.2432877799	-2.0403460291	2.0692206213
F	-3.7307897514	-3.7736076847	0.8847748852
F	-2.1773972695	-2.6682295656	1.9091377816
C	-2.7249534849	2.0585660396	-0.7400146659
C	-2.5212434589	1.6041906294	-2.0428975264
C	-3.9001028446	2.7020905802	-0.3617361862
C	-3.5381254655	1.7817228523	-2.9827237158
H	-1.5789272133	1.1236245507	-2.3062443690
C	-4.9063369039	2.8780296478	-1.3139047096
H	-4.0193170000	3.0401565796	0.6678651125
C	-4.7312009030	2.4137826576	-2.6207679613
H	-3.3931708821	1.4226281150	-4.0039157889
H	-5.8364509946	3.3744140297	-1.0279726983
H	-5.5252331135	2.5460687015	-3.3588422021
C	-3.0149739581	1.1935530020	2.8514101137
C	-4.4053528878	1.2900819441	2.8820625851
C	-2.1984724025	2.0177617235	3.6256051857
C	-4.9925099400	2.2409988795	3.7195750200
H	-4.9924377520	0.6314120322	2.2414658220
C	-2.8020941883	2.9708449345	4.4507883323
H	-1.1137117867	1.9050324480	3.5760974605
C	-4.1947415531	3.0837271061	4.5015533389
H	-6.0809322888	2.3275875514	3.7553854031
H	-2.1758123578	3.6258409613	5.0606897769
H	-4.6592341190	3.8285432183	5.1513199566
C	2.4276420173	2.1185182400	-1.7335186051
C	1.1580550265	2.4341226617	-1.2321372096

C	2.9336753733	2.8460144120	-2.8231268962
C	0.4052933316	3.4534115739	-1.8144725237
H	0.7778752934	1.8814110173	-0.3767207833
C	2.1806253626	3.8660258980	-3.4045416416
H	3.9312226225	2.6142347033	-3.2031558005
C	0.9120570985	4.1707986439	-2.9020636286
H	-0.5796728165	3.6908242868	-1.4114949749
H	2.5872065656	4.4286218045	-4.2484082217
H	0.3208931496	4.9712100044	-3.3536319930
C	3.3856962574	-0.2511443003	-3.2317956353
O	2.2608486122	-0.0944494532	-3.6845244475
O	4.3669004836	-0.7989727895	-3.9718997928
C	4.0098579184	-1.2127862034	-5.2900665685
H	3.2005153049	-1.9575861536	-5.2584962023
H	3.6681534196	-0.3551226676	-5.8882197455
H	4.9157923868	-1.6485885315	-5.7282539762
C	2.7665180593	3.4399328964	1.1914258808
H	2.9799606932	3.8810651250	0.2109568574
H	1.6769373979	3.4993545391	1.3483674078
H	3.2451224346	4.0651030913	1.9617471623

TS1Ba

0 1

C	-1.8581247242	-2.1926020540	-1.1483625128
C	4.4752178996	-1.3075634514	-1.3468421642
C	5.5380782802	-1.6011977572	-0.4514772274
C	6.0618650944	-0.5776219369	0.3077916100
C	5.5591651862	0.7552995634	0.1980676302
C	4.5322423784	1.0862659769	-0.6616158861
C	3.9820691726	0.0421777660	-1.4371686876
C	3.6725629188	-2.1003499421	-2.1894919047
C	2.6352450033	-1.2430367234	-2.6726163518
N	2.9531236293	0.0743914878	-2.3139021894
H	2.0631307354	-1.4113213453	-3.5852927365
H	5.9162832866	-2.6212437508	-0.3586240060
H	6.8718707556	-0.7801541510	1.0114907826
H	5.9974213411	1.5375057655	0.8226724869
H	4.1375581334	2.1001128973	-0.7232140476
H	2.2391430723	0.8274314398	-2.3313137784
C	3.7538477920	-3.5721694014	-2.3816142365
H	3.2313233395	-3.9011680432	-3.2902263165
H	3.2926934338	-4.0930782555	-1.5220450863
H	4.8000369691	-3.9089630915	-2.4364657326
O	-1.6628921413	-1.0448930457	-1.8300771361
C	-0.9052507774	-3.1634638089	-1.2865916400
C	1.0975735483	-1.6512566322	-1.3999937548
C	0.3878493457	-2.8339765645	-2.0195890731
H	0.1600418242	-2.5903881412	-3.0691394827
H	1.0144404397	-3.7326792308	-2.0064180711
C	1.6095129335	-1.6815571027	-0.0499467016
C	1.9092855635	-0.4635768676	0.5918608726
C	1.8403046462	-2.8852406054	0.6649515438
C	2.4121099560	-0.4171243937	1.8845980671

H	1.7083012045	0.4688303296	0.0726047193
C	2.3353648751	-2.8494291451	1.9539107712
H	1.5752063790	-3.8459888589	0.2200272440
C	2.6284314461	-1.6150948663	2.5769342542
H	2.5895758909	0.5520579020	2.3440244775
H	2.4918157373	-3.7644299684	2.5277381749
O	3.0921724161	-1.6845018473	3.8394511399
C	3.3289501081	-0.4821104119	4.5442481663
H	3.6618768350	-0.7704726030	5.5495062822
H	4.1173201553	0.1207716600	4.0598655823
H	2.4112765122	0.1255521856	4.6220171914
P	-0.3139854335	2.1435424297	-1.1414448231
O	0.8242726289	1.6705009187	-1.9925226087
O	-1.4627494294	2.7596459368	-2.1048667288
O	0.1285706640	3.4226128341	-0.2268266533
N	-1.0768986851	1.0427855777	-0.2256005150
S	-1.0393721315	0.7574996300	1.3457283596
O	-1.0455149582	-0.6839536924	1.5969418424
O	-0.1084534625	1.6016722021	2.1068850613
H	-1.6761069855	-0.2599433654	-1.2069860730
H	0.6474361353	-0.6963905750	-1.6668592150
C	-2.7341751916	1.3382283898	1.8801305725
F	-3.6833249684	0.6886821670	1.2110641905
F	-2.8881510632	1.1061673946	3.1823253776
F	-2.8601540068	2.6449945212	1.6553579663
C	1.4511562146	3.7959300099	-0.0444207511
C	2.0254955173	3.6285847356	1.2175005843
C	2.1767374784	4.3527962520	-1.1011188278
C	3.3457740569	4.0396645281	1.4219465366
H	1.4223897367	3.1696383342	1.9999371146
C	3.4967515861	4.7559806892	-0.8835490047
H	1.7050280036	4.4585761529	-2.0786642908
C	4.0849337762	4.6035566558	0.3770220915
H	3.7991364515	3.9165856964	2.4086914896
H	4.0680803554	5.1927405296	-1.7060523147
H	5.1160799973	4.9239853706	0.5424197346
C	-2.7560834162	3.0022713800	-1.6569204028
C	-3.7201104729	2.0041050522	-1.7971802120
C	-3.0638729860	4.2338224905	-1.0803813529
C	-5.0184485477	2.2462820350	-1.3463536620
H	-3.4356394865	1.0475819747	-2.2357638828
C	-4.3686223188	4.4678724721	-0.6397079809
H	-2.2775055273	4.9817232983	-0.9703065047
C	-5.3458942847	3.4766946909	-0.7687748637
H	-5.7722074561	1.4608163196	-1.4336705477
H	-4.6185461095	5.4279387433	-0.1821767347
H	-6.3618784663	3.6608238370	-0.4125031225
C	-0.9980430015	-4.5425450562	-0.7787557892
O	-0.0325994415	-5.2721387470	-0.6184926207
O	-2.2511952998	-4.9643205391	-0.5402850704
C	-2.3882924785	-6.2549529810	0.0411125343
H	-1.8502010264	-6.3131060390	0.9998562890
H	-3.4635159683	-6.4025403027	0.2000377130
H	-1.9881551033	-7.0326662589	-0.6269617147
C	-3.0993030725	-2.2294460013	-0.3407883038

C	-3.1095293438	-2.6663721887	0.9925224889
C	-4.2812416786	-1.7224900507	-0.9034044215
C	-4.2838097033	-2.6126093435	1.7388736731
H	-2.1800997916	-3.0005068709	1.4509730309
C	-5.4606025516	-1.6819359546	-0.1586626265
H	-4.2699913498	-1.3690632739	-1.9359911311
C	-5.4636491482	-2.1252333816	1.1656280681
H	-4.2759188704	-2.9346554495	2.7828610405
H	-6.3774395683	-1.2965696270	-0.6114028429
H	-6.3815714124	-2.0798965278	1.7568585617

TS1Bb

0 1

C	-1.7478326599	-2.3035128400	1.1461417817
C	2.4907389479	-1.7164327232	-2.4582766552
C	3.6713365742	-2.2542426829	-3.0312235013
C	4.7063742493	-1.3965977159	-3.3381755166
C	4.5919555487	0.0057920755	-3.1009183268
C	3.4462318253	0.5728995285	-2.5756906053
C	2.3830825905	-0.2943372421	-2.2513612749
C	1.3067433098	-2.2971987646	-1.9547869240
C	0.5553208451	-1.2354287757	-1.3716651808
N	1.1778557384	-0.0300953075	-1.6959238110
H	-0.5183814994	-1.2531993790	-1.1830930175
H	3.7600840071	-3.3289532625	-3.2049409584
H	5.6306127697	-1.7857608225	-3.7706712976
H	5.4385121836	0.6508528467	-3.3483468219
H	3.3666587073	1.6447240168	-2.3909807461
H	0.8793024840	0.8738231909	-1.2802829220
C	0.9276153602	-3.7362451934	-1.9890893754
H	-0.1606234865	-3.8652723167	-1.9173489526
H	1.3751042972	-4.2983927655	-1.1527772075
H	1.2765600843	-4.2043996599	-2.9220133171
O	-1.4386596237	-1.1201858375	1.7016878432
C	-0.7008033391	-3.1731512460	0.9529256181
C	1.1934536389	-1.4023279827	0.6578049128
C	0.7190376674	-2.7084407051	1.2336242927
H	0.5394758871	-0.5640928851	0.8830866505
H	1.4160594447	-3.5168658914	0.9972452369
H	0.8353734051	-2.5428766106	2.3258504050
C	2.5950744243	-1.0521225288	0.6466267882
C	2.9677458026	0.3098206665	0.7817093074
C	3.6224304831	-1.9950054087	0.4290949205
C	4.2924986744	0.6982528626	0.7227062539
H	2.1909092721	1.0626070807	0.9142439767
C	4.9551517615	-1.6136203257	0.3470445348
H	3.3697328632	-3.0479245517	0.2963562387
C	5.2994955866	-0.2547599686	0.4826665432
H	4.5795634373	1.7447989066	0.8312998119
H	5.7158292742	-2.3682221995	0.1542087166
O	6.5576084771	0.2168449478	0.3928009033
C	7.6100801679	-0.6703153079	0.0756436702
H	7.4425221697	-1.1598803978	-0.8996743899

H	8.5233644211	-0.0640234860	0.0236180027
H	7.7371683611	-1.4463810323	0.8503046171
H	-1.9203936252	-0.3592701539	1.2513136884
N	-2.0647672832	0.9676851394	0.1865565783
P	-0.9706551020	2.1347205037	0.4984717816
S	-2.5849591883	0.7499084880	-1.3198844345
O	0.3891960847	2.0631773645	-0.1385971742
O	-1.7163469384	3.5526098109	0.2450192164
O	-0.8654983803	2.0579216029	2.1258700729
O	-2.0406358159	1.7231409544	-2.2774310831
O	-2.5533110806	-0.6763026172	-1.6698771734
C	-1.0451976724	4.6740842197	-0.2206941955
C	-0.7397958933	4.7757490096	-1.5785438918
C	-0.7179144038	5.6869526554	0.6810083447
C	-0.0902870372	5.9240949029	-2.0357126262
H	-1.0219262329	3.9559073349	-2.2408464743
C	-0.0683446345	6.8318005900	0.2090797690
H	-0.9847154490	5.5692928553	1.7329445723
C	0.2481869286	6.9512416495	-1.1472389638
H	0.1521505516	6.0175563133	-3.0970934889
H	0.1883497648	7.6333349766	0.9059311799
H	0.7549416086	7.8470385169	-1.5134443232
C	0.3422723905	1.9229759862	2.7892955709
C	0.5766803439	0.7433745007	3.4985594580
C	1.2951434283	2.9443988216	2.7514727007
C	1.7868832866	0.5867709835	4.1790201524
H	-0.1841050642	-0.0372561673	3.4822053211
C	2.5023105522	2.7729291324	3.4329890160
H	1.0943392742	3.8493567913	2.1761772043
C	2.7530776117	1.5962064341	4.1459224042
H	1.9781387040	-0.3369870103	4.7303076581
H	3.2537790279	3.5654147107	3.4012327912
H	3.7017702440	1.4654040008	4.6707566179
C	-4.3991087714	1.1619076866	-1.1630784774
F	-4.5398056188	2.4301330952	-0.7908263007
F	-4.9926663940	0.9824846185	-2.3404158579
F	-4.9750847828	0.3746128933	-0.2601687128
C	-0.9203037350	-4.6033794731	0.6744503023
O	-1.9536112820	-5.1536345514	0.3622993458
O	0.2312620399	-5.3287346674	0.8220476729
C	0.1085445395	-6.7259421885	0.5859429197
H	-0.2242393385	-6.9242637207	-0.4447283025
H	1.1050257762	-7.1541742103	0.7540738468
H	-0.6224484746	-7.1790061325	1.2722012083
C	-3.1929234126	-2.4965312562	0.8894311660
C	-4.0974763558	-2.0203432285	1.8551178415
C	-3.6926580713	-3.0431751087	-0.3018454970
C	-5.4730094407	-2.1204377613	1.6495129905
H	-3.7118455573	-1.5749399368	2.7738109465
C	-5.0659268140	-3.1211893437	-0.5147340049
H	-3.0019735108	-3.3768131239	-1.0717085181
C	-5.9598187357	-2.6694117290	0.4614230843
H	-6.1647817847	-1.7575648994	2.4131231317
H	-5.4429385991	-3.5305724790	-1.4545435974
H	-7.0370088105	-2.7353670898	0.2894063184

TS2Ba

0 1

C	-1.6586558505	-0.5858903372	0.8508378545
C	-3.4818631848	-0.3770462031	-2.4833199630
C	-4.0786892794	-1.4695263327	-3.1214130773
C	-3.3312067000	-2.1823427321	-4.0601845344
C	-2.0077333465	-1.8138254074	-4.3701815274
C	-1.3992484696	-0.7172998362	-3.7597702966
C	-2.1588577725	-0.0129466457	-2.8242596015
C	-3.9143280894	0.5337669060	-1.4101159749
C	-2.8362573051	1.4815483040	-1.2755760341
N	-1.8196842312	1.1057331183	-2.0659627697
H	-3.4744970143	0.0259517672	-0.2702518653
H	-5.1034602861	-1.7645369817	-2.8844349336
H	-3.7780051212	-3.0416832303	-4.5652231352
H	-1.4472749702	-2.3949905644	-5.1057296791
H	-0.3793339683	-0.4176348786	-3.9985510260
H	-0.8641203565	1.4662565993	-1.9852433458
O	-0.6261149358	0.1055372709	0.6731274243
C	-2.8883144623	0.0970886789	1.1672224836
C	-2.6915448446	2.4269496692	-0.1362268319
C	-2.7984616045	1.6119807176	1.2407511285
H	-3.5810368144	3.0770355787	-0.1593931578
H	-3.6887024386	1.9725754973	1.7666272540
H	-1.9247619849	1.8880064202	1.8400500491
C	-1.4565634275	3.2991489806	-0.3237638317
C	-1.4770865925	4.2798928043	-1.3341577527
C	-0.2492556989	3.0844764624	0.3437307883
C	-0.3286034156	4.9734864771	-1.6969531398
H	-2.4098325001	4.4803661604	-1.8702483133
C	0.9158531750	3.7708896294	-0.0051608602
H	-0.1867950138	2.3277703343	1.1198540218
C	0.8911360430	4.6972963822	-1.0534422407
H	-0.3412498236	5.7153272756	-2.4975507849
H	1.8373255319	3.5277336730	0.5202610234
O	1.9863944950	5.3480138989	-1.5088426601
C	3.2510149647	4.9348483945	-1.0242658009
H	3.3667954845	5.1452261108	0.0531146882
H	4.0010902529	5.5078180213	-1.5850871533
H	3.4123502113	3.8555620091	-1.1878271531
H	0.8078390604	-0.0091620010	0.2777505252
N	1.8639556458	0.1357889777	0.0800716792
S	2.1808752511	0.5097837381	-1.4857320879
O	3.0859660142	2.0769216790	1.5557472636
O	3.5449462868	1.0028981111	-1.6181477928
O	1.0466153889	1.1977446535	-2.1046413523
P	2.8092150668	0.6346867333	1.4065537272
O	1.9870944069	-0.0847564280	2.6065685285
O	4.1552399208	-0.2322209087	1.3555258471
C	0.8897275715	0.4304396780	3.3001208067
C	4.3280845688	-1.5469108280	0.9486164341
C	-0.0934770416	-0.4848003595	3.6753542534

C	0.7783087436	1.7858118753	3.6123726002
C	3.5267689134	-2.5801959110	1.4369771811
C	5.3710449249	-1.7941420854	0.0566377921
C	-1.2306515753	-0.0305141397	4.3424810080
C	-0.3664721459	2.2253147184	4.2853173654
H	1.5656324920	2.4779285801	3.3169004681
C	3.7749860041	-3.8859930689	1.0043187839
C	5.6109321971	-3.1036731367	-0.3613101964
C	-1.3739833261	1.3272903763	4.6455705418
H	-2.0160693748	-0.7423631086	4.6017112230
H	-0.4677924539	3.2870951575	4.5208267057
C	4.8116744748	-4.1519210572	0.1057180286
H	3.1578576356	-4.7024657995	1.3868482170
H	6.4230740061	-3.3040400469	-1.0637903498
H	-2.2709089648	1.6833644991	5.1563845002
H	5.0001098118	-5.1750804409	-0.2262778446
C	-3.9738315402	-0.4940614685	1.9689211700
O	-5.0487525356	0.0388358551	2.1740994786
O	-3.6715786812	-1.7037310885	2.5006403783
C	-4.7207990608	-2.3626768297	3.1995296541
H	-5.5947536146	-2.5160218255	2.5476982548
H	-5.0436274276	-1.7743640622	4.0717672783
H	-4.3127316454	-3.3294614405	3.5198975482
C	-1.5640603352	-2.0460383133	0.5520913309
C	-0.3255024382	-2.6851220562	0.7222690914
C	-2.6259142273	-2.7684563212	-0.0139494926
C	-0.1558011195	-4.0192624810	0.3559016597
H	0.5117766989	-2.1226580266	1.1329990317
C	-2.4532666396	-4.0976469421	-0.3963672414
H	-3.5849108686	-2.2815852431	-0.1849611190
C	-1.2199161591	-4.7294645345	-0.2068943344
H	0.8166486755	-4.4970337544	0.4910518114
H	-3.2823008105	-4.6400388242	-0.8563942495
H	-1.0858318598	-5.7708128832	-0.5093803899
H	5.9616273991	-0.9527293820	-0.3076403363
C	2.1135619089	-1.1917881055	-2.2827004373
F	0.9913908553	-1.8045109459	-1.9187994914
F	3.1537373174	-1.9276412367	-1.9322008229
F	2.1142121174	-1.0271834189	-3.6038325692
H	2.7373923589	-2.3605542262	2.1548540436
H	0.0198221229	-1.5357269928	3.4095849501
C	-5.3649710395	0.8295853604	-1.0932677982
H	-5.8320533649	1.4279992868	-1.8913134082
H	-5.9309085520	-0.1086635880	-0.9964623142
H	-5.4707116931	1.3556970392	-0.1353858047

TS2Bb

0 1

C	-0.0906823553	-1.4472837818	-1.5091633170
C	-1.6071317719	-2.2371604124	2.1898584459
C	-1.7300751764	-3.4839836444	2.8177457405
C	-0.6991295435	-3.9062813306	3.6578783146
C	0.4400015285	-3.1055864392	3.8777014538

C	0.5766289622	-1.8555634196	3.2765199126
C	-0.4640970613	-1.4398214257	2.4445094577
C	-2.4309924616	-1.5106241348	1.2196980703
C	-1.7543716469	-0.2635480799	1.0193015240
N	-0.5968063396	-0.2702606891	1.7030650802
H	-1.8040753125	-1.7673279800	-0.0980620454
H	-2.6080186732	-4.1118776364	2.6490600424
H	-0.7717957687	-4.8770165880	4.1539315069
H	1.2365562296	-3.4740555868	4.5275467008
H	1.4682933058	-1.2442161133	3.4165442841
H	0.0928401168	0.5111750684	1.6367789529
O	0.4797536280	-0.2805840258	-1.5956364601
C	-1.5167256344	-1.4567692983	-1.4759157627
C	-1.9792027555	0.6984762725	-0.1111191690
C	-2.2252687568	-0.1030651758	-1.4274286073
H	-1.0342603752	1.2414699507	-0.2297429075
H	-3.2922238263	-0.3165695734	-1.5492600435
H	-1.9168081388	0.5371911333	-2.2653910932
C	-3.0260551141	1.7585739967	0.2057634004
C	-2.7838610871	2.6097204814	1.3004599312
C	-4.1720495972	1.9826417067	-0.5555100957
C	-3.6586934700	3.6364429132	1.6229362951
H	-1.8732520438	2.4755725122	1.8893055990
C	-5.0636182890	3.0194429136	-0.2482754446
H	-4.3913697101	1.3596546281	-1.4228974427
C	-4.8103599458	3.8546064023	0.8461672757
H	-3.4695333382	4.2998097169	2.4687875612
H	-5.9448943514	3.1615242595	-0.8729919943
O	-5.6036831551	4.8878056429	1.2266707253
C	-6.7709997235	5.1569033973	0.4870440089
H	-7.4710682460	4.3019124643	0.4984650506
H	-7.2544194149	6.0197539631	0.9640572086
H	-6.5421660171	5.4103199892	-0.5642497176
H	1.4137741259	-0.1801045489	-1.1355076994
N	2.5185153074	0.3655924639	-0.1640640332
S	3.2805189554	-0.6522256665	0.8377035275
O	0.9163164581	1.9215538755	1.3520592891
O	3.7404220313	-0.0016917658	2.0707548804
O	2.5721515183	-1.9284814513	0.9253319700
P	2.0225947213	1.8495376163	0.3442962629
O	1.6050751682	2.5170790738	-1.0868363821
O	3.2631123502	2.7473375054	0.8474007673
C	0.4132288113	3.1764571397	-1.3430666172
C	4.5597843916	2.7093698845	0.3573402034
C	-0.2682131926	2.8268515950	-2.5109507720
C	-0.0849982393	4.1600684100	-0.4858012943
C	5.5883538780	2.6959979765	1.2990023567
C	4.8279256333	2.7104836970	-1.0129044738
C	-1.4703683725	3.4672326039	-2.8183219793
C	-1.2930129574	4.7865681715	-0.8025290966
H	0.4551368486	4.4108598894	0.4268854660
C	6.9118744635	2.6938653641	0.8587292810
C	6.1589251192	2.6965341013	-1.4388980687
C	-1.9899118717	4.4432963866	-1.9627846410
H	-2.0099925966	3.1910603683	-3.7274123176

H	-1.6988102859	5.5393505652	-0.1242061791
C	7.2018278275	2.6919469959	-0.5095564798
H	7.7224135542	2.6799939133	1.5911002092
H	6.3778086914	2.6924816140	-2.5093313517
H	-2.9416847512	4.9256300049	-2.1921821498
H	8.2395542502	2.6819865947	-0.8501374780
C	-2.3099906757	-2.5399867355	-2.1374217847
O	-3.5221532594	-2.5617973251	-2.1536381870
O	-1.5618533322	-3.4862212946	-2.7251694073
C	-2.2641181610	-4.5824304326	-3.3064665966
H	-2.8682155713	-5.1046139620	-2.5492720333
H	-2.9338246624	-4.2364625051	-4.1076420240
H	-1.4956604237	-5.2524905182	-3.7100379021
C	0.7663288977	-2.6261494245	-1.3605246547
C	2.0079482427	-2.6586798478	-2.0173139706
C	0.4083191854	-3.6790437756	-0.5006716277
C	2.8707793560	-3.7346635982	-1.8288398166
H	2.2936645877	-1.8365114096	-2.6748771985
C	1.2842132226	-4.7388223922	-0.2966188703
H	-0.5300235639	-3.6383267729	0.0516367730
C	2.5136733197	-4.7713491823	-0.9632169449
H	3.8361789175	-3.7510224066	-2.3378550436
H	1.0167639357	-5.5328184290	0.4034606761
H	3.2033389939	-5.6016588400	-0.7944308841
H	4.0058633942	2.7221280179	-1.7288857593
C	4.8410890457	-1.0660852976	-0.1427669990
F	5.8120969148	-0.2084719383	0.1364090823
F	4.6047282203	-1.0360998530	-1.4564882056
F	5.2358563888	-2.2926603603	0.1904578276
H	0.1486366320	2.0456442862	-3.1468137900
H	5.3294346529	2.6660279700	2.3574842776
C	-3.8995899576	-1.7753300267	0.9985154687
H	-4.4503287649	-1.6335559555	1.9425673615
H	-4.3303155316	-1.0988203245	0.2514066785
H	-4.0748449015	-2.8036403617	0.6514007422

TS3B

0 1

C	1.3047054914	2.7499780210	-0.7789036390
C	-3.9437100500	-1.7278823701	-1.0622002944
C	-4.9031541635	-2.3793012938	-0.2831038242
C	-4.4708321537	-3.0940087292	0.8399891033
C	-3.1117228578	-3.1508324999	1.1811903561
C	-2.1424052681	-2.4873676668	0.4205867008
C	-2.5820382983	-1.7924582242	-0.6961749713
C	-4.0259847751	-0.9159707736	-2.2759202898
C	-2.7558485766	-0.5314982756	-2.5768310818
N	-1.8482676074	-0.9525902386	-1.5729549777
H	-2.3905511501	0.0585780466	-3.4131823553
H	-5.9626380032	-2.3339838195	-0.5445203530
H	-5.2026342279	-3.6170300341	1.4602465390
H	-2.7938739508	-3.7144204502	2.0600274352
H	-1.1001621151	-2.4893072308	0.7268760902

H	-0.8743278306	-1.2313564189	-1.8838578968
O	2.0289845708	1.6433571277	-0.9751088503
C	-0.0627775368	2.7632280358	-0.9054635234
C	-1.3632941885	0.6927115699	-0.4876705944
C	-0.7880039003	1.5910229562	-1.5477698381
H	-0.0777404043	1.0102014005	-2.1530378068
H	-1.5713010692	1.9827455726	-2.2081375467
P	1.6783390944	-1.7554313833	-1.3642349249
O	0.6281941699	-1.5273553802	-2.4155352413
O	3.1323915866	-1.4061277244	-1.9583865323
O	1.8666483613	-3.3266470730	-0.9841157283
N	1.4055559910	-0.8609312947	-0.0274614037
S	1.5218926704	-1.2284647354	1.5244413530
O	0.1946025174	-1.2121792634	2.1657348872
O	2.4326458582	-2.3292865097	1.8539792233
H	1.5988126354	0.8085505662	-0.6598789395
C	-2.5806928580	0.9563650760	0.2373113028
C	-3.6980374781	1.6059252537	-0.3418151564
C	-2.7185277722	0.4095499050	1.5327763706
C	-4.8979006831	1.6896921329	0.3406898756
H	-3.6125556786	2.0430273512	-1.3339179857
C	-3.9188060937	0.4915358363	2.2257947013
H	-1.8586618095	-0.0869774158	1.9869156747
C	-5.0251565140	1.1186238723	1.6240636665
H	-5.7699150875	2.1822011465	-0.0924815689
H	-3.9903577276	0.0554523093	3.2207226992
O	-6.2370365998	1.2270244492	2.1983446040
C	-6.4557978325	0.6508513929	3.4713398458
H	-6.2794075100	-0.4384417216	3.4553544693
H	-5.8100118807	1.1085688015	4.2401816978
H	-7.5063614988	0.8439787394	3.7227608154
H	-0.6031417882	0.1653156950	0.0911889018
C	2.3305945461	0.3152279718	2.2052938426
F	3.5195385144	0.5234416874	1.6477721940
F	1.5525289120	1.3756940606	1.9742033109
F	2.4836187426	0.1699873265	3.5162159861
C	4.2290183244	-1.0392799899	-1.1798112121
C	4.7777900319	0.2248227043	-1.3927396326
C	4.7573715907	-1.9152793530	-0.2326272148
C	5.8852580717	0.6173966970	-0.6397861381
H	4.3109065643	0.8903415691	-2.1177308672
C	5.8616915758	-1.5062507570	0.5192104012
H	4.2953327838	-2.8894331379	-0.0768983131
C	6.4284807862	-0.2451598700	0.3180796805
H	6.3140607483	1.6103357659	-0.7940784207
H	6.2736441765	-2.1806705872	1.2731913316
H	7.2896200843	0.0688903445	0.9123032219
C	0.7929573031	-4.1629095336	-0.6953068396
C	0.7009278732	-4.7034838012	0.5884561877
C	-0.1449610364	-4.4699175750	-1.6850229175
C	-0.3530933209	-5.5737662683	0.8791566167
H	1.4382620220	-4.4138821751	1.3367388511
C	-1.1961636878	-5.3352928552	-1.3749686647
H	-0.0447944145	-4.0193384752	-2.6723548977
C	-1.3027362499	-5.8900905664	-0.0966324610

H	-0.4335022453	-6.0003891515	1.8818139401
H	-1.9392114892	-5.5730565511	-2.1395651098
H	-2.1298822459	-6.5625944705	0.1403581965
C	2.1719026173	3.9254196972	-0.5313851061
C	3.3470677793	3.7546207913	0.2201649448
C	1.9010115813	5.1811913781	-1.0977203497
C	4.2097131109	4.8299614128	0.4340332236
H	3.5748965236	2.7744931812	0.6363746639
C	2.7685992255	6.2511663991	-0.8910441113
H	1.0157803058	5.3125226250	-1.7213015881
C	3.9221164660	6.0811051075	-0.1178562352
H	5.1132114097	4.6882307064	1.0318629310
H	2.5490675636	7.2220769252	-1.3412361590
H	4.6007425626	6.9220643310	0.0448378105
C	-0.8651135740	3.8990575252	-0.4074968557
O	-0.5732078632	4.6637155415	0.4827387442
O	-2.0545219803	3.9964815100	-1.0665885203
C	-2.9471983993	4.9923351182	-0.5803304815
H	-3.2117570239	4.7999730120	0.4704298144
H	-2.4944127514	5.9927975112	-0.6465908180
H	-3.8435108478	4.9369296046	-1.2112010498
C	-5.2826780492	-0.5878910555	-3.0074713300
H	-5.9636276236	-0.0124632003	-2.3568463858
H	-5.0853345853	0.0072107947	-3.9104431296
H	-5.8205104157	-1.5028706560	-3.3059190729

TS4Ba

0 1

C	3.2919862070	-1.6820026285	2.3161736574
C	3.5952476377	-0.5905248416	1.4183196332
C	4.9390890826	-0.2443774116	1.1368887888
C	5.9305143506	-0.9835768113	1.7537023362
C	5.6471022759	-2.0624911456	2.6412813866
C	4.3461280788	-2.4126258717	2.9245693624
C	1.8954191728	-1.8269909101	2.3590572030
C	1.3581448983	-0.8873458733	1.4235690045
H	5.1853006046	0.5684043797	0.4581523675
H	6.9746808650	-0.7330187367	1.5505643411
H	6.4757634444	-2.6122222513	3.0916869498
H	4.1186233916	-3.2422277121	3.5967031488
N	2.4225563162	-0.0836286978	0.9609905018
C	2.1122008906	0.9029933190	-0.0877325160
H	1.1761341044	1.3704261080	0.2551899407
C	1.0822566011	-2.8084376511	3.1171541536
H	0.2610605480	-3.1771754162	2.4821908353
H	0.6088814788	-2.3265265027	3.9901996114
H	1.6830769120	-3.6567405888	3.4726648665
C	1.7896631011	0.1538807511	-1.3784350781
H	2.6744849647	-0.3255796766	-1.8125030854
H	1.4435386260	0.8829863402	-2.1212132772
C	0.7859383555	-1.9492055653	-0.0876292289
C	0.6569067512	-0.8498578573	-1.1797605199
H	-0.2387498559	-0.2875380942	-0.9021744875

C	3.1606124217	1.9859547001	-0.1978378632
C	3.9332395865	2.1884847073	-1.3509637404
C	3.3797516547	2.8262100856	0.8996287644
C	4.9045924743	3.1844749527	-1.3961501623
H	3.7848877596	1.5614813082	-2.2307522964
C	4.3525353458	3.8264100713	0.8740335876
H	2.7817153364	2.6887655219	1.8042364507
C	5.1285240277	4.0100063035	-0.2823288561
H	5.5084263475	3.3471769832	-2.2905540265
H	4.4937160917	4.4561397886	1.7517380813
O	6.0961006524	4.9469253273	-0.4144702478
C	6.3620593374	5.8155877342	0.6642139224
H	5.4786565442	6.4245671650	0.9265931638
H	7.1706557273	6.4821237869	0.3372073397
H	6.6925741664	5.2636931582	1.5623601996
O	-0.3754651054	-2.4528231593	0.3103938290
N	-2.4160319400	-0.7971278197	0.1781268335
P	-2.2658243058	0.7576109740	0.6651255522
S	-3.7262749557	-1.4371226884	-0.5125819793
O	-0.9193334932	1.0192457742	1.2642699843
O	-3.5256239745	1.0691625440	1.6260835490
O	-2.5983714733	1.7965675683	-0.5524433419
O	-3.3768756780	-2.7254350347	-1.1099661160
O	-4.5241635039	-0.4688560092	-1.2804009877
C	-4.8248404271	-1.9227664941	0.9411004752
H	0.4000664177	-0.3653850296	1.5470675353
H	-1.1674711419	-1.8039766424	0.2344125028
C	-1.7420897115	1.9458943156	-1.6254807048
C	-1.9307301172	1.1598733863	-2.7650742812
C	-0.7165208642	2.8929620846	-1.5573630499
C	-1.0734622536	1.3345774791	-3.8546492599
H	-2.7430262541	0.4328331329	-2.7690410962
C	0.1254039899	3.0665321487	-2.6590481169
H	-0.5979424406	3.4775386934	-0.6442203366
C	-0.0496527388	2.2873608065	-3.8080833301
H	-1.2075795894	0.7217085514	-4.7488052717
H	0.9301025085	3.8038803288	-2.6120863401
H	0.6149660202	2.4171940909	-4.6648580986
C	-4.3186552995	2.2089648969	1.6174721724
C	-5.3678105168	2.3025929093	0.7022156594
C	-4.0696098501	3.2114130080	2.5531534380
C	-6.1878352710	3.4316759880	0.7379445998
H	-5.5135141493	1.4992760058	-0.0209973835
C	-4.8995839174	4.3360877244	2.5776166849
H	-3.2352687231	3.0954238986	3.2470383583
C	-5.9585804226	4.4480961636	1.6723218661
H	-7.0124972709	3.5180289043	0.0264352378
H	-4.7156475797	5.1272555086	3.3085258019
H	-6.6057286506	5.3279341528	1.6937586977
F	-4.0819342005	-2.3833594062	1.9462184844
F	-5.5454724224	-0.8915672813	1.3720844473
F	-5.6537877765	-2.8839220655	0.5410652267
C	1.8156634425	-3.0278614051	-0.2827723898
C	1.4496407314	-4.3559432668	0.0022146099
C	3.1291268820	-2.7756160896	-0.7134226684

C	2.3745949368	-5.3920083213	-0.1173262040
H	0.4240177306	-4.5624063982	0.3039464235
C	4.0556082274	-3.8134843641	-0.8227998574
H	3.4505246639	-1.7723374602	-0.9656073375
C	3.6859924348	-5.1247258867	-0.5212721792
H	2.0661348294	-6.4171941723	0.1010175395
H	5.0730616327	-3.5886829312	-1.1497692939
H	4.4118273540	-5.9363428077	-0.6109669132
C	0.3319591421	-1.5425780618	-2.5039508068
O	1.1072470252	-1.6819531122	-3.4182310461
O	-0.9279709442	-1.9641170789	-2.4980228288
C	-1.4083640053	-2.6857465591	-3.6316804188
H	-2.4243455180	-2.9915813444	-3.3617055689
H	-1.4086979614	-2.0390365067	-4.5223657208
H	-0.7692647983	-3.5588858900	-3.8285807931

TS4Bb

0 1

C	-3.9222147770	-2.4013441526	-1.1625818172
C	-4.0673870688	-2.3230297675	0.2705006602
C	-5.2028799959	-2.8551469988	0.9179783142
C	-6.1608815108	-3.4637044068	0.1262677711
C	-6.0329127914	-3.5552247975	-1.2896507328
C	-4.9297704939	-3.0346695203	-1.9335210361
C	-2.7220093958	-1.7454278980	-1.5019390304
C	-2.1968787679	-1.2019747281	-0.2900913178
H	-5.3228543288	-2.7916115107	2.0001279839
H	-7.0478341371	-3.8896063278	0.6017082746
H	-6.8236111719	-4.0431956341	-1.8626321130
H	-4.8298909446	-3.0965945528	-3.0189412399
N	-2.9942535848	-1.6669420054	0.7743235037
C	-2.7524163157	-1.1697563768	2.1312877594
H	-3.4431449921	-1.7127491647	2.7953699711
C	-2.0982956981	-1.5415623063	-2.8287112080
H	-1.0099295540	-1.6855167873	-2.7552410109
H	-2.5301289142	-2.1988392060	-3.5958078030
H	-2.2489409351	-0.4930449859	-3.1450089104
C	-3.1189852934	0.3191247138	2.1467394553
H	-4.2032434432	0.4409542274	2.0357597984
H	-2.8862106733	0.7546389763	3.1251537372
C	-2.4829955614	0.7476903452	-0.4007866339
C	-2.3497541077	1.1194831545	1.0932993593
H	-1.2805055580	0.9996680312	1.3125948517
C	-1.3094391982	-1.4648331072	2.5334886444
C	-0.7246130294	-2.6947343407	2.1812220588
C	-0.5078467198	-0.5294841895	3.1937203085
C	0.6108271294	-2.9611381902	2.4470684840
H	-1.3196769846	-3.4444021088	1.6539901801
C	0.8396259756	-0.7782075747	3.4614060498
H	-0.8974633572	0.4460351789	3.4808550469
C	1.4168688371	-1.9924625412	3.0713199727
H	1.0711789598	-3.9034470012	2.1460671960
H	1.4360190961	0.0072587760	3.9205995337

O	2.7185648956	-2.3032264707	3.2349578819
C	3.5977611853	-1.2984150297	3.7116464011
H	3.3943172379	-1.0618559553	4.7714664502
H	4.6114381105	-1.7079275102	3.6189268964
H	3.5176336081	-0.3793593169	3.1111985344
O	-1.4605032094	1.1367378226	-1.1490695685
N	0.8142181276	0.8667732212	0.2281132408
P	1.6291385973	-0.3397578016	-0.5129958598
S	1.3998772123	1.9611530469	1.2403395241
O	0.7270330697	-1.3439137494	-1.1469798525
O	2.6125488134	0.4392400968	-1.5597748202
O	2.6426365099	-0.9928054851	0.5711449365
O	0.3146631694	2.4801674695	2.0823863514
O	2.6790014688	1.6169024702	1.8727175704
C	1.8208866052	3.4057794040	0.1134599596
H	-1.1177404715	-1.1393228395	-0.1361838785
H	-0.5970379281	1.1411184878	-0.6203404064
C	3.4399136990	-0.0993138772	-2.5251761292
C	3.3334389813	-1.4180396979	-2.9753524789
C	4.4128142479	0.7611261642	-3.0404733070
C	4.2315109869	-1.8736582221	-3.9447737013
H	2.5567063053	-2.0679884557	-2.5724381605
C	5.2970523572	0.2926603786	-4.0119752340
H	4.4602284928	1.7820013462	-2.6586244012
C	5.2134890408	-1.0279167885	-4.4660136241
H	4.1567126959	-2.9062210727	-4.2945313025
H	6.0607076930	0.9637677957	-4.4125444910
H	5.9107070837	-1.3947915969	-5.2223763976
C	3.9912524579	-1.2587762478	0.4175854956
C	4.3938089083	-2.5904563186	0.3219046884
C	4.9164372921	-0.2142353263	0.4091264729
C	5.7539550395	-2.8810342707	0.1997094473
H	3.6358571256	-3.3737347551	0.3498062984
C	6.2728709592	-0.5187197284	0.2776270180
H	4.5594290341	0.8098821305	0.5101382783
C	6.6956067889	-1.8473238097	0.1710483434
H	6.0787646066	-3.9213674991	0.1224762199
H	7.0049092877	0.2921082963	0.2610108129
H	7.7583952478	-2.0775758700	0.0682054220
F	1.9413264061	4.5122765300	0.8444322283
F	0.8571542891	3.5937817281	-0.7900046962
F	2.9693041137	3.1827145502	-0.5239010855
C	-3.7943300839	0.9729603268	-1.0907844855
C	-3.7838369440	1.6042127393	-2.3483920799
C	-5.0316462006	0.5831396359	-0.5485018666
C	-4.9713049867	1.8243513705	-3.0454216112
H	-2.8305201592	1.9366650198	-2.7564574671
C	-6.2168354637	0.7965125373	-1.2529023201
H	-5.0885318511	0.0997015174	0.4202457318
C	-6.1931250233	1.4150325742	-2.5044889267
H	-4.9414814562	2.3243737063	-4.0163851813
H	-7.1643818573	0.4750620702	-0.8152231056
H	-7.1230298708	1.5846762056	-3.0522368039
C	-2.5738577223	2.6194598849	1.3095450118
O	-3.1611377497	3.0769323496	2.2580115747

O	-1.9833806141	3.3505151241	0.3643617343
C	-1.8744131158	4.7524322015	0.6087322976
H	-1.2008087439	4.9221627672	1.4616727500
H	-1.4434220540	5.1837729375	-0.3017720988
H	-2.8585279398	5.1917415661	0.8250477077

TS4Bc

0 1

C	-0.9694225893	-1.3055544616	2.0777194130
C	-1.3307014308	-1.9213508780	0.8241041525
C	-0.5442996028	-2.9549424847	0.2751966417
C	0.5500485858	-3.3749797760	1.0061678981
C	0.8939946305	-2.8064407775	2.2689001803
C	0.1531074757	-1.7831944566	2.8074358198
C	-1.8555627193	-0.2441731648	2.3032640941
C	-2.6860290435	-0.1575436408	1.1346077302
H	-0.7588904411	-3.3789152705	-0.7050421268
H	1.1963893839	-4.1526505045	0.5957130388
H	1.7864881550	-3.1654227293	2.7795327151
H	0.4443775612	-1.3014533198	3.7401170392
N	-2.4596603071	-1.3228160497	0.3585230626
C	-3.0662982512	-1.4627254765	-0.9663879151
H	-2.7177074583	-2.4283942250	-1.3625608743
C	-1.8546906360	0.7007122299	3.4481376988
H	-0.9374402067	1.3123319448	3.4096896918
H	-2.7307354485	1.3606212243	3.4428259567
H	-1.8230313054	0.1490960864	4.4012929745
C	-2.5460325401	-0.3533971171	-1.8907933838
H	-1.4836342574	-0.5342615106	-2.1017238171
H	-3.0909615221	-0.3703647119	-2.8427200833
C	-1.7604768853	1.1257622904	-0.0046628752
C	-2.6774037766	1.0344941607	-1.2362782754
H	-3.7208881600	1.2082463448	-0.9453502757
C	-4.5845859952	-1.5259904346	-0.8211593504
C	-5.1385471407	-2.3740962401	0.1562808911
C	-5.4634927304	-0.7946639587	-1.6245433636
C	-6.5112492359	-2.4880591298	0.3212154219
H	-4.4724861162	-2.9502982513	0.8030147918
C	-6.8521202135	-0.8964457230	-1.4700969734
H	-5.0861756651	-0.1171421671	-2.3916195592
C	-7.3863514634	-1.7468183147	-0.4940930921
H	-6.9410138252	-3.1471910650	1.0775563439
H	-7.4987017957	-0.3047064084	-2.1170028552
O	-8.7087663871	-1.9168318327	-0.2619688127
C	-9.6381374407	-1.2071956813	-1.0501005269
H	-9.5467233296	-1.4640064878	-2.1205823223
H	-10.6357889595	-1.4983410687	-0.6965483675
H	-9.5211924451	-0.1145623207	-0.9362530024
O	-0.6091989821	0.5631684226	-0.2906531793
N	1.7251626550	0.3040370020	0.7230110736
P	2.4317099248	-0.3627520925	-0.5985199137
S	2.3328198830	0.6222972900	2.1596851548
O	1.5148413387	-1.1140120794	-1.4967331739

O	3.1602905341	0.9336974525	-1.2731569045
O	3.7670007127	-1.1976106394	-0.1593650479
O	1.2569566183	1.1572362678	3.0076074470
O	3.2191724137	-0.4030286650	2.7163856175
C	3.4561463592	2.1038855266	1.8984014654
H	-3.7036730370	0.2316367528	1.1678004085
H	0.2041769633	0.6031954119	0.3264328434
C	3.9919113595	0.8533444553	-2.3737870665
C	3.6028567355	0.1834777366	-3.5381904788
C	5.2271359819	1.4997288319	-2.2914202119
C	4.4734489254	0.1672166782	-4.6314289058
H	2.6324611042	-0.3133931517	-3.5651095826
C	6.0827100413	1.4814249601	-3.3944656874
H	5.4936528618	2.0001284741	-1.3595739828
C	5.7107599764	0.8146280040	-4.5665864988
H	4.1769900285	-0.3558121631	-5.5441230889
H	7.0499171900	1.9864012226	-3.3343557345
H	6.3844556375	0.7988950924	-5.4262841186
C	3.8054645533	-2.5674283701	0.0172080476
C	3.4967165651	-3.4351777724	-1.0346299326
C	4.1975807773	-3.0544813326	1.2659412046
C	3.5758764598	-4.8136951732	-0.8213863894
H	3.1805218218	-3.0221981963	-1.9915850639
C	4.2759335124	-4.4351103018	1.4629825995
H	4.4018608853	-2.3352594619	2.0586884135
C	3.9618867291	-5.3191201483	0.4247044287
H	3.3340394106	-5.4976119282	-1.6387552895
H	4.5804037558	-4.8223396132	2.4385321847
H	4.0231995172	-6.3980652470	0.5844117718
F	3.8498613962	2.5729218622	3.0794427443
F	2.8049780341	3.0761120478	1.2497582825
F	4.5309130525	1.7617519930	1.1906589225
C	-1.7771687080	2.4146166300	0.7547452283
C	-0.5681332836	3.0114042499	1.1340067473
C	-2.9859913384	3.0518303100	1.0785491670
C	-0.5664179085	4.2112922532	1.8458336017
H	0.3780753085	2.5457021799	0.8718644409
C	-2.9829814962	4.2538126350	1.7854164829
H	-3.9402300540	2.6066831086	0.7879183287
C	-1.7712487741	4.8348503834	2.1766753897
H	0.3884923970	4.6504595959	2.1414823969
H	-3.9297179014	4.7386434738	2.0344259989
H	-1.7697015421	5.7732145721	2.7360739570
C	-2.3932329524	2.1004554932	-2.2918155951
O	-3.1859382383	2.3680425120	-3.1630329489
O	-1.1902518231	2.6586639694	-2.1712427699
C	-0.8325922395	3.6453903466	-3.1401676354
H	-0.8608671532	3.2180826840	-4.1531168118
H	-1.5268011174	4.4975655521	-3.0960211238
H	0.1849636604	3.9622802604	-2.8847969167

TS4Bd

0 1

C	1.1538709528	0.5956368301	-2.3362888732
C	0.9345764759	1.6751712199	-1.4026943845
C	-0.3479852152	2.2568455096	-1.2818226950
C	-1.3435890650	1.7847013371	-2.1149238607
C	-1.1255040067	0.7500719024	-3.0701936971
C	0.1050469872	0.1521866916	-3.1850410152
C	2.4693309708	0.1385060688	-2.1699510862
C	3.0177936538	0.8910636550	-1.0872318787
H	-0.5620197193	3.0234488854	-0.5441436120
H	-2.3468013643	2.2040511956	-2.0245885978
H	-1.9623599451	0.4077247066	-3.6774476949
H	0.2702493250	-0.6813322294	-3.8669773891
N	2.1177646900	1.9323898174	-0.7680848629
C	2.4028207293	2.7728950272	0.4054459018
H	3.4587544391	3.0569498954	0.2865271475
C	2.2961763422	1.9477132990	1.6960457790
H	1.2483310918	1.6768189336	1.8801837264
H	2.6479225412	2.5627470495	2.5381379470
C	2.5798379691	-0.2798218891	0.5110400200
C	3.0950547038	0.6309538791	1.6535676214
H	2.8419809397	0.0547108736	2.5565066772
C	1.5819649606	4.0468580529	0.3942860517
C	0.6264971522	4.3548659400	1.3739086533
C	1.7562339865	4.9476510485	-0.6618609721
C	-0.1348101753	5.5165652503	1.2913460239
H	0.4465687496	3.6730490112	2.2051511112
C	0.9977597734	6.1144383637	-0.7643353946
H	2.4905884045	4.7230695070	-1.4398238734
C	0.0386338720	6.4058924797	0.2190080850
H	-0.8843458988	5.7563406272	2.0473196773
H	1.1597735564	6.7840680214	-1.6082454819
O	-0.7514915921	7.5052593455	0.2161094779
C	-0.6395647339	8.4267245926	-0.8447675282
H	0.3694852237	8.8738910482	-0.8947868641
H	-1.3717725703	9.2208350784	-0.6485272118
H	-0.8692691636	7.9591508237	-1.8189781636
O	1.2753204719	-0.2617112839	0.5739329804
N	-0.6709179837	-1.5603149134	-0.3920087045
P	-1.8607340676	-0.9771916518	0.5777009512
S	-0.7454544664	-2.6170526414	-1.5859838310
O	-1.5953966373	0.3673502536	1.1540608064
O	-2.0198647972	-2.1832090461	1.6679856888
O	-3.3088511774	-1.1270298790	-0.1613025541
O	0.6050790392	-2.7730806118	-2.1426212262
O	-1.8765091046	-2.4546457744	-2.5023372926
C	-1.0784614727	-4.2729132148	-0.7604964866
H	4.0786042290	1.0827462316	-0.9333727715
H	0.6574236486	-0.8963415691	0.0303815755
C	-3.9471500104	-0.1156106918	-0.8532818944
C	-4.2758287703	-0.3488303418	-2.1900425955
C	-4.2847164050	1.0846041312	-0.2213139202
C	-4.9523233078	0.6416754533	-2.9055516773
H	-3.9639476134	-1.2907413663	-2.6409472741
C	-4.9574956280	2.0677053121	-0.9512157768
H	-3.9971773100	1.2401659910	0.8176739184

C	-5.2918355158	1.8528756179	-2.2924918914
H	-5.2103516174	0.4668186099	-3.9530009943
H	-5.2209948292	3.0102713333	-0.4650768404
H	-5.8185964255	2.6254756001	-2.8572878592
C	-3.0159105635	-2.2141078907	2.6262927084
C	-3.7420775428	-3.3995901597	2.7576347339
C	-3.2682254685	-1.1161397576	3.4547303435
C	-4.7312787424	-3.4892635787	3.7386881801
H	-3.5193460099	-4.2266375084	2.0825028317
C	-4.2672157237	-1.2181971802	4.4266908427
H	-2.6826502643	-0.2057092018	3.3221620033
C	-4.9989798700	-2.4000594940	4.5745661814
H	-5.3014306626	-4.4153504079	3.8454067774
H	-4.4711514785	-0.3635399838	5.0766093030
H	-5.7765814034	-2.4725330515	5.3382956679
F	-0.2433079468	-4.4711680319	0.2661393776
F	-2.3300548171	-4.3305634151	-0.3100695963
F	-0.8950805915	-5.2472183694	-1.6477664954
C	4.5958540893	0.8383027970	1.6954140041
O	5.2369008834	1.5411635558	0.9426012670
O	5.1517432434	0.1452928177	2.6932831017
C	6.5742015333	0.2217964886	2.8125846867
H	6.8343430552	-0.3498434919	3.7107693570
H	7.0549312763	-0.2207170812	1.9271819675
H	6.9014447993	1.2670853553	2.9071658524
C	3.2688198068	-1.5875617218	0.2820357298
C	2.5167186141	-2.7663059115	0.3927395783
C	4.6402684400	-1.6804214576	-0.0144374208
C	3.1178569348	-4.0112502352	0.2008261003
H	1.4563458130	-2.7160155390	0.6296051406
C	5.2392171781	-2.9249404661	-0.1971378830
H	5.2409216893	-0.7798720289	-0.1423228657
C	4.4791869855	-4.0958012073	-0.0921867763
H	2.5060238315	-4.9121475139	0.2749663389
H	6.3034892518	-2.9822718050	-0.4372433498
H	4.9494862769	-5.0697029521	-0.2462396296
C	3.1250056135	-0.9844482233	-2.8890770348
H	4.2154986323	-0.9735338142	-2.7697088918
H	2.7298822970	-1.9417160764	-2.5097681963
H	2.8802105501	-0.9495988556	-3.9618030297

ECb

0 1

C	-2.0258895356	-1.5799525324	-1.4791308888
O	-0.9580557516	-2.1213641463	-1.2213389610
C	-2.0887738302	-0.0822245781	-1.6252291800
C	-2.0717381030	0.6857877857	-0.2551718619
C	-3.2660430102	0.7305694305	-1.1250660745
H	-1.4208089316	1.5597249622	-0.2856131275
H	-4.1544919411	0.1829803879	-0.8128877300
H	-3.4552597178	1.6637983910	-1.6548175271
C	-1.9919018462	-0.1166582209	0.9969272193
C	-0.7574029555	-0.2454556553	1.6441722961

C	-3.0873322929	-0.8165595733	1.5268154856
C	-0.6009714296	-1.0542938436	2.7688878206
H	0.1058879833	0.2862811778	1.2465306230
C	-2.9469779949	-1.6357747949	2.6432517888
H	-4.0706245039	-0.7261708147	1.0632845334
C	-1.6971272874	-1.7740222128	3.2668435355
H	0.3826383165	-1.1373149804	3.2276767157
H	-3.7953791656	-2.1878456635	3.0511112337
O	-1.6404368338	-2.6132350212	4.3309244980
C	-0.3883973255	-2.8620592278	4.9279914741
H	0.3343277209	-3.2706492631	4.1993737842
H	-0.5587967915	-3.6030958851	5.7200781332
H	0.0419103570	-1.9494482015	5.3786703741
C	-3.2548683746	-2.4062131640	-1.5735548095
C	-4.3639090842	-2.0147842246	-2.3407430181
C	-3.2734498553	-3.6398627634	-0.8997078113
C	-5.4791574585	-2.8485790763	-2.4310531669
H	-4.3441623742	-1.0679132960	-2.8821208625
C	-4.3969904679	-4.4587481793	-0.9736211399
H	-2.3958038696	-3.9260049644	-0.3181219089
C	-5.5003814597	-4.0642155640	-1.7402369788
H	-6.3352900528	-2.5492378771	-3.0396677230
H	-4.4156576443	-5.4098267942	-0.4367996391
H	-6.3802658325	-4.7092805635	-1.8013374333
C	-1.2600324969	0.5504852859	-2.6965520468
O	-1.2957345104	1.7337331997	-2.9583708535
O	-0.4853436939	-0.3312130384	-3.3362751720
C	0.3422393657	0.1572058475	-4.4036853055
H	0.6361911337	-0.7293253870	-4.9779533940
H	1.2392826928	0.6388191986	-3.9907357394
H	-0.2200439308	0.8624351954	-5.0306098828
N	1.5792763324	-1.3413775901	-1.0040960211
S	2.3552119961	-2.7948438040	-0.8389166567
O	2.9780435653	0.5010807708	-2.4663744285
O	3.7925843252	-2.5725825894	-0.9215955354
O	1.6608977817	-3.8152653490	-1.5998876319
P	2.3821449576	0.1279798121	-1.1666194257
O	1.1707803599	1.1025863590	-0.6646852611
O	3.3843864476	0.1320040004	0.1013294828
C	1.4129943137	2.4699362727	-0.5291839110
C	4.7485765244	0.4099360455	0.0737125422
C	2.0656802273	2.9319008768	0.6161911618
C	0.9735867985	3.3442245872	-1.5225382866
C	5.2414127696	1.5628918669	-0.5372335316
C	5.5889303435	-0.4871370260	0.7307638980
C	2.2812119659	4.3038936341	0.7685457576
C	1.1913698152	4.7154067409	-1.3549057678
H	0.4438566797	2.9497462621	-2.3890728818
C	6.6139684841	1.8185722525	-0.4787919219
C	6.9568556194	-0.2145100217	0.7868002867
C	1.8444310978	5.1989879084	-0.2147995614
H	2.7915226805	4.6734692566	1.6606307514
H	0.8464846735	5.4094890336	-2.1245886953
C	7.4727453234	0.9370341208	0.1835960752
H	7.0115444530	2.7160293676	-0.9582870807

H	7.6237459525	-0.9124687344	1.2981445503
H	2.0137914338	6.2709746518	-0.0930673202
H	8.5443272177	1.1441289473	0.2251518053
H	5.1583865964	-1.3857815217	1.1708419920
H	0.5421230223	-1.4502723597	-1.1750386968
C	-2.4871737611	3.8655358048	0.7155264901
C	-1.0026587525	3.3653925992	2.3294120327
C	-2.1857743263	2.7967693296	2.7438085776
C	-3.1561056350	3.1028817589	1.7201849800
H	-0.0265425637	3.3560206127	2.8087395317
N	-1.1788882423	4.0068269175	1.1158138707
C	-4.5150413469	2.7859917062	1.5438678076
H	-5.0439671095	2.1935945480	2.2945276956
C	-3.1435497145	4.3126091285	-0.4382349127
H	-2.6133733722	4.8799790221	-1.2059525387
C	-5.1715133720	3.2349370901	0.4014439810
C	-4.4920248050	3.9928015098	-0.5781413588
H	-6.2283193240	2.9987861858	0.2549413544
H	-5.0320595657	4.3278426332	-1.4670192886
H	-0.4529933773	4.4885699756	0.6010880466
C	1.9954672774	-3.1963145646	0.9586824759
F	2.6589579606	-4.2986552228	1.2881786645
F	2.3952795627	-2.1970980049	1.7464912408
F	0.6952214730	-3.3954490565	1.1329264872
H	4.5638117503	2.2370397899	-1.0605961197
H	2.3960091948	2.2169834354	1.3697016095
C	-2.4260121250	1.9957827430	3.9826386465
H	-3.2067081602	2.4516573549	4.6154729870
H	-2.7590738860	0.9748663577	3.7371438943
H	-1.5106517542	1.9100582194	4.5865548774

IN1Ba

0 1

C	-0.5056572759	2.0498293372	-1.1893383723
C	-2.7366954355	-3.2541128884	0.3469047747
C	-3.1316748093	-4.0611411985	1.4647741291
C	-2.1638842746	-4.4788462846	2.3343063051
C	-0.7789078838	-4.1293154370	2.1320795232
C	-0.3424120580	-3.3811651440	1.0691559792
C	-1.3333386741	-2.9127232664	0.1638927574
C	-3.4423716034	-2.6471389507	-0.6666163363
C	-2.4829643640	-1.8353956441	-1.4854267699
N	-1.1896465073	-2.1270590744	-0.8933004666
H	-2.4866912796	-2.1675007419	-2.5404713294
H	-4.1819889610	-4.3161968863	1.6155666656
H	-2.4290958823	-5.0814981487	3.2050167128
H	-0.0428634479	-4.4655466357	2.8660620302
H	0.7031004189	-3.1124612586	0.9454759219
H	-0.2385944781	-1.8491937097	-1.2243388265
O	0.1994193945	0.9259817856	-1.0165308290
C	-1.6599157680	1.8930240265	-1.9136105901
C	-2.8900562528	-0.3192703716	-1.4837462797
C	-1.9631944447	0.5106302629	-2.4353150985

H	-1.0196538215	-0.0194143893	-2.6051439055
H	-2.4833544609	0.5976736793	-3.3994347016
C	-3.0760309217	0.2342503490	-0.0835860942
C	-4.2973289863	0.8346337226	0.2428681997
C	-2.0766551367	0.2151696881	0.9084803249
C	-4.5368075611	1.3905010956	1.5047716412
H	-5.0736148626	0.9120729254	-0.5224075565
C	-2.2964252019	0.7567932222	2.1675827647
H	-1.0902955800	-0.1868770342	0.6971825148
C	-3.5314722251	1.3480107807	2.4804026562
H	-5.5015544454	1.8554085338	1.7059267518
H	-1.4984180153	0.7418210187	2.9102417298
O	-3.6571111238	1.8541307736	3.7327044943
C	-4.8539983353	2.5020306210	4.0881282906
H	-4.7323195188	2.8429074500	5.1249373747
H	-5.0543340830	3.3778773479	3.4442756943
H	-5.7235418224	1.8211958289	4.0354345429
O	1.4713127959	-2.0490483201	-1.2835062556
N	2.0700881657	-0.1132238630	0.4951768154
S	1.9286825936	-0.4312530409	2.0447147059
O	0.8250587886	0.3500517625	2.6197783288
O	2.0525216822	-1.8529158374	2.3949915733
H	0.9526834608	0.9634701431	-0.3749695767
H	-3.8866499637	-0.3098959169	-1.9455966365
P	2.5007987926	-1.1306267446	-0.6917431865
O	3.8086463093	-1.9185152585	-0.1299129173
O	3.0737726764	-0.1594325495	-1.8628645896
C	4.6072441098	-2.8164504931	-0.8083313950
C	3.6369771560	1.0832006454	-1.6027593957
C	4.1835368753	-3.5132352617	-1.9440658205
C	5.8900816455	-3.0029844090	-0.2854105840
C	4.7287129454	1.2193804633	-0.7434148874
C	3.0543907706	2.1949386524	-2.2110852274
C	5.0726280696	-4.3984496853	-2.5607206658
C	6.7626712710	-3.8946184769	-0.9098391498
H	6.1751439993	-2.4460487880	0.6087896356
C	5.2307117757	2.4959363050	-0.4826015960
C	3.5710089081	3.4651583286	-1.9492785003
C	6.3594173320	-4.5936068141	-2.0524481548
H	4.7479177567	-4.9435269825	-3.4504884273
H	7.7654445463	-4.0412628771	-0.5013334081
C	4.6541752893	3.6202991473	-1.0802278135
H	6.0727010329	2.6092943194	0.2041299434
H	3.1052251467	4.3391243578	-2.4072627021
H	7.0449025246	-5.2883936639	-2.5425691263
H	5.0429141581	4.6175387500	-0.8633418159
H	2.1917845976	2.0493356514	-2.8618433960
C	-2.7829765484	2.8271811735	-2.0158528239
O	-3.8471158807	2.5223569275	-2.5299685344
O	-2.5928818569	4.0362203091	-1.4499462944
C	-3.7177664865	4.9028521921	-1.4221776222
H	-4.0995212762	5.0937045539	-2.4366361194
H	-4.5323267136	4.4656211255	-0.8236806093
H	-3.3701707254	5.8363286421	-0.9616485537
C	0.0955873337	3.2613184360	-0.5844948551

C	0.1942075849	4.4682101120	-1.2947937881
C	0.7056266280	3.1583926094	0.6782642117
C	0.8816232600	5.5513400888	-0.7503903629
H	-0.2567586681	4.5446280997	-2.2834370705
C	1.3948380679	4.2445143456	1.2180857792
H	0.6488315822	2.2323245960	1.2531100824
C	1.4853908906	5.4428950477	0.5070738120
H	0.9605318294	6.4822717981	-1.3173986207
H	1.8711353696	4.1406041870	2.1950988503
H	2.0335765102	6.2901453945	0.9264280428
H	5.1586548766	0.3383622585	-0.2680615194
H	3.1717805469	-3.3675519767	-2.3224892585
C	3.4581180500	0.3798470218	2.7543551314
F	3.4696335635	1.6792137534	2.4552984773
F	3.4668667149	0.2413776058	4.0774164307
F	4.5608237452	-0.1828230736	2.2555053259
C	-4.9078251738	-2.6430357469	-0.8899618387
H	-5.3278437418	-1.6987476514	-0.4989156369
H	-5.1570213369	-2.6888379315	-1.9610877144
H	-5.4024616682	-3.4742686566	-0.3696035487

IN1Bb

0 1

C	-0.6364302814	1.7422848146	-1.7523350520
C	2.9542209946	0.5026014289	2.5166470368
C	3.9111630397	0.7259325592	3.5573845854
C	4.0597083492	-0.2309576913	4.5245975222
C	3.2687705512	-1.4351879676	4.5080907975
C	2.3313784942	-1.6954838901	3.5412505777
C	2.1592627928	-0.7130680889	2.5239134369
C	2.5674785483	1.2460767971	1.4148489664
C	1.5214520368	0.4704554765	0.6835368336
N	1.3285289171	-0.7104114426	1.5015467565
H	0.5762573940	1.0527457542	0.6833363387
H	4.5102783117	1.6382230105	3.5674096402
H	4.7856421246	-0.0937490505	5.3282044210
H	3.4275292397	-2.1667349305	5.3044513428
H	1.7305127442	-2.6054008472	3.5333285796
H	0.7024355536	-1.5069675894	1.2184330447
O	-0.8667861431	0.4364157670	-1.9629492513
C	0.6705312402	2.1540801006	-1.7731915748
C	1.8018391869	0.0094604686	-0.7893054415
C	1.7949243469	1.1389144578	-1.8364704971
H	0.9436050561	-0.6330296900	-1.0090283609
H	2.7406030623	1.6920817564	-1.8159393067
H	1.7738502658	0.6224842731	-2.8122570471
C	3.0420605792	-0.8594641425	-0.8525933119
C	2.9133638732	-2.2596672661	-0.8457285817
C	4.3326572823	-0.3211769113	-0.8695746727
C	4.0311263521	-3.0849947098	-0.8648253738
H	1.9190734175	-2.7079881614	-0.8143258658
C	5.4691265260	-1.1369032626	-0.8880261795
H	4.4738137300	0.7618720726	-0.8733696418

C	5.3213185296	-2.5315513614	-0.8874009645
H	3.9284385329	-4.1713012252	-0.8680271419
H	6.4557187311	-0.6748503524	-0.9058050726
O	6.3546016139	-3.4124401788	-0.9076194442
C	7.6713958797	-2.9166267851	-0.9365299490
H	7.9019168743	-2.3074368106	-0.0434018065
H	8.3369090959	-3.7900612926	-0.9523126565
H	7.8616231177	-2.3053457274	-1.8374845316
H	-1.5474675495	0.0675689799	-1.3250361083
N	-2.0779542960	-0.7901019804	0.0816560816
S	-2.2073724608	0.0274307615	1.4564908319
O	-0.2131063631	-2.6809424549	0.6245154320
O	-2.1867731977	-0.8203302433	2.6539494910
O	-1.3556798119	1.2299068540	1.4304786483
P	-1.5569216110	-2.3373461988	0.0506726567
O	-1.6199052852	-2.6553164261	-1.5498597517
O	-2.6666464237	-3.3298101413	0.6865061495
C	-0.5365355952	-3.1797998684	-2.2438655043
C	-4.0283367497	-3.0795044753	0.7380669851
C	-0.0899440598	-4.4762327875	-1.9812383650
C	0.0848766752	-2.3798536459	-3.2036312226
C	-4.6649203736	-3.2735233707	1.9635961728
C	-4.7388926681	-2.6682292927	-0.3916675260
C	1.0048907348	-4.9736694188	-2.6921699338
C	1.1751031748	-2.8921510349	-3.9114615243
H	-0.2792039959	-1.3643488067	-3.3571088456
C	-6.0409839625	-3.0620439190	2.0563666733
C	-6.1138337629	-2.4470917058	-0.2810676570
C	1.6402159332	-4.1851213055	-3.6565293821
H	1.3649591013	-5.9844348694	-2.4862438966
H	1.6731964140	-2.2676391952	-4.6566285450
C	-6.7686866384	-2.6459335124	0.9372424807
H	-6.5447786640	-3.2088224859	3.0146159211
H	-6.6751770194	-2.1175430147	-1.1585318602
H	2.5016754141	-4.5755833494	-4.2022829022
H	-7.8439484855	-2.4711443656	1.0163070453
C	1.0965973109	3.5626411685	-1.8614128884
O	2.2382638522	3.9458877011	-1.6590384958
O	0.1301160902	4.4173824296	-2.2421731567
C	0.4765558289	5.7951008402	-2.2827720005
H	0.8247378262	6.1441499271	-1.2981728343
H	1.2770321327	5.9791744029	-3.0154174427
H	-0.4373514689	6.3283676631	-2.5721225518
C	-1.8524376561	2.5632854631	-1.5426858105
C	-2.9463788458	2.3713962608	-2.4003467523
C	-1.9664578136	3.4730151224	-0.4810020986
C	-4.1202409438	3.1050183898	-2.2241010138
H	-2.8656782834	1.6468875570	-3.2128943768
C	-3.1451172753	4.1901257711	-0.2942798418
H	-1.1376328222	3.5852197826	0.2168275832
C	-4.2223358008	4.0150828596	-1.1701498427
H	-4.9615101852	2.9563064983	-2.9050895495
H	-3.2321735201	4.8784131651	0.5494771771
H	-5.1466956193	4.5781397597	-1.0196898663
H	-4.2171344148	-2.5226542739	-1.3382208968

H	-4.0650406381	-3.5665792018	2.8256777600
H	-0.5908303175	-5.0735101168	-1.2181126987
C	-3.9542701537	0.7241411724	1.3411706122
F	-4.8239177674	-0.1412270996	1.8485619615
F	-4.0094251676	1.8541280503	2.0423378971
F	-4.2825593433	0.9785602972	0.0793129623
C	3.0006591424	2.6190133993	1.0708669340
H	3.8847768489	2.9230721158	1.6471647371
H	3.1840119080	2.7638329108	-0.0026130955
H	2.1818236721	3.3217244461	1.3076041098

IN2Ba

0 1

C	1.4261474123	1.7560195395	2.9902654652
C	1.5165609917	1.5969814809	4.3846938397
C	0.3630066168	1.7286317810	5.1527746657
C	-0.8816527666	2.0227070860	4.5558689249
C	-1.0020237623	2.1933447325	3.1795914689
C	0.1582120046	2.0477027399	2.4106930969
C	2.3753849003	1.6674697033	1.9055214557
C	1.6629342507	1.8921274762	0.7361181958
N	0.3357430282	2.1242170421	1.0493235249
H	2.4746443945	1.3638833697	4.8565487379
H	0.4170004536	1.5961017169	6.2361310993
H	-1.7718140059	2.1060569677	5.1837195437
H	-1.9615876801	2.4024239131	2.7063878137
H	-0.4248548851	2.3110211470	0.4014036909
C	2.1110813756	1.7511148968	-0.6938636778
C	1.7446506895	0.3509795866	-1.2543041831
H	3.2064221524	1.8168240435	-0.6963567030
H	2.1520430443	0.2569442718	-2.2715951310
H	0.6536970142	0.2684032336	-1.3311975162
C	1.5867819017	2.8625700396	-1.5899578478
C	2.3999417629	3.9740604849	-1.8694557567
C	0.2932518909	2.8531626673	-2.1244544978
C	1.9430726882	5.0314591527	-2.6466197242
H	3.4137373007	4.0090000260	-1.4617915501
C	-0.1860110052	3.9119460360	-2.9043507278
H	-0.3851846905	2.0209524658	-1.9340015144
C	0.6397513255	5.0116673488	-3.1707949379
H	2.5762264213	5.8938035104	-2.8633146281
H	-1.2049293543	3.8640439974	-3.2869769890
O	0.2737628893	6.0869104576	-3.9143722375
C	-1.0311355877	6.1352086861	-4.4406921580
H	-1.2241366984	5.3009021487	-5.1396487490
H	-1.1151403389	7.0838584314	-4.9875400069
H	-1.7965935530	6.1109294045	-3.6441175995
N	-1.5273254385	-0.7510238540	0.9020878691
S	-1.9302081437	-1.0665921204	2.4819343078
O	-2.2953088987	1.7177547457	0.0099827366
O	-3.0411196040	-0.2077736403	2.8684919350
O	-0.7162007278	-1.2426488300	3.2537958211
C	-2.6497774557	-2.7924602182	2.3093341577

P	-2.3716984281	0.2505320930	-0.1555679968
O	-3.8546447503	-0.3763582542	-0.1066579512
O	-1.7353627691	-0.1825681672	-1.5784971029
C	-5.0189219461	0.3964537835	-0.1714693957
C	-1.6492446026	-1.4157513683	-2.2123881937
C	-5.6444363317	0.5645837119	-1.4043584167
C	-5.5352513454	0.9400916004	1.0022488710
C	-2.3461309760	-2.5485653032	-1.7894605247
C	-0.7995015876	-1.4578286951	-3.3201203636
C	-6.8316096708	1.2998014291	-1.4605056147
C	-6.7215472168	1.6741807187	0.9301816758
H	-5.0032552782	0.7784061757	1.9402319860
C	-2.1780867116	-3.7404462200	-2.5009348188
C	-0.6442955521	-2.6550709110	-4.0182327070
C	-7.3701635584	1.8549992239	-0.2958531309
H	-7.3352823785	1.4392325497	-2.4197795186
H	-7.1397105808	2.1089435196	1.8408401580
C	-1.3328195327	-3.8007929749	-3.6108827485
H	-2.7182849941	-4.6317087138	-2.1735991893
H	0.0310602037	-2.6945319586	-4.8752566930
H	-8.2971630438	2.4305931720	-0.3438733349
H	-1.2028758321	-4.7395340231	-4.1523305533
C	3.7457903871	-0.9593765399	-0.3594225686
O	4.5302972792	-0.3241478383	-1.0225059207
O	4.1082632725	-1.9155177257	0.5068213186
C	5.5082518560	-2.1804998362	0.6151969918
H	5.9220835441	-2.4719278720	-0.3613812162
H	6.0408575324	-1.2873068397	0.9734220736
H	5.6076318339	-3.0002513368	1.3360084607
H	-0.2710326817	-0.5505588300	-3.6157557052
H	-0.6550761495	-1.2453324895	0.5937416356
C	2.2374025447	-0.7990574482	-0.3638149888
H	1.9599070154	-0.5598076288	0.6756948833
C	1.4905936184	-2.0970596906	-0.6492565854
O	0.4540306188	-2.3253013786	-0.0347413356
C	1.9509393148	-3.0591835912	-1.6810017552
C	2.8737262527	-2.7114121338	-2.6826876986
C	1.3790619743	-4.3431413022	-1.6815267053
C	3.2096983279	-3.6357950518	-3.6725447833
H	3.3225282230	-1.7188758482	-2.7078320785
C	1.7343270420	-5.2696406183	-2.6567877240
H	0.6487467348	-4.5846850850	-0.9087739144
C	2.6457602058	-4.9151491865	-3.6581221015
H	3.9172089326	-3.3564855912	-4.4562233058
H	1.2929528815	-6.2686955467	-2.6470474535
H	2.9182342794	-5.6392278990	-4.4298616700
H	-5.2021971953	0.1201038101	-2.2978340297
H	-3.0101862422	-2.5092044640	-0.9281632102
F	-1.7620005418	-3.5879445017	1.7202313872
F	-3.7585640703	-2.7623411563	1.5743624488
F	-2.9345696200	-3.2600237806	3.5164008121
C	3.8327008439	1.3549083200	2.0530587201
H	3.9975829564	0.3126990782	2.3807754884
H	4.3842649023	1.4954637805	1.1126220049
H	4.3021038572	2.0018929960	2.8129919645

IN2Bb

0 1

C	0.2635926728	-4.1739987056	0.7121398982
C	-0.0281827905	-5.4404109411	0.1805575102
C	-1.3418279409	-5.9024669629	0.2312235415
C	-2.3700563917	-5.1199076568	0.7944800613
C	-2.1095563918	-3.8564714320	1.3250701305
C	-0.7880179132	-3.4073903450	1.2763279840
C	1.4673572261	-3.3705001235	0.7811044937
C	1.1215311747	-2.1755128140	1.3664313947
N	-0.2550172388	-2.1730338390	1.6368351608
H	0.7575879376	-6.0460865526	-0.2771380558
H	-1.5854776691	-6.8830964637	-0.1844651559
H	-3.3925987087	-5.5040286844	0.8044927183
H	-2.8975425786	-3.2247533040	1.7378265351
H	-0.6930071162	-1.5656811164	2.3249617981
C	1.9365805496	-0.9161822848	1.5325404678
C	2.0712995486	-0.2076597319	0.1649721094
H	2.9434922078	-1.2216561573	1.8577336611
H	1.0854672294	0.0672039279	-0.2305498916
H	2.4839661162	-0.9069466201	-0.5742797841
C	1.3616713664	-0.0122711709	2.6103007260
C	1.8738130919	-0.0197723602	3.9176872748
C	0.2574402728	0.8061735426	2.3517670179
C	1.2923363128	0.7451929073	4.9228515185
H	2.7449381366	-0.6379420903	4.1487276327
C	-0.3528348177	1.5705125787	3.3488224278
H	-0.1583067439	0.8596234263	1.3480328558
C	0.1645062968	1.5397975593	4.6509032862
H	1.6887119706	0.7413629393	5.9397993552
H	-1.2302118383	2.1605936661	3.0891084675
O	-0.3483726552	2.2357607412	5.6946367080
C	-1.5015957539	3.0185259679	5.4835995519
H	-1.3229506669	3.8209041058	4.7452175781
H	-1.7536250639	3.4731035576	6.4507749672
H	-2.3538458160	2.4058518198	5.1393020141
N	-1.6144962532	-0.7543605313	-0.4459619047
S	-1.6808877153	-1.7018018233	-1.8379828165
O	-3.0056703079	-0.6298140215	1.8270921301
O	-2.9626583126	-2.3578630375	-2.0144787176
O	-0.4089098658	-2.4066170302	-1.8786375434
C	-1.5814854472	-0.3861406963	-3.1727158311
P	-2.8497180159	-0.0513294187	0.4738553438
O	-4.0532504223	-0.1311839527	-0.5873351099
O	-2.4918549504	1.5261781664	0.5971349986
C	-5.2912077835	0.4858875375	-0.4736003011
C	-2.2574511780	2.3823872934	-0.4808805690
C	-5.8893020055	0.8945711280	-1.6654378662
C	-5.9048166981	0.6869176716	0.7636663052
C	-3.2979540424	3.1904650281	-0.9390942172
C	-0.9869188798	2.4410535824	-1.0489580735
C	-7.1318783861	1.5273041880	-1.6147112377

C	-7.1456113665	1.3311893484	0.7968274849
H	-5.4188833123	0.3389217859	1.6766711627
C	-3.0558231267	4.0620396757	-2.0035417447
C	-0.7569379493	3.3115135570	-2.1161559137
C	-7.7608846756	1.7524469240	-0.3850085186
H	-7.6088925366	1.8508068450	-2.5426646405
H	-7.6349872079	1.4968869085	1.7593001553
C	-1.7896498513	4.1216301728	-2.5954721925
H	-3.8655485561	4.6957298068	-2.3725362960
H	0.2357364597	3.3309176149	-2.5689168383
H	-8.7313915190	2.2517672148	-0.3492924127
H	-1.6096074941	4.8004889451	-3.4320071422
C	4.3868750844	0.6434664612	0.5322562190
O	4.9511656569	0.7949424715	1.5879897631
O	4.9477247932	0.0448573540	-0.5316786037
C	6.2931830150	-0.4077702473	-0.3724044935
H	6.3554461421	-1.1620097440	0.4260466236
H	6.5834959848	-0.8447104814	-1.3349696187
H	6.9546785457	0.4323676024	-0.1144766461
H	-0.1904502313	1.7950769674	-0.6881256137
H	-0.9049871851	-1.1799138631	0.1875485533
C	2.9536846888	1.0455049148	0.2280835850
H	2.6320993838	1.6773723891	1.0701253383
C	2.8459870575	1.8855354195	-1.0505758998
O	2.0661701229	1.5748668415	-1.9307529669
C	3.6879001745	3.1157056105	-1.1694565744
C	3.7596158564	3.7426733203	-2.4258285977
C	4.3791322128	3.6754852396	-0.0810909406
C	4.5131967504	4.9010530603	-2.5950167541
H	3.2160803517	3.2934838948	-3.2586814490
C	5.1277629032	4.8419721965	-0.2522742314
H	4.3394939485	3.2133620413	0.9056133938
C	5.1980125311	5.4541489242	-1.5062120085
H	4.5697608320	5.3777963312	-3.5763698242
H	5.6572748968	5.2741830546	0.5996460857
H	5.7874970758	6.3649180408	-1.6367575636
F	-0.4137863543	0.2306658529	-3.1119024796
F	-1.7047587474	-0.9953727227	-4.3454200818
F	-2.5656203393	0.4993782563	-3.0341193750
H	-4.2785977238	3.1263611866	-0.4672799183
H	-5.3640171934	0.7177524170	-2.6044858633
C	2.7981351242	-3.7497335158	0.2145557158
H	3.5793061319	-3.0201919520	0.4755634288
H	3.1249768087	-4.7367261532	0.5820297499
H	2.7559640261	-3.8119594048	-0.8867455151

IN3Ba

0 1

C	-2.3479131763	-1.2350189229	0.6742387551
C	0.8835378103	4.5313957280	0.4501774469
C	1.9699392229	5.1807930680	-0.1650834005
C	2.6105266521	4.5619608748	-1.2347107375
C	2.1797121327	3.3049872054	-1.7093419505

C	1.1003726987	2.6484434667	-1.1280322026
C	0.4692048089	3.2590245397	-0.0380727567
C	0.0184329131	4.8633563206	1.5561618896
C	-0.8500712288	3.8049230134	1.6933573790
N	-0.5836096317	2.8266066257	0.7448711052
H	-1.6485647958	3.6769334098	2.4201625466
H	2.3052411602	6.1568415061	0.1955999669
H	3.4633452801	5.0484895227	-1.7133123285
H	2.7054188069	2.8250421852	-2.5369386795
H	0.7665741372	1.6916181835	-1.5210449429
C	0.0761903014	6.1056547484	2.3872921810
H	1.0537682704	6.2121164383	2.8889470123
H	-0.7002491987	6.1046319091	3.1674228614
H	-0.0669716639	7.0120571359	1.7737591545
O	-1.2396337405	-1.6711984051	0.3910284808
C	-2.5061402637	-0.3135529376	1.8737446768
C	-1.2794806724	1.5619863566	0.5564183212
C	-1.4734286523	0.8431082927	1.9094539611
H	-0.4876565403	0.4928519462	2.2430944347
H	-1.8284707715	1.5469869738	2.6740051168
P	2.2620693687	-0.3563739035	1.0902137372
O	1.7964705614	0.8874240882	1.7343303320
O	2.2795362721	-1.5872435555	2.1276396968
O	3.7265202966	-0.3882844128	0.4371184220
N	1.2671118370	-0.9567202480	-0.1536792909
S	1.4260791760	-0.9742568993	-1.7903573445
O	0.0879258839	-0.8786158840	-2.3664618772
O	2.5254448742	-0.1286813019	-2.2251211905
C	-2.5563352557	1.6802456136	-0.2569352168
C	-3.7233249845	2.2623334603	0.2668880249
C	-2.6117202904	1.1277026233	-1.5389936657
C	-4.9127140787	2.2499392004	-0.4500627944
H	-3.7071517881	2.7192846581	1.2596207029
C	-3.7978018296	1.1158536955	-2.2790568641
H	-1.7221852437	0.6541136747	-1.9607432771
C	-4.9636486526	1.6553435887	-1.7247527347
H	-5.8287877687	2.6833423673	-0.0445588878
H	-3.8030781688	0.6499937472	-3.2631777914
O	-6.1788246501	1.6386681842	-2.3266893012
C	-6.2846727511	1.0964435466	-3.6242723779
H	-5.6587373609	1.6474607785	-4.3485498948
H	-5.9970595119	0.0309122240	-3.6448840795
H	-7.3384954713	1.1903894384	-3.9181355429
H	-0.5946852417	0.9449237944	-0.0330764935
H	0.3107189065	-1.2554201400	0.1457581469
H	-3.5052271612	0.1287315531	1.8830963728
C	1.9744561978	-2.7357283816	-2.1189214757
F	1.1629727241	-3.5843860394	-1.5005640962
F	1.9335123121	-2.9560570512	-3.4269964765
F	3.2172969259	-2.9051597313	-1.6820446069
C	2.2720368112	-2.9363766345	1.7766818642
C	1.0761761679	-3.6429326353	1.9057651595
C	3.4432855391	-3.5440344675	1.3275090185
C	1.0618096205	-4.9975607827	1.5658820389
H	0.1799628827	-3.1324815383	2.2630889403

C	3.4110121239	-4.8994445932	0.9898201565
H	4.3541094661	-2.9515201790	1.2351714109
C	2.2231558751	-5.6267451248	1.1059632379
H	0.1308254127	-5.5615657165	1.6578977200
H	4.3205919112	-5.3860604544	0.6302543326
H	2.2025267214	-6.6854761601	0.8378699228
C	4.5008042417	0.6858781731	-0.0182504771
C	5.3468176122	0.4111205281	-1.0910357241
C	4.4523163571	1.9435587763	0.5791705196
C	6.1763069186	1.4239245913	-1.5728236710
H	5.3223145290	-0.5809277928	-1.5419317670
C	5.2867806276	2.9474887390	0.0800590215
H	3.7543942702	2.1398993205	1.3917303677
C	6.1500966997	2.6946259227	-0.9877181094
H	6.8402775491	1.2197921986	-2.4160557784
H	5.2422367912	3.9413332464	0.5290739070
H	6.7961672872	3.4876373498	-1.3707824156
C	-3.5200166124	-1.6121842012	-0.1563281669
C	-4.8365922059	-1.2178944866	0.1331541692
C	-3.2649509858	-2.3579011422	-1.3236700261
C	-5.8795028637	-1.5633058046	-0.7271265782
H	-5.0598661056	-0.6272577578	1.0204607918
C	-4.3057733771	-2.7001890712	-2.1794513088
H	-2.2324116703	-2.6293238438	-1.5467683956
C	-5.6163939603	-2.3034445451	-1.8816460290
H	-6.8971519990	-1.2385917310	-0.5022119989
H	-4.0995173074	-3.2719922177	-3.0870162039
H	-6.4341115214	-2.5697928325	-2.5559829104
C	-2.3520385543	-1.1170012261	3.1599138276
O	-1.7125896117	-2.1302215669	3.2956327570
O	-3.0048613764	-0.5106832449	4.1623881086
C	-2.8652570433	-1.1040715940	5.4570946221
H	-1.8071412421	-1.1326250167	5.7560034429
H	-3.4418785971	-0.4735520773	6.1438910376
H	-3.2576753682	-2.1315582804	5.4555887392

IN3Bb

0 1

C	2.3215920779	-1.4518166567	-0.9750195872
C	-1.0694208068	4.3823568723	-0.9392633548
C	-2.2044823225	5.0077617653	-0.3922145688
C	-2.7906055449	4.4676236999	0.7491608636
C	-2.2558838986	3.3168321439	1.3642362277
C	-1.1220531561	2.6890510222	0.8560893564
C	-0.5447167715	3.2191021407	-0.3040299843
C	-0.2356250882	4.6493308518	-2.0854308733
C	0.7242700990	3.6622149461	-2.0984160449
N	0.5455491507	2.7906117228	-1.0335856867
H	1.5282894815	3.5153280777	-2.8145167709
H	-2.6224256780	5.9020701158	-0.8623250158
H	-3.6835390752	4.9322259828	1.1730492890
H	-2.7395267606	2.8978509157	2.2482496203
H	-0.6921785652	1.8272620542	1.3624505735

C	-0.4005424677	5.7689355250	-3.0638143786
H	-1.3832833575	5.7291142883	-3.5655306988
H	0.3733854931	5.7369351053	-3.8457782323
H	-0.3359322004	6.7540663713	-2.5697255535
O	1.3308195553	-1.3654762882	-0.2639546364
C	2.8346071678	-0.2617716371	-1.7716203770
C	1.3457678004	1.6252896522	-0.6685967088
C	1.8728152855	0.9333979134	-1.9391000058
H	0.9924708770	0.6133370137	-2.5143562328
H	2.4279830132	1.6545597933	-2.5553041734
P	-2.0213298425	-0.5282322316	-1.1740716573
O	-1.3932435258	0.5587168240	-1.9487484089
O	-1.9289002291	-1.9271555591	-1.9748264975
O	-3.5718210401	-0.4299285206	-0.7883487771
N	-1.2713024338	-0.8943032899	0.3098385436
S	-1.7226324834	-0.7109173092	1.8867534744
O	-0.5030205629	-0.4762881205	2.6510060301
O	-2.9125366578	0.1108720064	2.0147975092
C	2.3994208173	1.9675391151	0.3698674922
C	3.3742773162	2.9503055800	0.1329602613
C	2.3998469895	1.3170935123	1.6056960927
C	4.3269857185	3.2591075832	1.0956026806
H	3.3888073498	3.4833783330	-0.8198995530
C	3.3529726615	1.6142849271	2.5860204181
H	1.6410862427	0.5618853803	1.8216633512
C	4.3260666312	2.5892663242	2.3319981606
H	5.0882306708	4.0211134877	0.9191491333
H	3.3151415982	1.0874189458	3.5388674634
O	5.3048553670	2.9445717010	3.2044363506
C	5.3328209383	2.3400400884	4.4757346853
H	4.4054149520	2.5347180106	5.0436176209
H	5.4798530721	1.2463220844	4.4087774121
H	6.1816117554	2.7806880567	5.0155043655
H	0.6475410146	0.9304452259	-0.1993357688
H	-0.2561966882	-1.0848938260	0.2222278588
H	3.0108551214	-0.6293109269	-2.7953496546
C	-2.2595644079	-2.4463146680	2.3511522493
F	-1.3552070061	-3.3296203408	1.9405539714
F	-2.3750708008	-2.5142224279	3.6705628235
F	-3.4333589748	-2.7170810415	1.7917563604
C	-2.0601864904	-3.1960921463	-1.4275969068
C	-0.9053705952	-3.9616466488	-1.2644021306
C	-3.3168054494	-3.6927497154	-1.0854082883
C	-1.0118995831	-5.2503997033	-0.7406355713
H	0.0583286016	-3.5365638395	-1.5431553548
C	-3.4103491316	-4.9839163800	-0.5587958158
H	-4.1971095234	-3.0637402916	-1.2211772360
C	-2.2633760354	-5.7632324841	-0.3840219875
H	-0.1094973992	-5.8520485562	-0.6093565062
H	-4.3894922774	-5.3800173946	-0.2803148823
H	-2.3448889137	-6.7708385526	0.0293489969
C	-4.3627890622	0.7190657919	-0.6401206801
C	-5.4033165509	0.6221720413	0.2808652436
C	-4.1437194535	1.8722785828	-1.3903405365
C	-6.2600285685	1.7109153581	0.4457711536

H	-5.5084580581	-0.2914507383	0.8658539596
C	-5.0089523016	2.9541860657	-1.2073405353
H	-3.3005012958	1.9328161057	-2.0769174378
C	-6.0672429358	2.8784996925	-0.2998436673
H	-7.0772238223	1.6474374503	1.1680688024
H	-4.8353402602	3.8693632799	-1.7757557283
H	-6.7353053677	3.7318305409	-0.1641090443
C	3.0159354845	-2.7676985671	-1.0959160836
C	4.0704542759	-3.0009936382	-1.9950244224
C	2.5745224840	-3.8173948345	-0.2698667178
C	4.6634174066	-4.2613803269	-2.0719594723
H	4.4378987501	-2.2055276815	-2.6446209882
C	3.1685223296	-5.0745323335	-0.3471334776
H	1.7611555347	-3.6216549548	0.4299193684
C	4.2132272442	-5.2995243561	-1.2509142527
H	5.4795439995	-4.4345871842	-2.7766362681
H	2.8210953314	-5.8822581679	0.3011605731
H	4.6793857327	-6.2856409773	-1.3137877013
C	4.2253083233	0.1933455255	-1.3147844026
O	4.9159839184	0.9439061917	-1.9547359918
O	4.5812560441	-0.3564613815	-0.1501002862
C	5.8601822858	0.0128545260	0.3743821248
H	5.9462270604	1.1052481167	0.4300976940
H	5.9106280556	-0.4256240934	1.3772326383
H	6.6624593422	-0.3870946375	-0.2638132179

IN4Ba

0 1

C	-0.6503407794	-1.5886076594	1.6316621851
C	-1.0604213406	-1.6934489751	0.2741651344
C	-1.1559916970	-2.9276298715	-0.3821455119
C	-0.8441235956	-4.0678019210	0.3508179494
C	-0.4444605002	-3.9867636824	1.7022044679
C	-0.3422727074	-2.7601635641	2.3481101612
C	-0.6271004992	-0.1808421565	1.9623406232
C	-1.0716805731	0.4970910188	0.8314280572
H	-1.4699902212	-2.9956518016	-1.4228008651
H	-0.9073712167	-5.0447934466	-0.1323528054
H	-0.2074541713	-4.9041255692	2.2459678579
H	-0.0138854931	-2.7029567180	3.3860433882
N	-1.3191759616	-0.4169319768	-0.1899779055
C	-1.6788371660	-0.1260022725	-1.5729760881
H	-0.9430089411	-0.6617359343	-2.1970140983
C	-0.2199446728	0.3876968573	3.2906663589
H	0.0942891251	1.4353292156	3.2040501538
H	-1.0434617880	0.3535461501	4.0233943947
H	0.6153556351	-0.1898922344	3.7136961750
C	-1.4861366929	1.3723220569	-1.8688319748
H	-0.4206458962	1.5538591377	-2.0546802027
H	-2.0260090712	1.6326047682	-2.7889047331
C	-1.1454979418	1.9875275058	0.5782600389
C	-1.9424270140	2.2968449370	-0.7299902625
H	-1.6587498992	3.3218169036	-1.0021761154

C	-3.0566302472	-0.6646947722	-1.9546595724
C	-3.4530403854	-0.6206650768	-3.2945201819
C	-3.9415879499	-1.2352991478	-1.0287014732
C	-4.6963081161	-1.1040898355	-3.7136904272
H	-2.7797659526	-0.1975063023	-4.0455639148
C	-5.1802173999	-1.7252095918	-1.4263280768
H	-3.6594525947	-1.2871371369	0.0217670348
C	-5.5743968817	-1.6602515788	-2.7724748256
H	-4.9643518577	-1.0421989717	-4.7678162602
H	-5.8702561908	-2.1680369481	-0.7058194494
O	-6.8023123390	-2.1567774760	-3.0655828299
C	-7.2546815727	-2.1122208800	-4.3986206703
H	-6.6029194525	-2.6962654483	-5.0734960768
H	-8.2598710260	-2.5540885387	-4.4046348598
H	-7.3162202803	-1.0757487436	-4.7768678576
O	0.1822722901	2.4776656938	0.3813781880
H	1.3302558157	-0.3426752829	1.2596423128
N	2.3284610826	-0.4279859587	0.9841538699
P	2.7380727352	0.0632907959	-0.5845928417
S	3.3072353655	-1.1761348571	2.1067260312
O	1.5709009935	0.7437563968	-1.2069439954
O	4.1066945891	0.8795326887	-0.4608304957
O	3.3298499025	-1.2030577377	-1.3551109294
O	2.4637436278	-1.8597894358	3.0722677611
O	4.4217087732	-1.8127824651	1.4224492821
H	0.6266474696	1.9218610169	-0.2903616377
C	4.0333600619	0.2821730548	3.0504934044
F	3.0724962723	1.1629765487	3.3164138103
F	4.5394799462	-0.1776527976	4.1847169378
F	4.9866519687	0.8655278248	2.3415858228
C	2.5493538258	-2.3088655294	-1.7076489069
C	1.6741530005	-2.2136402884	-2.7894562229
C	2.7199291151	-3.4940444208	-0.9960293677
C	0.9695328964	-3.3536958310	-3.1837656267
H	1.5675815734	-1.2616412281	-3.3108593909
C	2.0069359508	-4.6241456493	-1.4031045202
H	3.4036599731	-3.5103664236	-0.1468404187
C	1.1409213007	-4.5593724152	-2.4980234404
H	0.2845136223	-3.2967115621	-4.0328127660
H	2.1282367068	-5.5601507554	-0.8541506666
H	0.5881173804	-5.4479540855	-2.8103037920
C	4.2395365904	2.2479551653	-0.2166036351
C	3.3022642547	2.9724752859	0.5191315206
C	5.3897179967	2.8474885767	-0.7275176937
C	3.5292526658	4.3347935177	0.7337364405
H	2.4002138838	2.5109291876	0.9190083352
C	5.6059595376	4.2070696645	-0.4958820859
H	6.0967876549	2.2397755671	-1.2939340781
C	4.6757518342	4.9555525014	0.2325600526
H	2.7925831239	4.9070034207	1.3017998768
H	6.5060373456	4.6829938294	-0.8916178237
H	4.8450778984	6.0199847718	0.4081206009
C	-1.7275982011	2.7924350936	1.7415455583
C	-1.3154691440	4.1221264549	1.9051334567
C	-2.6873046730	2.2711593658	2.6184711496

C	-1.8540900680	4.9148610449	2.9216451195
H	-0.5579557702	4.5254956937	1.2332462091
C	-3.2240707513	3.0616332042	3.6360443446
H	-3.0086740606	1.2370391203	2.5055081909
C	-2.8115981219	4.3880246031	3.7912220475
H	-1.5207706738	5.9494443392	3.0349417225
H	-3.9666301882	2.6357542129	4.3154371763
H	-3.2318524560	5.0057318401	4.5886065535
C	-3.4579282398	2.3380377953	-0.6466166016
O	-4.1342640596	3.0917426518	-1.3023634610
O	-3.9766168344	1.4284212051	0.1912365175
C	-5.3984781869	1.4136812475	0.3057773907
H	-5.8604339300	1.2016080595	-0.6682931614
H	-5.6378572417	0.6162240327	1.0188991737
H	-5.7608570983	2.3837679316	0.6760467602

IN4Bb

0 1

C	1.5093883230	2.0856209169	-0.0993728198
C	2.4518203671	1.6063501057	-1.0461146225
C	3.1144849309	2.4625446924	-1.9335353247
C	2.7974267823	3.8188158106	-1.8783705311
C	1.8596107671	4.3137164150	-0.9476853113
C	1.2180155373	3.4590359746	-0.0546470135
C	1.0455114842	0.9528345482	0.6663391320
C	1.7058575466	-0.1544314993	0.1723734274
H	3.8605470107	2.0887367926	-2.6363588641
H	3.2915797236	4.5118521434	-2.5634159587
H	1.6424985517	5.3846808436	-0.9258901737
H	0.5007547043	3.8454212223	0.6711826551
N	2.5693756660	0.2383700624	-0.8653615212
C	3.6774980140	-0.5393488949	-1.4066154278
H	3.6859506855	-0.3717778702	-2.4977406813
C	0.0405490485	1.0728073047	1.7687592809
H	-0.0046296709	0.1789180803	2.3971836274
H	0.3004778139	1.9206806841	2.4235013404
H	-0.9704298748	1.2888149383	1.3891584047
C	3.4328691090	-2.0440011271	-1.1912915359
H	2.7300835594	-2.4090595354	-1.9535873529
H	4.3764918048	-2.5865677952	-1.3340722419
C	1.5191936517	-1.6389075144	0.4291401227
C	2.8585002265	-2.3835269412	0.1897971417
H	2.6369306366	-3.4600932333	0.1985916354
C	5.0359216691	-0.0786188301	-0.8783548588
C	5.1772570597	0.7952008403	0.1996116720
C	6.2029229307	-0.5413037698	-1.5096213169
C	6.4394692921	1.1927256287	0.6546701745
H	4.2922173309	1.1745554525	0.7079169154
C	7.4641289211	-0.1592214132	-1.0711935332
H	6.1240334776	-1.2159166737	-2.3672748514
C	7.5942250105	0.7141018770	0.0220020021
H	6.5035183280	1.8784797996	1.4988574153
H	8.3711647600	-0.5197041206	-1.5593430212

O	8.8611449322	1.0387587575	0.3837651208
C	9.0547039995	1.9087488904	1.4736126306
H	8.6370489340	1.4943016955	2.4092312394
H	10.1397992827	2.0295546894	1.5912384072
H	8.6006703936	2.9001735631	1.2942827806
O	1.1301657579	-1.9377683218	1.7513918431
H	-1.5460405417	-1.2968195971	-0.4124075899
N	-2.3553816250	-0.7203536944	-0.1428765344
P	-2.6443224148	0.5576280736	-1.2535416765
S	-2.7930093376	-1.0096845977	1.4239236984
O	-1.4013205891	1.0991767286	-1.8278879222
O	-3.5987570922	-0.0854943925	-2.3759352704
O	-3.6530649009	1.5217503162	-0.4668521930
O	-1.7008526577	-1.7671683555	2.0349704526
O	-3.3811162110	0.1616846090	2.0451612975
H	0.1731371285	-1.8052388403	1.8532325719
C	-4.1804126960	-2.2630050435	1.2755988140
F	-3.8215697742	-3.2413212196	0.4524212419
F	-4.4128059770	-2.7615167023	2.4809447707
F	-5.2726589964	-1.6674895835	0.8227692287
C	-3.3675473737	2.7085031214	0.2192506727
C	-4.1868022008	2.9984750921	1.3087878163
C	-2.3530220966	3.5720969509	-0.1927334754
C	-3.9884461076	4.1929726212	2.0021530876
H	-4.9486325804	2.2782291854	1.6048316714
C	-2.1693685429	4.7639337950	0.5158173240
H	-1.7133853583	3.3117779547	-1.0356048279
C	-2.9807394097	5.0795516559	1.6079363141
H	-4.6227468112	4.4275402637	2.8599609565
H	-1.3796088865	5.4491255932	0.2002511243
H	-2.8279111611	6.0133917402	2.1531332335
C	-4.8009551093	-0.7526465814	-2.1603901486
C	-4.8221634431	-2.1371777322	-2.3171984864
C	-5.9550506296	-0.0359326066	-1.8424923264
C	-6.0279818084	-2.8208207513	-2.1476723533
H	-3.8949244262	-2.6568011877	-2.5545646799
C	-7.1540743796	-0.7319644980	-1.6744445741
H	-5.9018075415	1.0464953341	-1.7276577128
C	-7.1942165180	-2.1215402683	-1.8254477657
H	-6.0523195466	-3.9067059879	-2.2635595689
H	-8.0637305508	-0.1814663547	-1.4241044649
H	-8.1355481066	-2.6588605746	-1.6917311332
C	0.4946720481	-2.2351792054	-0.5656962509
C	0.1703189245	-1.6165596124	-1.7834992353
C	-0.1061413364	-3.4674227375	-0.2548336507
C	-0.7206007981	-2.2244318162	-2.6758825129
H	0.5789695638	-0.6366187099	-2.0230545089
C	-0.9952546873	-4.0726354024	-1.1445173069
H	0.1229891472	-3.9401837598	0.7009870391
C	-1.2953496577	-3.4587363803	-2.3651352525
H	-0.9763517481	-1.7125698595	-3.6056519938
H	-1.4582252204	-5.0264259588	-0.8815650687
H	-1.9809302553	-3.9389678885	-3.0673064219
C	3.9067486485	-2.2253818165	1.2859852800
O	4.7900817113	-3.0330403422	1.4466482744

O	3.7742575805	-1.1191489302	2.0215569351
C	4.7399593137	-0.9298787110	3.0519954632
H	4.6938250530	-1.7550496830	3.7780241330
H	5.7528028371	-0.8829680612	2.6271104186
H	4.4833266709	0.0201472197	3.5360490805

IN4Bc

0 1

C	2.3507109885	-2.4218370791	-1.6179079481
C	3.1497491039	-2.5881863284	-0.4522761811
C	4.0004259172	-3.6889893125	-0.2831072447
C	4.0208598834	-4.6449324462	-1.2955227317
C	3.2208754723	-4.5085752197	-2.4513609450
C	2.3887940153	-3.4065433784	-2.6212480646
C	1.6304828949	-1.1818508465	-1.4723439591
C	2.0183490106	-0.6435358096	-0.2604220580
H	4.6312637275	-3.7916355966	0.6013078203
H	4.6710030616	-5.5172158725	-1.1941352385
H	3.2612129728	-5.2815655322	-3.2223951172
H	1.7735832028	-3.3053666193	-3.5185222482
N	2.9154831501	-1.5089099120	0.3737967824
C	3.6058762586	-1.2698497341	1.6282947133
H	3.6146662182	-2.2217341464	2.1850454226
C	0.6269218677	-0.6585977854	-2.4507339589
H	0.0132010536	0.1416004175	-2.0221686840
H	1.1080692158	-0.2539544517	-3.3579499566
H	-0.0542294369	-1.4641333240	-2.7607446284
C	2.8033589260	-0.2531413063	2.4565801130
H	1.8649137958	-0.7088855447	2.8041714879
H	3.3949904251	0.0360332456	3.3364381559
C	1.4990404716	0.5808772462	0.4519228115
C	2.4888079349	0.9728537852	1.5939689687
H	3.4184402390	1.3215307858	1.1243746873
C	5.0497547699	-0.8305472725	1.4186453616
C	5.5035439911	-0.3295267566	0.1975329885
C	5.9598726700	-0.8984359455	2.4866119506
C	6.8218390069	0.1110895093	0.0342581261
H	4.8237787531	-0.2926885397	-0.6554787621
C	7.2720428776	-0.4669203595	2.3428358250
H	5.6344526468	-1.3007877121	3.4506314208
C	7.7156358777	0.0475060620	1.1115140967
H	7.1368612095	0.4928793860	-0.9361976585
H	7.9835468431	-0.5194470698	3.1687369767
O	9.0105044811	0.4448486815	1.0610896463
C	9.5157101170	0.9705971720	-0.1444939492
H	8.9728299512	1.8820555504	-0.4533755065
H	10.5669927319	1.2273135883	0.0410412547
H	9.4659742286	0.2334563656	-0.9661403651
O	0.2397713079	0.1844830312	1.0102067307
H	-1.1276860202	-0.2086379778	0.1437334015
N	-2.1601086780	-0.2460724923	-0.0786274659
P	-2.9715567989	1.0282159516	0.6946744496
S	-2.6476189433	-1.8101624913	-0.3697711517

O	-2.3186560976	1.4582243982	1.9545250198
O	-2.9806843953	2.2313690263	-0.3766137802
O	-4.5144158586	0.5966654292	0.7026848685
O	-1.4399738040	-2.5914339299	-0.5809076138
O	-3.7082428795	-2.2242031451	0.5359488214
H	-0.0090739869	0.7969465277	1.7335433056
C	-3.4778530874	-1.6638121564	-2.0452899790
F	-2.6211690424	-1.2215798953	-2.9573949539
F	-3.9076779865	-2.8695782356	-2.3933063875
F	-4.5134792335	-0.8322399785	-1.9709715026
C	-5.2851680470	0.0147579037	1.7104926068
C	-6.3511620858	-0.7738415000	1.2830047729
C	-5.0309102302	0.2403240196	3.0620388715
C	-7.1903101025	-1.3485613448	2.2373176347
H	-6.4919787806	-0.9379808197	0.2146283111
C	-5.8784918464	-0.3494324540	4.0053546791
H	-4.1849742273	0.8579265500	3.3624755271
C	-6.9568514654	-1.1394637388	3.6007157466
H	-8.0264792979	-1.9717744001	1.9122479872
H	-5.6889263129	-0.1833706057	5.0683805000
H	-7.6131829037	-1.5947254992	4.3454063593
C	-3.4142990576	2.1326859291	-1.6973458414
C	-2.4708310776	1.9307980115	-2.7029569601
C	-4.7721324888	2.2701214248	-1.9844109341
C	-2.9037659323	1.8547341849	-4.0287987855
H	-1.4183374020	1.8356712625	-2.4401977876
C	-5.1914634728	2.1928372174	-3.3139167347
H	-5.4789823613	2.4217811910	-1.1683056723
C	-4.2609016431	1.9830522916	-4.3367486866
H	-2.1721152481	1.6895814660	-4.8226671293
H	-6.2530450872	2.2947712822	-3.5504074541
H	-4.5947774465	1.9202170206	-5.3747624858
C	1.3397714938	1.7896534895	-0.4751961194
C	2.3016683151	2.0688895633	-1.4562029113
C	0.2698151743	2.6762098786	-0.3076679560
C	2.1823004741	3.1975876534	-2.2673808277
H	3.1393887197	1.3836630754	-1.5966929110
C	0.1472988230	3.8060207202	-1.1220262032
H	-0.4844688447	2.4929092338	0.4541815692
C	1.0998104787	4.0692248351	-2.1070912091
H	2.9356685138	3.3948241378	-3.0337545815
H	-0.7077668304	4.4716515058	-0.9859590066
H	1.0018268852	4.9485514832	-2.7478765507
C	2.0152433884	2.1578506963	2.4173252349
O	2.6736166157	3.1276748946	2.6806849329
O	0.7414611005	1.9895416341	2.8486690766
C	0.1143142343	3.0758137824	3.5428699297
H	0.3046552440	4.0192284861	3.0129725813
H	-0.9548634108	2.8377452074	3.5447185942
H	0.5133531207	3.1502573747	4.5649326662

IN4Bd

0 1

C	1.6025030148	2.3611317346	-0.3559855735
C	2.5452652600	1.8104388475	-1.2598008311
C	3.2230677384	2.5988203940	-2.1973400140
C	2.9108436660	3.9562246172	-2.2430671220
C	1.9627277928	4.5192897457	-1.3624868486
C	1.3110295374	3.7341380579	-0.4151614476
C	1.1449167427	1.2932384350	0.5017832759
C	1.8044371590	0.1481855659	0.0929598189
H	3.9746437760	2.1736007128	-2.8641888684
H	3.4167036877	4.5974629316	-2.9688185267
H	1.7487034595	5.5895050101	-1.4209982488
H	0.5886108620	4.1744901585	0.2737336670
N	2.6508708648	0.4572347486	-0.9869146829
C	3.8228803889	-0.3204108508	-1.3706891460
H	3.9431952240	-0.2208575870	-2.4617701525
C	0.1486578887	1.5214234686	1.5944746339
H	0.1049738094	0.6997488348	2.3133568614
H	0.4138133188	2.4321419235	2.1556642145
H	-0.8636274720	1.6997266857	1.2003481573
C	3.5840081196	-1.8027267833	-1.0637088685
H	2.8749137515	-2.2308299514	-1.7853701604
H	4.5315770873	-2.3429064063	-1.1936597024
C	1.6634623776	-1.3013558273	0.5393598893
C	3.0556544159	-2.0042389439	0.3549398856
H	3.7162673024	-1.5226419026	1.0901515747
C	5.0932466126	0.1985788634	-0.7094022182
C	5.0718292703	0.8542193916	0.5239292266
C	6.3358522537	-0.0142507261	-1.3264667872
C	6.2527358761	1.2805045079	1.1406332011
H	4.1185723990	1.0504141358	1.0184298016
C	7.5189918749	0.4024756687	-0.7278580722
H	6.3771975598	-0.5137239941	-2.2992293776
C	7.4874020322	1.0537197778	0.5170257210
H	6.1927189136	1.7909699995	2.1011370797
H	8.4874047179	0.2432899183	-1.2054941753
O	8.6871840483	1.4279578439	1.0282170654
C	8.7204292900	2.0880001042	2.2721363069
H	8.3014110618	1.4619560694	3.0805297199
H	9.7768303565	2.2927820644	2.4910615156
H	8.1679127511	3.0445691478	2.2443486172
O	1.3645649708	-1.3898117671	1.9118907177
H	-1.4083521574	-1.1578077761	-0.1922040329
N	-2.2137978501	-0.5484124071	0.0079652508
P	-2.5229986892	0.5656234227	-1.2613598910
S	-2.6107914296	-0.6126758821	1.6114159130
O	-1.2925528863	1.0212177792	-1.9298469918
O	-3.5010191856	-0.2236100901	-2.2644804653
O	-3.5147752843	1.6319935169	-0.5935566594
O	-1.5002358160	-1.2745048306	2.2960818734
O	-3.1890053843	0.6344790858	2.0748626435
H	0.4079633599	-1.2942544588	2.0496233933
C	-3.9998266419	-1.8714805446	1.6769032926
F	-3.6620052325	-2.9595265195	0.9945445637
F	-4.2030308950	-2.1908554753	2.9465379936
F	-5.1026919031	-1.3435647550	1.1691856237

C	-3.2210431242	2.9131872496	-0.1114994842
C	-3.9913729883	3.3562323433	0.9623033880
C	-2.2469559350	3.7169750942	-0.7030796768
C	-3.7848169238	4.6461580936	1.4527039672
H	-4.7216480595	2.6797100728	1.4045784268
C	-2.0546902391	5.0065416338	-0.1970847997
H	-1.6419565440	3.3374210627	-1.5258613780
C	-2.8179135847	5.4755384711	0.8744653952
H	-4.3806214946	5.0014550628	2.2964978276
H	-1.2949469831	5.6441335835	-0.6540425914
H	-2.6588528085	6.4844159946	1.2611732247
C	-4.6976624027	-0.8503486856	-1.9297507726
C	-4.7218770772	-2.2430939556	-1.8889090326
C	-5.8445000038	-0.0928962970	-1.6893969805
C	-5.9230380039	-2.8927516152	-1.5970089670
H	-3.7998473243	-2.7930417052	-2.0716348098
C	-7.0389385676	-0.7550180016	-1.3973015867
H	-5.7893254574	0.9947700737	-1.7292658241
C	-7.0819197329	-2.1518772643	-1.3499673200
H	-5.9494393917	-3.9840665464	-1.5578195426
H	-7.9427777870	-0.1721904233	-1.2060331124
H	-8.0196242278	-2.6623520349	-1.1201057290
C	0.6101517822	-2.0501285794	-0.3000576267
C	0.2879824258	-1.6625116491	-1.6098279882
C	-0.0120304277	-3.1892509352	0.2402018697
C	-0.6184356594	-2.4099621375	-2.3696811566
H	0.7106166354	-0.7488809584	-2.0245151752
C	-0.9155515456	-3.9343326760	-0.5185901683
H	0.2184347790	-3.4831755882	1.2645262685
C	-1.2103006258	-3.5552617270	-1.8322098955
H	-0.8732870742	-2.0770999560	-3.3777322049
H	-1.3945849839	-4.8124053822	-0.0797051725
H	-1.9079802604	-4.1458334362	-2.4307113997
C	2.9945819063	-3.4657996811	0.7620575282
O	3.0917598037	-3.8820416343	1.8875061153
O	2.8083122184	-4.2744111613	-0.3048117752
C	2.6617463632	-5.6618506642	-0.0231774919
H	1.7565583589	-5.8395327826	0.5778290833
H	2.5741346660	-6.1650770807	-0.9938338415
H	3.5294005327	-6.0438188680	0.5347727750

P5B

0 1

C	-0.3787853970	-2.5206057982	2.8651967227
C	-1.5309202526	-2.0394654343	2.1905929273
C	-2.5534423080	-1.3962122554	2.9016070427
C	-2.4045809455	-1.2544969707	4.2827061543
C	-1.2713972338	-1.7378359415	4.9596849599
C	-0.2534801487	-2.3744931139	4.2544786542
C	0.5253786450	-3.0708483303	1.8779318601
C	-0.0616730558	-2.8817886364	0.6498288683
H	-3.4377632322	-0.9997852935	2.4163494560
H	-3.1959019091	-0.7538758966	4.8460348148

H	-1.1897103706	-1.6087336314	6.0413029622
H	0.6367123021	-2.7439180958	4.7682745952
N	-1.3402050879	-2.3032001228	0.8208897534
C	-2.1958683840	-2.0107310923	-0.2419592794
C	-1.7157944780	-2.0288982052	-1.5203319275
C	0.4115723430	-3.1917042315	-0.7477629793
C	-0.2448757039	-2.2349712433	-1.7615463535
C	-3.6142972436	-1.7275803145	0.1111531388
C	-4.4385708712	-2.7728314098	0.5433196723
C	-4.1180329666	-0.4211575782	0.0735307259
C	-5.7544095498	-2.5162026514	0.9328275696
H	-4.0411944305	-3.7892902217	0.5818698914
C	-5.4297480576	-0.1644990318	0.4684699457
H	-3.4748430181	0.4015042868	-0.2369508126
C	-6.2519672177	-1.2109387811	0.8986886910
H	-6.3915479499	-3.3377060140	1.2685059992
H	-5.8062486708	0.8600556852	0.4457876759
H	2.6002319755	0.8335681574	1.2220627165
N	2.2644240842	1.5707105068	0.5388034652
P	0.6477414756	1.3980479370	0.1547675457
S	3.4044136447	2.6096848361	-0.0681715904
O	0.1107020552	0.1526618824	0.7591518215
O	-0.0629459877	2.7689752751	0.6223482529
O	0.6580790863	1.4671973999	-1.4549965033
O	4.6767901623	1.9057873646	-0.1051249154
O	2.8598772713	3.3488885288	-1.1958038236
O	2.5625444912	-0.5318259062	2.1843068796
H	3.0259390123	-1.2404851947	1.7115323467
H	1.6183898347	-0.7413450629	2.0438875529
C	3.5320302511	3.8372288889	1.3429103490
F	3.8403033047	3.1930110683	2.4628958536
F	4.4809946058	4.7187749845	1.0594170354
F	2.3723175296	4.4611386829	1.4958369877
C	-1.4244643958	2.8651150084	0.9362085357
C	-1.9774000119	2.0846170473	1.9523524418
C	-2.1906867105	3.7752455399	0.2100098821
C	-3.3355841879	2.2309870447	2.2440770049
H	-1.3651059399	1.3578450894	2.4849339815
C	-3.5459734257	3.9129462746	0.5173117599
H	-1.7235768830	4.3465893399	-0.5927328981
C	-4.1207939380	3.1434758114	1.5340452735
H	-3.7786668858	1.6144848047	3.0284895696
H	-4.1551525222	4.6210593422	-0.0487411786
H	-5.1816819823	3.2520419614	1.7698854910
C	-0.3978662757	2.0064267755	-2.1902732357
C	-0.1784364878	3.1865269158	-2.8968259576
C	-1.6307851748	1.3607850010	-2.1971807924
C	-1.2383398735	3.7263071320	-3.6323024786
H	0.8036130906	3.6584662810	-2.8448880521
C	-2.6833139891	1.9100531324	-2.9314945349
H	-1.7547062330	0.4474300554	-1.6195077426
C	-2.4876012731	3.0941193498	-3.6497890656
H	-1.0862589251	4.6515970926	-4.1929038660
H	-3.6500790756	1.4018918262	-2.9334734381
H	-3.3091841757	3.5266541573	-4.2250830454

C	1.9216183950	-3.1232432839	-0.8893771318
C	2.5882464807	-1.9003701952	-0.6945616740
C	2.6915629326	-4.2341856586	-1.2302163451
C	3.9689179151	-1.7935608240	-0.7988900614
H	2.0036033412	-1.0195900909	-0.4422909684
C	4.0867747265	-4.1527538242	-1.3440152389
H	2.2028495740	-5.1980292726	-1.3972410758
C	4.7355258977	-2.9300860969	-1.1220746096
H	4.4747016507	-0.8356526645	-0.6541790309
H	4.6512342394	-5.0471018093	-1.6059564490
O	6.0747113835	-2.7452249325	-1.2021656179
C	6.8976739309	-3.8395951541	-1.5322625481
H	7.9295116904	-3.4647046152	-1.5415512706
H	6.8176497990	-4.6523843447	-0.7877229515
H	6.6553325289	-4.2486932403	-2.5297260724
H	-7.2801671095	-1.0085783486	1.2081508398
H	0.2601834912	-1.2567414658	-1.7133703016
H	0.0883936979	-4.2191980333	-0.9972301773
H	-0.0699634378	-2.6159418140	-2.7732516019
C	-2.5962910034	-1.7444547754	-2.6737283156
O	-3.7963654380	-1.5661861733	-2.6653813232
O	-1.8735291279	-1.6582206291	-3.8186353736
C	-2.5975066513	-1.3138284310	-4.9929123303
H	-3.4450075142	-1.9972695397	-5.1501247075
H	-2.9862692973	-0.2863221724	-4.9205415652
H	-1.8846261518	-1.3855384661	-5.8237895884
C	1.8098053690	-3.7475177385	2.2481422885
H	2.4227743947	-4.0061379098	1.3785229818
H	2.4082518982	-3.1087439283	2.9185076422
H	1.6056300663	-4.6785615757	2.8046432206

P7B

0 1

C	3.5830292079	1.4081272131	2.5578051333
C	4.0443260048	0.0883000854	2.2780945804
C	4.4318096841	-0.7952476561	3.2961349139
C	4.3595464665	-0.3333318258	4.6065324411
C	3.9121521909	0.9744986906	4.9061130456
C	3.5249000483	1.8457428511	3.8956412209
C	3.2535767931	2.0298916162	1.3068995592
C	3.4970614543	1.0750656019	0.3137955761
H	4.7606063610	-1.8115185955	3.0730491651
H	4.6497313346	-0.9981494825	5.4236686627
H	3.8674832130	1.2969402455	5.9488207484
H	3.1723418042	2.8520435432	4.1333205386
N	4.0131762767	-0.0826848489	0.9193713530
C	4.3947337362	-1.2768503442	0.1906758948
H	5.2596250992	-1.7162561146	0.7147704975
C	4.8628324045	-0.8360769050	-1.2070845272
H	5.8094246283	-0.2774058742	-1.0998278858
H	5.0745539839	-1.7141505403	-1.8261957747
C	3.2502598402	1.0503171435	-1.1147529972
C	3.8177913749	0.0424513016	-1.8549829850

C	3.2883423573	-2.3198658432	0.1213872256
C	1.9968382824	-2.0849540565	0.5992401806
C	3.5642102172	-3.5651212434	-0.4708195200
C	0.9888966376	-3.0518052634	0.4602800304
H	1.7429966094	-1.1358399603	1.0765377769
C	2.5780601106	-4.5303771020	-0.6119909398
H	4.5729280973	-3.7821061959	-0.8336743441
C	1.2705437688	-4.2779289488	-0.1578435223
H	-0.0065545617	-2.8263034085	0.8350637555
H	2.7857450611	-5.4945573307	-1.0794281898
O	0.3719525157	-5.2664826518	-0.3617534479
C	-0.9805762889	-5.0406030635	-0.0212806216
H	-1.4015806194	-4.1849439280	-0.5740666141
H	-1.5297282684	-5.9480028052	-0.3038964755
H	-1.1042343647	-4.8703236238	1.0612782969
H	-0.9918661548	-0.9272794670	-0.8933506434
N	-1.6903301351	-0.7662467277	-0.1165052480
P	-1.3096183756	0.5012307827	0.9203121800
S	-3.1705390368	-1.4954456888	-0.3125746721
O	0.0908281213	0.5415931728	1.3917307440
O	-1.7118654566	1.8968278455	0.2056658104
O	-2.4656143429	0.2271511292	2.0177314202
O	-4.2783239191	-0.5556734308	-0.2320502510
O	-3.0177421259	-2.4280298814	-1.4210622488
O	0.3939433944	-0.7453254634	-1.7574827756
H	1.0897604377	-1.1602823348	-1.2237631458
H	0.8654552860	-0.3847520276	-2.5389399393
C	-3.3351548467	-2.5622312924	1.2631337837
F	-4.2432877799	-2.0403460291	2.0692206213
F	-3.7307897514	-3.7736076847	0.8847748852
F	-2.1773972695	-2.6682295656	1.9091377816
C	-2.7249534849	2.0585660396	-0.7400146659
C	-2.5212434589	1.6041906294	-2.0428975264
C	-3.9001028446	2.7020905802	-0.3617361862
C	-3.5381254655	1.7817228523	-2.9827237158
H	-1.5789272133	1.1236245507	-2.3062443690
C	-4.9063369039	2.8780296478	-1.3139047096
H	-4.0193170000	3.0401565796	0.6678651125
C	-4.7312009030	2.4137826576	-2.6207679613
H	-3.3931708821	1.4226281150	-4.0039157889
H	-5.8364509946	3.3744140297	-1.0279726983
H	-5.5252331135	2.5460687015	-3.3588422021
C	-3.0149739581	1.1935530020	2.8514101137
C	-4.4053528878	1.2900819441	2.8820625851
C	-2.1984724025	2.0177617235	3.6256051857
C	-4.9925099400	2.2409988795	3.7195750200
H	-4.9924377520	0.6314120322	2.2414658220
C	-2.8020941883	2.9708449345	4.4507883323
H	-1.1137117867	1.9050324480	3.5760974605
C	-4.1947415531	3.0837271061	4.5015533389
H	-6.0809322888	2.3275875514	3.7553854031
H	-2.1758123578	3.6258409613	5.0606897769
H	-4.6592341190	3.8285432183	5.1513199566
C	2.4276420173	2.1185182400	-1.7335186051
C	1.1580550265	2.4341226617	-1.2321372096

C	2.9336753733	2.8460144120	-2.8231268962
C	0.4052933316	3.4534115739	-1.8144725237
H	0.7778752934	1.8814110173	-0.3767207833
C	2.1806253626	3.8660258980	-3.4045416416
H	3.9312226225	2.6142347033	-3.2031558005
C	0.9120570985	4.1707986439	-2.9020636286
H	-0.5796728165	3.6908242868	-1.4114949749
H	2.5872065656	4.4286218045	-4.2484082217
H	0.3208931496	4.9712100044	-3.3536319930
C	3.3856962574	-0.2511443003	-3.2317956353
O	2.2608486122	-0.0944494532	-3.6845244475
O	4.3669004836	-0.7989727895	-3.9718997928
C	4.0098579184	-1.2127862034	-5.2900665685
H	3.2005153049	-1.9575861536	-5.2584962023
H	3.6681534196	-0.3551226676	-5.8882197455
H	4.9157923868	-1.6485885315	-5.7282539762
C	2.7665180593	3.4399328964	1.1914258808
H	2.9799606932	3.8810651250	0.2109568574
H	1.6769373979	3.4993545391	1.3483674078
H	3.2451224346	4.0651030913	1.9617471623

TS1Ba

0 1

C	-1.8581247242	-2.1926020540	-1.1483625128
C	4.4752178996	-1.3075634514	-1.3468421642
C	5.5380782802	-1.6011977572	-0.4514772274
C	6.0618650944	-0.5776219369	0.3077916100
C	5.5591651862	0.7552995634	0.1980676302
C	4.5322423784	1.0862659769	-0.6616158861
C	3.9820691726	0.0421777660	-1.4371686876
C	3.6725629188	-2.1003499421	-2.1894919047
C	2.6352450033	-1.2430367234	-2.6726163518
N	2.9531236293	0.0743914878	-2.3139021894
H	2.0631307354	-1.4113213453	-3.5852927365
H	5.9162832866	-2.6212437508	-0.3586240060
H	6.8718707556	-0.7801541510	1.0114907826
H	5.9974213411	1.5375057655	0.8226724869
H	4.1375581334	2.1001128973	-0.7232140476
H	2.2391430723	0.8274314398	-2.3313137784
C	3.7538477920	-3.5721694014	-2.3816142365
H	3.2313233395	-3.9011680432	-3.2902263165
H	3.2926934338	-4.0930782555	-1.5220450863
H	4.8000369691	-3.9089630915	-2.4364657326
O	-1.6628921413	-1.0448930457	-1.8300771361
C	-0.9052507774	-3.1634638089	-1.2865916400
C	1.0975735483	-1.6512566322	-1.3999937548
C	0.3878493457	-2.8339765645	-2.0195890731
H	0.1600418242	-2.5903881412	-3.0691394827
H	1.0144404397	-3.7326792308	-2.0064180711
C	1.6095129335	-1.6815571027	-0.0499467016
C	1.9092855635	-0.4635768676	0.5918608726
C	1.8403046462	-2.8852406054	0.6649515438
C	2.4121099560	-0.4171243937	1.8845980671

H	1.7083012045	0.4688303296	0.0726047193
C	2.3353648751	-2.8494291451	1.9539107712
H	1.5752063790	-3.8459888589	0.2200272440
C	2.6284314461	-1.6150948663	2.5769342542
H	2.5895758909	0.5520579020	2.3440244775
H	2.4918157373	-3.7644299684	2.5277381749
O	3.0921724161	-1.6845018473	3.8394511399
C	3.3289501081	-0.4821104119	4.5442481663
H	3.6618768350	-0.7704726030	5.5495062822
H	4.1173201553	0.1207716600	4.0598655823
H	2.4112765122	0.1255521856	4.6220171914
P	-0.3139854335	2.1435424297	-1.1414448231
O	0.8242726289	1.6705009187	-1.9925226087
O	-1.4627494294	2.7596459368	-2.1048667288
O	0.1285706640	3.4226128341	-0.2268266533
N	-1.0768986851	1.0427855777	-0.2256005150
S	-1.0393721315	0.7574996300	1.3457283596
O	-1.0455149582	-0.6839536924	1.5969418424
O	-0.1084534625	1.6016722021	2.1068850613
H	-1.6761069855	-0.2599433654	-1.2069860730
H	0.6474361353	-0.6963905750	-1.6668592150
C	-2.7341751916	1.3382283898	1.8801305725
F	-3.6833249684	0.6886821670	1.2110641905
F	-2.8881510632	1.1061673946	3.1823253776
F	-2.8601540068	2.6449945212	1.6553579663
C	1.4511562146	3.7959300099	-0.0444207511
C	2.0254955173	3.6285847356	1.2175005843
C	2.1767374784	4.3527962520	-1.1011188278
C	3.3457740569	4.0396645281	1.4219465366
H	1.4223897367	3.1696383342	1.9999371146
C	3.4967515861	4.7559806892	-0.8835490047
H	1.7050280036	4.4585761529	-2.0786642908
C	4.0849337762	4.6035566558	0.3770220915
H	3.7991364515	3.9165856964	2.4086914896
H	4.0680803554	5.1927405296	-1.7060523147
H	5.1160799973	4.9239853706	0.5424197346
C	-2.7560834162	3.0022713800	-1.6569204028
C	-3.7201104729	2.0041050522	-1.7971802120
C	-3.0638729860	4.2338224905	-1.0803813529
C	-5.0184485477	2.2462820350	-1.3463536620
H	-3.4356394865	1.0475819747	-2.2357638828
C	-4.3686223188	4.4678724721	-0.6397079809
H	-2.2775055273	4.9817232983	-0.9703065047
C	-5.3458942847	3.4766946909	-0.7687748637
H	-5.7722074561	1.4608163196	-1.4336705477
H	-4.6185461095	5.4279387433	-0.1821767347
H	-6.3618784663	3.6608238370	-0.4125031225
C	-0.9980430015	-4.5425450562	-0.7787557892
O	-0.0325994415	-5.2721387470	-0.6184926207
O	-2.2511952998	-4.9643205391	-0.5402850704
C	-2.3882924785	-6.2549529810	0.0411125343
H	-1.8502010264	-6.3131060390	0.9998562890
H	-3.4635159683	-6.4025403027	0.2000377130
H	-1.9881551033	-7.0326662589	-0.6269617147
C	-3.0993030725	-2.2294460013	-0.3407883038

C	-3.1095293438	-2.6663721887	0.9925224889
C	-4.2812416786	-1.7224900507	-0.9034044215
C	-4.2838097033	-2.6126093435	1.7388736731
H	-2.1800997916	-3.0005068709	1.4509730309
C	-5.4606025516	-1.6819359546	-0.1586626265
H	-4.2699913498	-1.3690632739	-1.9359911311
C	-5.4636491482	-2.1252333816	1.1656280681
H	-4.2759188704	-2.9346554495	2.7828610405
H	-6.3774395683	-1.2965696270	-0.6114028429
H	-6.3815714124	-2.0798965278	1.7568585617

TS1Bb

0 1

C	-1.7478326599	-2.3035128400	1.1461417817
C	2.4907389479	-1.7164327232	-2.4582766552
C	3.6713365742	-2.2542426829	-3.0312235013
C	4.7063742493	-1.3965977159	-3.3381755166
C	4.5919555487	0.0057920755	-3.1009183268
C	3.4462318253	0.5728995285	-2.5756906053
C	2.3830825905	-0.2943372421	-2.2513612749
C	1.3067433098	-2.2971987646	-1.9547869240
C	0.5553208451	-1.2354287757	-1.3716651808
N	1.1778557384	-0.0300953075	-1.6959238110
H	-0.5183814994	-1.2531993790	-1.1830930175
H	3.7600840071	-3.3289532625	-3.2049409584
H	5.6306127697	-1.7857608225	-3.7706712976
H	5.4385121836	0.6508528467	-3.3483468219
H	3.3666587073	1.6447240168	-2.3909807461
H	0.8793024840	0.8738231909	-1.2802829220
C	0.9276153602	-3.7362451934	-1.9890893754
H	-0.1606234865	-3.8652723167	-1.9173489526
H	1.3751042972	-4.2983927655	-1.1527772075
H	1.2765600843	-4.2043996599	-2.9220133171
O	-1.4386596237	-1.1201858375	1.7016878432
C	-0.7008033391	-3.1731512460	0.9529256181
C	1.1934536389	-1.4023279827	0.6578049128
C	0.7190376674	-2.7084407051	1.2336242927
H	0.5394758871	-0.5640928851	0.8830866505
H	1.4160594447	-3.5168658914	0.9972452369
H	0.8353734051	-2.5428766106	2.3258504050
C	2.5950744243	-1.0521225288	0.6466267882
C	2.9677458026	0.3098206665	0.7817093074
C	3.6224304831	-1.9950054087	0.4290949205
C	4.2924986744	0.6982528626	0.7227062539
H	2.1909092721	1.0626070807	0.9142439767
C	4.9551517615	-1.6136203257	0.3470445348
H	3.3697328632	-3.0479245517	0.2963562387
C	5.2994955866	-0.2547599686	0.4826665432
H	4.5795634373	1.7447989066	0.8312998119
H	5.7158292742	-2.3682221995	0.1542087166
O	6.5576084771	0.2168449478	0.3928009033
C	7.6100801679	-0.6703153079	0.0756436702
H	7.4425221697	-1.1598803978	-0.8996743899

H	8.5233644211	-0.0640234860	0.0236180027
H	7.7371683611	-1.4463810323	0.8503046171
H	-1.9203936252	-0.3592701539	1.2513136884
N	-2.0647672832	0.9676851394	0.1865565783
P	-0.9706551020	2.1347205037	0.4984717816
S	-2.5849591883	0.7499084880	-1.3198844345
O	0.3891960847	2.0631773645	-0.1385971742
O	-1.7163469384	3.5526098109	0.2450192164
O	-0.8654983803	2.0579216029	2.1258700729
O	-2.0406358159	1.7231409544	-2.2774310831
O	-2.5533110806	-0.6763026172	-1.6698771734
C	-1.0451976724	4.6740842197	-0.2206941955
C	-0.7397958933	4.7757490096	-1.5785438918
C	-0.7179144038	5.6869526554	0.6810083447
C	-0.0902870372	5.9240949029	-2.0357126262
H	-1.0219262329	3.9559073349	-2.2408464743
C	-0.0683446345	6.8318005900	0.2090797690
H	-0.9847154490	5.5692928553	1.7329445723
C	0.2481869286	6.9512416495	-1.1472389638
H	0.1521505516	6.0175563133	-3.0970934889
H	0.1883497648	7.6333349766	0.9059311799
H	0.7549416086	7.8470385169	-1.5134443232
C	0.3422723905	1.9229759862	2.7892955709
C	0.5766803439	0.7433745007	3.4985594580
C	1.2951434283	2.9443988216	2.7514727007
C	1.7868832866	0.5867709835	4.1790201524
H	-0.1841050642	-0.0372561673	3.4822053211
C	2.5023105522	2.7729291324	3.4329890160
H	1.0943392742	3.8493567913	2.1761772043
C	2.7530776117	1.5962064341	4.1459224042
H	1.9781387040	-0.3369870103	4.7303076581
H	3.2537790279	3.5654147107	3.4012327912
H	3.7017702440	1.4654040008	4.6707566179
C	-4.3991087714	1.1619076866	-1.1630784774
F	-4.5398056188	2.4301330952	-0.7908263007
F	-4.9926663940	0.9824846185	-2.3404158579
F	-4.9750847828	0.3746128933	-0.2601687128
C	-0.9203037350	-4.6033794731	0.6744503023
O	-1.9536112820	-5.1536345514	0.3622993458
O	0.2312620399	-5.3287346674	0.8220476729
C	0.1085445395	-6.7259421885	0.5859429197
H	-0.2242393385	-6.9242637207	-0.4447283025
H	1.1050257762	-7.1541742103	0.7540738468
H	-0.6224484746	-7.1790061325	1.2722012083
C	-3.1929234126	-2.4965312562	0.8894311660
C	-4.0974763558	-2.0203432285	1.8551178415
C	-3.6926580713	-3.0431751087	-0.3018454970
C	-5.4730094407	-2.1204377613	1.6495129905
H	-3.7118455573	-1.5749399368	2.7738109465
C	-5.0659268140	-3.1211893437	-0.5147340049
H	-3.0019735108	-3.3768131239	-1.0717085181
C	-5.9598187357	-2.6694117290	0.4614230843
H	-6.1647817847	-1.7575648994	2.4131231317
H	-5.4429385991	-3.5305724790	-1.4545435974
H	-7.0370088105	-2.7353670898	0.2894063184

TS2Ba

0 1

C	-1.6586558505	-0.5858903372	0.8508378545
C	-3.4818631848	-0.3770462031	-2.4833199630
C	-4.0786892794	-1.4695263327	-3.1214130773
C	-3.3312067000	-2.1823427321	-4.0601845344
C	-2.0077333465	-1.8138254074	-4.3701815274
C	-1.3992484696	-0.7172998362	-3.7597702966
C	-2.1588577725	-0.0129466457	-2.8242596015
C	-3.9143280894	0.5337669060	-1.4101159749
C	-2.8362573051	1.4815483040	-1.2755760341
N	-1.8196842312	1.1057331183	-2.0659627697
H	-3.4744970143	0.0259517672	-0.2702518653
H	-5.1034602861	-1.7645369817	-2.8844349336
H	-3.7780051212	-3.0416832303	-4.5652231352
H	-1.4472749702	-2.3949905644	-5.1057296791
H	-0.3793339683	-0.4176348786	-3.9985510260
H	-0.8641203565	1.4662565993	-1.9852433458
O	-0.6261149358	0.1055372709	0.6731274243
C	-2.8883144623	0.0970886789	1.1672224836
C	-2.6915448446	2.4269496692	-0.1362268319
C	-2.7984616045	1.6119807176	1.2407511285
H	-3.5810368144	3.0770355787	-0.1593931578
H	-3.6887024386	1.9725754973	1.7666272540
H	-1.9247619849	1.8880064202	1.8400500491
C	-1.4565634275	3.2991489806	-0.3237638317
C	-1.4770865925	4.2798928043	-1.3341577527
C	-0.2492556989	3.0844764624	0.3437307883
C	-0.3286034156	4.9734864771	-1.6969531398
H	-2.4098325001	4.4803661604	-1.8702483133
C	0.9158531750	3.7708896294	-0.0051608602
H	-0.1867950138	2.3277703343	1.1198540218
C	0.8911360430	4.6972963822	-1.0534422407
H	-0.3412498236	5.7153272756	-2.4975507849
H	1.8373255319	3.5277336730	0.5202610234
O	1.9863944950	5.3480138989	-1.5088426601
C	3.2510149647	4.9348483945	-1.0242658009
H	3.3667954845	5.1452261108	0.0531146882
H	4.0010902529	5.5078180213	-1.5850871533
H	3.4123502113	3.8555620091	-1.1878271531
H	0.8078390604	-0.0091620010	0.2777505252
N	1.8639556458	0.1357889777	0.0800716792
S	2.1808752511	0.5097837381	-1.4857320879
O	3.0859660142	2.0769216790	1.5557472636
O	3.5449462868	1.0028981111	-1.6181477928
O	1.0466153889	1.1977446535	-2.1046413523
P	2.8092150668	0.6346867333	1.4065537272
O	1.9870944069	-0.0847564280	2.6065685285
O	4.1552399208	-0.2322209087	1.3555258471
C	0.8897275715	0.4304396780	3.3001208067
C	4.3280845688	-1.5469108280	0.9486164341
C	-0.0934770416	-0.4848003595	3.6753542534

C	0.7783087436	1.7858118753	3.6123726002
C	3.5267689134	-2.5801959110	1.4369771811
C	5.3710449249	-1.7941420854	0.0566377921
C	-1.2306515753	-0.0305141397	4.3424810080
C	-0.3664721459	2.2253147184	4.2853173654
H	1.5656324920	2.4779285801	3.3169004681
C	3.7749860041	-3.8859930689	1.0043187839
C	5.6109321971	-3.1036731367	-0.3613101964
C	-1.3739833261	1.3272903763	4.6455705418
H	-2.0160693748	-0.7423631086	4.6017112230
H	-0.4677924539	3.2870951575	4.5208267057
C	4.8116744748	-4.1519210572	0.1057180286
H	3.1578576356	-4.7024657995	1.3868482170
H	6.4230740061	-3.3040400469	-1.0637903498
H	-2.2709089648	1.6833644991	5.1563845002
H	5.0001098118	-5.1750804409	-0.2262778446
C	-3.9738315402	-0.4940614685	1.9689211700
O	-5.0487525356	0.0388358551	2.1740994786
O	-3.6715786812	-1.7037310885	2.5006403783
C	-4.7207990608	-2.3626768297	3.1995296541
H	-5.5947536146	-2.5160218255	2.5476982548
H	-5.0436274276	-1.7743640622	4.0717672783
H	-4.3127316454	-3.3294614405	3.5198975482
C	-1.5640603352	-2.0460383133	0.5520913309
C	-0.3255024382	-2.6851220562	0.7222690914
C	-2.6259142273	-2.7684563212	-0.0139494926
C	-0.1558011195	-4.0192624810	0.3559016597
H	0.5117766989	-2.1226580266	1.1329990317
C	-2.4532666396	-4.0976469421	-0.3963672414
H	-3.5849108686	-2.2815852431	-0.1849611190
C	-1.2199161591	-4.7294645345	-0.2068943344
H	0.8166486755	-4.4970337544	0.4910518114
H	-3.2823008105	-4.6400388242	-0.8563942495
H	-1.0858318598	-5.7708128832	-0.5093803899
H	5.9616273991	-0.9527293820	-0.3076403363
C	2.1135619089	-1.1917881055	-2.2827004373
F	0.9913908553	-1.8045109459	-1.9187994914
F	3.1537373174	-1.9276412367	-1.9322008229
F	2.1142121174	-1.0271834189	-3.6038325692
H	2.7373923589	-2.3605542262	2.1548540436
H	0.0198221229	-1.5357269928	3.4095849501
C	-5.3649710395	0.8295853604	-1.0932677982
H	-5.8320533649	1.4279992868	-1.8913134082
H	-5.9309085520	-0.1086635880	-0.9964623142
H	-5.4707116931	1.3556970392	-0.1353858047

TS2Bb

0 1

C	-0.0906823553	-1.4472837818	-1.5091633170
C	-1.6071317719	-2.2371604124	2.1898584459
C	-1.7300751764	-3.4839836444	2.8177457405
C	-0.6991295435	-3.9062813306	3.6578783146
C	0.4400015285	-3.1055864392	3.8777014538

C	0.5766289622	-1.8555634196	3.2765199126
C	-0.4640970613	-1.4398214257	2.4445094577
C	-2.4309924616	-1.5106241348	1.2196980703
C	-1.7543716469	-0.2635480799	1.0193015240
N	-0.5968063396	-0.2702606891	1.7030650802
H	-1.8040753125	-1.7673279800	-0.0980620454
H	-2.6080186732	-4.1118776364	2.6490600424
H	-0.7717957687	-4.8770165880	4.1539315069
H	1.2365562296	-3.4740555868	4.5275467008
H	1.4682933058	-1.2442161133	3.4165442841
H	0.0928401168	0.5111750684	1.6367789529
O	0.4797536280	-0.2805840258	-1.5956364601
C	-1.5167256344	-1.4567692983	-1.4759157627
C	-1.9792027555	0.6984762725	-0.1111191690
C	-2.2252687568	-0.1030651758	-1.4274286073
H	-1.0342603752	1.2414699507	-0.2297429075
H	-3.2922238263	-0.3165695734	-1.5492600435
H	-1.9168081388	0.5371911333	-2.2653910932
C	-3.0260551141	1.7585739967	0.2057634004
C	-2.7838610871	2.6097204814	1.3004599312
C	-4.1720495972	1.9826417067	-0.5555100957
C	-3.6586934700	3.6364429132	1.6229362951
H	-1.8732520438	2.4755725122	1.8893055990
C	-5.0636182890	3.0194429136	-0.2482754446
H	-4.3913697101	1.3596546281	-1.4228974427
C	-4.8103599458	3.8546064023	0.8461672757
H	-3.4695333382	4.2998097169	2.4687875612
H	-5.9448943514	3.1615242595	-0.8729919943
O	-5.6036831551	4.8878056429	1.2266707253
C	-6.7709997235	5.1569033973	0.4870440089
H	-7.4710682460	4.3019124643	0.4984650506
H	-7.2544194149	6.0197539631	0.9640572086
H	-6.5421660171	5.4103199892	-0.5642497176
H	1.4137741259	-0.1801045489	-1.1355076994
N	2.5185153074	0.3655924639	-0.1640640332
S	3.2805189554	-0.6522256665	0.8377035275
O	0.9163164581	1.9215538755	1.3520592891
O	3.7404220313	-0.0016917658	2.0707548804
O	2.5721515183	-1.9284814513	0.9253319700
P	2.0225947213	1.8495376163	0.3442962629
O	1.6050751682	2.5170790738	-1.0868363821
O	3.2631123502	2.7473375054	0.8474007673
C	0.4132288113	3.1764571397	-1.3430666172
C	4.5597843916	2.7093698845	0.3573402034
C	-0.2682131926	2.8268515950	-2.5109507720
C	-0.0849982393	4.1600684100	-0.4858012943
C	5.5883538780	2.6959979765	1.2990023567
C	4.8279256333	2.7104836970	-1.0129044738
C	-1.4703683725	3.4672326039	-2.8183219793
C	-1.2930129574	4.7865681715	-0.8025290966
H	0.4551368486	4.4108598894	0.4268854660
C	6.9118744635	2.6938653641	0.8587292810
C	6.1589251192	2.6965341013	-1.4388980687
C	-1.9899118717	4.4432963866	-1.9627846410
H	-2.0099925966	3.1910603683	-3.7274123176

H	-1.6988102859	5.5393505652	-0.1242061791
C	7.2018278275	2.6919469959	-0.5095564798
H	7.7224135542	2.6799939133	1.5911002092
H	6.3778086914	2.6924816140	-2.5093313517
H	-2.9416847512	4.9256300049	-2.1921821498
H	8.2395542502	2.6819865947	-0.8501374780
C	-2.3099906757	-2.5399867355	-2.1374217847
O	-3.5221532594	-2.5617973251	-2.1536381870
O	-1.5618533322	-3.4862212946	-2.7251694073
C	-2.2641181610	-4.5824304326	-3.3064665966
H	-2.8682155713	-5.1046139620	-2.5492720333
H	-2.9338246624	-4.2364625051	-4.1076420240
H	-1.4956604237	-5.2524905182	-3.7100379021
C	0.7663288977	-2.6261494245	-1.3605246547
C	2.0079482427	-2.6586798478	-2.0173139706
C	0.4083191854	-3.6790437756	-0.5006716277
C	2.8707793560	-3.7346635982	-1.8288398166
H	2.2936645877	-1.8365114096	-2.6748771985
C	1.2842132226	-4.7388223922	-0.2966188703
H	-0.5300235639	-3.6383267729	0.0516367730
C	2.5136733197	-4.7713491823	-0.9632169449
H	3.8361789175	-3.7510224066	-2.3378550436
H	1.0167639357	-5.5328184290	0.4034606761
H	3.2033389939	-5.6016588400	-0.7944308841
H	4.0058633942	2.7221280179	-1.7288857593
C	4.8410890457	-1.0660852976	-0.1427669990
F	5.8120969148	-0.2084719383	0.1364090823
F	4.6047282203	-1.0360998530	-1.4564882056
F	5.2358563888	-2.2926603603	0.1904578276
H	0.1486366320	2.0456442862	-3.1468137900
H	5.3294346529	2.6660279700	2.3574842776
C	-3.8995899576	-1.7753300267	0.9985154687
H	-4.4503287649	-1.6335559555	1.9425673615
H	-4.3303155316	-1.0988203245	0.2514066785
H	-4.0748449015	-2.8036403617	0.6514007422

TS3B

0 1

C	1.3047054914	2.7499780210	-0.7789036390
C	-3.9437100500	-1.7278823701	-1.0622002944
C	-4.9031541635	-2.3793012938	-0.2831038242
C	-4.4708321537	-3.0940087292	0.8399891033
C	-3.1117228578	-3.1508324999	1.1811903561
C	-2.1424052681	-2.4873676668	0.4205867008
C	-2.5820382983	-1.7924582242	-0.6961749713
C	-4.0259847751	-0.9159707736	-2.2759202898
C	-2.7558485766	-0.5314982756	-2.5768310818
N	-1.8482676074	-0.9525902386	-1.5729549777
H	-2.3905511501	0.0585780466	-3.4131823553
H	-5.9626380032	-2.3339838195	-0.5445203530
H	-5.2026342279	-3.6170300341	1.4602465390
H	-2.7938739508	-3.7144204502	2.0600274352
H	-1.1001621151	-2.4893072308	0.7268760902

H	-0.8743278306	-1.2313564189	-1.8838578968
O	2.0289845708	1.6433571277	-0.9751088503
C	-0.0627775368	2.7632280358	-0.9054635234
C	-1.3632941885	0.6927115699	-0.4876705944
C	-0.7880039003	1.5910229562	-1.5477698381
H	-0.0777404043	1.0102014005	-2.1530378068
H	-1.5713010692	1.9827455726	-2.2081375467
P	1.6783390944	-1.7554313833	-1.3642349249
O	0.6281941699	-1.5273553802	-2.4155352413
O	3.1323915866	-1.4061277244	-1.9583865323
O	1.8666483613	-3.3266470730	-0.9841157283
N	1.4055559910	-0.8609312947	-0.0274614037
S	1.5218926704	-1.2284647354	1.5244413530
O	0.1946025174	-1.2121792634	2.1657348872
O	2.4326458582	-2.3292865097	1.8539792233
H	1.5988126354	0.8085505662	-0.6598789395
C	-2.5806928580	0.9563650760	0.2373113028
C	-3.6980374781	1.6059252537	-0.3418151564
C	-2.7185277722	0.4095499050	1.5327763706
C	-4.8979006831	1.6896921329	0.3406898756
H	-3.6125556786	2.0430273512	-1.3339179857
C	-3.9188060937	0.4915358363	2.2257947013
H	-1.8586618095	-0.0869774158	1.9869156747
C	-5.0251565140	1.1186238723	1.6240636665
H	-5.7699150875	2.1822011465	-0.0924815689
H	-3.9903577276	0.0554523093	3.2207226992
O	-6.2370365998	1.2270244492	2.1983446040
C	-6.4557978325	0.6508513929	3.4713398458
H	-6.2794075100	-0.4384417216	3.4553544693
H	-5.8100118807	1.1085688015	4.2401816978
H	-7.5063614988	0.8439787394	3.7227608154
H	-0.6031417882	0.1653156950	0.0911889018
C	2.3305945461	0.3152279718	2.2052938426
F	3.5195385144	0.5234416874	1.6477721940
F	1.5525289120	1.3756940606	1.9742033109
F	2.4836187426	0.1699873265	3.5162159861
C	4.2290183244	-1.0392799899	-1.1798112121
C	4.7777900319	0.2248227043	-1.3927396326
C	4.7573715907	-1.9152793530	-0.2326272148
C	5.8852580717	0.6173966970	-0.6397861381
H	4.3109065643	0.8903415691	-2.1177308672
C	5.8616915758	-1.5062507570	0.5192104012
H	4.2953327838	-2.8894331379	-0.0768983131
C	6.4284807862	-0.2451598700	0.3180796805
H	6.3140607483	1.6103357659	-0.7940784207
H	6.2736441765	-2.1806705872	1.2731913316
H	7.2896200843	0.0688903445	0.9123032219
C	0.7929573031	-4.1629095336	-0.6953068396
C	0.7009278732	-4.7034838012	0.5884561877
C	-0.1449610364	-4.4699175750	-1.6850229175
C	-0.3530933209	-5.5737662683	0.8791566167
H	1.4382620220	-4.4138821751	1.3367388511
C	-1.1961636878	-5.3352928552	-1.3749686647
H	-0.0447944145	-4.0193384752	-2.6723548977
C	-1.3027362499	-5.8900905664	-0.0966324610

H	-0.4335022453	-6.0003891515	1.8818139401
H	-1.9392114892	-5.5730565511	-2.1395651098
H	-2.1298822459	-6.5625944705	0.1403581965
C	2.1719026173	3.9254196972	-0.5313851061
C	3.3470677793	3.7546207913	0.2201649448
C	1.9010115813	5.1811913781	-1.0977203497
C	4.2097131109	4.8299614128	0.4340332236
H	3.5748965236	2.7744931812	0.6363746639
C	2.7685992255	6.2511663991	-0.8910441113
H	1.0157803058	5.3125226250	-1.7213015881
C	3.9221164660	6.0811051075	-0.1178562352
H	5.1132114097	4.6882307064	1.0318629310
H	2.5490675636	7.2220769252	-1.3412361590
H	4.6007425626	6.9220643310	0.0448378105
C	-0.8651135740	3.8990575252	-0.4074968557
O	-0.5732078632	4.6637155415	0.4827387442
O	-2.0545219803	3.9964815100	-1.0665885203
C	-2.9471983993	4.9923351182	-0.5803304815
H	-3.2117570239	4.7999730120	0.4704298144
H	-2.4944127514	5.9927975112	-0.6465908180
H	-3.8435108478	4.9369296046	-1.2112010498
C	-5.2826780492	-0.5878910555	-3.0074713300
H	-5.9636276236	-0.0124632003	-2.3568463858
H	-5.0853345853	0.0072107947	-3.9104431296
H	-5.8205104157	-1.5028706560	-3.3059190729

TS4Ba

0 1

C	3.2919862070	-1.6820026285	2.3161736574
C	3.5952476377	-0.5905248416	1.4183196332
C	4.9390890826	-0.2443774116	1.1368887888
C	5.9305143506	-0.9835768113	1.7537023362
C	5.6471022759	-2.0624911456	2.6412813866
C	4.3461280788	-2.4126258717	2.9245693624
C	1.8954191728	-1.8269909101	2.3590572030
C	1.3581448983	-0.8873458733	1.4235690045
H	5.1853006046	0.5684043797	0.4581523675
H	6.9746808650	-0.7330187367	1.5505643411
H	6.4757634444	-2.6122222513	3.0916869498
H	4.1186233916	-3.2422277121	3.5967031488
N	2.4225563162	-0.0836286978	0.9609905018
C	2.1122008906	0.9029933190	-0.0877325160
H	1.1761341044	1.3704261080	0.2551899407
C	1.0822566011	-2.8084376511	3.1171541536
H	0.2610605480	-3.1771754162	2.4821908353
H	0.6088814788	-2.3265265027	3.9901996114
H	1.6830769120	-3.6567405888	3.4726648665
C	1.7896631011	0.1538807511	-1.3784350781
H	2.6744849647	-0.3255796766	-1.8125030854
H	1.4435386260	0.8829863402	-2.1212132772
C	0.7859383555	-1.9492055653	-0.0876292289
C	0.6569067512	-0.8498578573	-1.1797605199
H	-0.2387498559	-0.2875380942	-0.9021744875

C	3.1606124217	1.9859547001	-0.1978378632
C	3.9332395865	2.1884847073	-1.3509637404
C	3.3797516547	2.8262100856	0.8996287644
C	4.9045924743	3.1844749527	-1.3961501623
H	3.7848877596	1.5614813082	-2.2307522964
C	4.3525353458	3.8264100713	0.8740335876
H	2.7817153364	2.6887655219	1.8042364507
C	5.1285240277	4.0100063035	-0.2823288561
H	5.5084263475	3.3471769832	-2.2905540265
H	4.4937160917	4.4561397886	1.7517380813
O	6.0961006524	4.9469253273	-0.4144702478
C	6.3620593374	5.8155877342	0.6642139224
H	5.4786565442	6.4245671650	0.9265931638
H	7.1706557273	6.4821237869	0.3372073397
H	6.6925741664	5.2636931582	1.5623601996
O	-0.3754651054	-2.4528231593	0.3103938290
N	-2.4160319400	-0.7971278197	0.1781268335
P	-2.2658243058	0.7576109740	0.6651255522
S	-3.7262749557	-1.4371226884	-0.5125819793
O	-0.9193334932	1.0192457742	1.2642699843
O	-3.5256239745	1.0691625440	1.6260835490
O	-2.5983714733	1.7965675683	-0.5524433419
O	-3.3768756780	-2.7254350347	-1.1099661160
O	-4.5241635039	-0.4688560092	-1.2804009877
C	-4.8248404271	-1.9227664941	0.9411004752
H	0.4000664177	-0.3653850296	1.5470675353
H	-1.1674711419	-1.8039766424	0.2344125028
C	-1.7420897115	1.9458943156	-1.6254807048
C	-1.9307301172	1.1598733863	-2.7650742812
C	-0.7165208642	2.8929620846	-1.5573630499
C	-1.0734622536	1.3345774791	-3.8546492599
H	-2.7430262541	0.4328331329	-2.7690410962
C	0.1254039899	3.0665321487	-2.6590481169
H	-0.5979424406	3.4775386934	-0.6442203366
C	-0.0496527388	2.2873608065	-3.8080833301
H	-1.2075795894	0.7217085514	-4.7488052717
H	0.9301025085	3.8038803288	-2.6120863401
H	0.6149660202	2.4171940909	-4.6648580986
C	-4.3186552995	2.2089648969	1.6174721724
C	-5.3678105168	2.3025929093	0.7022156594
C	-4.0696098501	3.2114130080	2.5531534380
C	-6.1878352710	3.4316759880	0.7379445998
H	-5.5135141493	1.4992760058	-0.0209973835
C	-4.8995839174	4.3360877244	2.5776166849
H	-3.2352687231	3.0954238986	3.2470383583
C	-5.9585804226	4.4480961636	1.6723218661
H	-7.0124972709	3.5180289043	0.0264352378
H	-4.7156475797	5.1272555086	3.3085258019
H	-6.6057286506	5.3279341528	1.6937586977
F	-4.0819342005	-2.3833594062	1.9462184844
F	-5.5454724224	-0.8915672813	1.3720844473
F	-5.6537877765	-2.8839220655	0.5410652267
C	1.8156634425	-3.0278614051	-0.2827723898
C	1.4496407314	-4.3559432668	0.0022146099
C	3.1291268820	-2.7756160896	-0.7134226684

C	2.3745949368	-5.3920083213	-0.1173262040
H	0.4240177306	-4.5624063982	0.3039464235
C	4.0556082274	-3.8134843641	-0.8227998574
H	3.4505246639	-1.7723374602	-0.9656073375
C	3.6859924348	-5.1247258867	-0.5212721792
H	2.0661348294	-6.4171941723	0.1010175395
H	5.0730616327	-3.5886829312	-1.1497692939
H	4.4118273540	-5.9363428077	-0.6109669132
C	0.3319591421	-1.5425780618	-2.5039508068
O	1.1072470252	-1.6819531122	-3.4182310461
O	-0.9279709442	-1.9641170789	-2.4980228288
C	-1.4083640053	-2.6857465591	-3.6316804188
H	-2.4243455180	-2.9915813444	-3.3617055689
H	-1.4086979614	-2.0390365067	-4.5223657208
H	-0.7692647983	-3.5588858900	-3.8285807931

TS4Bb

0 1

C	-1.0810437705	-0.5589705082	-2.6469393673
C	-0.4675895402	-1.6622927656	-1.9470572476
C	0.9388606656	-1.8111787013	-2.0003055151
C	1.6626304896	-0.8689961972	-2.7064034187
C	1.0662894462	0.2460002809	-3.3561268031
C	-0.2968763863	0.4011810813	-3.3393469199
C	-2.4702376725	-0.6683389779	-2.5026976779
C	-2.6949327707	-1.7753395919	-1.6320022113
H	1.4557631403	-2.6327500465	-1.5211937598
H	2.7473788144	-0.9796345798	-2.7458201087
H	1.7022701217	0.9854811913	-3.8437418447
H	-0.7798966798	1.2588521853	-3.8061426964
N	-1.4639659453	-2.4226471205	-1.3892049783
C	-1.4186078202	-3.5375073433	-0.4135860615
H	-1.9976682666	-4.3491653269	-0.8867373614
C	-3.4984902568	0.2368681759	-3.0761801225
H	-3.5279828008	1.1679614292	-2.4855284531
H	-4.4981763345	-0.2141420943	-3.0803697842
H	-3.2284284211	0.5244847622	-4.1035175295
C	-2.0985926393	-3.1926804243	0.9341470639
H	-1.3891034151	-2.6896203976	1.5965609976
H	-2.3591382203	-4.1480803973	1.4133042825
C	-2.9667382473	-0.9526446584	0.2014372639
C	-3.3299259036	-2.2927398098	0.8780479887
H	-4.1758642591	-2.7532748731	0.3540220514
C	-0.0137449187	-4.0487539404	-0.1693590460
C	0.8443621286	-3.4119128470	0.7473199349
C	0.4806472713	-5.1310880294	-0.8997222452
C	2.1508575726	-3.8486808139	0.9132489712
H	0.5334115324	-2.5144948358	1.2853150416
C	1.8018834630	-5.5705043816	-0.7563079471
H	-0.1681457905	-5.6368952958	-1.6201866754
C	2.6493093740	-4.9205762600	0.1534143415
H	2.8198219100	-3.3510073581	1.6164217486
H	2.1537961391	-6.4100408736	-1.3547233087

O	3.9491873023	-5.2434085447	0.3592631447
C	4.5247873935	-6.2842332336	-0.3981227096
H	4.0223920615	-7.2503685201	-0.2129747872
H	5.5727718694	-6.3603430431	-0.0805095447
H	4.4915216773	-6.0672066062	-1.4807498240
O	-1.7768714258	-0.5698547507	0.5741772012
N	-0.2574822956	1.3879756337	-0.0662795651
P	1.1619728367	1.0071204774	0.6606150973
S	-0.5421627581	2.7110629347	-0.9312574915
O	1.1424835324	-0.2518185846	1.4494170831
O	1.5223183752	2.3561409565	1.5208594775
O	2.3089152620	1.0537066255	-0.5043322663
O	-1.8021990413	2.5203063169	-1.6627857916
O	0.6223669675	3.2632967119	-1.6247042888
C	-0.9924713286	4.0409449437	0.3448037738
H	-3.5919037253	-2.3912413926	-1.6798228571
H	-1.3268692766	0.3063962901	0.2292020978
C	3.5609041110	0.4737859889	-0.4595364362
C	4.6344500224	1.2359486584	-0.9241655307
C	3.7478478732	-0.8437615519	-0.0323585682
C	5.9102312330	0.6690624052	-0.9570226672
H	4.4515642102	2.2633364517	-1.2398185073
C	5.0273982095	-1.4016862581	-0.0752941948
H	2.8937370592	-1.4073550247	0.3303229357
C	6.1131387331	-0.6493477519	-0.5348173574
H	6.7530679610	1.2664971928	-1.3129758615
H	5.1641217891	-2.4378689823	0.2459591646
H	7.1133653913	-1.0875675981	-0.5653164771
C	2.8361770622	2.7299824060	1.7467212773
C	3.3111143349	3.8799131231	1.1147665616
C	3.6618494245	1.9512287976	2.5597103982
C	4.6414089604	4.2574666108	1.3089137616
H	2.6354211025	4.4357169919	0.4640951712
C	4.9931145930	2.3347919314	2.7372417222
H	3.2558871399	1.0487184603	3.0194207343
C	5.4853822627	3.4857783722	2.1149718939
H	5.0243636062	5.1544167974	0.8159475367
H	5.6504497807	1.7244557943	3.3607981310
H	6.5280858929	3.7797905351	2.2548741569
F	-1.5460850286	3.4963953272	1.4338926283
F	0.0741099803	4.7399117553	0.7115980244
F	-1.8809806735	4.8719904441	-0.2019607203
C	-3.8117962554	-1.9861239795	2.2982055078
O	-3.1007661385	-1.8528926076	3.2590000129
O	-5.1488056013	-1.8644826240	2.3293608562
C	-5.7147287231	-1.4568228602	3.5770255446
H	-5.3165235033	-0.4757445167	3.8753125415
H	-6.7972819976	-1.3965853356	3.4148088857
H	-5.4814530481	-2.1860250454	4.3667041977
C	-4.0512924338	0.0604528267	0.0573672670
C	-3.7916877705	1.3883198912	0.4195627158
C	-5.3378265732	-0.2853185462	-0.3903425190
C	-4.7846881680	2.3626078816	0.3053147564
H	-2.8137114030	1.6598766946	0.8056061232
C	-6.3302041482	0.6845872424	-0.4985010325

H	-5.5670602895	-1.3166172423	-0.6640482517
C	-6.0539859120	2.0153739241	-0.1577618138
H	-4.5510310362	3.3945531125	0.5742798218
H	-7.3248548171	0.4054283291	-0.8534729788
H	-6.8320166489	2.7764631068	-0.2521347635

TS4Bc

0 1

C	-0.9694225893	-1.3055544616	2.0777194130
C	-1.3307014308	-1.9213508780	0.8241041525
C	-0.5442996028	-2.9549424847	0.2751966417
C	0.5500485858	-3.3749797760	1.0061678981
C	0.8939946305	-2.8064407775	2.2689001803
C	0.1531074757	-1.7831944566	2.8074358198
C	-1.8555627193	-0.2441731648	2.3032640941
C	-2.6860290435	-0.1575436408	1.1346077302
H	-0.7588904411	-3.3789152705	-0.7050421268
H	1.1963893839	-4.1526505045	0.5957130388
H	1.7864881550	-3.1654227293	2.7795327151
H	0.4443775612	-1.3014533198	3.7401170392
N	-2.4596603071	-1.3228160497	0.3585230626
C	-3.0662982512	-1.4627254765	-0.9663879151
H	-2.7177074583	-2.4283942250	-1.3625608743
C	-1.8546906360	0.7007122299	3.4481376988
H	-0.9374402067	1.3123319448	3.4096896918
H	-2.7307354485	1.3606212243	3.4428259567
H	-1.8230313054	0.1490960864	4.4012929745
C	-2.5460325401	-0.3533971171	-1.8907933838
H	-1.4836342574	-0.5342615106	-2.1017238171
H	-3.0909615221	-0.3703647119	-2.8427200833
C	-1.7604768853	1.1257622904	-0.0046628752
C	-2.6774037766	1.0344941607	-1.2362782754
H	-3.7208881600	1.2082463448	-0.9453502757
C	-4.5845859952	-1.5259904346	-0.8211593504
C	-5.1385471407	-2.3740962401	0.1562808911
C	-5.4634927304	-0.7946639587	-1.6245433636
C	-6.5112492359	-2.4880591298	0.3212154219
H	-4.4724861162	-2.9502982513	0.8030147918
C	-6.8521202135	-0.8964457230	-1.4700969734
H	-5.0861756651	-0.1171421671	-2.3916195592
C	-7.3863514634	-1.7468183147	-0.4940930921
H	-6.9410138252	-3.1471910650	1.0775563439
H	-7.4987017957	-0.3047064084	-2.1170028552
O	-8.7087663871	-1.9168318327	-0.2619688127
C	-9.6381374407	-1.2071956813	-1.0501005269
H	-9.5467233296	-1.4640064878	-2.1205823223
H	-10.6357889595	-1.4983410687	-0.6965483675
H	-9.5211924451	-0.1145623207	-0.9362530024
O	-0.6091989821	0.5631684226	-0.2906531793
N	1.7251626550	0.3040370020	0.7230110736
P	2.4317099248	-0.3627520925	-0.5985199137
S	2.3328198830	0.6222972900	2.1596851548
O	1.5148413387	-1.1140120794	-1.4967331739

O	3.1602905341	0.9336974525	-1.2731569045
O	3.7670007127	-1.1976106394	-0.1593650479
O	1.2569566183	1.1572362678	3.0076074470
O	3.2191724137	-0.4030286650	2.7163856175
C	3.4561463592	2.1038855266	1.8984014654
H	-3.7036730370	0.2316367528	1.1678004085
H	0.2041769633	0.6031954119	0.3264328434
C	3.9919113595	0.8533444553	-2.3737870665
C	3.6028567355	0.1834777366	-3.5381904788
C	5.2271359819	1.4997288319	-2.2914202119
C	4.4734489254	0.1672166782	-4.6314289058
H	2.6324611042	-0.3133931517	-3.5651095826
C	6.0827100413	1.4814249601	-3.3944656874
H	5.4936528618	2.0001284741	-1.3595739828
C	5.7107599764	0.8146280040	-4.5665864988
H	4.1769900285	-0.3558121631	-5.5441230889
H	7.0499171900	1.9864012226	-3.3343557345
H	6.3844556375	0.7988950924	-5.4262841186
C	3.8054645533	-2.5674283701	0.0172080476
C	3.4967165651	-3.4351777724	-1.0346299326
C	4.1975807773	-3.0544813326	1.2659412046
C	3.5758764598	-4.8136951732	-0.8213863894
H	3.1805218218	-3.0221981963	-1.9915850639
C	4.2759335124	-4.4351103018	1.4629825995
H	4.4018608853	-2.3352594619	2.0586884135
C	3.9618867291	-5.3191201483	0.4247044287
H	3.3340394106	-5.4976119282	-1.6387552895
H	4.5804037558	-4.8223396132	2.4385321847
H	4.0231995172	-6.3980652470	0.5844117718
F	3.8498613962	2.5729218622	3.0794427443
F	2.8049780341	3.0761120478	1.2497582825
F	4.5309130525	1.7617519930	1.1906589225
C	-1.7771687080	2.4146166300	0.7547452283
C	-0.5681332836	3.0114042499	1.1340067473
C	-2.9859913384	3.0518303100	1.0785491670
C	-0.5664179085	4.2112922532	1.8458336017
H	0.3780753085	2.5457021799	0.8718644409
C	-2.9829814962	4.2538126350	1.7854164829
H	-3.9402300540	2.6066831086	0.7879183287
C	-1.7712487741	4.8348503834	2.1766753897
H	0.3884923970	4.6504595959	2.1414823969
H	-3.9297179014	4.7386434738	2.0344259989
H	-1.7697015421	5.7732145721	2.7360739570
C	-2.3932329524	2.1004554932	-2.2918155951
O	-3.1859382383	2.3680425120	-3.1630329489
O	-1.1902518231	2.6586639694	-2.1712427699
C	-0.8325922395	3.6453903466	-3.1401676354
H	-0.8608671532	3.2180826840	-4.1531168118
H	-1.5268011174	4.4975655521	-3.0960211238
H	0.1849636604	3.9622802604	-2.8847969167

TS4Bd

0 1

C	1.1538709528	0.5956368301	-2.3362888732
C	0.9345764759	1.6751712199	-1.4026943845
C	-0.3479852152	2.2568455096	-1.2818226950
C	-1.3435890650	1.7847013371	-2.1149238607
C	-1.1255040067	0.7500719024	-3.0701936971
C	0.1050469872	0.1521866916	-3.1850410152
C	2.4693309708	0.1385060688	-2.1699510862
C	3.0177936538	0.8910636550	-1.0872318787
H	-0.5620197193	3.0234488854	-0.5441436120
H	-2.3468013643	2.2040511956	-2.0245885978
H	-1.9623599451	0.4077247066	-3.6774476949
H	0.2702493250	-0.6813322294	-3.8669773891
N	2.1177646900	1.9323898174	-0.7680848629
C	2.4028207293	2.7728950272	0.4054459018
H	3.4587544391	3.0569498954	0.2865271475
C	2.2961763422	1.9477132990	1.6960457790
H	1.2483310918	1.6768189336	1.8801837264
H	2.6479225412	2.5627470495	2.5381379470
C	2.5798379691	-0.2798218891	0.5110400200
C	3.0950547038	0.6309538791	1.6535676214
H	2.8419809397	0.0547108736	2.5565066772
C	1.5819649606	4.0468580529	0.3942860517
C	0.6264971522	4.3548659400	1.3739086533
C	1.7562339865	4.9476510485	-0.6618609721
C	-0.1348101753	5.5165652503	1.2913460239
H	0.4465687496	3.6730490112	2.2051511112
C	0.9977597734	6.1144383637	-0.7643353946
H	2.4905884045	4.7230695070	-1.4398238734
C	0.0386338720	6.4058924797	0.2190080850
H	-0.8843458988	5.7563406272	2.0473196773
H	1.1597735564	6.7840680214	-1.6082454819
O	-0.7514915921	7.5052593455	0.2161094779
C	-0.6395647339	8.4267245926	-0.8447675282
H	0.3694852237	8.8738910482	-0.8947868641
H	-1.3717725703	9.2208350784	-0.6485272118
H	-0.8692691636	7.9591508237	-1.8189781636
O	1.2753204719	-0.2617112839	0.5739329804
N	-0.6709179837	-1.5603149134	-0.3920087045
P	-1.8607340676	-0.9771916518	0.5777009512
S	-0.7454544664	-2.6170526414	-1.5859838310
O	-1.5953966373	0.3673502536	1.1540608064
O	-2.0198647972	-2.1832090461	1.6679856888
O	-3.3088511774	-1.1270298790	-0.1613025541
O	0.6050790392	-2.7730806118	-2.1426212262
O	-1.8765091046	-2.4546457744	-2.5023372926
C	-1.0784614727	-4.2729132148	-0.7604964866
H	4.0786042290	1.0827462316	-0.9333727715
H	0.6574236486	-0.8963415691	0.0303815755
C	-3.9471500104	-0.1156106918	-0.8532818944
C	-4.2758287703	-0.3488303418	-2.1900425955
C	-4.2847164050	1.0846041312	-0.2213139202
C	-4.9523233078	0.6416754533	-2.9055516773
H	-3.9639476134	-1.2907413663	-2.6409472741
C	-4.9574956280	2.0677053121	-0.9512157768
H	-3.9971773100	1.2401659910	0.8176739184

C	-5.2918355158	1.8528756179	-2.2924918914
H	-5.2103516174	0.4668186099	-3.9530009943
H	-5.2209948292	3.0102713333	-0.4650768404
H	-5.8185964255	2.6254756001	-2.8572878592
C	-3.0159105635	-2.2141078907	2.6262927084
C	-3.7420775428	-3.3995901597	2.7576347339
C	-3.2682254685	-1.1161397576	3.4547303435
C	-4.7312787424	-3.4892635787	3.7386881801
H	-3.5193460099	-4.2266375084	2.0825028317
C	-4.2672157237	-1.2181971802	4.4266908427
H	-2.6826502643	-0.2057092018	3.3221620033
C	-4.9989798700	-2.4000594940	4.5745661814
H	-5.3014306626	-4.4153504079	3.8454067774
H	-4.4711514785	-0.3635399838	5.0766093030
H	-5.7765814034	-2.4725330515	5.3382956679
F	-0.2433079468	-4.4711680319	0.2661393776
F	-2.3300548171	-4.3305634151	-0.3100695963
F	-0.8950805915	-5.2472183694	-1.6477664954
C	4.5958540893	0.8383027970	1.6954140041
O	5.2369008834	1.5411635558	0.9426012670
O	5.1517432434	0.1452928177	2.6932831017
C	6.5742015333	0.2217964886	2.8125846867
H	6.8343430552	-0.3498434919	3.7107693570
H	7.0549312763	-0.2207170812	1.9271819675
H	6.9014447993	1.2670853553	2.9071658524
C	3.2688198068	-1.5875617218	0.2820357298
C	2.5167186141	-2.7663059115	0.3927395783
C	4.6402684400	-1.6804214576	-0.0144374208
C	3.1178569348	-4.0112502352	0.2008261003
H	1.4563458130	-2.7160155390	0.6296051406
C	5.2392171781	-2.9249404661	-0.1971378830
H	5.2409216893	-0.7798720289	-0.1423228657
C	4.4791869855	-4.0958012073	-0.0921867763
H	2.5060238315	-4.9121475139	0.2749663389
H	6.3034892518	-2.9822718050	-0.4372433498
H	4.9494862769	-5.0697029521	-0.2462396296
C	3.1250056135	-0.9844482233	-2.8890770348
H	4.2154986323	-0.9735338142	-2.7697088918
H	2.7298822970	-1.9417160764	-2.5097681963
H	2.8802105501	-0.9495988556	-3.9618030297
